# Supplementary material for: Overexpression of GmCAMTA12 Enhanced Drought Tolerance in Arabidopsis and Soybean
Source: Int J Mol Sci. 2019 Sep 29;20(19):4849. doi: 10.3390/ijms20194849 (PMC6801534; doi:10.3390/ijms20194849)
Supplement: Supplementary file 1 [file ijms-20-04849-s001.pdf]

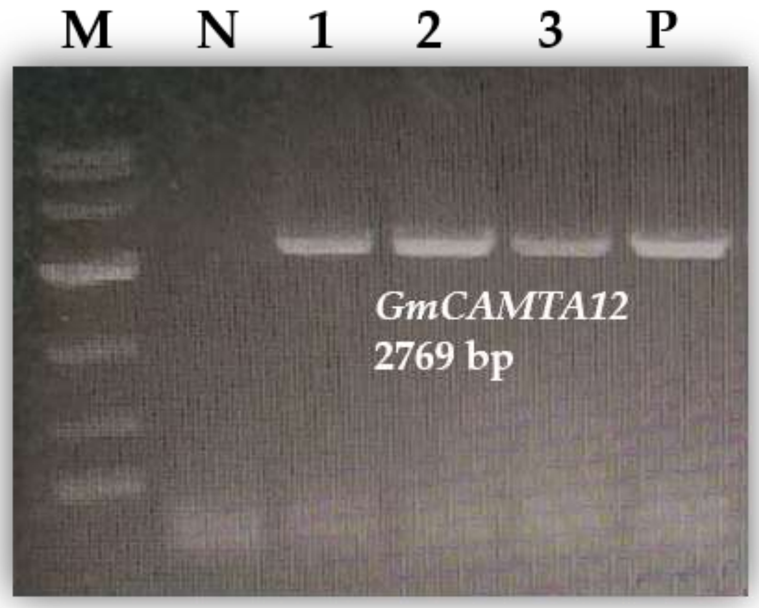

Figure S1. Gene-specific PCR of the EHA105 Transformants harboring OE construct.
M = DL 10,000 bp (TaKara), N = NTC (No template/negative control), 1-3 = EHA105 samples, P- Positive control (OE recombinant plasmid).

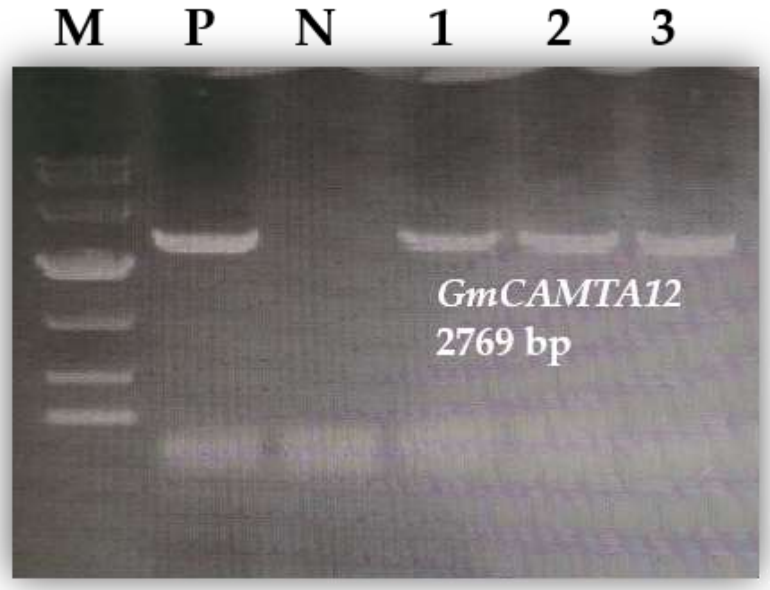

Figure S2. Gene-specific PCR of the K599 Transformants harboring OE construct.
M = DL 10,000 bp (TaKara), P- Positive control (OE recombinant plasmid), N = NTC (No template/negative control), 1-3 = K599 samples.

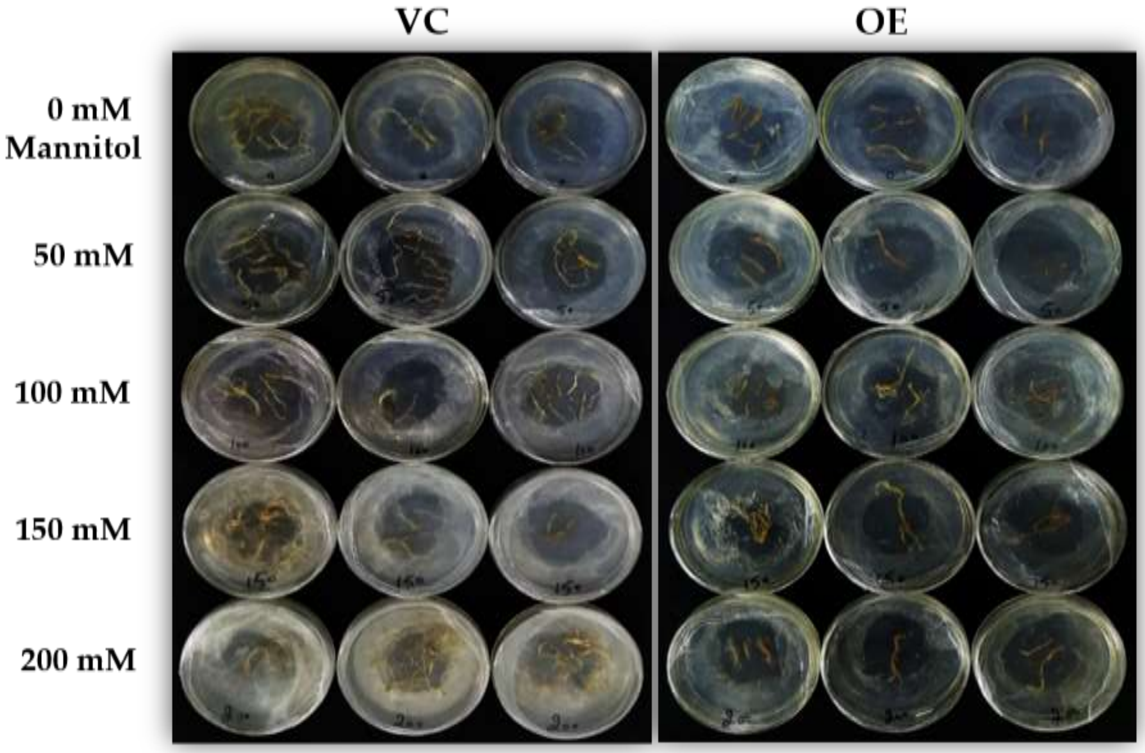

Figure S3. Hairy roots culture for fresh and dry weight. Comparison of growth efficiency (Fresh and Dry weight) of VC (Vector control) and OE (Overexpressing GmCAMTA12) hairy roots on MS with various mannitol concentrations.

Genomic sequences of Soybean CAMTA family

>Glyma.05G178200 (GmCAMTA1)
TTTTCTTCTCTTTGATAGTACAACACAACACAACACAACAATGCTATCACAGACAAGTTCCCGCG
TTGAAAACTTTTCTTTTTTCGTTCATTGGTATTTGGTACATCTATCTCCCTCTTAACACCCATTACAC
TCTCTCTCCGCGTTAAAAAAACTATCTGAACTTTCCTAGTTATTACAATTTATTACACAACCCATC
TCTCTTTTTTGTTTCGCTTAGATTTAGAAACACAGACTTTTCCAAAGTGCTCGTTTACCTCAAACCT
CAGAAAAATAAACATCGAGTTATCACTTGTTTTCTTTTGTCGGGAGACAAAAACATTGATCAGTT
CAGCTTCTCACTTCCATGGCTGAGGCCAGACACTACGTCCCCCCCTCTCAATTGGGTCAGCTTTCC
TTCACTTTAATTCCTCTTTATGTTTCGTTTCTCATCATTTCAAACTCTGAAAAAAGAAACCTTTTTTT
TAAAAGCAACCATGATAGTGAAGCATTGAAATGCCATCTCTTTATGTTTTAACATTTCCTTCGACG
TATTTATTGTTGTTAACTTTGTTTTCAATTTCTATGCTTTGATGGACACAGATATTAAGCAAATTAT
TTTAGAAGCACAGCATCGATGGCTACGTCCGGCTGAAATCTGTGCAATTCTCAGTAATCACAAGA
AGTTTCTAATTGCTTCAGAACCTGCACATATGCCACCAAGTATGTTGTTTCTCTTCTGCCAAAAGA
TAACACTAGTTACCTTAAGTTCTCAATAGCTTCATTTGGAGAAAAGGATTTAAAATATTGTTTTTT
TCTTTTATTTGAAGGGAGTGTAACGGGGAGGATTAGGATTTTAATCTATTCGAAAAATCTGTATCT
TATGAGGACTGAGACCTGCATGAAAACTTGACACAATTATGCCCAATATCTAATTGCAAAATGGT
CATTCTCAGGGTTCTTAGTGAGTGTTTCTGTGAGTGGTTGTAAAATTGATTTTGGTTAAGATTAATT
CTAAGCGAAGTGATTTATATTTGGATAATTTTATTCTAAAAGCAAGTTAACAAGAGAACTCAGTT
TATGATCTTTTATTCCATGCAAAAGTTACCTGAGGTTGATTTTACTCTTAATCAAAACTGGGCTGA
AAATCAATTTCTCAATGTGAGACCAAACATATGAGCATTTACCTAAAATTACTTTAGCTGATATTT

CAAGGTGACTTTAGTTGATCCTTCCAAACATGCACTTACCATATTTTTCCTTGACATTAGGTGGTTC
ACTTTTCTTATTTGATCGGAAGGTGCTGAGATACTTCAGAAAGGATGGCCATAACTGGAGGAAGA
AAAAAGATGGAAAAACAGTTAGAGAAGCTCACGAGAGACTAAAGGTAAATATGCTGCATGTAA
GGTTATAAGTCTTAAGCACTGGTACAATCACATCTACCATAAAATGAGAAGCAAAGGTCTCTGTA
AGCTTTATTTACTATCATTAATGTTAATTCTGCTTTGGGAAGTAAAATGTATTGAGAGAATCTTTCT
TCTTTATAAACTGTTGCATTGAATGATTCGTGATATGATGCTCTCAGGCTGGAAGCGTGGATGTGT
TGCATTGCTACTATGCCCATGGAGAAGAAAATGAAAATTTTCGAAGACGCACATATTGGCTACTT
GAAGAGTAAGTGTTTCTGTTTAATATTTTTGCAGTTCGGTATAAAAAATCCCCTTTTTATTTAATGT
TCTATCTTCATTTTCCTGATTCTGCTGTCTTCTCACCCCCTCCTAACTTTCTTCCTTCCCTCATTCTCG
GTCTCACAAACATACAGTAATATCCAGAAAGGATTGAAGAAAACCTGTGCTCTTCTTCTAATGTG
TTTGTTCTTGCTTGAAGATGGTGTGCTTTAAACTAGAACTAATGAGATATATCCTGTAAATGTTGA
TCACATTCTATTGGTTTAAACTGATTATAATAGACTTCTAGAAATTGCTAATTTGTTTCCATGGTTC
TTATATGTGGAATGGGCTCCTCTTCTCACCCTTTTTATTCAACATTGCTGCAGGGAACTCTCTCACA
TTGTTCTCGTCCATTATCGACATGTGAAGGTAACATGTTCTGATTTTATTAGTATTTTTTGCCTAATT
GTTATTTGACAAAAGCTCAAGAAGAATTGCTTACTTTTACATTCCCAGTATAATTAAGAGAACAA
TGAACTAGTAACATGTAATTGTGTTGCAGAGTTATATGTGAAGTAGGAAAGTAAAACATGGAAA
TTCCATCCTAACTTGAGTATGGTATTCCCCAAATCATGTAACTTGACTTTCCATGCTAATAATATA
TACTGCCTAGAAATAACAAAGCTGGCACTGCTTCCTTAATCAATACTTGGTCTGATAAGTTTCAA
CACCAGACGATTTTCTTGAATGCTAGATGCTATAAATTGGTGACATATTATTCCTTTCTATGACTA
TGCACAATTCTCTTTTCCCCTGTCTCAGATGTTGACTCTGTACTCTAATTCTTAAAAAATAAAAAA
GGCTAACATTCTCATCAGGAGTTTTGAACTATGTGATTAAATATAATTGTGATCTGCTATATCTTC
CCATCTATGAATAATTTCATTTTGGGCCTTCATTCCTTTCTCTCATTTGTCTTGTTCAAAATAATCA
GGGAACCAAGGCAAATTTTACATGTGCTAAAGAAAATGAAGAAACTCTTCCTTATGCTCAACAA
ACTGATAAAATTATGCCCAAAACAGAGATGGAAACTTCTTTATCATCTACTCTTCATCCACATAG
TTACCAGGTTCCTTCACAAACTATGGATAGAAGCATGAACAGTTCCCAAGCATCAGAATATGAA
GAAGCAGAATCTGGTATTTCTTTTGTGCACATTAATAATTTAAACTGTTCTGTTTTCAATTTTTCAT
GTCTAGAGGACATTCTGAGTAACCTTTTTGTTGACTTTGTTCTTCAGTATCACTTTTTCCTGTGTGTA
TTTAAATTTTTTTAACTTGATGTAATTTTTACAGCATTCAACAACCATGCAAGTTCTGAGTTTTATT
CTTTCCTTGAGTTAGAACGCCCTGTTGAGAAGATTACACCTCAACCTGCTGATTCTTATTCTCCTC
GGCCACTCACAAGTAAATTTCTTCTTTTTAAATTGTTTGAGTTTAACTTTGATGCATTGTCACTGTA
AATTTACACTATCACTCAGTCATACACTGTTGTACCATTCAAAGAGTATGACTTTTTTGAGCTAAT
TGTGAGAATCAAACCTTTTTAAACGATGTGACAGTGCATGATTGCAAGATACTGTAAAAAACTAA
TAATGCATCAAAATCAAATACCTTGTTTTATATCTTCCATTCATACTGGCCTTTTCATCAGGAAAG
TCTGTACCTAACATGAACTGCATTATTGAATCTGGGACAGATGATCAAGAGAAGTCACCTGTCAT
TCCTGGGGTGAATTATATCTCACTCACTCAAGACAATAAAATCAAAGACATTCATAATTTTGGAT
TGACATATGAATCCCCAAAACCCCTTGGCTTTTCCTCATGGGAAGGTATCTTAAAAAATAATGCT
GGAAGTCAACATGTGCCTTTTCAGCCTTTATTTCCTGGAACACAGCCTGACAACATGGGAATCAA
TAGCAAATTTTCCCAAGGACATGAGATAATGGTGCCATATTTGACCACCAGCATTGCTAAGCAGC
ATGAGAATGGAAGTCTCATACAAGCTGAAGGAAATTGGCAGGTATTGTGTACTAGTAAAATCGG
TACTTCAAATGCTAGTGGTACTCATTTGTTGCAATATTTTATGGTATATAATAATATTTAGTTCGAA
ACAAATAGCCAGGCTCTGCAGTATGTTCTGTTGGAGCTTTGACAAGGTATTATATGATTATATCTT
TATGTCAAAAGTCAAAAGACTTGAAAATAATGTCACTACAAAGTGATGTTGGTATGATGCTGCA
AATATTTTTGACATCTTACTTGTTTCGTGTTTCTGTAGAAAAAATATATATAAGCAATGGATTTTTC
TTTATGTGTGACTATTTTTTAAACATGTTACATGTTTCTTAATTTTTATTTCTTTCCTTTGAATTCATT
TAATTGATTAAAGCTTATACTTGTTAACAGGCTTATGATGTTGATTCTTTACGCATGTCCAGTTGGC
CTATAGATAGTGCTTATTCAGGCTCATCATGTGACATAACTTGCAGCAACCGTGAGCAGGAGGTT
AATGATGTTGATTTGCAGAAATCCTTGGAACAGTGTCTTCTACATCCATACAAACAAAACAAGGT
TTTCATGCAAAATGATCCTCAGGAAAAACTTTTGAATGAAAAAGAAAAGATAAAATCAGACCTA

GAGGCCAATAGAATCCTGGATGGAATAGAAGACACATATTTCACTTTTAAAAGGACTCTGTTAG
ATGGATCTCCAGCAGAAGAGGGTCTGAAGAAGCTTGACAGTTTCAACCAATGGATGAGTAAAGA
GCTTGCAGATGTGGAAGAGTCTAATAAACCATCCACTTCTGGTGGTTATTGGGATACAGTTGAAA
GCGAAAATGAGGTTGGCAACACAACTATTCCTTCACAAGGGCACCTGGACACCTATGTATTGGA
TCCATCTGTTTCCCATGATCAGCTTTTTAGCATTATAGACTATTCCCCAAGCTGGGCATTTGAAGG
GTCAGAAATTAAGGTATTTTCTTTTGATATTCTGCATTTGCTATCAATTTTGATCATATGTTCATTG
AATTGTTGCTGTTGCTTTTTTTCTTATTTGTCAGTTATAGTCTGATTATTTATTTCTTCACTAATGAA
AAAAATCGTACAGGTTATTATTTCTGGACGATTCTTAAGAAGTCAACATGAAGCAGAACAAGGT
AAATGGTCATGCATGTTTGGCGAGGTAGAAGTGCCAGCAGAGATCATTGCAAAAGGTGTTCTTTG
TTGTCATACTCCTCCACACAAGGCTGGGAGGGTACCTTTCTATGTAACTTGTTCCAATAGGTTAGC
ATGTAGTGAGGTGAGAGAATTTGATTTCCAAGTCAACTATACTCCAGAAGTTAACACTACAGGTG
AGAATAGAGGCAGCACTTTTGATACTTTTAGTATCCGATTTGGAGAACTGTTGTCCCTGGGGCAT
GCCTTTCCTCAGAATTCAGATTCAATTAGTGTTAGTGAGAAATCTCAACTGAGAAGTAAAATCAA
TTCTTTACTGAGGGAGGAGGAGGATGATTGGGACAAGCTACTGAAACTTACTCAAGAGGAAGAT
TTTTCTCCAGAAAATTTACAGGAGCAGCTGCTTCAAAATCTTCTGAAAGATAAGTTACATGCATG
GCTCCTTCAGAAAATAACAGAAGAGGGGAAAGGCCCTAATATATTAGATGAGGGTGGCCAAGG
TGTGCTTCATTTTGCATCTGCTCTTGGCTATGATTGGGCCCTAGAACCCACAATAGTTGCTGGTGT
GAATGTGAACTTTCGGGATGTAAATGGATGGACTGCTCTTCATTGGGCAGCATTTTGTGGCAGGT
GAGTACCTCGGCACTTGTGCATGAAACACATTTCACCAGAATTGAATTTATATGCAACATCCTTG
ACATTACATTAAATTAATACGATGGGTCACTGATATTAATTGCAACTTTGCAAGAGTGATAGTAT
GTGAGAAAAAATAGATTAGTGCTGAGAGAAAATATGTGAAATGTGCCTAAAAAGAAGTTAGAA
TGTTCTTACTTGTTGATACTCAATATACTTCATTATAGGTTTGCAGGATGGAACGCCATTGATGAA
GAAACTATAAGAAAAAATCTACTGAAAAAACCTATAGTATACTATGATTGGACGCACCTGAGTC
TTTTGTCATGAATGAAAACGTCTAGGATAAACTGACTAGAAAGAATCAATATATTGTTACTGCTT
TGAGTTTCTTTTTCATTTTCTTGCTTACTTCATCTTTCATTAAGTTCTTGCATCTTACTTTATCAGGGA
GCGCACAGTTGCTTTCCTCATCTCTCTCGGCGCAGCACCTGGAGCACTGACTGATCCATGTCCAG
AACATCCTTCTGGTAGAACACCAGCTGACCTAGCTTCTGCAAATGGACACAAAGGAATTGCAGG
GTATCTTGCTGAGTCTTCACTAAGTGCACACCTCACAACTCTCGATTTGAACAGGGACGCAGGAG
AAAATTCCGGAGCAAAAGTAGTCCAAAGAGTCCAAAACATTGCTCAAGTTAATGATCTTGATGG
TTTATCATATGAATTGTCATTGAAAGATTCATTGGCTGCAGTGCGTAATGCCACCCACGCTGCAGC
TCGTATTCATCAAGTTTTCAGAATGCAATCATTTCAGAGAAAACAACTGAAGGAATATGATGATG
ACAAACTTGGATTATCTGATGAACGTGCTCTTTCACTTGTAAAAATGAATATGAAATCACACAAG
TCTGGACCACGTGATGAGCCTGTCCATGCTGCTGCAGTACGAATCCAAAACAAATTCCGCAGTTG
GAAGGGAAGAAGAGAATTTTTAATGATTCGACAACGCATAGTAAAAATTCAGGTACATTTGTCT
CTAACATGTGATATGTGCAAATGGCATTATTATTTGTGTCTATACCTTTCTAGGCCTTTTGACTAAG
CATGTGTAGTATTTATTGTTTGTGTTGAATGTTTAATCTTATTCTCCTTTTGGCTCCTTTTAATTGGA
TCCAATTTATAACGTGTCTATTTCCATGGTATAGGCTCACGTGAGAGGTCACCAGGTCAGGAAAA
GCTGTGGGAAGATAATTTGGTCCGTTGGAATCTTGGAGAAGGTTATTTTACGTTGGCGTCGAAAA
GGTAGTGGTTTGCGTGGATTTAAACCGGAGGCCAATTCTGAGGGAACTATGATACAAGATGTATC
TTCAACAGATGATGACTATGATGTCTTAAAAGAAGGCAGGAAGCAAACAGAGCAAAGGTTGCA
GAAAGCCCTAGCTAGGGTGAAGTCAATGGTTCAGTATCCAGAGGCAAGAGACCAATACCATAG
GCTGTTGAATGTTGTAACTGAGATCCAAGAAAATCAGGTATTTTACTTATTCGTGTATATTTTTAA
AAAAGTTTCTCACTCGTACTGCTGAAAAAAGAAAAATAAAGTTTCTCAATTATCCTGCTTTAAAT
CATTTTCTCTTTTAGACAGTATATAACAGCAGTAGTTTTCAATCTCTAAAAAGATTTGGCTCTTTTA
AAGTTAATAAAGATAAAGTAATTTCAGTTTGAATTTTGAAAATTAATGGTTAATATTATAACACA
TGCATGATTTTATAATGCATGTGTATGTAATATATTGACTATTGATTTTTCCGTCACAATTCAAATT
ACTTTTTTTTTAAATGAGTTAATTCAGAGGAGTTGAATTCTCTGAAAAAGAGTGTAAAATAAATA
CCTGAATATGGTTTTTCTCCCTTTAGTATTTTACATAAAGCTCAATTTGCTTCTAGTAAAGCCAAAG

AGCCTTAAATGGCACTGGCTTGATCCCCGGATTGAATCCCCTCATTTGTAACCAAAAAATAAGTT
GCTTCTATCGAATGAAGCTGTTAGAGGTGTATATTGGATGGGCTACCTTAAAAAGCTTGTTAAGA
AATTATATGTATATTTGTTTAAACTACCAGGATATATATAAAATTGACATCATGCATAGTATCTTC
TTGTGACTGGCAGTAGAGCTTAGACTTCTGTATATTTCTTTTGCAGGTAAAGCATGAGAGCAGTTA
TAACAATTCAGAAGAACCGAGAGAATTCGGTGACCTCAACGATCTTGAAGCATTGTTGGACGAA
GATATTTTCATGCCTACAGCAACTTAGAAGTTAGCTTTCATTGTTCATTGTATGACTTTGAATTTCA
CAGCAGAGTTAGGTGATCAACTGTCTCACTTTGATTGTTATAGTAAATTAGCAATCTTAATTCTCA
TTCTTTTGTTATTAAGGGGTGATTTGCTAACATGTCACACAAAAATTAAAAAAAAATGCATAACC
ATT

>Glyma.08G135200 (GmCAMTA2)

TTTTCTTCTCTTTGATACAACAACACAACAATGCTTTCACACACAAGTTCCCGCGTTGAAAACTTT
TCTTTTTTCGTTCATTGGTATTGGTATTTGGTATATTCATCTCCCTCTTAGCACCCCTTATTAGTATT
ATTACACTCTCTCTCCGCGTTAACAAAACTATCTCTGAACTTTCCTAGTTATTACAATTTATTACAC
AACCCATCTCTCTTTCTGGTTTCGCTTAGATTTAGAAACACAGACTTTTCCAAAGTGCTCGTTCGTT
TACCTCAAACCTCAAAGAAAAAAAAAAAACATTGAGTTATCACTTGTTTTCTTTTTTGGGAAGACA
AAAACATTGAGTTCAGCTTCTCACTTCCATGGCTGAGGCCAGACTCTACGCTCCCCCCTCTCAATT
GGGTCAGCTTTTCTTCACTTTTAATTCCTCTCTATATATCATTTCTCATCATTTGACATCCGCAGAA
TTCAAACTCTGATTTTTTTTAAAGCAACCATGATAGTGAAGCATTGAATTGCCATCATATCTTTGT
GTTTTAACATTTCCTGTTACCTATTTATTGTTGTTGACTTTGTTTTGAATTTCTATGCTTTGATGGAC
CCAGATATTAAGCAAATTATTTTAGAAGCACAGCATCGATGGCTACGTCCGGCTGAAATTTGTGC
AATTCTCGGTAATTATAAGAAGTTTCGAATTGCTCCAGAACCTGCACATATGCCACCAAGTATGA
TTTCTCTTCCGCCAAAAGATAACACTAGTTATCTTAAGTTCTCAATAGCTTCATTTGGAGAAAATG
ATTTAAAAAATTGTTTTTTTATTCTTTTATTTGAATGGAGATGGGGAGGATTAGGATTTTAATCTAT
TCGAAAAATCTGTATCTTGTGAGGACTGGGACCTGCATGAAAACTTGACACAATTATGCCCTATA
TCTAATTGCAAAATGGTCATTCTCGGGGGTCTTAGTGAGTGTTTTGGTGAGCGTTTGTAAAATTGA
TTTTGGTTAAAATTAATTTTTTAAGTGAAAAATGATTTATATTTGGATAATTTATTCTAAAAGCAA
GTTAACAAGAAAAATCAGTTTATGATCTTTTATCCCAAGCAAAAGTTACCTAAGGTTGATTTTACT
CTTAATCAAATCTGCGCATCAATTTCTCAGTGTGAGACCAAACATGTGAGCATTTACCTAAAATT
ACTTTAGCTGATATTTCAACGTGACTTTAGTTGATCCTTCCAAACACGCACTAACTATATTTTTCCT
TGACATTAGGTGGTTCACTTTTCTTATTTGATCGGAAGGTGCTGAGACACTTCAGAAAGGATGGC
CATAACTGGAGGAAGAAAAAAGATGGAAAAACAGTGAGAGAAGCTCATGAGAGACTAAAGGT
AACTATGCTGCATCTAAGGTTATAAGTCTTAAGCACTGTTACAATCACATCTAACTTGGCCATAA
AATGAGAAGCAAAGGTGCTGTAAGCTTTATTTACCATCATTAATGTTAATTCTGCTTTGGGAAGTA
AAATGTATTGAGAGAATCTTTCTTCTTTATAAACTGTTGCATTGAATGAGTCCTGATATGATGCTC
ACAGGCTGGAAGTGTGGATGTGTTGCATTGCTACTATGCCCATGGAGAAGAAAATGAAAATTTTC
AAAGACGCACGTATTGGTTGCTTGAAGAGTAAGTGTTTCTGTTTAATATTTTCACAGTTCAGTATG
AAAAATCCCCTTTTTATTTAATGTTCTATCTTCATTTGCCTGATTCTGCTCTATCTTTTTCTCTCACCT
CCTCCTAACTTTCTGCCTTCCCTCATTCTCGGTCTCACAAACTTAAAGTAATATCCAGAAAGGAAT
GAAGAAAACCTGGGCTCTTCTTCTTATGTGTTTTTTCTTGTTTGAGGATGGTGTGCTTTAAACTAGA
ACTAATGAGATATATCCTGTAAATGTTGATCACACTCTATTGGTTTAAACTAATTATAATAACCTT
CTAGAAATTGCTAATTTGTTTCTATGGTTCTTATCTGTGGAATAGGCTCCTCTTCTCACCCTTTTTAT
TCAACATTGTTGCAGGGAACTCTCTCACATTGTTCTCGTCCATTATCGACAAGTGAAGGTAACAC
ATGTTATGATTTTATTAGTATTTTTTGCCTAATTGTTATTAGACAAAAGCTCAAGAAGAATTGCTTA
CTTTTACATTCCCAGTACAATTAAGAGAACAATGAACTAGTAACATGTAATTGTGTTGTAGAATT
ATATATTTAGTAGGAAAGTTAAACTTGGAAATTCCATCCTAACTTGAGTATGGTATTCCCCCAAA
TCATGTAACTAGACCTTCCATGCTAATAATATATACTGCCTAAAAATAACAAAACTGGCACTGCT

TCCTTAATCAATACTTGGTCTGATAAGTTTCAAAACCAGACGATTTTCTTGAATGATGCTATAAAT
TGGTGACATATTATTCCTTTCTATGACTATGCAGTATGCACAATTCTCCTTCCCCCTTTCTCAAATT
TTGACTCTGTACTGTGATCCTTAAAAAATAAAAAAAGGATAACATTTTCATCAGGAGTGAACTTT
TTTAACTATGTAATAAATATAATCGTGAGCTGCTATATCTTCCCATCTATGAATAATTTCATTTGG
GCCTTCATTCCTTTCTCTCATCTGTCTTGTTCAAAATAATCAGGGAACCAAGGCGAATTTTACAAG
TGCTAAAGAAAATGAAGAATCTCTTCCTTATGCTCAACAAACTGATAAAATTATGCCCCAAACA
GAGATGGACACTTCTTTATCATCTACTCTTCATCCACATAGTTACCAGGTTCCTTCAAAAACTGTC
GATACAAGCATGAACAGTGCCCAAACATCAGAATATGAAGAAGCTGAATCTGGTATTTCTTTTGT
GCGCATTAATAATTTAAAATGTTCTGTTTTCAATTTTTCATATCTAGAGGGCATTCTGAGTAGCCTT
TTTGTTGACTTTGTTCTTCGTATCACTTTCTTGTGTGTATTTAAATTTTTTAACTGTGTAATTTTTACA
GCATTCAATAACCATGCAAGTTCTGAGTTTTATTCTTTCCTCGAGTTACAACGCCCTGTTGAGAAG
ATTTCACCTCAACCTGCTGATTTTTATTCTCCTCGGCCACTCATAAGTAAATTTCTTCATTTTAAAT
TGTTTTTGAGTTTAACTTTGATGCATTGTCAGTTTGTCACTGTAAATTTACATTACCACTCTGTCAT
ACATTGTCGTATCATTAAATGAGTATGACTTTTTTGAACTAATTTTAAGAATCAAACCTTTTTAAA
TGTTGTGACAGTGCATGATTGCATGATAGTGTAAAAAACTTTACACTAATATTGCATCAAAAATC
AAATACCTTGTTATATATCTTCCATACATACTGGCCTTAATCAGGAAAGTCTGTACCTAACATGAA
TCATATTATTGAAACTGGGACAGATGATCAAGAGAAGTTACCTATTATTCCTGGGGTGAATTATA
TCTCACTCACTCAAGACAATAAAAACAAAGACATTCTTAATGCTGGATTGACATATGAATCCCCA
AAACCCCTTGGCTTTTCCTCATGGGAAGGTATCTTAGAAAATAATGCTGGAAGTCAACATGTGCA
TTTTCAGCCTTTATTTCCTGGAACACAGCCTGATAACATGGGAATCAATAGCAATTTTTCTCAAGG
AGAAGAGATAATGGTGCCATATTTGACCACCAGCATTGCTAAGCAGCATGAGAATGGAAGTATC
ATTAAAGCTGAAGGAAATTGGCAGGTATTGTGTACTAGTAAAATCAGTACTTCATCTGTTAGTGG
TACTTATGTTATATAAGAATATTTAGTTCTAAACAAATATCCAGGCTCTGCAGTATGTTCTGTAGG
AGCTTTGACAAGGTATAATATCTTTATGTCAAAAGTCAAAAACTTGAAAATAATGTCACTAAAAA
GTGTTGTTGGTATGATGCTGCAAATATTTTTGACATCTTAATGACTTGTTTCGTGTTTCTGCAAACA
TATAATCAAGCAATGGATTTTGCTTTATGGTTGACTGTTTTGTAAACATGTTACATGTTTCTTTCCT
TCAAACTCATTTAATTGATTAAAGCTTAATCATCCTTGTTAACAGGTTTATGATGTTGATTCTTTAC
GCATGTCCAGTTGGCCTATAGATAGTGCTTATTCAGGCTCAACATGTGAGGTAAGTTGTAGCAAC
TGTGAGCAGGAAGTTAATGATGTTGATTTTCAGAAATCCTTGGAACAGTGTCTTCTACATTCACAC
AAACAAAACAAGGTTCTCATGCAAAATGATCTTCAAGAAAAACTTTTGAATGAAAAAGAAAAG
ATAAAATCAAATCTAGAGGCCTATGGAATAGAAGACACATATTTATCTTTTAAAAGGACTCTGTT
AGATGGACCTCCAGCTGAAGAGGGTCTGAAGAAGCTTGACAGTTTCAACCAATGGATGAGTAAA
GAGCTTGGAGATGTGGAAGAATCAAATAAACCATCCACTTCTGGTGGTTATTGGGATACAGTTGA
AACTGAAAATGAGGTTGGCAACACAACTATTCCTTCCCAAGGGCACCTGGACACCTATGTATTGG
ATCCATCTGTTTCCCATGATCAGCTTTTTAGCATTATTGACTATTCCCCAAGCTGGGCATTTGAAG
GGTCAGAAATTAAGGTATTTTCTTTTGATATTCTGCACTTGCCATCAGTTTTGATCACATGTTCATT
GAATTGTTGTAGTTGCTTTTCTGCTTATTTGTCAGTTATATTCTGATTATTTATTTCTTCATTTATGAA
AAAAAAATGTATAGGTTATCATTTCTGGAGAATTCCTAAGAAGTCAACATGAAGCAGAACAATG
TAAGTGGTCATGCATGTTTGGTGAGGTAGAAGTGCCAGCAGTGATCATTGCAAAAGGTGTTCTTT
GTTGTCATACACCTCCACACAAGGCTGGGAGGGTACCTTTCTATGTAACTTGTTCCAATAGGTTAG
CATGTAGTGAAGTGAGAGAATTTGATTTCCAAGTCCACTATACTCCAGAAGACACTACAGGTGA
GAATAGAGGAAGCACTTTTGATACTTTTAGTATCCGATTTGGAGAACTTTTGTCCCTGGGGCATGC
CTTTCCTCAGAATTCAGATTCAATTAGTGTAAGTGAGAAATCTCAACTGAGAAGTAAAATCAATT
CTTTATTGAGGGAGGATGATGATGATTGGGACAAGCTACTAAAACTTACTCAAGAGAAAGATTTT
TCTCCAGAAAATTTACGGGAGCAGCTGCTTCAAAATCTTCTGAAAGATAAGTTACATGCATGGCT
CCTTCAGAAAATAACTGAAGAAGGGAAAGGCCCTAATGTATTAGATGAGGGTGGCCAAGGTGTG
CTTCATTTTGCAGCTGCTCTTGGCTATGATTGGGCCCTAGAACCCACAATAGTTGCTGGTGTGAAT
GTGAACTTTCGTGATGTAAATGGATGGACTTCTCTTCATTGGGCAGCATTTTGTGGCAGGTGAGTA

CCTCAGTACTTGTGCATGAAACACATTTCATCAGAATTGGATTTATATGTAACATCCTTGACATTA
CATTAACTTAATAGGATGGGTCACTGATATTAATTGCAATTTTGCAAGAGCGTTAGTATGTGAGG
AAAAATAGATTACTGCCGAGAGAAAATATGTGAAATGTGCCTAAAAGAAGTTAAAATGCTCTTG
TTGATACTTGATATACTTCATTATATGTTTGAAGGATGGAATGCGATTGATGAAGAAATTATAAG
AAAAAATCTACTGAAAAAACCTATAGTATACTATGATGGGATGCACCTGAGTCTTTTGTTATGAA
TGAGTTTCTTTTTTCATTTTCTTGCATACTCTTGTCATCTTTCATTAAGTTCTTGCATCTTACTTTATC
AGGGAGCGCACAGTTGCTTTCCTCATCTCTCTCGGCGCAGCACCCGGAGCACTGACTGATCCATG
TCCAGAACATCCTTCTGGTAGAACACCAGCTGACCTAGCTTCTGCAAATGGACACAAAGGAATT
GCAGGGTATCTTGCTGAGTCTTCACTAAGTGCACACCTCACAACTCTCGATTTGAACAGGGACGC
GGGAGAAAATTCCGGAGCAAAAGTAGTCCAAAGACTCCAAAACATTGCTCAAGTTAATGATCTT
GATGGTTTATCATATGAACTGTCATTGAAAGATTCACTGGCTGCAGTGTGTAACGCCACCCAAGC
CGCAGCTCGTATTCATCAAGTTTTCAGAATGCAATCATTTCAGAGAAAACAACTGAAGGAATATG
ATGATGACAAACTTGGATTATCTGATGAGCGTGCTCTTTCACTCATAAAAATGAATGTAAAATCA
CACAAGTCTGGACCACGTGACGAGCCTGTCCATGCTGCTGCAATACGAATCCAAAACAAATTCC
GCAGTTGGAAGGGAAGAAGAGAATTTTTAATGATTCGACAACGCATAGTAAAAATTCAGGTACA
TTTGTCTCTAACATGTGATATGTGCAAATGGCAATATTATTTGCGTCTATACCTTTCTAGGCCTTTT
GGCTAAGAGCATGTGTAGTATTTATTGTTTGTGTTGAATGTTTAATCTTATTCTCCTTTTGGCTCCTT
TTAATTGGATCCAATTTATAACGTGTCAATTTCCATGGTATAGGCTCACGTGAGAGGTCACCAGG
TCAGGAAAAGCTGTGGGAAGATAATTTGGTCCGTTGGAATCTTGGAGAAGGTTATTTTACGTTGG
CGTCGAAAAGGTAGTGGCTTGCGTGGATTTAAACCGGAGGCCAATTCTGAGGGAACTATGATAC
AAGATGTATCTTCAACAGATGATGACTATGATGTCTTAAAAGAAGGCAGGAAGCAAACAGAGCA
AAGGTTGCAGAAAGCCCTAGCTAGGGTGAAGTCAATGGTTCAGTATCCAGAGGCAAGAGACCA
ATACCATAGGCTGTTGAATGTTGTAACTGAGATCCAAGAAAATCAGGTATTTTACCTATTTGTGTA
TATTTAAAAAAAAAAAATTCTCACTCGTATCACAGAAAAAAGAAAAAAAAAAGCTTCTCAATTATC
CTGCTTTAAATCATTTTCTCTTCTAGACAGTATATAACAGCAGTAGTTTTCATTCTGGAAAAAGAT
TTGGCTCTTTTAAAGTTAATAAAGATAAAGTAATTTCAGTTTGCATTTTGAAAATTAATGGTTAAT
AATATAACACATTCATGATTTTATGATGCTTGTGTATGTAATATATTGACTATTGATTTTCCCGTCA
CAACTCAAATTACTTATTTTATTTTTTTAAATGAGTTACTTCAGAGGAGTTGAATTCTCTAAAAAA
GAGTGTAAAAAAAAAAACCTGAATATGGTTTTTCTCCCTTTAGTAATTTTCATAAAGCTCAATTTGC
TTCTATTAAAGCCAAAGAGCCTTAAATGGCACTGGCTTGATCCCCGGAGTGAATCCCCTCATTTG
TAACCAAAAAATAAGTTGCTTCTATCGAATGAAGCTGTTAGAGGTGTATATTGGATGGGCTACCT
TAAAAAGCTTGTTAAGAAATTATATGTATATTTGTTTAAACTACCAGGATATATATATATATATAT
ATATATATAATTGACATCATGCATAGTATCTTCTCGTGACTGGCAGTAGAGCTTAGACTTCTGTAT
ATGTCTTTTGCAGGTAAAGCATGAGAGCAGTTCTAACAATTCAGAAGAACCGAGAGAATTCGGT
GACCTCAATGATCTTGAAGCATTGTTGGACGAAGATATTTTCATGCCTACAGCAACTTAAAATTT
CATTGTTAAGGGGTGATTTAGAATTTCACAGCTGAGTTAGGTGATCAACTGTCTCACTTTGATTGT
TATAGTAAATTAGTAATCTTAATTCTCATTCTTTTGTTGTTAAGGGGTGATTTGCTAACATGTCACA
CAAGAATTAAAAAAAAAAATGCATAACCATTAAAACCATTCTTACAATGCAAAATAAACATGTCA

>Glyma15G053600 (GmCAMTA3)

AATTGATAATACTAGTAGTAAAAGTTACTCTGCTGTATAGTATAAATACTAAATATACAGTATCA
GTTTTTTCACTTTATCGCGCGTCAGTTGAGAAAAGGAAAAGAAAGGATAAAAAGAAAAGAAAA
AAAGTCTCTGTCGTCTCTCTCGTCCTTCGTCGTTCGCGTTGATCTCAGATTCAGTCTTCGCCATGGC
GGAGGGTGCTTCTTACGGATTGCGTCGTCCATTAGGTCATTTTCTAATTGCTTTTTTCTTCTTTTTTT
TTTGCTTCATAATTTTTCATTCAATGCTTCGATCCTGTGAGTTTAATTGTTATTTTCGACACGATTTC
TTGTCACGGAGCTTCAGCTATTGAATTCTGTTTCTATCGTTGAGTGGGTTCCTGTTGTTGGGACTAA
GGTTCAGTTACTAATAATGGCTTCGCTAATGAATGGACCCAAGCTTCGGTCCTTACTGAGGTTGA

CAAAAACTGTGCTCAATTGATTTGGGGAATTAACTATTTTGGGATTTTTTTCAGTCCAATTAGGCG
TTATAGGTGTTAAAACGTCTTTGGCACTAGGTTTGAACGACATTAACTATTGATAGTACTGCTTCA
CTGCATCAATTTTCCACTTGTTCGGGTTCTGACTGGTGCAACTAACTAATATGCAGACCCAATCCC
CCTCTCCCCCCAAAATGAGCGAACCTAGTATTGTCAAATTTGTAACTATTTCAAAATGATTTAGTT
GGATGTAGAAAGCACCATGCATTTGAGGGATTTTCCTTGCAGAGAAAAACTGAAAGAAGCATCA
ATTTTTAGAAAGTTAGATTTATTCATGGAGACAAAATTAAGGACTATCATTTGTCCATTTTTTTATG
AATCCTGTTCTTATTGGTGCAAAATGTAAAAGAAAAAAGTTGGATTCAGACTAGTGGTATTTGGT
AAGGTCTGACATGAATATATGTTGAGCCCTGGCTTCATATGGTAAGGCGGCTGTTTTAACTGTTCT
TGCTTCCTCTTATGGTGTGTGAAGAAGTTTGAAAATACACCGGGGAAAGCAAAAGTGAATTTGAG
TGAAGGATACATTGCTTCAAAATATAACATACACAATGGCCTTGTCGGCAAATTAATTCTATCAA
TTCATAGTGGCCATATAATGCTGGGGTTCTTCTTCTTCTTGCCTTCTGATGTGCCTTGCGTCTTACCT
CTGTGTTGATTACAAGCATATTCTTCTGCGGTTCTGCCTCTCTCATTCTCTGTATACTAATACCATT
AATATAGTTATCAACAATCCCTAAAATTGTAGTCACTGTGCAGTATTTATTTTAACTCATAATAAC
AGGGTATTTATAGATCACCCTTATGAACATAAGGAAAGTTACCCATATAAATCTATGAATATTTA
TTTTCAGTTAAAGTTTAGTTCAAAGTTATCATATACAAGATATTTTCCTTATACATCATTTTCATCA
CATGTATATCTTCATATATAATTTTGCCTCTTTTGGTAGGATCTTTCGATTTATGATATTTCATCCCC
CTCCCGATCTCGTGTGAGATCTCGATTCTGACAACGTTGGTCAAAACAACTTTTTGGACAAAAGC
TCCACAACATTTTGATTTTAAACTATTTTAAACACAAGCTTTTCCAAATAATCACATAAAAAGACT
AAAACCGAACTGATTCTGACGAAAGGAATATATTTGGTAGATAGATTGATTTTTGGGGGGACTAA
CAGGCTGCTTAATTTATGTACTGGATACTTTGACCTCAATATTGGACTCATGATTGACAATGTGGG
TGTTCTTTTCATATGCCAGTTTATCTTTCTTTTTTGATTCTGTAACAAAAATATTCTTATTGAAGCAT
GTCACTTTTGTGGCCATGATTTGATTCTTTAAGCTTGCTTGAATAATTGTATTTGTGTATGTGATATT
ATTCAAAAATCAAATATTTAACTGAATGCCTTATATCAATGTGCAGATATTCAGCAATTACAATTT
GAAGCCCAGCATCGATGGTTGCGACCTGCAGAAATTTGTGAAATTCTTCGTAATTATCGGATGTT
TCATATCACATCAGAACCTCATAACAGGCCACCAAGTATGGTTACTGCCTGTTACTGATAAATTA
ATTGGCACCTGTATATACTATTATATAATTTTTTACATATATCCATTGGATGGATTGCTTAAGAAG
CTTACACCAATACTGATTTTCAATGATGTGTTTGTTGCAACTGGGTGTTAATGAACTTTCTGGCCG
AAATTATTAACATCTGTCTATGTGCCGAACAATGTGTTTCAACATGGAAGTATTGCCCTCCTTGGG
TTCACTTAGCTGCATTTAATTCCTTTTAAAGATGTATCAAGCATTTTAATTTTGCTTTATCTGAACTT
AAGTTATTCATGTTCCAATAGATCCTATATATTCATTTCAAGGAATAGAGGCACATTGGGTAATTC
AGTAAATATTTTTTCATGAAATTTGGTAGCAAAAAAGCCCCTGATATGCTTATTGAATTATCTTCG
CAATTCAGTAGTTTTTATGATATTATTAGTAGTATTTTGTTGTTTTGCTAAAATCTTGTTTCGTTGTT
AACTTCAATCCATCTTTTAGCTCCTAATCCTATTCCTATCATTACTTCATGCTTACTAAGACATCAC
TTAATGACTTGTATTTCATGATTTCAGGTGGTTCACTTTTTCTTTTTGATCGAAAGGTTTTGAGATA
CTTTAGAAAGGATGGGCATAATTGGAGAAAGAAAAAAGATGGAAAAACTGTGAAAGAAGCTCA
TGAAAAGTTGAAGGTAAGCTTATAATTTACTTCAGTTTTGAGTTTGTAGTTAATCAAATTTTCGTA
AGACTAGCGTATACATAGGATGATTGGAATTTTCTCCAATTGGAAAGCAAGTCTTTATACCATTA
TGCAATTATTCACTGGAGTTGTAACATCATTTAAGTAAATTTAGGTTGGAAGTGTTGATGTGTTGC
ACTGCTACTATGCCCATGGAGAAGAAAATGAAAATTTTCAAAGGCGAAGCTACTGGATGCTTGA
ACCGTGAGTATGAATTAATTTACTTACAAGACTTTGTGTATTGGAAATTAAAGTACATTCATATTT
CATGTGTTTGTTGCTTTGTAAGTTTCTTAGCAAGTACTGCATTGGTGCATTTGCTGTCATATCTCAT
GTCAATGTATAGATATAAGTTTTTAAGGGTGTTTCTTTATCCTCAAACTTAGTGAATAGAGTGTTA
GAGCTACATTTTTGGCTTTTCTTTGGTGTTTGTATGCTTCAAAGGAGTTGCTAGCTTAGACATGTTG
ATTGAGAAGCTTTACTGCATTCTTCTTCCCTGTTCTTTTTTATCTAATAAATTGTAGATAACAGTTTT
TAATCTTACCGGGTTGCAACTATTTTGCTGCATCTTACCTAATTTGAGTTTTCAATGTTCATCTACT
TAAATATTTTTTGTCTTACAATTAATCAGAATATGGTTTTCTTGTTATTTTTCTTTTATTGCAGGGAT
ATGATGCACATAGTATTTGTCCATTACTTGGAAGTTAAGGTATTTTGCTCATTTCTTCATATTTTTTC
TGTTATTATTCTTTCTTGTTAGTTGTTATTACCAATGGTTATGTATTTTTATCAATTATAAGTTATAA

CTCGCATTTCTAGCTTTTGAACTTTATCTATTTTTTACTTGAAACGTTTTACCTCCATAACTTGAGTT
TTGATGTTTTACAAAATATGATGATACATTTGAAGGGAGAAAGAATGAAACAACTGCAGTTCTTG
TAGGCTATGTTGAGCCATCATGAATTATTAGTGATATCATTGCAACTCATTTTGGGTCTTTATATTA
TTTAATAACCATGGCTGTAATTAATGAAGTATACACAGGATGAGTTTTTCATAATGCGTCTTGAGT
AGGAAACAGCCACTAAGCCATTTTTAGATATGATTTGTATGGCATCTAGATTGAGTTCAATTTGAT
CAATTGTCAATGTGATTTTTTTTAATACTATCAGTATTACCTAATGACATGTTAACATGTCATTAAA
AGAGTTTGAGTTCCTAGTTGTTGTGATAGTCAAACATGGGCCCAATGGGTCATATTGCACATCAG
CAGCATAAGCCCATGGATTAGAGAGAATATGTCAGGAAAAATAAGTAGAGTCAGGGTAAGAAT
GATGAGGTGGCAGAGAGAGAGAAAGGGGTGAGCAAATTGTGTGAGTGTTTCTGTTATATGGGGATT
GTGTAGGGCGAGTGTATGATGAGAGAATCATTTTCAGTTAGTGGCAGAGAAATTTCTATTCTCTTA
GGGAGCTGAGCTCTGTTGGCTGTAGGGATATTCATTTCTCTGCATTTTCCTTCTATATCAGAAATA
AAACATTGTTTTTCATTATTGATTCATTTTCCATGTCATTTGTTATTCTGTGATTTTGGCTTGCATAA
AAAATATGCCTTTTTAGCATTGATAAACCAGTGTGGCTAAGCTTAAGCATTATACCCATTGAAAA
TGTTGAATTGTATTATTTGCTCTTCAGCTGGTATACTATTTTTTTCCTGATCCCATTTCATTAAAAGT
CTCAATTGATGATATAAAAGATAATGATATGCTCAAATGGGCATGATAGTACATGTTCGGTTTGT
GGGTTGGGTTCAACCTAGGTGCTCATGCCCTAATGGCTTAAATAAGTAAGGGCTTGTGCCTAATA
TACGGTGTTATGTGTCAAAAGTTGTGTTTCTACCAACAGCTTAAGCTTTTGGCATGGTGGTCATAT
CTCTTGGTATTAGAGTTGGTCTGGGTTCAAATCTCAGGTTTGAAATGTTTCCAAATCATCTCAAGG
TTTGAGGACTAACCTTTTCTAAGAGGGAGGAAATGATATGCCCAAAGAGTCATGAGGTATATATT
GAGTTTGTGGGTTGGATTCAAGCCGGTGGCCTGTGTCCAATGACTAAAACAGGAAAGTGCTTGTG
CTTGATGCAGTTTTATGAGTCAAGACTCAAGAGTTGCACATCTGCCAACATCTTAAGCTTTTGGCA
TGTTGATAAAAGCACTCTTGGTCATATCATACAATCCTTTTTTAAAATATCTTGAAATCTATTTTTA
TCATATTCTTCTTGGGATCAAGCATAAAGAATTTGAAGCTGTTCAATGTGGAAGAATCTGTGTTTT
GTAGTTCTGGTCTTGATTGACTTGAGTGCATGGGTGACTTCTTATTCATAGATTTCTTCTGGTTTTTT
ATTTCTCAGGGTAACAAAAATATTGTTGTAAATAATGAAGGTGATGAAGTCCCAACAGATTCCCA
AAAGGTAACTTCTCCATCGTCTAGCCTTCCCACACATCATAGCTGTGTTTCTTCACTGAGTACAGA
TTCCGTGAGCCCAACAACCAGTTTGATGTCTTTACATGAAGATGCTGATTCTGGTAATTCTGCTTA
AATTTATTTCATGAGGGTTCAAAAATTTGTGGCCTGTGAAGTGAATTAATATGCCAAGAGCCTGTT
TGGATAAATTTCTCCGTAAACACTTTTAGGAGAAGGAAAAATGAAACAAGTTTTTCCATAAGCTT
AAATTAACTTATGTATAAATTATTTGGTAGAAGCTTTCTCATTTTAAGCTTATGGAGAAACAAACT
TATTTCATTTTTCTTTTTATTTGCTTCTACTATAAGTGTTTATGGAGAAGTTTATTGGAACATGCCCC
AAGTCTTTAACTATCTTGGGGTACTGTTACAACAAATATTTGTGTAATAGTTTGGCCCATTTGATC
ACCCTTAAATGATTTAATCATAATTCTTTCCAGAGGATATCCACCATGCAAGTTCTGGGTTGCACC
CATTACATGAGTCACAACATAGTGGGAATAGTCCATTGACGGAAAAGATTGGTGCTGGTTCGAA
CAGTTCATATCTTATGCATCCCTTTTCAGGTTACCTTCCCTCCTTTTTCTTCATTTTTGTTGTCTCACA
TGTATATTATAACTTCTATTAGGCAACCTCTGACATGTGTTTCCCCCATATATATATCTTAAAATAT
TTTTAGGTGATAATGAACAGTCATCAATTTCTGGAACGGATTATGTCCCTGTTGTTCATGGGGATA
AATTTAGAGGAAATGATACTGCTTATACTGATGGTCAGAAACCGCATGGCATGGCACCATGGGG
CACTGTCTTGCAAAGCACTGCCAAATTGCACAATGATCCTTCTCTTGCATCATTTCCTTCCATCCT
ACCTAGTTCAATGGGAGACGTCCTTGAACAAGAACATACCATTTTTGGTGACCTTTTAATGAGCA
AAAGTGGTCTCACTGAGGAAGCAGAGAGTTCTCAGTCTCTCCAATCAAATTGGCAGGTACATACT
GTTACTGCATAGTATATCTATCAGTAGTATTATCTCACATCTATGGCTATTATTTTTGGGATGGAA
AAGACATAATTTGACAGTTTTATGGGACTAAAGCCTGTGCCAGAGCCACTTGACCTGCAATTTTTT
ATTAATCTTTTTAGTTAAACTTAATGATGAATATATTAACTATCTAGTAATGGGCTGCATTTGTTAT
ATAAAAAGGTTTTGAATTGTTTCTGAAACTCTAGAAATGCTTTTGTTGGGAAAAACCCAAGTCCT
GCATTGACTAGAGATAGTGCCAAGATAGGGTACATAAGTGGGGAATAACCCTCACCTTATAAGC
TGTTTTGTAGGGCTGAGTTAGGATCCAAACCCAAATTCTAAGAGCCATAATTTCAGATTACAGTTT
TTTTAGTAACTTGAACTTTCTCCAGATGTATCCATATCCAATCTTTTTGTTGAGATAAATATTTTTT

AGCTTGAACTCTTCGTAAATAACTTGTTGGGTAACCATGACTGGGTGTTGGTTTTCATAATATAT
CTTAATCTGTTGTCTTTAAAAAAAAATGACTGGGTGTTGCCTTGGTGTCAATTGTATCTTTTGATTG
CTTAATTTGAAACTGATAGTTTTAACTTTGGTTTCTCTTGTATGTAATTTCAAACTACTACTGCTTA
TGAGATTTGGCTGAAAACTATAACTATATTCATTTTGTTATTTAATTTGATGCAGATTCCTTTTGAA
GACAATTCAGGAGGCATGCCAATGTTAACCCAAACCCAATCATTTGGTCTGCAATTTAGATCTGA
TTATGGCACCGGTTTACTAGGGAACGAAACTCGTAATGCAAGTTCTGAAATTGCTCCGATCCTGT
ACAGTTTTCATGGTGAACCAAAAGAGCAGCCTATGCAGCAAAACTATCCACAAGAGCTTGAAGA
TGGACAATCTCAACATGCACTAAAATCCAATTCTGCAAATAAAGTTCCTGATGAAGAAACTATC
AACTATGGCTTAACAGTGAAGAGCACATTATTGGACAGAGACGAGAGCCTGAAGAAGGTTGATA
GCTTCTCTCGGTGGATTACTAAGGAACTTGGAGAGGTGGCTGATTTGAATATGCAGTCCTCACCT
GGTATTTCATGGAGTACAGATGAATGCCAGCATGTGATAGATGATACCTCATTGAGCCCATCTCT
CTCTCAAGACCAACTGTTTAGTATAAATGATTTTTCACCTAAATGGGCTTATGCTGAGTCTGAAAT
TGAGGTACTGATCTTGTGGAAAATCTATTTTATTATTATTATTATTATTATTATGAAGAAGAATCA
GATGTAGTTTGATTGACATGCTATCCTGTTCAAAACTATACAGGTGTTGATTATTGGATCATTTTTG
AAGAGTCAACCAGAGGTGACAACCTGTAACTGGTCCTGTATGTTTGGGGAAGTTGAGGTTCCTGC
TGAGGTCTTAGCTGATGGAATTTTGTGCTGTCAAGCTCCTTGTCATAAGGTTGGACGGGTCCCTTT
CTATGTAACATGTTCCAATAGGTTAGCTTGTAGTGAAGTGCGGGAATTCGATTTCAGAGAGGGCT
TTGCTAGAAATGTAGATTTTGCAGATTTTTATATCAGTTCAACTGAAATGCTGCGTCATTTGAGAC
TGGAGGATTTTCTTTCTTTAAAGCCAGTGGACCCTTCAAATCATTCTTTTGAGGGTGATATGGAGA
AAAGAAACTTAATTTTTAAGCTCATTTCACTGAGAGAGGAGGAGGACTATTCAATTAAGGATGA
AGTTACTAGAGAATTGGATATATCCCAACACATGGTGAAGGAGCATCTGTTTCACAGGCAGTTTA
AAGAGAAGCTGTACTCATGGCTTCTTCACAAAGTAACTGAGAATGGTAAAGGGCCAAATGTATT
AGATGAGGATGGGCAAGGTGTTCTACATTTAGCAGCTTTTCTTGGTTATGATTGGGCCATAAATCC
AATTATATCTGCTGGAGTTAATATCAACTTCCGAGATGTGAATGGATGGACAGCTCTTCATTGGG
CTGCATCCTGTGGCAGGTAAGGTTCCTTGAGATTTTTTTTTCCTCTTTATTGAGTACAGTTGGTTGA
ACATAAGTTACGTCTTCTAATCTGGTTGAACCTTCTTTTACCTCCCTGATATTCTTAATTTTTTTTTC
CACTTTTATCAAGTGAATTTGTTTACAAAATTGGTTTGTCCTAGCATATGAAGCAAATTTAAGTTTT
CTTTAAAGGGTGGTAAGAAATCTCCAGTGTTTTGCATGAATGGACTTGTTCCACACTTCCACCCCC
TCCTCTCCTTGGTGCTAAACGTTAATACAGTTTTTATTATGTGTTTAAAATAGTAAATTAGCTAACT
TAGAGTTATCCCTAGAGCAATTGCTTTGCTTAGTCAAAGGCAAATCACAGTCTGCTTGCAGCATT
GACTTTCTCAAATATATTATGTTCAGCATAACATAATTTTTTCACTTATGTTATTCTTTCCTCCTTTT
CTCAGAGAGCGGACAGTTGCTGTCCTTGTCTCCATGGGTGCAGATTGTGGAGCACTGACAGATCC
ATCTCCCGCATTTCCATCTGGTAGAACAGCTGCAGATCTTGCCTCTAGCTATGGGCACAAAGGAA
TTTCTGGTTTTCTTGCTGAGTCTTCATTAACTCACCACCTCGAAACCCTCACAATGGATGATCAAA
AAGGTGGTCAGCAAGAAATTTCAGGAATGAAGGTGGTGCAGACAGTTTCAGAACGATCTGCAAC
ACCTGTGCACTATTGTGATATCCCAGATGCCATCTGCCTCAAGGATTCACTTACTGCTGTGCGTAA
TGCAACACAAGCTGCTGATCGTATACATCAAGTATACAGAATGCAGTCATTTCAGAGGAAGCAG
TTAACTCAGTATGAAGGTGATGATGAGCTTGGATTGTCAGATCAGCAAGCTCTTTCTCTGCTAGCT
TCCAGGGCATGCAAATCTGGACAAGGGGATGGTTTAGCCAATGCTGCTGCAGTACAGATACAGA
AAAAGTTTCGTGGTTGGAAGAAGAGAAAAGAATTCTTGATGATTCGTCAAAGAGTTGTTAAAAT
CCAGGTACGTTGATCTGTTCTTTTTACTTGATTTTTCATTTTCAATATTCTGGACTTTCTTTTTAAGT
AAACCTGTATATTGTTAGATGGTCTTAATTTATCATCCAATCATCTTATGAATTATTCTGCTATTTT
GGAAATTAAAGGTGGAAAATTGATTGCATTGGAGATTTTGATTGATAAAAACTCTGGATGTGGAT
AAGAATGGAAAAATGCTTCTTAAACTTTTTCATCTCTTTGCTCCTTCTATAACTACTGTATACATGT
GGATCACACATTGGGATTGTCAAAAACTGTAGAAAACCATCCAGAATGTGAGGAAAGAATGCA
GTAAAGTGTTCTTTATATTGGGTTCTTCCTTCCATCATTCCATGCATTTACTTGGTGATTTGGTGTTG
TGTCAACATTCATCAAATCATGAACTTGAGCACTTAGTGGGCCGTAAAAGCAGTCGTATGTCATG
GCAGATCCAATGACAACTAAGACCACTTGAAATAGGGAGCATTTAATTCCCAAGAAAGTACACC

AAAGGATATATAGCACTATTGTCTAAAAGTCTTTCCTTACCTCCTGCCACGACCTGTAAAGTAGA
AAATTCAATTTAAGGACACAACTTGTATCCACACAAAAAAAGATTTAGGAAGCTACCGGAAAAG
ATATTCAGAGACTTCCCAAAGCTTTAAGTTCAATTATCTCTGGCCTGAGTCGTCTTTTAAGACTTG
GGTGTGAAGTTGATGTTTGCCAAGCAACTTTACCCTTCCCCTGCTTTGCTACTTGCACCTCCAATG
TAACTTCCATCCCACCCCCTTTTCCCAAAAGAAGATATGACTACTTAAGATATTTTCCTGTTTTTC
CCCTATCTTTCATTTTTTTGTGTCCCTATGCCCTCGACACATGCTTCCACTGACTGTTGATATCTGTG
GTGTCAAGGCCCATGTTAGAGGTCACCAGATAAGGAAACAGTATAAACCAATCATATGGTCTGT
GGGAATCCTGGAGAAGGTCATATTACGTTGGAGGCGCAAGGGTAGCGGTTTACGTGGATTTCGA
CCAAATGCCATTAATAAAGTCCCCAATCAACAAAATGATTCTCTGAAAGAGGATGATTATGATTA
TCTCAAGGAGGGACGGAAACAAAAGGAAGAAAAGATCCAAAAGGCCCTATCAAGGGTGAAGT
CCATGGTTCAGTATCCAGAGGCACGTGCTCAATACCGGCGTCTGCTAAATGTTGTTGAGGACTTC
CGTCAAACCAAGGTATTTGTTAACATCTGGCTGGTGTCTAAACTCTTTAATATTTGGCTTAATGAT
ATGCTCATAGCTGTTTGATAACTGGCAGGCATCTAACAAGGGTTTGATCAATTCTGAAGAGACAG
TTGATGGTGTGGAGGATTTGATTGATATTGATATGCTTTTAGACGATGATAATTTCATTCCTATAG
CATTTGATTGATCCTGCAATGGCTACTCCCATCTTCCCCACTGTAAATACATGGCCAGTTTGGCTT
TATTTGGCATTGGGGAGTCCTAACATCGTCTTGATGTATATATTTCTAATCAAAATTACCCGGATC
CAAAGGCTGCATATAAATAGATTATGATAGCTAGAATGCACTCTGGTTTGACAGTTAAGATATTG
TAGTCTAATGAAACATGGTTTATAGAAGATTTTTTTTTTTAATAGGCAGATGGTTGGGGGTTATA
TCATAATGATGTAACTTGCTAAAATATTAATCTGCAATGTGTACAAAAGGCGACTGTGGCGAGAG
AAACTATATACTAAGTGCTAATTATGATTTATGATATCAAGCTTGTTTCATCGTTT

>Glyma08G072100 (CAMTA4)

GGTGGTAGCTGTCCCGCCTGTCCGGGTTATTCACTTCGTCACGTCACTGTGTGAAAAGAAGCTGA
CTGGAAAGAGCGAAAGAAAGTAAGAACGAACGAGCGAAAGCAAAAAAGTGTGTGTTGATTGA
GATACTGCTAACTCCTTCAACGTTTACGCGTGGATCTCTCTCTCTCTCTCTCTGATTCTTCGGTCTTC
GCCATGTCGGAGCGTTCGTCTTTCGGATTGGGACCTCGATTAGGTCACATTTTTTACTGATTCATTC
GTTTGATTTTCTGCTTCTTTTTGTCTGAATAGAGTTTAATATTTGATTGAGAGTTTTAGAATGCCTG
ATCTTGCTGTTTGATTCTTTGTCGGAACGATTCGCCTCGCATAACCTGACTTCGTGAATTTTTGATT
ATTGGTTGTAGGATTATGGAAACGAGTTAATAGATAGGGGGTTTATTTTTTTTGTGCGTGAAAAAT
CCGTTTAGGTTTTTTTATTGGAAATGACTTGGTTAGTTCAATTAGGGTTTTTGGGTGAAAATGAAG
AAGCGTCTTTGATGGCATCTATGTGACTTTGTCTATTAGGTGTGAATGGCTTGGACTTCGGATTAG
TATTGGTTTGCATTGGATAGAGTATATGCTTCAATGATGAAGAATTATAGAAGCTATTATTGTTGT
AAATATGTATGGTACTATGTGCTAATTGGCTGAAAAGCCAATATGTGTCTGCTACAAATTGGGTTT
GTATCACAGTAGCAGTTTGCATCTCTATTCCGAATGCTATAACAAACTATATTCTGCTCTGCTTTCT
CAATAGTAACAAATTTATGTGGCTTAAAGAAATTCTTCTATGGCTACGTCCTCACTGGATTTTCAC
TATGCGATGAGAGATATGCCTGGCAAGAGATTAGACACCTTTCCCTTTGACAATTTCAGAGAAGA
ATAAGCTGCGTAATTGCTTTTTACCCATTTTCTCTTGGAAAAGTTTTGCTGCAAGATAATCTCTTTG
CTTGCTTTAGCTTTACTGTCACAGGGGTGAAAATGAGCTGGACTGGGCCGGGCTGGGCCTTACCT
AAGCTCGGCCTGGCCTGTTATATATTTCTATAGTCCAAGTCTCGGCTTGTTGCCCATCACGAGCCT
ATTTTTTAGGCCAGGGCCTGGCCTTTATGTAAGCCTGTTTGTCCTGTTGGTCTATTTAAAGGCTTGT
TTCATATTAAGATGTGTAAAAATAATTTTTTCCTTTAACAAATCCAAAGGTTTTCATTAAAAATAA
TAAGGTAATTTTAAACACAGGATGTGGCCAAAACTTACCTAAAAACATTACATGTGATACGCTAC
CAAAGCATTTTAAGCCCCACCAAAAGAAAATAGTCCTTATTCCTGCTATACCTGTCTATGCTTATA
GAAATTAACTTATTTTAAATATTAAAATGATTCAAATTGTCAAACCACTAAAATAATTTTATAATT
TCATTAAAATAATAATAATATTATACATCATATTAATAATAATATTTTTATAAAAAAAAATTTGTTG
CTAAATAGGCTGGCCTTAACTGGGCTGGGTCATTGGCCCTCGACGGGTGTCCTGGCCTACTTCCA
CCCCTACGTGAGCAACATACTCTGCTTCTGTGATAGGAAGAGACAACAATATTTGTAGTTGAGAT

GCTCAACTTAAAGCCCTTCCACCCAAGGTGTAAGCATAGTCAATAGTGTTTCTTCTGTTATCAAAA
GCTCCACATAAGTATACTTTAATGCAACCTTACTGAGTGTCCCTTTTTAAAACAACGTGACTATGT
ATTAGCTGTTACCTCTCAATTGCCAAAGGATCCATTTAAGTCTCTTGATGTTGCTTCCCTAATTAA
ATATGAACCTGCTCATAACTCCACATGCTATAGTGACTCAGATAGAACCAGTTGCATGCTTTAGA
CTACCAACTGCTAAAGCCTCGAAACTCGAGACATGAAATTATTTTCTTCTTATATACTGGGTAATT
GATCTGTAGCCTGAAGTGACTTTATTCTCTTCTTATATATTGGGTAATTGATCTGTAGCCTGAAGTG
ACTTGCCTCTTAAGTGCTAACAAGTTTTGAATCTTACGTTGTTAATTTTCTCAACATCTTTTCAATA
TTACCAGCTTGTAAAAGCTTCAAACTGAAACTACTTGTCACTTTCTTCCAAACAGTTCTCATCCCA
TGAATTTGATTTGTAGCTGCCTATTTTTTAATAGCAAAATCTTTTGGTAGTTGTTGTTTTAGCGTGTT
GACACTGTGGTTGCTAGATCCTCTATTCATCAATGCATCAAATGAATAGGATAATATGGCATTAA
AATTTTAAACGAGCAGCAGCAGTAATCTGTATGACATCTCCTAAAGTGAATTAGTCATAGTTACA
CATGAATAAATCAAATTTCTTATGCATGTGAGATTGTAAGAATTAAAGTTATTATTGATATGATTA
GTGTAGCTGTAGTTTGATTCTTAAGGTTTGCCTCAGTCATTGCATTGTGCATGTGATGATATAGCA
GTCCAATATTTAAGTGAATGCCATGTTCTATTATTTATCAATATGCAGATCTTCAGCAATTACAAC
TTGAAGCCCAGCATAGGTGGTTGCGCCCTGCAGAAATATGTGAAATTCTTCGTAATTATCGGATG
TTTCAAATCACATCAGAACCACCTAACAGGCCACCAAGTATGGTTTCTGCATATGTTACCGATCA
ATTTATTAATGCCACCTATGCCAGTCTTGTCCATATATGGGTAGATTGAATTGCTCAAAAGGCTTA
AAATTACTAATCCTCCATGAATTGTTTGTTGCAACTGGGTATTTCTGAGGTTGTCTGGCAACCTTA
TAGATGCCATTCATGGAGGTTGGACATATCTGTTTTTCTCTTGCAGCAAAAATATGAAATACTTAA
ATATTTTGTCTTTCTTAAGTTGTTTGACAACCTTATACATGCCAACCTTTTAGATCCCATAGGATCT
TTGCTTCACTGCAAAGGGCACCTGATACATTGTAATCATGCCTGTATTTTCTAGGGTACTATTGAT
AGTTTTGTTGCTCATGCTAAAATCTTGCTTCATTGTAACTTTAATTCATCTTTTCATGTATTCGATGA
TTGCAGGTGGTTCCCTTTTTCTATTTGATCGGAAGGTTTTAAGGTACTTTAGAAAAGATGGACATA
ATTGGAGAAAGAAAAAAGATGGAAAGACTGTGAAGGAAGCACATGAAAAGTTGAAGGCAAGC
ATGAAATTTCTTTTGTTGAGTTTGTGATTGATAAAATCTTTATAAGATCATATATATATATGTTATA
ACTCCTATTATTTCAGTTTGTTTTTCTCTAATTGGAAAACAACTCTTTATACCCCAAAAAATTACTT
GCTGAGATTGTAATTTCCTTTGAACAATTAGGTGGGAAGTGTTGATGTGTTACACTGCTACTATGC
CCATGGAGAAGAAAATGAAAATTTTCAGAGGCGAAGCTATTGGATGCTTGAACTGTGAGTGCGA
TGAACTGGTTTACATTTCATTTTTGTTTATTAATTGTCGTTGAACTTGAAGTACTATATTATCCATG
GCAGTTGCTGTCTGAATTTCTTACTAAGTACTGCATTACTACTTAGTAGCATAAGTATATTTGTAA
TTGTATTTGGAGTCAGTTAATAGTTAATTTATAACAAGTATATATTACTGGACTTGACTGTTTTTGT
GTTTCTCAGAAAATTTTTTAGTTCCCAATATTCTACTGAAATAATTTATTTTTAATAAAATTAATTA
GAATGTGAATTTGTTTTGCTGCAGGGATATGATGCATATAGTATTTGTCCACTACTTGGATGTTAA
GGTATTTTGTTTGTTTTCTTTATTTCGCACTTTCTTATTAAGGTATTTTGCTTCCTTACTAGAGCAAG
AAATATATCATTGCACAGCCTTGTTGTTGGTGCAATTTTAACCCGAGTGTGTAACTGTGTATGACA
TCTAGTAGCTCAAGCCTAGTCTTTGGAGTTTTTTTATCCTTAAGACTTCCCTCTAAACTTGATGGAG
GAAATAGAGTACTAGTGTACTACTGTACAAGTACTATTTTCCTTCCCTTAAACATTGAGTATAATG
TGAACAGATAATCTATCTTCCATCTGATTTCCTTCTCAATTTCTTACCATCATGGATTGGAGAATGT
GCTTCATAATTGTAAGCCTGGATAGCAATGTTTAAGAACATCCACCAAATGCATACTAACATGCA
ATATTTGTTGCTGGTCTGCTGCTAAATTTATGTTTTTCACTTTTTACCAAGAGAAGACATCCTTGAT
TTTTAATCTTCAGAAACCCCAATTGATGATATTGAGTAAGTTGCATAATCATTTTGAAAAGATCCT
GAACTCTGTTTCTATAATATTCTTGAAATCAGGCAGAAAACTTTTGAATCCAATATTGATTCACTT
GTTTGATCATTGATGACCTATTGACTTAATATTAATAGGTGTTTTCTGGGTTCTTCGTCTTTCAGGTT
AACAAAACCAATATTGGTGGAAAAACATATTCTGATGAGGTTACATCAGATTCCCAGAAGAGCA
GTTCTTTGTCATCTGGCTTTCCCAGAAATTATGGTAGTATGCCTTCAGGAAGTACAGATTCCATGA
GCCCAACGAGCACTTTGACATCTTTATGTGAAGATGCTGATTCAGGTAACTCTGATATATTTAATG
GTTGTTACACTTAATTCTTTTGATCTGAATTTAACGTGCCAATGCTTTAAATATCATGGTTTACTAT
TATGCCATACTCAAGTGTTTGGTCCATTTGGTCTAACTGGTTTAATCAAACTTCTTCCTAGAGGAT

ATTCATCAAGCAAGTTCAGGATTACACTCCTATCGTGAATCACAAAATTTGGGGAATGATCGTCC
GATGGATAAGATTCATGCTCGTTCAAATAGTTCCTATCTTATGCATCCATTTTCAGGTTATCTCATC
TTTTTATTCTTATTGGTCTTAGTCTCTAAGATGATTATGTTGTTTAAATTCTATAGTTTTATGCATAG
AGGGAGGAAAAGGAGATCAGGAGACTGAATACATTGATACTGATATAAACTTTATTTGATATGA
TGTTTTACCTGTGTTCAGAAGTGATACAAAACACAAAATACTAATCCCTATTTAAATAGGAAAAC
AAATCACAAGAAAATAAGGAAATATGGAAACTAGTCTTTAGATGCTCTAAATTAAGATACAATC
AAGATATTGGATTTTTTTTTTTTGTATATTCTCACATATTTAATATCTTAAATAACATCTGATTAATA
AGCTTTTCCTGGGATTTGACATTTGAATTTTGTTGTATGTTTTGAAACGTTTTCAGATAATCATGGG
CAGTTACCAGTTTCTGGAGCAGAATATATCCCACATGTTCAGGGGAATAAGTCTAGAGCTAGTGA
TACTACTTACATTGAGGGTCAGAGAGCACATGGCATTGCATCCTGGGACAATGCCATGGAACAG
TCTGCAGGAAAGCATGCTGATCCTTCTCTTGTATCATCTACGTCCATTCCATCAAGTGCAATGGGA
AACATACTTGACAAAAACCATACTGTTCCAGGCAACCTTTTAGGGCATAAAATTGCCCTCACTGA
AGTGGAAAGGGGCGCTCAGCCTGTCCAATCAAACTGGCAGGTACTTACTGTTATTTCTCGCTATA
TCTTCAGTAGTGTACTCTTGCTTGCATGCATATATCTGATGTCTCAGCATAAATATGAGTATTCTTG
AGGTTCTCTTAGGAACAGTTGTGCTATCTTGTTGGATGGGACAATTTATGGAATTTTCTGTCCCAG
AAACCATAGTTTCTGCAATTGTGGTGGAGCGGCACCCTGCTCCTCCTCCAAGGGCAGTCATGGTG
TGGTGTGGTGCATTTCACGGCAAATACTGGGCACTTTTCAGAGGAGGCATCGAATCTTTATGCTTC
TTAGCAATCTATTTATAATTTTGTTCTAGAGCACCTGCTTCTGTTTTGCACTTCTGCAACACTTCCT
GTCATGAACCTCTGTTTTGCAACTTCCAGCAGTGCAAAAAATAGAGTGACCATCACGCTATCCAT
TATCCGGCTATAGCATTTTTTCCCGGGGAGGGGGGGGGGGGGGGTTCTCTGCGCTATTACCCGCT
ACTGACTACTATGCTAGTTGATTAAGTTTTAAGTGCATTCTGTGTTGTTGCTAATGGACTTTATGCT
TGATGAGGTGTGTATGGTTCACCTATTCTTTTATCTTTGAAAAGAATTGCTTGAGGAGGGGAAGCT
AGGGAAGGTGAGAATTTGTGGGAGCTGTTGCTTCATATCAAAAGAATATTAAGAGATTCTAGAA
GATGGATTTGTGGAAAAAGTGGCCTGGGAATATACAGTTTCTTTCCAGGGAGTTAGTCATTTAGC
CATGTATTTTAAATTTTTATTAAGTTGCAATACAATAGAATGATGAATTAGGATAGAGCTTATTCG
TAACTTCTTGTTACTGGTGTACATGTGTAGAACTGAATGTTTTGAATTGTTTCTGAACTTGTAGAA
ATGCTAAAGAACATAGTAGTGCTTATCTTAAATTTTTGCTAGACTTTCTTTTTTACTTTATGTTTGTC
CTAATGTATCCTTATTTAATCTGCTGAGATGAGAATTTAGTAGTTTGATGGAGGGAGTGGCCTTGC
ATTAGTTTGGTGTCAATTTTATATTTGATCTGTAACCCTGATGTTTTAACTTGTAGTGGTATCTCAT
AAAAAAATTTAATCTGCACTACTTTGTGAGATTTAGCAATGACGTATAACTATTTTCATTTTGTTA
TTTTAATTGTTGATTCAGATTCCTTTTGAAGACAATACTGGAGAATTGCCTAATTGGGGCTTTACC
CAATCATTGGGTTTGGAATTTGGATCTGATTATGGTACTAGTTTACTGGGGGATGTAACTAATAAT
GCAGGCCCAGAAATTGATCCTGAGCTGTTCACTTTTAATGGTGAGTTAAAAGAGCAGTATACACA
TGGGCAATCTCAACCTGCACTGAAATCCAATTCTGCGTATGAAGTTCCTGGTGAAGCAAGTATCA
ATTATGCCTTGACCATGAGGCGTGGATTATTGGATGGAGAGGAGAGCCTCAAGAAGGTTGATAG
TTTCTCTAGGTGGATGACCAAAGAACTTGCAGGTGTGGATGATCTGCATATGCAGTCTTCCCCTG
GTATTTCATGGAGCACAGATGAATGTGGGGATGTGATAGATGATACATCATTGCACCTGTCCCTG
TCTCAAGACCAGCTGTTTAGCATAAACGATTTTTCACCTAAATGGGCTTATGCTGAATCAGAAAT
TGAGGTAATTTGTCTGAGATTCACACAATTATAATATATATTTTAGCAACCATTTATGGTTGTGCA
GTTTGTTTTCCTTTTCTTGTATGTCGTGAGCATAAGTAATTAAATATGTATGGCAGTTGGATTCTAC
TTTAGATTTATATCTTTTTGCCTTGGATATTGACAGCCTTTTTTCAGGTCAGATGCATATAGTTTTGT
TCTTTATTTATTTGAATATTCCCAACATGTATCTCTTCTCGTTGTTACTGAATATTTTTTGTATTTCAT
GGTGTATCCAGTATCCTGTTGATATTCTTTATTTGCTTTGGAGTTTTATACATATTAATGGTTCACTC
ATTTTTCTATGGGTTTCTACCAAAATATGAAATGAAGAGAAGTCAGCTGTAATTTGACTTATATCT
TATCCTATTCAAAACTATACAGGTGTTGATTGTTGGAACATTTTTAAAGAGTCAACCTGTGGTGGC
AAAATGTAACTGGTCCTGTATGTTTGGGGAAGTGGAGGTTCCCGCTGAGGTTTTAGCTGATGGAA
TTTTGTGCTGTCAAGCTCCACCTCATAAGATTGGACGGGTTCCCTTCTATGTAACATGTTCCAATA
GGTTTGCTTGTAGTGAAGTTCGGGAATTTGAGTACAGAGAGGGCTTCGATAGAAATATAAATTTT

CCAGATTTTTTTAACAATTCAAGTGAAATGGAGCTTCATTTGCGACTGGTGGGGTTACTTTCGTTA
AACTCAATGCACACTTTAAATCAAGTGTTTGAGGGTGATATGGATAAAAGAAACTTAATATTCAA
GCTTATTTCACTGAAAGAGGAAGAGGAATATTCAAGTAAGGAAGAGACAACTGCAGAGATGGA
TATATCACAACAGAAGTTGAAGGAGCATATGTTTCACAAGCAGGTTAAGGAGAAGTTGTACTCA
TGGCTTCTTCACAAAGTTACTGAAACTGGTAAAGGGCCTCTTGTATTAGATGAAGAGGGGCAAGG
CGTGTTACACTTAATTGCTGCTCTTGGTTATGATTGGGCTATAAATCCTATCATAACTGCTGGAGT
CAATATCAACTTCCGTGACGTGAATGGATGGACAGCTCTACACTGGGCTGCATTCTGTGGCCGGT
AAAGACCTAATTTCTTTTAATTGTATTGAGTTGATTTTGTGTATGTTGGAACTAATTATCTGGGTGA
AACCTCTTTTGCTACCCTGATTATTTCCTTTTTCCAGAGTTTTGGAATTTTGGATTTGATTAAATATG
AAACCATTAGGTGGTATGAGAATTCTAGCCCCTTTCCTTTAGGAAATGTCTTCTCTTTTTTTTTAAT
TGTTAATATTTATTTTATTTTGATGCCAAGGTTGGTGGATTTTTTATACATGTGAATTTGTTCAAAA
ATAATTTTAAAGCAATTGCTTTGGTTAGTTACAGACAAATTTAAAGCTATATTCTGTGATGCTTTC
AGCATTAGAGTATTCCTTTTCAAAATGTCCTCAGATATATTATCTTCATTCAGCATAACATGAATT
GGTTCCTAATATTATTATTTGTCCTCTGCTCAGAGAGCGAACAGTTGCTGTCCTTGTCTCCATGGAT
GCAGCCGCTGGTGCATTGACAGATCCATGTCCCGAATTTCCTTTGGGTAGAACACCTGCAGATCT
TGCTTCTAGCAAAGGCCACAAAGGAATTTCTGGTTTTCTTGCTGAGTCATTATTAACTAGCCACCT
CGAATCACTCACAATGGATGAAAATAAAGATGGTAGGAAAGAAACTTCAGGAATGAAAGTAGT
GCAGACAGTTTCAGAGCGAACTGCGACACCTGTGCTTAATGGTGATATACCTGATGATATCTGCC
TTAAGGATTCTCTTAATGCTGTCCGTAATGCTACACAAGCTGCTGATCGTATATATCAAGTATTCA
GAATGCAGTCATTTCAGAGGAAGCAGTTAGCTCTGTATGAAGATGATGAATTTGGATTGTCAGAT
CAACAAGCTCTTTCTCTCCTAGCTTCTAAGGCGTGCAGATCTGGTCAAGGAGAGGGTTTAGCCAA
TGCTGCTGCAATACAAATACAGAAAAAGTTTCGTGGTTGGACGAAGAGAAAAGAATTCTTGATT
ATTCGCCAAAGAATTGTTAAAATCCAGGTTCATTGTTCTGCTCTCTTTATTTTCCTGTCTGTATTTTT
AACAAGGCAAATTATAAATTTAGTTGCTACATTTTTGTTTGTCCCTACATGAAAAAAAACTACATT
TTGGTCCCTACGTGAAAAATTGCTTACATATTTGTCCTTAACTGCCTAATGAAGTCCTAAAGGTGT
TAGTTTTTTCATATAGGGACCAAAACTAAAAATGGATCTGTCCGGTGTAGGGGCCAAAATAAAGT
GTAAAATTTTCGTTTTATGACTAAAATGAAAAATGATGTTATTGTGTTAAGTAGAGAGGGGCAGT
ATCTAATACCGTACCTCGATTTACTATTTGTACCTTGCATATTATTATTTTTATTTTTATTCTTATTTA
TATTATTATATAAAGATAGTCTATAATCACTAGAATTTCTATATATTTATACTTAGTATATTGTAAT
GTTGCCAAACAGCAGGGATCAAATCATAAATGTTTGTTCATGTAGGAACTAAAATGTAGAATTTA
TGGAAGGTGTAGAAACTAATGTTTTCTCATTGAACATCCTATCAACTATTCTGCTATTTAGTTTCA
CCTGATAACATTGCATTGGAGGGTTCAATTGATGAAAACTTCGGGAGGAATGCTAAAATCCTTTT
TTATCAAATACTTCCCCATCTCTGTTTGCCCTTTATATATATATAACTTGCACGCACATAGAGATC
ACGTTGGGTGTCAATATTGATAATGATTTCCAGTTGTAAAGAAATCAGAGGTTAATAGAAATCAT
GTTTTGCGGCCGTTCATTTCTTGTTGATGTCTTTTCCATCTCTTCTTTCTGAAGTATTTGCAGTCTGG
TTGCCGTAGAAGGCTGGAGATTGAGCTCCCTTTTTCTGTGTTTAATATTTTATATGTGCTCTTGGTA
CATTTTATGCATCCTTCTACTGACTTGAGTCCATGGTGTTCAAGGCCCATGTAAGAGGTCACCAGG
TCAGGAAGCAGTATAAACCAATCATTTGGTCTGTGGGAATCCTGGAAAAGGTTATATTACGCTGG
AGACGCAAGGGCAGTGGTTTACGTGGATTTCGACCAGCTTCCCAGAACAAGGTCCCCGAACAAC
CAAGCGAATCTCCGAAAGAGGATGACTATGATTACCTCAAGGAAGGAAGGAAACAAAGTGAAG
TGAAGTTCAAAAAGGCCCTTTCACGGGTGAAGTCCATGGTTCAGTATCCAGAGGCACGGGCTCA
ATATCGGCGGGTGCTAAATGTAGTTGAGGACTTCCGTCAAACCAAGGTATTTTCTAAGTTATGTCT
TGCCCTACGTATAAACTCATGATCAATACTTGCCTTAATGATATGTTCTTTTGATGTCTACCAGGG
AGGTAATTTGAATTTGATAAATTCGGAAGAGACAGTCGACGGTGTGGAGGATTTGATTGATATTG
ACATGCTTTTAGATGATGAAAATTTCTTACCTATAGCTTTCGATTGATTCTCTGCTGTTGCTCCACC
CACCTCCCCGGTAAATAGATGGCCAGTTGGTTTTATTTGGCATTGGAGAGATCCTAACATCGTGT
GCTGCTGTATATATTTATATTTATGTATAATGTACAATCAGAATTCCTTGGATCATAGGTCTTCCTG
TTTTCTAGCAAAGAACTAGTTAGATCCAAGGGTGGGTTATAATAGATTATGGAACCTAGAATGCG

TTCAGGTTTGGTAGTTCGGATATATAATATACATGGTTTAATTTTATATATGGTGATTATGTATATG
ACAGGATGATTTCATTTAGATTGAAGTATGTTTTTAATTCTTAAATTTGTATCATTTTTTAAAAATT
TAGTCTTATTATAATTTCTGTTTT

>Glyma05G117000 (GmCAMTA5)

CGCCTATTCGGGTTATTCCCTTGGTCACGTCACTGTGTGAAAAGAAGCTGACTGGAAAGAGCGAA
AAGCGAAGAAAGTAAGAACGAACGAAAGCAAAAAATTGTGTGCTGATTGAGATACTGATAACT
CCTTCAACGTTTCCGCGTGGATCTCTCTCTCTGATTCTTCAGTCTTCGCCATGGCGGAGCGTTCGTG
TTTCGGATTGGGACCTCGATTAGGTCACTTTTTTTTATTATTGATTCAGTCGCTGATTTTCTGCTTCT
TATTGTCGGAATAAAGTTTAATATTTGATTAAGAGTTTTAGAATGTCTGATCATGTAGTTTGATTTT
TGTTGGAACGACTTCGCGTAACCTAACTTCGTGAAATTTTTATTTTGAATGTTGGTTCTAGGATTAT
GAAAACGAGTTAATCGATTGGGGGTTTCATTTTTTTGTGTGAAAATTCGGTTTAGGTTTTTTTTTTT
TTTGCAAATGACTTGATTAGTTCAATTAGTTTTTTTTTTTTTTTGGTGAAAATGAAGAAGCGTCTTTG
ATGGCATTTACGTGACTTTCTGTTAGGTGTGAATGGCTTGAACTTCGGATTGGTATTGGTTTGCATT
GAATAGAATATTTGTTTCTCTGATGAAGAACTGTAGTTAGAAGCTATTATTGTAGTAAATATGTAT
GGTACCCTGTGCTAATTGGCTGAAAAACTAATATGTGTCTGCTGCAAAATGGGTTTGTAGCCCAG
TAGCCGTTTGCATCCGTATTCCGAATGCTAAAACAAACTATAGTCTGCTCTGCTTTCTCTATAGTA
ACAAATTTACGTGGTTCAAAGAAATTTTTCTATGGCTATGTCCTCATTGGATTTTCACTTTGTGATG
AGAGATATGCCCGGCAGGAGATTAGACACCTTTCCCTTTCAATTTACCTTTAACAATTTCAGAGA
AGAATAAGATGCTTAATTGCTTTTTACCCATTTCCTCTAGGAAAAGTTTTACTGCTAGATAATCTC
TCTGCTTGCCTTATCTTTACTGACAGCAACTTACTCTGCTTCTGTGATTGGAAGAGCCAGCAATAT
TTGTAGTTGAGATGCTCAACTTAAAGCCCTTCCAGCCAAGGTGTCAGCATAGCCAATAGTGTTTCT
TCTGTTATCAAAATCTCCACATAAGTATACTTTTTGCTGAGTGTCACTGTTAAAACAACTTGACTA
TGTTGTATTAGCTGTTACCTCTCAATTGCCAAAGGATCCATTTAAGTCTCTTGATGTTGCTTCCCTA
ATTAACTATGAACCTGCTCATAACTCCACATGCCCATGCTATAGTGGCTCAGATAGAATCAGTTG
CATGCTTTAGAGTCTTAGACTACCAACTGCTCAATCTTTGGGTAATTGAAATTATTCTTTTTTTCTA
TATTGGGTAATTGATCTGTAGCCTGAAGTGACATGCCACCGAAGTGCTAACAACTTTTGAATCTT
ACATTGTTAATTTTCTCAACACCTTTTCAATATTACCAGCTTGTAAAAGCTTCAAACTCAAACTAC
TTGTCACTTTCTTCCAAACAGTTATCATCCTTGAATTTTCTTTGTAGCTGCCAATTTTTTAATAGCA
AAATCTTTTAGTAGTTGTTGTTTTAGCATGCTGATACTGTGGTTGCTACATCCTCCATTTACAATTG
CATCATTGTAAATTAATAGGATAATATGGCAATCATCAATGTTTTAAACGACCAGCAGCAGCAGC
CGTAATCTGTATCACATATCCTAAAGTGAATTAGTTATAGTTACACATGAATAAATCAAATTTCTT
ATGCATTTGAGATTGTAAGAATTAAAGTTATTATTGATATGATTACTTGATTAGTGTATTGATATG
ATTACTTGATTAGTGTGGCTGTAGTTTGATTCTTAAAGTTTGCTTCAATAATTGTATTGTGCATGTG
ATAATATAGCAGTCTAATATTTTAGTGAATGCCATCTTCTATTATTTATCAATCTGCAGATCTTCAG
CAATTACAACTTGAAGCCCAGCATAGGTGGTTGCGCCCTGCAGAAATATGTGAAATTCTTCGCAA
TTATCAGATGTTTCAAATCACATCAGAACCACCTAACGGGCCACCAAGTATGGTTTCTGCATATG
CTACTGATCGATTTATTAATGCCACCTATGTCAGTCTTGTCCACATATGGATATGGGTAGATTGAA
TTGCTCATAATGCTTAAAATTGCTAATCCTCCTTGAATTGTTTCTTGCAATTTTCATCTCTGTATTTC
TAAGGTTGCCTGGCAACCTTATAGATGCCAATTATGGAGGTTGTACATATCTGTCTTTCTTTTGCA
GCAAAAGACTTAAATATTTTTGTCTTTCTTAAGTTGTCTGACAACCTTATACATGCCAACCTTTTA
GATCCCAAAAGATCTTTGTTTCACTGCAAAGGGCACCTGATACATTGTAATCATGTCTGTATTTTC
TAGGGTACTATTAATAGTTTTGTTGTTCATGCTAAAATCTGGCTTCATTGTTAACTTTAATTCATCT
TTTCATGCATTCAATGCTTGCAGGTGGTTCCCTTTTTCTATTTGATCGGAAGGTTTTAAGGTACTTT
AGAAAAGATGGACATAATTGGAGAAAGAAAAAAGATGGAAAGACTGTGAAGGAAGCTCATGA
AAAGTTGAAGGCAAGCATGGAATTTTCTTATGTTGAGTTTGTGATTGATGAAATCTTTATAAGAGC
ATACATATATATATATATTATAACTTATTATTTCAGTTTTATTTTTCTCTAATTGGAAAACAAGTCT

TTATACCCTAAAAAATTACTTGCTGAGGTTGTAACATCCTTTGAGCAATTAGGTGGGAAGTGTTG
ACGTGTTACACTGCTACTATGCCCATGGAGAAGAAAATGAAAATTTTCAGAGGCGAAGCTATTG
GATGCTTGAACCGTGAGTGTGATGAACTAGTTTACATTTCATTTTTGTTTAGTAATTGTCTTTAAAC
TTGAAATACTACTACTTTATTTTCCATGACAATTGCTGTCTATATTTCTTACTAAGTACTGGCATTA
CTACTTAGTATAGCATAAGTGTATTTATACTTGCATTTGGAGTCAGTTAATAGTTAATTATAACAA
GTATATAACTATATATTACTGAACTGGACTGTTTTTGTGTTTCTCAGCAAATTTTTAAGTTCCCAAT
ATTCTACTGAAATAATTTATTTTTATTAAAATTAATTAGAATAAGAATTTGTTTTGTTGCAGGGAT
ATGATGCATATAGTATTTGTTCACTACTTGGATGTTAAGGTATTTTGCTTGTTTTCTTTATTTCACAC
TTTTTTATTAAGATATTTTGCTTCCTTACTAGAGCAAGAAATGTATCATTGCTTGGTTTTGTTGCCG
GTGCAGTTTTGACCTGAGTGTGTATGACATCTAGTAGCTCAAACCTAGTTTGTTCTGAGTTTTTTTT
GTCCTCAAGACTTCCCTCTAGAATTGAAGGAAGAAATAGAGTACTAGTGTACTACTGTACAAGTA
CTATTTTCATTCCCTTAAACATTGACTATATGTGAACAAATAATCTATCTTCCATCTGACTTCCTTC
TAAGTTTCTTACCATCATGGATTGGAGAATGTGCTTTTTTAATTGTAAGCCTGGATAGCTATGTTTA
AGCATTATAACATCCACCAAATGCTAACATACTAACATTCAAATTTGCTCTTCTGCTACTAAATTT
ATGTTTTTCACTTTTTACCATGAAAAGACATCCTTGGTTTTTAATCTTCAGAAATCCCATTTGATGA
TATTGAGTAAGTTGCATAATCCTTTTGAAAAGATCCTGAACTCTGTTTCTATAATATTCTAGAAAT
CAGGCAGAAAACTTTTGAATTCAATATTGACTCACTTGTTTGATCATTGATGACTTAGTATTAATA
GGTGTTTTGTGGGTTCTTCACCTTTCAGGTTAACAAAACCAATGTTGGTGGAAAAACATATTCTGA
CGAGGTTACATCAGATTCCCAGAAGAGCAGTTCTTTGTCATCTGGCTTTCCCAGAAATTATGGTA
GTGTGCCTTCTGGAAGTACAGATTCCATGAGCCCAACAAGCACTTTGACATCTTTATGTGAAGAT
GCTGATTCAGGTAATTCTGATATATATTTAATGGTTGTCACACTTAATTCTTTTGATCTGAATTAAC
ATGCCAAGGCTTTAAATATCTTGGTTTACTGTTATGCCATGCTCACATGTTTGGTGCATTTGGTCTA
ACTGGTTTAATCAAACTTCTTCCTAGAGGATATTCATCAAGCAAGTTCAGGATTACACTCCTATCG
TGAATCACAAAATCTGGGGAATGATCGTCCGATGGATAAGATTGATGCTCGTTCAAATAGTTCAT
ATCCTATGCATCCGTTTTCAGGTTACCTCATCTTTTTATTCTTATTTGTCTTAGTCTCTAAGATGATT
ATTTTGTAACTTCTATTTTGTTTAAATTATATAGTTTTATGTATAGAGGGAGGATAAGGAGACTGG
GAGACTGAATACATTGATACTGATATCAACTTTATTTGATATGATGTTTTACCTGTGTTCAGAAAT
GATACAAAACACAAAATACTAATCCCTATTTAAATAAGAAAGCAAATAACAAGAAAATAAGGA
AATATGGAAACTAGTCTTAAGATGCTCTAAATTAATATACAATCAAGATATTGGATATTTTTTTGT
ATATTCTCACATATCTAATGTCTTAAATAACATCTGCATTAATAAGCCTTTTCTGGGATTTGACATT
TGAATTTTTTTGTATGTTTTGAAACGTTTTCAGGTGATCATGGGCAGTTACCAGTTTCTGGAGCAG
AATATATCCCACATGTTCTGGGGGATAAGTCTAGAGCTAGTGATACTACTTACATTGAGGGTCAG
AGAGCACAGGGCATTGCATCTTGGGACAATACCATGGAACAGTCTGCAGGAGAGTATGCTGATC
CTTCTCTTGTATCATCTACAACCATTCCATCAAGTGCAGTGGGAAACATCCTTGAAGAAAACCAT
ACTGTTCCTGGCAAACTTTTAGGGCGTAAAAATGCCCTAACTGAAGAGGAAAGGGGCTCTCAGC
CTGTCCAATCAAATTGGCAGGTACTTACTGTTAAATTTCTCGCTATATCTTCCCGTAGTGTACTCTT
GCTTGCATGCATACATCTGATTTCTCAGCATAAAGATGAGTATTATTTGAGGTTCTATTAGGAACA
GTTGTGCTATCTTGTTGTATGGGACAATATCTGGAATTTTCTGTCCCAGAAACCATAGTTTCTGCA
ATTGCGGTGGAGTGGCACCCTGCTCCTCCTCCAAGGGCAGTCACGTCGTGGTGTGGTGCATTCCA
TGGCAAATAGCGGGCGCTTTTCAGAGGCATCAAATCTTCATGCTTCTTAGCAATCTATTCATTTGT
TCTAGAGCACCTGCGCCTTCTTCTTGCTTCTGTTTTGCACTTCTGCAACATCTTCCTGTCATCAACC
TCTGTTCTGCAACTTCCAGCAATGCAAAAAATAGAGCAACGATCATGCTATCCATTATCCTGCTA
TAGCATTTTTTCCCTGGGGGGCTTTCATCTTTGAAAAAGAAATAATTGCTTGAGGATGGGGAAGA
TGAAAATTTGTGGGAGTTGTTGCTTGATATTAAAAGAAATTAAGAGATTCTAGAAGGTGGATTTA
TGGAAAAAGTGGCCTGGGAATACAGTTACTTTTTTTTTCCAGGAATTAGTCATTTAGCCATGTATT
TTAATAAGTTGCAATACAATATAATGATGTATTGGGATGGAGCCTATTCATAACTTTGTGTTACTG
GTGTACATGTGTAGCACTGAATGTTTTGAATTGTTTCTGAACTTGTAGAAATTCTAAAGAACATAG
TAGCGTTTATCTTAAATTTTTATTACAGACTTTCTTTTTTACTTTATGTTTGTCCTAATGTATCCTTAT

TTAATCTGCTGAGATGAGAATTAAGTAGTTTGATGGGGGGAGTGGCCTTGCATTGGTTTGATGTC
AATTTTATATTTGATCTGTAACCCTGATGTTTTAACTTGTAGTGGTATCTCATCAAAATCTTTTATC
TGCACTAGTTTGTGAGATTTAGCATTGATATATAACTATTTTTATTTTGTTATTTTAATTGTTGATTC
AGATTCCTTTTGAAGACAATACAGGAGAATTGCCTAATTGGGGCTTCACCCAATCATTGGGTTTG
GAATTTGGATCTGATTATGGTGCTAGTTTACTGGGGGATGTAACTAATAATGCAGGCCCAGAAAT
TGTTCCTGAGCTGTTCACTTTTAATGGTGAGTTAAAAGAGCAGTCTGTGCACCAAAACTTCTCAAA
GCTGTATACACATGGGCAATCTCAACCTACACTGAAATCCAATTCTGAATATGAAGTTCCTGGTG
AAGCAAGTATCAATTATGCCTTGACCATGAGGCGTGGATTATTGGATGGAGAGGAGAGCCTCAA
GAAGGTTGATAGTTTCTCTAGGTGGATGACTAAAGAATTTGCAGGTGTGGATGATCTGCATATGC
AGTCTTCCCCTGGTATTTCATGGAGCACAGATGAGTGTGGGGATGTGATAGATGATACCTCATTG
AATCTGTCCCTGTCTCAAGACCAGCTGTTTAGCATAAATGATTTTTCTCCTAAATGGGCTTATGCT
GAATCAGAAATTGAGGTACTTTGTTTGAGATTCACACAATTATAATATATATTACTTATGGTTGTG
CTGTTTGTTTTTCTTTTCTTGTATGTCGTGAGCGTATGTATGGCACTTGGAATCTACTTTAGATTAAT
ATCTTTTGCCTCGGATATTGACGGCTTTTTTTTGAGGTCAGATGCATATAGTTTTGTTCTTTATTTAT
TTGGATATTCCCAATGTGTGTCTGTTCTCTTTGTTAATGAATATTTCTTGTATTTCATGATATATCCT
GTTTATATTCTTTATTTGCTTTGGAGTTTTATACTTATGGTTTACTCATTTTTCTATGGTTTTCTACCA
CAACATGAAATGAAGAGAAGTCAGCTGTAATTTGACTGATATCTTATCCTATTCAAAATTATACA
GGTGTTGATTGTTGGAACATTTTTGAAGAGTCAACCTGTGGTGGCAAAATGTAACTGGTCCTGTAT
GTTTGGGGAAGTGGAGGTTCCTGCTGAGGTTTTAGCTGATGGAATTCTGTGCTGTCAAGCTCCACC
TCATAAGATTGGACGGGTTCCCTTCTATGTAACATGTTCCAATAGGTTTGCTTGTAGTGAAGTTCG
GGAATTTGAGTACAGAGAGGGCTTTGATAGAAATATACAATTTGCAGATTGTTTTAACAATTCAA
CTGAAATGGTGCTTCATTTGCGACTGGTGGGGTTACTTTCTTTAAACTCAGTACGCACTTCAAATC
AAGTGTTTGAGGGTGATATGGATAAAAGAAGCTTAATATTCAAGCTTATTTCACTGAAAGAGGA
AGAGGAATATTCAAGTAAGGAAGAGACAACTGCAGAGATGGATATATCAAAACACAAGTTGAA
GGAGCTTATGTTTCACAAGCAGGTTAAGGAGAAGTTGTACTCATGGCTTCTTCACAAAGTTACTG
AAACTGGTAAAGGGCCTCTTGTATTAGATGAAGAAGGGCAAGGCGTGTTACACTTAATTGCTGCT
CTTGGTTATGATTGGGCCATAAATCCAATCATAACTGCTGGAGTCAATATCAACTTCCGTGATGTG
AATGGATGGACAGCTCTACACTGGGCTGCATTCTGTGGCCGGTAAAGACCTAATTTCTTTTATTTG
TTTTGAGTTGATTTGGTGTATGTTGGAACTACTTATCTTGTTGAAACCTCTTTGGCTACCCTGATTA
TTTCATTTTTCCAGAGTTTTGGAATTTTGGATTTGATTAAATATGAAACCATTAGGTGGTATGAAA
ATTCTGGCCCCTTTCCTGTAGGAAATGTCTTTCTTTCTTTTTTCATTGTTAATATTTATTCTATTTTGA
TGCCAAGGTCGGTGGATTTTTTATACATGTGAATTTGTTGAAAATTAATTTTAAAGCAATTGCTTT
GGTCAGTTAAAGACAAATTTAAAGCTATATTCTGTGATGCTTTCAGCATTAGAGTATTCCTTTTCA
AAAATGTCCTCAGATATATTATTTTCGTTCAGCATAACATGAATTGGTTCCTTATATTATTATTTGT
CCTCTGCTCAGAGAGCGAACAGTTGCTGTCCTTGTCTCCATGGGTGCAGCAGCTGGAGCATGGAC
AGATCCATGTCCAGAATTTCCTTCGGGTAGATCACCTGCAGATCTTGCTTCTAGCAAAGGCCATA
AAGGAATCTCTGGTTTTCTTGCTGAGTCATTATTAACTGGCCACCTTGAATCACTCACAATGGATG
AAAATAAAGATGGTAGGAAAGAAACTTCAGGAACGAAAGTAGTGCAAACAGCTTCAGAGCGAA
CTGCGACACCTGTGCTTTATGGTGATATACCAGATGCTATCTGCCTTAAGGATTCTCTTAATGCTG
TCCGTAATGCTACACAAGCTGCTGATCGTATATATCAAGTATTCAGAATGCAGTCATTTCAGAGG
AAGCAGTTTGCTCAGTATGAAGATGATGAATTTGGATTGTCAGATCAACAAGCTCTTTCTCTCCTA
GCTTCTAAGACGTGCAAATCTGGTCAAGGAGAGGGTTTAGCCAATGCTGCTGCAATACAAATAC
AGAAAAAGTTTCGTGGTTGGACGAAGAGAAAAGAATTCTTGATCATTCGCCAAAGAATTGTTAA
AATCCAGGTTCATTGTTCTGCTCTCTTTAGTTTCCTGTCTGTCTATATTTTAATTAGGTAAATTATAA
ATTTAGTCGCAAAGGACGAGCCCTGGTGCAGCGGTAAAGTTGTGCCTTGGTGACTTGTTGGTCAT
GGGTTCGAATCCGGAAACAGTCTCTTTGCATATGCAAGGTTAAGGCTGCGTACAACATCCCTCCC
CCATAAATGGGGTACGAAGTTTTTTATAAATTTAGTCGCAATATTTTTGTTTGGTCCCTACATGAA
AAATTGCTTACAAATTAGTCCTTACTGTTTAAGTCTTAATGGTGTTAGTTTTTAGTTTTTTTCATATA

AAACTAAAAATGATCTGTCGGGTGTAGGGAAGTGTAAAATTTTCGCTTTATAACTAAAATGAAA
AACGATGTTATTTTGTAAAATAGAGAGGGGCCGAATCTAATACCGTTGTAATGTTTCCAAACAGC
AGGGATCAAATCATAAATGTTTGTTCATGTAGGAACTAAAATGCAGAATTTATGTAAGGTGTAGA
AACTAATTTTTTAATTTTACCTTCTTAATATTATGGTGGTTCATTTTACTTTGACGTTGATTTCTCAT
TGAACATCCTATCAACTATTCTGCTATTTAGTTTCACCTGATAACATTGCATTGGAGGGTTCAATT
GATGAAAACTTAGGGTTCAAATACTTCGCCATTTCTGTTTGCCCTTTATATAACTTGCATGCACGT
AGAGATCACGTTGGGTGTCAATATTGGTAAAGAAATCAGAGGTTAATAGAAATCATGTTTCGCTA
TGGTGTTCAAGGCCCATGTAAGAGGTCACCAGGTCAGAAAGCAGTATAAACCAATCATTTGGTC
CGTGGGAATCCTGGAAAAGGTCATATTACGCTGGAGACGCAAAGGCAGTGGCTTACGTGGATTT
CGACCGGCTGCCCTGAACAAGGTCCCTGAACAACCAAGCGAATCTCCGAAAGAGGATGACTATG
ATTATCTCAAGGAAGGAAGGAAGCAGAGTGAAGTAAAGTTCAAAAAGGCCCTTTCACGGGTGA
AGTCCATGGTTCAGTATCCAGAGGCACGGGCTCAATACCGGCGGGTGCTAAATGTAGTTGAGGA
CTTCCGTCAAACCAAGGTATTTTGATTTGATTGTCTAACTTATGTCTTGTCCTACTTATAAACTCAT
GATGGATACTTGGTTTAATGATATGTTCTTTTGATGTCCTACCAGGGAGGTAATTTGAATTTGATA
AATTCGGAAGAGACAGTTGATGGTGTGGAGGATTTGATTGATATTGACATGCTTTTAGATGATGA
AAATTTCTTACCTATAGCATTCGATTGATTCTCTGCTGTTGCTCCACCCACCCCCGGTAAATAGAT
GGCCAGTTGGTTTTATTTGGCATTGGAGAGATCCTAACATGGTGTGCTGCTGTATATATATTTATA
TTTATATATAATATACAATCAGAACTACTTGGATCATAGGTCTTCCTGTTTTCAAGCAAAAAACTA
ATTAGATCCAAGGGTGGGTTTATAAATAGATTATGAAACCTAGAATGCGTTAAGGTTTGATAGTT
CAAATACATGATAGACATGGTTTAATTTTATATATGATGATTTGTATATGACATGATTATTTTTATT
TAGATCGAAATATGCTTTTAATTCTTAAATTTGTATCATTTTTT

>Glyma.08G178900 (GmCAMTA6)

AAAAAGTTCATTCTATCAGTTTTTCACTTTATCGCGTCAGTTGAGACTTGAGAAACAGTCCAGGA
GAGAAAAAAAAGGATAAAAAGAAAAGTCTCTGTCGTCCTTCGTCGTTCGCGTTGATCTCAGATTC
AGTCTTCGCCATGGCGGAGGGTGCTTCTTACGGATTGCGTCGTTCATTAGGTCATTTTCTAATTATT
TTTTTTCTTTTGCTTCATAATTTCTCATTCAATGCTTCGATCCTGCGAATTTAATTGTTTTTTTTCGAC
GCGATTTCTTGTCATGGTGCTTCAGCTATTGAATTCTGTTTCCTTCGTTTAGTGGGTTCCTGCTGTTG
GGACTTAGTTTCAGTTACTAATGATGACTTCGCTAATGAATGGACCTAACGTTCGGTCCTTACTGA
GGTTGACAAAAATTGTGCCCAATTGATTTGGGGGAAATAACTATTTAGGGATATTTTTTAGTCCA
ATTATGTTTAAAGTGTCTTTGGCACTAGGTTTGAACGACATGAACCATTGATAGGTACTGCTTCAC
TGCATCAGTTTTCCACTTGTTCGGGTTCTGAGTGGTGAAACTTTTTTTTTTTTATCGGCAAAATTGTT
AATTGTTGGTTTTTTGTTAGTTGAGGGAATCAAACCCACAACCTTTCCCTCCCTTCTCCTTTTACCA
CCAAACCAACCTTATAACTTCCTCTTTGTGGTGAAACTAACTAAGATGCAGCTCCAATCCCCCCA
AAGGAACTAAAGCTAGTATTGTCAAATTTGTAACAATTTCAAAACGATTTAGTTGGATGTAGAAA
GCACCATGCATTTGAGGGATTTTCCTTGCAGAGAAAAACTGAAAGAAGCATCAATTTTTAGAAA
GTTAGATTTATTCATGGAGACAAAATTAAAGGATATCATTTTTCAAATTTTTTATGAATCCTATTCT
TATTGGTGCAAAATGTAAAAGAAAAAGTTTGATTCAGATAAGTGGTATTTGATAATGTCTGACAT
GAATGTATGTTGAGCCCTGGTATGGTAAGGTAAGGCTGCTGTTTCAACTGTTCTTGCTTCCTTTTAT
GGTATGCAAAGAAGTTTGAACTTTAAATGAAGATTCATTGGGGAAAGAAAAAATGAATTTAAGG
ATACATTGCTTCAAATTATAACATAGATAGTGGCCATGTCGGTAAATTAGTTCTATAAATAAAGA
GATCTGATGGCTAAAAGAAGAGCCCAGTGGAATCAACAATTTTCATTCAGACAAAAATAGTGGA
GTAGATTTTCATTTGTTCTCTCTTAAATGGTGAGTTGCTATCATGAACCTCTAGCCTTGTATTTACTT
CAAACTTACACATAGCCATCAAAAGTGAAAGTTAAACTGAAGGTTTGTAGCTGACATGTGATTTA
ATTTTTTGAAGCAAGCCAATACCTCCTTTCAGGATATGATGTTACACCTTAACTTGTTGGTGTTTTC
TTGAGGTTGTACATATTTATCATACAGATGATTGACATATTATTTTTTTGAGTTAGATAGAAAATG
GTTGGCGGGTTCTGGCCATACCTGCAAACAATCATTAGCTGGATCAGAGAAGAGGATGTAGTGA

TTTGGGTATTATGGGGCGCCTAATTTCTACATTGAGTAGTATAAGATGCTCAGTGAAGGACTTAA
GTGCCTTGGTTCTCCCCCCCCCCCCCCTTGGTTGCTAGCTTTGAAGCGTGGGTTCTCTAAGTGTTTG
GATGCTTATCATGTAGTGTCTGCTTGTATTTCATTAGTTTTACTCAAAACCAAATTTGTCAAAATTG
CGTTAAAGATTGCAGGATTGCATGATCCTATGATTTTCATGCGTGCATGATTGGTTGCAGGGTTGT
TTTACTGCAAGATACTTTTGAATTGCAATTTTCCTGGTCCAAGATTCCACAATCTGTGGCATCTCC
ATCTTCCTCTGGATCAGGTTTTGAAATGAATCCCATTTACTGGACCTGACGCATTTCAGATGCCCT
CTCTTATTGCTCTCTGATTTTCAGTCTCTCACACTCTCAGACTCTTGCTTGCTTCACCTCATCTCAGT
CTTGCTCTTGTTTGCTTTGTCACTGCCATATTACGCTGGTGTTCTTCTTCTTCTTGCCTTCTTCTGCTG
TGCCTTGCGTCTTGTGTCTTACCTCTGTGTTGATTACAAGCATATTCTTCTGCGGTTCTGCCTCTCTC
ATTCTCTGTATACAATTAATATAGTTATCAAGAATCCCTAAAATTGTAGTCACTTTGACTGTGCAG
TATTTATTTTAACTTATAATAACAGGGTATCTATAGATCACCCTTAACAAAATCAGTATTTGTCAA
GTTATCCTGTGTACATAACACGTACATTATGTAGGTTAATATGAACATAAGGGAAGCTATCTATA
TAAATCTATGAATATATATTTTCAGTTTAAGTTTAGTTCAAAGATATCATTTACAAGATATTTTCCT
TATCCATCCTTTTTATCACATGTATATCTTCATATATAATTTTGCCTCTTTTGGTAGGATCTTTCGAT
TTATGATCTTTCATCCCCCTCCTGATCTCGTGTAAGATCTTGATTTTGACAACATTGCTCAAAACA
ACTTTTCAGACAAAAACTCCACAACATTTTGATTTTAAACTAATTTTAACACAAGCTCTTCCACAT
AATCACATAAAAAGACTAAAATCAAACTGATTCTGTTGAAAGGAGTATATTTAGTAGATAAATT
GATTTTTGAGGGGACTAATAGGCTGCTAAGTTTATGTACTGGATACTTTGACCTCAATATTGGACT
CATGATTGACAATGTGGGTGTTCTTTTCATGTCAGTTTATCTTTCTCTTTTTGATTCTATAACAAAA
ATATTCTTATTGATGCATGTGACTTTTGTGGCCATGATTTGATTATTTAAGCTTGCTTGAATAATTG
CGTATTTGTGTATGTGATATAATTCAAAAATCAAATATTTAACTGAATGCCTTATATCAATGTGCA
GATATTCAGCAATTACAATTTGAAGCCCAGCATCGATGGTTGCGACCTGCAGAAATTTGTGAAAT
TCTTCGTAATTATCGGATGTTTCATATCACATCAGAGCCTCATAACAGGCCACCAAGTATGGTTAC
TGCATGTTACTGATAAATTAATTGGCACCTGTATATACTATTATATAATTTTTTACATGTATGCATT
GGATGAATTGTTCAAGAAGCTTACACCAATACTGATTTTCAATGATGTGTTTGTTGCAACTTGGTA
TAAATGAACTTTTTGGCCGAAACTATTAACATCTTTCTAGGTGCCTAACAATGTGTTTCAACATGG
GAGTATTGCCCTCCTTGGGTTCACTTAGCTGAATTTAATTCCTTTTAAAGATGTATCAAGCATTTTA
ATTTTGCTTTATCTGAACTTAAGGTATTCATGTTCCAATAGATCCTATATATTCAGTTCAAGGAAT
ACATGCACATTGGGTAATTCAGTAAATATTTTTTCATGAAGTTTGGTAGCAAAAAAGCTCCTGAT
ATGCCTATTGAATTAACTTTGCAATTCAGTAGTTTTAATGATATTATTAATAGTATTTTGTTGTTTTG
CTAAAATCTTGTGTTGTTGTTAACTTCAATCCATCTTTTAGCTCCTAATTGTATTCCTATCATTACTT
CATGCTTACTAAGACCTCACTTAATGACTTGTATTTCATGATTTCAGGTGGTTCACTTTTTCTTTTTG
ATCGAAAAGTTTTGAGATACTTTAGAAAGGATGGGCATAATTGGAGAAAGAAAAAGGATGGAA
AAACTGTGAAAGAAGCTCATGAAAAGTTGAAGGTAAGCTTATAATTTGCTTCAGTTTTGAGTTTG
TAGTTAATCAAATTTTTGTAAGACTAGCATATATATAGGATGATTGGATATTTTTTTTCCAATTGG
AAAGCAAGTCTTTATACCATTATGCAATTATTCACTGGAGTTGTAACATCATTTAAGCAAATTTAG
ATTGGAAGTGTTGATGTGTTGCACTGCTACTATGCCCATGGAGAAGAAAATGAAAATTTTCAAAG
GCGAAGCTACTGGATGCTTGAACCGTGAGTATGAATTTACATACAAGATTGTGTAATATGCCTTG
GAAATTAAAGTACATTCATATTTCATGTGTTTGTTGCTTTGTAAGTTTCTTAGCAAGTACTGCATTG
GTGCATTTGCTGTCATATCTCATGTAAATGTACATATACAAGTTTTTAAGGTTGTTCTTTACCCTCA
AACTTAGTGAATAGAGCTACATTTTTGGCTTTTCTTTGGTGTTTGCATGTCGATTGAGAAGCTCTAC
TGCATTCTTCTTCCCTGTTCTCCTTTGTTTTTAATGTTTTTGAAAGATTTCTTATCCTATCCGTAGTTG
TTACCCACTAATCCACTTATTTCTCCCTAATATAATTTCCTTTTTATCTAATAAGTTATAACAGTTTT
TAATGTTATTGGGTTGCAAATATTTTGCTGCATCTTAGCTAATTTGAGTTTTCAATGTACATCTACT
TAAATATTTTTTGTCTTCTTACAATTAATCAGAATATGGTTTTCTTTTATTGCAGGGATATGATGCA
CATAGTATTTGTCCATTACTTGGAAGTTAAGGTATTTTGCTCATTTGTTTATATTTTTGCTGTTATTA
CACTTTCTTGTTATTACCAATGGTTGTGTTTTTATCAATTACAAGTTATAACTTTATTTCTGCAGTGT
TATGAGTCAAGAGTTGCACCTCTGCCAACATCTTAAGCTTTTGGCATGGTGATAAAAGCACTCTT

GGTCATATCATACCATCCTTTTTTAAAAGATCTTGAAATCTATTTCTATCATATTCTTGTTGCGATC
AAGCATAAAGAATTTGAAGCTGTTCAATGTAGAAGAATATGTGTTTTGTAGTTCTGATCTTGATTG
ACTTGAGTGCATGGGTGACTTATTCATATATTTCTTCTGGCTTTTTATTTCTCAGGGTAACAAAAAT
ATTGTAGTAAATACTGAAGGTGATGAGATCCCATCAGATTCCCAAAAGGTAACTTCTTCATCGTC
TAGCCTTCCCACACATCATAGCAGTGTTCCTTCTCTGAGTACAGATTCCGTGAGCCCAACAACCA
GTTTGATGTCTTTACGTGAAGATGCTGATTCTGGTAATTCTGCTTAAATTTATTTCACAAGGGTTCA
AAATTTTTATGGTCTGTGAAGTGAATTATATGCTAAGGGCCTGTTTGGATTAGTTTCTCCATAAAC
AATTTTAGGAGAAGAAAATAAGAAGGAAAAAATGAAACGAGCTTTTCCATAAGCTAAAATTAGAT
TATGTATAAGTTAATTTGTAGAAGCTCTCTCATATTAGTTTCTCTAAAAGATGATTTTTAACTTATG
CATAAGCTAATTTAAGCTTATGGAGAAACTTATTTCATTTTTCTTCTCCTATAAGTGTTTATGGAGA
AGTTTATTGGAACATGCCCCAAGTCTTTAACTATCTTGGTGTACTGTTACAACAAATATTTGTGTA
ATAGTTTGGCATTTGGCCCATTTGATCACTCTTAAATGATTTAATCATAATTCTTTCCAGAGGATAT
CCACCAAGCAAGTTCTGGGTTGCGCCCATTATATGAGTCACAACATAGTGGGAATGGTCCATTGA
CGGAAAAGATTGGTGCTGGTTCGAACAGTTCATATCTTATTCATCCCTTTTCAGGTTACCTTCCCT
CCTTTTTCTTCAATTTTGTTGTCTCACATGTATATTATAACTTCTATTAGGCAACCTTTGACATGTGT
TTCCCCCATATATATATATATATATATATATATATATCTTAAAATATTTTTAGGTGATTATGAACAG
TCATCAATTTCTGGAACGGATTATATCCCTGTTGTTCATGGGGATAAATTTAGAGGAAATGATACT
GCTTATATTGATGGTCAGAAAACACATGACGTGGCAACATGGAGCACTGTCTTGCAAAGCACTG
CCAAATTGCATAATGATCCTTCTCTTGCATCATCTCCTTCCATTCCATCTAGTTCAATGGGAGATGT
CCTTGAACAAGAACATACCATTTTTAGTGACCTTTTAATGAGCAAAAGTGGTCTCACTGAGGTAG
CAGAGAGCTCTCAGTCTCTCCAATCAAATTGGCAGGTACATACTGTTACCGCATAGTATATCTAT
CAGTAGAATTATCTCACATATACGGCTATTATTTTTGAGGATGGAAAAGACATAATTTGACAGTT
TTATGGGACTAAAGCTTGTGCACACTTGAACTGCTATTTTCATTAATAATTTTAGTTTAACTTAATG
ATGAATATATTAACTATCTGGTGTCGGGCTGCATTTGTCATATTAAAAGGTTTTGAATTGTTTCTG
AAAGTCTAGAAATGCTATTGTTGGGAAAAACCCAAGTCCGGCATAGAGATAGTGTCAAGATAGA
GTATATAAGTGGGGGGTAACTCTCACCTTATAAGCCGATTTTGTAGGGTTGAGTTAAAACCAAAC
CCAAATTCTAAGAGCCATAATTCCAGATTACAATTTTTTTAGTAACTCGAACTTTCTCCAGATGTA
TCCGTATCCAATCTTTTTGTTGAGATAGGTATTTATTTGCTTGAACTCTTCGTAAATAACTTGTTGG
GTAACCATGACTGGGTGTTGGTTTTCATAATATATTTTAATTTGTTGGCTTTAAAAAAATGACTGG
GGGTTGGCTTGGTGTCAATTGTATTTTTTGATTGCTTAATTTGAAACTGATACTTTTAACTTTGGTTT
CTCTTATATGTAATTTCAAACTACTACTACTAATGAGATTTTGCTGAAAAATATAACTATTTATTTT
TTTATTTAATTTGACTCAGATTCCTTTTGAAGACAATTCAGGAGGCATGCCAATGTTAACCCAAAC
CCAGACATTTGGTCTGCAATTTAGATCTGATTATGGCACCGGTTTACTAGGGAATGAAACTCGTA
ATGCATGTTCTGAAAGTGCTGCGATCCTGTACAGTTTTAATGGTGAACCAAAAGAGCAGCCTATG
CAGCAAAACTATCTACAAGAGCTTGAAGATGGACAATCTCAACATGCACTAAAATCCAATTCTG
CAAATAAAGTTCCTGATGAAGAAACTATCAACTATGGCTTAACAGTGAAGCGCACATTATTGGA
CAAAGACGAGAGCCTGAAGAAGGTTGATAGCTTCTCTCGGTGGATTACTAAGGAACTTGGAGAG
GTGGCTGATTTGAATATGCAGTCCTCACCTGGTATTTCATGGAGTACAGATGAATGCCAGCATGT
GATAGATGATACCTCATTGAGCCCATCTCTCTCTCAAGATCAACTGTTTAGTATAAATGATTTTTC
ACCTAAATGGGCTTATGCTGAGTCCGAAATTGAGGTACTGATCTTGTGGAAAATCTATATTATTAT
TATTGTTGTTGTTAAAAAATGTCAGATTCCATTACAATCGAAAAAATAATTTTTTTCTGCCAACTG
TTTACTGGTTCTGTGGTCTCCTTTTCTCCCTTCCTATTAGTTATTATATGAATAAGTACTTAATATGT
ATACATGTCATTTTCATTGACCATAAGAACTTTTTCTTTTCTCCTCAAATATCAAATGCTTGTCCTA
GTCAGATATTTGCAGTTTTTGCTCTTAATTTTTTAATTTGTAATGCCCTCCATTTTGTATTTATTTTTG
TATTTCTTCTGCATCTTGGTATTTCATAACACATCTGACACTCTTCCTTCTTTTCCCCCTCTCCTTTT
GTATGTTATATATGATTAATGGTTTTACTTCTTTTTCTAGTTGAGATTGAGTCTATTGCAAGATAAA
ATGAAGAAGAATAGGATGTAGTTTGATTGACATCTTATCCTGTTCAAAACTATACAGGTGTTGAT
TATTGGATCATTTTTGAAGAGTCAACCAGAGGTGACAACCTGCAACTGGTCCTGTATGTTTGGGG

AAGTTGAGATTCCCGCCAAGGTCTTAGCTGATGGAATTTTGTGCTGTCAAGCTCCGCGTCATAAG
GTTGGACGGGTCCCTTTCTATGTGACATGTTCCAATAGGTTAGCTTGTAGTGAAGTGCGGGAATTC
GATTTCAGAGAGGGCTTTGCCAGAAATGTAGATTTTGCAGATTTTTATAACAGTTCTGAAATTCTG
CTTCATTTGCGACTAGAGGATTTTCTTTCTTTAAAGCCAGTGGACCCTTCAAATCATTCTTTTGAGG
GTGATATGGAGAAAAGAAACTTGATTTTTCAGCTCATTTCACTGAGAGAGGTGGAGGAATATTCA
ATTAAGGATGAAGTGACTACAGAATTGGATATATCCCAACACATGGTGAAGGAGCATCTGTTTC
ACAAGCAGTTTAAAGAGAAGCTGTACTCATGGCTTCTTCACAAAGTAACTGAGAGTGGTAAAGG
GCCAAATGTATTAGATGAGGATGGGCAAGGTGTGCTACATTTAGCAGCTTTTCTTGGCTATGATT
GGGCCATAAATCCAATTATATCTGCTGGAGTTAATATCAACTTCCGAGATGTGAATGGATGGACA
GCTCTTCATTGGGCTGCATCCTGTGGCAGGTAAGGTTCCTTGAGATTTTTTTCCTCTTTATTGAGTA
CAGTTTGTTGAACATAAGTTATGTCTGTTAATCTTGTTGAACCTTCATTTACCGCAACATATGAAA
CAAATCTAAGTTTCTTTCTTTAAAGTGTGGTTAAGAAATATCCAGTGTTTTAGATGAATGGACTTG
TTCCCCTCCTCTCCCTCCCCTAATATTTAGATTGTTTTAGTTCTAAACGTTGCTTCAGTTTTTATTGT
GTGTGTAAAATAGTAAATTAGCTAACTTAGAGTTATCCCTAGAGCAATTGCTTTGGTTAGTCAAA
GGCAAATCACAATCTGCTTTCAGCATTGACTTTCTCAAATATATTATGTTCAGCATAACATGATTT
TTTTTCTTGTTATTCTTTGTCCTTTTATCAGAGAGCGCACAGTTGCTGTCCTTGTCTCCATGGGTGCA
GATTGTGGAGCATTGACAGATCCATCTCCCTCATCTCCAGCAGGTAGAACAGCTGCAGATCTTGC
CTCTAGCTATGGGCACAAAGGAATTTCTGGTTTTCTTGCCGAGTCTTCATTAACTCACCACCTCGA
AACCCTCACAATGGATGATCAAAAAGGTGGTCGGCAAGAAATTTCAGGAATGAAAGCGGTGCA
GACAGTTTCAGAACGATCTGCAACACCCGTGCACTTTGGTGATATGCCAGATCTCTGCCTCAAGG
ATTCGCTTACTGCTGTGCGTAATGCAACACAAGCTGCTGATCGTATACATCAAGTATACAGAATG
CAGTCATTCCAGAGGAAGCAGTTAACTCAGTATGAAAGTGATGAGCTTGGATTGTCAGATCAGC
AAGCACTTTCCCTTCTAGCTTCCAGAGCATGCAAATCTGGACAAGGAGATGGTTTAGCCAATGCT
GCTGCAGTACAGATACAGAAAAAGTTTCGTGGTTGGAAGAAGAGACAAGAATTCTTGATGATTC
GTCAAAGAGTTGTTAAAATCCAGCTACGTTGATCTGCTCTTTTTACTTGATTTTTCATTTTCATTATT
CTGGACTTTTTTTTAAGTAAACATGTATATTGTTAGATGGTCTTAATTTTTCATCAAATCATCTTAC
TAATTATTCTGCTATTTTGGAAATTAAAGGTGGAGAATTGATTGCATTGAAGATTTTGATTGATAA
AAACTCTGGATAAGAATGGAAAAATGCTTCTTAAACTTTTGCATCTCTTTGCTCCTACTATAACTA
GTGTATACATGTGGATCACACATTGGGATTGTCAAAAACAAAAACTGGAGAAAACAATCCAGAA
TGTGAGGAAAGCATGCAGTAAAATGTTCTTTATATTGGGTTCTTCCTTCCATCATTCCATGCATTT
ACTTAGTGATTTGGTGTTGTGTTAACATTCATCATATCATGAGCTTGCGCACCTAGTGGGCCTTAA
AAGCATTTAACTCCCAAGAAAGTACACCAAAAGATATCATATATAGCACTTGTTGCCTAAAAGT
CCTTCCTTATCCTCCTGCCACGACCTGTAAAGTAGAAAATTCAATTTGAGGACACAACTTATATTC
ACAAAAAATTAAGAAAAAAAGATTTAGGAAGCTACCAGAAAAGATACAGTGACTTCCCAAAGC
TTTAAGTTCAATTATCTCTGGCCTGAGTCTTTTAAGACTTAAGTGTGAAGTTGATGTTTGCCAAGC
AACTTTACCCTTCCCCTACTTTGCTACTTGTACCTCCAATGTAACCTCCACCTGGCTCCCTTTCCCA
AAAAGAAAATATGACTACTTCAGAGATTTTCCTGCTTTTTTTTTTGGTTTTTTTTCCTATCTTTCATT
TTTTTTTTGTGTCCCTATGCACTTGGCACATGCTTCCACTGACTGTTGAAATCTGTGGTGTCAAGGC
CCATGTAAGAGGTCACCAAGTAAGGAAACAGTATAAACCAATCATCTGGTCTGTGGGAATCCTG
GAGAAGATCATATTACGTTGGAGGCGCAAGGGTAGTGGTTTACGTGGATTTCGACCAAATGTCAT
TAATGAAGTCCCCGATCAACAAAATAATTCTCTGAAAGAGGATGATTATGATTATCTCAAGGAG
GGACGGAAACAAAAGGAAGAAAAGATCCAAAAGGCCCTATCAAGGGTGAAGTCCATGGCTCAG
TATCCAGAGGCACGTGCTCAATACCGGCGTCTGCTAAATGTTGTTGAGGACTTCCGTCAAACCAA
GGTATTTGTTAACATCTGGCAGGTGTCTAAACACTTTAATGTTTGGCTTAATGATATGCTCATAGT
TGTTTGATATCTGGCAGGCATCTAACGAGGGTTTGATCAATTCTGAAGAGACAGTTGATGGTATG
GAGGATTTGATTGATATTGATATGCTTTTAGACGATGATAATTTCATTCCTATAGCATTTGATTGAT
CCTGCAATGGCTACTCTCATCGTCCCCACTGTAAATACATGGCCTGTTTTTGGCTTTATTTGGTATT
GGGGAGTCCTAACATCATCTTGATGTAGTATATATTTCTAATCAAAATTACTTGGATCCAAGGCTG

CATATAAATAGATTATGATAGCTAGAATGCACTCTGATTTGACAGTTCAGGTATTGTAGTCTAATG
CAACATGGTTTGATTTTATTTATGATTATTTTAGTATGACTGTTTATAGATGATTGTTTTTTTTGTTTT
GATAGGCAGACGGTTGGGGGTTATAACATAATGATGTAACTTGCTAAAGTATTAAACTGCAAGTT
GTACAAAAGTCGACTGTGGCGAGAGAAACTATATACTAAGTGCTAATTATGATATCAAGCTTGTT
TTATCGTTTGCATTTAAATTCCTTTTTTTATCAGTTAGTTCTACTTCTGCAACTTGTCTTTTTAGTTAT
AGCATCCCCATTTTTCTTAAAAGGATTTCCACTCTGCATTGTAGATGATATAGGTGTGGTTTGCTTC
TGGTTATCTTCTTCATTCTTATCACTTCATGGTTTGAAATGTATTGAGTTTTTGTTTGTTATCATCAC
ATTTTTTTTTTCTTGACAAATGTTAATCGTTAGTTTTTGTTAATGAGA

>Glyma.17G038800 (GmCAMTA7)

AACCCGCGTACTATCGTGGACATGCTTTGGCTTACATCGTAAGAAACAAGTGCCACGTCGGACAT
GTGTGAACGCTACGTGTTGGGCTTTGACTTCAACTCTCTCTTTTCTTTAAGTACCTTTCACGCGCCA
CCGTTGCTCTCCGTCCCTGTCTTGTCCCTCTCTTTTCTTTTCATTATTATTCCTTCATTTCTATTTCCTT
CAACACTCTTTCATTTCCCTCTTACTCTGTTTTGGCCTTTTCCTTCATCGCTTTCATCCAATTCAATT
CAATTCTCTGCACTGGCTTTTTGAAATTTCCATGTGTGAGCGAGAGATTATTGCTATGTTGTAATC
ATGGCTGAGACCACAAAATACATTCCCAACTCACAATTAGGTTAGAGATTCCCCTTTTTTCAATTT
TCGTCGTTATCATCATCATCCTTGTGCCTTTTTTAATTTACATTTTTCTTAGTAGTTTCTGTTGCAAT
CATTTCATTCTCTCTGCATGCATGCATGTGTTACTGTCTGTCGATGGGATCACTTTTATTTCAGTAT
TCCTGGTCAAATGGGATGTCATTTTCTTCTTCCCTTTCGTTTACATGTTTTCAATTGCTTAACTTGGA
TTGGATCACTGATCACAATTGGGAATTTCGTACTTTGATGTGAGATATAGTTAGCCATGTCACACT
TGAATTAAATTCATGCATTGCCTGCGCATTATTGTATACTTATTTAAACTTTGGTACCATTGTTGCC
TTCATTGAATATAAGTATAACTTCATGAATCAAACAGAGTAATGTATTTTGTTTAGTTTGATTTTTT
TTCCCCATGTCTAGTATAATACCCTTTGAATCTTTGAATGTTTCCTGGTACGTAATAGAGCTTGAA
GAGATATTGAATGAAGCAGAACATCGATGGCTTCGACCTGCTGAAATATGTGAGATACTTCGCA
ATCATAAGAAGTTTAAATTAACACCAGATCCACCTGTTATGCCTCCAGGTATTTCCTATTTTTCCC
TGAACTTCATGCCCCTCTTGATTTTCATATTTGCTACAGTCAAGGAAGGGGAGGGAATGCATTTTA
CCTCATTCGGGGCTTGAATAACCGATTCAGTGGGTGACCAATGGGGTAACAAGCTAACACCAGG
GAGATAGAAGGGTGAAGGGAGAAGGGAGGGAGGAGAGGTCACGGTTTCGAATCCCCCAGCTGA
CAATTCTAACAAATCTAACAAACTAATATTTGTCGATGAACAGAAAAAAAAAAAAAAAAAGCT
CACACCACGTGGTCCTTTGATTCCTTTATCAAAGGGCACTTCATTGTTGCTGTCATGATAATCTAA
AACATGAAACAATTGTCATGCACTCATGCTTTATAATTTAGAATTTCCTTGTTTTGTTTTTCATTGA
GCATTTGAGATGGAGAAACTAACTGATAATGGTGATAATAGAACTCAGTTAGCTAAGTTAGCTTC
ACCAACGCACCAAAGGAAAGACCTTGATCGTTATATATGTTTTGCCTGCTACGAGGATGGAGTGT
TAATTTTGACATCAAATTGTTATTCCTTTTTACCAGCTGGATCACTATTCTTGTTTGATCGAAAAGC
ACTTCGTTACTTTCGCAAGGATGGTCACCGGTGGAGGAAGAAAAAAGATGGGAAGACTGTAAGA
GAAGCTCATGAAAAGTTGAAGGTGTGCTGTTCATTTTATTAGTTGTAAGTTTCACCTTTTAGAATT
GTTATTTTAATTGAACCTCTTGCATGTTTCAAATTGTTGAGTTGCAACTTTGGAATAGCCTCATGAA
ATTAGTCACCTTTAGATTCCTATTGTTTAACATACAATACCATGGCAATGCATTAGGAGACACCTC
TGGTTACATAAGTGTGTAGCTTCATGGATGGTCCTGAAATTCATGGCTTGTTTAACTATTTTGTGA
ATGTGATCATTAATATATGAAGCACAAGAAGCTTGATATCTAAGGAGGATGTCCTTCTTTAAGTG
TTCAATTATTAAACCTTAATTTGAATCCATGGATTTATAATTTCATATAATTTACTGTTAATTTAAA
ATTAATGGAACAACATAAAGTTGTCACACCATTCCTTTTTTTCAGTTCATTATATATTCTTTATTGG
GACTATTCTATTGTTGTTTTTTTCCTTAGCTGGACATATGTTAATAGTGGTTGAGGAATCTCCTAGG
ATTATGCTAAAGCTAACTAGTTTTTTTCCTTCCAATTTCATATCCAATTGGTACTTGCCTTTTTTCTT
GACAGGTAATATTTCATTCAAATTCATTTATGATTAATATAAGTAACACAATGGATAATAAGCTA
AGAAATTTTTGTAAACAAGCTTGTTTATGTTGAGATAAAATAGCAAACATTAGAAGTGCTTTGAC
AATGTTTTGGCTTTATTGTATAAAGCTTATATTTTGGCAATTGTATCAAATGAACATATTAAGTAA

TTGGTATTTTAAAGTGTAGGCTGGTAGTGTGGATGTCTTGCATTGTTACTATGCTCATGGTGAGGA
TAATGAATACTTCCAACGGAGAAGTTATTGGATGCTTGACGAGTGAGTTTCTGTCATCCTCTTAAT
TTCAGGTTTTGTAGTTCCTATATTTGATTTTTTTAAGAGGATATTCAGTATGGTGCTATGTTGCTAA
ATGCTAATGTATTTGGTCAATCTGTAAATTTATATTGAGCAATAATAAATCGAGCTTTAAGTTTGT
ATTTTGCGTAAAACACTGAAAATAATGTTTTTCTGATGTAGTTTTTTTCTTTTGGTTCTTAAGGGGC
CAATATTAATAAATCCAAGATTGGACCTTGCTAAATAGCAATCTAAACAGTCCAGTTGCAGGGTG
GATTTATTTTTATAAAAAAAATAAATTAAAATACTGATATATAAAGATTTCTAAATTTCAATAATT
AAACAAAAAAAATCCATATTTCATTGATTGATGATGAAAATCCAGCAATATTATAAATATTGAA
AACCGATGAATAAGATTGCTGTCTGTGGTGAGCTCTTATTTTAATGGACTTATTTTTTGAAGGTTTG
GGTCTGCATGAAATTACAGACTGTTCCACTAGTTCTGGCCCTTTTTAATGTATTAGCATTGATAAG
AAAAAAAGTGACAACTTGAACATCGTGTTGGTTTGCAGGCAGTTAGAGCACATTGTCCTTGTGCA
TTATCGTGAAATTAAAGAGGTATGCATGTGTTCTTCTCTGTTTTTTGAATTTTTTTTTAATATCATAT
AAAGCTATCTAGATAAGATGCTGCTATATTGTAACCAGAATCCAGTTTATTCTGATTGGTGTTAAG
CTCATCTATATTTTTTTTATGTTATCATTCTTCCAAGCTTGTTTATGATGGTGTGCCACTTATGTGGG
TTGTGACCAATCAGAAAGACTGACTAATGATTGACCTGTCAATGGATTTTCTGATCTTATTGTGAT
TATTCAAATTTGCAAGTTTTGTGCATTTAAGTGTTGTTGTATTATGTATGCTTTACAGGGCTGCAAG
TCTGGCATCTCACATTTGCCTGTAGTTCCAGTAACCCTGGTTGGTAGTTCTCAAAATACTTCAGTG
CTTTCCTCTACCAAAATAAATTCACCAATATCTCTAGTTCAGACATCTTTCACATCAAGTGCAAAT
AAAGTTTATCAGAATGGACGAGCCTCAGAGCATGAGGATGTCAATTCAAAGAATGGTCCTCAAG
CCTCCTCTCATGCTCAGCCTATCAGCAATTATGTACTTCACAGTGCACCTTGGCTTACACATGAAG
CTGCAGGTAAGTTGTATGAGACTGTAGACGTAGGATTCATTCAAATTATGATGAATGATTTGAAA
TTAGGAATAAGCTTGTTATGTTGGTTCTTGTGTGTATTAATTTTCACTGCAAGTAGATCCCTAGTGC
CATTATTGTTTTCTCTTATGACAAAGCATCAATAATGCAGATTTTAAAATTTATTGTTTTATATGTT
ACTGATCTTTCATAATGCTCTTTTACAGAATTACTCATTATTATATTATCGGGATTATTTTCATTTTG
ATAGTTCAAAGTTAATATGCCTTCACTTTATCTTTTTAAAGTAAATTTCAACTATAAAAATGTTGA
TTTATTTTAATTTATCTTGGGATGCAACCATTGTTTTATTTTGTTTCGTTATTAGTCAAATGATTTTT
GTGGCAAAGATAATGCCTATTGTTTTGGTCCTCATTTACTCTCACACCTCTAACTATTTTATTTGGT
CCATGGAGAATAGCAGTTTTCTTTGAATTTTTAACATGTGATTTTTTTTAATGATTTAGGGTTTTCT
GAGTTGTTAAGGAACCCCTTGATTTCATCATGGCCATCTTCTTTCCCTAGCTACTCTCCTGGTACTG
GCTTGTCTCCCTGGACCTCAATTCAGAACTCTAGCAGAAACACAATTAATATGCATGATGGAAAG
CATCATGTTGAAGCCTCTGAAGCAGACTTAACTGTTCGTAAGCTAAGCAATGCTGGGTTAGATTC
TGTTCACCGAATGCAAGATGGTGTAATTTTCAGAGATAGGCTCATCACTGACATGTGCGTCCAGC
CAGTTATAGATTTGCCTACTGTTAATCAGGTGTAGTAATTGTGATTATCAATAATTTTTAAACTTTG
ACTGCAACATCTCTACTTTCCTTTATCATTCGTCATTATTTTATAGCCTCAAAAGTTTGGGTAGATT
TGTGCTTCTGTGTCTGAAACAGAAAACTTAAACAGTCCAAGTGATCATTAGCTTTGTCAATGCATT
TGATATGCAAGTTAACAACATAATGTATTTTATGTTCTTTAGGTCTATGTTGTGCCTGACTTTCATG
CATAATAATATGACTAGCTGAAATCCTGTAGGCTTCTGGATGTTACTAATGCAATCTCGTTCCTGT
GTGTTTTTTATATAGTTTACTAGCATGCTCATTCACCAATGTGACATTTTTTTTAATTATATTGTGGC
AGGTGAAAAACGAGCATGGTCTGGATTCATTTCATGCTCAAGTCCATGATCATAATGATCATCCA
GTTGTTGCAACTACTAAAATCCTAGTTGAACAAAAACTTCAAGATGGTGGTTTATACAATGATGA
ATCAGAACAAGTTGAATATGGAGAAATGAAGAAACTTGATAGTTTTGGAAGGTGGATGGATAAG
GAAATTGGTGGAGACTGTGATAATTCCTTGATGGCTTCAGATTCTGGTAATTATTGGAGCACACTT
GATGCTCATAGTGAGGATAAGGAGGTATCTAGTTTACGCCACATGCAGTTGGATGTGGATTCATT
AGGTCCTTCTCTTTCCCAAGAACAACTATTTAGTATCCATGACTTTTCTCCTGATTGGGCATATACT
GGAGTTAGAACAAAGGTGTGTGCCTTTTCATGCACATGCTTGAAATTTTATGCATTTGTATGTTTA
TGAATTCACAATATAAAATCTGGTTTTGATTTATTTCTTCCTTTTGTGAAAAGGTTTTAATAGTTGG
CACATTTCTGGGGAGTAAAAAGCCCTCTAGTGAGACCAAATGGGGATGTATGTTTGGTGAAATTG
AGGTTTCAGCTGAAGTTTTGGCGGATAATGTCATAAGGTGTCAGACTCCTTTGCATTCACCTGGAC

GTGTACCATTCTATATTACCTGCAGTAATAGGTTAGCCTGCAGTGAGGTCAGGGAATTTGAATTTG
ATGAAAATCCAACCAAATTTTTAGGTCCTGAGGGTATTAAAATCTCACCAGAAGAAGAGGTACG
ACTTCAAATGCGACTTCTTAAACTAGTAGACTTGGGACCTGACAATAAGTGGTTGAAGTGCTCCG
TTTCAGAATGCGAAAAATGTAAACTCAAGGGAACAATGTATTCCGTGAGAGATGATAGTGGAGT
ATTTGAAGAAACTTTCCAGATTGATGGAATTGGTCATATAAACCATAGAGATATATTATTTCAGA
GATTAGTGAGAGACAAGCTTTATGAGTGGTTGATATATAAGGTTCATGAAGGAGGAAAAGGACC
ACATGTGTTGGATGATGAAGGACAAGGAGTAATACATTTGGCAGCGGCACTTGGTTATGTGTGGG
CTATGGCCCCATTAGTTGCAGCTGGTATCAGTCCTAACTTCAGAGACTCTCGTGGAAGAACAGGA
CTTCACTGGGCATCATATTTTGGGAGGTCAGAAACTCTTCATGTTTTTCTGTGCTTGTATCTGGTAT
TATCAGGGATTTATCTTCAACAAAAATGCATATTGTAATTCCTGTATCTTCTGTCTTTATTTGGTTG
GTGGATAAATAAAATTATACTCTCAGTGTATTCTATGAATTTTTCTTTTCAAAACTTCAACTTTTTT
TTCATCTATGCCGGAAATTTGCATGCCCAGTTTCATGTTGTTGGGGATGATTCTCATTGCATATTGT
CTTTGAGTCCTTAGACTTGAGTTTTATGTGTTCCCTCTCTCTAATTATTGACTTGCATTTTATGGGCT
ATATATAGTAATTTTATATAATGAATGTGTATCTATTCTCACTCTGTCTTGTGTTCCTTCTTTCCAGA
GAAGAAACTGTTATTGTGCTAGTTCAGTTAGGTGCAACCCCAGGTGCTGTTGAGGATCCGACATC
AGCATTTCCTCGAGGACAAACGGCAGCTGATTTAGGTTCAAGTAGAGGACATAAAGGCATTGCT
GGGTATTTGGCAGAGGCAGATCTGACAAATCAATTATCTGTATTGACTGTCAAGGAAAATGAAA
CTGGCAACATTGCCACAACAATAGCAGCTAACAGTGCTCTTCAGTCTGTTGAAGATGACTCATCT
AGTATGACAATGGATGAGCAACATTATCTCAAAGAGTCACTTGCTGTGTTTCAAAAATCAGCTCA
TGCAGCTGCTTCAATCCTAGCAGCCTTTAGAGCAAGGTCATTCTGTCAGAGACAATTAGCCCAAA
GTAGCAGTGATATTTCTGAAGTACTTGACGTAGTTGCTGATTCTTTGAGCAAGGTACAGAACAAG
GGTCACTTTGAGGATTATTTACATTTTGCAGCTTTAAAGATTCAAAAGAGATATCGGGGATGGAA
AGGAAGAAAGGATTTTTTGAAGATACGTGACCGCATTGTAAAAATCCAGGTACATGTGTGCTTAC
AGTGTATTCCACTACCATCAAATTATGATCATGCCAGACTTCTAGAAGATTTTCATGACAAGTTTA
ACCATCAGCTTCTGTTCTGTTTCACCCTAAAATAATTTTGTTTTCATTCTTTTGGACTTCAATCTATA
AAAATTTTATATGGTGACTGGAGTTTTTCTTAATATGAGCTTATTGTGATTTCATTTGGCATTTACC
ATTTGAGTAAACCTCCATTCAAGACCCTGAGATTTTCTTAATATGTACTGACGGTGTAAAACATTC
AATCACAAACCATTATATATGTTAAGTTTGTTGATTTTTTACAATAAATATCTTAAAAGTAGTTTT
ACATTACCATTAAACTCTTCATTTTATATCTGCAATGGAGACAATATTACATCTTCATTTAATTGA
ATTGTTCTATATAGGCTCATATTAGAGGACACCAAGTTCGTAAGCAGTACAAAAAAGTTGTGTGG
TCTGTTAGCATTGTGGAAAAAGCAATCCTGCGCTGGAGACGGAAAGGGGCTGGTCTGCGAGGAT
TCCGGGTTGGGCAGCCAGTTGGTGTTGTGGTTAAAGATGCCGAGAAAAGTGACGAGTATGAATTT
CTTAGCATTGGGAGGAGACAAAAATCAGATGATGTAAAGAAAGCTCTAGATAGAGTCAAGTCCA
TGGTTCGTAACCCAGAGGCAAGGGATCAGTATATGAGGCTCATCATGAAATATGAAAAGTTTAA
GGTACTCATTATTTATTTCCTTTTGTTGGGCCATGTTTTTTATGCCATTGTGGTGCACAGATGGCTTT
CAAGTTTTGGTTGATACAGTTTTGGTGAATTGATTAAAAAAGAAGATATAATCAGTTCTTGTTTAT
TGAAAACAAATCTGATTTGCTAATTTTATTTTGCTGCTTGTTTTGGCATACTGTTGAACAGATTGAT
GATGGAGGAAGCAGTCAATCACAGCATGTCGGTTAGATTCTGAAGGAAGGAAAGAGGAATACC
GATGGAGCTTATTGTTTATACTAAAATCTTACGATATACTTTCTATGTAAATACCATCTAACATAG
TTTCTAGTTTATATGAGCGTTCAGATACTTTGAAAGGTAGAAGAAAATTCAGAAAAAAAAGAAA
AGGAAGGGGATGCCTTTTTATTCCCCTTATATTATATCAGTTGAATAGAAGTCAGTTAAGTTTTTTT
TTTTTTTTTTTTTGTGAAATAGAAATCAGTTAGTTGATATAATGTAATAATATTGTATAGACTTAAA
TAAAATGTGGATTTTCGCAGTATATTACTTTTATTTAT

>Glyma.15G143400 (GmCAMTA8)

TTTTATTAAAAAAAAGAAAATAATTCGAAAATAGTAAGTGAAGAAAGAAAAAGCAACAGAGGA
GACCTGAGTGACATTATTCAGTTCCGACTTACCGTTTCTCTTTCTTCCAATCTCCGGAATCAGGTA

AAATTTCTACCTTTTTCTCTCTTCGCATTTGACTTTTGACTTGTTTTTTTTCTTTTTTTGTTCTCTCTTC
AGTTCACAACTAGTTATCTGATTCTGTATATTATCTGACTTCTTGCCAAGGTTGTCGGTGCTGCAAT
TGTTAGTTTTGTGCTAAGAGAAGAAGCTGTTGGGAAAGTTTCTTTTGTTGTGATGATGTCACACAA
CTTAACGGGGCAGCTTGTGAGTGCAGAGATACATGGCTTTCACACGTTGGAGGGTACTTGCCTTC
TTCCTCTCTTACCTCTCTATTTGGTTTGTGCTCATATTTGGTTGAACCTCAATTTTAACTAAGTTTCA
ATTTTGAGAAGTTCCCTCTTGTTTTTTTTTTTTTTTTTTTGGGAGGGGGGTGATCAAGTTTGAATTGT
GTGTGTAAGTTGTTGTTTGGTCTCATGTGTAGCTGGTTTTTGGCTCCTAGAGATGATTGTGTGTGAT
TTTCAATGCAAGGTTGGAGTGAAGAAAGAAAGTGTGGTTGGTTTTGGTTGTGGATTGTTTCTCCTT
GATCAAGTGTAAACTTCTCTTTGTGTTTTCTTGGAGAAAATAAGGGAATTTGGGGTTTTAAAGATT
GTTCTGGTATTGGTTGTTGTTTAAACTTGATTAAGTTCAAACTGTTTTGGGAGAAGCAACTAGAAA
TCATTCTTTGAGAACTTAATTAAGGTTAAGGGTATTGGTACTGAGGTCAAAAGCAGGGTACCATG
ACAACTCCCCAATTTTATTGTGTTGTTTCCTTATATTAGATAGACTGTGTGTGGATAAAATTTCTCG
ATAAATGCTTATATAGGAGAAGCAAATAAAACACAAAATGAATCAAGCTTCTTCCATAAGTTAA
AATCTACTTAAGCATCTTAGTTAAAACCTACTAGTAAAATCATTTCCTGTATTATGCATAAGATTT
AGTAAATGTTACTTGTTAGTATCTTAGACACTTGTGATTGCAAAATATATTGATTTTGTGAACTCT
GCAATATGTTCAAACAAAATGTCGCAGCATGTAGATAGAACTTAGAAGGTTAGTGACTTTACAA
GGATTGATCCCAAGGCTCAGTAAACCAGCTGAGATGTCCAACTTACATATAAATATCGGCTTTTG
TTAGGATGTGAAATAGTTCAAGGAAGCAAAAGATTAAAATCTCAAGATGTGAAAGTGTGGCTTT
AACTTTTGGTTCAATATGGAATAGAAAATTGTACTTTTCAATATGGGGTTTATATTTGCTATGTATT
TCTTTGCTATTTGCTTCCAACTGCAGGTATTATTACTACTACCAATTATGTGGTCAATGGTCAATGC
ATATTAATCATAACTGCTGATTTGTTTTTCTAAAGTAATTTCTTATGTGGCCCAGATTTGGATGTTA
GTAATACTATGGAGGAAGCAAAAAGCAGATGGCTTCGTCCCAATGAGATCCATGCCATACTCTG
TAATCACAAATATTTCAAGATCAATGTCAAGCCAGTGAATTTGCCCAAAAGTAATCACATGCTGC
TATTAACAGCAACTTGATGTTGTAAATTAGATATGCATTGTCAAATAGATCATTTCTCATCAACTT
TGTTCTCATCTCTTTCAACTTCTATTTCTTAATTTCCATGAAACATTTTGATTTTGTTGCTTCTGAGC
ATTTCATGTTTTGCAGACGGTATTATTATTGTAAACATGTAGATCCTTCAGTTCTTGTTGAAGCATG
TCTTAACAAAAAGTAAAATACATAAAAAGAAGAAAAAGTTTTATACACAAATTTATGCATTGTT
CAAGTTTATATGCTATAAAACCTCTATGATTGATTTCTGGCATTATTCCTTCGTAGATCTATTAAAT
GGAATTTTAATTAAAGTAATATTTTTGCATCTAACTAACAAATTGTTTGAATTATTGAGAATTATA
TGATGTCCAACAGGATTCCAATCATCAGTGATAATGTTATATTATAATTTTGTTTGTAAAACAGGT
GGTACTATTGTACTATTTGACCGCAAGATGCTTAGGAACTTCAGAAAAGATGGGCACAATTGGA
AAAAGAAAACCGATGGGAAAACTGTCAAAGAAGCCCATGAACACTTAAAAGTGAGGATTTTAA
TTTTGTGGGCGTTTATTTGAAGTTGTGCTAGCAAGTACCCCATTGAATGTAGTTTATTGAAGATGT
ATCTAGGGAGTTTTAATGGTTTGTCCTATATTGAATTTGGTCTTCTAATGGTTTTGCCTACTATAGT
AAAATTGGTCTTTTTTAATAGTCTCTGATGGTTTGTTGTGCCTTCCCTGATCCTGTTTAATTGAACTT
TCAAAATAGTTTTACTGAATCTTTGGTTGTTTAGAGTGGCCAATGGCATCAGCTGATCAATGTAGA
GTGTTTTTGGCGAGATGAAGGGAATAAAATATTGTGACATGAATATGATGGAGTATATCAAGTGG
CCTCATGAGTATTTGGGTTAAATATTGAAATAAGCCTCTAGAATTGGGTCAGTGGTGAGATGTTTG
GATTGTTTTCATGTGATTATAGGTTTGAATGACCTGCCACCTTGATACTAAAATAGGTTAAATATT
AAAACACTGGGAGCTTTCTAAAGCTCAGTTATGCATTTTACTAGTTTTATTACACCATCTTTAATT
CAATGTGCTTTCTTCAATGAGCTGCGAGTGGTAATTTGTTGTTGGACTTCGGAGTTTTGACATTCTG
TCGATTTAGACATGATTTATGTTAGATTTAGGAGTATTAATTCAGTAATTGGGTACTGCTAGGTTT
GTATCACACATTCTCTATGCTTTGTACATCTATAGTTGTAAAATATTTAGTTATATTTTATCATTCTT
TCTTGGTGACATTTGAGGAATAAATTATGTTTTCTCTTCAATCTTGTCACCTTATTACACTATATTTT
CTATTATCAACTTCTCTTCCTACAGTTTTTGTTATGTGATTCCAATGTTTGATTCCTACTATTATTTTT
TCCATCATCAATCAGGTTGGCAATGAAGAAAGGATCCACGTATATTATGCACATGGCCAAGATA
ACCCAACCTTTGTTCGTAGATGTTATTGGTTGCTTGATAAGTATGTTTTGCTGGACTTTAGGTTCCC
AATTCCAATATATCATGCATGTCTGTATTTGTGAACTGAGTTAGTAACAAGGGTTATACATTTCCT

GAATTTTTGTTAAACAGAGAATAAAAATAAGTTTGCTAATAGGAACTTGACTTACTAATGTTATG
ATTTTATTCAGGAATCTGGAGCACATAGTCCTTGTTCATTATCGTGACACACAAGAGGTTGTGTTA
ATCCCTTTCTTTCTTCCTTATTCCTCTACAACATTTAACTTCATATCCAAATGATGACTAAGATTTC
CTATTTGCATGTTGCTTTGCATGATAGAAAAACCTTATATTTTTAGTTTATTGTAGAGATTATGGTG
AGTTTGTATTGTTGAAATTGTGATAATATTACCGAATATCTTGTAGATCTTGTAATATTCTTTCCCT
GGTTATGGGAAACCTCTTTGAGCCTTCGTACAAAATTTCTATATATATGATAAGGCTGGGAGACT
CTCTCCTAAGGAAGCTTTTCAGTCAAAAAGTTGTTCCTTGTTTCTGTTTCAAGTTGGTAGCAGAGC
GTACATCAAGTTGGCCTGATCATTTGAGTGTTTCTGGATGCGCAGAAATTGTCTATAAGAATAAT
GTCTCACACCAACCATGTTTTTAAAATGGTCTGGTTGGAAATGAAATTTATTCTCTGCATGAACTT
CGCTGGTACTAATGCAAGGCATCTCAATAAAGCTCCTCTAAAGGGAATTTGTGTCTAAAGTGAGC
AAGGGGTGTATCCACCCTTAAATTTTAGTGAGTTAAAATCACACTGTAATTTAAATTAAATCAAA
GGGTGGATACACCCTTACTTAGAGGAGCCTTTCCATGACTCCCAGTGTTGGATATGTGTCTGATA
GAACTGGACCAGAAAAGGTGAAATTGTTTTATTCGAATTAGAATGCAGTAATTACACACTCTGAT
GGATTTGAGGTCTCATCAGCCTCCCATTCTCCTATCACTTTCTGTTCAACCCCTAACTAACCCCTCC
CACAGCTATTTATAGCCTCTTCTCTAACCACAATTGGTTATAACTAACTAACTAACAGTTATCTTA
GCTGTCCTACAACTGACAGCTCTCTAACTCTTCCAAGGGTATTTTCTAACATACACCTTTCCCATC
TATTTCCCTCTCTATCAGTGTCTTTTATGATTTACCATTTGAACTGGTTATTGAATAAAATTTGGCT
CTCTAACCTTTGTTAGGTGGTGGTTTATGCTACAGTTACAATTGCAAGGTTCTCCTGCTACACCCG
TAAATTCAAATTCAAGTTCTGCCTCTGATCCAGCTGCATCTTGGATTCCATCAGAAGACTTGGATT
CTGGGGTGAACAGTGCTTATGCTGTTGGTGAGAAATAACTTTTAGATGATTTCAGTCTATGTTCTT
ACAAGTTATATTCTTGAGGCTATCAATATGAAAGGCTTCTTACATTTCTTTTTAATATTGGCATCTT
ATATTTAGAAGATGGTAAGTGCTTGTTAGGGGAAAAAAAGCAGGATAATTAGTTAAGATAATTA
AATAAAAGAACATGTGCAACGTGCATGTCTTTTTATACATTGCAAATTTTTATTCCTCCGACTCCC
ACCATTTTTGTCTTAAGCCCATCTTTCCAAAGGAAACCATAAATAAAAGCCTTTTTATTAGTTGGA
TATGATCTAATGATCCTATATATATGGTTTCTATTGTTGCTGTCATACATACTGTTGCTATCTTTCCT
TGACTAGGTATATATATCCTCATCCCTTTTTTCTTTTTTTGGAGATAAACAGAGCTTAATGACAATT
TAACTGCCAAAAGTCATGAGCAGAGGCTTCATGAGATTAATACACTTGAATGGGATGACCTAGT
GGTTCCAAATGTCAACACTTCTACTACATCTAATGGAGGTAATTGTTTGCTCTTTAACAGGACCCT
TTGCTTGTTGGGAAAGAACCTTAATATTTTATATGTTAAATTTACTTTTGCCAGGCAACGTCCCAT
ATTCATTTCAAGAAAATCAAAGTTTACTGAGTGGAAGATTTGGCAATGTAAGATTATATTGTCTTT
CTTTCATTTGTGTTCTTTATTAAGCTGTGTTGTAATTTTTCATTTATGTTTTGTGGAAGGTTTTAAGG
GACCATTAGTCCTTTGAATTTCTTTTAGATAGAAAAATAAATTTAATAGGAGAATAAATAAGTAA
AGCACAGCTATGGGGAGGGTTAGATATTCTTTGAAATAAAAAGAGAGGGAAAAGAAAGATTAA
AGAATGTGGCCTACCAATCTTCGTGTACGGGAAATGAAAACTCCCTTGAAACAGCCTTGAGAAG
CATGCCAAAGAAAGGCCAATAACACCAGCATATTCAAAGCACATTTAGGGGTAAAATGCAGGC
ATTCTTTTTCAATCAGACATACCAAAGAGCAAGCTGCACACAGCCAAAAAAAGTTTCATCCCCTT
GTTGTTAGTCCTGTATGAGTTTATTGTTATTTTGGCTAATCCTTTTTAAAGTGTATGTAATACTGTCA
AAAGAGAGTTTATATCATTTATTTATAAAAGTAGATGCTCAAGCATTCAGGATTAAGCATTGCAA
TTGCACTTGTTTTGTCTCGCTGTTGAAATTATTGTTCTTCTGTTGATTATAGGAAGTTATACACTGTT
TTTTCTTTTCTTTTATTTTTGGCACTTATTACTTGTTCTCATGTGTTCTCTTGGGCTAAGACTTAACTC
AGTTGTACCCATTAACTAAGGTCCTAGGAACTCGGTATATAATATTTCTTTCACACTTATATAAGT
AAAATGTAAATATATTTTATATATCACTTAAATTGGTAGAAAAATCGTTCTTATCAATAGACAAA
ACATAAAAGGTTATTGTTTATATAGATAAAATTTTGTATCTCTTAAATATATTCTCATGTTCTTCTG
CTTCTTTCTGTCTTTCTTCTCGTGATACTTGCTTTGTGATAGCTGCTGTTGTAACCTATATAGGGCTT
TTGTTACCAGGTATCCAGTAATCCTTCAGCAGAAATTCCTTCTTTTGGTAATTTAACTCAGCCAGT
TTCTGGGAGCAATAGTGCCCCTTACAGTTTCCCAGATAGTGCTATTCTTCTGAAAAATAGTCCTAT
ATCATCGGGAGGCGTTGACACCTTGGGCACTTTGGTCAACGAAGGCTTGCAAAGTCAGGACAGC
TTTGGAACGTGGATGAACATCATATCGGATACACCATGTTCCATAGATGAATCAGCTCTTAAAGC

ATCAATTTCTTCTGTTCATGTACCATATTCATCACTGGTGGCAGATAATCTGCAATCTTCTCTGCCA
GAACAAGTATTTAATCTCACTGAGGTGTCCCCTACATGGGCATCTTCCACTGAGAAAACAAAGGT
ATCCTTTTGTTTCTAATATGTCTAATTGCTAGCAATTATTTTCTGTTATAATTGTATCCTTTAGCACC
AAATGTAATATTTAGTTTCTTAGTTAAATTCTCTGCGTTTTCATTCAGTCTTGGACAATATAGTATG
GCTATGGAATTTCAAAGCTTCGAACTATAAAACCTACAACTCCACGTGGAGCACACATTGTCAAT
TTTTGCAATGTAAAAAAAAATGCATTTTCAGTAGGCATCATTAGCACTCTGGCTACAAGCGCGATC
AATTCATATGTTTGCTACCATTTTTTCTTTTTGGCAATTATAAGTGAAAATTCAATGTCTCTTTACTT
CTTGCAGGTCCTGGTAACTGGATATTTTCATAATAACTATGAAAACCTTGCAAAGTCCAACCTAC
TGTGTGTCTGTGGTGATGTAAGTGTTCCTGTAGAAATTGTTCAGGTTGGAGTTTACCGGTGCTGCG
TACCACCACACTCGCCTGGATTGGTCAATCTCTATTTGAGCTTTGATGGCCATAAGCCCATCAGCC
AAGTTGTTAATTTTGAGTATCGTACTCCTATTTTGCATGAGCCAACTGCTTCAATGGAAGAGAAAT
ACAACTGGAATGAGTTTCGACTTCAGATGAGGCTTGCTCATTTGCTCTTTGCTTCAGACACGAGCC
TTAACATTTTCTCAAGTAAAGTATCACCGAATGCACTGAAAGAGGCTAGAAGATTTTCCTTCAAA
ACATCATATATCTCCAAAAGTTGGCAATACTTGATGAAGTCAATTGATGACAATACAATTCCATT
TTCAAAAGTAAAAGATTCCTTGTTTGAAACTGCATTGAAAAACAAATTAAAGGAATGGCTTTTGG
AACGAATTATCTTAGGCAGAAAGAGTACTGAATATGATGCGCAAGGGCAAGGTGTAATTCATTT
GTGTGCAATGCTAGGATATAGTTGGGCAATCTCCTTATTTTCTTGGTCGGGCTTGTCGCTGGATTTC
CGTGACAAATTTGGATGGACAGCTCTTCATTGGGCAGCATCTTATGGAATGTACGATACATGGAT
ACTCCACTTGCCTGAACAATTAGCCTAGCTGCAACCACATTGACCTGAAAATCATTTTGTGACTA
GTTATAAAAAATAAAAATAAAAAATCATTTTCTAACTTGATTTCACTGTTCAAGTTCTTTCCAGTT
TCCTTTCACTAAAGATAAACAGGCAGAGCATGATATATATGATCTTACCATCAAATTTGGCAATA
TATGATCTTTTCAGTTTCCATATGATACTTTCTTGTGTGCATTTGATTTATTTTTAATCCAGCAACGA
GCATGAACAAACTTTTCATTGTTTATTTGATTTAAAATTTAAAATTTGTGTCTGCAGGGAGAAAAT
GGTTGCAACTCTGTTGTCTTGTGGGGCAAGGCCAAACTTGGTCACAGATCCAACTCCACAATATC
CTGGTGGATGTACTGCTGCTGATCTTGCCTATATGAAGGGATGTGATGGCTTAGCTGCTTTCCTTT
CTGAGAAGTCTCTGGTTGAGCAGTTCAATGAAATGAGTTTGGCTGGAAATATCAGTGGCTCACTG
GAAACTAGCTCAACTGATCCAGTAAATGCTGAGAACCTTACTGAGGACCAACTGTACGTTAAGG
AGACTTTGGCAGCTTACCGTATATCTGCCGAGGCTGCAGCACGGATTCAAGCTGCATTTAGGGAG
CATTCATTTAAATTACGATATAAGGCAGTTGAAATTATCAGCCCAGAGGAAGAAGCACGTCAAA
TAGTAGCAGCAATGAGGATTCAACATGCCTTTCGCAACTATGAGTCAAAAAAAAAGATGACAGC
CGCCGCTCGGATTCAGCATAGATTTCGTACATGGAAGTACCGTAGAGAATTTCTCAACATGCGGC
ATCAAGCTATCAAAATTCAAGTAAGACCTACTCATCCTTTGTTCTTGTTGACTATCCTGAGTAAAT
ATGTCACAATGAAAAATGATGTTATTTGTTCGAGGCAATTTTTGGATGGATTGACTGTGGATTCAT
GTCAATTAGCACATATAATGTGTCCTGTGCCACAAGTTATGAGAACATTCATGCTCATCCAAGCA
CTTTTAGTGACCACATATTTGAACTTAACAAGTCAACTATTTGTTTGTTGACTATAATTTTTTACCA
GTTTGTAAGAAGCTAGCAAATTGACCAAAATCATGGCAAAGAAAGGTTAGCCTTAAATCATAAT
CTGAAATAGGGCTTATACATTTTAGAGAATGTTGGTGTAGTATAATACCACTTGAATTTTGAGTCT
AGGAGTGGACTTTCTTTCCTGTCTACTCATAGCTTGCTTTCTAAAGTTTAATTGAGACCAACTGCTT
GAGTGGATAACACAAGTTACACCCGGGAAAAGATTAATCGTCTGTGTTTTCAATGCAGGCTGCTT
TCAGGGGCTTTCAAGCACGGAAGCAATATCGGAAAATCATATGGTCTGTTGGGGTGCTTGAGAA
AGTGATCCTGCGTTGGCGTTTAAAGAGAAAAGGCTTTCGTGGTCTCCAGGTTAATCCTGCCAGGG
AGGAGACGCAGGAAAGTGATTCAATTGCAGAGGAGGATTTCTTTCGCACCGGCCGGAAACAAGC
TGAAGAACGCATTGAGAGGTCTGTCATACGTGTGCAGGCCATGTTCCGTTCTAAGAAAGCACAA
GAAGAATATAGAAGGATGAAGCTGACACATAATCAAGCAAAGGTGAGTAACACATGCGAATGT
AATGTCTCATGTTTTTATTTTGTTTCATTAGCTAAAATATTTTTTTAGTCTCTCAAATTTGAGATATT
TTTTATTTTGGTCCTTCAATTAAAAAACTGCTCTTTTTAGTCCCTCAAATTCATAAAATGAGCAAA
ACTAAACTTACCCAAATTTGAGGGACTAAAAACATGTTTAAACTTATTTCATTTCACATGTTTGTT
TACTTTTTTGAACTGCTTTATGCAGCTGGAACTGGAATTAGAAGAATTCCTTGATTCTGAAGTTGA

CATGTTACCCAAGACATAGCTTGGTGTCTTTTCCAGAACAAGGTTTCCCCATATTTGGGATGTAGC
CAACTCCTGTAACAAACTAGTGTGTAGTGATAGCTTGGTGTCTTCTTTTTGTATAGAAAGTAGATC
CTGTAAATTCCCTAGTGTAGATTTGAACCCATGACCCTTTCATATTGCTTTAATGTCTTGAATGGAT
CGTCCGTGTGTAATTAAGCTCCCTATGAGGGATGTTTCTGATGCAAGATCAAGTTTGTAACTTAAA
TAACAAGCCTTCTTAGATTTATTTAGTTATTTCATTTAGTATAAAGTTTTGGATTTGTCTTTGTCTTT
GGGACTTTAGGAAGATCAATTGCAACAAT

>Glyma.09G038300 (GmCAMTA9)

TAAAATCCATCATATCTGAGAGAAGCCAATGGCAGAACTTTAGAAACCGGGAATATTTTTCATTA
AAAAAAAGAAAATAATTAGAAAATAGAAAGTGAAGAAAGAAAAAGGAACAGAGTAGCAGAGT
GTCATTTTGCCGTTTCTCTTTCTTCCGATTCCAACAGACCAATCAGGTAAAATTCCTACCTTTTTTT
CTCTCCGCGTATTTGATTGTTGACTTTTTTTTTTTTTTTTGTTCTCTCTTCACTTCACAACTTGTTTTCT
GATTCTATATATTATGCTCTTCACTTATTGTTCTCTTTTGTTTGATCATGTCTTTGCTGACTTCTTGTT
AAGGTTGTCGGTGATGCAATTGTTGTTGGTTTTGTGCTAAGAGAAGAAGCTGTTGGGAAGGTTTCT
TTCGTCTTTTGATGATGATGTCACACAACTTAACGGGGCAGCTTGTGGGTGCAGAGATACATGGC
TTTCACACGTTGGATGGTACTTGCATTCTTTCTCTCTTACCACTCTATTTGCTTCAACCTCAATTTTC
ATCAAGTTTCAATTTTGAGAAGTTCCCTCTTTTTTTTTTTTTTTGGTGGGGGGTGGGGGGTGTGATG
AAGTTTGAATTTTGTTTGTAAGTTGTTTTTTTGTCTCATTTATAGCTAGTTTTTGGCTCCTAGAGATG
GTTGTGTGTGAGTTTCAATGCAACCTTGGAGTGAAGAAAGAAAGTGTGGTTGGTTTGGCTGTGGA
TTGCTTCTCCTTGATCAAGTGTGAACTTCTCTTTGTGTTTTCTTGGAGAAAAGGAGGGAATTTGGG
GTTTTGGAGATTGTTCTAGTATTGGCTGTTGTTTAAACTTGATTAAGTTCAAACACTTTTTGGGAGA
ACCAACTAGAAATCATTCTTTGTGAACTTTTAAATTATGGGTATTGGTACTAAGGTTAAAAAGCT
GGGTATCATGACAACTGCCCAATTTTATTGTGTTGTTTCTGTATATTAGAGACTGTGTTTGGATAA
AATTTCTCAATAACACTTATATAGGAGAAGCAAATAAGGGACAAAATGAATCAAACTTGTTCGT
AAGTTAAAATTAACTTATGCATCTCAGTTTTTGAAGTAGTTAGATGAGAAAGGTTTTATAAACGT
AGGAGTGCGTAAGTGGATTTTAACTTACGTGAGAAATTTAATTCATTTTTTTCTTCTCCTATAAGC
ATTTATTGAGAGGTTTATCCAAATGAGATCAAAGCACTTTTTTTTTTGTTTGTCAGCAGACTTAAC
AATGTTCATTTACTTTTACCATATGTTTGAAATACAGTACTGAGATCAACTAAACTGAAATTTTCT
GAAGTATTGTTAATAGTTAATTCTATGTATTGTGTTTGCTATGACTATGAGTAAAATAGTTTCCTGT
ATTATGTATAAGATTTATTAATTGTTACTTGTTAGTATCTTAGACTGTTGTGACTGCAGAATATATT
AATTTTGTGAATTCTGCAACATGTTCAAACAAAACTTTGCAACATATAGTTAGAATTTAGAAAGT
TTGTGACTTTACAACGATTTGTCCCAAGACTCAGTAAACCAGGCGAGATGTCCAACTTACGTATC
AATATCAACTTTTGTTAGGATGTGAAATAGTTAAAGGAAGCAAAAGATTAAAATCTCAAGATGT
GAAAGTGCGGCTTTAACTTTTGGTTCAATATGGTATAGAAAATTGTACTTTTCAATGAGGGGTTTA
TATTTGCTATGTGTTTCTTTGCTATTTGCTTCCAACTGCAGATATTATCACTACTACCAATTGTGTG
GTCAATGGTAAATGCATATTAATCATCACTGTTCATTTGTTTTTCTAAAGTAATTTCTTATGTTGCC
CAGATTTGGATGTTAGTAATACTATGGAGGAAGCAAAAAGCAGATGGCTTCGTCCCAATGAGAT
TCATGCCATACTCTGTAATCACAAATATTTCAAGATCAATGCCAAGCCAGTAAATTTGCCCAAAA
GTAATCACACACTGCTATTAGCAGCAACTTGATGTTTGTGAATTAGATAGATATGCATTGTCATAT
AGATCGTTTCTCATCAATTTTGTTCTTGTCTCTTTCAACTTTTATTTCTTAATTTTCATGAAACATTTT
GAGTTCGTTTCTCCTGAGCATTTCATGTTTTGCGGATTGTGTTATAAAGATTATTGTAAACATGTAG
ATCCTGAAGTTCTTGTTGAAGCATGTCTTAACAAAAAGTAAAATACATAAAAGAAGAAAAAGTT
TTATACACAAATTTATGCATTGTTCAAGTGTATATGTTATGAATATAAAGCAACATAATACCTCTG
ATTGATTTCTGGCATTCTTCCTTCATAGATCTATTAAATGGAATTTTAATTACAGTAATGTTTTTGC
AACTAACTAACAAGTTGTTTGAAGTATTGAGAATTATATGATGCACAACAGGATTCTGATCATCT
GTGCTAATGTTATATTATAATTTTGTTTGTAAAACAGGTGGTACTATTGTACTATTTGACCGCAAG
ATGCTTAGGAACTTCAGAAAAGATGGGCACAATTGGAAAAAGAAAACCGATGGAAAAACTGTT

AAAGAAGCCCATGAACACTTAAAAGTGAGGATTTTAAATTTCGTGGGCGTTTATTTGAAATTGTG
TTAGCAAGTACCCCACTGAATGTAGTTTATTGAAGATGTATCCAGGGAGTTTGAATGGTTCCTCCT
ATATTGAATTTGGTCTTCTAATGGTTTTGCCTACTATAGTAAAATTGGTCTTTTTTAATAGTGTCTG
ATGTTTTGTTGTGCCTTCCTTGATCCAATTTAATTGGACTTTCAAAATGGTTTTACTGAATCTTTGGT
TGTATAGAGTGATCAATGCCGTTAGTTGATCAATCGTAGAGTGTTTTTGGCAAGATGGACTGTGA
AGGGAATAAAATATTGTGACATGAATATGATGGAGTATATCAAGTGGCCTTATGAGTTTTTAGGT
TAAATATTTAAATAAGCCACTAGAATTGGGTTAGTGGTGAGATGTTTAGATAGTTTTCATGAGATT
ATAGGTTTGAATGACCTGCCACCTTGATACTAAAATAAGTTAAATATTAAAACACTGGGAGCTTT
CTAAAGCTCAGTTGTGCATTTTACTAGTTTTATTGACACCATCTTCAATTCAATGTGCTTTCTTCAA
TGAGCCATGAGTGGTAATTTGTTGTTGGACTTTTGGAATTGTCACATTCTGTGGATTTAGACATCA
TTTCTGTTAGATTTAGGAGTAATAATTAAGTAATGTGATACTTCTAGATTTATATCACACATTCTCT
ATACTTTGGGTTGTAAAATATTTAGTTATATTTTTATCATTCTTTCTTGGTGACATTTGAGGAATAA
ATTATATTTCTCTTTAATATTGTCACCTTATTACACTATATTTTTTGTTATCAGCTTCTCTTCCTACA
GTTTTTGTTATATGTGATTCCAATGTTCGATTGAGTCACTAATGATGCGTGTCTAATTTTTTACATT
CCTTCTGTTCATTATTTTTTCTGTCATCAATCAGGTTGGCAATGAAGAAAGGATCCACGTATATTA
TGCACATGGCCAGGATAAACCAACCTTTGTTCGTAGATGTTATTGGTTGCTTGATAAGTATGTTTT
TCTGGACTTCAGGTTCTCAATTCCAATATATCATGCGTGTCTGTATTTGTGAACTGAGTTAGAAAC
AGGGGTTATACATTTCCTGCATTTTTGTTAAACAGAGAATAAAGATATGTTTGCAAATAGTAATGT
GACTTACAAATGTTATGATTTTATTTTCAGGAGTCTGGAGCATATAGTCCTTGTTCATTATCGTGAC
ACACAAGAGGTTAGAAATTGTGTTAGAATATTTTATTGATTTCTTTGTAATTGTTTCCTTTTAATTT
GCTATACATATTACATAATTAGGGTAGTTAGGCTGAATATCTTTTAGATATTGTAATATTTGTTCCC
CTAGTTATGGGAAACCTCTTTGATCCTTTGTACAAAATTTCTTTATGTATGATAAGGCTTGGAGAC
TCTCTCCCAAGCAAGTTTTTCAGTCAAAATATTGTTCTTTGTTTCTGTTTCAAGTTGGTAGCAGAGC
CTACATCAACTGGGCCTGATCATTTGAGTGTGTCTGGATGCACAAAAATTGTCTATAAGAATAAT
GTCTCACACCAACAATGTTTTTAAATTGGTCTGGTTGGAAATTTCATTTATTCTCTGTGTGAACTAC
TCTGGTACTTTATTTTTTTATTTTTTATTTTTGCTTTTTAAAATTTGGGAGGGGGATAAAGTATCTTG
TATACTGTTGTTTTCCAATGCAAGGCATCTGAAAAAAACTCCTCTAAAGGGAATTTGTGTCTAAA
GTGAGCAAGGGGTGTTTCCATCCTTAAATTTTAATGAGTTAAAATCACACTCTAATTGGAGATAT
AAGGTTTGTTGGTTGGTTAAGGAGGGAAAGGTCGTGGGTTTGATCCCTCCCGCTAACAAAAAATA
ACAATTAACATTTGTCAATAAAAAAAAATACACTCCAATTTAAGTTAAATCAAAGGGTGGATAC
ACCCTTAGTTAGAGGAGCTTTTCCCTGACTCCCAGTGTTGGATGTGTCATTATGCTTTACCATTTGA
ATTAGTTATTGAATAAAATTTGGCTTTCTAACCTTTGTTAGGTGGTGGTTTATGATACAGTTACAAT
TGCAAGGTTCTCCTGCTACACCTGTAAATTCAAATTCAAGTTCTGTCTCTGATTCAGCTGCATCTT
GGATTCCATCAGATGACTTGGATTCTGGGGTGAACAGTGCATATGCTGTTGGTGAGAAATAACTT
TAGATGATCTCAGTCTATGTTCTTACATTTCTTTGTAATACATCTTATATTCAGAAGATGTTAAGTG
TTTGTTAGGAAAAAAAATGGAGCAGGATAATTAGTTAAAATAATTAAATAAAAAAATATGCGCAG
TGTGCATGTCTTTTTATACATTGCAAATTTTTATTCCTCCCACTCCCGCCATTTTTGTCTTAAGCCCA
TCTTTCCAAAGGAAACCATAAATAAGAGCCTTTTTATTAGTTGGTTATGATCTAATGATCCTATAT
ATATGGTTTCTATTGTCGCTGTTGTCCATACTGTTGCTTTCTTTCCTTGACTAGGTATATAATATTAG
GAAGTCCCACATCACCTGCCTCAGTTCTTGGGATGTAGCTTATATATCTGTTGGACAACTTCACTT
AGTGCCTATTGATTTTAAGTTGAAATCTAACATATATCCTCATCCCTTTTTTCTTTTTTCTTTTTTGA
GATAAACAGAGCTTAATGACAGTTTAACTGCCAAAAGTCATGAGCAGAGGCTTCATGAGATTAA
TACACTTGAATGGGATGACCTAGTGGTTTCAAATGCCAACACTTCTACTACATCTAATGGAGGTA
ACTGTTTGCACTTTAAGAGAACCCTTTGCTTGTTGGGAAAGAACCTTATTATTTTATATGTTAAATT
TACTTTTGGCAGGCAATGTTCCATATTCATTTCAACAAAATCAAAGTTTACTGAATGGAAGCTTTG
GCAATGTAAGATGATTGCCTTTCTTCTATTTGTGTTTTTTATTAAGCTGTGTTGTGCTTTTTCATTTA
TGTTTGTGGAAGGTTTTATGGGACCATTACTCCTTTGAATTTTGTTAGATGAAAAAAGAAATTTAT
TTGGAGAGAAAAGAAGTAAAGCACAACTATGGGGAGGGTAAGATGTTCTTTGAAATAAAAAGA

GAGGAAAAAGAAAGATGAAAGAATGTGGCCTACCAATCTTTGTGTATGGGAAATGAAAACTCCC
TTGAAACAGCCTTGAGCAGCATGCCAAAGAAAGGCCAATAACACTCAAAGCAAATCTAGGGGT
AAAATGCAGGCATTCTTTTTCAATCAGCCATACCGACACAAGCTGCGCACAGCCAAAAACGTTTC
ACCCCCTTGTTGTTAGTCCATTATGAGTTTTCATTATTTTTTGGCTAATCCTTTTTAAAGTGTATGTA
ATACTGTCAAAAGAGACAGCTTTATATCATTTTTTTGCAAAAGGTTAAGTAGATGCTCAAGCATC
CAGGATCTAGCATTGCTCATGTCTTGTCTCACTATTGAAACTATTGTTCTTCTGTTGATTATACGAA
GTTATACACTGTTTTCTTTTCTTTTTATTTTTGGCACTTATTACTTGTTTTCATGGTGTTCTCTTGAGC
TAAGACTTAACTCAGTTGTACCCATTTACCAAAGTCCAAGGAACTTGGTATATAATATATCTTTCA
CACTTATATAAGTAAAATGTAAATATATTTTATATCTCTTAAATTGGCTGAAAAATCGTTCTTATC
AATAGATGAAACATAAAAGGTTATTGTTTATATTGATAAAATTTTGTATCTCTTAAATATATTTTC
ATGTTCTTCTGCTTCTTTCTGTCTTTCTTCTAGTGATACTTGCTTTGTGATAGCTGCTGTTGTAACCT
ATATAGGGCTTTTGTTACCAGGTATCCAGTGATCCTTCAGCAGAAATTCCTTCTTTTGGTAATTTA
ACTCAGCTAGTTTCTGGGAGCGATAGTGCTCCTTATAGTTTCCCAGAAAGTGCTGATCTTTTGAAA
AGTAGTCCTTTATCTTCGGGTGGTGTTGACACCTTGGGCACTTTGGTCAACGAAGGCTTGCAAAGT
CAGGACAGCTTTGGAACATGGATGAACATCATGTCGGATACACCATGTTCCATAGATGAATCAG
CTCTTGAAGCAACAACTTCTTCTGTTCATGTACCATATTCATCACTGGTGGCAGATAATAAGCAAT
CTTCTCTGCCAGAACAAGTATTTAATCTCACTGAGGTGTCCCCTGTGTGGGCATCTTCCACCGAGA
AAACAAAGGTTTCCTTTTGCTTCTCATATGTCTAATTGCTATCAATTATTTTCTGTTATAATTGTCTC
CTTTAGCACAAAATGTAATATTTAGTTTCTTGGTTAAATTCTCTGCATTTTCATTCAGTCTTGGACA
ATATAGTATGGCTGTGGAATTTCAAAGCTTCAAACTATGAAACCTAAAACTCCATGAGGAGCAC
ACTATCAATTCTTTTTCAGTGTAAAAAAACAAAGAGCATTTTCAGTAGGCACCATTAGCACTCTG
GCTACAAGCATGATTAATTCATATGTTTGCTACCATTTTTATTTTTTTTGCAATTATAAGTAAACAT
TTAATGTCTCTTTACTTCTTGCAGGTCCTGGTAACTGGATATTTTCATAATAACTATCAACACCTTG
CAAAGTCCAACCTACTGTGTGTCTGTGGTGATGTAAGTGTTCCTGTAGAAATTGTTCAGGTTGGAG
TTTACCGGTGCTGGGTATCACCACACTCTCCTGGATTGGTCACTCTCTATTTGAGCTTTGATGGCC
ATAAGCCCATCAGCCAAGTTGTTAATTTTGAGTATCGTACTCCTATTTTGCATGAGCCAACTGCTT
TAATAGAAGAGAAATACAACTGGGATGAGTTTCGACTTCAGATGAGGCTTGCTCATTTGCTCTTT
GCTTCAGACAAGAGCCTTAACATTTTCTCAAGTAAAGTATCAACTAATGCTCTGAAAGAGGCTAG
AAGATTTTCCTTCAAAACATCATATATCTCTAAAAGTTGGCAACACTTGATGAAGTCAATTGATG
ACAAAACAATTCCATTTTCACAAGTAAAAGATGCCTTGTTTGAAACTTCATTGAAAAACAAATTA
AAGGAATGGCTTTTGGAACGAATTATCTTAGGCAGCAAGAGTACTGAATATGATGCGCAAGGGC
AAGCTGCAATTCATTTGTGTGCAATGCTGGGATATAATTGGGCAATTTCCTTATTTACATGGTCGG
GCTTGTCGCTGGATTTCCGTGACAAATTTGGATGGACTGCTCTTCATTGGGCAGCATATTATGGAA
TGTATGGTACATGGATATCCTAGCTGCAACAACATTGACCTAAAATTCATTTTCTGACTAGTTATA
ATTTTTTTTTAAAAAATCATTTTTTGACTTGATTTCACTGTTCATGTTCTTTTCAGTTTCCTTTTACTG
TAGTGATAAACAGGCGCAATGTAATATATATGATCTTATCATGAAATTAGGCACTATATGATCTT
TTCAGTTTCCATATAATACTTGTGTGCATTTTTTTCTTTTTAATCCACTAACGAGCATGAACAAACT
TTTCATTGTTTATTTGATTCAAAATTTAAAATTTGTGTCTGCAGAGAGAAAATGGTTGCAACTTTGT
TATCTTGTGGGGCAAGGCCAAACTTGGTCACAGATCCAACTCCTCAATATCCAGGTGGATGTACT
GCTGCTGATCTTGCTTATGTGAAGGGATGTGATGGCTTAGCTGCTTACCTTTCTGAGAAGTCTCTG
GTTGAGCAGTTCAATGACATGAGCTTGGCTGGAAATATCAGTGGCTCACTGGAAACTAGCTCAAC
TGATCCAGTAAATGCTGCGAACCTTACCGAGGACCAACTTTACCTAAAGGAGACTTTGGAAGCTT
ACCGTACAGCTGCCGAGGCAGCAGCACGTATTCAAGCTGCATTTAGGGAGCATTCATTTAAATTA
CGATATCAAGCAGTTGAAATTATGAGCCCAGAGGAAGAAGCACGTCAGATAGTAGCAGCAATG
AGGATTCAACATGCCTTTCGCAACTATGAGTCAAAGAAAAAGATGGCAGCCGCTGCTCGGATTC
AGCTTAGATTCCGTACATGGAAGTACCGTAGAGAATTTCTCAATATGCGGCGTCAGGCTATTAAA
ATTCAAGTAAGACCTACTCTTCCTGTCGGTTCTTGTTGATTGTCCTGAGTAAACATGTCATAATGA
AAAATGAAGTTATTTGTTCAAGTCAATTTTTGGATGGATTGACTGTGGATTCATGTCAATTGGCAC

ACATCTTAATGTGTTCTGTTTCACAAGTTATGAGAACATTCATGTTCATCCAAGCACTCTTAGTGA
CCACATATTTGAACTTGACAAGTCAACTGTTTTTCTTGTTGACTTTGAATAATTTTTACCTGTTTGA
GAATAAGGAAAATTACCACAGTCATGGCAAAGAAAGGTTACCCTTAAATCATAATCTAAAATAG
AACTTATACTTTTGATAGAATGTAGGTGTAGTATAATACCACTTGAATTTTGAACTTAGGAATTGA
CTTTCTTTCCCATCTAATCATAGCTTACTCTCTAAAGTTTAATTAAGACCAACAGCTTGACTGGAT
AATACAATTTACACCCTGGAAAAGATTAATGGCATATACTTTCAATGCAGGCTGCTTTCAGGGGC
TTTCAAGCACGGAAGCAATATCGGAAAATCGTATGGTCTGTTGGGGTGCTTGAGAAAGTGATCCT
GCGTTGGCTTTTAAAGAGAAAAGGCTTCCGTGGTCTCCAGGTTAATCCTGCCGAGGAGGAGACA
CAGGAAAGTGATACAATTGCAGAGGAGGATTTCTTTCGCACCAGCCGGAAACAAGCCGAAGAA
CGTGTTGAGAGGTCTGTCATACGTGTGCAGGCCATGTTCCGTTCTAAGAAAGCACAAGAAGAAT
ATAGAAGGATGAAGCTGACACATAATCAAGCAATGGTGAGTAACACATGCAAATGTCAAATGTC
TCATGTTTTTATTTTATTTCATTGGCTTAAAATAAGTTTTTAGCCCCTCAAATTTGAGACATTTTTGC
TTTTGGTCCTCTGATTAAAAAACTGCCCTTTTTAGTCCCTCAAATTCATAAAATGTTGTTTCTAGCC
TCTCAACTTCACTTTGGGGGACTAAAAAGACAAACCAAACTTACCCCAAATTTGACGAACTAGA
AACATGTTTAAGCCTATTTCATTTCACATGTTTGTTTACTTTGTTGAACTGCTTTATGCAGCTGGAT
GAATTAGAAGAATTCCTAAATTCTGAAGATGACATGTTACCCAAGACATAGCTTGGTGCCTTTTC
CAAAACAAGGTTTCCCCATATTTGGGATGTAGCCAACTCCTGTGACAAACTAGTGTGTAGTGATA
GCTTGGTGTCTTCTTTTATATAGGAAGCAGATCCTGTAAATTACCTAGTGTAGATTTTGAACCCAT
GACCCTTTCATATTGCTTTAATGTCTTGAATGGGTCGTCCGTGTGTAATTAAGCTTCCCTATGAGG
GATGCTTCTAATGCAAGATCAAGTTTGTAACTTTGTTTTTGGTGAAAAATCAAGTTTGTAACTTAA
ATAACAAGCCTTCTTAGATTTGAATTGACATAATTGTTGTTGGAACTCTTCTATGTAATAAATTAA
GAGTCCATTATATGATGATTGTTGGGTTTATATTTTATATATTGACTAAAAC

>Glyma.05G148300 (GmCAMTA10)

CTCAAATCAACTCTAAGCTAAGCTTCAACTAACGCCAACTGCGTCTGTTTCAGCTTTCTGCTTTTG
CCACTTCCTTTCTCAGATCCATTATTCTTCGGAACCAACTCGCACCGCAGCGAATCTAGCAGCTTC
TGCGGTTCCAACAATGCTCTGTGTCCGGTTCTACTACTTGTAGCTTTTTATTCCTCGGATTCAACAC
CATGACACCTGGTAAACGTGTCTTTACTTTTTCTTAATGATTAATGGTTGTTATTATTATGTCTTGTT
TAGCAAAAGAAATTGGAAAATTGGGATGTTTAATGTTTTGGTGCGATGTATAGTGAATTGAATTG
AGTTTGACTTGGTTTGATTGGGTAGTTGAAGTGAACCACATCACTTGTGACTTTGTGTGTGTGCAT
TCTGTTTAGTTTTCTGAGGTTGGTGCTTTCAATAGGGACCATGTGAGTTTTGGAATTTGTTTGGAAT
CGGTGAAAATCAAACACTCGCACTGACAGTAAAGCACTTTAGTATATTCTCTGTAGCATGCTTTTT
GCAATTTGATTTAATTTACTCAATTATTTTGCTGCCCCTAACACATTTTGCTTCCAAATGATATGGA
ATATTGGAATAATTCATTGGTGCTGTACAGAGAAAAAAAAAAATTAAGTTGTTAGTTACGTTGTGC
AGTTCTTATTAATGCATAAAAATGTCCCCAGCTTATCTAATTCTTCTGCTATAAGAAGGTAGAAA
AGAAAACAATGATGATGATGATGGGAAGGGTTCTAAATTATTTGACGTATTTATGTTGTGGTTTTA
GGTTACGAATACGATATTAATGATCTGCATCAAGAGGCTCAAGCAAGATGGTTGAAGCCTGCAG
AAGTGATGTACATTTTACAGAATCATGAAAAGTTCCAGTTCACCCAAGAGCCCCCACAACAGCC
AACCAGTAAGATCTGTGTTCCCTTTATCCAATTGATTTTAGAAATAAAAATAAATGACAGTTTTTG
TCATTGTTTCTTCAGAGTTGACCATAAAATGCTGATTGTTAACATTATCCATTGATCTTCTTAACAG
GTGGATCTCTGTTTCTGTTTAACAAAAGAGTCCTGCGTTTCTTCCGTAAAGATGGTCATAACTGGC
GTAAGAAAAGAGATGGAAGAACTGTGGGAGAAGCACACGAAAGGCTGAAGGTGTGTTAGTAGT
GTCTATACTATTGTGTTTATTAGTTGTCCATGAAAAAGGAAAACCTGTTAGCTAGTAAGTGTCTAG
AGGGGCTATCAGTTGTAGAAATGCAGTCCATAAATTAATATTTAAGAGGGGGAAAATTGTTGACT
CTCCTGAATTGTTAGAAAAAATATATTTGTCTTTGATAGATGTCTCAAACTTGGTGAATGGTGACA
GATGGTATTATTAAAAATATTTAAAAATCCTTATTGGAAGTTTCATTGAAGGCTTTCAAGATGCTT
TTGAAATAATGAATGATTTGCCTATCAACTGCCCTTATATACATTAGATATAACTTATTTCTGTTTA

GTGATTCTTTTTTAGCATGTTTATCAAAACATTATTCACTTATTGCTGCTATATTTATGAGTTTAGG
CCAACATTTGTTTTTGTGATGGTATTCTGTATACTGATTTCTTCAATTTAAATGATTGTACTAATCT
GCATGACTAGGTTGGAAATGTTGAAGCCTTAAATTGTTACTATGCACACGGAGAGCAGAACCCT
ACTTTCCAGAGGCGGAGCTATTGGATGTTGGATCCGTGAGTAAATTTTAACTATTAGCTAACTGTA
TATCATTCGTATTATTAAGCTTATCACTACATGTTGTATCCTTTAAAACAAACATGCAATTTTCGG
GAAAAAGCCATCCTAGAGGCTAACTAAATTTTAAGTGAATTGCAAAGATTAGTTTATCCTAGACT
TTGAAACAGAATCAGCCTTCGATGTACATAACTATTTGTGTGATATGCTTATATATATGTTATTGT
AATAGTCATTCATAATCACTCATACTATTGTTTCATGGCAGGGCATATGATCATATTGTTCTTGTG
CATTATAGGAACACAAGTGAGGTGAGTCTCGGATACGTGATATCAGATAAACATAATAATAATA
CTTGTAGCTTGTGCATAGTGCCCCTCCAGAAAAATTTGGGGAATCCTTTTGTGCAAGATTGCTCAC
TGTCGTAACATGTTTTGGATTAGTTGTTTTACACACATCCTCCCTATTTACTGTTCACTTGTGATAC
TCAAATAATTTAATTCATTTTCCTGTTCCGTGAAATTTCAAACATTTTCTGTATTCTTTCATATTTAC
ATTTTGTAGATGGCTCGTGCAATTGTCCATTAGCTGTTAATATTTCTTTTTTAAGTTTGGTATGTTGA
TTCTTTCACTCACCATTATAATTACTACTGTGTTCTAACTCCCTCTCTCCCCCTCCCCTCTCTCATTT
TCTATTTTCAAACTTCATTTAAAAATGTACATGTATGCTTCCAAAGTCATTTAATAATGAATTTTAG
TAAGCACGGATAGTGTAAAGGTTTTTTACATTGTCATCCAATTGTAGATTGTCATGTATGGTAACA
TTGTTGACTTGTAATAATTTCTAATTGCGTGAATATTAGACCCTTTAATAATTACTGTGCAAAACA
ATTCAACTATGAATTAAAGTCATTCCCCATACATTCTCTTAACTTCTTGAAACTTTTTCTATTCCCT
ATGTTTATGAACATCTCTTTATGCTCTTCTACCAAGCAGAACTTACCTACGGAGATGCCTACACCT
CACCTCCCATATCCTAATTTGAAGACCAGATGCACCTTGGATAACCATTTTCCTACTTATGAAGCT
TTGATAGTTAATTCTCTATGATAAGGACGATTGGTATAAGAAAAAGTCAGTGATTATTCCAAAGA
TTTGTAATGTCCTGTTGCTTTTCTGCACTAATTATTTCATTTGATGGCTTTGTGCCTCAAATAAAGA
TATACTTAAATTTTCTTAAGGCCTTTAATAAAAAAATCGAAATCTTCTTAAGAATCATATTTGGTA
TCTTGGGTGGAATTATTAGATTATATTGTTTTATTCTCTGTGTGCATAGGAACATTGTTGTGCAGCC
CATCTTTGAGTTTTGCATTAATGCATTCACTCTCATGTTATAGGGAAAGCTCAGTTCTGGAGCTGG
GGCACAATTGTCACCAAGTTCCTCTTCTGTGTATACTCAGAGTCCTAGCCCATATTCTACTCAGAA
TCCAGGGTCAACATCCATACTTGGCGATTCATATGAACCTAATCAGAGTTTCTCCAGTCCAGGGT
CTACAGAAGTTACGTCTGATATGTTCGTACTGAACAATAAAATGGGTCACATGGATGGGACAGA
TACAGAATCAGGAACCTCGCCTGAACTCGAGGTTACTCAAGCTTTGCGCCGATTGGAGGTGCAGT
TAAGTTTGAATGAGGACAACTTTGAAGATATTGTTTCCTTCGGAAGTAAGCACGAAACTACACAT
GATTCAAACCCTCAGCACGACCAAAGGGTGATCAGCAATCAAGAGCAATCTGCTGCTTTCTCTG
GACCTGATGATCAAGGGCTATTTTATGATGGATATAATGGAAGGCAAGGTAAATTAAATAAACTT
GATCTGTTGAGAACACATGGTTTAAATCTGTGAAATATTTTAGTGCTTAATCTATATTGTGTTTTAA
CCTGTTCACATCCAGATATATGTCTTCTACTTATCTAATTGTGCTTGCAGCATCTTACACCTGTACA
GCTAGTTTTCTGTAATTTGTTGATGGAAATTCATAGCATACCTGTTTAGATTAAGTTGGTCACAAC
TATCATAGCCATCTTCTTGTTTGGTGTATTTGTTTAATTTTTCGCACTGAAACATAATTCCCTAATT
GTTATATGCTGGTTGTGTTGTGTGAATTATTAGAATTTGGGTTTGAGATTAATTCAATCTCAAAAG
CTAGCTCATTAGGTGGTAGTTGTCCCCCCACTTTTATATATTATTTTAGTCAATGTTGGACTTGGGT
TTTTCCCAATATGAATCTTAAGAGAAATATGTGATTCTTATTATAAGCCAAGTAACCAGGAAAAA
AAATGGTTCTAAACAACTTACTCACGGCTAGGTTTGTTTGTATTTGACATTACTCCAGTAGACTTT
ATAAAAACATAAAAATGTGGAACATGCTGTAAAGGTTTGATTGGAAAATTCCATATGTTACATA
ATAAATAAGTGTTTTTGTTCTTTTATATTGTTGATAAGGAAAATAAAATAAATGTTCTAGTCTTGA
TAAATTGCATCTCGCTGACAGGTTAGCACTTGACTAACCTTTAATAATAACAAGCAAATTTGAAG
TTTGAGAGAAAAGGTTGTGATATTATTTTAAAAGATGCCATGAAGAATATAACACAACATACTTA
CAGGGTGGTGATAGCCAACCTGAATAAGGATCCTAATTAAACAAGGAAAATACTAAATTTAAGG
AAAAATTCAAGGAAGATACTAAAACAGTCTTGAAGTATACTTATACAATGGATTTTGGGCTCAA
AATCACGCTCAAGAAGGTAAATCCCGGTTGCAACAACAAGAGGCTCGAAAGGAGCTTTCTGTTG
ATATCATATAGGCCCTGTAATTCCAACTAGATAAAAAGTTATGGTTGTTTAAATCTCACTTTCCTA

TTTAGGATCTATGGGTCATACGTGTGGGTTTCCGACACTCTACTCACTCTAAGAAATCTTTTGTTA
CATTTGCCGCCAATGGTTAGTTTGTGAAGTTATTGTGGTTTTGCCTCTGCTTGAAGTTTTTCCTAGT
AAATTAGTTATTATTGTTAGACAAACGGTCTCAATAACTTAAGAGAGGGGGTGGGGGGGTGAATT
AAGTCTTACAAATTTTCCCGTTAACAAATTTTAATTCCCTTTTAAATGATATATGATAGGCTTAGA
ATGCAGAAGAAGAAGAAGCAATCAATTTAATAATGTTCTTTTAAATGTGCAAGACAAAGTAAAC
TGCAATAAAATAACTGAGATAAGGGAAGAGAGAAATGCAAACTCGTTTTGTCCTGGTTTGGTCA
CTTCCCGTGCCTACGTCCAGTCCTCAAGCAACCCACTTGAGATTTTCCACTATCTCTATAAATTCTT
TACAGATTTTGAACACACCTTGGGATCCCTTATCCTTGTATACAATATTCTCGCAATCCAAGAAAC
AAACAGTCTCTTGATTACAATTGAGTTTATGAGATGAATAGAAGAATTTCTCTCCTTTAGAGTAA
ATGATACAAATTGAAGTTCCTAGATGAATTCTCAATAAATTTGCAAGTGTTTGGTCAAGAGTTGTT
TAGAGAGGAATTGACAATTAAGTTCTCTTTGAAACTCTCTCTTTTGTATACTTTTCGAAGTCAGAC
ACACATATATATAGGCCCATTGTGCCTTTCCAAATGGTTTGAAGAGATGTATCTTTTCAAATAGTT
TTTCTGAAGTTTTCTCACTGTAATCGATTATAGGATTCTGGTAATCGATTACACAGTTATATTTCTG
AAAAGTCATGACTTTTCAAAGGTTTTTTTGAAATCTCTTCACTGGTAATCGATTACAGGATTCTGG
TAATCGATTACACAGATCAAAATTCAAACAGTTTTATGCCATTAGTCAAAAATATATTCACAAAC
TCTCTAGTAATCGATTACACAATCTGGTAATCGATTACCAGTTCAAAAATATCTTTTTAAACTGAT
TTAGCCTTTGATACTAAAGATGTTGAGGCCAAACTAAAGCAATCAAATTGAGATTCTTTAAACAA
ATTTACTAAGTTTTTATCTTAGATTTACTTGATCTTGTTATTTTGACTTGATTCAATCCATGCTCATC
AAGTAACCTTTTTGGCATCATCAAAACCTGCATGATATTCATTCACATTTTCTCCCTTTTTGATGAT
GACAACTATTATCAAGTAAATTCTTTTGGCATCATCAAAAACCTTATGCATACTTTCACAATTATC
TGTTCAGAACCTTTTTTTTTAACTTTTATTTTTTTAATTTCAAGTGAAATTCAGCTTTTTATAGTTTGT
TTTGTCCTCTTCATTATGGAAAATCAAATGGGAAGAGAGGAAGGTAAAAGATGCTAAAACTGTTT
ATTGTTATAGGTGATGGTGGTGAATTTTATCATGAATTAATAGACCATGGTTACCCTGATGGAAAT
GAAAAGGCTTTATGGACAGAGGTGCTGGAATCATGTAAGTCCTCATCTGCAGTCAAGTTACCACA
GAAAAATGTATACATGCCAGTTGAAAATGTAAGTGTTGCTTCGACATATTTAATAAAATTTATAT
CATTTTGCAATTTTATGTTTGCTCAGATGTTGAAATAATTTTCTTCATGTGTTTCTGCAATACTTAA
AATGTTTGATTCTTAAAGCTGGAAAATTCAGTATCTTCTGCAAGAAGGGTACCAGTTTCCAACCA
GGAAAACAGTCACTGGCTCAACTTCAACAGTAATAATTCTGAAAACTGTATGATTTACTTTCACC
TGGTTAACATTTTTGGCATATTTTGGTGTTTCTTCAACTTCTGTCCCCTCCCATGTTAGCAGCTGTTT
TCTCACAACCTCAAGGTGTTGATGAAGTCAAATTTCCTGTCTATTCTTCTATGGTGGAAACACAAG
TAATTAATTCTGACTACTATGAAACATTGTTTGACCAAAGCCAAATTGGAGCACCTCCAGATGCA
AATTCAAGCTTGACTGTTGCACAAAAACAGAAATTTACAATTAAGACAATCTCCCCAGAATGGG
GTTATGCCACTGAGACTACCAAGGTTTGTGCCTTTAAAGCCCAACTCCTTGCATGCCCTATACAAT
GATTTATTACTCTTTGCATAACTGGTATCTTGTGAACAATGAAAGTGCTCATTCTTTTGATTCATAA
CTATTGGTGCTTATACCTTACCTAGGTTCCTGAAGAGCTTTAAAAAAATTTCTTAACTTAGTTGAG
AAACTTTAAATCTATATGAAATTCTTGTGGTAGTGGTGGTATAAAGCAATTATGTATAAAGCTCA
GTCTATTTATAATAATTAGGTTAGGGAAATGTTGCTCAAGCTTGTGTCTTTGGACATTTAAGACTT
AACAAGTTGGGGAATACATGTCGTCTCTCTTTTTTTTCCTTTCATCTTTCTTATCTTAAAAAATAAT
GTTTAGTGTTGATTTTGTCCAAATTTGATTCCTAAAGCTTCATATGCATAGGTCATTGTTGTTGGGT
CTCTTCTCTGTCATCCCTCGGATTCTGCATGGGCTTGTATGTTTGGTGATGTTGAAGTTCCTGTTGA
GATAATTCAGGATGGTGTAATCTCTTGTGAAGCTCCATCTCACCTTCCTGGAAAGGTTACTCTGTG
CATTACTTCTGGAAACCGGGAGTCCTGCAGTGAGGTCAGAGAGTTTGAATATCGTGATAAGACC
AATAGTTGCACTCAATGTACTCAATCAGAAACAGAAGCCACTAGAAGTCCAGAAGAGCTGTTAT
TACTTGTTAGATTAGAGCAAATGCTTCTTTCTGCATCAACAATAAAGAATGACAACATAGAATCT
GGAATTCCTCTCATAAAACAGAAAGCTGATGATGACTCGTGGAGCCATATTATAGAGGCTCTTCT
AGTTGGCAGTGGAACGTCGACTGGTACTGTTGATTGGCTTCTTGAAGAGCTTCTAAAGGATAAGC
TTCAACAGTGGCTTTCTTGCAGATCTCAGGAAAAAGATGAAGAGACAGGCTGCTCTTTGTCCAAG
AAAGAGCAAGGGATTATTCACATGGTTGCTGGGTTAGGTTTTGAGTGGGCCTTGAACCCTATTCT

CACTTGTGGAGTCAATATAAATTTCCGTGACATCAATGGGTGGACTGCCCTTCATTGGGCTGCAC
GTTTTGGAAGGTAATAGAGAACTCTAGAGAGCTTGCTACCATGTTTACTGATTTATTTAGTTGGGG
TGAAAGATGTTAATATTGCTATACAAGGCTTATAGGTAATGAAGATAGGGAAAGCATACATACT
TGACAAACAAAATGTGCTTGGTAAAATATGCATGTAATTTTATTTTTATTTTCATTTGGAGAAGGG
AATGCAGAAGCAAGGAAAGATAACATGGAGAAGGAAAGGAGAATAAACTATCTGATCTCTTAT
TATAGGTATAGAGCACTAAAATGCAAGGAAAAAGATAAATGTTTCAGATATTCAAGTCTGAAAA
TTAAATTTCTGGAGATGCAGACTGCAGGATAGATGTAAGAAAAACTGATGTTCAGTTAAAATGAT
ATCAAAATACACTGCTTTCAAATTCTGAAATTAACAAAATCCTTGTAGGTTAAGTTAATTGGCTTT
CTGCAATTCCTAGCCGGTAAGTCAAAAGTAGAAATTGATGACAATACTGAGGGTATAATAACGA
CATGTAGCATGATTTCTTAATCATTCAAAGCAAATGCTGAGTTGCTGACTTTTATTTCTAATTAAA
AAAATTTGGTTCATCCAGTCATCTGCCAGAACTGATGATCATATCAAGAAAATTAATGTCTTGTTT
CTTCAGAAACCATTTTATAATTATCTGTTCCATTTTAAAGTATTTTGGATGTTATTTTCCTGAAGCT
TTGTTATTCCATATAGGGAAAAAATGGTTGCTTCACTTATAGCTTCTGGAGCATCTGCTGGAGCTG
TGACAGATCCAAATGCACAAGATCCAACCGGTAAAACTGCTGCATCTATTGCAGCCGGCAATGG
GCACAAGGGACTGGCAGGGTATCTTTCAGAGATAGCTGTAACAAGCCATCTGTCATCTCTTACAC
TGGAAGAGAGTGAATTATCTAAAAGTTCTGCTGAGCTTCAGGCAGATATGACTGTTAACAGTGTC
TCCAAGGAAAATCTTACTGCCAGTGAGGATCAGGCATCACTCAAAGATACCTTGGCTGCTATCAG
AAATGTAACTCAGGCAGCTGCGCGGATACAATCTGCTTTTCGTTCACATTCTTTTAGAAAACGAA
GAGCAAGAGAAGTAGCTGCTAGTGCAGGTGGCATTGGTACCATTTCAGAGATTTCTGCTATGTCA
AAACTTGCCTTTCGGAACTCACGTGAATATAATTCAGCTGCTTCAGCTGCTTTATCGATTCAGAAG
AAATATCGAGGTTGGAAAGGTCGCAAAGATTTCTTAGCGTTGCGCAAAAAAGTAGTGAAGATAC
AGGTAATAGATGTTAACTTTATAGTTTGACTAGAACAAAATGAATTCCAAGAGCTAAATAGATAT
TATTCTTAATTTATATATAACTAGCACTTAAAGATACTCTGAAAGTGAATAAATTTGATTTTCAAA
CCAACAGTTGGTTATAGGCAAGCTACACATGCACGAGCATACTTTGATCATCTTCAATGGCATTA
TTATCAACCTTAGTTATTTGTAATTTATTTGGGCAGGCTCATGTGAGGGGCTACCAGGTTAGGAAG
CATTACAAGGTAATATGGGCAGTTGGAATCTTGGACAAGGTTGTCTTACGATGGAGGAGAAAAG
GAGCTGGTTTACGAGGTTTCCGACAAGAGATGGACATCAACGAAAATGAAAATGAAGATGAAG
ATATTCTCAAGGTGTTCCGAAAACAGAAAGTTGATGTAGAAATTGAAGAGGCTGTTTCAAGGGT
GCTGTCCATGGTCGACTCACCAGATGCTCGTGAGCAATATCATCGCATGCTTGAGAAATATCGTC
AAGCTAAGGTAAATAAATTAGTCTAAAAGCCGAAGAGTAAAGTGTTTGCATTTTATTAAGCAGA
GCAACCAATATCTCTTTAGCTTGTGTTAGTTTGCCACTAGTTGAATAATTTAAACATTGTTTTGCAT
GAGGCTTCGATGGGGCATTCCGTGCTCAATCAGAAATAAAGACAAAATAGTAACCATACAGAGC
TATCAACCATTTTGACCTTTTAGAATGCAATTGCTTTGTGCTGTTAATGCATAACTTGTCAATTCCC
TTGCAGGCTGAACTTGCGGGTACGAGTGACGAGGCATCATTATCAACTTCTGTAGGAGATGACTT
ATTTATTGATGATTTTTATCCATTTCCCTAGGAGCAACGCTAGCCTTGGTAATCTTCATTATTTTGA
ATGATCAATTTGTGGCACAACCATTTCGATGGTCTTTGTATCTAGATGCCTAGAACAAGCTGACTT
ATTAGTTTGAGACAGTCTAGATTGTATAGTGAAAATAACAATGAACATTGTTACTTTGTAAAATA
TGAAATGGTCTTGAATGCAGGTGGGGTGGGGTTTCGAATTGAATGGATTAAGTTTCTTTAGCATCT
CTTTGCGTGCACTCTTGACATCTTCGCGAATTTGATATAGCTTATGGCTGCTACCTTCTTTTACAAC
TGTACAAGGTATCTGAAAATGTAGCATCATTGGATGAAATGTTGGAATGAGGTTAGCTTCGGGGT
TGTTTGAGGTTCGAATAAAAATTTGCTATTAGGGTTGTTTGAGGTTCGAATAACAAATTGCTATTA
TTGTTACTATAGGGAGGTATAATCACATATTTAGTCTTTATAATTGTACAAATT

>Glyma.18G005100 (GmCAMTA11)
TTTAACGAATTAAAATCACTTGGTTACTAAGAAAAAGAATTAGTAATGTGTGTTTAGAATTAGAC
AAAATAAAAGTGTAGGTAAGACAGAAGGAAAAAAGTAAACAAGAAACATTCTGGTTCTCTTAG
GTGATTGCAAATGCTCAGAATTAATTGGGGGTATAAAATAATGACACCAAACCTTTTTCAACCAA
GTTATCTCTGTCTGTCAGTGGGCCAGTTCAAGTCTGAGAAAAGGCCGGTGCTTGTGTTGTGTGTTG

TGGCTTGTTTAACTCTACAATAATATATCATCATCAACTTTTTCATTCCTTAGGTTTGTGTTTGTGG
AGAAGCTAGTGCTATTTCTCCAATTTTAGTTCCCCTATCCTCTCCGCGATTAATTACTATGCCACCT
GGTAAGACCTTTCTCTTCATATTGCTCCCTTCCGCTTTTCAGTTTTACTGGGGATTAGCGATGCTTA
ATCAAATCTCAATATTGTTGTGTTTGGTTTGACGATAATACTCTAGTAGAACTCCAATTGATGGCC
AAATAGAAACAAACTTTTGTTTCTGTGAATCTAGACTTAATTTGATTGTAAATTTATGCTTGTTAAT
GCTTCACACCTTTTTTTTGGAAAATACGGTTGGGAGTTGGGAATCGAGAGTTAAGACAGTACTCT
ACAGTTCACTTTTGCTACCACACAACTTTTACTTCATGGACCCAATTTTTGTCCTTCTTACTCACAC
GAGGGAATAATTGATTGGACTGATATTACAGAGATAGAAAAGAGGATTATTAAGTTCTTACTTCT
TAGTGTGTGTGATCACACTTAGGTTCTGCATGATTCCCTTGTCCCTACCAACCAAGACAGAAGAT
AGGAAGTAACAATAGGATGAGTTATGAATTGTTGGACTAATTTGACATTGTCATGGCAGGTTTGG
AGTACAATATCGATGATCTGTTTCAAGAAGCTAAGAGGAGATGGCTCAAGCCTGTGGAAGTGCT
TTACATTTTACGGAATCACGACCAGTGCGAGTTCACCCACCAGCCCCCTCATCAGCCAGCTGGTA
ACACCCGTGCTCTCTTTGTTGAAATTTATGAAAGAAACGAAGGACAGTTTTTCCGACTCTTATCT
GCTACTGGATTATGGGTTACTGATTGTCGAAAATGTTGCCAATTGACTTTCTTAACAGGTGGATCC
CTGCTTCTGTTTAATCGAAGAATCATGCGTTTCTTCCGTAAAGATGGTCATAACTGGCGGAAGAA
AAAAGATGGCAAAACTGTGGGAGAAGCGCATGAACGGCTTAAGGTCTGTTATTAGTGTCTTATG
GTGTTGTGATAATTAAAATTACCTACAATGATCATTAAATTTTTTCACACGAGGGTGAGGCATGGT
GCAACTGTAAGGTTACTACCTTGTGACCTTAAAGGTCACAAGTTCAAATCCAAGAAATAGCCTCT
TCACAGGCTACATGCATCTACCCTCCCAAAACCTCTCCAAGCGGGAACCTTGTCTGCTAGGCTGT
CCTTTTTTTATTTGACATCTATTTGTCTTGCTGCAGCTCATGTCAGCCACTATCATCTGCCCACTTTT
GACATGCTTTATGTTTTATGCAAATGCTTCAATTTGCCCTCTAATTTGGACCAAGCTCCTTCCTTTC
TTTTAGTCTCAACTGTCAAATAAGAACAACGCAGAAAAAAAGAGAAGATGAAACATACAATAA
TCATACCATACTAACAGCTTGAAATTTTCATACCAAAGGAAATTAGAGCTTCTTCTACTTCATCAC
TTTCTTTATACTTGGATTTGGTTTATTTTCTTGCTTTAATTGCAAAAGTCTGCTAAGGTTTCAAGCA
GGTCTGAGTGATTAAATTATAGTTCATAAATGTAGTAAATTATTAGGAAGAAGTATTCAAATTTC
AGGCATATTGAAACAAAGTATTATTCGAAACTAATTAATGATCCTCATTGGAAATTTCATTTTCA
AATCTATCTCCTAATCTGCTCTGTGCCATATATAGCTTCCAAAAAGCTTTTGGAAAAAATAGTAA
GTATTTAGTTTGTCCAGCACTCATTATGTGCATGAGCTGTAACAGAACATTGAGATACAACCAAT
TTGTTGTTTAATAGTTTTACTGGTATGGAGCTTTTTTCTTTTTTAATTATAATTCACTTATTTTTGCTA
TGTTGATGCCTTTGTGCCAACATTTGTTTTGCCATAGAATTCTATGTATTGATTTTCTCAATCTGATT
GATCATTCTAATCTGCATGGTTAGGTTGGCAATGTTGAAATCCTAAACTGTTACTATGCACATGGA
GAGGAGAACCGTACTTTTCAGAGACGGAGCTATTGGATGTTGGAGCCGTGAGTAAATTTTAACTA
TTAGCTGGATACCAACCATATTATTATGTTACCACTACTTGTATTGTATCTTTTAAGATCTACCTAC
ACACTTACAAAGCTATATTAAAAGGCAACTTTAATTACCAAGATTAAATTATCTCGACTGTGAAA
CAAAATCAACTTTCAGTATATGTAACTTGTATTAAGATGGGTTCATTTATATGCCAATATGTTATT
GTAGTGCATGTTCATATTTGCTCATACACTATTGTTTCTTGACAGGGAATATGACCATATTGTTCTT
GTGCATTATAGAGAAACTAGTGAGGTGAGTTTCCAGAACATGATATCAGATAAACATAATATGG
TACTTGCAGCTTATTATGAGTGTGAAAAGGTGGGAATCCCTTTTGTGCAACACATCTCACTGTCCT
ACACTCCTACCCTGTTTCTGAAAACTAAAATAAAACTTTAAGCACATCTTCCCTTTTCAGTGTTCA
GATTTGAAGCTTCAATTAAATAATTTTGTTTGTTTGTTTTTCTTATGAAATTTCAACCCTCTTTAGTA
TTTCTTATGAATACTGCATTTCTGTATTCTCTAATTCTTTACTTTGTGTAAATGACTTACAAAGTAG
TCCATGAGCTGTTTATATTTCTTCCATAAGTTCAACTCTCTCTCTCACACAATCACATTTTTCAGTT
CTAAAACTCTCCAATAAATTAACATGTGTATTTCCAAAGTAAATTAAATTGTGATGAAATAGACA
TTTGCAATATCTTAACTTCTTGAAACTTTGTTTATATCCTCCTCTTGAAATGTACTTTTCTTTATGCT
CAGCGGCATTACTCTCTAACTACATAGATCTCCTCGCTACAGTCTACTTTCTCAGTGTAAAGGGCC
ATTTCCTCTTGTATTTGGAATTCTCCTCTTGTATCTCGTTCCTTAGATAACAGTTTCACTCTTCCTTT
CACATCTGTTCCTATCTCTCTCCTATTTCTTTATTTTGTTCGTTGCTTGAGAATTGTATCAGCAACCT
AAGGTTGAGTTTGACATTACTGTTTACTCTCATTTTACAGGGGAAATCCAAATCTGAACATGTCAC

ACAATTGTCATCAGGTTCCTCTCCTGTTTTAGTCAGAGTCATAGCTCGTATACTACTCACAACCC
AGGAACAGCATCCATGTTTGGTGATTCATGTGAACCTAATCAGAAATTTTCTAGTTCTGGGTCTTT
AGAAGATACATCCGAAGCACAAGCCTTGCGCCAATTGGAAGAACAGCTGAGTTTGAATGAGGA
CATTTTCAATGAAATTGCACTTGATTTAATCCCAGGTCAAGATCAAAGGGTGGTTTATAAGCAAG
ATAACTCTGTAGCTTTGTCTGGACCAAATGATCCAGGACAACCTTGTGATGGATATAATGGAAGA
GAAGGTAATTTAGACAAACTTGAGCTGTTAATGTTTTTTTTCGAGGGGGGAATAAAATTAATATA
TGTTTGAAGTTAGTGAAACATTTGAATTATTATTCTATTTTATGCTTTTATCTTGTATTGTGTTTTGA
TTTGTTCAGTTATAGATGCTTGTCTTCTACTTATGCAACATATGAAGATGGCTTCTTCAGCCCTCAC
AGCTAATTTGCCGTGACTGTTGAGTTAAATTTATAACATAACTGCTTATTTTAAGTTGTCCAAGGC
TATCATAGCTATATATATATCAGTATGACCATACTTCTATTCTTCTAATTTGTCTATGGTACCCTTCTG
TATGATAGATTTTCTTTAAGTGAAACCTAATTCCCCAATTGTTATATGCTGGTTGTTTCATTTGAAT
CTATCATCCACTCAACCTTTTTTTTTCAAACTGTCACATTCAGCTTTATATCAGACTCAAATTTATT
TCATAAGATCTTTATAGTTTGTTTTCGCTACTCCTTATTATAATTAAACAACAACTGTAGAGAGAG
GAAAGCAAAATCTTCTGAAATTATTGTATTTTCTGTAGATGACAGTGGTACATATTATCATGACTT
TCTTGATGATTGTCCTGGTGGAAATGAAAAAACTATATACTGGACTGAAGTGCTGGAATCGTGTA
AGCCCTTATCTGTGACCAAATTACCAGATCAACATGCATATGACGCGATTGAAAATGTGAGTATT
CCTAAGATATACTTCATGTCATCAAGAGTTTATAATTCATTTTTCTTTTGCAACTTGCAAAGAAAT
AAAATCTCTATGCTTAAAACAGGGAAAGTCATTATTTTCTTCAGGAAGAGGAATGATTGCCAACC
GGGAAAAAAATCAGTGGCTAAACTCCAACAGTAATAATGTTGAAAACTGTATGACTTACTTCTG
CCTTAACTTGAAAATTTCCCTTTTTGTTGCATATAATGAAATTTCTTCCATCTCTCTTTGATGTTCCA
TATCACCAGCTGTTTTCTTGTTTCCTCAAGACATTGGAGTCAAATTTCCTCCATATTCTATGGTAGA
AACTCCAGGAACTAACTATGACTACTATGAAACCTGTTTTGATCAATTCCAAAATCAAGAACCTC
TGGGTGTAGATTCAAGCTTCACTGTTGTGCAGAAACAGAAATTTACAATTAGGGCAGTTTCCCCA
GAATACTGTTATGCCACTGAGACTACAAAGGTTTGGGCCTTTTAATTTCAACACTTGTTATGTCCT
ATGTAATGATTTATCACTCTTTGCACTAGGGAATTTATCTGCTGCTTCTTGGTGCTTATTACTCAGC
TTTACCTATGTTCTTGAAGAGCTTTAAAATGCATACACAACATGGTTCAAATATGTATGCATTTTT
TGTATAATGTAATTATATATGTAATAACTAGTTATATATATAATAACTAGGGAGTTAAAAATGAA
AGCTCTTAGCTTTTGATTTCAATACCATTTGGGGCATATACATTGTTTCTACTCTCTTTCCATTTGA
AATTTTAATATTTTATATGATACAAATACTGTTGATGTTGTCTATATTTGAATCCTAATTTCCCAAG
TTATATGCATAGGTGATCATTATTGGGTCATTTCTATGCCATGACTCGGATTCTACCTGGGCCTGT
ATGTTTGGTGATGTTGAAGTTCCTGCAGAGATAATTCAGGATGGTGTAATCTGTTGTGAAGCTCCA
TCTTATCTTCTTGGGAAGGTTAATTTGTGTGTTACTTCAGGAAATAGGGTGCCATGCAGTGAAGTG
AGGGGGTTTGAGTTTCGAAATAAGACTACTAGTTGCACTCGCTGTAATTCATTGGAAACAGAAGG
CAGCAAAAGTCTAGAAGATCTGTTATTACTTGTTCGATTTGCAGAGATGCTCCTTTCTGCTTCAAC
TACAAAGGATGACAGAATAGAATCTGGAAGTTATCTTTCAACAGAACAGAAAGATGATGATGAT
TCATGGAGCCATATTATTATAGACACTCTTCTAGATGGCACCAGAACATCATCTGATACTGTTAA
CTGGCTTCTTGAAGAGCTGCTGAAGGATAAGTTGCAGCTCTGGCTTTCTAACCGAAGAGATGAAG
GGACAGGCTGTTCTTTTTCCAGGAAAGAACAGGGGATAATACACATGATTTCTGGGTTGGGTTTT
GAGTGGGCCTTGAGCCCCATTCTTAGTTGTGGTGTGAATATAAATTTCCGTGACATCAATGGGTG
GACCGCTCTTCATTGGGCTGCTAGGTTTGGGAGGTAATAGAAAACTGAGTTGTTGATATTGATAT
GCAATTGTGATAGCATTTTGTTTGAAAGCCTTGAATAATATACTTGATAAAATCTGTTTAGAAACC
TTTACAAATCCTTGGGAGAAGGGAATACCAATGCAAAGAAATAGAACAAGAAGGACAAGTAAA
GCCTTTAATACCTACCCAGCAAACATTAGAAATTGATGGCTGCATTTAAGGAGTAATAACCACAT
CTAACAGTATGCTTCACATGCAACAGGATGTTTCAATCATTCTTGAACATGTATATAAAGCCTAA
TCAATCAAAGCCAAATGTTTAGTTTTTTATTTGAACAAAAATGCCTTTTTCCCACCATATGGGATC
AGCTACATTAATTAAATTATGACATTGTGTTGGACCAAAAACTGAATTTTAAGAAATGTCATTTA
AATCAAAATGTTTTTAAACGGTTTCTCTGGAGTTCTCGTTGGTCTTCCGCTATCTCTATTGATCAGA
CTGCCCTCCATATAATTAATTCGCCTTACCTAGGCCTCTATTGGTCTTCTCACATGATCAAACAAT

CTTATTAGACCAAATTCTGTCATAAATGATCATATCAAGGTTGAAACTCTTGCTTTTAAAAGCCTT
TATAAATGATCTATCCATTCTAATTGTTCTGGGAATTTGGGCCACTGAAATATTTTGTTTCATGATA
GGGAAAAAATGGTTGCTTCGCTTATAGCTTCTGGTGCATCTGCTGGAGCAGTGACAGATCCAAGT
TCACAAGATCCAACAGGTAAAACTGCTGCATCTATTGCAGCTAGCCATGGCCATAAGGGACTGG
CAGGTTATCTTTCAGAGGTAGACCTAACAAGCCATCTGTCATCCCTCACATTGGAAGAGAGTGAG
CTTTCTAAAGGATCTTCTGAGCTTGAAGCCGAGTTAACTGTCAGTAGTGTCTCTAAAGAAAATCTT
GTGGCCAGTGAGGATCAGGTTTCACTACAAGCTTTCCTGGATGCTGTTAGAAATGCAGCTCAGGC
AGCTGCACGGATACAAGCTGCTTTTCGTGCACATTCTTTTAGAAAACGGAAAGAAAGAGAAGCT
GCTGCTGACGCTGGTCTAGATGGATATTGTATTGATGCAGGTAGCATTGATAATAACATTTCAGT
GCTTTCTGCCGTGTCAAAACTTAGTTCTCAGAGCTGTCGTGATTACAATTTAGCTGCCTTATCGATT
CAGAAGAAATATCGAGGCTGGAAAGGTCGTAAAGAATTCTTAGCATTGCGCCAGAAAGTAGTTA
AGATACAGGTAACCGTAATAGAAATTGTTAATACAGTTTTACTATACTAATTGAATTCCAAGAGT
TCTGCATGTACCTGTATATTGGCAGTGTAGAAGTGTAGGTACTTTGTAAAGTGAATAAATAATAA
ATTTTTTTATAAATTGGAATGAGGGGGGAGATAAAAGATAGCTAGACATGCACATGCATACATC
AAGAACTTACTTTTAACTATTTACTAGTATTAACCTTGGTTTTTCTAAATTTATTTGGGCAGGCTTG
TGTAAGGGGATACCAGGTTCGGAAGCAATACAAGTTAATATTATGGGCAGTTGGAATCTTGGAC
AAGGTTGTGCTACGATGGCGCAGAAAACGAATTGGTATACGAAGTGTCCGGCAAGAAATGGAAT
CAAATGAAGAAGAAAGTGATGATGAAGATTTTCTCAGCGTGTTCCGGAAAGAGAAAGTAAATGC
AGCAATTGAAAAGGCTTTGAAGCAGGTGCTTTCCATGGTCCATTCCTCTGGTGCTCGTCAGCAAT
ATAGGCGCTTGCTTTTGTTGTATCGTCAAGCCAAGGCCAAGGTAAATACGCTAATAAAAGCAAA
AAATAAATTATAGGTCTGGGATAACACCGGTTAACATCTCTGGTATCTGTTAGTTATACCACTTTT
TGCATTCAGATTGATCAAATAAGAAGTAAGTGTTGAGCATAGCTTTCATAGTCCATTTCTAATTTT
CTTTACTGAGTAAGATAACTGTAACGCTAGCAAGTAGCAACACGATATTCTGTTTGGACACACTA
TTTATTACTAGATGAAACCCAATTCTAGTTTCTCATAAAATGTTGAAAATCAGAATATAATCTGAT
CCCATTCTTTATTAGTGGGGCCAGCAGCATAAGGTTTTACTCAACAATAAAAGTGTCAAATAGTG
GGTTGCTAATCATAAAGTAATTAGGACCTAATTAAACATGCTAAGCTAGCATTTAGCAATGTTTTT
TATGTGAGGAGAGGTTCCCTGTTTTGTACAATGCATAACTTGTTGATTCTTTTACAGACTGAACGT
GGCAGCACGAGTGATGAAGCACCCTTATCAACTTCGGAAGAGGAAGTTTCCAATATGGAAGATG
ATGATTTGTGTCAATTTTGGGAAACGTTCTGGCCTTCCTAGTTTTATTTCTCTTTGGGGATAAATTT
CTGACTTCCCAGCTTATATGGTGTGAGTATTTGGATGTGTAGAATAAGCTTGATTGAGCTTTTAGT
TTGTAAATCAGTCTAGGTTGTATAGTGATAATGGCGATGGACACTTTCTCTTTTTATGTAAAACTG
GCCTTCAATGCTCTGGTGGTATATAGGGTTTCAAATACAGTGAAGTTTTTATTTCTTATTTCTTGTC
TTACATTTCATCAGAAGAACTAGACAGAAGAAACCACAAAGATAAAAATGTTATATTATTAAGA
TATTAATC

>Glyma.17G031900 (GmCAMTA12)

AATAATTTAACGCATAAGAGTCAGACTGTAGAATAGAAAACAGAAAGGAACAGGAAAGAGTGT
CATTCAGCTCCAACGAGGCGATCATATTTCTCTGCAATCCGAACAGACCCACCCTTCTCTGCAAT
TAGGTAAATTTACCACCTTTTCTATTTTTTCTCACTACTCTTCTAATTTTTCACCCTGTTTTTTATTTT
ATTTTTAGTTGAGAACGATGTTTTTTTCTTTTATCGAGTCACCACTTTGCTCTTTTTTTCATTTTTCAT
GTAATATGCAGTTGCTGAGTTTTAGGTGAGAGAAAAGTGTTCGGAATTTCCCAAGGTTTCTGTGA
ATATGGCGAATAACTTAGCGGCGCAACAACTGGTTGGTTCAGAGATACATGGATTCCACACATTG
CAAGGCACGTACTTCACTGTTGATTAAGTGTCTAACACTGAGATGTAGTTCTTGTTTCTTAGTTAA
TAATTATCGATGATGGTAATTTATATTAATGTTGTGAGTAGTACTAGATGTTCAGACACAATGTCC
TGATATTTAATTGGAACTAAAATGATTTTACATAAGTTGTTCCCTCGCTTTGATAAATTATCTGAT
AAGCCTCCTATTGTATGGATGATAATTTTTGTCATCTGGTGGGAGACATCAGCCCTTTAAGTGGAT
TTTAGAACCTCGAGAGATTAGTCTTTGGTTTTCGGCTCAAGGATACCCCGTGTTAACAAAAAAAA

AAATGTTAGTTTTTGTTAGTCAAAAGGAAAGGAAATTAATGAAAGCACGTTCTCTGTTTATTTGTT
GTTCTGACATGTTTTCTTATATATGCCCCAGATTTGGATGTGGGGAGCATTATGGAGGAAGCAAG
AACCAGATGGCTCCGTCCCAATGAGATTCATGCCATGCTTTGTAATTACAAGTATTTTACCATTAA
TGTCAAGCCAGTGAATTTGCCAAAAAGTAATTATCAATTAGCTGTAACTTGATGTTTGTGATCATA
TCCATTCTGCTCTATCGTGCATTTTTTTATAGACAACGTGTCATCTCATCATATTCATTTTGTGACTT
TGTCTGCATACCTTAGTTTCCATTAATTTTTGGAGCTTTTGTGATCTAGCTTGTTTTATAATTTGTGA
GCTTCTACACCCTTTATTGTTGGTTTAAAATTTATTTAGTTTTAAATTTCTATTCTTAGGAGGATCTA
TTTTCCTCTAATCTCTTTGTAATCTTCTGCTTTTACTTTAATGATTTTCTTCTTTCATAAAAAAATGGA
CCATTTATTTGTATATATCATGAATAAAGCAGTATAAGGTTTTACTGTGAGTGACTTTTCATTCACT
TATCTAATAGAACTTTGATTAGTTATTTTGCATACAAATAAGTTTTCACTATTAATGATTAGACAA
TGTCAAATATCTGGTGAAAATACTGTTGTTGTTGTTGTTATGGTTATTTTGTGTGTCAACAGGTGGT
ACTATTGTCTTATTTGATCGCAAGATGCTTAGGAACTTCCGTAAAGATGGGCACAACTGGAAAAA
GAAAAAGGATGGGAAAACCGTTAAAGAAGCTCATGAGCACCTAAAAGTGAGGATTTTCATTTTC
TTGGGTGCTTTTATCCGTGATAATTTTTTTATTTTAATTTTGTTTCTTTAACATATTGTAGATGGGAT
AATTTATCATTTTTTGTAATGTGGAGTATGAATGAATTAAACACTAACAGAGATGAGAGGGTATA
GCAAGTAGCCTGACAAGCATTAGATGTGGTTAAATTTTGAAAGACATAAGGCTTCTTGGAGCTCA
GTTTTCTATTCTACTGATTTTATTGATGTTGTTCAATTCATTGTGCTTTCTCTATAAATAGTACTTTTT
TCTTTTAGGAGAGTTTTGGTGTTTAGTGAGTTTATTAGATATTGACCTCTTCTAGATCTGTAAGTGC
TGAAGTATTTAACTGGTGATGCTTACTTTATATAAGGCATTATCTTGGATTTGTATGTCATATGATT
TTTGTACAATGATCCAGTCACATATCTATCATTGTTTCTGGGTAGTATTTGAGAAAGAGATCAGTT
TATGGCTTTCTGCTTTAATTTTATCACCAAGTTAAGTGTTTATTCTGTATGATCATAATTCCTTCCTC
CAGTTTTTGTTATTTTTTATTCATTAGCATCATATCTGATTTTTGACATTCTTTCTGGTCATTTTTTTC
TTTTCCCCATTCATCAGGTTGGTAATGAAGAAAGGATCCATGTATATTATGCACATGGCCAGGAT
AACCCAAATTTTGTCCGAAGATGTTATTGGCTGCTTGATAAGTATGCATATCAACTGTTTCTTAAA
GCTCCCATTCCAAAATTTTATGCATGTTCATACTGCTGAATTATTTTGTAGTATGGTGCTTCTGTTA
TTCTTGTATATATTGGTTAAAGCATAAAATTAAAATAAGTTTTCTAATGACAATGTGACTTAAAAA
TTGTTTTCACTATTTTCTTTTCAGGAGTATGGAACACATAGTCCTTGTTCATTACCGTGAAATACAG
GAGGTTGTGTTCAGCTGCTCACCATTGCCTCTATTTTTTTTCCTTTTAAAAGTTCAATTTAATGTCA
CAAACCATGATTTGTGTGTCATGTGTATGTTGTTTTGTCTGGAAGAAAGACTATATACATATATAG
TCATTAGTCACTTTGATTTACTCTTTATGACACTTTAAATATGAGTTTTATTTCTAATTATAACCATT
CACTGTTAACTGCTAAGTGCTAGGATTTTAGAATGCACCTTAAATATGACACTTAGCAGTTAAAA
TCTCTTTATAATTAGAAATAAACTTCATGTTTAAGTTGCCATTTTTGCTCTAAAGTTTTATTGTGAA
AATAGATATAACTGGCTTTGTGACATTCTTTGGGTTGTTGTTTATGATACAGATGCAAGGTTCTCC
CGTCACTCCTGTAAATTCACACTCCAGTTCTGTCTCTGATCCTCCTGCACCATGGATTTTATCAGA
AGAAATAGATTCAGGAACCACAACTGCATATGCTGGTGGTGAGAAATAACTTTTATATTTCTGTG
GTCTATCTTCTTAAAAGTTGTACTGCTGTAAGCTATTAATATGAAAGGCTAATGAGTAAGACATTT
GTTTAAAATACTTATATTTTAATTTTGAAGATGTTAAGTGTTTGTTTGGAAACAATGAGAGGCAGG
ATAATGAGTTGCAAGAAAAGAACACGTGCTATGTGCATGTCTTTATATATTGCAGATATATTCCTT
CTACCTCATTTCTGCTCATCTTTCCAAAGGAAGCATAATATATTTTTTTACCAAGGGAATAGTTGG
TTGAATGAGGTGCATGAGTTGTTATAAATCCCATGGTACATGTCTTTGATTCCTATGGATAAAAAA
AAAAGATTTTTTTTTTGTTGGCTTATAATGCATGCTGTGTATGAAAAAAATAAAAATGATTAAATA
AACTATGACTGTAGTTGTCTTGATAAGGTATATATCTCATCCTTTCTGATTTGGACAAAAACAGAC
ACGAGTGCCAATATAAATGTCAAAAGTCATGAACTCAGACTTCATGAGATTAATACACTTGATTG
GGATGACCTGGTGGATGCAAATGATCATAACACAACTACTGTGCCTAATGGAGGTAAATGGTAG
TTCATCAAAGCATAATCAGTCACTTGTCTGGATTGCACCATATATTTTATTATAACTTTAAATTAA
CTTTTTCCAGGTACTGTTCCATATTTTGATCTACAAGATCAAATTTTACTGAATGACAGTTTCAGCA
ATGTAAGATAATATTATCTTTCTTTTATTTGTATTCATGACTGTTTTTTGTTGTCTTTTTCTCATTTGA
GTTCTGTGATGGACAGTTAGTTCTTTGACATTTTTCACATGGGAAAGAAAATTTATTAGAGAGAA

AATGAGTGAAGCACTGGAGAATAACTAGGGGGAGCATAAGATATCCTCTTTTCTAATACTAAAA
ATGGAAAGGTCAAGAAAGGAGAAAGATTCCAAGATGCAGCAAAAGTCCGTTCAATCTTTCCATG
TTGATAGTGAAAACTATCATGAAAAAGCCATGAGCTATTAATCACCAGAGAGAAGCCAAAAGTA
CAAACTTATCCTAAAGCAAACCAAGGGACCAAGAGATCCTAAAAATGCAGGCATTCTGCTTCAA
CCAAACAGGCCAAAGAACTGCAAAAACTGCATTGTCATAAAACTTTTGCCTCCTGATTGTTGGTC
CTTTGAAATTTATCATAAGACCATTTTATCCTATTTAAAGTATATGTTATAGAGATTTTATTATATT
TCCTAGACAACATGTTTTATAGATGTTCAGGCATTCAGCATCCAGGACTTCGGTTTTCACTATTAG
ATTATTGTTTAATTATACATGATTAGGAGTTAAAATGTCATTTTACCTTTGTGATACTATTTTTCAA
ATCACCTGCAATCACATCTAAAAAAGGCCACAGCCATTTTGAAGAACATGCCTTGAAATTATAGT
GAAGGAAATGCTTATTTTTACTTTTTATACCTCATATTTGTATCATTTCCTTTGTTTCCTCTTCCCTCT
TCTCCCTTTTTTTTTTCGTTCTTTTTTCTGGAGATACTAACTTTGTAATAGCCACTGCTTTGTTATACT
TGCTTTGTCACCAGGTAGCCAACAATCTATCTGCAGACATTCCTTCTTTTGGTAGTCTCACTCAGC
CTATTGCTGGGAGCAACAGTGTTCCTTACAATTTTTCAAGTGTTAATCTTCAGACGATGGACGATC
AAGCAAATCCACATGAGCAGAGGAATAATACAGTTTCCTTAAGTGGTGTTGATTCCTTGGACACT
TTGGTCAATGATAGATTGCAAAGTCAGAACAGCTTTGGAATGTGGGTAAACCCCATCATGTCTGA
TTCACCATGTTCAGTAGATGACCCAGCTCTTGAATCCCCAGTTTCATCCGTTCATGAACCATATTC
ATCTCTGATAGTGGATAGTCAGCAATCTTCTCTGCCAGGACAAGTATTTACTATCACTGATGTATC
CCCTACATGCGTATCTTCCACTGAGAAATCAAAGGTTTCCTTTGCCCTTCTTGTAGATCTACTTGCT
ATTATTTTCAGTTTTAAGCATATCTTTCCTTAATGTTTTATTTAGTTTCTTACTTCTTAGTTGATTCCT
TAGTCTTTTCTTTCAATCTTGATTGATTGAGTTCCTGTTGAATTCCATAGCTGTAGACTGCAGAAAT
ATAAACTCCGAGTATTATGAATTATTAGTTCTTTTCAATCTAGCCAAATTCATTTGTAATTGGTTAT
GAATTGCTCTTTGGCTGCAAGCATGCACATCTTCATGTATACAAATTATAACAGAATTTAACATA
GTACATACTTGCTGCTTGATGAATTCATATGTTTGCTACCTGTTATTTTGACTTTCTGAATTCCTTTA
CTTGCAGGTCCTGGTGACTGGATTTTTTCATAAGGACTATATGCACCTTTCAAAGTCCAATCTATT
ATGTGTCTGTGGTGATGTTAGTGTTCCTGCAGAAATTGTTCAGGTTGGGGTCTACCGGTGCTGGGT
ATCACCACATTCTCCAGGATTCGTGAATCTCTACATGAGCATTGATGGCCATAAGCCTATTAGCC
AAGTTGTTAATTTTGAATATCGTACTCCTGCTTTGCATGATCCCGCCGTTTCAATGGAAGAGAGTG
ACAATTGGGATGAGTTTCAACTACAAATGAGGCTTGCATATTTGCTCTTTAAACAATTGAACCTTG
ATGTTATCTCCACTAAAGTATCACCCAATAGACTTAAAGAGGCTAGACAATTCGCCCTCAAAACA
TCATTTATTTCCAATAGTTGGCAATACTTAATCAAGTCAACTGAAGACAACCAAATTCCATTTTCA
CAAGCAAAAGATGCTCTATTCGGAATTGCATTGAAAAGCAGACTAAAGGAATGGCTTTTGGAAA
GAATTGTTTTAGGCTGCAAGACTACTGAATATGATGCTCATGGCCAAAGTGTAATCCATTTGTGTG
CAATTTTGGGATATACTTGGGCTGTTTCCTTGTTTTCATGGTCAGGCTTGTCATTGGATTTCCGTGA
TAGGTCTGGATGGACAGCTCTTCATTGGGCAGCATATTGTGGAAGGTATAATACACAGACATTCC
ACTTGTTTAAACCATTAGCTCTTCTGTAACTACATTGACCTAAAAATCCTTTTTTGGCGTGGTTTCA
CCATTCACATTCTTTTCTGTTTTTTTTTCCTTCATACACTTCACATTAACATAATCAATGTGAATTTA
GCATGATTTTGGGTAAAGTAGGCTGCTTGAACTTTGAATTGAAATTTTCTTATACATTTTGATTTAA
AATTTGTATCTTCAGGGAGAAAATGGTTGCAACTCTGTTGTCTGCTGGGGCGAAGCCAAATTTGG
TCACTGATCCTACTCCCCAAAATCCTGGTGGATGCACTGCTGCTGATCTTGCTTATATGAGGGGTC
ATGATGGCTTAGCAGCTTATCTTTCAGAAAAATCTCTGGTTCAGCACTTCAATGACATGAGCTTAG
CTGGAAATATCAGCGGCTCACTTGAAACTAGCACGACTGATCCGGTAATTTCTGCAAACCTTACG
GAGGACCAGCAGAACCTGAAGGATACATTGGCAGCTTACCGAACTGCTGCTGAGGCAGCTTCCC
GCATACATGCTGCATTTAGGGAACACTCTCTAAAACTGAGAACTAAGGCGGTTGCATCTTCCCAT
CCAGAGGCTCAAGCACGCAAAATAGTTGCAGCAATGAAGATTCAGCATGCCTTTAGGAACCACA
AGACAAAGAAAGTGATGGCAGCTGCTGCTCGCATACAGTGTACATACCGTACATGGAAGATTCG
TAAAGAATTTCTCAACATGCGTTGTCAGGCTGTTAAAATTCAAGTAAAAGCTTCTCTTCTAGTTGT
CACATACATTAACTGCAGATTTTGTTGGGCAAATTGACTAACATCTATGTTAAAATGCAGGCTGC
GTTCCGGTGCTTTCAAGTGCGGAAGCATTATTGCAAAATTCTTTGGTCTGTTGGTGTCGTTGAGAA

AGCAGTGCTGCGTTGGCGTTTGAAGAGAAGGGGTTTCCGTGGGCTCCAGGTCAAAACTGTCGAA
GCGGGAACTGGAGATCAGGATCAGCAAAGTGATGTAGAGGAGGAATTCTTCCGGGCTGGTAGG
AAACAAGCTGAAGAACGTGTTGAGAGATCTGTGGTACGTGTGCAGGCTATGTTCCGTTCGAAGA
AAGCACAAGAAGAGTACAGGAGGATGAAATTGGCCCTTGATCAAGCAAAGGTGACAGTTAATC
TATACATTTTATTAATCCTAAGAGGATTTGGTCTTGCATGTTTTTTCACTTCTAGAATACTCCTTTGT
GCAGCTTGAGCGAGAATTCGAACGACTTCTTAGTACTGAAGTTGACATGCAACTGAAGACATAG
ATTACCCAAAAAGATTCTAAATTTTGGGATGCAGTATCCAGTAGATTTTGTAAGTCCCCCTTGACT
CTTTCAGACTTTCTTAGTATCCTAGAATGGGTCATTATATAAAGTTTTCATCTGTCATTGGGCTCTC
TATGGTTGTAACTTCTGATGCAAAGATACTGTGCCACCCCAAAGTTTAATATACAGAATACTAGA
ATAGTTATAGCTATAAGCCTGGTCTGGGGATGGTAATGGCTTCTTCGTTTCTTTTAACTCAAAAAT
AGCAGAGGCAACCAAAGATCAGTGCGATTTTTTTTATTATTATTTATAGGCGCCAACACATCTTC
AATATTAGCCCATATTTACAAAGATACTCTTTGTTTCTTAAATCACCATATCCAATATCAGTTATC
TCTCTAGATTAATGATAAAATTTATTATATGATACTCCCCAATATTATGTTAGTTAAAATTTTAAAT
CCAAATTCAAAACAAATTGTAGCAATATCAAAGAGCACGAGATAATCCAATGTCATTTCATTGGT
GTTATTTTTATGGACACGGTTATAATTCTTAAGACTATAGGGTTAGATAGGGATGAGTAAAGTTTG
GTCACAATTTTAGTGGCTTTGTTTCTATATGAAGCTGCCTTGGTAGTGCGTGCTTCC

>Glyma.07G242000 (GmCAMTA13)

GTAAGAGTCAGACTGTAGAATAGAAAACAGAAAGGAACAAGAAAGAGTGTCATTCAGCTCCAA
CGAGGCGATCATATTTCTATCGGTTCCAATACGAACAGACCCACCCTTCTCTGCAATTAGGTAAA
CTTACCACCTTTTTCTATTTTTTCTCACTACCCTTTTAGTTTCTCATCCTGTTTCTGATTTCTTTTAGTT
GAGAACGATGATTGTTTTCTTTTATTGAGTCGTCACTTTGCTCTTGTTTCATTTTTCATGTAATACGC
ACTTGCTGAGTTTTAGGTGGGAGAAAAGTTTTCGGAATTTCCGAAGGTTTCTGTGAATATGGCGA
ATAACTTAGCGGTGCAACTGGTTGGTTCAGAGATGCATGGATTCCACACATTGCAAGGCACGTAC
TTCACTGTTGGTCTTTGAATTATGCCTATGTTTATTTTGATCAAATGACTAAGATGCAGTTTTTTTTT
TTTCCTTATCGATAGGGTAATTTATATTAATGTTGTGAGTAGTACTAGATGTTCAAACACAATGTC
CTGATATTTAATTGGAACCAAAATGATTTTACATAAGATGGTCCCTAGGTTTGATAAATTATCTGA
TAAGCCTACTATTGTATGGATGATAATTTTTGTCATCTGGTGGGAGAGGTCAGCCCTTTAAGTGGA
TCATAGAACCTTGTGGGATTAGTCTTTGGTTTTTGGCTGGAGGATACCCCGTGTTTACAAAAAAAA
AAAAAAATAGTTTTTGTTAGGCTATGTCAAAGGGAAAGGAAACTAATGAAAGAATTAACCTTCT
CTGTTTATTTGTTGTTCTGACATGTTTTCTTATATATGCCCCAGATTTGGATGTGGGGAGCATTATG
GAGGAAGCAAGAACCAGATGGCTCCGTCCCAATGAGATTCATGCCATGCTCTGTAATTACAAGT
ATTTTACCATTAATGTCAAGCCAGTGAATTTGCCAAAAAGTAATTATCATTTAGCTGCAACTTGAT
GTTCGTGAATGGATATGCATCATATCCATTCTGCTCTATCGTGCATTTTCTTATAGACAATGTGTCA
TCTCATCATATTCATTTGTGTCTGTTATGTCTGCATATCTTAATTTCCATAAACTTTTGGAGCTTTTG
TGATCTAGCTTGTTTTATAAATTGTGAGCTTCTGTGCCCTTTATTGCTGGTTTAAAATTTATTTAGTT
TGAAGTTTCTATTCTTAGGAGGGATCTATTTTCCTCTAATCTCTTTGTAATCTTCTGCTTTTACTTTA
ATGATTTTCTTATTTCATAAAAAAATGCACCATTTATTTGTATATATCATGAATAAAGCAGGATAA
GGTCTCACTGCGAGTGACTTTTCATTCATGGAGCTAATAGAATTTTGATTAGTTATTTTGCATCCA
AATAAGTTTTCACTATTAATGATTAGACAATGTCAAGTATCTGGTGAAAATATTGTTGTTGTTATA
ATTATTGTTATTTTGTGTGTCAACAGGTGGTACTATTGTCTTATTTGACCGCAAGATGCTTAGGAA
CTTTCGAAAAGATGGGCACAACTGGAAAAAGAAAAAGGATGGAAAACTGTTAAAGAAGCTCA
TGAGCACTTAAAAGTGAGGATTTTCATTTTCTTGTTTGTTTTTTTTGTGTTAATTTTTTATTTTATTTT
AATTTAGTTTCTTTAACATATTGTAGATAGGATAATTTATCATTTTTTGTTATGTGGAATATGAATG
AATTAAACACTGACACAGAGATGAGAGGGTATAGCAAGTAGCCTGACAAGCATTACATGTGGTT
AAATCTTGAAAGACATAAGGCTTCTTGGAGCTCAGTTTTCTATTTTGCCGATTTTATTGATGTTGTT
CAATTCATTGTGCTTTCTCTATAAATAGTATTTTTTTTTTCTTTTAGGAGAGTTTTGGTGTTTAGTGG

GTTTAGATATTGTCCTCTGCTAGATCTGTAAGTGCTGAAGTATTTAACTGGTGATGTTTAATTTATA
TAAGGCATTATCTTGGATTTGTATGTCATATACGATTTTTGTACAATGATCGAGTCACATATCTAT
CATTATTTCTGGGTAGTATTTGGGAAACAGATCAGTTTGTGGCTTTCTGCTTTTAATTTTATCATCA
AGTTAAGTGTTTATTCTGTATGATCATATAATTCCTTCCTCCAGATTTTGTTATTCTTGATTCATTAG
CATCATATCTGATTTTTGACATTCTTTCTGGTCATTTTTTTTTCTTTTCCCCATCCATCAGGTTGGTA
ATGAAGAAAGGATCCATGTATATTATGCACATGGCCAGGATAACCCAAATTTTGTCCGAAGATGT
TATTGGCTGCTTGATAAGTATGCATGTCAGCTGTTTCTTAAAGCTCCAATTCCAAAATTTTATGCA
TGCTCATACTGCTGAATTATGTTGTAGTATGGTGCTTCTGTTATTCTTGTATATATTGGTTAAAGTA
TAAATTAAAATAAGTTTGCTACTTGCTAATAACAATGTGACTTAAAATTGTTTTCACTATTTTCTTT
TCAGGAGTATGGAACACATAGTCCTTGTTCATTACCGTGAAACACAGGAGGTTGTTTTCAGTTGTT
CACCATTTCCTCTATTTTTTTTCCCTTTCAAAAGTTCAATTTAACGTCACCAACCATGATTTATGTG
TGTCATGCGTATGTTGTTTTGTCTGGAAGAAAGACCATATACATACATAGTCATTAGTCAGTTTGT
GATTTACTGATTTTTAGCCTTTTGAACATGTCTGGATACGGCATCATTATGTCTATAAGAAAATTTT
CATACAGTGAAAATGTTTTTTCAGTTGGTCTGGATGAAAACTTCATTTGTTTTCTGTGTATAGTGCA
CAGGCTCTTTCATTTTGGGTATTGTGAAGCAGCTGTGTATAGTGTTGTTTTCCATTATGTGTTTGAA
AGGATATCCATGCCCAACAAATGGATTTATACCAGTTTTGATGATGGATATAAGCTCCAAAAGTT
GATTGTAGGACAGGGGAAAACCATTTTTTGTTTCAAGTCCATTGGTTTTGTCTTATTTCCTATGTTG
GACGGATATGCTGTGGAATGGCAAAATATTAACAGAGCCATCATTAGTAGGAGCATGACGGCTT
ATTAGCTTGCATTAAAAATTGGCCAAGTACAGCATTATAGATTCTATTGTATAAAACCCCTTTTTT
TTAATGAGCTGTTAATGTGAATTGGTTCTTTAAAAGTCGACAGTTTGTTATTGTGCTGCTTTGCTTA
AATATACTCCAGTTTCTTTTTACTTAATATTTGCCTAGAGTTGGAGTCTAATTTTCTCTATTGTATTT
TTTTCAATTAAAGGGGTAACCCTGTGGCTTTTGTGGCAGAGGTGACCCATGCCATATCCCTGGAA
AAAATTTAAGTTTCAAAGAACCCAAGGTTGTTTTCTTGCAATGATATTAAAAGTGTTTGCTAATAT
AGAAGTTATATCCCACTCATTGTTTCATTATGTTGAAAAGTACATTTATTTCTCCTTCCAGTCTTCT
TTTAGCATTTTAGAATAAAGTTCATTTTTTTTTCTCTCTATAAACATAAAAAGTAGTTTTCAAAAAA
TGCTTGTCAGTGCTAGGCTTTAATCACTTAGTTTCTCTTCATGAATGACACTTAGCAGTTAAAACA
TCTTTATAATTAGATATAAACTCATGTTTAAGGTGACATTTGTGCTCTAAAATTTGATTGTGAAAA
TGGATATAAATTGGCTTTGTGACATTCTTTGGGTTGTTGTTTATGATACAGATGCAAGGTTCTCCTG
TCACTCCTGTAAATTCACACTCCAGTTCTGTCTCTGATCCTCCTGCACCGTGGATTTTATCAGAAG
AAATAGATTCAGGAACCACAACTGCATATACTGGTGGTGAGAAATAATTTTTATATTTGTGGTCT
ATCTTCTTAAAAGTTGTACTGGTGTAAGCTATTAATATGTAAGGCTAATGAATAATGACATTTGTT
TAAAATATTCATATTTTAATTTTGAAGATGTTAAGTGTTTGTTTGGAAACAGTGAGAGGCAGGATA
ATGAGTTGCAAGAAAAGAACATGTGTCATGTGCATGTCTTTATATATTGCAGATATATTCCTTCTA
CCTCATTTCTGCTCATCTTTCCAAAGGAAGCAGAAAATAATATATTTTTTTTACCAAGGGAGTCTA
GCTCAGTTAGTTGAGTAGGGTGCGTGAGTTGTTATGAATTCCATGGTACCCATATTCGATTCCTAT
GGATAAAAAAAAAATAAGAATTTTTTTGTTGGTTTATAATGCTGTGTATATCTGGTTGTTTAGTTGA
TGCCTTTATTTTATGAAAAAAATAAAAACGACTAAATAAACTATTACTGTAGTTGTCTTTACTTGA
TAAGGTGTATAATTTCAGCCTTTCTGATTTGGACAAAAACAGACATGAGTAACAATATAAATGTC
AAAAGTCATGAACTCAGACTTCATGAGATTAATACACTTGAATGGGATGACCTAGTGGACACAA
ATGATCATAACGCCTCTACTGTGCCTAATGGAGGTAAATGGTAGTTGATTAAAGCACAATCAGTC
ACTTGTCTGGATTGCACCATATATTTTATTATAACTTAAAATTAACTTTTTGCAGGCACTGTTCCAT
ATTTTGATCAACAAGATCAAATTTTACTGAATGACAGTTTCGGCAATGTAAGATAATATTATCTTT
CTTTTATTTGTATTCATGACTCTTTTTGTTTTCTTTTTCTCATTTGAGTTCTGTGATGGACAGTTAGTT
CTTTGACTTTTTTTTTACCTGGGAAATAAATTTTATTAGAGAGAAAATGAGTGAAGCACTGGAGA
ATAACTAGGTGGAGGATAAGATATCCTCTTTTCTAATTTCTAATATTAATAATGGAAAGGTCAAG
AAAGGAGAAAGATTCCAAGATGCAGCAAGGCTATTCAATCTCTTTGCATGTTGATAGTGAACACT
ATCATGAAATAGCCATGAGAGAAGCCAAAAGTACAAACCTATCCTAAAGCAAACCAAGGGGTC
AAGAGATCCTAAAATTGCAGGCATCCTTCTTCAACCAAACAGGCCAAAGAACTGCAAAAACTGC

AGTGTCACAACTTTTGCCTCCTGATTGTTGGTCCTTTGAAATTTATCATAAGACCATTTCATCCTCT
TTAAAGTATATGTAATAGAGATTTTATTATATTTCCTAGACAACATGTTTTATAGATGTTCAGTCAT
TCAGCATCCAGGACATCGGCATTTAAAGTGTCTTTTTGACTATTATATTATTGTTTCATTATACATG
CTTAGGAGTTAAGATGCCATTTTACCTTTGTGATACTATTTTTCAAATCACCTGTAATCACATAAA
AAAGGCCACAGCCATTTTGAAGAACAGGCCTTGAAATTATAGTGAAGCAAATACTTATTTTTACT
TATTATACCTCACTTATATCATTTCCTTTACTTCCTCTTCCTACTTCTTCCTCTTTTTTCTGAAGATAC
TTGCTTTTTAATAGCCGCTGCTTTGTAATACTTGCTTTGTCACCAGGTAGCCAACAATCTATCTGCA
GAAATTCCTTCTTTTGGTAATCTTACTCAGCCAATTGCTGGGAGCAACAGAGTTCCTTACAATTTT
TCTGAAAGTGTTACTCTTCAGACGATGGACAATCAAGCAAATCCACATGAGCAGAAGAATAATA
CAGTTTCCTTAAGTGGTGTTGATTCCTTGGACACTTTGGTCAATGATAGATTGCAAAGTCAGGACA
GCTTTGGAATGTGGGTGAACCACATCATGTCTGATTCACCATGTTCAGTAGATGACCCAGCTCTTG
AATCACCAGTTTCATCCATTCATGAACCATATTCGTCTCTGGTAGTGGATAGTCAGGAATCTTCTC
TGCCAGAACAAGTATTTACTATCACTGATGTATCCCCTACATGTGTATCTTCCACCGAGAAATCA
AAGGTTTCCTTTGCCCTTCTTGTAGATCTACTTACTAGCAAATATTATCTGCTTTAAGCATATCTTT
CTTTAACAGTTAACTCTAATGTTTTATTTGGTTTCTTACTTCTTAGTTGATTCCGTAGTCTTTTATTTC
AATCTTGATTGAGTTCCTGTTGAATTCCATAGCTGTAAACTGCAAAAATATAAACTCCAAGGATT
ATTTTTAGTTCTTTTCAATCTTGCCAAAATCACTTTTAATTGGTGTTATGAGTTGCTCTTTGGCTACA
AGCATGCACATCTTCATGTATACAAATTGTAACAGAATTTAACTGAGTACATACTTGCTGCTTGAT
GAATTCATATGTTTGCTACCTGTTATTTTGACTTTCTGAATTCCTTTACTTGCAGGTCCTGGTGACT
GGATTTTTTCTTAAGGACTATATGCACCTTTCAAAGTCCAATCTATTATGTGTCTGTGGTGATGTTA
GTGTTCCTGCAGAAATTGTTCAGGTTGGGGTCTACCGTTGCTGGGTATCACCACACTCTCCAGGAT
TTGTGAATCTCTACTTGAGCATTGATGGCCATAAGCCTATTAGCCAAGTTGTTAATTTTGAATATC
GTACTCCTGCTTTGCATGATCCTGCAGTTTCAATGGAAGAGAGTGACAATTGGGACGAGTTTCGA
CAACAAATGAGGCTTGCATATTTGCTCTTTGCTAAACAATTGAACCTTGATGTTATCTCCAGTAAA
GTATCACCTAATAGACTTAAAGAGGCTAGGCAATTCGCCCTCAAAACATCATTTATTTCCAATAG
TTGGCAATACTTAATCAAGTCAACTGAAGACAACCAAATTCCATTTTCACAAGCAAAAGATGCC
CTGTTCGGAATTACATTGAAAAACAGACTAAAGGAATGGCTTTTGGAAAGAATTGTTTTAGGCTG
CAAGACTACTGAATATGATGCTCATGGCCAAAGTGTAATCCATTTGTGTGCAATTTTGGGATATA
ATTGGGCTGTTTCCTTATTTTCATGGTCAGGCTTGTCATTGGATTTCCGTGATAGGTTTGGATGGAC
AGCTCTTCATTGGGCAGCATATTGTGGAAGGTATAATACACAGACATCCCACTTGTTTAAACCAT
TAGCCCTTCTGTAACTACATTGACCTAACAATCCTTTTTTGGCATGGTTTCACCATTCATGTTCTTT
TCTGTTTCTCTTTTCCCTACATAAAGGTGAACATTAACATAATCAATGTGGATTTAGAATGATGTT
GGGTAAAGTAGACTGTTTGAACTTTGAATTAAAATTTTCTTATACATTTGATTTAATATTTGTATCT
TCAGGGAGAAAATGGTTGCAACTCTGTTGTCTGCTGGGGCGAAGCCAAATTTGGTCACTGATCCT
ACTCCACAAAATCCTGGTGGATGCACTGCTGCTGATCTTGCTTATATGAGGGGTCATGATGGCTT
AGCAGCTTATCTTTCAGAAAAATCTCTGGTTCAGCACTTCAATGACATGAGCTTAGCTGGAAATA
TCAGTGGCTCACTGGAAACTAGCACGACTGATCCAGTAAATCCTGCAAACCTTACCGAGGACCA
GCAGAACCTGAAGGATACGTTGACAGCTTACCGAACTGCTGCTGAGGCAGCTTCCCGCATACAT
GCTGCATTTAGGGAACACTCTCTAAAACTGAGAACTAAGGCGGTTGCATCTTCCAATCCAGAGGC
TCAAGCACGCAAAATAGTTGCAGCAATGAAGATTCAGCATGCCTTTAGGAACCACGAGACAAAG
AAAATGATGGCAGCTGCCGCTCGCATTCAGTGTACATACCGTACATGGAAGATTCGTAAAGAAT
TTCTTAACATGCGTCGTCAGGCTGTTAAAATTCAAGTAAAAGCTTCTCTTCTAGTTGGCACATACC
TTAACAGCAGATTTTGTTGGGCAAATTGACTAACATCTATGTTACAATGCAGGCTGCATTCCGGT
GCTTTCAAGTTCGGAAGCATTATCGCAAAATTCTTTGGTCTGTTGGTGTGGTTGAGAAAGCAGTGC
TGCGTTGGCGTTTGAAGAGAAGGGGCTTCCGTGGTCTCCAGGTCAAAACTGTCGATGCGGGAACC
GGAGATCAGGATCAGCAAAGTGATGTAGAGGAGGAATTCTTCCGGACTGGTAGGAAGCAAGCC
GAAGAACGTGTTGAGAGATCTGTAGTACGTGTGCAGGCTATGTTCCGTTCGAAGAAAGCACAAG
AAGAGTACAGGAGGATGAAATTGGCCCTTAATCAAGCAAAGGTAACTGTTAATCTATACATTTA

ATTAATCCTAGGAGGATTTGGTTTTGCATGTTTTTCACTTGTAGAATTTTCCTTTGTGCAGCTGGA
GCGGGAATACGAACAACTTCTTAGTACTGAAGTGGACATGCAACTGTAGATTTTGTAAGTTCCCC
TCGACCGTTTAAGACTTCCTTAGTATCCTAGAATGGGTCATTATATAAAGTTTTCATCTGTTATTGG
GCTCTATGGTTGAAACTTCTGATGCAAGGATACTGTGCCACCCCAAAGTTTAATATACTGAATAC
TAGAATAGTTATAGCCACAAGCCTGGTCCGGGGATGGTAATGCTTCTTCGTTTCTTTTAACTAAAA
AATAGCAGAGGCAACAAAGATCAGAGCGAATTTTTATAATTATTATTTATAGGCGCCAACACAT
CTTCAGTATTAGCCGATATGTACAAAGACACTTTTTGTTTCTTAAATTACCATATCCAATATCAGT
TATCTCTTTAGATTAATGATTAAATTTATCATGATACTCACCGATATTATGTAAGTTAAATCTAAA
TTAAAAACAAATTGTATCAATATCAAAGAGCAAGAGATAATCCAATGTCATTTGATTGGTATTTT
TTACGGGCACGTTTGTAAC

>Glyma.11G251900 (GmCAMTA14)

GCTTATCCATAAAATTTTCAAAACAATAATTACAATACATTGGCATTATAAATATTCATTATACTA
CCATCGATCTAATCCGCCTCGAGCAAATAAGTTCATGGACACAATTGTTGTCCTTCCTACCCACAT
GGAGGAATAATTGATTGGATTTCTATAGTTTCAGATATAGAAAAGACGATTATTATTAAGTTCTTA
CTTCTTAGTGTGTGTGTGGGCACCATTAAGTTCTGCATGATTCCAAGACTGACGTGAGCAAGAAA
CAATAAGATGAGTTCTGAATTCTTGGGCTAATTTGACATTGTCATGGCAGGTTTGGAGTACAGCA
TCGATGATCTGTTTCAAGAAGCTAAGAGGAGATGGCTCAAGCCTGTGGAAGCTCTTTACATTCTA
CGGAATCACGACCAGTGCAAGTTCACCCACCAGCCCCCTCATCAGCCAGCTGGTAACATAACAT
CCTGTGTTTTCTTTGTTCAAATGAAGGACAGGTTTTTCCGATTATCATAGGTTACTGATTGTCGAAG
ATGTTGCCTATCCACTTTCTTAACAGGTGGATCCCTGTTTCTGTTTAATCGTAGAATCATGCGTTCC
TTCCGTAAAGATGGTCATAACTGGCGGAAGAAAAAAGATGGCAAAACTGTGGGAGAAGCGCAT
GAACGGCTTAAGGTCTGTTATTAGTGTCCGTGGTGTTGTGATAATTAAGATTACCTACAATGATCA
TTAAAAGGTTTCACATGAGGGTGAGGCATGGTGCAACTGCATGTAGGTCTCAGGTTCAAATCCTA
GAAGTAGCCTCTTCACATGCGGTGATAAGGCTACATGCATCTACCCTCCCAAAACCTCTCCAAGT
GGAAGCCTTGTCTACTATGCTGTCCTTTTTCTTTTTTTTGACATCTATTTGTCTTGCTGCAGCTCATG
TCAGCCATTACCATCTTGCCAACTTTTGGCATGCTTTATTTTTTATGCAAATGCCCCAATTTGCACT
CTAATTTGGACCAAGCTCCTTCCTTTCTTTTAGTCTCAAATAAGAACCATGCAGAAAAAAATAGA
TGAAACATACAATAATTGTACCATACTGAGAGCTTGAAGTTTTCATACCAATGGAAATTATACGT
TCTTCTACTTCACTTTCTTTCCTTTTTATTTATTAGAGGAAATTGAATAATAAAAATTTGACATTGTT
GCTGTAATATCTTGTATGCATGATGTATTTCTACTTGGATTTTGTTTATTTTCTTGCTTTAATTGCAA
AAGTCTGCTAATGTTTCAAGCAGGTCTGAGTGATTAAATTACAGTCCATAAATGTACTAAATTATT
AGGAAGAAGTATTCAAATCTCAGACATATTGAAACAAAGTATTAATCGAAAATAATTAACGATC
CTCATTGGAAATTTCATTTTCAAATCTATCTCCTAATCTGCTCTGTACCATATATAGCTTCCAAAA
AGCTTTTGGGACAAATAGTAAGTTTTTTTATCTAGCACTCATTTTGTGCATGAGCTGTAACAGAA
CATTGAGATGTAACCAATTTGTTGTTTAATAGTTTTACTGGTATGGAGCATTTTTCTCTTTTAAATA
TAATTCACTTATTTTTTGCTATATTGATGCCTTTGTGCCAACATTTGTTTTGCGATAGAATTCTATGT
ATTGATTTTCTCAATCTGATTGGTCATTCTAATCTGCATGTTTAGGTTGGCAATGTTGAAATCCTAA
ACTGTTACTATGCACATGGAGAGGAGAACCGTACTTTTCAGAGACGGAGCTATTGGATGTTGGAG
CCGTGAGTAAATTTTAACTATTAGCTGGATACCAACCATATTATTATGTTGCCACTAGTTGTATCT
TTTAAGATCCACACACAAACTTACAAAGGTATATTAAAGGCAACTTTATTACCAAGATAAAATTA
TCTTGACTGTGAAACAAAATCAACCTTCAGTATATGTAACTAATATTAACATGGGTTCATTTATAT
GCCAATATGTTATTGTAGTGCATGTTCATATTTGCTCATACACTATTGTTTCTTGACAGGGAATATG
ATCATATTGTTCTTGTGCATTATAGAGAAACAAGTGAGGTGAGTTTCCAGAACATGATATCAGAT
AAACATAATATAGTATTTGCAGTTTATTATGTGTGAAAAGGTGGGAATCCTTTTTAAGCAACACA
TCTCAAAGAAAAGTATGTTCATCTATTTTGTTCATTGCTTGGGAATTGTATCAACAACCCAAGGTT
GAGTTTGACATTACTGTTTACTCTCACATTTTGCAGGGGAAATCCAATTCTGAGCATGTCACACAA

TTGCCATCAGGTTCCTCTCCTGCTTTTAGTCAGAGTCATAGCTCGTATACTGCTCACAACCCAGGA
ACAGCATCCATGATTGGTGATTCATGTGAACCTAATCAGAATTTTTCTTTTCCTGGGTCTTTAGAA
GTTACGTTCGAAGCACAAGCCTTGCGCCAATTGGAAGAACAGCTGAGTTTGAATGATGATGGCTT
CAATGAAATTGCACTTGATTTAGTGTCAGGTCAAGATCAAAGGGTGGTTTATAAGCAAGATAAAT
CTGCAGCTTTGTCTGGACCAAATGATCTAGGACAACCTTGTGATGGATATAATGGAAGACAAGGT
AATTTAGACAAACTTGAGCTGTTAATGTTTTTTTTGGAGCGGGGGAATAAAATTAATATATGTTTG
AAGTTAGTGAAACATTTGAATTATTATCCTATTTTACGCTTTTATCTTGTATTGTGTTTAGATTTGTT
CAGTTATAGATGCTTGTCTTCTACTTACGCAACATATGCAGGTGGCTTCTTCCACCCTCACAGCTA
ATTTATAACATACCTACTTATTTTAAGTTCTCTAAGGTTATCATAGCCATATATATATATATATATA
TATATATATATATATATATATATATATATATATATATATATATATATAGATTATATCTTCTATTCTT
CTAATTTGTCTATGGTACCCTTCTGTTTGATAGATTTTCTTTAAGTGAAACTTCCCCAATTGTTATA
TGCTGGTTGTTTCATGTGAATCTTTTAAAAGAGAGTGTAGGGAGATTATTGTGAATCAGGTAACC
AGAAATCAAAAAGGTTTGAAACAAAAGCCTCACAATTAGGTCTCTTGTATCTAGAATCACTCCA
GGAGGCATTCAAGAAATCTTCGTAAAAAAATATATTAACCATGCAGCACAGTTTTCAAATATTTA
ATGCACAGAACTCTATGTGTTAGATAATATGTCAATGTTGTTTATCCTCTTTTGTGCTTGAGAAATT
AAATTAAAAGGCATTCTAGTTTGTATTATTCAGTGAGTTGGGTTACAACATGTGTATCCATATATA
TACAAATAAAATGTTATCTAAAAGATAAACCAATCTTATGTAAAAGTTAAACTGGTTATCTAATC
TAATCTTCTAAAAGATAAACTACATGTCTGATTAAAATTAGCACTCCCCTAGTCCCCTTAAAGTTG
GAGCATATATGCCTTGTTACATATGTAAGTTATCCTAGGACCTCATAAGGACTTAGTCGATATGA
CTGCAAGTTGTTCCTTAAAATTCACAATGGTAGTCTTCATGATGCCAGGAGGATCTTTTCTCTAAT
GAAGCAACAATCAACCTCTATATGTTTAGTCCTCTTGAGAAAGATCAGATTGGAGGAAAGATGCT
AAGCTTGGTTGCCACAAGCAAGTTCCATAGGACCTCACAAGTAAATGCTTTAGCCATAAAAATTC
ATAAGTCATATTAGCCATAGCACGATATATTGCTTTGGTACATGACCTTGCAACCACAGTCCGTTT
CTTACTTTTTCGTGAGATTAAGTTACCACTGATGAAAAGATAAATTGCATATCTTATTACAATCAA
CAAAGTGTTTCGTTGTAAATTAGTTACTCATGATCCACTCAACTTTTTTTTCCCAAACTGTCACATT
CAGCTTTAGATCAGACTCAATTTATTTCACTAGATCTTTATAGTTTGTTTTTGCTACTCCTAATTAT
AATTAAACAACAACTGTAAAGAGAGGAAAGCAAAATCTTCTGAAATTATTGTATTTTCTGTAGAT
GACAGTGGCACATATTATCATGATTTTCTTGATGATTGTCCTGGTGGAAATGAAAAAACTATATA
CTGGACGAAAGTGCTGGAATCGTGTAAGCCCTTATCTGTGACCAAATTACCAGATCAACATGCAT
ATGAGGCGATTGGAAATGTGAGTATTCCAAAGATATACTTCATGCTGTCAAGAGTTTATAATACA
TTTTTCTTTTGCAACTTGCAAAGAAATAAAATCTCTATGCTTAAAACAGGAAAATACATTATTTTC
TTCGGGAAGGGGAGTGATTGCCAACCTGGAAAACAATCAGTGGCTAAACTCCAACAGTAATAAT
ATTGAAAACTGTATGACTTATTTCTACCTTAACTTGCTTGATGATTTCCCTTTTTTTGCATATAATG
AAACTTCTTCCATCTCTCTTTGATGTTCCATATCACTTGCTGTTTTATTGTTTCCTCAAGATGGTGGG
GTCAAATTTCCACCATATTCTCTAGCAGAAACTCCAGGAGCTAACTCTGACTACTATGAAACATT
TTTTGACCAATTCCAAAATCAAGGACCTCTGGGTGTAGATTCAAGCTTGACTGTTGTGCAGAAAC
AGAAATTTACCATTAGGGCAGTATCCCCAGAATACTGTTATTCTACCGAGACTACAAAGGTTTGT
GCCTTTTAATTTCAACACTTACTATGTCCTATTAATGATTTATCACTCTTTGCACTAGGGACTGTTA
TCTGCCGAATGATGGAATTGTTATTGTTTTTTGGTGCCTACTGCTCAGCTTTACCTATGATGTTCTT
GAAGAGCTTTAAAATGCATACACAACATGGTTCAAATACGTATGCATTTTTGGTATAATGTAATT
ATATATGTAATAACTAGGGAGTTAAAAAGGCAAGCTCTTAGCTTTTGATTTCAATACTTAATCATT
TGGGACATATACTTTGTGTCTACTCTCTTTCCGTTTGAACTTTTATTATTTTATTTATGATATGATAC
AAATACTATTGATTTTGTCTATATTTGAATCCTAATTTCCCAAGTTATATTCATAGGTGATCATTAT
TGGGTCATTTCTCTGCCATGACTCGGATTCTACCTGGGCCTGTATGTTTGGTGATGTTGAAGTTCCT
GCAGAGATAATTCAGGACGGCATAATCTGTTGTGAAGCTCCATCCAATCATCTTGGAAAGGTTAA
TTTGTGCATTACTTCAGGAAATAGGGTGCCATGCAGCGAAATGAGGGAGTTTGAGTTTCGAAATA
AGACTACTAGTTGCACTCGCTGTAATTCATTGGAAACAGAAGGAAGCAAAAGTCCTGAAGATCT
GTTATTACTTGTTCGATTTGCAGAGATGCTCCTTTCTTCTTCAACTACAAAGGATGACAGAATAGA

ATCTGGAAGTCATCTTTCAACAGAACAGAAAGATGATGATGATTCGTGGAGCCATATTATAGAC
ACTCTTCTAGATAGCACTAGAACACCATCTGATGCTGTTAAATGGCTTCTTGAAGAGCTGCTGAA
GGATAAGTTGCAGCTCTGGCTTTCTAACCGAAGAGATGAAGGGACGGGCTGTTCTTTGTCCAAGA
AAGAGCAGGGGATAATACACATGGTTTCTGGGTTGGGTTTTGAGTGGGCCTTGAACCCCATTCTC
AGTTGTGGTGTGAATATAAATTTCCGTGACATCAATGGATGGACCGCTCTTCATTGGGCTGCACG
GTTTGGGAGGTAATAGAAAACTGATTTGTTGATATTGATGTGCAATTGTGATAGCATTTTTGTTTC
AAAGCCTTGAATAATATACACATGATCAAATCTACTTAGAAACCTTTACTTTATCCTTGGGAGAA
GGGAATACCAATGCAAAGAAAGAGAACAAGGAGGACAAATAAAGCCTGTATCAAATACCTGCC
CAGTAACAGTATGCTTCACATGCAACAGGATGTTTCAATCATTCTTGAACATGTATATAAAGCCT
AATCAATCAAAGCCAAATGTTTAGTTTTTTGTTTCAACAAAAAAGGCTTTCCCCACCATATGGGA
TCGGCTACATTAATTAAATAACAACATTGTGTTGGACCAAAAACTGAATTTTAAGAAATTTCATT
TAAATCGAATTGTTTTTAAACCTTTTCTCTGGAGTTTTCCTTGGTCTTCCCCTATCTCTATTTGATCA
GACTGTACTCCATATAATTAATTCGTCTTACCAAGGCATCTATTGGTCTTCTAGCATGATCAAACC
ATCTTATTAGACCAAATTCTGACATAAATGATCATATCAAGATTGAAACTCTTGCTTTTAAAAGTC
TTTATAAATGATCTGTTCCATTCTAAGTGTTCTGGAAATTCAGGTCACTGAATATTTTGTTTCATGA
TAGGGAAAAAATGGTTGCTTCACTTATAGCTTCTGGTGCATCTGCTGGAGCAGTGACAGATCCAA
GTTCACAAGATCCAACCGGTAAAACTGCTGCATCTATTGCAGCCAGCCATGACCATAAGGGTCT
GGCAGGTTATCTTTCAGAGGTAGACCTAACAAGCCATCTGTCGTCCCTCACATTGGAAGAGAGTG
AGCTTTCTAGAGAATCTTCTGAGCTTGAAGCTGAGTTAACTGTCAGTAGTGTCTCTGAAGAAAAT
CTTGTGGCCAGTGAGGATCAGGTTTCACTAAAAGCTTCCTTGGATGCTGTTAGAAATGCAGCTCA
GGCAGCTGCACGGATACAAGCTGCTTTTCGTGCACATTCTTTTAGAAAACGGAAAGAAAGAGAT
GCTGCCGCCACAGTTCTAGATGGATATTGTATTGATGCAGGTAGCATTGATAATAACATTTCAGT
GCTTTCTGCCATGTCAAAACTTAGTTCTCAGAGCTGGCGTGATTACAAAGCTGCTTTGTCGATTCA
GAAGAAATATCGAAATTGGAAAGGTCGTATAGAATTCTTAGCATTGCGCCAGAAAATAGTTAAG
ATACAGGTAACTGTAATAGAAGTTGTTATTACAGTTTTGCTATACCAATTGAATTCCAAGAGTTCT
GTATGTACCTGGACATAGGCACTGTAGAAATGTAGGTACTTTGTAATGTAAATAAATAATAAATT
TGTTAATAAATTGGAAGGAGGGGGGAGATAAAAGACATAGCTAGACATGCACATGCATATATCA
AGAATAACCTTGGCTTTTCAATATTTATTTGGGCAGGCTTGTGTGAGGGGTTACCAGGTTCGGAAG
CAATACAAGCTAATATTATGGGCAGTTGGAATCTTGGACAAGGTTGTGTTACGATGGCGGAGAA
AACGAATTGGTATACAAAGTGTCCGGCAAGAAATGGAGTCAAATGAAGAAGAAAGTGATGATG
CAGATTTTCTCAATGTGTTCCGGAAAGAGAAAGTAAATGCAGCAATTGAAAAGGCCTTGAAGCG
GGTGCTTTCCATGGTCCACTCCACTGGTGCTCGTCAGCAATATAGACGCTTGCTTTCGTTGTATCG
TCAAGCCAAGGTAAATACACTAATGAAAGCAAAAAAGAAAATATAGGTCTGGGATACCACCAG
TTAGTTAAACCACTTTTTGCATTCAGATTGATCAAATAATAAGTGATTGTTACGAGTATAGCTTTC
ATAGTCCATTTCTTTACTGAGAGACAAATAAGATAACTGTAACGCTAGCGAGTAGCCACATGATA
TTCTCTTTGGACACACTGTTATTACTAGATGAAACCCAATTCTGGTGTCTCATAAAATGTTGAAAT
TTGAAAATCAGAATACAATCTGATCCCATTCTTTATTAGTGGGGCTAGCTGCATGAGGCTTTACTC
AACAATAAAAGTGTCAAATAGTGTGTTGCTAACACTTTTCATAAAGTAATTAGGAGCTTAATTAA
ACATGCTAAGCTAGCATATAGCAATGTTTTTTTATGTAAGGAGAGGTTCCCTGTTTTGTGCGATCA
TAACTTATTGATTCTTTTACAGATTGAACATGGCAGCACAAGTGATGAAGCACCGTTATCAACTT
CGGAAGAGAATGCTTCCAATATGGAAGATGATGATTTGTGTCAATTTTTGGACACCTTCTTCTGGC
CTTCCTAGTTTTTATTTATCTTTGATGATAAATTTCTGGCTTCCCCGCTCATATGGTCTGTGTATTTG
AATGTCTAGGTTGTATAGTGATAATGGCGATGGACACTTTCTCTTTTTATGTACAAAATGGGCTTC
AAGGCTCTGGTGGTATAGGATTTCAAATTCA

>Glyma.08G105200 (GmCAMTA15)

CTTTCTGCTTTTGCAACTTCCTTTCTCCGGTCCATTGTTTGCAGCGAATCTAGCAGCCTCTGCGGTT
CCAAAAATGCTCTGTGTCCGGTTCTACTACCTGTACTTGTAGCTTTTTATTCCTCGGATTCAACACC
ATGACACCTGGTAAACGTGTCTTTACTTTTTCTTAATGTTGTTATTATTATGATGTCTTGTTTAGAA
AAAGAAATTGGAAAATTGGGATGTTTAATGGTTTGGTGCGATGTACAGTGAAGTGAATTGAGTTT
GACTTGGTTTGATTGGCTAGTTGAAGTGAACCACATCACTCGTGACTTTGTATGTCTGTGTGTGCA
TTTTTGTTTAGTTTGCTGAGATTGGTGCTTTCAATAGGGACCATTTGAGTTTTGGAATTTGTTTGGG
ATGGTAAAAATCAAACATTCGCACTGACAGTAAAGCACTTTAGTATATTCTCTGTAGCATGCTTTT
TGCAATTTGATTTAATTTACTCAATTATTTTGCTGCTCCTAACACATTTTGCTTCCAAATGATATGG
AATATTGGAATAATTGATAGGTGCTGTACTGAGAAAGAGAATTTTAAGATTTTAGTTACATTGTG
CAGTTCTTATTAATGCATAAAAATGTCCCCGGCTTATCTAATTCTTCTGCTATCAGAAGGTAGAAA
AGAAAACAATGTTGATGATGATGGGATGGGTCTATATTTTTTGACTTATTTATGATGTGGTTTTAG
GTTACGAGTATGATATTAATGATCTGCATCAAGAGGCTCAAGCCAGATGGTTGAAGCCTGCAGA
AGTGATGTACATTTTACAGAATCATGAAAAGTTCCAGTTCACCCAAGAGGTCCCACAACAGCCA
ACCAGTAACATCTGTGTTCCCTTTATCCAATTGATTTTAGAAATAAAAATAAATGACAGTTTTTCT
CATTGTTTCTTCAGAATTGACTATAAAATGCTGATTGTTAACCTTATCAATTGATCTTCTTAACAGG
TGGATCCCTGTTTCTGTTTAACAAAAGAATCCTGCGATACTTCCGTAGAGATGGTCATAACTGGC
ATAAGAAAAGTGGTGGAAGAACTGTGGGAGAAGCACACGAAAGGCTGAAGGTGTGCTACTTGT
GTCTATACTGTTGTATTTGTTAGTTGTCCATGAAAAAGGAAAACTTGTTTGCAGGTGTCTAGAGGG
GGGTCTCAGTTGTAGAAATGCAGTCCATAAATTAATATGTAAGAGGAGAAAAATTGTTGACTCTC
CTGAATTGTTAGAAAAAAATATATTCATAACTTTGATAGATGTCTCAAACTTGGTGAATGGCGAC
AGATGGTATTATTAAAAAATACTTAAAAATCCTTATTTGAAGTTTCATTGAAGACTCTCAAGATG
CTTTTGAAATAGAATGATTTGACTATCAGCTGCCCCTTATATACAATAGATATAACTTATTTCTGTT
CAGTGATTATTTTTTAGCATGTTTTAAAAACATTATTCACTTATTGTTGCTATATTTACGAGTTTAG
GCCAACATTTGTTTTTGTGATGGTATTCCGTATATTGATTTCTTCAATCTAAATGATTGTACTAATC
TGCATGACTAGGTTCTAAATGTTGAAGCCTTAAATTGTTACTATGCACGCGGAGAGCAGAACCCT
GCTTTCCAGAGGCGGAGCTATTGGATGTTGGATCCGTGAGTAAATTTTAACTATTAGCTAACTGTA
TATCCTTCACACCATTAAGCTTATCTCACTACATGTTGTATCCTTTAAAACAAACATGCAATTTTC
AGGAAAAAGTCATCCTAGAGGTTAACTAAATTTTAAGTGAATTGCCAAGATTAATTTATCCTAGA
CTTTGAAACAGAATCAGCCTTCAATGTACATAACTATTTGTGTGATATGCTTATATATATATGTTA
TTGTAATAGTTATTCATAATCACTCATGCTATTGTTTCATGGCAGGGCATATGAGCATATTGTTCTT
GTGCATTATAGGAACACAAGTGAGGTGAGTCTCCGATACATGATATCAGATGAATTTAATAATA
GTACTTGTAGCTTGTGCATAGTGTCCCTCCAGAAAAAATTGGGGAATCCTTTTGTGCAAGATTGCTC
ACTGTCCTAACCTGTTTCTGATTAGTTGTTTCACACATATCCTTCCTATTTACTGTTCACCTGTGAA
ACTCAAATAATTTAGTTCATTTTCCTGTTCCATGAAATTTCAAACATTTTCTGTATTCTTTCATATTT
ACTTTTTGTAGATGGCTCGTCCAATTGTCAATTAGCTGTTAATATTTCTTGTTTAAGTTTGGTATGTT
GATTCTTTTATTCACCATTATAATTACTACTGTGTTGTAACTCCCTCTCCCCACCTCCCCTCTCTCAT
TTTCTATTTTCAAACTTCATTTATAAATGTACATGTATGCTTCCAAAGTCAGTGATTATTCCTAAGA
TTTGTAATGTCCTGTTGCTTTTCTGCACTAATTGTTTCATTTAATGGCTTTGTGCCTCAAATAAAGA
TATACTTAAATCTTGTTAAGAATCATATTTGATATCTTGGGCGGAATTATTAGATTATATTGTTTTA
TTCTGTGTGTGCATAGGAACATTGTTGGGCAGCCCATCTTTGAGTTTTGCATTAATGTATGCACTCT
CATGTTATAGGGAAAGCTAAGTTCTGGAGCTGGGGCACAATTGTCACCAAGTTCTTCTGTCTATA
CTCAGAGTCCTAGCCCATATTCTACTCAGAATCCAGGGTCAACATCCATACTTGGTGATTCATAT
GAACCTAATCAGAGTTTCTCCAGTCCTGGGTCTACAAAAGTTACATCTGAGATATTCGTTCTGAA
CAATAAAATGGGTCACATGGATTGGGCAGATACAGAATCAGGAACCTCATCTGAACTCGAGGTT
ACTCAAGCTTTGCGCCGATTGGAGGTACAGTTAAGTTTGAATGAGGACAACTTTGAAGATATTGT
TTCCTTCGGCAGTAAGCATGAAACTGTTCATGATTCAAATCCCAAGCACGATCAAAGGGTGATCA
GCAATCAAGAGCAATCTGCAGCTTTCTCTAGACCCGATGATCAAGGGCTATTTTATGATGGATGT
AATGGAAGGCAAGGTAAATTAAACAAACTTGATCTGTTGAGAACACATGGTTTAAATCAGTGAA

ATATCTGAGTGCTTAATCTATATTGTGTTTTAACCTGTTCACATACAGATATCTCTGTCTTCTACTT
GTTTAACTGTGCTTGCAGCATCTTACACCTGCACAGCTAGTTTTCTGTAATTTGTTGATGGAAATTC
ATAGTATGCCTGTTTAGATTAAAGTTGGTCCCAACTATCATAGCCATCTTCTTGTTTGGTGTATCTG
TTTCATTTTTCTCATCGAAGCATAATTCCCTAATTGGTATGTGCTGGTTGCGTTGTGTGAATTATTA
GAATTTGGGTTTGAGACTAACTCAACCCCAAAAACTAGCTCATTGGGTGATAGTTGCCCCCCATT
TTTATACATTATTTTGGCATTATTTCTAGTCAATGTTGGACTTGGGTTTTTTCCAATATGAATCTTAA
GAGAAATATGTGATTCTTATTATAAGTCAAGTAACCAGGAAAAAAATGGTTCTAAACAACTTACT
CACGGCTAGGTTTATTTGTATTTGACATTACTCCAGTAGACTTTAAAAAAACATATAAAAATGTG
GAACAGGCTGTAAAGGTTTGATTGGAGAATTCCATATGTTACATAATAAATGAAGTGTCTTTCTTC
TTTTATATGGTTGATAAGGAAAATAAATCAAATGTTCTAGTCTTGATACATTGCATGTGGCTGACA
GGTTAGCAATTGACTTACTTTTAACAATAATAAGCAAATTTGAAGTTTGAGAGAAAAGATTGTGA
TATTATTTTAAAAGATGCCATGAAGAACATAACACAACATACTTACAGGGTAGTGATAGCCAAC
CTGAATAAGGATCCTAATTAAACAAAGAAAACACTAAATTTAAGGAAAAATTCAAGGAAAATA
CTAAAACAGCCTCAAAGTATACTTATATAATGGATTTTGGGCTCAAAATCACGCTCTAGAAGGTA
AATCCTGGTTGCAACAAGAGGCTCGAAAGGAAATTTCTGTTGATATCATATGGGCCCTGTAACTC
CATCTAGATCAAAAGTTATGGTTGTTTAAATCTCACTTTCCTATTCAGGATCTATGGGTCATACGT
GTGGGTTTCTGGCACTCTGCTCACTCTAAGAAATCTTTTGTTACATGTGTGGCCAAAAGTTTGTCT
GTGAAGTTATTGTGGTTTTGCCTCTGCTTGAAGTTTTTCCTTGTAAATTAGTTATTCATCCATTGAA
AACCTTTTTTTTTAACTTAAATATTTTTAATTTCAAGTGAAATTCTGCTCTTTATAGTTTTTTTGTCCT
CTTCATTATGGAAGCATCAAATGGGAAGAGAGGAAGGTAAAAGATGCTTAAAGTGTTTATTGTT
ATTGGTGATGGTGGTGAATTTTATCATGAATTAATAGATCATGGTTACCCTGATGCAAATGAAAA
GGCTTTATGGACAGAGCAGCTGGAATCACATAAGTCCTCATCTGCAGTCAAGTTACCTCAGAAA
AATGTATACATGCCAGCTGAAAATGTAATTATCGCTTCAACATATTTAATGAAATTTATATCATTT
TGCAATTTTATGTTTGCTCAGATGTTGAAATAATTTTCTTCATGTGTTTCTGCAATTCTTAAAATATT
TGATTCTTAAAGCAGGAAAATTCAGTATCTTCTGCAAGAAGGGTACCAGTTTCCAACCAGGAAA
ACAGTCACTGGCTCAACTTCAACTGTAATAATTCTGAAAACTGTATGATTTACTTTCACCTGGTTA
ACTTAACAACCTTTTTGGTATATTTTGGTGTTTCTTCAACTTCTGATTCCCCTCCCATGTTAGCAGC
TGTTTTCTCACAACCTCAAGGTGTTGATGAAGTCAAATTTCCTGCCTATTCTTCTATGTTGGAAAC
ACAAGTAATTAATTCTGACTACTATGAAACATTGTTTGACCAAAGCCAAATTGGAGCACCACCA
GATGCAAATTCAAGCTTGACTGTTGCACAAAAACAGAAATTTACTATTAAGACTATCTCCCCAGA
ATGGGGTTATGCCACCGAGACTACCAAGGTTTGTGCATTTAAAGCCCAATTCCTTGCATGCCCTA
TGCAATGATTTATTACTCTTTGCATAACCGGTGTCTTGTGAACAATGAAAGTGCTCGTTCTTTTGAT
TCATAACTATTGGTGCTTATACCTTAACCTAGTCCCTGAAGAACTTTAAAATTTATTCTTAACTTTG
TTGAAGTCTATATGAAATTCTTGTGGTAGTGGTGGTGGTATAAAGCAATTATATATAAAGCTCAGT
CTATTTATAATAATTAGGTTAGGGAAATGTTGCTCAAGCTTGTGTCTTTGGACATTTAAGACTTAT
CAAGTTGGGGCATATATGTCATCTCTCTTTTTTTTCCCTTTCATCTTTCTTATCTTAAAATGATGTTT
AGTGTTGATTTTGTCCAAATTTGATTCCTAAAGCTCCATACTCTATGCATAGGTCATTGTTGTTGGG
TCTTTTCTCTGTCATCCCTCGGATTCTGCATGGGCTTGTATGTTTGGTGATGTTGAAGTTCCTATTG
AGATAATTCAGGATGGTGTAATCTCTTGTGAAGCTCCATCTCACCTTCCTGGAAAGGTTACTCTGT
GCATTACTTCTGGAAACTGGGAGTCCTGCAGTGAAGTCAGAGAGTTTGAATATCATGATAAGACC
AATAGTTGCACTCGATGTACTCAATCCGAAACAGAAGCCACTAGAAGTCCAGAAGAGCTGTTAT
TACTTGTTAGATTAGGGCAGATGCTTCTTTCTGCATCAACAATAAAGAATGACAACATAGAATCT
GGAATTCCTCTAATAAAACCGAAAGCTGATGATGATTCATGGAGCCATATTATAGATGCTCTTCT
AGTTGGCAGTGGAACGTCGAGTGGTACTGTTGATTGGCTTCTTGAAGAGCTTCTAAAGGATAAGT
TTCAACAGTGGCTTTCTTTCAGATCACGGGAAAAAGATGAAGAGACAGGCTGCTCTTTGTCCAAG
AAAGAGCAAGGGATTATTCACATGGTTGCTGGGTTAGGTTTTGAGTGGGCCTTGAACCCTATTCT
CACGTGTGGAGTTAATATAAATTTCCGTGACATCAATGGATGGACTGCCCTTCATTGGGCTGCAC
GTTTTGGAAGGTAATAGAGAATTCTAGAGAGCTTGCTACTTTGTTTACTGATTTATTTAGTTTTGGT

GAAAGATGTTAGTATTGCTATACAAGGCTTATAGGTAATGAAGATGGGGAAAGCATGCATACTG
AGGATCACGAAAATGAAAGTCTTGTTTCTTTTAGAAACCATTTTATAATTATCTGTTCCATTTTAA
ATTATTTTGGATGTTATTTTACTGAAGCTTTGTTATTCCATATAGGGAAAAAATGGTTGCTTCACTT
ATAGCTTCTGGAGCATCTGCTGGAGCTGTGACAGATCCAAATGCACAAGATCCAACTGGTAAAA
CTGCTGCATCTATTGCAGCCAGCAGTGGGCACAAGGGACTGGCAGGGTATCTTTCAGAGATAGCT
GTAACAAGCCATTTGTCATCGCTTACACTGGAAGAGAGTGAATCATCTAAAAGTTCTGCTTATCT
TCAGGCGGATAGGACTGTTAACAGTGTCTCCAAGGAAAATCTTACTGCCAATGAGGATCAGGCG
TCACTCAAAGATACCTTGGCTGCTATCAGAAATGTAACTCAGGCAGCTGCGCGGATACAATCTGC
TTTTCGCTCACATTCTTTTAGAAAACGGAGAGCAAGAGAAGCAACTGCTAGTACAGGTGGCATTG
GTACCATTTCAGAGATTTCTGCTATGTCAAAACTTGCGTTTCGGAACTCACACGAATATAATTCAG
CTGCTTTATCCATTCAGAAGAAATATCGAGGTTGGAAAGGTCGTAGAGATTTCTTAGCATTGCGC
CAAAAAGTAGTGAAGATACAGGTAATAGATGTTAACTTTATAGTTTGACTAAAACAAAATGAAT
TCCAAGAGCTACATGGATATTATTCTTAATTTATATATAACTAGCACTTAAAGATACTCTGAAAGT
GAATAAATTTGATTTTTAAACCAAAAAACAGTTGGTTATAGGCATGCACGTGCATACTTTGATTTC
AACGGCATTATTATCAACCTTAGTTATTTCTAATTTATTTGGGCAGGCTCATGTGAGGGGTTACCA
GGTTAGGAAGCATTACAAGGTAATATGGGCAGTTGGAATCTTGGACAAGGTTGTCCTACGATGG
AGGAGAAAAGGAGCTGGTTTACGAGGTTTCCGACAAGAGATGGACATCAATGAAAATGAAGAT
GAAGATATTCTCAAGGTGTTCCGAAAACAGAAATTAGATGTAGAAATTGAAGAGGCTGTTTCAA
GGGTGCTGTCCATGGTTGACTCACCGGATGCTCGTGAGCAATATCATCGCATGCTTGAGAAATAT
CGTCAAGCTAAGGTAAATAAATTAGTTGAATAATTTAAATATTGTTTTGCATGAGGCTTCCATAG
GGCATTCCGTGCTCAATCAGAAATAAAGACAAAATAGTAACCATACAGAGCTATCAACCAGTTT
GACTTTTTAGAGTGCAATTGCTTTGTGCTGTCAATGCATAAATTGTCAATTCCCTTGCAGGCTGAA
CTTGCGGGTACGAGTGACGAAGCATCATTATCAACTTCTGTAGGAGATGACTTATTTATGGATGA
TTTTTATCCATTTCCCTAAGAGCAAACGCTAGCCTTGTTAATCTTCATTCTTCTGAATGATCAATTT
GTGGAACAAGCTGACTTATTAGTTTGAGACAGTCTAGATTGTATAGTGATAATAGCGATGGACAT
TGTTACTTTGTACAGTATGAAATGGTCTTGAATGCAGGTGGGTGGGGTTTCAAATTGAATGGATTA
AGTTTCTTT

CDS of Soybean CAMTA family

>Glyma05G178200 (GmCAMTA1)

ATGGCTGAGGCCAGACACTACGTCCCCCCCTCTCAATTGGATATTAAGCAAATTATTTTAGAAGC
ACAGCATCGATGGCTACGTCCGGCTGAAATCTGTGCAATTCTCAGTAATCACAAGAAGTTTCTAA
TTGCTTCAGAACCTGCACATATGCCACCAAGTGGTTCACTTTTCTTATTTGATCGGAAGGTGCTGA
GATACTTCAGAAAGGATGGCCATAACTGGAGGAAGAAAAAAGATGGAAAAACAGTTAGAGAA
GCTCACGAGAGACTAAAGGCTGGAAGCGTGGATGTGTTGCATTGCTACTATGCCCATGGAGAAG
AAAATGAAAATTTTCGAAGACGCACATATTGGCTACTTGAAGAGGAACTCTCTCACATTGTTCTC
GTCCATTATCGACATGTGAAGGGAACCAAGGCAAATTTTACATGTGCTAAAGAAAATGAAGAAA
CTCTTCCTTATGCTCAACAAACTGATAAAATTATGCCCAAAACAGAGATGGAAACTTCTTTATCA
TCTACTCTTCATCCACATAGTTACCAGGTTCCTTCACAAACTATGGATAGAAGCATGAACAGTTC
CCAAGCATCAGAATATGAAGAAGCAGAATCTGCATTCAACAACCATGCAAGTTCTGAGTTTTATT
CTTTCCTTGAGTTAGAACGCCCTGTTGAGAAGATTACACCTCAACCTGCTGATTCTTATTCTCCTC
GGCCACTCACAAGAAAGTCTGTACCTAACATGAACTGCATTATTGAATCTGGGACAGATGATCA
AGAGAAGTCACCTGTCATTCCTGGGGTGAATTATATCTCACTCACTCAAGACAATAAAATCAAAG
ACATTCATAATTTTGGATTGACATATGAATCCCCAAAACCCCTTGGCTTTTCCTCATGGGAAGGTA
TCTTAAAAAATAATGCTGGAAGTCAACATGTGCCTTTTCAGCCTTTATTTCCTGGAACACAGCCTG
ACAACATGGGAATCAATAGCAAATTTTCCCAAGGACATGAGATAATGGTGCCATATTTGACCAC
CAGCATTGCTAAGCAGCATGAGAATGGAAGTCTCATACAAGCTGAAGGAAATTGGCAGGCTTAT
GATGTTGATTCTTTACGCATGTCCAGTTGGCCTATAGATAGTGCTTATTCAGGCTCATCATGTGAC
ATAACTTGCAGCAACCGTGAGCAGGAGGTTAATGATGTTGATTTGCAGAAATCCTTGGAACAGT
GTCTTCTACATCCATACAAACAAAACAAGGTTTTCATGCAAAATGATCCTCAGGAAAAACTTTTG
AATGAAAAAGAAAAGATAAAATCAGACCTAGAGGCCAATAGAATCCTGGATGGAATAGAAGAC
ACATATTTCACTTTTAAAAGGACTCTGTTAGATGGATCTCCAGCAGAAGAGGGTCTGAAGAAGCT
TGACAGTTTCAACCAATGGATGAGTAAAGAGCTTGCAGATGTGGAAGAGTCTAATAAACCATCC
ACTTCTGGTGGTTATTGGGATACAGTTGAAAGCGAAAATGAGGTTGGCAACACAACTATTCCTTC
ACAAGGGCACCTGGACACCTATGTATTGGATCCATCTGTTTCCCATGATCAGCTTTTTAGCATTAT
AGACTATTCCCCAAGCTGGGCATTTGAAGGGTCAGAAATTAAGGTTATTATTTCTGGACGATTCTT
AAGAAGTCAACATGAAGCAGAACAAGGTAAATGGTCATGCATGTTTGGCGAGGTAGAAGTGCC
AGCAGAGATCATTGCAAAAGGTGTTCTTTGTTGTCATACTCCTCCACACAAGGCTGGGAGGGTAC
CTTTCTATGTAACTTGTTCCAATAGGTTAGCATGTAGTGAGGTGAGAGAATTTGATTTCCAAGTCA
ACTATACTCCAGAAGTTAACACTACAGGTGAGAATAGAGGCAGCACTTTTGATACTTTTAGTATC
CGATTTGGAGAACTGTTGTCCCTGGGGCATGCCTTTCCTCAGAATTCAGATTCAATTAGTGTTAGT
GAGAAATCTCAACTGAGAAGTAAAATCAATTCTTTACTGAGGGAGGAGGAGGATGATTGGGACA
AGCTACTGAAACTTACTCAAGAGGAAGATTTTTCTCCAGAAAATTTACAGGAGCAGCTGCTTCAA
AATCTTCTGAAAGATAAGTTACATGCATGGCTCCTTCAGAAAATAACAGAAGAGGGGAAAGGCC
CTAATATATTAGATGAGGGTGGCCAAGGTGTGCTTCATTTTGCATCTGCTCTTGGCTATGATTGGG
CCCTAGAACCCACAATAGTTGCTGGTGTGAATGTGAACTTTCGGGATGTAAATGGATGGACTGCT
CTTCATTGGGCAGCATTTTGTGGCAGGGAGCGCACAGTTGCTTTCCTCATCTCTCTCGGCGCAGCA
CCTGGAGCACTGACTGATCCATGTCCAGAACATCCTTCTGGTAGAACACCAGCTGACCTAGCTTC
TGCAAATGGACACAAAGGAATTGCAGGGTATCTTGCTGAGTCTTCACTAAGTGCACACCTCACA
ACTCTCGATTTGAACAGGGACGCAGGAGAAAATTCCGGAGCAAAAGTAGTCCAAAGAGTCCAA
AACATTGCTCAAGTTAATGATCTTGATGGTTTATCATATGAATTGTCATTGAAAGATTCATTGGCT
GCAGTGCGTAATGCCACCCACGCTGCAGCTCGTATTCATCAAGTTTTCAGAATGCAATCATTTCA
GAGAAAACAACTGAAGGAATATGATGATGACAAACTTGGATTATCTGATGAACGTGCTCTTTCA
CTTGTAAAAATGAATATGAAATCACACAAGTCTGGACCACGTGATGAGCCTGTCCATGCTGCTGC

AGTACGAATCCAAAACAAATTCCGCAGTTGGAAGGGAAGAAGAGAATTTTTAATGATTCGACAA
CGCATAGTAAAAATTCAGGCTCACGTGAGAGGTCACCAGGTCAGGAAAAGCTGTGGGAAGATA
ATTTGGTCCGTTGGAATCTTGGAGAAGGTTATTTTACGTTGGCGTCGAAAAGGTAGTGGTTTGCGT
GGATTTAAACCGGAGGCCAATTCTGAGGGAACTATGATACAAGATGTATCTTCAACAGATGATG
ACTATGATGTCTTAAAAGAAGGCAGGAAGCAAACAGAGCAAAGGTTGCAGAAAGCCCTAGCTA
GGGTGAAGTCAATGGTTCAGTATCCAGAGGCAAGAGACCAATACCATAGGCTGTTGAATGTTGT
AACTGAGATCCAAGAAAATCAGGTAAAGCATGAGAGCAGTTATAACAATTCAGAAGAACCGAG
AGAATTCGGTGACCTCAACGATCTTGAAGCATTGTTGGACGAAGATATTTTCATGCCTACAGCAA
CTTAG

>Glyma08G14370 (GmCAMTA2)

ATGGCTGAGGCCAGACTCTACGCTCCCCCCTCTCAATTGGATATTAAGCAAATTATTTTAGAAGC
ACAGCATCGATGGCTACGTCCGGCTGAAATTTGTGCAATTCTCGGTAATTATAAGAAGTTTCGAA
TTGCTCCAGAACCTGCACATATGCCACCAAGTGGTTCACTTTTCTTATTTGATCGGAAGGTGCTGA
GACACTTCAGAAAGGATGGCCATAACTGGAGGAAGAAAAAAGATGGAAAAACAGTGAGAGAA
GCTCATGAGAGACTAAAGGCTGGAAGTGTGGATGTGTTGCATTGCTACTATGCCCATGGAGAAG
AAAATGAAAATTTTCAAAGACGCACGTATTGGTTGCTTGAAGAGGAACTCTCTCACATTGTTCTC
GTCCATTATCGACAAGTGAAGGGAACCAAGGCGAATTTTACAAGTGCTAAAGAAAATGAAGAAT
CTCTTCCTTATGCTCAACAAACTGATAAAATTATGCCCCAAACAGAGATGGACACTTCTTTATCAT
CTACTCTTCATCCACATAGTTACCAGGTTCCTTCAAAAACTGTCGATACAAGCATGAACAGTGCC
CAAACATCAGAATATGAAGAAGCTGAATCTGCATTCAATAACCATGCAAGTTCTGAGTTTTATTC
TTTCCTCGAGTTACAACGCCCTGTTGAGAAGATTTCACCTCAACCTGCTGATTTTTATTCTCCTCGG
CCACTCATAAATGATCAAGAGAAGTTACCTATTATTCCTGGGGTGAATTATATCTCACTCACTCA
AGACAATAAAAACAAAGACATTCTTAATGCTGGATTGACATATGAATCCCCAAAACCCCTTGGC
TTTTCCTCATGGGAAGGTATCTTAGAAAATAATGCTGGAAGTCAACATGTGCATTTTCAGCCTTTA
TTTCCTGGAACACAGCCTGATAACATGGGAATCAATAGCAATTTTTCTCAAGGAGAAGAGATAA
TGGTGCCATATTTGACCACCAGCATTGCTAAGCAGCATGAGAATGGAAGTATCATTAAAGCTGA
AGGAAATTGGCAGGTTTATGATGTTGATTCTTTACGCATGTCCAGTTGGCCTATAGATAGTGCTTA
TTCAGGCTCAACATGTGAGGTAAGTTGTAGCAACTGTGAGCAGGAAGTTAATGATGTTGATTTTC
AGAAATCCTTGGAACAGTGTCTTCTACATTCACACAAACAAAACAAGGTTCTCATGCAAAATGA
TCTTCAAGAAAAACTTTTGAATGAAAAAGAAAAGATAAAATCAAATCTAGAGGCCTATGGAATA
GAAGACACATATTTATCTTTTAAAAGGACTCTGTTAGATGGACCTCCAGCTGAAGAGGGTCTGAA
GAAGCTTGACAGTTTCAACCAATGGATGAGTAAAGAGCTTGGAGATGTGGAAGAATCAAATAAA
CCATCCACTTCTGGTGGTTATTGGGATACAGTTGAAACTGAAAATGAGGTTGGCAACACAACTAT
TCCTTCCCAAGGGCACCTGGACACCTATGTATTGGATCCATCTGTTTCCCATGATCAGCTTTTTAG
CATTATTGACTATTCCCCAAGCTGGGCATTTGAAGGGTCAGAAATTAAGGTTATCATTTCTGGAG
AATTCCTAAGAAGTCAACATGAAGCAGAACAATGTAAGTGGTCATGCATGTTTGGTGAGGTAGA
AGTGCCAGCAGTGATCATTGCAAAAGGTGTTCTTTGTTGTCATACACCTCCACACAAGGCTGGGA
GGGTACCTTTCTATGTAACTTGTTCCAATAGGTTAGCATGTAGTGAAGTGAGAGAATTTGATTTCC
AAGTCCACTATACTCCAGAAGACACTACAGGTGAGAATAGAGGAAGCACTTTTGATACTTTTAGT
ATCCGATTTGGAGAACTTTTGTCCCTGGGGCATGCCTTTCCTCAGAATTCAGATTCAATTAGTGTA
AGTGAGAAATCTCAACTGAGAAGTAAAATCAATTCTTTATTGAGGGAGGATGATGATGATTGGG
ACAAGCTACTAAAACTTACTCAAGAGAAAGATTTTTCTCCAGAAAATTTACGGGAGCAGCTGCTT
CAAAATCTTCTGAAAGATAAGTTACATGCATGGCTCCTTCAGAAAATAACTGAAGAAGGGAAAG
GCCCTAATGTATTAGATGAGGGTGGCCAAGGTGTGCTTCATTTTGCAGCTGCTCTTGGCTATGATT
GGGCCCTAGAACCCACAATAGTTGCTGGTGTGAATGTGAACTTTCGTGATGTAAATGGATGGACT
TCTCTTCATTGGGCAGCATTTTGTGGCAGGGAGCGCACAGTTGCTTTCCTCATCTCTCTCGGCGCA

GCACCCGGAGCACTGACTGATCCATGTCCAGAACATCCTTCTGGTAGAACACCAGCTGACCTAG
CTTCTGCAAATGGACACAAAGGAATTGCAGGGTATCTTGCTGAGTCTTCACTAAGTGCACACCTC
ACAACTCTCGATTTGAACAGGGACGCGGGAGAAAATTCCGGAGCAAAAGTAGTCCAAAGACTC
CAAAACATTGCTCAAGTTAATGATCTTGATGGTTTATCATATGAACTGTCATTGAAAGATTCACTG
GCTGCAGTGTGTAACGCCACCCAAGCCGCAGCTCGTATTCATCAAGTTTTCAGAATGCAATCATT
TCAGAGAAAACAACTGAAGGAATATGATGATGACAAACTTGGATTATCTGATGAGCGTGCTCTTT
CACTCATAAAAATGAATGTAAAATCACACAAGTCTGGACCACGTGACGAGCCTGTCCATGCTGC
TGCAATACGAATCCAAAACAAATTCCGCAGTTGGAAGGGAAGAAGAGAATTTTTAATGATTCGA
CAACGCATAGTAAAAATTCAGGCTCACGTGAGAGGTCACCAGGTCAGGAAAAGCTGTGGGAAG
ATAATTTGGTCCGTTGGAATCTTGGAGAAGGTTATTTTACGTTGGCGTCGAAAAGGTAGTGGCTTG
CGTGGATTTAAACCGGAGGCCAATTCTGAGGGAACTATGATACAAGATGTATCTTCAACAGATG
ATGACTATGATGTCTTAAAAGAAGGCAGGAAGCAAACAGAGCAAAGGTTGCAGAAAGCCCTAG
CTAGGGTGAAGTCAATGGTTCAGTATCCAGAGGCAAGAGACCAATACCATAGGCTGTTGAATGT
TGTAACTGAGATCCAAGAAAATCAGGTAAAGCATGAGAGCAGTTCTAACAATTCAGAAGAACC
GAGAGAATTCGGTGACCTCAATGATCTTGAAGCATTGTTGGACGAAGATATTTTCATGCCTACAG
CAACTTAA

>Glyma15G053600 (GmCAMTA3)

ATGGCGGAGGGTGCTTCTTACGGATTGCGTCGTCCATTAGATATTCAGCAATTACAATTTGAAGC
CCAGCATCGATGGTTGCGACCTGCAGAAATTTGTGAAATTCTTCGTAATTATCGGATGTTTCATAT
CACATCAGAACCTCATAACAGGCCACCAAGTGGTTCACTTTTTCTTTTTGATCGAAAGGTTTTGAG
ATACTTTAGAAAGGATGGGCATAATTGGAGAAAGAAAAAAGATGGAAAAACTGTGAAAGAAGC
TCATGAAAAGTTGAAGGTTGGAAGTGTTGATGTGTTGCACTGCTACTATGCCCATGGAGAAGAAA
ATGAAAATTTTCAAAGGCGAAGCTACTGGATGCTTGAACCGGATATGATGCACATAGTATTTGTC
CATTACTTGGAAGTTAAGGGTAACAAAAATATTGTTGTAAATAATGAAGGTGATGAAGTCCCAA
CAGATTCCCAAAAGGTAACTTCTCCATCGTCTAGCCTTCCCACACATCATAGCTGTGTTTCTTCAC
TGAGTACAGATTCCGTGAGCCCAACAACCAGTTTGATGTCTTTACATGAAGATGCTGATTCTGAG
GATATCCACCATGCAAGTTCTGGGTTGCACCCATTACATGAGTCACAACATAGTGGGAATAGTCC
ATTGACGGAAAAGATTGGTGCTGGTTCGAACAGTTCATATCTTATGCATCCCTTTTCAGGTGATAA
TGAACAGTCATCAATTTCTGGAACGGATTATGTCCCTGTTGTTCATGGGGATAAATTTAGAGGAA
ATGATACTGCTTATACTGATGGTCAGAAACCGCATGGCATGGCACCATGGGGCACTGTCTTGCAA
AGCACTGCCAAATTGCACAATGATCCTTCTCTTGCATCATTTCCTTCCATCCTACCTAGTTCAATG
GGAGACGTCCTTGAACAAGAACATACCATTTTTGGTGACCTTTTAATGAGCAAAAGTGGTCTCAC
TGAGGAAGCAGAGAGTTCTCAGTCTCTCCAATCAAATTGGCAGATTCCTTTTGAAGACAATTCAG
GAGGCATGCCAATGTTAACCCAAACCCAATCATTTGGTCTGCAATTTAGATCTGATTATGGCACC
GGTTTACTAGGGAACGAAACTCGTAATGCAAGTTCTGAAATTGCTCCGATCCTGTACAGTTTTCAT
GGTGAACCAAAAGAGCAGCCTATGCAGCAAAACTATCCACAAGAGCTTGAAGATGGACAATCT
CAACATGCACTAAAATCCAATTCTGCAAATAAAGTTCCTGATGAAGAAACTATCAACTATGGCTT
AACAGTGAAGAGCACATTATTGGACAGAGACGAGAGCCTGAAGAAGGTTGATAGCTTCTCTCGG
TGGATTACTAAGGAACTTGGAGAGGTGGCTGATTTGAATATGCAGTCCTCACCTGGTATTTCATG
GAGTACAGATGAATGCCAGCATGTGATAGATGATACCTCATTGAGCCCATCTCTCTCTCAAGACC
AACTGTTTAGTATAAATGATTTTTCACCTAAATGGGCTTATGCTGAGTCTGAAATTGAGGTGTTGA
TTATTGGATCATTTTTGAAGAGTCAACCAGAGGTGACAACCTGTAACTGGTCCTGTATGTTTGGGG
AAGTTGAGGTTCCTGCTGAGGTCTTAGCTGATGGAATTTTGTGCTGTCAAGCTCCTTGTCATAAGG
TTGGACGGGTCCCTTTCTATGTAACATGTTCCAATAGGTTAGCTTGTAGTGAAGTGCGGGAATTCG
ATTTCAGAGAGGGCTTTGCTAGAAATGTAGATTTTGCAGATTTTTATATCAGTTCAACTGAAATGC
TGCGTCATTTGAGACTGGAGGATTTTCTTTCTTTAAAGCCAGTGGACCCTTCAAATCATTCTTTTGA

GGGTGATATGGAGAAAAGAAACTTAATTTTTAAGCTCATTTCACTGAGAGAGGAGGAGGACTAT
TCAATTAAGGATGAAGTTACTAGAGAATTGGATATATCCCAACACATGGTGAAGGAGCATCTGTT
TCACAGGCAGTTTAAAGAGAAGCTGTACTCATGGCTTCTTCACAAAGTAACTGAGAATGGTAAA
GGGCCAAATGTATTAGATGAGGATGGGCAAGGTGTTCTACATTTAGCAGCTTTTCTTGGTTATGAT
TGGGCCATAAATCCAATTATATCTGCTGGAGTTAATATCAACTTCCGAGATGTGAATGGATGGAC
AGCTCTTCATTGGGCTGCATCCTGTGGCAGAGAGCGGACAGTTGCTGTCCTTGTCTCCATGGGTGC
AGATTGTGGAGCACTGACAGATCCATCTCCCGCATTTCCATCTGGTAGAACAGCTGCAGATCTTG
CCTCTAGCTATGGGCACAAAGGAATTTCTGGTTTTCTTGCTGAGTCTTCATTAACTCACCACCTCG
AAACCCTCACAATGGATGATCAAAAAGGTGGTCAGCAAGAAATTTCAGGAATGAAGGTGGTGC
AGACAGTTTCAGAACGATCTGCAACACCTGTGCACTATTGTGATATCCCAGATGCCATCTGCCTC
AAGGATTCACTTACTGCTGTGCGTAATGCAACACAAGCTGCTGATCGTATACATCAAGTATACAG
AATGCAGTCATTTCAGAGGAAGCAGTTAACTCAGTATGAAGGTGATGATGAGCTTGGATTGTCAG
ATCAGCAAGCTCTTTCTCTGCTAGCTTCCAGGGCATGCAAATCTGGACAAGGGGATGGTTTAGCC
AATGCTGCTGCAGTACAGATACAGAAAAAGTTTCGTGGTTGGAAGAAGAGAAAAGAATTCTTGA
TGATTCGTCAAAGAGTTGTTAAAATCCAGGCCCATGTTAGAGGTCACCAGATAAGGAAACAGTA
TAAACCAATCATATGGTCTGTGGGAATCCTGGAGAAGGTCATATTACGTTGGAGGCGCAAGGGT
AGCGGTTTACGTGGATTTCGACCAAATGCCATTAATAAAGTCCCCAATCAACAAAATGATTCTCT
GAAAGAGGATGATTATGATTATCTCAAGGAGGGACGGAAACAAAAGGAAGAAAAGATCCAAA
AGGCCCTATCAAGGGTGAAGTCCATGGTTCAGTATCCAGAGGCACGTGCTCAATACCGGCGTCTG
CTAAATGTTGTTGAGGACTTCCGTCAAACCAAGGCATCTAACAAGGGTTTGATCAATTCTGAAGA
GACAGTTGATGGTGTGGAGGATTTGATTGATATTGATATGCTTTTAGACGATGATAATTTCATTCC
TATAGCATTTGATTGA

>Glyma08G072100 (CAMTA4)

ATGTCGGAGCGTTCGTCTTTCGGATTGGGACCTCGATTAGATCTTCAGCAATTACAACTTGAAGCC
CAGCATAGGTGGTTGCGCCCTGCAGAAATATGTGAAATTCTTCGTAATTATCGGATGTTTCAAAT
CACATCAGAACCACCTAACAGGCCACCAAGTGGTTCCCTTTTTCTATTTGATCGGAAGGTTTTAA
GGTACTTTAGAAAAGATGGACATAATTGGAGAAAGAAAAAAGATGGAAAGACTGTGAAGGAAG
CACATGAAAAGTTGAAGGTGGGAAGTGTTGATGTGTTACACTGCTACTATGCCCATGGAGAAGA
AAATGAAAATTTTCAGAGGCGAAGCTATTGGATGCTTGAACTGGATATGATGCATATAGTATTTG
TCCACTACTTGGATGTTAAGGTTAACAAAACCAATATTGGTGGAAAAACATATTCTGATGAGGTT
ACATCAGATTCCCAGAAGAGCAGTTCTTTGTCATCTGGCTTTCCCAGAAATTATGGTAGTATGCCT
TCAGGAAGTACAGATTCCATGAGCCCAACGAGCACTTTGACATCTTTATGTGAAGATGCTGATTC
AGAGGATATTCATCAAGCAAGTTCAGGATTACACTCCTATCGTGAATCACAAAATTTGGGGAATG
ATCGTCCGATGGATAAGATTCATGCTCGTTCAAATAGTTCCTATCTTATGCATCCATTTTCAGATA
ATCATGGGCAGTTACCAGTTTCTGGAGCAGAATATATCCCACATGTTCAGGGGAATAAGTCTAGA
GCTAGTGATACTACTTACATTGAGGGTCAGAGAGCACATGGCATTGCATCCTGGGACAATGCCAT
GGAACAGTCTGCAGGAAAGCATGCTGATCCTTCTCTTGTATCATCTACGTCCATTCCATCAAGTGC
AATGGGAAACATACTTGACAAAAACCATACTGTTCCAGGCAACCTTTTAGGGCATAAAATTGCC
CTCACTGAAGTGGAAAGGGGCGCTCAGCCTGTCCAATCAAACTGGCAGATTCCTTTTGAAGACA
ATACTGGAGAATTGCCTAATTGGGGCTTTACCCAATCATTGGGTTTGGAATTTGGATCTGATTATG
GTACTAGTTTACTGGGGGATGTAACTAATAATGCAGGCCCAGAAATTGATCCTGAGCTGTTCACT
TTTAATGGTGAGTTAAAAGAGCAGTATACACATGGGCAATCTCAACCTGCACTGAAATCCAATTC
TGCGTATGAAGTTCCTGGTGAAGCAAGTATCAATTATGCCTTGACCATGAGGCGTGGATTATTGG
ATGGAGAGGAGAGCCTCAAGAAGGTTGATAGTTTCTCTAGGTGGATGACCAAAGAACTTGCAGG
TGTGGATGATCTGCATATGCAGTCTTCCCCTGGTATTTCATGGAGCACAGATGAATGTGGGGATG
TGATAGATGATACATCATTGCACCTGTCCCTGTCTCAAGACCAGCTGTTTAGCATAAACGATTTTT

CACCTAAATGGGCTTATGCTGAATCAGAAATTGAGGTGTTGATTGTTGGAACATTTTTAAAGAGT
CAACCTGTGGTGGCAAAATGTAACTGGTCCTGTATGTTTGGGGAAGTGGAGGTTCCCGCTGAGGT
TTTAGCTGATGGAATTTTGTGCTGTCAAGCTCCACCTCATAAGATTGGACGGGTTCCCTTCTATGT
AACATGTTCCAATAGGTTTGCTTGTAGTGAAGTTCGGGAATTTGAGTACAGAGAGGGCTTCGATA
GAAATATAAATTTTCCAGATTTTTTAACAATTCAAGTGAAATGGAGCTTCATTTGCGACTGGTGG
GGTTACTTTCGTTAAACTCAATGCACACTTTAAATCAAGTGTTTGAGGGTGATATGGATAAAAGA
AACTTAATATTCAAGCTTATTTCACTGAAAGAGGAAGAGGAATATTCAAGTAAGGAAGAGACAA
CTGCAGAGATGGATATATCACAACAGAAGTTGAAGGAGCATATGTTTCACAAGCAGGTTAAGGA
GAAGTTGTACTCATGGCTTCTTCACAAAGTTACTGAAACTGGTAAAGGGCCTCTTGTATTAGATG
AAGAGGGGCAAGGCGTGTTACACTTAATTGCTGCTCTTGGTTATGATTGGGCTATAAATCCTATC
ATAACTGCTGGAGTCAATATCAACTTCCGTGACGTGAATGGATGGACAGCTCTACACTGGGCTGC
ATTCTGTGGCCGAGAGCGAACAGTTGCTGTCCTTGTCTCCATGGATGCAGCCGCTGGTGCATTGA
CAGATCCATGTCCCGAATTTCCTTTGGGTAGAACACCTGCAGATCTTGCTTCTAGCAAAGGCCAC
AAAGGAATTTCTGGTTTTCTTGCTGAGTCATTATTAACTAGCCACCTCGAATCACTCACAATGGAT
GAAAATAAAGATGGTAGGAAAGAAACTTCAGGAATGAAAGTAGTGCAGACAGTTTCAGAGCGA
ACTGCGACACCTGTGCTTAATGGTGATATACCTGATGATATCTGCCTTAAGGATTCTCTTAATGCT
GTCCGTAATGCTACACAAGCTGCTGATCGTATATATCAAGTATTCAGAATGCAGTCATTTCAGAG
GAAGCAGTTAGCTCTGTATGAAGATGATGAATTTGGATTGTCAGATCAACAAGCTCTTTCTCTCCT
AGCTTCTAAGGCGTGCAGATCTGGTCAAGGAGAGGGTTTAGCCAATGCTGCTGCAATACAAATA
CAGAAAAAGTTTCGTGGTTGGACGAAGAGAAAAGAATTCTTGATTATTCGCCAAAGAATTGTTA
AAATCCAGGCCCATGTAAGAGGTCACCAGGTCAGGAAGCAGTATAAACCAATCATTTGGTCTGT
GGGAATCCTGGAAAAGGTTATATTACGCTGGAGACGCAAGGGCAGTGGTTTACGTGGATTTCGA
CCAGCTTCCCAGAACAAGGTCCCCGAACAACCAAGCGAATCTCCGAAAGAGGATGACTATGATT
ACCTCAAGGAAGGAAGGAAACAAAGTGAAGTGAAGTTCAAAAAGGCCCTTTCACGGGTGAAGT
CCATGGTTCAGTATCCAGAGGCACGGGCTCAATATCGGCGGGTGCTAAATGTAGTTGAGGACTTC
CGTCAAACCAAGGGAGGTAATTTGAATTTGATAAATTCGGAAGAGACAGTCGACGGTGTGGAGG
ATTTGATTGATATTGACATGCTTTTAGATGATGAAAATTTCTTACCTATAGCTTTCGATTGA

>Glyma05G117000 (GmCAMTA5)

ATGGCGGAGCGTTCGTGTTTCGGATTGGGACCTCGATTAGATCTTCAGCAATTACAACTTGAAGC
CCAGCATAGGTGGTTGCGCCCTGCAGAAATATGTGAAATTCTTCGCAATTATCAGATGTTTCAAA
TCACATCAGAACCACCTAACGGGCCACCAAGTGGTTCCCTTTTTCTATTTGATCGGAAGGTTTTAA
GGTACTTTAGAAAAGATGGACATAATTGGAGAAAGAAAAAAGATGGAAAGACTGTGAAGGAAG
CTCATGAAAAGTTGAAGGTGGGAAGTGTTGACGTGTTACACTGCTACTATGCCCATGGAGAAGA
AAATGAAAATTTTCAGAGGCGAAGCTATTGGATGCTTGAACCGGATATGATGCATATAGTATTTG
TTCACTACTTGGATGTTAAGGTTAACAAAACCAATGTTGGTGGAAAAACATATTCTGACGAGGTT
ACATCAGATTCCCAGAAGAGCAGTTCTTTGTCATCTGGCTTTCCCAGAAATTATGGTAGTGTGCCT
TCTGGAAGTACAGATTCCATGAGCCCAACAAGCACTTTGACATCTTTATGTGAAGATGCTGATTC
AGAGGATATTCATCAAGCAAGTTCAGGATTACACTCCTATCGTGAATCACAAAATCTGGGGAAT
GATCGTCCGATGGATAAGATTGATGCTCGTTCAAATAGTTCATATCCTATGCATCCGTTTTCAGGT
GATCATGGGCAGTTACCAGTTTCTGGAGCAGAATATATCCCACATGTTCTGGGGGATAAGTCTAG
AGCTAGTGATACTACTTACATTGAGGGTCAGAGAGCACAGGGCATTGCATCTTGGGACAATACC
ATGGAACAGTCTGCAGGAGAGTATGCTGATCCTTCTCTTGTATCATCTACAACCATTCCATCAAGT
GCAGTGGGAAACATCCTTGAAGAAAACCATACTGTTCCTGGCAAACTTTTAGGGCGTAAAAATG
CCCTAACTGAAGAGGAAAGGGGCTCTCAGCCTGTCCAATCAAATTGGCAGATTCCTTTTGAAGAC
AATACAGGAGAATTGCCTAATTGGGGCTTCACCCAATCATTGGGTTTGGAATTTGGATCTGATTAT
GGTGCTAGTTTACTGGGGGATGTAACTAATAATGCAGGCCCAGAAATTGTTCCTGAGCTGTTCAC

TTTTAATGGTGAGTTAAAAGAGCAGTCTGTGCACCAAAACTTCTCAAAGCTGTATACACATGGGC
AATCTCAACCTACACTGAAATCCAATTCTGAATATGAAGTTCCTGGTGAAGCAAGTATCAATTAT
GCCTTGACCATGAGGCGTGGATTATTGGATGGAGAGGAGAGCCTCAAGAAGGTTGATAGTTTCTC
TAGGTGGATGACTAAAGAATTTGCAGGTGTGGATGATCTGCATATGCAGTCTTCCCCTGGTATTTC
ATGGAGCACAGATGAGTGTGGGGATGTGATAGATGATACCTCATTGAATCTGTCCCTGTCTCAAG
ACCAGCTGTTTAGCATAAATGATTTTTCTCCTAAATGGGCTTATGCTGAATCAGAAATTGAGGTGT
TGATTGTTGGAACATTTTTGAAGAGTCAACCTGTGGTGGCAAAATGTAACTGGTCCTGTATGTTTG
GGGAAGTGGAGGTTCCTGCTGAGGTTTTAGCTGATGGAATTCTGTGCTGTCAAGCTCCACCTCAT
AAGATTGGACGGGTTCCCTTCTATGTAACATGTTCCAATAGGTTTGCTTGTAGTGAAGTTCGGGAA
TTTGAGTACAGAGAGGGCTTTGATAGAAATATACAATTTGCAGATTGTTTTAACAATTCAACTGA
AATGGTGCTTCATTTGCGACTGGTGGGGTTACTTTCTTTAAACTCAGTACGCACTTCAAATCAAGT
GTTTGAGGGTGATATGGATAAAAGAAGCTTAATATTCAAGCTTATTTCACTGAAAGAGGAAGAG
GAATATTCAAGTAAGGAAGAGACAACTGCAGAGATGGATATATCAAAACACAAGTTGAAGGAG
CTTATGTTTCACAAGCAGGTTAAGGAGAAGTTGTACTCATGGCTTCTTCACAAAGTTACTGAAAC
TGGTAAAGGGCCTCTTGTATTAGATGAAGAAGGGCAAGGCGTGTTACACTTAATTGCTGCTCTTG
GTTATGATTGGGCCATAAATCCAATCATAACTGCTGGAGTCAATATCAACTTCCGTGATGTGAAT
GGATGGACAGCTCTACACTGGGCTGCATTCTGTGGCCGAGAGCGAACAGTTGCTGTCCTTGTCTC
CATGGGTGCAGCAGCTGGAGCATGGACAGATCCATGTCCAGAATTTCCTTCGGGTAGATCACCTG
CAGATCTTGCTTCTAGCAAAGGCCATAAAGGAATCTCTGGTTTTCTTGCTGAGTCATTATTAACTG
GCCACCTTGAATCACTCACAATGGATGAAAATAAAGATGGTAGGAAAGAAACTTCAGGAACGA
AAGTAGTGCAAACAGCTTCAGAGCGAACTGCGACACCTGTGCTTTATGGTGATATACCAGATGCT
ATCTGCCTTAAGGATTCTCTTAATGCTGTCCGTAATGCTACACAAGCTGCTGATCGTATATATCAA
GTATTCAGAATGCAGTCATTTCAGAGGAAGCAGTTTGCTCAGTATGAAGATGATGAATTTGGATT
GTCAGATCAACAAGCTCTTTCTCTCCTAGCTTCTAAGACGTGCAAATCTGGTCAAGGAGAGGGTT
TAGCCAATGCTGCTGCAATACAAATACAGAAAAAGTTTCGTGGTTGGACGAAGAGAAAAGAATT
CTTGATCATTCGCCAAAGAATTGTTAAAATCCAGGCCCATGTAAGAGGTCACCAGGTCAGAAAG
CAGTATAAACCAATCATTTGGTCCGTGGGAATCCTGGAAAAGGTCATATTACGCTGGAGACGCA
AAGGCAGTGGCTTACGTGGATTTCGACCGGCTGCCCTGAACAAGGTCCCTGAACAACCAAGCGA
ATCTCCGAAAGAGGATGACTATGATTATCTCAAGGAAGGAAGGAAGCAGAGTGAAGTAAAGTT
CAAAAAGGCCCTTTCACGGGTGAAGTCCATGGTTCAGTATCCAGAGGCACGGGCTCAATACCGG
CGGGTGCTAAATGTAGTTGAGGACTTCCGTCAAACCAAGGGAGGTAATTTGAATTTGATAAATTC
GGAAGAGACAGTTGATGGTGTGGAGGATTTGATTGATATTGACATGCTTTTAGATGATGAAAATT
TCTTACCTATAGCATTCGATTGA

>Glyma08G19101 (GmCAMTA6)

ATGGCGGAGGGTGCTTCTTACGGATTGCGTCGTTCATTAGATATTCAGCAATTACAATTTGAAGCC
CAGCATCGATGGTTGCGACCTGCAGAAATTTGTGAAATTCTTCGTAATTATCGGATGTTTCATATC
ACATCAGAGCCTCATAACAGGCCACCAAGTGGTTCACTTTTTCTTTTTGATCGAAAAGTTTTGAGA
TACTTTAGAAAGGATGGGCATAATTGGAGAAAGAAAAAGGATGGAAAAACTGTGAAAGAAGCT
CATGAAAAGTTGAAGATTGGAAGTGTTGATGTGTTGCACTGCTACTATGCCCATGGAGAAGAAA
ATGAAAATTTTCAAAGGCGAAGCTACTGGATGCTTGAACCGGATATGATGCACATAGTATTTGTC
CATTACTTGGAAGTTAAGGGTAACAAAAATATTGTAGTAAATACTGAAGGTGATGAGATCCCAT
CAGATTCCCAAAAGGTAACTTCTTCATCGTCTAGCCTTCCCACACATCATAGCAGTGTTCCTTCTC
TGAGTACAGATTCCGTGAGCCCAACAACCAGTTTGATGTCTTTACGTGAAGATGCTGATTCTGAG
GATATCCACCAAGCAAGTTCTGGGTTGCGCCCATTATATGAGTCACAACATAGTGGGAATGGTCC
ATTGACGGAAAAGATTGGTGCTGGTTCGAACAGTTCATATCTTATTCATCCCTTTTCAGGTGATTA
TGAACAGTCATCAATTTCTGGAACGGATTATATCCCTGTTGTTCATGGGGATAAATTTAGAGGAA

ATGATACTGCTTATATTGATGGTCAGAAAACACATGACGTGGCAACATGGAGCACTGTCTTGCAA
AGCACTGCCAAATTGCATAATGATCCTTCTCTTGCATCATCTCCTTCCATTCCATCTAGTTCAATG
GGAGATGTCCTTGAACAAGAACATACCATTTTTAGTGACCTTTTAATGAGCAAAAGTGGTCTCAC
TGAGGTAGCAGAGAGCTCTCAGTCTCTCCAATCAAATTGGCAGATTCCTTTTGAAGACAATTCAG
GAGGCATGCCAATGTTAACCCAAACCCAGACATTTGGTCTGCAATTTAGATCTGATTATGGCACC
GGTTTACTAGGGAATGAAACTCGTAATGCATGTTCTGAAAGTGCTGCGATCCTGTACAGTTTTAAT
GGTGAACCAAAAGAGCAGCCTATGCAGCAAAACTATCTACAAGAGCTTGAAGATGGACAATCTC
AACATGCACTAAAATCCAATTCTGCAAATAAAGTTCCTGATGAAGAAACTATCAACTATGGCTTA
ACAGTGAAGCGCACATTATTGGACAAAGACGAGAGCCTGAAGAAGGTTGATAGCTTCTCTCGGT
GGATTACTAAGGAACTTGGAGAGGTGGCTGATTTGAATATGCAGTCCTCACCTGGTATTTCATGG
AGTACAGATGAATGCCAGCATGTGATAGATGATACCTCATTGAGCCCATCTCTCTCTCAAGATCA
ACTGTTTAGTATAAATGATTTTTCACCTAAATGGGCTTATGCTGAGTCCGAAATTGAGGTGTTGAT
TATTGGATCATTTTTGAAGAGTCAACCAGAGGTGACAACCTGCAACTGGTCCTGTATGTTTGGGG
AAGTTGAGATTCCCGCCAAGGTCTTAGCTGATGGAATTTTGTGCTGTCAAGCTCCGCGTCATAAG
GTTGGACGGGTCCCTTTCTATGTGACATGTTCCAATAGGTTAGCTTGTAGTGAAGTGCGGGAATTC
GATTTCAGAGAGGGCTTTGCCAGAAATGTAGATTTTGCAGATTTTTATAACAGTTCTGAAATTCTG
CTTCATTTGCGACTAGAGGATTTTCTTTCTTTAAAGCCAGTGGACCCTTCAAATCATTCTTTTGAGG
GTGATATGGAGAAAAGAAACTTGATTTTTCAGCTCATTTCACTGAGAGAGGTGGAGGAATATTCA
ATTAAGGATGAAGTGACTACAGAATTGGATATATCCCAACACATGGTGAAGGAGCATCTGTTTC
ACAAGCAGTTTAAAGAGAAGCTGTACTCATGGCTTCTTCACAAAGTAACTGAGAGTGGTAAAGG
GCCAAATGTATTAGATGAGGATGGGCAAGGTGTGCTACATTTAGCAGCTTTTCTTGGCTATGATT
GGGCCATAAATCCAATTATATCTGCTGGAGTTAATATCAACTTCCGAGATGTGAATGGATGGACA
GCTCTTCATTGGGCTGCATCCTGTGGCAGAGAGCGCACAGTTGCTGTCCTTGTCTCCATGGGTGCA
GATTGTGGAGCATTGACAGATCCATCTCCCTCATCTCCAGCAGGTAGAACAGCTGCAGATCTTGC
CTCTAGCTATGGGCACAAAGGAATTTCTGGTTTTCTTGCCGAGTCTTCATTAACTCACCACCTCGA
AACCCTCACAATGGATGATCAAAAAGGTGGTCGGCAAGAAATTTCAGGAATGAAAGCGGTGCA
GACAGTTTCAGAACGATCTGCAACACCCGTGCACTTTGGTGATATGCCAGATCTCTGCCTCAAGG
ATTCGCTTACTGCTGTGCGTAATGCAACACAAGCTGCTGATCGTATACATCAAGTATACAGAATG
CAGTCATTCCAGAGGAAGCAGTTAACTCAGTATGAAAGTGATGAGCTTGGATTGTCAGATCAGC
AAGCACTTTCCCTTCTAGCTTCCAGAGCATGCAAATCTGGACAAGGAGATGGTTTAGCCAATGCT
GCTGCAGTACAGATACAGAAAAAGTTTCGTGGTTGGAAGAAGAGACAAGAATTCTTGATGATTC
GTCAAAGAGTTGCCCATGTAAGAGGTCACCAAGTAAGGAAACAGTATAAACCAATCATCTGGTC
TGTGGGAATCCTGGAGAAGATCATATTACGTTGGAGGCGCAAGGGTAGTGGTTTACGTGGATTTC
GACCAAATGTCATTAATGAAGTCCCCGATCAACAAAATAATTCTCTGAAAGAGGATGATTATGA
TTATCTCAAGGAGGGACGGAAACAAAAGGAAGAAAAGATCCAAAAGGCCCTATCAAGGGTGAA
GTCCATGGCTCAGTATCCAGAGGCACGTGCTCAATACCGGCGTCTGCTAAATGTTGTTGAGGACT
TCCGTCAAACCAAGGCATCTAACGAGGGTTTGATCAATTCTGAAGAGACAGTTGATGGTATGGA
GGATTTGATTGATATTGATATGCTTTTAGACGATGATAATTTCATTCCTATAGCATTTGATTGA

>Glyma17G04310 (GmCAMTA7)

ATGGCTGAGACCACAAAATACATTCCCAACTCACAATTAGAGCTTGAAGAGATATTGAATGAAG
CAGAACATCGATGGCTTCGACCTGCTGAAATATGTGAGATACTTCGCAATCATAAGAAGTTTAAA
TTAACACCAGATCCACCTGTTATGCCTCCAGCTGGATCACTATTCTTGTTTGATCGAAAAGCACTT
CGTTACTTTCGCAAGGATGGTCACCGGTGGAGGAAGAAAAAAGATGGGAAGACTGTAAGAGAA
GCTCATGAAAAGTTGAAGGCTGGTAGTGTGGATGTCTTGCATTGTTACTATGCTCATGGTGAGGA
TAATGAATACTTCCAACGGAGAAGTTATTGGATGCTTGACGAGCAGTTAGAGCACATTGTCCTTG
TGCATTATCGTGAAATTAAAGAGGGCTGCAAGTCTGGCATCTCACATTTGCCTGTAGTTCCAGTA

ACCCTGGTTGGTAGTTCTCAAAATACTTCAGTGCTTTCCTCTACCAAAATAAATTCACCAATATCT
CTAGTTCAGACATCTTTCACATCAAGTGCAAATAAAGTTTATCAGAATGGACGAGCCTCAGAGCA
TGAGGATGTCAATTCAAAGAATGGTCCTCAAGCCTCCTCTCATGCTCAGCCTATCAGCAATTATG
TACTTCACAGTGCACCTTGGCTTACACATGAAGCTGCAGGGTTTTCTGAGTTGTTAAGGAACCCCT
TGATTTCATCATGGCCATCTTCTTTCCCTAGCTACTCTCCTGGTACTGGCTTGTCTCCCTGGACCTC
AATTCAGAACTCTAGCAGAAACACAATTAATATGCATGATGGAAAGCATCATGTTGAAGCCTCT
GAAGCAGACTTAACTGTTCGTAAGCTAAGCAATGCTGGGTTAGATTCTGTTCACCGAATGCAAGA
TGGTGTAATTTTCAGAGATAGGCTCATCACTGACATGTGCGTCCAGCCAGTTATAGATTTGCCTAC
TGTTAATCAGGTGAAAAACGAGCATGGTCTGGATTCATTTCATGCTCAAGTCCATGATCATAATG
ATCATCCAGTTGTTGCAACTACTAAAATCCTAGTTGAACAAAAACTTCAAGATGGTGGTTTATAC
AATGATGAATCAGAACAAGTTGAATATGGAGAAATGAAGAAACTTGATAGTTTTGGAAGGTGGA
TGGATAAGGAAATTGGTGGAGACTGTGATAATTCCTTGATGGCTTCAGATTCTGGTAATTATTGG
AGCACACTTGATGCTCATAGTGAGGATAAGGAGGTATCTAGTTTACGCCACATGCAGTTGGATGT
GGATTCATTAGGTCCTTCTCTTTCCCAAGAACAACTATTTAGTATCCATGACTTTTCTCCTGATTGG
GCATATACTGGAGTTAGAACAAAGGTTTTAATAGTTGGCACATTTCTGGGGAGTAAAAAGCCCTC
TAGTGAGACCAAATGGGGATGTATGTTTGGTGAAATTGAGGTTTCAGCTGAAGTTTTGGCGGATA
ATGTCATAAGGTGTCAGACTCCTTTGCATTCACCTGGACGTGTACCATTCTATATTACCTGCAGTA
ATAGGTTAGCCTGCAGTGAGGTCAGGGAATTTGAATTTGATGAAAATCCAACCAAATTTTTAGGT
CCTGAGGGTATTAAAATCTCACCAGAAGAAGAGGTACGACTTCAAATGCGACTTCTTAAACTAG
TAGACTTGGGACCTGACAATAAGTGGTTGAAGTGCTCCGTTTCAGAATGCGAAAAATGTAAACTC
AAGGGAACAATGTATTCCGTGAGAGATGATAGTGGAGTATTTGAAGAAACTTTCCAGATTGATG
GAATTGGTCATATAAACCATAGAGATATATTATTTCAGAGATTAGTGAGAGACAAGCTTTATGAG
TGGTTGATATATAAGGTTCATGAAGGAGGAAAAGGACCACATGTGTTGGATGATGAAGGACAAG
GAGTAATACATTTGGCAGCGGCACTTGGTTATGTGTGGGCTATGGCCCCATTAGTTGCAGCTGGT
ATCAGTCCTAACTTCAGAGACTCTCGTGGAAGAACAGGACTTCACTGGGCATCATATTTTGGGAG
AGAAGAAACTGTTATTGTGCTAGTTCAGTTAGGTGCAACCCCAGGTGCTGTTGAGGATCCGACAT
CAGCATTTCCTCGAGGACAAACGGCAGCTGATTTAGGTTCAAGTAGAGGACATAAAGGCATTGC
TGGGTATTTGGCAGAGGCAGATCTGACAAATCAATTATCTGTATTGACTGTCAAGGAAAATGAA
ACTGGCAACATTGCCACAACAATAGCAGCTAACAGTGCTCTTCAGTCTGTTGAAGATGACTCATC
TAGTATGACAATGGATGAGCAACATTATCTCAAAGAGTCACTTGCTGTGTTTCAAAAATCAGCTC
ATGCAGCTGCTTCAATCCTAGCAGCCTTTAGAGCAAGGTCATTCTGTCAGAGACAATTAGCCCAA
AGTAGCAGTGATATTTCTGAAGTACTTGACGTAGTTGCTGATTCTTTGAGCAAGGTACAGAACAA
GGGTCACTTTGAGGATTATTTACATTTTGCAGCTTTAAAGATTCAAAAGAGATATCGGGGATGGA
AAGGAAGAAAGGATTTTTTGAAGATACGTGACCGCATTGTAAAAATCCAGGCTCATATTAGAGG
ACACCAAGTTCGTAAGCAGTACAAAAAAGTTGTGTGGTCTGTTAGCATTGTGGAAAAAGCAATC
CTGCGCTGGAGACGGAAAGGGGCTGGTCTGCGAGGATTCCGGGTTGGGCAGCCAGTTGGTGTTG
TGGTTAAAGATGCCGAGAAAAGTGACGAGTATGAATTTCTTAGCATTGGGAGGAGACAAAAATC
AGATGATGTAAAGAAAGCTCTAGATAGAGTCAAGTCCATGGTTCGTAACCCAGAGGCAAGGGAT
CAGTATATGAGGCTCATCATGAAATATGAAAAGTTTAAGATTGATGATGGAGGAAGCAGTCAAT
CACAGCATGTCGGTTAG

>Glyma15G15350 (GmCAMTA8)

ATGATGTCACACAACTTAACGGGGCAGCTTGTGAGTGCAGAGATACATGGCTTTCACACGTTGGA
GGATTTGGATGTTAGTAATACTATGGAGGAAGCAAAAAGCAGATGGCTTCGTCCCAATGAGATC
CATGCCATACTCTGTAATCACAAATATTTCAAGATCAATGTCAAGCCAGTGAATTTGCCCAAAAG
TGGTACTATTGTACTATTTGACCGCAAGATGCTTAGGAACTTCAGAAAAGATGGGCACAATTGGA
AAAAGAAAACCGATGGGAAAACTGTCAAAGAAGCCCATGAACACTTAAAAGTTGGCAATGAAG

AAAGGATCCACGTATATTATGCACATGGCCAAGATAACCCAACCTTTGTTCGTAGATGTTATTGG
TTGCTTGATAAGAATCTGGAGCACATAGTCCTTGTTCATTATCGTGACACACAAGAGTTACAATT
GCAAGGTTCTCCTGCTACACCCGTAAATTCAAATTCAAGTTCTGCCTCTGATCCAGCTGCATCTTG
GATTCCATCAGAAGACTTGGATTCTGGGGTGAACAGTGCTTATGCTGTTGAGCTTAATGACAATTT
AACTGCCAAAAGTCATGAGCAGAGGCTTCATGAGATTAATACACTTGAATGGGATGACCTAGTG
GTTCCAAATGTCAACACTTCTACTACATCTAATGGAGGCAACGTCCCATATTCATTTCAAGAAAA
TCAAAGTTTACTGAGTGGAAGATTTGGCAATGTATCCAGTAATCCTTCAGCAGAAATTCCTTCTTT
TGGTAATTTAACTCAGCCAGTTTCTGGGAGCAATAGTGCCCCTTACAGTTTCCCAGATAGTGCTAT
TCTTCTGAAAAATAGTCCTATATCATCGGGAGGCGTTGACACCTTGGGCACTTTGGTCAACGAAG
GCTTGCAAAGTCAGGACAGCTTTGGAACGTGGATGAACATCATATCGGATACACCATGTTCCATA
GATGAATCAGCTCTTAAAGCATCAATTTCTTCTGTTCATGTACCATATTCATCACTGGTGGCAGAT
AATCTGCAATCTTCTCTGCCAGAACAAGTATTTAATCTCACTGAGGTGTCCCCTACATGGGCATCT
TCCACTGAGAAAACAAAGGTCCTGGTAACTGGATATTTTCATAATAACTATGAAAACCTTGCAAA
GTCCAACCTACTGTGTGTCTGTGGTGATGTAAGTGTTCCTGTAGAAATTGTTCAGGTTGGAGTTTA
CCGGTGCTGCGTACCACCACACTCGCCTGGATTGGTCAATCTCTATTTGAGCTTTGATGGCCATAA
GCCCATCAGCCAAGTTGTTAATTTTGAGTATCGTACTCCTATTTTGCATGAGCCAACTGCTTCAAT
GGAAGAGAAATACAACTGGAATGAGTTTCGACTTCAGATGAGGCTTGCTCATTTGCTCTTTGCTT
CAGACACGAGCCTTAACATTTTCTCAAGTAAAGTATCACCGAATGCACTGAAAGAGGCTAGAAG
ATTTTCCTTCAAAACATCATATATCTCCAAAAGTTGGCAATACTTGATGAAGTCAATTGATGACA
ATACAATTCCATTTTCAAAAGTAAAAGATTCCTTGTTTGAAACTGCATTGAAAAACAAATTAAAG
GAATGGCTTTTGGAACGAATTATCTTAGGCAGAAAGAGTACTGAATATGATGCGCAAGGGCAAG
GTGTAATTCATTTGTGTGCAATGCTAGGATATAGTTGGGCAATCTCCTTATTTTCTTGGTCGGGCTT
GTCGCTGGATTTCCGTGACAAATTTGGATGGACAGCTCTTCATTGGGCAGCATCTTATGGAATGG
AGAAAATGGTTGCAACTCTGTTGTCTTGTGGGGCAAGGCCAAACTTGGTCACAGATCCAACTCCA
CAATATCCTGGTGGATGTACTGCTGCTGATCTTGCCTATATGAAGGGATGTGATGGCTTAGCTGCT
TTCCTTTCTGAGAAGTCTCTGGTTGAGCAGTTCAATGAAATGAGTTTGGCTGGAAATATCAGTGGC
TCACTGGAAACTAGCTCAACTGATCCAGTAAATGCTGAGAACCTTACTGAGGACCAACTGTACGT
TAAGGAGACTTTGGCAGCTTACCGTATATCTGCCGAGGCTGCAGCACGGATTCAAGCTGCATTTA
GGGAGCATTCATTTAAATTACGATATAAGGCAGTTGAAATTATCAGCCCAGAGGAAGAAGCACG
TCAAATAGTAGCAGCAATGAGGATTCAACATGCCTTTCGCAACTATGAGTCAAAAAAAAAGATG
ACAGCCGCCGCTCGGATTCAGCATAGATTTCGTACATGGAAGTACCGTAGAGAATTTCTCAACAT
GCGGCATCAAGCTATCAAAATTCAAGCTGCTTTCAGGGGCTTTCAAGCACGGAAGCAATATCGG
AAAATCATATGGTCTGTTGGGGTGCTTGAGAAAGTGATCCTGCGTTGGCGTTTAAAGAGAAAAGG
CTTTCGTGGTCTCCAGGTTAATCCTGCCAGGGAGGAGACGCAGGAAAGTGATTCAATTGCAGAG
GAGGATTTCTTTCGCACCGGCCGGAAACAAGCTGAAGAACGCATTGAGAGGTCTGTCATACGTG
TGCAGGCCATGTTCCGTTCTAAGAAAGCACAAGAAGAATATAGAAGGATGAAGCTGACACATA
ATCAAGCAAAGCTGGAACTGGAATTAGAAGAATTCCTTGATTCTGAAGTTGACATGTTACCCAAG
ACATAG

>Glyma09G04310 (GmCAMTA9)

ATGATGATGTCACACAACTTAACGGGGCAGCTTGTGGGTGCAGAGATACATGGCTTTCACACGTT
GGATGATTTGGATGTTAGTAATACTATGGAGGAAGCAAAAAGCAGATGGCTTCGTCCCAATGAG
ATTCATGCCATACTCTGTAATCACAAATATTTCAAGATCAATGCCAAGCCAGTAAATTTGCCCAA
AAGTGGTACTATTGTACTATTTGACCGCAAGATGCTTAGGAACTTCAGAAAAGATGGGCACAATT
GGAAAAAGAAAACCGATGGAAAAACTGTTAAAGAAGCCCATGAACACTTAAAAGTTGGCAATG
AAGAAAGGATCCACGTATATTATGCACATGGCCAGGATAAACCAACCTTTGTTCGTAGATGTTAT
TGGTTGCTTGATAAGAGTCTGGAGCATATAGTCCTTGTTCATTATCGTGACACACAAGAGTTACA

ATTGCAAGGTTCTCCTGCTACACCTGTAAATTCAAATTCAAGTTCTGTCTCTGATTCAGCTGCATC
TTGGATTCCATCAGATGACTTGGATTCTGGGGTGAACAGTGCATATGCTGTTGAGCTTAATGACA
GTTTAACTGCCAAAAGTCATGAGCAGAGGCTTCATGAGATTAATACACTTGAATGGGATGACCTA
GTGGTTTCAAATGCCAACACTTCTACTACATCTAATGGAGGCAATGTTCCATATTCATTTCAACAA
AATCAAAGTTTACTGAATGGAAGCTTTGGCAATGTATCCAGTGATCCTTCAGCAGAAATTCCTTC
TTTTGGTAATTTAACTCAGCTAGTTTCTGGGAGCGATAGTGCTCCTTATAGTTTCCCAGAAAGTGC
TGATCTTTTGAAAAGTAGTCCTTTATCTTCGGGTGGTGTTGACACCTTGGGCACTTTGGTCAACGA
AGGCTTGCAAAGTCAGGACAGCTTTGGAACATGGATGAACATCATGTCGGATACACCATGTTCC
ATAGATGAATCAGCTCTTGAAGCAACAACTTCTTCTGTTCATGTACCATATTCATCACTGGTGGCA
GATAATAAGCAATCTTCTCTGCCAGAACAAGTATTTAATCTCACTGAGGTGTCCCCTGTGTGGGC
ATCTTCCACCGAGAAAACAAAGGTCCTGGTAACTGGATATTTTCATAATAACTATCAACACCTTG
CAAAGTCCAACCTACTGTGTGTCTGTGGTGATGTAAGTGTTCCTGTAGAAATTGTTCAGGTTGGAG
TTTACCGGTGCTGGGTATCACCACACTCTCCTGGATTGGTCACTCTCTATTTGAGCTTTGATGGCC
ATAAGCCCATCAGCCAAGTTGTTAATTTTGAGTATCGTACTCCTATTTTGCATGAGCCAACTGCTT
TAATAGAAGAGAAATACAACTGGGATGAGTTTCGACTTCAGATGAGGCTTGCTCATTTGCTCTTT
GCTTCAGACAAGAGCCTTAACATTTTCTCAAGTAAAGTATCAACTAATGCTCTGAAAGAGGCTAG
AAGATTTTCCTTCAAAACATCATATATCTCTAAAAGTTGGCAACACTTGATGAAGTCAATTGATG
ACAAAACAATTCCATTTTCACAAGTAAAAGATGCCTTGTTTGAAACTTCATTGAAAAACAAATTA
AAGGAATGGCTTTTGGAACGAATTATCTTAGGCAGCAAGAGTACTGAATATGATGCGCAAGGGC
AAGCTGCAATTCATTTGTGTGCAATGCTGGGATATAATTGGGCAATTTCCTTATTTACATGGTCGG
GCTTGTCGCTGGATTTCCGTGACAAATTTGGATGGACTGCTCTTCATTGGGCAGCATATTATGGAA
TAGAGAAAATGGTTGCAACTTTGTTATCTTGTGGGGCAAGGCCAAACTTGGTCACAGATCCAACT
CCTCAATATCCAGGTGGATGTACTGCTGCTGATCTTGCTTATGTGAAGGGATGTGATGGCTTAGCT
GCTTACCTTTCTGAGAAGTCTCTGGTTGAGCAGTTCAATGACATGAGCTTGGCTGGAAATATCAGT
GGCTCACTGGAAACTAGCTCAACTGATCCAGTAAATGCTGCGAACCTTACCGAGGACCAACTTT
ACCTAAAGGAGACTTTGGAAGCTTACCGTACAGCTGCCGAGGCAGCAGCACGTATTCAAGCTGC
ATTTAGGGAGCATTCATTTAAATTACGATATCAAGCAGTTGAAATTATGAGCCCAGAGGAAGAA
GCACGTCAGATAGTAGCAGCAATGAGGATTCAACATGCCTTTCGCAACTATGAGTCAAAGAAAA
AGATGGCAGCCGCTGCTCGGATTCAGCTTAGATTCCGTACATGGAAGTACCGTAGAGAATTTCTC
AATATGCGGCGTCAGGCTATTAAAATTCAAGCTGCTTTCAGGGGCTTTCAAGCACGGAAGCAATA
TCGGAAAATCGTATGGTCTGTTGGGGTGCTTGAGAAAGTGATCCTGCGTTGGCTTTTAAAGAGAA
AAGGCTTCCGTGGTCTCCAGGTTAATCCTGCCGAGGAGGAGACACAGGAAAGTGATACAATTGC
AGAGGAGGATTTCTTTCGCACCAGCCGGAAACAAGCCGAAGAACGTGTTGAGAGGTCTGTCATA
CGTGTGCAGGCCATGTTCCGTTCTAAGAAAGCACAAGAAGAATATAGAAGGATGAAGCTGACAC
ATAATCAAGCAATGCTGGATGAATTAGAAGAATTCCTAAATTCTGAAGATGACATGTTACCCAA
GACATAG

>Glyma05G28090 (GmCAMTA10)

ATGACACCTGGTTACGAATACGATATTAATGATCTGCATCAAGAGGCTCAAGCAAGATGGTTGA
AGCCTGCAGAAGTGATGTACATTTTACAGAATCATGAAAAGTTCCAGTTCACCCAAGAGCCCCC
ACAACAGCCAACCAGTGGATCTCTGTTTCTGTTTAACAAAAGAGTCCTGCGTTTCTTCCGTAAAG
ATGGTCATAACTGGCGTAAGAAAAGAGATGGAAGAACTGTGGGAGAAGCACACGAAAGGCTGA
AGGTTGGAAATGTTGAAGCCTTAAATTGTTACTATGCACACGGAGAGCAGAACCCTACTTTCCAG
AGGCGGAGCTATTGGATGTTGGATCCGGCATATGATCATATTGTTCTTGTGCATTATAGGAACAC
AAGTGAGGGAAAGCTCAGTTCTGGAGCTGGGGCACAATTGTCACCAAGTTCCTCTTCTGTGTATA
CTCAGAGTCCTAGCCCATATTCTACTCAGAATCCAGGGTCAACATCCATACTTGGCGATTCATAT
GAACCTAATCAGAGTTTCTCCAGTCCAGGGTCTACAGAAGTTACGTCTGATATGTTCGTACTGAA

CAATAAAATGGGTCACATGGATGGGACAGATACAGAATCAGGAACCTCGCCTGAACTCGAGGTT
ACTCAAGCTTTGCGCCGATTGGAGGTGCAGTTAAGTTTGAATGAGGACAACTTTGAAGATATTGT
TTCCTTCGGAAGTAAGCACGAAACTACACATGATTCAAACCCTCAGCACGACCAAAGGGTGATC
AGCAATCAAGAGCAATCTGCTGCTTTCTCTGGACCTGATGATCAAGGGCTATTTTATGATGGATA
TAATGGAAGGCAAGGTGATGGTGGTGAATTTTATCATGAATTAATAGACCATGGTTACCCTGATG
GAAATGAAAAGGCTTTATGGACAGAGGTGCTGGAATCATGTAAGTCCTCATCTGCAGTCAAGTT
ACCACAGAAAAATGTATACATGCCAGTTGAAAATCTGGAAAATTCAGTATCTTCTGCAAGAAGG
GTACCAGTTTCCAACCAGGAAAACAGTCACTGGCTCAACTTCAACAGTAATAATTCTGAAAACTC
TGTTTTCTCACAACCTCAAGGTGTTGATGAAGTCAAATTTCCTGTCTATTCTTCTATGGTGGAAAC
ACAAGTAATTAATTCTGACTACTATGAAACATTGTTTGACCAAAGCCAAATTGGAGCACCTCCAG
ATGCAAATTCAAGCTTGACTGTTGCACAAAAACAGAAATTTACAATTAAGACAATCTCCCCAGA
ATGGGGTTATGCCACTGAGACTACCAAGGTCATTGTTGTTGGGTCTCTTCTCTGTCATCCCTCGGA
TTCTGCATGGGCTTGTATGTTTGGTGATGTTGAAGTTCCTGTTGAGATAATTCAGGATGGTGTAAT
CTCTTGTGAAGCTCCATCTCACCTTCCTGGAAAGGTTACTCTGTGCATTACTTCTGGAAACCGGGA
GTCCTGCAGTGAGGTCAGAGAGTTTGAATATCGTGATAAGACCAATAGTTGCACTCAATGTACTC
AATCAGAAACAGAAGCCACTAGAAGTCCAGAAGAGCTGTTATTACTTGTTAGATTAGAGCAAAT
GCTTCTTTCTGCATCAACAATAAAGAATGACAACATAGAATCTGGAATTCCTCTCATAAAACAGA
AAGCTGATGATGACTCGTGGAGCCATATTATAGAGGCTCTTCTAGTTGGCAGTGGAACGTCGACT
GGTACTGTTGATTGGCTTCTTGAAGAGCTTCTAAAGGATAAGCTTCAACAGTGGCTTTCTTGCAGA
TCTCAGGAAAAAGATGAAGAGACAGGCTGCTCTTTGTCCAAGAAAGAGCAAGGGATTATTCACA
TGGTTGCTGGGTTAGGTTTTGAGTGGGCCTTGAACCCTATTCTCACTTGTGGAGTCAATATAAATT
TCCGTGACATCAATGGGTGGACTGCCCTTCATTGGGCTGCACGTTTTGGAAGGGAAAAAATGGTT
GCTTCACTTATAGCTTCTGGAGCATCTGCTGGAGCTGTGACAGATCCAAATGCACAAGATCCAAC
CGGTAAAACTGCTGCATCTATTGCAGCCGGCAATGGGCACAAGGGACTGGCAGGGTATCTTTCA
GAGATAGCTGTAACAAGCCATCTGTCATCTCTTACACTGGAAGAGAGTGAATTATCTAAAAGTTC
TGCTGAGCTTCAGGCAGATATGACTGTTAACAGTGTCTCCAAGGAAAATCTTACTGCCAGTGAGG
ATCAGGCATCACTCAAAGATACCTTGGCTGCTATCAGAAATGTAACTCAGGCAGCTGCGCGGAT
ACAATCTGCTTTTCGTTCACATTCTTTTAGAAAACGAAGAGCAAGAGAAGTAGCTGCTAGTGCAG
GTGGCATTGGTACCATTTCAGAGATTTCTGCTATGTCAAAACTTGCCTTTCGGAACTCACGTGAAT
ATAATTCAGCTGCTTCAGCTGCTTTATCGATTCAGAAGAAATATCGAGGTTGGAAAGGTCGCAAA
GATTTCTTAGCGTTGCGCAAAAAAGTAGTGAAGATACAGGCTCATGTGAGGGGCTACCAGGTTA
GGAAGCATTACAAGGTAATATGGGCAGTTGGAATCTTGGACAAGGTTGTCTTACGATGGAGGAG
AAAAGGAGCTGGTTTACGAGGTTTCCGACAAGAGATGGACATCAACGAAAATGAAAATGAAGA
TGAAGATATTCTCAAGGTGTTCCGAAAACAGAAAGTTGATGTAGAAATTGAAGAGGCTGTTTCA
AGGGTGCTGTCCATGGTCGACTCACCAGATGCTCGTGAGCAATATCATCGCATGCTTGAGAAATA
TCGTCAAGCTAAGGCTGAACTTGCGGGTACGAGTGACGAGGCATCATTATCAACTTCTGTAGGAG
ATGACTTATTTATTGATGATTTTTATCCATTTCCCTAG

>Glyma18G00840 (GmCAMTA11)

ATGCCACCTGGTTTGGAGTACAATATCGATGATCTGTTTCAAGAAGCTAAGAGGAGATGGCTCAA
GCCTGTGGAAGTGCTTTACATTTTACGGAATCACGACCAGTGCGAGTTCACCCACCAGCCCCCTC
ATCAGCCAGCTGGTGGATCCCTGCTTCTGTTTAATCGAAGAATCATGCGTTTCTTCCGTAAAGATG
GTCATAACTGGCGGAAGAAAAAAGATGGCAAAACTGTGGGAGAAGCGCATGAACGGCTTAAGG
TTGGCAATGTTGAAATCCTAAACTGTTACTATGCACATGGAGAGGAGAACCGTACTTTTCAGAGA
CGGAGCTATTGGATGTTGGAGCCGGAATATGACCATATTGTTCTTGTGCATTATAGAGAAACTAG
TGAGGGGAAATCCAAATCTGAACATGTCACACAATTGTCATCAGGTTCCTCTCCTGTTTTTAGTCA
GAGTCATAGCTCGTATACTACTCACAACCCAGGAACAGCATCCATGTTTGGTGATTCATGTGAAC

CTAATCAGAAATTTTCTAGTTCTGGGTCTTTAGAAGATACATCCGAAGCACAAGCCTTGCGCCAA
TTGGAAGAACAGCTGAGTTTGAATGAGGACATTTTCAATGAAATTGCACTTGATTTAATCCCAGG
TCAAGATCAAAGGGTGGTTTATAAGCAAGATAACTCTGTAGCTTTGTCTGGACCAAATGATCCAG
GACAACCTTGTGATGGATATAATGGAAGAGAAGATGACAGTGGTACATATTATCATGACTTTCTT
GATGATTGTCCTGGTGGAAATGAAAAAACTATATACTGGACTGAAGTGCTGGAATCGTGTAAGC
CCTTATCTGTGACCAAATTACCAGATCAACATGCATATGACGCGATTGAAAATGGAAAGTCATTA
TTTTCTTCAGGAAGAGGAATGATTGCCAACCGGGAAAAAAATCAGTGGCTAAACTCCAACAGTA
ATAATGTTGAAAACTCTGTTTTCTTGTTTCCTCAAGACATTGGAGTCAAATTTCCTCCATATTCTAT
GGTAGAAACTCCAGGAACTAACTATGACTACTATGAAACCTGTTTTGATCAATTCCAAAATCAAG
AACCTCTGGGTGTAGATTCAAGCTTCACTGTTGTGCAGAAACAGAAATTTACAATTAGGGCAGTT
TCCCCAGAATACTGTTATGCCACTGAGACTACAAAGGTGATCATTATTGGGTCATTTCTATGCCAT
GACTCGGATTCTACCTGGGCCTGTATGTTTGGTGATGTTGAAGTTCCTGCAGAGATAATTCAGGAT
GGTGTAATCTGTTGTGAAGCTCCATCTTATCTTCTTGGGAAGGTTAATTTGTGTGTTACTTCAGGAA
ATAGGGTGCCATGCAGTGAAGTGAGGGGGTTTGAGTTTCGAAATAAGACTACTAGTTGCACTCGC
TGTAATTCATTGGAAACAGAAGGCAGCAAAAGTCTAGAAGATCTGTTATTACTTGTTCGATTTGC
AGAGATGCTCCTTTCTGCTTCAACTACAAAGGATGACAGAATAGAATCTGGAAGTTATCTTTCAA
CAGAACAGAAAGATGATGATGATTCATGGAGCCATATTATTATAGACACTCTTCTAGATGGCACC
AGAACATCATCTGATACTGTTAACTGGCTTCTTGAAGAGCTGCTGAAGGATAAGTTGCAGCTCTG
GCTTTCTAACCGAAGAGATGAAGGGACAGGCTGTTCTTTTTCCAGGAAAGAACAGGGGATAATA
CACATGATTTCTGGGTTGGGTTTTGAGTGGGCCTTGAGCCCCATTCTTAGTTGTGGTGTGAATATA
AATTTCCGTGACATCAATGGGTGGACCGCTCTTCATTGGGCTGCTAGGTTTGGGAGGGAAAAAAT
GGTTGCTTCGCTTATAGCTTCTGGTGCATCTGCTGGAGCAGTGACAGATCCAAGTTCACAAGATC
CAACAGGTAAAACTGCTGCATCTATTGCAGCTAGCCATGGCCATAAGGGACTGGCAGGTTATCTT
TCAGAGGTAGACCTAACAAGCCATCTGTCATCCCTCACATTGGAAGAGAGTGAGCTTTCTAAAG
GATCTTCTGAGCTTGAAGCCGAGTTAACTGTCAGTAGTGTCTCTAAAGAAAATCTTGTGGCCAGT
GAGGATCAGGTTTCACTACAAGCTTTCCTGGATGCTGTTAGAAATGCAGCTCAGGCAGCTGCACG
GATACAAGCTGCTTTTCGTGCACATTCTTTTAGAAAACGGAAAGAAAGAGAAGCTGCTGCTGAC
GCTGGTCTAGATGGATATTGTATTGATGCAGGTAGCATTGATAATAACATTTCAGTGCTTTCTGCC
GTGTCAAAACTTAGTTCTCAGAGCTGTCGTGATTACAATTTAGCTGCCTTATCGATTCAGAAGAA
ATATCGAGGCTGGAAAGGTCGTAAAGAATTCTTAGCATTGCGCCAGAAAGTAGTTAAGATACAG
GCTTGTGTAAGGGGATACCAGGTTCGGAAGCAATACAAGTTAATATTATGGGCAGTTGGAATCTT
GGACAAGGTTGTGCTACGATGGCGCAGAAAACGAATTGGTATACGAAGTGTCCGGCAAGAAAT
GGAATCAAATGAAGAAGAAAGTGATGATGAAGATTTTCTCAGCGTGTTCCGGAAAGAGAAAGTA
AATGCAGCAATTGAAAAGGCTTTGAAGCAGGTGCTTTCCATGGTCCATTCCTCTGGTGCTCGTCA
GCAATATAGGCGCTTGCTTTTGTTGTATCGTCAAGCCAAGGCCAAGACTGAACGTGGCAGCACGA
GTGATGAAGCACCCTTATCAACTTCGGAAGAGGAAGTTTCCAATATGGAAGATGATGATTTGTGT
CAATTTTGGGAAACGTTCTGGCCTTCCTAG

>Glyma17G03510 (GmCAMTA12)

ATGGCGAATAACTTAGCGGCGCAACAACTGGTTGGTTCAGAGATACATGGATTCCACACATTGC
AAGATTTGGATGTGGGGAGCATTATGGAGGAAGCAAGAACCAGATGGCTCCGTCCCAATGAGAT
TCATGCCATGCTTTGTAATTACAAGTATTTTACCATTAATGTCAAGCCAGTGAATTTGCCAAAAAG
TGGTACTATTGTCTTATTTGATCGCAAGATGCTTAGGAACTTCCGTAAAGATGGGCACAACTGGA
AAAAGAAAAAGGATGGGAAAACCGTTAAAGAAGCTCATGAGCACCTAAAAGTTGGTAATGAAG
AAAGGATCCATGTATATTATGCACATGGCCAGGATAACCCAAATTTTGTCCGAAGATGTTATTGG
CTGCTTGATAAGAGTATGGAACACATAGTCCTTGTTCATTACCGTGAAATACAGGAGATGCAAGG
TTCTCCCGTCACTCCTGTAAATTCACACTCCAGTTCTGTCTCTGATCCTCCTGCACCATGGATTTTA

TCAGAAGAAATAGATTCAGGAACCACAACTGCATATGCTGGTGACACGAGTGCCAATATAAATG
TCAAAAGTCATGAACTCAGACTTCATGAGATTAATACACTTGATTGGGATGACCTGGTGGATGCA
AATGATCATAACACAACTACTGTGCCTAATGGAGGTACTGTTCCATATTTTGATCTACAAGATCA
AATTTTACTGAATGACAGTTTCAGCAATGTAGCCAACAATCTATCTGCAGACATTCCTTCTTTTGG
TAGTCTCACTCAGCCTATTGCTGGGAGCAACAGTGTTCCTTACAATTTTTCAAGTGTTAATCTTCA
GACGATGGACGATCAAGCAAATCCACATGAGCAGAGGAATAATACAGTTTCCTTAAGTGGTGTT
GATTCCTTGGACACTTTGGTCAATGATAGATTGCAAAGTCAGAACAGCTTTGGAATGTGGGTAAA
CCCCATCATGTCTGATTCACCATGTTCAGTAGATGACCCAGCTCTTGAATCCCCAGTTTCATCCGT
TCATGAACCATATTCATCTCTGATAGTGGATAGTCAGCAATCTTCTCTGCCAGGACAAGTATTTAC
TATCACTGATGTATCCCCTACATGCGTATCTTCCACTGAGAAATCAAAGGTCCTGGTGACTGGATT
TTTTCATAAGGACTATATGCACCTTTCAAAGTCCAATCTATTATGTGTCTGTGGTGATGTTAGTGTT
CCTGCAGAAATTGTTCAGGTTGGGGTCTACCGGTGCTGGGTATCACCACATTCTCCAGGATTCGT
GAATCTCTACATGAGCATTGATGGCCATAAGCCTATTAGCCAAGTTGTTAATTTTGAATATCGTAC
TCCTGCTTTGCATGATCCCGCCGTTTCAATGGAAGAGAGTGACAATTGGGATGAGTTTCAACTAC
AAATGAGGCTTGCATATTTGCTCTTTAAACAATTGAACCTTGATGTTATCTCCACTAAAGTATCAC
CCAATAGACTTAAAGAGGCTAGACAATTCGCCCTCAAAACATCATTTATTTCCAATAGTTGGCAA
TACTTAATCAAGTCAACTGAAGACAACCAAATTCCATTTTCACAAGCAAAAGATGCTCTATTCGG
AATTGCATTGAAAAGCAGACTAAAGGAATGGCTTTTGGAAAGAATTGTTTTAGGCTGCAAGACT
ACTGAATATGATGCTCATGGCCAAAGTGTAATCCATTTGTGTGCAATTTTGGGATATACTTGGGCT
GTTTCCTTGTTTTCATGGTCAGGCTTGTCATTGGATTTCCGTGATAGGTCTGGATGGACAGCTCTTC
ATTGGGCAGCATATTGTGGAAGGGAGAAAATGGTTGCAACTCTGTTGTCTGCTGGGGCGAAGCC
AAATTTGGTCACTGATCCTACTCCCCAAAATCCTGGTGGATGCACTGCTGCTGATCTTGCTTATAT
GAGGGGTCATGATGGCTTAGCAGCTTATCTTTCAGAAAAATCTCTGGTTCAGCACTTCAATGACA
TGAGCTTAGCTGGAAATATCAGCGGCTCACTTGAAACTAGCACGACTGATCCGGTAATTTCTGCA
AACCTTACGGAGGACCAGCAGAACCTGAAGGATACATTGGCAGCTTACCGAACTGCTGCTGAGG
CAGCTTCCCGCATACATGCTGCATTTAGGGAACACTCTCTAAAACTGAGAACTAAGGCGGTTGCA
TCTTCCCATCCAGAGGCTCAAGCACGCAAAATAGTTGCAGCAATGAAGATTCAGCATGCCTTTAG
GAACCACAAGACAAAGAAAGTGATGGCAGCTGCTGCTCGCATACAGTGTACATACCGTACATGG
AAGATTCGTAAAGAATTTCTCAACATGCGTTGTCAGGCTGTTAAAATTCAAGCTGCGTTCCGGTG
CTTTCAAGTGCGGAAGCATTATTGCAAAATTCTTTGGTCTGTTGGTGTCGTTGAGAAAGCAGTGCT
GCGTTGGCGTTTGAAGAGAAGGGGTTTCCGTGGGCTCCAGGTCAAAACTGTCGAAGCGGGAACT
GGAGATCAGGATCAGCAAAGTGATGTAGAGGAGGAATTCTTCCGGGCTGGTAGGAAACAAGCT
GAAGAACGTGTTGAGAGATCTGTGGTACGTGTGCAGGCTATGTTCCGTTCGAAGAAAGCACAAG
AAGAGTACAGGAGGATGAAATTGGCCCTTGATCAAGCAAAGCTTGAGCGAGAATTCGAACGACT
TCTTAGTACTGAAGTTGACATGCAACTGAAGACATAG

>Glyma07G37090 (GmCAMTA13)

ATGGCGAATAACTTAGCGGTGCAACTGGTTGGTTCAGAGATGCATGGATTCCACACATTGCAAG
ATTTGGATGTGGGGAGCATTATGGAGGAAGCAAGAACCAGATGGCTCCGTCCCAATGAGATTCA
TGCCATGCTCTGTAATTACAAGTATTTTACCATTAATGTCAAGCCAGTGAATTTGCCAAAAAGTG
GTACTATTGTCTTATTTGACCGCAAGATGCTTAGGAACTTTCGAAAAGATGGGCACAACTGGAAA
AAGAAAAAGGATGGGAAAACTGTTAAAGAAGCTCATGAGCACTTAAAAGTTGGTAATGAAGAA
AGGATCCATGTATATTATGCACATGGCCAGGATAACCCAAATTTTGTCCGAAGATGTTATTGGCT
GCTTGATAAGAGTATGGAACACATAGTCCTTGTTCATTACCGTGAAACACAGGAGATGCAAGGTT
CTCCTGTCACTCCTGTAAATTCACACTCCAGTTCTGTCTCTGATCCTCCTGCACCGTGGATTTTATC
AGAAGAAATAGATTCAGGAACCACAACTGCATATACTGGTGACATGAGTAACAATATAAATGTC
AAAAGTCATGAACTCAGACTTCATGAGATTAATACACTTGAATGGGATGACCTAGTGGACACAA

ATGATCATAACGCCTCTACTGTGCCTAATGGAGGCACTGTTCCATATTTTGATCAACAAGATCAA
ATTTTACTGAATGACAGTTTCGGCAATGTAGCCAACAATCTATCTGCAGAAATTCCTTCTTTTGGT
AATCTTACTCAGCCAATTGCTGGGAGCAACAGAGTTCCTTACAATTTTTCTGAAAGTGTTACTCTT
CAGACGATGGACAATCAAGCAAATCCACATGAGCAGAAGAATAATACAGTTTCCTTAAGTGGTG
TTGATTCCTTGGACACTTTGGTCAATGATAGATTGCAAAGTCAGGACAGCTTTGGAATGTGGGTG
AACCACATCATGTCTGATTCACCATGTTCAGTAGATGACCCAGCTCTTGAATCACCAGTTTCATCC
ATTCATGAACCATATTCGTCTCTGGTAGTGGATAGTCAGGAATCTTCTCTGCCAGAACAAGTATTT
ACTATCACTGATGTATCCCCTACATGTGTATCTTCCACCGAGAAATCAAAGGTCCTGGTGACTGG
ATTTTTTCTTAAGGACTATATGCACCTTTCAAAGTCCAATCTATTATGTGTCTGTGGTGATGTTAGT
GTTCCTGCAGAAATTGTTCAGGTTGGGGTCTACCGTTGCTGGGTATCACCACACTCTCCAGGATTT
GTGAATCTCTACTTGAGCATTGATGGCCATAAGCCTATTAGCCAAGTTGTTAATTTTGAATATCGT
ACTCCTGCTTTGCATGATCCTGCAGTTTCAATGGAAGAGAGTGACAATTGGGACGAGTTTCGACA
ACAAATGAGGCTTGCATATTTGCTCTTTGCTAAACAATTGAACCTTGATGTTATCTCCAGTAAAGT
ATCACCTAATAGACTTAAAGAGGCTAGGCAATTCGCCCTCAAAACATCATTTATTTCCAATAGTT
GGCAATACTTAATCAAGTCAACTGAAGACAACCAAATTCCATTTTCACAAGCAAAAGATGCCCT
GTTCGGAATTACATTGAAAAACAGACTAAAGGAATGGCTTTTGGAAAGAATTGTTTTAGGCTGCA
AGACTACTGAATATGATGCTCATGGCCAAAGTGTAATCCATTTGTGTGCAATTTTGGGATATAATT
GGGCTGTTTCCTTATTTTCATGGTCAGGCTTGTCATTGGATTTCCGTGATAGGTTTGGATGGACAGC
TCTTCATTGGGCAGCATATTGTGGAAGGGAGAAAATGGTTGCAACTCTGTTGTCTGCTGGGGCGA
AGCCAAATTTGGTCACTGATCCTACTCCACAAAATCCTGGTGGATGCACTGCTGCTGATCTTGCTT
ATATGAGGGGTCATGATGGCTTAGCAGCTTATCTTTCAGAAAAATCTCTGGTTCAGCACTTCAAT
GACATGAGCTTAGCTGGAAATATCAGTGGCTCACTGGAAACTAGCACGACTGATCCAGTAAATC
CTGCAAACCTTACCGAGGACCAGCAGAACCTGAAGGATACGTTGACAGCTTACCGAACTGCTGC
TGAGGCAGCTTCCCGCATACATGCTGCATTTAGGGAACACTCTCTAAAACTGAGAACTAAGGCG
GTTGCATCTTCCAATCCAGAGGCTCAAGCACGCAAAATAGTTGCAGCAATGAAGATTCAGCATG
CCTTTAGGAACCACGAGACAAAGAAAATGATGGCAGCTGCCGCTCGCATTCAGTGTACATACCG
TACATGGAAGATTCGTAAAGAATTTCTTAACATGCGTCGTCAGGCTGTTAAAATTCAAGCTGCAT
TCCGGTGCTTTCAAGTTCGGAAGCATTATCGCAAAATTCTTTGGTCTGTTGGTGTGGTTGAGAAAG
CAGTGCTGCGTTGGCGTTTGAAGAGAAGGGGCTTCCGTGGTCTCCAGGTCAAAACTGTCGATGCG
GGAACCGGAGATCAGGATCAGCAAAGTGATGTAGAGGAGGAATTCTTCCGGACTGGTAGGAAG
CAAGCCGAAGAACGTGTTGAGAGATCTGTAGTACGTGTGCAGGCTATGTTCCGTTCGAAGAAAG
CACAAGAAGAGTACAGGAGGATGAAATTGGCCCTTAATCAAGCAAAGCTGGAGCGGGAATACG
AACAACTTCTTAGTACTGAAGTGGACATGCAACTGTAG

>Glyma11G36930 (GmCAMTA14)

ATGGCAGGTTTGGAGTACAGCATCGATGATCTGTTTCAAGAAGCTAAGAGGAGATGGCTCAAGC
CTGTGGAAGCTCTTTACATTCTACGGAATCACGACCAGTGCAAGTTCACCCACCAGCCCCCTCAT
CAGCCAGCTGGTGGATCCCTGTTTCTGTTTAATCGTAGAATCATGCGTTCCTTCCGTAAAGATGGT
CATAACTGGCGGAAGAAAAAAGATGGCAAAACTGTGGGAGAAGCGCATGAACGGCTTAAGGTT
GGCAATGTTGAAATCCTAAACTGTTACTATGCACATGGAGAGGAGAACCGTACTTTTCAGAGAC
GGAGCTATTGGATGTTGGAGCCGGAATATGATCATATTGTTCTTGTGCATTATAGAGAAACAAGT
GAGGGGAAATCCAATTCTGAGCATGTCACACAATTGCCATCAGAAGTTACGTTCGAAGCACAAG
CCTTGCGCCAATTGGAAGAACAGCTGAGTTTGAATGATGATGGCTTCAATGAAATTGCACTTGAT
TTAGTGTCAGGTCAAGATCAAAGGGTGGTTTATAAGCAAGATAAATCTGCAGCTTTGTCTGGACC
AAATGATCTAGGACAACCTTGTGATGGATATAATGGAAGACAAGATGACAGTGGCACATATTAT
CATGATTTTCTTGATGATTGTCCTGGTGGAAATGAAAAAACTATATACTGGACGAAAGTGCTGGA
ATCGTGTAAGCCCTTATCTGTGACCAAATTACCAGATCAACATGCATATGAGGCGATTGGAAATG

AAAATACATTATTTTCTTCGGGAAGGGGAGTGATTGCCAACCTGGAAAACAATCAGTGGCTAAA
CTCCAACAGTAATAATATTGAAAACTATGGTGGGGTCAAATTTCCACCATATTCTCTAGCAGAAA
CTCCAGGAGCTAACTCTGACTACTATGAAACATTTTTTGACCAATTCCAAAATCAAGGACCTCTG
GGTGTAGATTCAAGCTTGACTGTTGTGCAGAAACAGAAATTTACCATTAGGGCAGTATCCCCAGA
ATACTGTTATTCTACCGAGACTACAAAGGTGATCATTATTGGGTCATTTCTCTGCCATGACTCGGA
TTCTACCTGGGCCTGTATGTTTGGTGATGTTGAAGTTCCTGCAGAGATAATTCAGGACGGCATAAT
CTGTTGTGAAGCTCCATCCAATCATCTTGGAAAGGTTAATTTGTGCATTACTTCAGGAAATAGGGT
GCCATGCAGCGAAATGAGGGAGTTTGAGTTTCGAAATAAGACTACTAGTTGCACTCGCTGTAATT
CATTGGAAACAGAAGGAAGCAAAAGTCCTGAAGATCTGTTATTACTTGTTCGATTTGCAGAGATG
CTCCTTTCTTCTTCAACTACAAAGGATGACAGAATAGAATCTGGAAGTCATCTTTCAACAGAACA
GAAAGATGATGATGATTCGTGGAGCCATATTATAGACACTCTTCTAGATAGCACTAGAACACCAT
CTGATGCTGTTAAATGGCTTCTTGAAGAGCTGCTGAAGGATAAGTTGCAGCTCTGGCTTTCTAACC
GAAGAGATGAAGGGACGGGCTGTTCTTTGTCCAAGAAAGAGCAGGGGATAATACACATGGTTTC
TGGGTTGGGTTTTGAGTGGGCCTTGAACCCCATTCTCAGTTGTGGTGTGAATATAAATTTCCGTGA
CATCAATGGATGGACCGCTCTTCATTGGGCTGCACGGTTTGGGAGGGAAAAAATGGTTGCTTCAC
TTATAGCTTCTGGTGCATCTGCTGGAGCAGTGACAGATCCAAGTTCACAAGATCCAACCGGTAAA
ACTGCTGCATCTATTGCAGCCAGCCATGACCATAAGGGTCTGGCAGGTTATCTTTCAGAGGTAGA
CCTAACAAGCCATCTGTCGTCCCTCACATTGGAAGAGAGTGAGCTTTCTAGAGAATCTTCTGAGC
TTGAAGCTGAGTTAACTGTCAGTAGTGTCTCTGAAGAAAATCTTGTGGCCAGTGAGGATCAGGTT
TCACTAAAAGCTTCCTTGGATGCTGTTAGAAATGCAGCTCAGGCAGCTGCACGGATACAAGCTGC
TTTTCGTGCACATTCTTTTAGAAAACGGAAAGAAAGAGATGCTGCCGCCACAGTTCTAGATGGAT
ATTGTATTGATGCAGGTAGCATTGATAATAACATTTCAGTGCTTTCTGCCATGTCAAAACTTAGTT
CTCAGAGCTGGCGTGATTACAAAGCTGCTTTGTCGATTCAGAAGAAATATCGAAATTGGAAAGG
TCGTATAGAATTCTTAGCATTGCGCCAGAAAATAGTTAAGATACAGGCTTGTGTGAGGGGTTACC
AGGTTCGGAAGCAATACAAGCTAATATTATGGGCAGTTGGAATCTTGGACAAGGTTGTGTTACGA
TGGCGGAGAAAACGAATTGGTATACAAAGTGTCCGGCAAGAAATGGAGTCAAATGAAGAAGAA
AGTGATGATGCAGATTTTCTCAATGTGTTCCGGAAAGAGAAAGTAAATGCAGCAATTGAAAAGG
CCTTGAAGCGGGTGCTTTCCATGGTCCACTCCACTGGTGCTCGTCAGCAATATAGACGCTTGCTTT
CGTTGTATCGTCAAGCCAAGATTGAACATGGCAGCACAAGTGATGAAGCACCGTTATCAACTTC
GGAAGAGAATGCTTCCAATATGGAAGATGATGATTTGTGTCAATTTTTGGACACCTTCTTCTGGCC
TTCCTAG

>Glyma08G11080 (GmCAMTA15)

ATGACACCTGGTTACGAGTATGATATTAATGATCTGCATCAAGAGGCTCAAGCCAGATGGTTGAA
GCCTGCAGAAGTGATGTACATTTTACAGAATCATGAAAAGTTCCAGTTCACCCAAGAGGTCCCAC
AACAGCCAACCAGTGGATCCCTGTTTCTGTTTAACAAAAGAATCCTGCGATACTTCCGTAGAGAT
GGTCATAACTGGCATAAGAAAAGTGGTGGAAGAACTGTGGGAGAAGCACACGAAAGGCTGAAG
GTTCTAAATGTTGAAGCCTTAAATTGTTACTATGCACGCGGAGAGCAGAACCCTGCTTTCCAGAG
GCGGAGCTATTGGATGTTGGATCCGGCATATGAGCATATTGTTCTTGTGCATTATAGGAACACAA
GTGAGGGAAAGCTAAGTTCTGGAGCTGGGGCACAATTGTCACCAAGTTCTTCTGTCTATACTCAG
AGTCCTAGCCCATATTCTACTCAGAATCCAGGGTCAACATCCATACTTGGTGATTCATATGAACC
TAATCAGAGTTTCTCCAGTCCTGGGTCTACAAAAGTTACATCTGAGATATTCGTTCTGAACAATA
AAATGGGTCACATGGATTGGGCAGATACAGAATCAGGAACCTCATCTGAACTCGAGGTTACTCA
AGCTTTGCGCCGATTGGAGGTACAGTTAAGTTTGAATGAGGACAACTTTGAAGATATTGTTTCCTT
CGGCAGTAAGCATGAAACTGTTCATGATTCAAATCCCAAGCACGATCAAAGGGTGATCAGCAAT
CAAGAGCAATCTGCAGCTTTCTCTAGACCCGATGATCAAGGGCTATTTTATGATGGATGTAATGG
AAGGCAAGATCATGGTTACCCTGATGCAAATGAAAAGGCTTTATGGACAGAGCAGCTGGAATCA

CATAAGTCCTCATCTGCAGTCAAGTTACCTCAGAAAAATGTATACATGCCAGCTGAAAATGAAA
ATTCAGTATCTTCTGCAAGAAGGGTACCAGTTTCCAACCAGGAAAACAGTCACTGGCTCAACTTC
AACTGTAATAATTCTGAAAACTCTGTTTCTCACAACCTCAAGGTGTTGATGAAGTCAAATTTCCT
GCCTATTCTTCTATGTTGGAAACACAAGTAATTAATTCTGACTACTATGAAACATTGTTTGACCAA
AGCCAAATTGGAGCACCACCAGATGCAAATTCAAGCTTGACTGTTGCACAAAAACAGAAATTTA
CTATTAAGACTATCTCCCCAGAATGGGGTTATGCCACCGAGACTACCAAGGTCATTGTTGTTGGG
TCTTTTCTCTGTCATCCCTCGGATTCTGCATGGGCTTGTATGTTTGGTGATGTTGAAGTTCCTATTG
AGATAATTCAGGATGGTGTAATCTCTTGTGAAGCTCCATCTCACCTTCCTGGAAAGGTTACTCTGT
GCATTACTTCTGGAAACTGGGAGTCCTGCAGTGAAGTCAGAGAGTTTGAATATCATGATAAGACC
AATAGTTGCACTCGATGTACTCAATCCGAAACAGAAGCCACTAGAAGTCCAGAAGAGCTGTTAT
TACTTGTTAGATTAGGGCAGATGCTTCTTTCTGCATCAACAATAAAGAATGACAACATAGAATCT
GGAATTCCTCTAATAAAACCGAAAGCTGATGATGATTCATGGAGCCATATTATAGATGCTCTTCT
AGTTGGCAGTGGAACGTCGAGTGGTACTGTTGATTGGCTTCTTGAAGAGCTTCTAAAGGATAAGT
TTCAACAGTGGCTTTCTTTCAGATCACGGGAAAAAGATGAAGAGACAGGCTGCTCTTTGTCCAAG
AAAGAGCAAGGGATTATTCACATGGTTGCTGGGTTAGGTTTTGAGTGGGCCTTGAACCCTATTCT
CACGTGTGGAGTTAATATAAATTTCCGTGACATCAATGGATGGACTGCCCTTCATTGGGCTGCAC
GTTTTGGAAGGGAAAAAATGGTTGCTTCACTTATAGCTTCTGGAGCATCTGCTGGAGCTGTGACA
GATCCAAATGCACAAGATCCAACTGGTAAAACTGCTGCATCTATTGCAGCCAGCAGTGGGCACA
AGGGACTGGCAGGGTATCTTTCAGAGATAGCTGTAACAAGCCATTTGTCATCGCTTACACTGGAA
GAGAGTGAATCATCTAAAAGTTCTGCTTATCTTCAGGCGGATAGGACTGTTAACAGTGTCTCCAA
GGAAAATCTTACTGCCAATGAGGATCAGGCGTCACTCAAAGATACCTTGGCTGCTATCAGAAAT
GTAACTCAGGCAGCTGCGCGGATACAATCTGCTTTTCGCTCACATTCTTTTAGAAAACGGAGAGC
AAGAGAAGCAACTGCTAGTACAGGTGGCATTGGTACCATTTCAGAGATTTCTGCTATGTCAAAAC
TTGCGTTTCGGAACTCACACGAATATAATTCAGCTGCTTTATCCATTCAGAAGAAATATCGAGGT
TGGAAAGGTCGTAGAGATTTCTTAGCATTGCGCCAAAAAGTAGTGAAGATACAGGCTCATGTGA
GGGGTTACCAGGTTAGGAAGCATTACAAGGTAATATGGGCAGTTGGAATCTTGGACAAGGTTGT
CCTACGATGGAGGAGAAAAGGAGCTGGTTTACGAGGTTTCCGACAAGAGATGGACATCAATGA
AAATGAAGATGAAGATATTCTCAAGGTGTTCCGAAAACAGAAATTAGATGTAGAAATTGAAGAG
GCTGTTTCAAGGGTGCTGTCCATGGTTGACTCACCGGATGCTCGTGAGCAATATCATCGCATGCTT
GAGAAATATCGTCAAGCTAAGGCTGAACTTGCGGGTACGAGTGACGAAGCATCATTATCAACTT
CTGTAGGAGATGACTTATTTATGGATGATTTTTATCCATTTCCCTAA

Proteomic sequences of Soybean CAMTA family

>Glyma.05G178200 (GmCAMTA1)

MAEARHYVPPSQLDIKQIILEAQHRWLRPAEICAILSNHKKFLIASEPAHMPPSGSLFLFDRKVLRYFRK
DGHNWRKKKDGKTVREAHERLKAGSVDVLHCYYAHGEENENFRRRTYWLLEEELSHIVLVHYRHVK
GTKANFTCAKENEETLPYAQQTDKIMPKTEMETSLSSTLHPHSYQVPSQTMDRSMNSSQASEYEEAESA
FNNHASSEFYSFLELERPVEKITPQPADSYSPRPLTRKSVPNMNCIIESGTDDQEKSPVIPGVNYISLTQDN
KIKDIHNFGLTYESPKPLGFSSWEGILKNNAGSQHVPFQPLFPGTQPDNMGINSKFSQGHEIMVPYLTTS
IAKQHENGSLIQAEGNWQAYDVDSLRMSSWPIDSAYSGSSCDITCSNREQEVNDVDLQKSLEQCLLHP
YKQNKVFMQNDPQEKLLNEKEKIKSDLEANRILDGIEDTYFTFKRTLLDGSPAEEGLKKLDSFNQWMS
KELADVEESNKPSTSGGYWDTVESENEVGNTTIPSQGHLDTYVLDPSVSHDQLFSIIDYSPSWAFEGSEIK
VIISGRFLRSQHEAEQGKWSCMFGEVEVPAEIIAKGVLCCHTPPHKAGRVPFYVTCSNRLACSEVREFDF
QVNYTPEVNTTGENRGSTFDTFSIRFGELLSLGHAFPQNSDSISVSEKSQLRSKINSLLREEEDDWDKLLK
LTQEEDFSPENLQEQLLQNLLKDKLHAWLLQKITEEGKGPNILDEGGQGVLHFASALGYDWALEPTIV
AGVNVNFRDVNGWTALHWAAFCGRERTVAFLISLGAAPGALTDPCPEHPSGRTPADLASANGHKGI
AGYLAESSLSAHLTTLDLNRDAGENSGAKVVQRVQNIAQVNDLDGLSYELSLKDSLAAVRNATHAAA
RIHQVFRMQSFQRKQLKEYDDDKLGLSDERALSLVKMNMKSHKSGPRDEPVHAAAVRIQNKFRSWK
GRREFLMIRQRIVKIQAHVRGHQVRKSCGKIIWSVGILEKVILRWRRKGSGLRGFKPEANSEGTMIQDVS
STDDDYDVLKEGRKQTEQRLQKALARVKSMVQYPEARDQYHRLLNVVTEIQENQVKHESSYNNSEEP
REFGDLNDLEALLDEDIFMPTAT

>Glyma.08G135200 (GmCAMTA2)

MAEARLYAPPSQLDIKQIILEAQHRWLRPAEICAILGNYKKFRIAPEPAHMPPSGSLFLFDRKVLRHFRK
DGHNWRKKKDGKTVREAHERLKAGSVDVLHCYYAHGEENENFQRRTYWLLEEELSHIVLVHYRQVK
GTKANFTSAKENEESLPYAQQTDKIMPQTEMDTSLSSTLHPHSYQVPSKTVDTSMNSAQTSEYEEAESA
FNNHASSEFYSFLELQRPVEKISPQPADFYSPRPLINDQEKLPIIPGVNYISLTQDNKNKDILNAGLTYESP
KPLGFSSWEGILENNAGSQHVHFQPLFPGTQPDNMGINSNFSQGEEIMVPYLTTSIAKQHENGSIIKAEG
NWQVYDVDSLRMSSWPIDSAYSGSTCEVSCSNCEQEVNDVDFQKSLEQCLLHSHKQNKVLMQNDLQ
EKLLNEKEKIKSNLEAYGIEDTYLSFKRTLLDGPPAEEGLKKLDSFNQWMSKELGDVEESNKPSTSGGY
WDTVETENEVGNTTIPSQGHLDTYVLDPSVSHDQLFSIIDYSPSWAFEGSEIKVIISGEFLRSQHEAEQCK
WSCMFGEVEVPAVIIAKGVLCCHTPPHKAGRVPFYVTCSNRLACSEVREFDFQVHYTPEDTTGENRGST
FDTFSIRFGELLSLGHAFPQNSDSISVSEKSQLRSKINSLLREDDDDWDKLLKLTQEKDFSPENLREQLLQ
NLLKDKLHAWLLQKITEEGKGPNVLDEGGQGVLHFAAALGYDWALEPTIVAGVNVNFRDVNGWTSL
HWAAFCGRERTVAFLISLGAAPGALTDPCPEHPSGRTPADLASANGHKGIAGYLAESSLSAHLTTLDL
NRDAGENSGAKVVQRLQNIAQVNDLDGLSYELSLKDSLAAVCNATQAAARIHQVFRMQSFQRKQLK
EYDDDKLGLSDERALSLIKMNVKSHKSGPRDEPVHAAAIRIQNKFRSWKGRREFLMIRQRIVKIQAHVR
GHQVRKSCGKIIWSVGILEKVILRWRRKGSGLRGFKPEANSEGTMIQDVSSTDDDYDVLKEGRKQTEQR
LQKALARVKSMVQYPEARDQYHRLLNVVTEIQENQVKHESSSNNSEEPREFGDLNDLEALLDEDIFMP
TAT

>Glyma.15G053600 (GmCAMTA3)

MAEGASYGLRRPLDIQQLQFEAQHRWLRPAEICEILRNYRMFHITSEPHNRPPSGSLFLFDRKVLRYFRK
DGHNWRKKKDGKTVKEAHEKLKVGSVDVLHCYYAHGEENENFQRRSYWMLEPDMMHIVFVHYLEV
KGNKNIVVNNEGDEVPTDSQKVTSPSSSLPTHHSCVSSLSTDSVSPTTSLMSLHEDADSEDIHHASSGLH
PLHESQHSGNSPLTEKIGAGSNSSYLMHPFSGDNEQSSISGTDYVPVVHGDKFRGNDTAYTDGQKPHG
MAPWGTVLQSTAKLHNDPSLASFPSILPSSMGDVLEQEHTIFGDLLMSKSGLTEEAESSQSLQSNWQIPF
EDNSGGMPMLTQTQSFGLQFRSDYGTGLLGNETRNASSEIAPILYSFHGEPKEQPMQQNYPQELEDGQS

QHALKSNSANKVPDEETINYGLTVKSTLLDRDESLKKVDSFSRWITKELGEVADLNMQSSPGISWSTDE
CQHVIDDTSLSPSLSQDQLFSINDFSPKWAYAESEIEVLIIGSFLKSQPEVTTCNWSCMFGEVEVPAEVLA
DGILCCQAPCHKVGRVPFYVTCSNRLACSEVREFDFREGFARNVDFADFYISSTEMLRHLRLEDFLSLKP
VDPSNHSFEGDMEKRNLIFKLISLREEEDYSIKDEVTRELDISQHMVKEHLFHRQFKEKLYSWLLHKVTE
NGKGPNVLDEDGQGVLHLAAFLGYDWAINPIISAGVNINFRDVNGWTALHWAASCGRERTVAVLVS
MGADCGALTDPSPAFPSGRTAADLASSYGHKGISGFLAESSLTHHLETLTMDDQKGGQQEISGMKVVQ
TVSERSATPVHYCDIPDAICLKDSLTAVRNATQAADRIHQVYRMQSFQRKQLTQYEGDDELGLSDQQA
LSLLASRACKSGQGDGLANAAAVQIQKKFRGWKKRKEFLMIRQRVVKIQAHVRGHQIRKQYKPIIWSV
GILEKVILRWRRKGSGLRGFRPNAINKVPNQQNDSLKEDDYDYLKEGRKQKEEKIQKALSRVKSMVQY
PEARAQYRRLLNVVEDFRQTKASNKGLINSEETVDGVEDLIDIDMLLDDDNFIPIAFD

>Glyma.08G072100 (GmCAMTA4)

MSERSSFGLGPRLDLQQLQLEAQHRWLRPAEICEILRNYRMFQITSEPPNRPPSGSLFLFDRKVLRYFRKD
GHNWRKKKDGKTVKEAHEKLKVGSVDVLHCYYAHGEENENFQRRSYWMLELDMMHIVFVHYLDV
KVNKTNIGGKTYSDEVTSDSQKSSSLSSGFPRNYGSMPSGSTDSMSPTSTLTSLCEDADSEDIHQASSGLH
SYRESQNLGNDRPMDKIHARSNSSYLMHPFSDNHGQLPVSGAEYIPHVQGNKSRASDTTYIEGQRAHG
IASWDNAMEQSAGKHADPSLVSSTSIPSSAMGNILDKNHTVPGNLLGHKIALTEVERGAQPVQSNWQI
PFEDNTGELPNWGFTQSLGLEFGSDYGTSLLGDVTNNAGPEIDPELFTFNGELKEQYTHGQSQPALKSN
SAYEVPGEASINYALTMRRGLLDGEESLKKVDSFSRWMTKELAGVDDLHMQSSPGISWSTDECGDVID
DTSLHLSLSQDQLFSINDFSPKWAYAESEIEVLIVGTFLKSQPVVAKCNWSCMFGEVEVPAEVLADGILC
CQAPPHKIGRVPFYVTCSNRFACSEVREFEYREGFDRNINFPDFFNNSSEMELHLRLVGLLSLNSMHTLN
QVFEGDMDKRNLIFKLISLKEEEEYSSKEETTAEMDISQQKLKEHMFHKQVKEKLYSWLLHKVTETGKG
PLVLDEEGQGVLHLIAALGYDWAINPIITAGVNINFRDVNGWTALHWAAFCGRERTVAVLVSMDAA
AGALTDPCPEFPLGRTPADLASSKGHKGISGFLAESLLTSHLESLTMDENKDGRKETSGMKVVQTVSER
TATPVLNGDIPDDICLKDSLNAVRNATQAADRIYQVFRMQSFQRKQLALYEDDEFGLSDQQALSLLAS
KACRSGQGEGLANAAAIQIQKKFRGWTKRKEFLIIRQRIVKIQAHVRGHQVRKQYKPIIWSVGILEKVIL
RWRRKGSGLRGFRPASQNKVPEQPSESPKEDDYDYLKEGRKQSEVKFKKALSRVKSMVQYPEARAQYR
RVLNVVEDFRQTKGGNLNLINSEETVDGVEDLIDIDMLLDDENFLPIAFD

>Glyma.05G117000 (GmCAMTA5)

MAERSCFGLGPRLDLQQLQLEAQHRWLRPAEICEILRNYQMFQITSEPPNGPPSGSLFLFDRKVLRYFRK
DGHNWRKKKDGKTVKEAHEKLKVGSVDVLHCYYAHGEENENFQRRSYWMLEPDMMHIVFVHYLD
VKVNKTNVGGKTYSDEVTSDSQKSSSLSSGFPRNYGSVPSGSTDSMSPTSTLTSLCEDADSEDIHQASSGL
HSYRESQNLGNDRPMDKIDARSNSSYPMHPFSGDHGQLPVSGAEYIPHVLGDKSRASDTTYIEGQRAQ
GIASWDNTMEQSAGEYADPSLVSSTTIPSSAVGNILEENHTVPGKLLGRKNALTEEERGSQPVQSNWQI
PFEDNTGELPNWGFTQSLGLEFGSDYGASLLGDVTNNAGPEIVPELFTFNGELKEQSVHQNFSKLYTHG
QSQPTLKSNSEYEVPGEASINYALTMRRGLLDGEESLKKVDSFSRWMTKEFAGVDDLHMQSSPGISWST
DECGDVIDDTSLNLSLSQDQLFSINDFSPKWAYAESEIEVLIVGTFLKSQPVVAKCNWSCMFGEVEVPAE
VLADGILCCQAPPHKIGRVPFYVTCSNRFACSEVREFEYREGFDRNIQFADCFNNSTEMVLHLRLVGLLS
LNSVRTSNQVFEGDMDKRSLIFKLISLKEEEEYSSKEETTAEMDISKHKLKELMFHKQVKEKLYSWLLHK
VTETGKGPLVLDEEGQGVLHLIAALGYDWAINPIITAGVNINFRDVNGWTALHWAAFCGRERTVAVL
VSMGAAAGAWTDPCPEFPSGRSPADLASSKGHKGISGFLAESLLTGHLESLTMDENKDGRKETSGTKV
VQTASERTATPVLYGDIPDAICLKDSLNAVRNATQAADRIYQVFRMQSFQRKQFAQYEDDEFGLSDQQ
ALSLLASKTCKSGQGEGLANAAAIQIQKKFRGWTKRKEFLIIRQRIVKIQAHVRGHQVRKQYKPIIWSV
GILEKVILRWRRKGSGLRGFRPAALNKVPEQPSESPKEDDYDYLKEGRKQSEVKFKKALSRVKSMVQYP
EARAQYRRVLNVVEDFRQTKGGNLNLINSEETVDGVEDLIDIDMLLDDENFLPIAFD

>Glyma.08G178900 (GmCAMTA6)

MAEGASYGLRRSLDIQQLQFEAQHRWLRPAEICEILRNYRMFHITSEPHNRPPSGSLFLFDRKVLRYFRK
DGHNWRKKKDGKTVKEAHEKLKIGSVDVLHCYYAHGEENENFQRRSYWMLEPDMMHIVFVHYLEV
KGNKNIVVNTEGDEIPSDSQKVTSSSSSLPTHHSSVPSLSTDSVSPTTSLMSLREDADSEDIHQASSGLRPL
YESQHSGNGPLTEKIGAGSNSSYLIHPFSGDYEQSSISGTDYIPVVHGDKFRGNDTAYIDGQKTHDVAT
WSTVLQSTAKLHNDPSLASSPSIPSSSMGDVLEQEHTIFSDLLMSKSGLTEVAESSQSLQSNWQIPFEDNS
GGMPMLTQTQTFGLQFRSDYGTGLLGNETRNACSESAAILYSFNGEPKEQPMQQNYLQELEDGQSQH
ALKSNSANKVPDEETINYGLTVKRTLLDKDESLKKVDSFSRWITKELGEVADLNMQSSPGISWSTDECQ
HVIDDTSLSPSLSQDQLFSINDFSPKWAYAESEIEVLIIGSFLKSQPEVTTCNWSCMFGEVEIPAKVLADGI
LCCQAPRHKVGRVPFYVTCSNRLACSEVREFDFREGFARNVDFADFYNSSEILLHLRLEDFLSLKPVDPS
NHSFEGDMEKRNLIFQLISLREVEEYSIKDEVTTELDISQHMVKEHLFHKQFKEKLYSWLLHKVTESGKG
PNVLDEDGQGVLHLAAFLGYDWAINPIISAGVNINFRDVNGWTALHWAASCGRERTVAVLVSMGAD
CGALTDPSPSSPAGRTAADLASSYGHKGISGFLAESSLTHHLETLTMDDQKGGRQEISGMKAVQTVSER
SATPVHFGDMPDLCLKDSLTAVRNATQAADRIHQVYRMQSFQRKQLTQYESDELGLSDQQALSLLASR
ACKSGQGDGLANAAAVQIQKKFRGWKKRQEFLMIRQRVAHVRGHQVRKQYKPIIWSVGILEKIILRW
RRKGSGLRGFRPNVINEVPDQQNNSLKEDDYDYLKEGRKQKEEKIQKALSRVKSMAQYPEARAQYRRL
LNVVEDFRQTKASNEGLINSEETVDGMEDLIDIDMLLDDDNFIPIAFD

>Glyma.17G038800 (GmCAMTA7)

MAETTKYIPNSQLELEEILNEAEHRWLRPAEICEILRNHKKFKLTPDPPVMPPAGSLFLFDRKALRYFRK
DGHRWRKKKDGKTVREAHEKLKAGSVDVLHCYYAHGEDNEYFQRRSYWMLDEQLEHIVLVHYREIK
EGCKSGISHLPVVPVTLVGSSQNTSVLSSTKINSPISLVQTSFTSSANKVYQNGRASEHEDVNSKNGPQAS
SHAQPISNYVLHSAPWLTHEAAGFSELLRNPLISSWPSSFPSYSPGTGLSPWTSIQNSSRNTINMHDGKH
HVEASEADLTVRKLSNAGLDSVHRMQDGVIFRDRLITDMCVQPVIDLPTVNQVKNEHGLDSFHAQVH
DHNDHPVVATTKILVEQKLQDGGLYNDESEQVEYGEMKKLDSFGRWMDKEIGGDCDNSLMASDSGN
YWSTLDAHSEDKEVSSLRHMQLDVDSLGPSLSQEQLFSIHDFSPDWAYTGVRTKVLIVGTFLGSKKPSSE
TKWGCMFGEIEVSAEVLADNVIRCQTPLHSPGRVPFYITCSNRLACSEVREFEFDENPTKFLGPEGIKISP
EEEVRLQMRLLKLVDLGPDNKWLKCSVSECEKCKLKGTMYSVRDDSGVFEETFQIDGIGHINHRDILFQ
RLVRDKLYEWLIYKVHEGGKGPHVLDDEGQGVIHLAAALGYVWAMAPLVAAGISPNFRDSRGRTGL
HWASYFGREETVIVLVQLGATPGAVEDPTSAFPRGQTAADLGSSRGHKGIAGYLAEADLTNQLSVLTV
KENETGNIATTIAANSALQSVEDDSSSMTMDEQHYLKESLAVFQKSAHAAASILAAFRARSFCQRQLA
QSSSDISEVLDVVADSLSKVQNKGHFEDYLHFAALKIQKRYRGWKGRKDFLKIRDRIVKIQAHIRGHQV
RKQYKKVVWSVSIVEKAILRWRRKGAGLRGFRVGQPVGVVVKDAEKSDEYEFLSIGRRQKSDDVKKAL
DRVKSMVRNPEARDQYMRLIMKYEKFKIDDGGSSQSQHVG

>Glyma.15G143400 (GmCAMTA8)

MMSHNLTGQLVSAEIHGFHTLEDLDVSNTMEEAKSRWLRPNEIHAILCNHKYFKINVKPVNLPKSGTI
VLFDRKMLRNFRKDGHNWKKKTDGKTVKEAHEHLKVGNEERIHVYYAHGQDNPTFVRRCYWLLDK
NLEHIVLVHYRDTQELQLQGSPATPVNSNSSSASDPAASWIPSEDLDSGVNSAYAVELNDNLTAKSHE
QRLHEINTLEWDDLVVPNVNTSTTSNGGNVPYSFQENQSLLSGRFGNVSSNPSAEIPSFGNLTQPVSGS
NSAPYSFPDSAILLKNSPISSGGVDTLGTLVNEGLQSQDSFGTWMNIISDTPCSIDESALKASISSVHVPYSS
LVADNLQSSLPEQVFNLTEVSPTWASSTEKTKVLVTGYFHNNYENLAKSNLLCVCGDVSVPVEIVQVG
VYRCCVPPHSPGLVNLYLSFDGHKPISQVVNFEYRTPILHEPTASMEEKYNWNEFRLQMRLAHLLFASD
TSLNIFSSKVSPNALKEARRFSFKTSYISKSWQYLMKSIDDNTIPFSKVKDSLFETALKNKLKEWLLERIILG
RKSTEYDAQGQGVIHLCAMLGYSWAISLFSWSGLSLDFRDKFGWTALHWAASYGMEKMVATLLSCG
ARPNLVTDPTPQYPGGCTAADLAYMKGCDGLAAFLSEKSLVEQFNEMSLAGNISGSLETSSTDPVNAE
NLTEDQLYVKETLAAYRISAEAAARIQAAFREHSFKLRYKAVEIISPEEEARQIVAAMRIQHAFRNYESK
KKMTAAARIQHRFRTWKYRREFLNMRHQAIKIQAAFRGFQARKQYRKIIWSVGVLEKVILRWRLKRKG

FRGLQVNPAREETQESDSIAEEDFFRTGRKQAEERIERSVIRVQAMFRSKKAQEEYRRMKLTHNQAKLE
LELEEFLDSEVDMLPKT

>Glyma.09G038300 (GmCAMTA9)

MMMSHNLTGQLVGAEIHGFHTLDDLDVSNTMEEAKSRWLRPNEIHAILCNHKYFKINAKPVNLPKSG
TIVLFDRKMLRNFRKDGHNWKKKTDGKTVKEAHEHLKVGNEERIHVYYAHGQDKPTFVRRCYWLLD
KSLEHIVLVHYRDTQELQLQGSPATPVNSNSSSVSDSAASWIPSDDLDSGVNSAYAVELNDSLTAKSHE
QRLHEINTLEWDDLVVSNANTSTTSNGGNVPYSFQQNQSLLNGSFGNVSSDPSAEIPSFGNLTQLVSGS
DSAPYSFPESADLLKSSPLSSGGVDTLGTLVNEGLQSQDSFGTWMNIMSDTPCSIDESALEATTSSVHVPY
SSLVADNKQSSLPEQVFNLTEVSPVWASSTEKTKVLVTGYFHNNYQHLAKSNLLCVCGDVSVPVEIVQ
VGVYRCWVSPHSPGLVTLYLSFDGHKPISQVVNFEYRTPILHEPTALIEEKYNWDEFRLQMRLAHLLFAS
DKSLNIFSSKVSTNALKEARRFSFKTSYISKSWQHLMKSIDDKTIPFSQVKDALFETSLKNKLKEWLLERII
LGSKSTEYDAQGQAAIHLCAMLGYNWAISLFTWSGLSLDFRDKFGWTALHWAAYYGIEKMVATLLSC
GARPNLVTDPTPQYPGGCTAADLAYVKGCDGLAAYLSEKSLVEQFNDMSLAGNISGSLETSSTDPVNA
ANLTEDQLYLKETLEAYRTAAEAAARIQAAFREHSFKLRYQAVEIMSPEEEARQIVAAMRIQHAFRNYE
SKKKMAAAARIQLRFRTWKYRREFLNMRRQAIKIQAAFRGFQARKQYRKIVWSVGVLEKVILRWLLKR
KGFRGLQVNPAEEETQESDTIAEEDFFRTSRKQAEERVERSVIRVQAMFRSKKAQEEYRRMKLTHNQA
MLDELEEFLNSEDDMLPKT

>Glyma.05G148300 (GmCAMTA10)

MTPGYEYDINDLHQEAQARWLKPAEVMYILQNHEKFQFTQEPPQQPTSGSLFLFNKRVLRFFRKDGH
NWRKKRDGRTVGEAHERLKVGNVEALNCYYAHGEQNPTFQRRSYWMLDPAYDHIVLVHYRNTSEG
KLSSGAGAQLSPSSSSVYTQSPSPYSTQNPGSTSILGDSYEPNQSFSSPGSTEVTSDMFVLNNKMGHMDG
TDTESGTSPELEVTQALRRLEVQLSLNEDNFEDIVSFGSKHETTHDSNPQHDQRVISNQEQSAAFSGPDD
QGLFYDGYNGRQGDGGEFYHELIDHGYPDGNEKALWTEVLESCKSSSAVKLPQKNVYMPVENLENSV
SSARRVPVSNQENSHWLNFNSNNSENSVFSQPQGVDEVKFPVYSSMVETQVINSDYYETLFDQSQIGAP
PDANSSLTVAQKQKFTIKTISPEWGYATETTKVIVVGSLLCHPSDSAWACMFGDVEVPVEIIQDGVISCE
APSHLPGKVTLCITSGNRESCSEVREFEYRDKTNSCTQCTQSETEATRSPEELLLLVRLEQMLLSASTIKN
DNIESGIPLIKQKADDDSWSHIIEALLVGSGTSTGTVDWLLEELLKDKLQQWLSCRSQEKDEETGCSLSK
KEQGIIHMVAGLGFEWALNPILTCGVNINFRDINGWTALHWAARFGREKMVASLIASGASAGAVTDP
NAQDPTGKTAASIAAGNGHKGLAGYLSEIAVTSHLSSLTLEESELSKSSAELQADMTVNSVSKENLTAS
EDQASLKDTLAAIRNVTQAAARIQSAFRSHSFRKRRAREVAASAGGIGTISEISAMSKLAFRNSREYNSA
ASAALSIQKKYRGWKGRKDFLALRKKVVKIQAHVRGYQVRKHYKVIWAVGILDKVVLRWRRKGAGL
RGFRQEMDINENENEDEDILKVFRKQKVDVEIEEAVSRVLSMVDSPDAREQYHRMLEKYRQAKAELA
GTSDEASLSTSVGDDLFIDDFYPFP

>Glyma.18G005100 (GmCAMTA111)

MPPGLEYNIDDLFQEAKRRWLKPVEVLYILRNHDQCEFTHQPPHQPAGGSLLLFNRRIMRFFRKDGHN
WRKKKDGKTVGEAHERLKVGNVEILNCYYAHGEENRTFQRRSYWMLEPEYDHIVLVHYRETSEGKSK
SEHVTQLSSGSSPVFSQSHSSYTTHNPGTASMFGDSCEPNQKFSSSGSLEDTSEAQALRQLEEQLSLNEDI
FNEIALDLIPGQDQRVVYKQDNSVALSGPNDPGQPCDGYNGREDDSGTYYHDFLDDCPGGNEKTIYW
TEVLESCKPLSVTKLPDQHAYDAIENGKSLFSSGRGMIANREKNQWLNSNSNNVENSVFLFPQDIGVKF
PPYSMVETPGTNYDYYETCFDQFQNQEPLGVDSSFTVVQKQKFTIRAVSPEYCYATETTKVIIIGSFLCHD
SDSTWACMFGDVEVPAEIIQDGVICCEAPSYLLGKVNLCVTSGNRVPCSEVRGFEFRNKTTSCTRCNSLE
TEGSKSLEDLLLLVRFAEMLLSASTTKDDRIESGSYLSTEQKDDDDSWSHIIIDTLLDGTRTSSDTVNWLL
EELLKDKLQLWLSNRRDEGTGCSFSRKEQGIIHMISGLGFEWALSPILSCGVNINFRDINGWTALHWAA
RFGREKMVASLIASGASAGAVTDPSSQDPTGKTAASIAASHGHKGLAGYLSEVDLTSHLSSLTLEESELS

KGSSELEAELTVSSVSKENLVASEDQVSLQAFLDAVRNAAQAAARIQAAFRAHSFRKRKEREAAADAG
LDGYCIDAGSIDNNISVLSAVSKLSSQSCRDYNLAALSIQKKYRGWKGRKEFLALRQKVVKIQACVRGY
QVRKQYKLILWAVGILDKVVLRWRRKRIGIRSVRQEMESNEEESDDEDFLSVFRKEKVNAAIEKALKQV
LSMVHSSGARQQYRRLLLLYRQAKAKTERGSTSDEAPLSTSEEEVSNMEDDDLCQFWETFWPS

>Glyma.17G031900 (GmCAMTA12)

MANNLAAQQLVGSEIHGFHTLQDLDVGSIMEEARTRWLRPNEIHAMLCNYKYFTINVKPVNLPKSGTI
VLFDRKMLRNFRKDGHNWKKKKDGKTVKEAHEHLKVGNEERIHVYYAHGQDNPNFVRRCYWLLD
KSMEHIVLVHYREIQEMQGSPVTPVNSHSSSVSDPPAPWILSEEIDSGTTTAYAGDTSANINVKSHELRL
HEINTLDWDDLVDANDHNTTTVPNGGTVPYFDLQDQILLNDSFSNVANNLSADIPSFGSLTQPIAGSN
SVPYNFSSVNLQTMDDQANPHEQRNNTVSLSGVDSLDTLVNDRLQSQNSFGMWVNPIMSDSPCSVDD
PALESPVSSVHEPYSSLIVDSQQSSLPGQVFTITDVSPTCVSSTEKSKVLVTGFFHKDYMHLSKSNLLCVCG
DVSVPAEIVQVGVYRCWVSPHSPGFVNLYMSIDGHKPISQVVNFEYRTPALHDPAVSMEESDNWDEFQ
LQMRLAYLLFKQLNLDVISTKVSPNRLKEARQFALKTSFISNSWQYLIKSTEDNQIPFSQAKDALFGIALK
SRLKEWLLERIVLGCKTTEYDAHGQSVIHLCAILGYTWAVSLFSWSGLSLDFRDRSGWTALHWAAYCG
REKMVATLLSAGAKPNLVTDPTPQNPGGCTAADLAYMRGHDGLAAYLSEKSLVQHFNDMSLAGNIS
GSLETSTTDPVISANLTEDQQNLKDTLAAYRTAAEAASRIHAAFREHSLKLRTKAVASSHPEAQARKIV
AAMKIQHAFRNHKTKKVMAAAARIQCTYRTWKIRKEFLNMRCQAVKIQAAFRCFQVRKHYCKILWS
VGVVEKAVLRWRLKRRGFRGLQVKTVEAGTGDQDQQSDVEEEFFRAGRKQAEERVERSVVRVQAMF
RSKKAQEEYRRMKLALDQAKLEREFERLLSTEVDMQLKT

>Glyma.07G242000.1(GmCAMTA13)

MANNLAVQLVGSEMHGFHTLQDLDVGSIMEEARTRWLRPNEIHAMLCNYKYFTINVKPVNLPKSGTI
VLFDRKMLRNFRKDGHNWKKKKDGKTVKEAHEHLKVGNEERIHVYYAHGQDNPNFVRRCYWLLD
KSMEHIVLVHYRETQEMQGSPVTPVNSHSSSVSDPPAPWILSEEIDSGTTTAYTGDMSNNINVKSHELRL
HEINTLEWDDLVDTNDHNASTVPNGGTVPYFDQQDQILLNDSFGNVANNLSAEIPSFGNLTQPIAGSN
RVPYNFSESVTLQTMDNQANPHEQKNNTVSLSGVDSLDTLVNDRLQSQDSFGMWVNHIMSDSPCSVD
DPALESPVSSIHEPYSSLVVDSQESSLPEQVFTITDVSPTCVSSTEKSKVLVTGFFLKDYMHLSKSNLLCVC
GDVSVPAEIVQVGVYRCWVSPHSPGFVNLYLSIDGHKPISQVVNFEYRTPALHDPAVSMEESDNWDEF
RQQMRLAYLLFAKQLNLDVISSKVSPNRLKEARQFALKTSFISNSWQYLIKSTEDNQIPFSQAKDALFGIT
LKNRLKEWLLERIVLGCKTTEYDAHGQSVIHLCAILGYNWAVSLFSWSGLSLDFRDRFGWTALHWAA
YCGREKMVATLLSAGAKPNLVTDPTPQNPGGCTAADLAYMRGHDGLAAYLSEKSLVQHFNDMSLAG
NISGSLETSTTDPVNPANLTEDQQNLKDTLTAYRTAAEAASRIHAAFREHSLKLRTKAVASSNPEAQAR
KIVAAMKIQHAFRNHETKKMMAAAARIQCTYRTWKIRKEFLNMRRQAVKIQAAFRCFQVRKHYRKIL
WSVGVVEKAVLRWRLKRRGFRGLQVKTVDAGTGDQDQQSDVEEEFFRTGRKQAEERVERSVVRVQA
MFRSKKAQEEYRRMKLALNQAKLEREYEQLLSTEVDMQL

>Glyma.11G251900 (GmCAMTA14)

MAGLEYSIDDLFQEAKRRWLKPVEALYILRNHDQCKFTHQPPHQPAGGSLFLFNRRIMRSFRKDGHN
WRKKKDGKTVGEAHERLKVGNVEILNCYYAHGEENRTFQRRSYWMLEPEYDHIVLVHYRETSEGKSN
SEHVTQLPSEVTFEAQALRQLEEQLSLNDDGFNEIALDLVSGQDQRVVYKQDKSAALSGPNDLGQPCD
GYNGRQDDSGTYYHDFLDDCPGGNEKTIYWTKVLESCKPLSVTKLPDQHAYEAIGNENTLFSSGRGVI
ANLENNQWLNSNSNNIENYGGVKFPPYSLAETPGANSDYYETFFDQFQNQGPLGVDSSLTVVQKQKF
TIRAVSPEYCYSTETTKVIIIGSFLCHDSDSTWACMFGDVEVPAEIIQDGIICCEAPSNHLGKVNLCITSGN
RVPCSEMREFEFRNKTTSCTRCNSLETEGSKSPEDLLLLVRFAEMLLSSSTTKDDRIESGSHLSTEQKDDD
DSWSHIIDTLLDSTRTPSDAVKWLLEELLKDKLQLWLSNRRDEGTGCSLSKKEQGIIHMVSGLGFEWAL

NPILSCGVNINFRDINGWTALHWAARFGREKMVASLIASGASAGAVTDPSSQDPTGKTAASIAASHDH
KGLAGYLSEVDLTSHLSSLTLEESELSRESSELEAELTVSSVSEENLVASEDQVSLKASLDAVRNAAQAAA
RIQAAFRAHSFRKRKERDAAATVLDGYCIDAGSIDNNISVLSAMSKLSSQSWRDYKAALSIQKKYRNW
KGRIEFLALRQKIVKIQACVRGYQVRKQYKLILWAVGILDKVVLRWRRKRIGIQSVRQEMESNEEESDD
ADFLNVFRKEKVNAAIEKALKRVLSMVHSTGARQQYRRLLSLYRQAKIEHGSTSDEAPLSTSEENASNM
EDDDLCQFLDTFFWPS

>Glyma.08G105200 (GmCAMTA15)

MTPGYEYDINDLHQEAQARWLKPAEVMYILQNHEKFQFTQEVPQQPTSGSLFLFNKRILRYFRRDGHN
WHKKSGGRTVGEAHERLKVLNVEALNCYYARGEQNPAFQRRSYWMLDPAYEHIVLVHYRNTSEGKL
SSGAGAQLSPSSSVYTQSPSPYSTQNPGSTSILGDSYEPNQSFSSPGSTKVTSEIFVLNNKMGHMDWADTE
SGTSSELEVTQALRRLEVQLSLNEDNFEDIVSFGSKHETVHDSNPKHDQRVISNQEQSAAFSRPDDQGLF
YDGCNGRQDHGYPDANEKALWTEQLESHKSSSAVKLPQKNVYMPAENENSVSSARRVPVSNQENSH
WLNFNCNNSENSVFSQPQGVDEVKFPAYSSMLETQVINSDYYETLFDQSQIGAPPDANSSLTVAQKQK
FTIKTISPEWGYATETTKVIVVGSFLCHPSDSAWACMFGDVEVPIEIIQDGVISCEAPSHLPGKVTLCITSG
NWESCSEVREFEYHDKTNSCTRCTQSETEATRSPEELLLLVRLGQMLLSASTIKNDNIESGIPLIKPKADD
DSWSHIIDALLVGSGTSSGTVDWLLEELLKDKFQQWLSFRSREKDEETGCSLSKKEQGIIHMVAGLGFE
WALNPILTCGVNINFRDINGWTALHWAARFGREKMVASLIASGASAGAVTDPNAQDPTGKTAASIAA
SSGHKGLAGYLSEIAVTSHLSSLTLEESESSKSSAYLQADRTVNSVSKENLTANEDQASLKDTLAAIRNVT
QAAARIQSAFRSHSFRKRRAREATASTGGIGTISEISAMSKLAFRNSHEYNSAALSIQKKYRGWKGRRDF
LALRQKVVKIQAHVRGYQVRKHYKVIWAVGILDKVVLRWRRKGAGLRGFRQEMDINENEDEDILKVF
RKQKLDVEIEEAVSRVLSMVDSPDAREQYHRMLEKYRQAKAELAGTSDEASLSTSVGDDLFMDDFYPF
P

Protein sequences (of CAMTA family of Arabidopsis, soybean, tomato and maize) for phylogenetic tree

>AtCAMTA1

MARKKKSVSFSHLDIARNEGNKIISFSVHAYSDRLGFVDSLFDYESLRSLLVDFWVYPSMVDRRSFGSITP
PLQLDMEQLLSEAQHRWLRPTEICEILQNYHKFHIASESPTRPASGSLFLFDRKVLRYFRKDGHNWRKK
KDGKTIREAHEKLKVGSIDVLHCYYAHGEANENFQRRCYWMLEQYYYRKASSHWVLVATLSLFSFGYL
RPSWVRHLMHIVFVHYLEVKGNRTSIGMKENNSNSVNGTASVNIDSTASPTSTLSSLCEDADTVLVQGI
VNKQVPSYDHLLNLKLEIAMVGHLLLACVMFHRFMGTESEKMQPSNTDSMLVEENSEKGGRLKAEHI
RNPLQTQFNWQDDTDLALFEQSAQDNFETFSSLLGSENLQPFGISYQAPPSNMDSEYMPVMKILRRSED
SLKKVDSFSKWAIKELGEMEDLQMQSSRGDIAWTTVECETAAAGISLSPSLSEDQRFTIVDFWPKSAKTD
AEVEVMVIGTFLLSPQEVTKYNWSCMFGEVEVPAEILVDGVLCCHAPPHTAGHVPFYVTCSNRFACSE
VREFDFLSGSTQKINATDVYGTYTNEASLQLRFEKMLAHRDFVHEHHIFEDVGDKRRQISKIMLLKEEK
EYLLPGTYQRDSTKQEPKGQLFRELFEEELYIWLIHKVTEEGKGPNILDEDGQGILHFVAALGYDWAIKP
VLAAGVNINFRDANGWSALHWAAFSGREETVAVLVSLGADAGALTDPSPELPLGKTAADLAYANGH
RGISGFLAESSLTSYLEKLTVDSKENSPANSCGEKAVQTVSERTAAPMTYGDVPEKLSLKDSLTAVRNAT
QAADRLHQVFRMQSFQRKQLCDIGDDEKIDISDQLAVSFAASKTKNPGQGDVSLSCAATHIQKKYRG
WKKRKEFLLIRQRIVKIQAHVRGHQVRKQYRTVIWSVGLLEKIILRWRRKGNGLRGFKRNAVAKTVEP
EPPVSAICPRIPQEDEYDYLKEGRKQTEERLQKALTRVKSMVQYPEARDQYRRLLTVVEGFRENEASSSA
SINNKEEEAVNCEEDDFIDIESLLNDDTLMMSISP

>AtCAMTA2

MADRGSFGFAPRLDIKQLLSEAQHRWLRPAEICEILRNHQKFHIASEPPNRPPSGSLFLFDRKVLRYFRK
DGHNWRKKKDGKTVKEAHEKLKVGSIDVLHCYYAHGEDNENFQRRCYWMLEQDLMHIVFVHYLEV
KGNRMSTSGTKENHSNSLSGTGSVNVDSTATRSSILSPLCEDADSGDSRQASSSLQQNPEPQTVVPQIM
HHQNASTINSYNTTSVLGNRDGWTSAHGNRVKGSNSQRSGDVPAWDASFENSLARYQNLPYNAPLT
QTQPSTFGLIPMEGKTEKGSLLTSEHLRNPLQSQVNWQTPVQESVPLQKWPMDSHSGMTDATDLALFG
QGAHENFGTFSSLLGSQDQQSSSFQAPFTNNEAAYIPKLGPEDLIYEASANQTLPLRKALLKKEDSLKKV
DSFSRWVSKELGEMEDLQMQSSSGGIAWTSVECENAAAGSSLSPSLSEDQRFTMIDFWPKWTQTDSEVE
VMVIGTFLLSPQEVTSYSWSCMFGEVEVPADILVDGVLCCHAPPHEVGRVPFYITCSDRFSCSEVREFDFL
PGSTRKLNATDIYGANTIETSLHLRFENLLALRCSVQEHHIFENVGEKRRKISKIMLLKDEKEPPLPGTIE
KDLTELEAKERLIREEFEDKLYLWLIHKVTEEGKGPNILDEDGQGVLHLAAALGYDWAIKPILAAGVSI
NFRDANGWSALHWAAFSGREDTVAVLVSLGADAGALADPSPEHPLGKTAADLAYGNGHRGISGFLA
ESSLTSYLEKLTVDAKENSSADSSGAKAVLTVAERTATPMSYGDVPETLSMKDSLTAVLNATQAADRL
HQVFRMQSFQRKQLSELGGDNKFDISDELAVSFAAAKTKKSGHSSGAVHAAAVQIQKKYRGWKKRKE
FLLIRQRIVKIQAHVRGHQVRKQYRAIIWSVGLLEKIILRWRRKGSGLRGFKRDTISKPTEPVCPAPQEDD
YDFLKEGRKQTEERLQKALTRVKSMAQYPEARAQYRRLLTVVEGFRENEASSSSALKNNTEEAANYNE
EDDLIDIDSLLDDDTFMSLAFE

>AtCAMTA3

MAEARRFSPVHELDVGQILSEARHRWLRPPEICEILQNYQRFQISTEPPTTPSSGSVFMFDRKVLRYFRKD
GHNWRKKKDGKTVKEAHERLKAGSVDVLHCYYAHGQDNENFQRRSYWLLQEELSHIVFVHYLEVKG
SRVSTSFNRMQRTEDAARSPQETGDALTSEHDGYASCSFNQNDHSNHSQTTDSASVNGFHSPELEDAE
SAYNQHGSSTAYSHQELQQPATGGNLTGFDPYYQISLTPRDSYQKELRTIPVTDSSIMVDKSKTINSPGV
TNGLKNRKSIDSQTWEEILGNCGSGVEALPLQPNSEHEVLDQILESSFTMQDFASLQESMVKSQNQELN
SGLTSDRTVWFQGQDMELNAISNLASNEKAPYLSTMKQHLLHGALGEEGLKKMDSFNRWMSKELGD
VGVIADANESFTQSSSRTYWEEVESEDGSNGHNSRRDMDGYVMSPSLSKEQLFSINDFSPSWAYVGCEV
VVFVTGKFLKTREETEIGEWSCMFGQTEVPADVISNGILQCVAPMHEAGRVPFYVTCSNRLACSEVREF
EYKVAESQVFDREADDESTIDILEARFVKLLCSKSENTSPVSGNDSDLSQLSEKISLLLFENDDQLDQML
MNEISQENMKNNLLQEFLKESLHSWLLQKIAEGGKGPSVLDEGGQGVLHFAASLGYNWALEPTIIAGV
SVDFRDVNGWTALHWAAFFGRERIIGSLIALGAAPGTLTDPNPDFPSGSTPSDLAYANGHKGIAGYLSE
YALRAHVSLLSLNDKNAETVEMAPSPSSSSLTDSLTAVRNATQAAARIHQVFRAQSFQKKQLKEFGDK
KLGMSEERALSMLAPKTHKSGRAHSDDSVQAAAIRIQNKFRGYKGRKDYLITRQRIIKIQAHVRGYQFR
KNYRKIIWSVGVLEKVILRWRRKGAGLRGFKSEALVEKMQDGTEKEEDDDFFKQGRKQTEDRLQKAL
ARVKSMVQYPEARDQYRRLLNVVNDIQESKVEKALENSEATCFDDDDDLIDIEALLEDDDTLMLPMSS
SLWTS

>AtCAMTA4

MSSVAEDNSFTCDIATIFVAICRNPPANPSDSLFQYEISTLYQEAHSRWLKPPEVLFILQNHESLTLTNTA
PQRPTSGSLLLFNKRVLKFFRKDGHQWRRKRDGRAIAEAHERLKVGNAEALNCYYAHGEQDPTFRRRI
YWMLDPEYEHIVLVHYRDVSEREEGQQTGGQVYQFAPILSTQNVSYNQYIGDSSDIYQQSSTSPGVAEV
NSNLEGSASSSEFGQALKMLKEQLSIGDEHVNSVDPHYIQPESLDSLQFLEYSDIDHLAQPTTVYQRPEN
NKLERCYGGNFGAQYSAKNDSNKLERCYGGYVGGAEYHSSNLMLVKNGSGPSGGTGGSGDQGSESW
KDVLEACEASIPLNSEGSTPSSAKGLLAGLQEDSNWSYSNQVDQSTFLLPQDLGSFQLPASYSALVAPEN
NGEYCGMMEDGMKIGLPFEQEMRVTGAHNQKFTIQDISPDWGYANETTKVIIIGSFLCDPTESTWSCM
FGNAQVPFEIIKEGVIRCEAPQCGPGKVNLCITSGDGLLCSEIREFEYREKPDTCCPKCSEPQTSDMSTSP

NELILLVRFVQTLLSDRSSERKSNLESGNDKLLTKLKADDDQWRHVIGTIIDGSASSTSTVDWLLQELLK
DKLDTWLSSRSCDEDYITCSLSKQEQGIIHMVAGLGFEWAFYPILAHGVNVDFRDIKGWSALHWAAQF
GSEKMVAALIASGASAGAVTDPSRQDPNGKTAASIAASNGHKGLAGYLSEVALTNHLSSLTLEETENS
KDTAQVQTEKTLNSISEQSPSGNEDQVSLKDTLAAVRNAAQAAARIQAAFRAHSFRKRKQREAALVA
CLQEYGMYCEDIEGISAMSKLTFGKGRNYNSAALSIQKNFRGYKDRKCFLELRQKVVKIQAHVRGYQIR
KNYKVICWAVRILDKVVLRWRRKGVGLRGFRQDVESTEDSEDEDILKVFRKQKVDVAVNEAFSRVLSM
SNSPEARQQYHRVLKRYCQTKAELGKTETLVGEDDDGLFDIADMEYDTLFSLP

>AtCAMTA5

MAGVDSGKLIGSEIHGFHTLQDLDIQTMLDEAYSRWLRPNEIHALLCNHKFFTINVKPVNLPKSGTIVL
FDRKMLRNFRKDGHNWKKKKDGKTIKEAHEHLKVGNEERIHVYYAHGEDTPTFVRRCYWLLDKSQE
HIVLVHYRETHEVHAAPATPGNSYSSSITDHLSPKIVAEDTSSGVHNTCNTGFEVRSNSLGSRNHEIRLH
EINTLDWDELLVPADISNQSHPTEEDMLYFTEQLQTAPRGSVKQGNHLAGYNGSVDIPSFPGLEDPVYQ
NNNSCGAGEFSSQHSHCGVDPNLQRRDFSATVTDQPGDALLNNGYGSQDSFGRWVNNFISDSPGSVD
DPSLEAVYTPGQDSSTPPTVFHSHSDIPEQVFNITDVSPAWAYSTEKTKILVTGFFHDSFQHLGRSNLICIC
GELRVPAEFLQMGVYRCFLPPQSPGVVNLYLSVDGNKPISQLFSFEHRSVQFIEKAIPQDDQLYKWEEFE
FQVRLAHLLFTSSNKISVLTSKISPENLLEAKKLASRTSHLLNSWAYLMKSIQANEVPFDQARDHLFELT
LKNRLKEWLLEKVIENRNTKEYDSKGLGVIHLCAVLGYTWSILLFSWANISLDFRDKQGWTALHWAA
YYGREKMVAALLSAGARPNLVTDPTKEFLGGCTAADLAQQKGYDGLAAFLAEKCLVAQFKDMQTAG
NISGNLETIKAEKSSNPGNANEEEQSLKDTLAAYRTAAEAAARIQGAFREHELKVRSSAVRFASKEEEA
KNIIAAMKIQHAFRNFEVRRKIAAAARIQYRFQTWKMRREFLNMRKKAIRIQAAFRGFQVRRQYQKIT
WSVGVLEKAILRWRLKRKGFRGLQVSQPDEKEGSEAVEDFYKTSQKQAEERLERSVVKVQAMFRSKKA
QQDYRRMKLAHEEAQLEYDGMQELDQMATEES

>AtCAMTA6

MDGDGLGRLIGSEIHGFHTLQDLDVQTMLEEAKSRWLRPNEIHAILYNPKYFTINVKPVNLPNSGRIILF
DRKMLRNFRKDGHNWKKKKDGRTVKEAHEHLKVGNEERIHVYYAHGEDNTTFVRRCYWLLDKARE
NIVLVHYRDTQEAATTSGDSISSPISVSEQTFPNRVAAEDIDTVVRNHDISLHDINTLDWDELLVPTDLN
NQSAPTVDNLSYFTEPLQNAANGTAEHGNATVADGSLDALLNDGPQSRESFGRWMNSFISESNGSLE
DPSFEPMVMPRQDPLAPQAVFHSHSNIPEQVFNITDVSPAWAYSSEKTKILVTGFLHDSYQHLERSNLY
CVCGDFCVPAEYLQAGVYRCIIPPHSPGMVNLYLSADGHKPISQCFRFEHRAVPVLDKTVPEDNQDSK
WEEFEFQVRLSHLLFTSSNKLNVLSSKISPHNLRDAKKLASKTNHLLNSWAYLVKSIQGNKVSFDQAKD
HLFELSLKNRLKEWLMEKVLEGRNTLDYDSKGLGVIHLCASLGYTWSVQLFSLSGLSLNFRDKQGWTA
LHWAAYYGREKMVAALLSAGARPNLVTDSTKDNLGGCMAADLAQQNGYDGLAAYLAEKCLVAQF
RDMKIAGNITGDLEACKAEMLNQGTLPEDEQSLKDALAAYRTAAEAAARIQGAFREKALKAARSSVI
QFANKEEEAKSIIAAMKIQNAFRKYDTRRKIEAAYRIQCRFQTWKIRREYLNMRRQAIRIQAAFRGLQA
RRQYKKILWSVGVLEKAVLRWRQKRKGFRGLQVAAEEDSPGEAQEDFYKTSQRQAEERLERSVVRVQ
AMFRSKKAQQDYRRMKLTHEEAQLEYGCLEDI

>GmCAMTA1

MAEARHYVPPSQLDIKQIILEAQHRWLRPAEICAILSNHKKFLIASEPAHMPPSGSLFLFDRKVLRYFRK
DGHNWRKKKDGKTVREAHERLKAGSVDVLHCYYAHGEENENFRRRTYWLLEEELSHIVLVHYRHVK
GTKANFTCAKENEETLPYAQQTDKIMPKTEMETSLSSTLHPHSYQVPSQTMDRSMNSSQASEYEEAESA
FNNHASSEFYSFLELERPVEKITPQPADSYSPRPLTRKSVPNMNCIIESGTDDQEKSPVIPGVNYISLTQDN

KIKDIHNFGLTYESPKPLGFSSWEGILKNNAGSQHVPFQPLFPGTQPDNMGINSKFSQGHEIMVPYLTTS
IAKQHENGSLIQAEGNWQAYDVDSLRMSSWPIDSAYSGSSCDITCSNREQEVNDVDLQKSLEQCLLHP
YKQNKVFMQNDPQEKLLNEKEKIKSDLEANRILDGIEDTYFTFKRTLLDGSPAEEGLKKLDSFNQWMS
KELADVEESNKPSTSGGYWDTVESENEVGNTTIPSQGHLDTYVLDPSVSHDQLFSIIDYSPSWAFEGSEIK
VIISGRFLRSQHEAEQGKWSCMFGEVEVPAEIIAKGVLCCHTPPHKAGRVPFYVTCSNRLACSEVREFDF
QVNYTPEVNTTGENRGSTFDTFSIRFGELLSLGHAFPQNSDSISVSEKSQLRSKINSLLREEEDDWDKLLK
LTQEEDFSPENLQEQLLQNLLKDKLHAWLLQKITEEGKGPNILDEGGQGVLHFASALGYDWALEPTIV
AGVNVNFRDVNGWTALHWAAFCGRERTVAFLISLGAAPGALTDPCPEHPSGRTPADLASANGHKGI
AGYLAESSLSAHLTTLDLNRDAGENSGAKVVQRVQNIAQVNDLDGLSYELSLKDSLAAVRNATHAAA
RIHQVFRMQSFQRKQLKEYDDDKLGLSDERALSLVKMNMKSHKSGPRDEPVHAAAVRIQNKFRSWK
GRREFLMIRQRIVKIQAHVRGHQVRKSCGKIIWSVGILEKVILRWRRKGSGLRGFKPEANSEGTMIQDVS
STDDDYDVLKEGRKQTEQRLQKALARVKSMVQYPEARDQYHRLLNVVTEIQENQVKHESSYNNSEEP
REFGDLNDLEALLDEDIFMPTAT

>GmCAMTA2

MAEARLYAPPSQLDIKQIILEAQHRWLRPAEICAILGNYKKFRIAPEPAHMPPSGSLFLFDRKVLRHFRK
DGHNWRKKKDGKTVREAHERLKAGSVDVLHCYYAHGEENENFQRRTYWLLEEELSHIVLVHYRQVK
GTKANFTSAKENEESLPYAQQTDKIMPQTEMDTSLSSTLHPHSYQVPSKTVDTSMNSAQTSEYEEAESA
FNNHASSEFYSFLELQRPVEKISPQPADFYSPRPLINDQEKLPIIPGVNYISLTQDNKNKDILNAGLTYESP
KPLGFSSWEGILENNAGSQHVHFQPLFPGTQPDNMGINSNFSQGEEIMVPYLTTSIAKQHENGSIIKAEG
NWQVYDVDSLRMSSWPIDSAYSGSTCEVSCSNCEQEVNDVDFQKSLEQCLLHSHKQNKVLMQNDLQ
EKLLNEKEKIKSNLEAYGIEDTYLSFKRTLLDGPPAEEGLKKLDSFNQWMSKELGDVEESNKPSTSGGY
WDTVETENEVGNTTIPSQGHLDTYVLDPSVSHDQLFSIIDYSPSWAFEGSEIKVIISGEFLRSQHEAEQCK
WSCMFGEVEVPAVIIAKGVLCCHTPPHKAGRVPFYVTCSNRLACSEVREFDFQVHYTPEDTTGENRGST
FDTFSIRFGELLSLGHAFPQNSDSISVSEKSQLRSKINSLLREDDDDWDKLLKLTQEKDFSPENLREQLLQ
NLLKDKLHAWLLQKITEEGKGPNVLDEGGQGVLHFAAALGYDWALEPTIVAGVNVNFRDVNGWTSL
HWAAFCGRERTVAFLISLGAAPGALTDPCPEHPSGRTPADLASANGHKGIAGYLAESSLSAHLTTLDL
NRDAGENSGAKVVQRLQNIAQVNDLDGLSYELSLKDSLAAVCNATQAAARIHQVFRMQSFQRKQLK
EYDDDKLGLSDERALSLIKMNVKSHKSGPRDEPVHAAAIRIQNKFRSWKGRREFLMIRQRIVKIQAHVR
GHQVRKSCGKIIWSVGILEKVILRWRRKGSGLRGFKPEANSEGTMIQDVSSTDDDYDVLKEGRKQTEQR
LQKALARVKSMVQYPEARDQYHRLLNVVTEIQENQVKHESSSNNSEEPREFGDLNDLEALLDEDIFMP
TAT

>GmCAMTA3

MAEGASYGLRRPLDIQQLQFEAQHRWLRPAEICEILRNYRMFHITSEPHNRPPSGSLFLFDRKVLRYFRK
DGHNWRKKKDGKTVKEAHEKLKVGSVDVLHCYYAHGEENENFQRRSYWMLEPDMMHIVFVHYLEV
KGNKNIVVNNEGDEVPTDSQKVTSPSSSLPTHHSCVSSLSTDSVSPTTSLMSLHEDADSEDIHHASSGLH
PLHESQHSGNSPLTEKIGAGSNSSYLMHPFSGDNEQSSISGTDYVPVVHGDKFRGNDTAYTDGQKPHG
MAPWGTVLQSTAKLHNDPSLASFPSILPSSMGDVLEQEHTIFGDLLMSKSGLTEEAESSQSLQSNWQIPF
EDNSGGMPMLTQTQSFGLQFRSDYGTGLLGNETRNASSEIAPILYSFHGEPKEQPMQQNYPQELEDGQS
QHALKSNSANKVPDEETINYGLTVKSTLLDRDESLKKVDSFSRWITKELGEVADLNMQSSPGISWSTDE
CQHVIDDTSLSPSLSQDQLFSINDFSPKWAYAESEIEVLIIGSFLKSQPEVTTCNWSCMFGEVEVPAEVLA
DGILCCQAPCHKVGRVPFYVTCSNRLACSEVREFDFREGFARNVDFADFYISSTEMLRHLRLEDFLSLKP
VDPSNHSFEGDMEKRNLIFKLISLREEEDYSIKDEVTRELDISQHMVKEHLFHRQFKEKLYSWLLHKVTE

NGKGPNVLDEDGQGVLHLAAFLGYDWAINPIISAGVNINFRDVNGWTALHWAASCGRERTVAVLVS
MGADCGALTDPSPAFPSGRTAADLASSYGHKGISGFLAESSLTHHLETLTMDDQKGGQQEISGMKVVQ
TVSERSATPVHYCDIPDAICLKDSLTAVRNATQAADRIHQVYRMQSFQRKQLTQYEGDDELGLSDQQA
LSLLASRACKSGQGDGLANAAAVQIQKKFRGWKKRKEFLMIRQRVVKIQAHVRGHQIRKQYKPIIWSV
GILEKVILRWRRKGSGLRGFRPNAINKVPNQQNDSLKEDDYDYLKEGRKQKEEKIQKALSRVKSMVQY
PEARAQYRRLLNVVEDFRQTKASNKGLINSEETVDGVEDLIDIDMLLDDDNFIPIAFD

>GmCAMTA4

MSERSSFGLGPRLDLQQLQLEAQHRWLRPAEICEILRNYRMFQITSEPPNRPPSGSLFLFDRKVLRYFRKD
GHNWRKKKDGKTVKEAHEKLKVGSVDVLHCYYAHGEENENFQRRSYWMLELDMMHIVFVHYLDV
KVNKTNIGGKTYSDEVTSDSQKSSSLSSGFPRNYGSMPSGSTDSMSPTSTLTSLCEDADSEDIHQASSGLH
SYRESQNLGNDRPMDKIHARSNSSYLMHPFSDNHGQLPVSGAEYIPHVQGNKSRASDTTYIEGQRAHG
IASWDNAMEQSAGKHADPSLVSSTSIPSSAMGNILDKNHTVPGNLLGHKIALTEVERGAQPVQSNWQI
PFEDNTGELPNWGFTQSLGLEFGSDYGTSLLGDVTNNAGPEIDPELFTFNGELKEQYTHGQSQPALKSN
SAYEVPGEASINYALTMRRGLLDGEESLKKVDSFSRWMTKELAGVDDLHMQSSPGISWSTDECGDVID
DTSLHLSLSQDQLFSINDFSPKWAYAESEIEVLIVGTFLKSQPVVAKCNWSCMFGEVEVPAEVLADGILC
CQAPPHKIGRVPFYVTCSNRFACSEVREFEYREGFDRNINFPDFFNNSSEMELHLRLVGLLSLNSMHTLN
QVFEGDMDKRNLIFKLISLKEEEEYSSKEETTAEMDISQQKLKEHMFHKQVKEKLYSWLLHKVTETGKG
PLVLDEEGQGVLHLIAALGYDWAINPIITAGVNINFRDVNGWTALHWAAFCGRERTVAVLVSMDAA
AGALTDPCPEFPLGRTPADLASSKGHKGISGFLAESLLTSHLESLTMDENKDGRKETSGMKVVQTVSER
TATPVLNGDIPDDICLKDSLNAVRNATQAADRIYQVFRMQSFQRKQLALYEDDEFGLSDQQALSLLAS
KACRSGQGEGLANAAAIQIQKKFRGWTKRKEFLIIRQRIVKIQAHVRGHQVRKQYKPIIWSVGILEKVIL
RWRRKGSGLRGFRPASQNKVPEQPSESPKEDDYDYLKEGRKQSEVKFKKALSRVKSMVQYPEARAQYR
RVLNVVEDFRQTKGGNLNLINSEETVDGVEDLIDIDMLLDDENFLPIAFD

>GmCAMTA5

MAERSCFGLGPRLDLQQLQLEAQHRWLRPAEICEILRNYQMFQITSEPPNGPPSGSLFLFDRKVLRYFRK
DGHNWRKKKDGKTVKEAHEKLKVGSVDVLHCYYAHGEENENFQRRSYWMLEPDMMHIVFVHYLD
VKVNKTNVGGKTYSDEVTSDSQKSSSLSSGFPRNYGSVPSGSTDSMSPTSTLTSLCEDADSEDIHQASSGL
HSYRESQNLGNDRPMDKIDARSNSSYPMHPFSGDHGQLPVSGAEYIPHVLGDKSRASDTTYIEGQRAQ
GIASWDNTMEQSAGEYADPSLVSSTTIPSSAVGNILEENHTVPGKLLGRKNALTEEERGSQPVQSNWQI
PFEDNTGELPNWGFTQSLGLEFGSDYGASLLGDVTNNAGPEIVPELFTFNGELKEQSVHQNFSKLYTHG
QSQPTLKSNSEYEVPGEASINYALTMRRGLLDGEESLKKVDSFSRWMTKEFAGVDDLHMQSSPGISWST
DECGDVIDDTSLNLSLSQDQLFSINDFSPKWAYAESEIEVLIVGTFLKSQPVVAKCNWSCMFGEVEVPAE
VLADGILCCQAPPHKIGRVPFYVTCSNRFACSEVREFEYREGFDRNIQFADCFNNSTEMVLHLRLVGLLS
LNSVRTSNQVFEGDMDKRSLIFKLISLKEEEEYSSKEETTAEMDISKHKLKELMFHKQVKEKLYSWLLHK
VTETGKGPLVLDEEGQGVLHLIAALGYDWAINPIITAGVNINFRDVNGWTALHWAAFCGRERTVAVL
VSMGAAAGAWTDPCPEFPSGRSPADLASSKGHKGISGFLAESLLTGHLESLTMDENKDGRKETSGTKV
VQTASERTATPVLYGDIPDAICLKDSLNAVRNATQAADRIYQVFRMQSFQRKQFAQYEDDEFGLSDQQ
ALSLLASKTCKSGQGEGLANAAAIQIQKKFRGWTKRKEFLIIRQRIVKIQAHVRGHQVRKQYKPIIWSV
GILEKVILRWRRKGSGLRGFRPAALNKVPEQPSESPKEDDYDYLKEGRKQSEVKFKKALSRVKSMVQYP
EARAQYRRVLNVVEDFRQTKGGNLNLINSEETVDGVEDLIDIDMLLDDENFLPIAFD

>GmCAMTA6

MAEGASYGLRRSLDIQQLQFEAQHRWLRPAEICEILRNYRMFHITSEPHNRPPSGSLFLFDRKVLRYFRK
DGHNWRKKKDGKTVKEAHEKLKIGSVDVLHCYYAHGEENENFQRRSYWMLEPDMMHIVFVHYLEV
KGNKNIVVNTEGDEIPSDSQKVTSSSSSLPTHHSSVPSLSTDSVSPTTSLMSLREDADSEDIHQASSGLRPL
YESQHSGNGPLTEKIGAGSNSSYLIHPFSGDYEQSSISGTDYIPVVHGDKFRGNDTAYIDGQKTHDVAT
WSTVLQSTAKLHNDPSLASSPSIPSSSMGDVLEQEHTIFSDLLMSKSGLTEVAESSQSLQSNWQIPFEDNS
GGMPMLTQTQTFGLQFRSDYGTGLLGNETRNACSESAAILYSFNGEPKEQPMQQNYLQELEDGQSQH
ALKSNSANKVPDEETINYGLTVKRTLLDKDESLKKVDSFSRWITKELGEVADLNMQSSPGISWSTDECQ
HVIDDTSLSPSLSQDQLFSINDFSPKWAYAESEIEVLIIGSFLKSQPEVTTCNWSCMFGEVEIPAKVLADGI
LCCQAPRHKVGRVPFYVTCSNRLACSEVREFDFREGFARNVDFADFYNSSEILLHLRLEDFLSLKPVDPS
NHSFEGDMEKRNLIFQLISLREVEEYSIKDEVTTELDISQHMVKEHLFHKQFKEKLYSWLLHKVTESGKG
PNVLDEDGQGVLHLAAFLGYDWAINPIISAGVNINFRDVNGWTALHWAASCGRERTVAVLVSMGAD
CGALTDPSPSSPAGRTAADLASSYGHKGISGFLAESSLTHHLETLTMDDQKGGRQEISGMKAVQTVSER
SATPVHFGDMPDLCLKDSLTAVRNATQAADRIHQVYRMQSFQRKQLTQYESDELGLSDQQALSLLASR
ACKSGQGDGLANAAAVQIQKKFRGWKKRQEFLMIRQRVAHVRGHQVRKQYKPIIWSVGILEKIILRW
RRKGSGLRGFRPNVINEVPDQQNNSLKEDDYDYLKEGRKQKEEKIQKALSRVKSMAQYPEARAQYRRL
LNVVEDFRQTKASNEGLINSEETVDGMEDLIDIDMLLDDDNFIPIAFD

>GmCAMTA7

MAETTKYIPNSQLELEEILNEAEHRWLRPAEICEILRNHKKFKLTPDPPVMPPAGSLFLFDRKALRYFRK
DGHRWRKKKDGKTVREAHEKLKAGSVDVLHCYYAHGEDNEYFQRRSYWMLDEQLEHIVLVHYREIK
EGCKSGISHLPVVPVTLVGSSQNTSVLSSTKINSPISLVQTSFTSSANKVYQNGRASEHEDVNSKNGPQAS
SHAQPISNYVLHSAPWLTHEAAGFSELLRNPLISSWPSSFPSYSPGTGLSPWTSIQNSSRNTINMHDGKH
HVEASEADLTVRKLSNAGLDSVHRMQDGVIFRDRLITDMCVQPVIDLPTVNQVKNEHGLDSFHAQVH
DHNDHPVVATTKILVEQKLQDGGLYNDESEQVEYGEMKKLDSFGRWMDKEIGGDCDNSLMASDSGN
YWSTLDAHSEDKEVSSLRHMQLDVDSLGPSLSQEQLFSIHDFSPDWAYTGVRTKVLIVGTFLGSKKPSSE
TKWGCMFGEIEVSAEVLADNVIRCQTPLHSPGRVPFYITCSNRLACSEVREFEFDENPTKFLGPEGIKISP
EEEVRLQMRLLKLVDLGPDNKWLKCSVSECEKCKLKGTMYSVRDDSGVFEETFQIDGIGHINHRDILFQ
RLVRDKLYEWLIYKVHEGGKGPHVLDDEGQGVIHLAAALGYVWAMAPLVAAGISPNFRDSRGRTGL
HWASYFGREETVIVLVQLGATPGAVEDPTSAFPRGQTAADLGSSRGHKGIAGYLAEADLTNQLSVLTV
KENETGNIATTIAANSALQSVEDDSSSMTMDEQHYLKESLAVFQKSAHAAASILAAFRARSFCQRQLA
QSSSDISEVLDVVADSLSKVQNKGHFEDYLHFAALKIQKRYRGWKGRKDFLKIRDRIVKIQAHIRGHQV
RKQYKKVVWSVSIVEKAILRWRRKGAGLRGFRVGQPVGVVVKDAEKSDEYEFLSIGRRQKSDDVKKAL
DRVKSMVRNPEARDQYMRLIMKYEKFKIDDGGSSQSQHVG

>GmCAMTA8

MMSHNLTGQLVSAEIHGFHTLEDLDVSNTMEEAKSRWLRPNEIHAILCNHKYFKINVKPVNLPKSGTI
VLFDRKMLRNFRKDGHNWKKKTDGKTVKEAHEHLKVGNEERIHVYYAHGQDNPTFVRRCYWLLDK
NLEHIVLVHYRDTQELQLQGSPATPVNSNSSSASDPAASWIPSEDLDSGVNSAYAVELNDNLTAKSHE
QRLHEINTLEWDDLVVPNVNTSTTSNGGNVPYSFQENQSLLSGRFGNVSSNPSAEIPSFGNLTQPVSGS
NSAPYSFPDSAILLKNSPISSGGVDTLGTLVNEGLQSQDSFGTWMNIISDTPCSIDESALKASISSVHVPYSS
LVADNLQSSLPEQVFNLTEVSPTWASSTEKTKVLVTGYFHNNYENLAKSNLLCVCGDVSVPVEIVQVG
VYRCCVPPHSPGLVNLYLSFDGHKPISQVVNFEYRTPILHEPTASMEEKYNWNEFRLQMRLAHLLFASD
TSLNIFSSKVSPNALKEARRFSFKTSYISKSWQYLMKSIDDNTIPFSKVKDSLFETALKNKLKEWLLERIILG
RKSTEYDAQGQGVIHLCAMLGYSWAISLFSWSGLSLDFRDKFGWTALHWAASYGMEKMVATLLSCG

ARPNLVTDPTPQYPGGCTAADLAYMKGCDGLAAFLSEKSLVEQFNEMSLAGNISGSLETSSTDPVNAE
NLTEDQLYVKETLAAYRISAEAAARIQAAFREHSFKLRYKAVEIISPEEEARQIVAAMRIQHAFRNYESK
KKMTAAARIQHRFRTWKYRREFLNMRHQAIKIQAAFRGFQARKQYRKIIWSVGVLEKVILRWRLKRKG
FRGLQVNPAREETQESDSIAEEDFFRTGRKQAEERIERSVIRVQAMFRSKKAQEEYRRMKLTHNQAKLE
LELEEFLDSEVDMLPKT

>GmCAMTA9

MMMSHNLTGQLVGAEIHGFHTLDDLDVSNTMEEAKSRWLRPNEIHAILCNHKYFKINAKPVNLPKSG
TIVLFDRKMLRNFRKDGHNWKKKTDGKTVKEAHEHLKVGNEERIHVYYAHGQDKPTFVRRCYWLLD
KSLEHIVLVHYRDTQELQLQGSPATPVNSNSSSVSDSAASWIPSDDLDSGVNSAYAVELNDSLTAKSHE
QRLHEINTLEWDDLVVSNANTSTTSNGGNVPYSFQQNQSLLNGSFGNVSSDPSAEIPSFGNLTQLVSGS
DSAPYSFPESADLLKSSPLSSGGVDTLGTLVNEGLQSQDSFGTWMNIMSDTPCSIDESALEATTSSVHVPY
SSLVADNKQSSLPEQVFNLTEVSPVWASSTEKTKVLVTGYFHNNYQHLAKSNLLCVCGDVSVPVEIVQ
VGVYRCWVSPHSPGLVTLYLSFDGHKPISQVVNFEYRTPILHEPTALIEEKYNWDEFRLQMRLAHLLFAS
DKSLNIFSSKVSTNALKEARRFSFKTSYISKSWQHLMKSIDDKTIPFSQVKDALFETSLKNKLKEWLLERII
LGSKSTEYDAQGQAAIHLCAMLGYNWAISLFTWSGLSLDFRDKFGWTALHWAAYYGIEKMVATLLSC
GARPNLVTDPTPQYPGGCTAADLAYVKGCDGLAAYLSEKSLVEQFNDMSLAGNISGSLETSSTDPVNA
ANLTEDQLYLKETLEAYRTAAEAAARIQAAFREHSFKLRYQAVEIMSPEEEARQIVAAMRIQHAFRNYE
SKKKMAAAARIQLRFRTWKYRREFLNMRRQAIKIQAAFRGFQARKQYRKIVWSVGVLEKVILRWLLKR
KGFRGLQVNPAEEETQESDTIAEEDFFRTSRKQAEERVERSVIRVQAMFRSKKAQEEYRRMKLTHNQA
MLDELEEFLNSEDDMLPKT

>GmCAMTA10

MTPGYEYDINDLHQEAQARWLKPAEVMYILQNHEKFQFTQEPPQQPTSGSLFLFNKRVLRFFRKDGH
NWRKKRDGRTVGEAHERLKVGNVEALNCYYAHGEQNPTFQRRSYWMLDPAYDHIVLVHYRNTSEG
KLSSGAGAQLSPSSSSVYTQSPSPYSTQNPGSTSILGDSYEPNQSFSSPGSTEVTSDMFVLNNKMGHMDG
TDTESGTSPELEVTQALRRLEVQLSLNEDNFEDIVSFGSKHETTHDSNPQHDQRVISNQEQSAAFSGPDD
QGLFYDGYNGRQGDGGEFYHELIDHGYPDGNEKALWTEVLESCKSSSAVKLPQKNVYMPVENLENSV
SSARRVPVSNQENSHWLNFNSNNSENSVFSQPQGVDEVKFPVYSSMVETQVINSDYYETLFDQSQIGAP
PDANSSLTVAQKQKFTIKTISPEWGYATETTKVIVVGSLLCHPSDSAWACMFGDVEVPVEIIQDGVISCE
APSHLPGKVTLCITSGNRESCSEVREFEYRDKTNSCTQCTQSETEATRSPEELLLLVRLEQMLLSASTIKN
DNIESGIPLIKQKADDDSWSHIIEALLVGSGTSTGTVDWLLEELLKDKLQQWLSCRSQEKDEETGCSLSK
KEQGIIHMVAGLGFEWALNPILTCGVNINFRDINGWTALHWAARFGREKMVASLIASGASAGAVTDP
NAQDPTGKTAASIAAGNGHKGLAGYLSEIAVTSHLSSLTLEESELSKSSAELQADMTVNSVSKENLTAS
EDQASLKDTLAAIRNVTQAAARIQSAFRSHSFRKRRAREVAASAGGIGTISEISAMSKLAFRNSREYNSA
ASAALSIQKKYRGWKGRKDFLALRKKVVKIQAHVRGYQVRKHYKVIWAVGILDKVVLRWRRKGAGL
RGFRQEMDINENENEDEDILKVFRKQKVDVEIEEAVSRVLSMVDSPDAREQYHRMLEKYRQAKAELA
GTSDEASLSTSVGDDLFIDDFYPFP

>GmCAMTA11

MAGLEYSIDDLFQEAKRRWLKPVEALYILRNHDQCKFTHQPPHQPAGGSLFLFNRRIMRSFRKDGHN
WRKKKDGKTVGEAHERLKVGNVEILNCYYAHGEENRTFQRRSYWMLEPEYDHIVLVHYRETSEGKSN
SEHVTQLPSEVTFEAQALRQLEEQLSLNDDGFNEIALDLVSGQDQRVVYKQDKSAALSGPNDLGQPCD
GYNGRQDDSGTYYHDFLDDCPGGNEKTIYWTKVLESCKPLSVTKLPDQHAYEAIGNENTLFSSGRGVI

ANLENNQWLNSNSNNIENYGGVKFPPYSLAETPGANSDYYETFFDQFQNQGPLGVDSSLTVVQKQKF
TIRAVSPEYCYSTETTKVIIIGSFLCHDSDSTWACMFGDVEVPAEIIQDGIICCEAPSNHLGKVNLCITSGN
RVPCSEMREFEFRNKTTSCTRCNSLETEGSKSPEDLLLLVRFAEMLLSSSTTKDDRIESGSHLSTEQKDDD
DSWSHIIDTLLDSTRTPSDAVKWLLEELLKDKLQLWLSNRRDEGTGCSLSKKEQGIIHMVSGLGFEWAL
NPILSCGVNINFRDINGWTALHWAARFGREKMVASLIASGASAGAVTDPSSQDPTGKTAASIAASHDH
KGLAGYLSEVDLTSHLSSLTLEESELSRESSELEAELTVSSVSEENLVASEDQVSLKASLDAVRNAAQAAA
RIQAAFRAHSFRKRKERDAAATVLDGYCIDAGSIDNNISVLSAMSKLSSQSWRDYKAALSIQKKYRNW
KGRIEFLALRQKIVKIQACVRGYQVRKQYKLILWAVGILDKVVLRWRRKRIGIQSVRQEMESNEEESDD
ADFLNVFRKEKVNAAIEKALKRVLSMVHSTGARQQYRRLLSLYRQAKIEHGSTSDEAPLSTSEENASNM
EDDDLCQFLDTFFWPS

>GmCAMTA12

MANNLAAQQLVGSEIHGFHTLQDLDVGSIMEEARTRWLRPNEIHAMLCNYKYFTINVKPVNLPKSGTI
VLFDRKMLRNFRKDGHNWKKKKDGKTVKEAHEHLKVGNEERIHVYYAHGQDNPNFVRRCYWLLD
KSMEHIVLVHYREIQEMQGSPVTPVNSHSSSVSDPPAPWILSEEIDSGTTTAYAGDTSANINVKSHELRL
HEINTLDWDDLVDANDHNTTTVPNGGTVPYFDLQDQILLNDSFSNVANNLSADIPSFGSLTQPIAGSN
SVPYNFSSVNLQTMDDQANPHEQRNNTVSLSGVDSLDTLVNDRLQSQNSFGMWVNPIMSDSPCSVDD
PALESPVSSVHEPYSSLIVDSQQSSLPGQVFTITDVSPTCVSSTEKSKVLVTGFFHKDYMHLSKSNLLCVCG
DVSVPAEIVQVGVYRCWVSPHSPGFVNLYMSIDGHKPISQVVNFEYRTPALHDPAVSMEESDNWDEFQ
LQMRLAYLLFKQLNLDVISTKVSPNRLKEARQFALKTSFISNSWQYLIKSTEDNQIPFSQAKDALFGIALK
SRLKEWLLERIVLGCKTTEYDAHGQSVIHLCAILGYTWAVSLFSWSGLSLDFRDRSGWTALHWAAYCG
REKMVATLLSAGAKPNLVTDPTPQNPGGCTAADLAYMRGHDGLAAYLSEKSLVQHFNDMSLAGNIS
GSLETSTTDPVISANLTEDQQNLKDTLAAYRTAAEAASRIHAAFREHSLKLRTKAVASSHPEAQARKIV
AAMKIQHAFRNHKTKKVMAAAARIQCTYRTWKIRKEFLNMRCQAVKIQAAFRCFQVRKHYCKILWS
VGVVEKAVLRWRLKRRGFRGLQVKTVEAGTGDQDQQSDVEEEFFRAGRKQAEERVERSVVRVQAMF
RSKKAQEEYRRMKLALDQAKLEREFERLLSTEVDMQLKT

>GmCAMTA13

MANNLAVQLVGSEMHGFHTLQDLDVGSIMEEARTRWLRPNEIHAMLCNYKYFTINVKPVNLPKSGTI
VLFDRKMLRNFRKDGHNWKKKKDGKTVKEAHEHLKVGNEERIHVYYAHGQDNPNFVRRCYWLLD
KSMEHIVLVHYRETQEMQGSPVTPVNSHSSSVSDPPAPWILSEEIDSGTTTAYTGDMSNNINVKSHELRL
HEINTLEWDDLVDTNDHNASTVPNGGTVPYFDQQDQILLNDSFGNVANNLSAEIPSFGNLTQPIAGSN
RVPYNFSESVTLQTMDNQANPHEQKNNTVSLSGVDSLDTLVNDRLQSQDSFGMWVNHIMSDSPCSVD
DPALESPVSSIHEPYSSLVVDSQESSLPEQVFTITDVSPTCVSSTEKSKVLVTGFFLKDYMHLSKSNLLCVC
GDVSVPAEIVQVGVYRCWVSPHSPGFVNLYLSIDGHKPISQVVNFEYRTPALHDPAVSMEESDNWDEF
RQQMRLAYLLFAKQLNLDVISSKVSPNRLKEARQFALKTSFISNSWQYLIKSTEDNQIPFSQAKDALFGIT
LKNRLKEWLLERIVLGCKTTEYDAHGQSVIHLCAILGYNWAVSLFSWSGLSLDFRDRFGWTALHWAA
YCGREKMVATLLSAGAKPNLVTDPTPQNPGGCTAADLAYMRGHDGLAAYLSEKSLVQHFNDMSLAG
NISGSLETSTTDPVNPANLTEDQQNLKDTLTAYRTAAEAASRIHAAFREHSLKLRTKAVASSNPEAQAR
KIVAAMKIQHAFRNHETKKMMAAAARIQCTYRTWKIRKEFLNMRRQAVKIQAAFRCFQVRKHYRKIL
WSVGVVEKAVLRWRLKRRGFRGLQVKTVDAGTGDQDQQSDVEEEFFRTGRKQAEERVERSVVRVQA
MFRSKKAQEEYRRMKLALNQAKLEREYEQLLSTEVDMQL

>GmCAMTA14

MAGLEYSIDDLFQEAKRRWLKPVEALYILRNHDQCKFTHQPPHQPAGGSLFLFNRRIMRSFRKDGHN
WRKKKDGKTVGEAHERLKVGNVEILNCYYAHGEENRTFQRRSYWMLEPEYDHIVLVHYRETSEGKSN
SEHVTQLPSEVTFEAQALRQLEEQLSLNDDGFNEIALDLVSGQDQRVVYKQDKSAALSGPNDLGQPCD
GYNGRQDDSGTYYHDFLDDCPGGNEKTIYWTKVLESCKPLSVTKLPDQHAYEAIGNENTLFSSGRGVI
ANLENNQWLNSNSNNIENYGGVKFPPYSLAETPGANSDYYETFFDQFQNQGPLGVDSSLTVVQKQKF
TIRAVSPEYCYSTETTKVIIIGSFLCHDSDSTWACMFGDVEVPAEIIQDGIICCEAPSNHLGKVNLCITSGN
RVPCSEMREFEFRNKTTSCTRCNSLETEGSKSPEDLLLLVRFAEMLLSSSTTKDDRIESGSHLSTEQKDDD
DSWSHIIDTLLDSTRTPSDAVKWLLEELLKDKLQLWLSNRRDEGTGCSLSKKEQGIIHMVSGLGFEWAL
NPILSCGVNINFRDINGWTALHWAARFGREKMVASLIASGASAGAVTDPSSQDPTGKTAASIAASHDH
KGLAGYLSEVDLTSHLSSLTLEESELSRESSELEAELTVSSVSEENLVASEDQVSLKASLDAVRNAAQAAA
RIQAAFRAHSFRKRKERDAAATVLDGYCIDAGSIDNNISVLSAMSKLSSQSWRDYKAALSIQKKYRNW
KGRIEFLALRQKIVKIQACVRGYQVRKQYKLILWAVGILDKVVLRWRRKRIGIQSVRQEMESNEEESDD
ADFLNVFRKEKVNAAIEKALKRVLSMVHSTGARQQYRRLLSLYRQAKIEHGSTSDEAPLSTSEENASNM
EDDDLCQFLDTFFWPS

>GmCAMTA15

MTPGYEYDINDLHQEAQARWLKPAEVMYILQNHEKFQFTQEVPQQPTSGSLFLFNKRILRYFRRDGHN
WHKKSGGRTVGEAHERLKVLNVEALNCYYARGEQNPAFQRRSYWMLDPAYEHIVLVHYRNTSEGKL
SSGAGAQLSPSSSVYTQSPSPYSTQNPGSTSILGDSYEPNQSFSSPGSTKVTSEIFVLNNKMGHMDWADTE
SGTSSELEVTQALRRLEVQLSLNEDNFEDIVSFGSKHETVHDSNPKHDQRVISNQEQSAAFSRPDDQGLF
YDGCNGRQDHGYPDANEKALWTEQLESHKSSSAVKLPQKNVYMPAENENSVSSARRVPVSNQENSH
WLNFNCNNSENSVFSQPQGVDEVKFPAYSSMLETQVINSDYYETLFDQSQIGAPPDANSSLTVAQKQK
FTIKTISPEWGYATETTKVIVVGSFLCHPSDSAWACMFGDVEVPIEIIQDGVISCEAPSHLPGKVTLCITSG
NWESCSEVREFEYHDKTNSCTRCTQSETEATRSPEELLLLVRLGQMLLSASTIKNDNIESGIPLIKPKADD
DSWSHIIDALLVGSGTSSGTVDWLLEELLKDKFQQWLSFRSREKDEETGCSLSKKEQGIIHMVAGLGFE
WALNPILTCGVNINFRDINGWTALHWAARFGREKMVASLIASGASAGAVTDPNAQDPTGKTAASIAA
SSGHKGLAGYLSEIAVTSHLSSLTLEESESSKSSAYLQADRTVNSVSKENLTANEDQASLKDTLAAIRNVT
QAAARIQSAFRSHSFRKRRAREATASTGGIGTISEISAMSKLAFRNSHEYNSAALSIQKKYRGWKGRRDF
LALRQKVVKIQAHVRGYQVRKHYKVIWAVGILDKVVLRWRRKGAGLRGFRQEMDINENEDEDILKVF
RKQKLDVEIEEAVSRVLSMVDSPDAREQYHRMLEKYRQAKAELAGTSDEASLSTSVGDDLFMDDFYPF
P

>SlCAMTA1

MADSRRYGLNAQLDIEQILLEAQHRWLRPAEICEILKNYQKFRIAPEPPNRPPSGSLFLFDRKVLRYFRK
DGHSWRKKRDGKTVKEAHERLKAGSIDVLHCYYAHGEENENFQRRSYWMLEEEMSHIVLVHYREVK
GNRTNFSRIREPQQVTPDLQETDEDVHSSEVDSSASAKFYPNDYQVNSQVTDTTSFSSAQASEYEDAESV
YNQHPTSGFHSFLDAQPSAGDGLAVPYHPIPFSNDQVQFAGSSGTSFSSIPPGNGNTSTANTYVPSRNLD
FASWGTISVNNPAAYQSLHFQPSGQSSANNMMHEQGNTTMGQICSNDFTRQEHENHIDGLGNWQTS
EVDSSFISKWSMDQKLNPDLTSGQTIGSSGVYGVEHHNSLEASQLLPAQQDKHPIQNELQSQLSDANIG
GSLNADLDHNLSLGVKTDYSALKQPLLDGVLKREGLKKLDSFDRWVSKELGDVSESHMQSNSSSYWD
NVGDEDGVGNSTIASQVQLDTYVLSPSLAQDQIFSIIDFSPNWAFSGSEIKVLITGRFLKSQQEVENCSWA
CMFGELEVPAEVIADGVLRCHTPVQKAGRVPFYITCSNRLACSEVREFEFRVTEGQDVVANPNSCSSSES
LLHMRFGKLLSLESFVSQTSPPISEDNVSYISSKINSLLRDDDNEWEEMLHLTNENNFMAEKVKDQLLQ
KLLKEKLHVWLLQKVAEGGKGPNILDEGGQGVLHFAAALGYDWAVPPTIAAGVSVNFRDVNGWTA

LHWAASYGRERTVGFLISLGAATGALTDPTPKHPSGRTPADLASSNGHKGIAGYLAESSLSSHLFSLELK
EKKQGENEQAFGEAVQTVSERTATPAWDGDWSHGVSLKDSLAAVRNATQAAARIHQVFRVQSFQRK
QLKEYGGSEFGLSDERALPLLAMKTNRAGQHDEPHAAAVRIQNKFRSWKGRRDFLLIRQRIIKIQAHV
RGHQVRNKYKNIIWSVGILEKVILRWRRKGSGLRGFKPEAPTEGSNMQDQPVQEDDYDFLKEGRKQTE
ERLQKALERVKSMVQYPEARDQYRRLLNVVSDMQEPNSTAASYNSAEAVDFNDDLIDLGDLLDDDTF
MPTAP

>SlCAMTA2

MDITQILSEVQHRWLRPAEICEILRNHRKFHLTPEAPFRPVSGSVFLFDRKVLRYFRKDGHNWRKKKDG
KTVKEAHEKLKVGSIDVLHCYYAHGEEDDNFQRRSYWMLEQDLMHIVFVHYLEVKGNKVNVSSIRST
KSVHPNYPNDCSLSDSFSTRHKKLTSANADSTSLASTLTEAHEEAESEDSHQACSRFHSYPDRASGMDS
HLVENGDTISSSYGSPQSSVEYTPLPGIDGSGKCDLGNFASGPQRTIDLGSWEPLPQHCLNGEMVCQDD
FKNNLSVHGNWQYSFGQSPLQFHGQNVNQDLIADSSYDLGLPSDLLTVRGPSYLYSNEKEEQLAQLNL
QFLKSLVEVQGDINQENSMDMLELGDYSTIKQPHLSSVKVEEGLKKVDSFSRWVAKELEDVEELHMQP
SNQMSWNVIDTEEEGSCLPSQLHVDSDSLNLSLSQEQVFSIIDFSPNWAYSNLETKVLITGRFLKSEGELV
EYKWSCMFGEVEVPAEVLADGVLRCHAPPHKPGVLPFYVTCSNRLACSEVGGFEYRFGPYQEVGAAD
VSMTEKHLLERIENLLSLGPVSSCRSSDSMEDSEEKRSTVNKIIPMMEEENQPIIERASYGDTSQCGVKED
LYFERKLKQNFYAWLVHQVTDDGRGRTLLDGEGQGVLHLVAALGYDWAFKPILASGVSVDFRDMNG
WTALHWAAFYGREKTVVSLVSLGASPGALTDPSAEFPLGRTPADLASANGHKGISGFVAESSLTTHLSK
LTVTDAKEELDSEVCEAKVGETVTERVAVSTTENDVPDVLSLKDSLAAIRNATQAAARIHQIFRVQSFQ
RKQIIEHCDNELSSDENAIAIVASRACKLGQNNGIAHAAAIQIQKKFRGWNKRKEFLLIRQKIVKIQAHI
RGHQVRKKYEPIIWSVGILEKVILRWRRKRSGLRGFRSEAVMSKPSTQEDSLPEDDYDFLKEGRKQTEVR
MQKALARVKSMTQYPEGRAQYRRLLTAAEGLREVKQDGPIQIPEIPEDTIYPEEELFDVDSLLDDDTFMS
IAFE

>SlCAMTA3

MAESGYNTNDLVQEGRFRWLRPAEVLFILQNHDDRQLAHQPPQKPASGSMFLFNKRVLRYFRKDGHS
WRKKKDGRTVGEAHERLKVGNAEALNCYYAHGEKNSNFQRRSYWILDPAYEHIVLVHYRDITEDESR
PGYGEICSDAVIHSNGMNVSDITRMMEGVSNSPKVEISQALRRLEEQLNLNDDSSSDIYSLYSEIENSND
AENVVHDKSSLVQIQDNSNNFLFLPHSGESSESRDQLLNLDNSMWKEMLDHCRSSPASQPQAKCFEKL
DENGMLQTSSGSEPIEAIKSDRWPIIGGKEALKCSVTNLKQVDDFKYIGCAQINVFGSYPDQCTTIFDQD
QIGISSETNMSLTIVQKQKFTIHDISPDWGYASDATKVVIIGSYLCNPSEYTWTCMFGDTEVPVQIIKDGAI
RCQAPPHLPGKVALCVTTGNRTPCSEVREFEYRAKFDDRGQNVVPEVGGASKSSEELLLLVRFVQMLLS
DSSVQIGDGSELSNDILEKSKASEDSWSQVIESLLFGTSTSTVTIDWLLQELLKNKLQQWLSSKLQVKNNE
MVYSLSRKDQGIVHMIAGLGFEWALHPVLNAGVSANFRDIRGWTALHWAARFGREKMVASLIASGA
FAGAVTDPSSQDPFGKTAASIASSCGHKGVAGYLSEVALTSHLTSLTLEECDVSKGTADIEAEQTISNITT
TSPVTHEDQLSLKDTLDAVRNAAQAAARIQSAFRAHSFRKRRLREAAHVATTCRDEYCILSNDVLGLS
AASKLAFRNVRDYNSAALSIQRKYRGWKGRKDFLVFRQKVVKIRAHVRGYQVRKEYKVCWAVGILEK
VVLRWRRRGVGLRGFRLEDEPIEESENEDILKLFRKQKVDAAINEAVSRVLSMVDSPEARQQYRRILEKY
RQAKVSQSSI

>SlCAMTA4

MAESGYDINDLVREAQIRWLKPAEVLFILRNHENHQLSSEPSQKPPSGSLFLYNKRVLRFFRKDGHSWR
KKKDGRTVGEAHERLKVGNAEALNCYYAHGEQNPSFQRRSYWMLDPAYDHIVLVHYRDIIEGRQNP

AFMSESSPISSAFSPSPSSYSTPHTGSTGIASECYEQYQNQSSPGEICSDAIINNNGTTDTIGRTEEVISSPGLE
MCQALRRLEEQLSLNDDSLKEIDPLYGDAINDDSSLIQMQGNSNRLLLQHHSGESSESHHRDLTQDAH
VWKDMLDHYGVSAAAESQTKYLHKLDENAMLQTLSERRAIEAYESYKWRDFSDKETQTAPVQAFKQ
LEDFKYPTYPPDITTFGSNPDEYTTIFDQDQIGTSLEDEMSLTIAQKQKFTIRHISPDWGYSSEPTKIVIIGSF
LCNPSECTWTCMFGDIEVPIQIIQEGVICCQAPRHLPGKVTLCVTSGNRESCSEVREFEYRVKPDDCARN
NQPDVEGAYRSTDELLLLVRFVQLLLSDLSVQKRESSELGNDLLEKSKASEDSWSQIIESLLFGTSVPMVT
IDWLLQELLKDKFQQWLCSKLQQKDNQIDCSLSKKEQGIIHMVAGLGFEWALHPILNAGVSANFRDIN
GWTALHWAARFGREKMVASLIASGASAGAVTDPSSRDPVGKTAASIASCCGHKGLAGYLSEVALTSH
LSSLTLEESELSKGTADVEAERTISSISNTSATINEDQRSLKDTLAAVRNAAQAAARIQSAFRAHSFRKRQ
QREFGVSATTSVDEYGILSNDIQGLSAASKLAFRNPREYNSAALAIQKKYRGWKGRKDFLAFRQKVVKI
QAHVRGYQVRKQYKVCWAVGILEKVVLRWRRRGVGLRGFRHDPESIDEIEDEDILKVFRKQKVDAAL
DEAVSRVLSMVESPGARQQYHRILEKYRQSKAELEGADSETASTAHGHV

>SlCAMTA5

MESNRAGQLTGKEIHGFRTLQDLDIPSILEEAKMRWLRPNEIHAILCNYKYFNIFVKPVNLPTSGTIVLFD
RKMLRNFRKDGHNWKKKKDGKTVKEAHEHLKVGNDERIHVYYAHGEDLPTFVRRCYRLLDKSLEHI
VLVHYRETQETRGAPETSVAKSSPATPVNSSSSSDPSDPSGWILSEECNSVDEQAYGASQHANLEPNRD
MTAKTHEQRLLEINTLDWDELLAPNDPNKLMATQEVGGRASVGQQSQCEVNGYSLNDGSSSMARAPI
ASLESFVGQVAGSDAVNFNPLNDMSFRSGDGQMTSNFQKKESGVMTVGAGDSFDSLNKDGLQTQDSF
GRWINYFISDSSGSADELMTPESSVTIDQSYVMQQTFNITEIFPSWALSTEETKILVVGHFPGRQSPLAKSN
LFCVCADVCFTAEFVQSGVYRCVISPQAPGLVNLYLSLDGNTPISQVMTFEFRAPSAHKWTDPLEDQSN
WDEFRVQMRLAHLLFSTSKSLSIFSSKVHQNSLNDAKKFVRKCAYITNNWAYLIKSIEGRKVPSMHAKD
CLFELSLQTKFHEWLLERVIEGCKTSERDEQGQGVIHLCAILGYTWAIYPFTWSGLSVDYRDKHGWTAL
HWAAHYGREKMVATLLSAGANPNLVTDPNSENPDGYTAADLASKNGFDGLGAYLAEKALVAHFEA
MTLAGNVSGSLQTTTEPINPENFTEEELYLKDTLAAYRTAADAAARIQAAFREQSFKLQTKAVESVNQE
TEARNIIAAMKIQHAFRNYESRKKLAAAARIQYRFRTWKMRKDFLAMRRHAIKIQAVFRGYKERKQYR
KIVWSVGVLEKAVLRWRLKRKGFRGLQVQSSESVDIKPDGEVEDFFRASRKQAEERVERSVVRVQAMF
RSKRAQEEYSRMKMAHNNALLEYKRLINPDN

>SlCAMTA6

MESSVSGRLLGCEIHGFRTMQDLDIPNIMEESKMRWLRPNEIHAILCNHKYFNINVKPVNLPKSGTIVLF
DRKMLRNFRRDGYNWKKKKDGKTVKEAHEHLKVGNDERIHVYYAHGEDNTTFVRRCYWLLDKTLE
HVVLVHYRETQEVSSNSTVAQGSPAAPVSSGSALSDPADLSASWVLSGELDSAVDQQYSASRHAHLEP
NRDMTVQNHEQRLLEINTLEWDDLLAPGDPNKMVATQQAVGKTAYVQHTSYEQRNLCELNGYSFD
GGVSSSLERISTFNNSNEITFQTVDGQMTSSFEKNESGVMTVSTGDSLDSLNQDRLQTQDSFGRWMNYL
IKDSPESIDDPTPESSVSTGQSYAREQIFNITEILPAWAPSTEETKICVIGQFHGEQSHLESSSLRCVCGDAC
FPAEVLQPGVYRCIVSPQTPGLVNIYLSFDGNKPISQVMSFEFRAPSVHVWTEPPENKSDWDEFRNQMR
LAHLLFSTSKSLNILSSKIHQDLLKDAKKFAGKCSHIIDDWACLIKSIEDKKVSVPHAKDCLFELSLKTRL
QEWLLERVVEGCKISEHDEQGQGVIHLCAILGYTWAVYPFSWSGLSLDYRDKYGWTALHWAAYYGRE
KMVATLLSAGAKPNLVTDPTSENLGGCTASDLASKNGHEGLGAYLAEKALVAQFKDMTLAGNISGSL
QTTTESINPGNFTEEELNLKDSLTAYRTAADAAARIQAAFRERALKVRTKAVESSNPEMEARNIIAAMKI
QHAFRNYEMQKQLAAAARIQYRFRTWKMRKEFLHMRRQAIKIQAVFRGFQVRRQYRKIIWSVGVLEK
ALFRWRLKRKGLRGLKLQSTQVTKPDDVEEDFFQASRKQAEERIERSVVRVQAMFRSKQAQEQYRRM
KLEHDKATLEYEGTLNPDTEMD

>SlCAMTA7

MAVDLEQILKELHHRWLLPHEVCQILRNHQSFCLTQQLQLKPPAGSIFLYDRKLLPNFCKDGHHWRK
NKDGQTIKEAHEKFKAGSVDVLHCYYVHGEGNKNFQRRSYWMLEEQLEHIVLVHYRDVKEGYRLGA
SRLQPVHPGLLLENPDSSSKPCFVFGPAFQKSHTSNPSLVDLKEQALSSELHSGDSKGLVAFSRSKERFQL
NPQVRAFMSSGFRKFERNLNVMLQRKFYSGHYNLADLRSSKLTYAKLYAGKAVANNRSRLAITSGKVF
EENIHVAPPQIQNISSSQTVVTPDAAVKTSSLDGGLNSDEVGSLKKLDILGKWMDREFAGGNKSLMSSD
SGNYWNTLDTDNGDKEVSTLSRHLLLEANSVGTSPSQKQLFRIFDFSPQWAFSGVETKVLIVGTFLVHR
KYLTCLKWSCMFGEVEVSAEVQTQSIRCQVPFHAPGHVPFYVTCGNRLACSEVREFEYREKSSELALAL
RPSDEVHLQVQLVKLLYSGLNKKFLDCSSRECENCKLKTQLCSLKCQTGNATERLEDLLAVIECDHINF
KDVQIQNFMKDKLYEWLVSRAHEEDKGPNILNDQGKGVIHLVAALGYEWGLLPLIAAGISPNFRDAC
GRTALHWAAHYGREDMVIALIKLGVAAGAVDDPTTASPGGRTAADLASSRGYKGIAGYLAESDLTSH
HQLLATSKNALDTIGAGLEAEKVYESAVQEIVPLNGTIDDDVSLKASLASLRKSAHAAALIQAAFRARS
FRQRQLRESRNDVSEASLDLVALGSLNKVQKVNCFEDYLHSAAINIQQKYCGWKGRREFLKVHNQIVK
MQALVRGHEVRKQYKKFVWAVSILEKGILRWRRKKTGLRGFWPEKTSETGIVEREKEEEYDYLSIGLKQ
KCAGVEKALGRVESMVRHPEARDQYMRMVAKFKSCKLDDGGREVNRSSPPV

>ZmCAMTA1

MQQQQQGLDIGKLQEVVKTRWLKPQEVLKILQNHELFTISHKPPQKPQSGSWFLFNRRVLRYFRNDGF
EWQKKRNGKTINEAHERLKVDNVDALNCYYARGDKNPTFQRRIYWMLDPAYEHIVLVHYRDVLEGSI
SVSARNDSSTLNQNGSASRAEVHSSPGWTSELIAHCTNSCSPGSAEEVSSQISASESDLIQHKAALRKLK
QLSLEDKEDCDVNAEDVPADNEPIILPVIQNEEPGTSRNHDDIFDVLDFSEDHINGTGTHPCHSAIDVLK
NSDTWLEDDQLEAILHPACMTLTENQWFRIHEVSPESAFSYESTKVIIVGDFLCNPPHSSWQVLFGDVK
VCVEIIQQGVIRCHTPCLDAGKVRMCLLDGNGKSCSEAREFEFLEKPTKCMIDGNTNPCNEAQDVKLH
QIPTKSSEELSLLLHYVHTLFDGHASGLFSNFSLPLQNLGCGIQSNQMDVMKKAYKQLDPENVVSSVME
VLLNDKFKQWLSSKCEQNIDGDHLLPKQYRNIIHTVAALGYDLALKPLLSSGVPINYRDANGWTALH
WAARFGREDMVVALLTAGAAAGALSHPTSEDPAAKTPASIALAYGFKGLSAFLSEAQLTTHLDSIESKE
NGKLDSREEGICRAVDRISDKSSHVHGGTDDQLALKDSLGAVRNAVQAAGRIQAAFRIFSFKKKKEMA
LGNRNSCCLSISEAGAVSHDMLEKAVLSIQKNFRCWKKRKEFLKMRNNVVRIQARVRAHQERNKYKE
LISSVGILEKVMIRWYHKGVGLRGFNSGAMTIDEEVDEDVAKVFRKLRVETAIDEAVSRVSCIIGSPKAM
HQYRRMLNRYQQTKDDQEK

>ZmCAMTA2

MPRSGSLFLFDRKVLRYFRKDGHNWRKKKDGKTVKEAHEKLKAGSIDVLHCYYAHGEENENFQRRTY
WLLEEDFTHIVLVHYLEVKGCKQSFNRVKEELMQLSNVDSPSCSNSITSQNQMGPQNMEAAESPISGQI
SEYEDTESDNCRASSRYHPLAEMQQLVDGVITENMLYPSASTVGSRQGYHGEMLPITDNLDNRSFSNH
DIARMLDGANIGLSDVSSTLFDSVPFNEPFTNYSAGFTEPTLHSSFACLEANDLDDSSCLQTFTSEALYTN
HLNQKEADALGFTGILASEVNRDRYNDGSIKHSLLKQSSLDLLTIEAPGLKKNDSFSRWMSKELEELDA
YVVNPSLSQDQLFSILDVSPSCAYIGTNTKVSVTGTFLVNKKHVESHRWSCMFGDVEVPAEVLTDGTLR
CYAPAHQSGRVPFYVTCSNRVACSEVREFEYRDSEAHYMETSRSQANGVNEMHLHIRLEKLLTLGPDD
HQMLVISSGNEKYEIMNAINSLMLDGKWSNQESSVKEVVSTARVQSLKKLVKEKLHQWLICKVNDDG
KGPNVLCKEGQGVIHLVAALGYDWAIRPIMIAGVNVNFRDAHGWTALHWAASLGRERTVSVLIANG
AAAGALTDPTSEFPSGRSPADLASVNGHKGIAGFLAESALTSHLSALTIRESNDSTVEACGLPFAEDLTGI
DSVHLAGEGPDAESLEGSLSAVRKSTQAAARIFQAFRVESFHRKKVVEYGDDTCGLSDECTLSLVSLKN

VKPGQHDTHLHSAAVRIQNKFRGWKGRKEFMIIRQRIVKLQAHVRGHQVRKNYRKVVWSVGIVEKVI
LRWRRKRPGLRGFRPEKQLEGPSQIQPAKAEDEYDFLHDGRRQAEARLQRALARVHSMSQYPEAREQ
YHRLTTCVAEMKQSRMMQDEMLSQAAGGADDFMAGLEDLICIDDAPPRCLPFGEAEPFYIR

>ZmCAMTA3

MAEARRHAVAPQLDIEQILKEAQHRWLRPAEICEILKNYRNFHIAPEPPNRPPSGSLFLFDRKVLRYFRK
DGHSWRKKKDGKTVKEAHERLKSGSVDVLHCYYAHGEGNENFQRRSYWMLEEDFMHIVLVHYLEVK
GGKSTSRIRGHDDMLQAARTDSPLSQLPSQTTEGGNSLSGQASEYEETESDIYSGGAGYHPFSWTQHHE
NGGGPVIGTSIPSSYVPALPLGNLQGFPATVTNTDIYSRQDALPVTLNEPGLAIEFNGADNQLDPSSLNG
LVKPFLQMSTPQSTVPSELFPFTEEHGNESFTFHEVYSNGLSIKDAEIVGTNEESVWKLPGAISSIPSEDSFQ
QNDRSLEETISYPLLKTRSSNLSEMLKDSFKKSDSFTRWMSKALGEVDSQIKSSSGVYWNSEETNNIIETSS
CDQLDQCTIDPVLAQEQLFSIVDFSPSWTYAGSKTRVLINGKFLNSAELKRCKWSCMFGEVEVPAEISAD
GILRCYSPSHKPGRVPFYVTCTNRLACSEIREFEFRPSVTQYMDAPSPHGATNKTYLQMRLDNLLSLGH
NEYQATVSNPTKEMVDLSKKISSLMTDNDSWSQLLKLASDNEPVTDDDQDEFFEKHLKEKLHIWLVH
KASDGGKGPNVLDDEGQGVLHLAAALGYDWVIRPAVSAGVNINFRDAHGWTALHWAAFCGRERTV
VALIALGAAPGALTDPTPIFPTGSTPADLASANGYKGISGFLAESSLTSHLQTLDLKEGKGSNPPEISGLP
GIGDVTERRASPLAGEGLQAGSMGDSLGAIRNAAQAAARIYQVFRVQSFQRKQAVQYEDDNGAVSDD
RAISLLSVKPSKPVQLDPLHAAATRIQNKYRGWKGRKEFLLIRQRIVKIQAHVRGHQVRKHYRKIIWSV
GIVEKIILRWRRKGAGLRGFRSTEGATVGSSSNLIQNKPEDDYDFLQQGRKQTEERLQKALARVKSMAQ
YPDARDQYQRILTVVTKIQESKAMQEKMLEESTEMDEGFFMSDFKELWDDDIPMPSWS

>ZmCAMTA4a

MSQSFDINVLREEARSRWLKPSEVYYILQNHERFPITHEAPKKPPSGSLFLYNRRVNRYFRRDGHTWRRK
KDGRTVGEAHERLKVGNVDSLSCYYAHGEQNPCFQRRCFWMLEPAYEHIVLVQYREVAEGRYYSSQL
SNGPPEPLSSLGYPNAICGNQYHRSTSGTSEGSESHQSYSNLSSVTEVSSYSGNKEYNKNDGSLLSIPEVG
HTCQQNQTGNGNSKNKSELNMALKKIAEQLSLGEDDDDDYIYSNQTHSMGGDNQIKQIRQEGTQKG
LSRNIAPSWEDVLHSSSGLPTSSIYQQSDVKYQKKSEYQPPEILDSSDLRIQLSATKRFLLGPEASIDSPSLN
SVLRNRVNSVTDTISAYDSRFESSLNPDWQTKTALTFQSNSQGSEITELFDHDHFEPYSREDTTISLGQTN
KFNIREVSPEWAFSYEITKVIITGDFLCDPSNLCWAVMFGDNEVPVEIVQPGVLRCHTPLHSNGNLRICIT
SGNREVCSEFKDFEFRSKPTSSSFTDIAPSSRHLKSSEELLLLAKFARMLLSGNGNREVPDGDPQSGQCPK
LKTNEELWDRLINELKVGCENPLSSVDWIVEQLLKSNLQQWLSVKLRGFNGTDFLSKQEQGIIHLISALG
YEWALSPVLSAGVGLNFRDSNGWTALHWAAYFGREKMVAALLAAGASATAVTDPTAQDPVGKTAA
FLASERGHTGLAGYLSEVSLTSYLASLTIEESDVSKGSAEVEAERAVEGISQRNAQRHGGTEDELSMKDS
LAAVRNAAQAAARIQNAFRAFSFRKRQQKTARLRDVYGMTQEDIDELAAASRLYHQAHASSGQFYD
RAAVSIQKKYKGWKGRKHFLNMRRNAVKIQAHVRGHQVRKKYRTIVSTVSVLEKVILRWRRKGHGLR
GFRAEQQPMVEAIEEDDEEDDDFDDDEAVKVFRRQKVDQAVKEAVSRVLSMVDSTEARMQYRRMLE
EFRQATAELEGSNEVTSIFDSDLELLGINNFM

>ZmCAMTA4b

MGFNRILTNGAPIGLFFGGSLFLYNRRVNRYFRRDGHTWRRKKDGRTVGEAHERLKFFVHVFLQTWSK
HSHRKQGRCRIPWPRVIVPNCGSGSPWVIVPNARESLLQHSHDARTCVSVGQVEFHGCFRLSQMNAYY
GALGAFGQVGNVDALSCYYAHGEQNPSFQRRCFWMLEPAYEHIVLVQYREVDVVRNYSSQLSNGLPE
PLSSLGYPNAICGNQYHSSSSGASECSESHQSYSNLSSVTEVSSYSGNKEYTKNDGGLLSIPEVGHTCLQQ
NRTDNGNSKNKSGLNIALKKIAEQLSLGEDDDDYIFSNQAHSVGGDNQVKHIQQEGTQKGLSRNIAPS

WEDVLQSSSGLPISSIYQQSDVEYQKNSEYHPPERLDSSDLRIQLAAAKKFLLGPEATVDSPSLNSVLRNR
ANCVTDTISAYDSRFGSSLNPDWQTKTALTFQSNSQGSEITELFDHGHFEHYSREDTTFSLGQTNKFNIR
EISPEWAFSYEITKVIITGDFLCNPSNLGWAVMFGDSEVPAKVVQPGVLLCHTPLHCSGNLRICITSGNR
EVCSEFKDFEFRSKPSSSFTDIAPSSRHLKSSEELLILAKFARMLLSGNGNPEVPDGDPQSGQCPKLKMDE
GLWDRLIEELKVGCESPLSSVDWILEELLKSKLQKWLSVKLRGFNGTDSISKHDQGIIHLISALGYEWALS
SVLSVGVGLNFRDSNGWTALHWAAYFGSCSFRRNTKVAKVATFLNMRRNAVKIQAHVRGHQVRKKY
RTIVSTVSVLEKVILRWRRKGHGLRGFRAEQQSMVEAIEEDDEEDDDFDDDEAVKIFRRQKVDQAVKE
AVSRVLSMVDSTEARMQYRRMLEEFRQATNWKDQMK

>ZmCAMTA5

MAGGAGGRDPLVASEIHGFLTCADLNFDKLMMEAGTRWFRPNEIYAVLANYARFKVHAQPIDKPISG
TVVLYDRKVVRNFRKDGHNWKKKKDGKTVQEAHEKLKIGNEEKVHVYYARGEDDPNFFRRCYWLL
DKELERIVLVHYRQTSEESALPPSHVEAEVAEVPRINMIHYTSSTDSASAHTELSSSAAAAPEDINSNGGG
AVSSETDNQGSSLESFWVDLLESSMKNDTPVDASACGGSLVSNQQTNNGMGDSGNNILYINATSNAIF
SPPTNVVSEAYANPGLSQVSESYFGSLKDQANHAPSLLTSDLDSQSKQHTNSLMKTPVSDNMPNDVPA
RQNSLGLWKYLDDDISLDDNPSSGILPTEQVTGEIPFQITEISSEWAYCTEDTKVLVVGCFHENYRHLAG
TNLFCVIGDQCVDANIVQTGVYRFIARPHAPGRVNLYLTLDGKTPISEVLSFHYRMVPDSQNLAEDEPQ
KSKLQMQMRLARLLFTTNKKKIAPKLLVEGSKVSNLLSASTEKEWMDLSKFVTDSKGTYVPATEGLLEL
VLRNRLQEWLVEKLIEGHKSTGRDDLGQGPIHLCSCLGYTWAIHLFSLSGFSLDFRDSSGWTALHWAA
YCGREKMVAALLSAGANPSLVTDPTHDVPGGQTAGDLAAGQGYHGLAAYLSEKGLTAHFEAMSLSK
GK

RSTSRTESLKRNTKEFENLSEQELCLRESLAAYRNAADAASNIQAALRERTLKLQTKAIQLANPENDAS
AIVAAMRIQHAYRNYNRKKMMRAAARIQSHFRTWQIRRNFMNMRRQAIKIQAAYRGHQVRRQYRK
VLWSVGVVEKAILRWRKKRKGLRGIATGMPVAMATDAEAASTAEEDYYQVGRQQAEDRFNRSVVRV
QALFRSHRAQQEYRRMKVAHEEAKVEFGRK

>ZmCAMTA6

MDAESPISGQISEYEDAETDNSRASSRYHPFTEMQQPVDGTVMGNFFGASSPSVSVNNLAAGYLGEMQ
PTGANFTSHFATRNDIASVFNDTGSELGGGPKTSIDSVLLGEPFPEYPGGFMESTLYSSVATLGNSLEDGL
QTFMSEALYTNNLTQKEVDALGAAGITSSKYLKNDAVGHWSLPISMSWYDVTLYFQTENDGYTDQSV
RYPLLKQSSSDLFKMEPDGLKKFDSFSRWMSNELPEVVDLDIKSSSDAFWSTTETVNVADGSSIPINEPLD
VFVVSPSLSQDQLFSIIDVSPSWAYNGTKTKVLITGTFLAKKEDVENCSWSCMFGDSEVSAEVLVDGSLR
CYTPVHHSGRVPFYVTCSNRVACSEVREFEFRDSETHYMDISDKHTTGINEMHLRIRLDKLLSLEPEDYE
KYVLSNGNKSELINTISSLMLDNNLSNLALPSDEKELCTVQDQNLEKQVKEKLYYWLIHKIHDDGKGP
NVLGKEGQGAIHLVAALGYDWAIKPIVAAGVNINFRDIRGWTALHWAACCGRERTVGALIASGAASG
ALTDPTQQYPSGRTPADLASENGHKGIAGFLAESALTSHLSALTLKESPSGNVEEICGLTAAEGFAASSSS
QLACVNSQEESLKDSLGAVRKSTQAAARIFQAFRVESFHRKKVIEYGDDDCGLSDERTLSLVSLRNPKSG
HGDSHSAAVRIQNKFRGWKGRKEFMLIRQKIVKIQAHVRGHQVRKNYRKVVWSVGIVEKVILRWRRK
GRGLRGFQPEKQLEGPSWQIQPAKAEAEDEYDFLKDGRKQATGRLDRALARVRSMNQYPEARDQYRR
LQACVNSLRESQAMQDRMLADSAGTDGGDFMTELEELCRDDGDAPMSTIS

>ZmCAMTA7a

MLEEDYMHIVLVHYLETKGGKSSRARGNNIIQEAAVGSPSQIMEVESSLSGQASEYEEAESDIYSGGAGY
DSFTWMQQHENGTGPVIDSSLFSSYTPASSIGNYQGQHATQNKSFYPVNQHNGPLILNGSSDMLGTNG
RANQTDLPSWNSVIELDEPGQMPHLQFPVPSDQGATTEGLGVDYLTFDEVYSDGLSLNDIGAAGTHGK
SYLQFSSATGDLSATENSLPQQNDGSLEEAAIGYPFLKTQSSNLSDILKDSFKKTDSFTRWMSKELPEVED
SQIHSSSGGFWSTGEANDIIEASSHEPLDQFTVSPMLSQEQLFSIVDFAPNWTYVGSKTKILVAGNILNDS
QITERCKWSCMFGEVEVPAKILADGTLICYSPQHKLGRVPFYITCSNRLACSEVREFEFRPTVSQYMDAP
SPHGETNKVYFQIRLDKLLSLEPDEYQATVSNPSLEMIDLSKKISSLMASNDEWSNLLKLAVDNEPSTAD
HHDQFVEKLIKEKLHVWLLNKVGMGGKGPSVLDDEGQGVLHLAAALGYDWAIRPTLAAGVNINFRD
VHGWTALHWAAICGRERTVVALIALGAAPGALTDPTPDFPGSTPADIASANGQKGISGFLAESSLTSHL
QALNLKEANMAQISGLPGIGDVTERDSLHPPSGDSLGPVRNAAQAAARIYQVFRVQSFQRKQAAQSED
DKGGMSDERALSLLSVKPPKSGQLDPLHSAATRIQNKFRGWKGRKEFLLIRQRIVKIQAHVRGQQVRK
HYRKIVWSVGIVEKVILRWRRRGAGLRGFRSTEGSVESSNGGTSSSSIQDKPSGDDYDFLQEGRKQTEER
LQKALARVKSMAQYPEARDQYHRILTVVSKMQESQAMEEKMLEESAGMDFMSEFKELWDDDTPIPGY
I

>ZmCAMTA7b

MASAEARRLAVVPQLDIEQILKEAQHRWLRPAEICEILKNYRNFRIAPEPPNRPPSGSLFLFDRKVLRYFR
KDGHNWRKKNDQKTVKEAHERLKSGSIDVLHCYYAHGEENINFQRRTYWMLEEDYMHIVLVHYLET
KGGKSSRARGNNMIQEAAVDSPLSQLPSQTMEGESSLSGQASEYEEAESDIYSGGAGHDSFTWVQQHE
NGTGPMIASSVFSSYTPALSIGNYHGLHATQNTSFYPVNQLNSPVILNGSSAMLGTNGCANQTDLPSW
NSVIELDHEPVQMPDLQFPVPPDQGTSTEGLGVDYLTFDEVYSDGLSLQDIGATGTHGESYLQFSSGTG
DLAATVNSFPQENDGSLEAAIGYPFLKTQSSNLSDILKDSFKKTDSFTRWMSKELPEVEDSQIQSSSGAF
WSSEEANNIIEASNHEALDQFTVSPMLSQDQLFSIVDFSPNWTYVGSKTKILVAGNILNDSQITERSKWS
CMFGEVEVPANILADGTLICYSPQHKPGRVPFYITCSNRLACSEVREFEFRPTVSQYMDAPSPHGETNKV
YFQIRLDKLLSLGPDEYQATVSNPTLEMVDLSRKISSLMASNDEWSNLLKLAVDNEPSTADQQDQFAE
NLIKGKLHIWLLNKVGMGGKGPSVLDDEGQGVLHLAAALGYDWAIRPTLAAGVNINFRDIHGWTAL
HWAAFCGRESTVVALIALGAAPGALTDPTPDFPGSTPADLASSNGQKGISGFLAECSLTSHLQVLNLKE
ANMAQISGLPGIGDVTERDSLQPPSGDSLGPVRNATQAAARIYQVFRVQSFQRKQAAQYEDKGGMSD
ERALSLLSVKPPKSGQLDPLHSAATRIQNKFRGWKGRKEFLLIRQRIVKIQAHVRGHQVRKHYRKIVWS
VGIVEKVILRWRRRGAGLRGFRSQEGSVESSSGGTSSSSIQNKSSGDDYDFLQEGRKQTEERLQKALARV
KSMAQYPEARDQYQRIFTVVSKMQESQAMQEKMPEESAEMDMSEFKELWDDDAPIPGYF

GmCAMTAs 2000 bp upstream sequences for cis-motifs analysis

>Glyma.05G178200

ATGCCCCGATAAAAAAAAGAAAGCTACGTGTGACAACCCTATGAAGGAAAGAGATACCAACCA
ACTACATTCTGAAGGAGAGCACCATGAGTCACGACCAGGAGGTGGCTGTTCCAGCAGGTGAAAG
CCTAATTATGTGCATGTCGTGAGAAAGATAAGTCAAGTATTTCACAACTCAAAAAAAAAAAAAC
AAATTGAAAAATGCCAAGATGTCAAACGCCTTGGATAAGAATACGATCGACAATACTTTTCCGG
AATAGAAACTAAAATTATATATGTTTGTTTGATCGACCATGTTAAAACTTTAGTTCAGACTTTAGA
CAACAGAAAATGGTATTTTTTTTTCAATTAATAGACAGAAAACATATAATACGTCGTGTGTTTTGT
TTCAGGTTTCGCTTTCTTCGATGTGAATAATACATGCCTTATATATATTACTTTTCTCGGTTCTTTTT
ATCCACTAATAAACTACATTTTTGTTTATTTATTTATATATCTTTTTAATTATACCATTATCAATATC
TAGCTAATTCTTAAATACTTTTATACTCATCATAAATGAGAAAGAAATAATACTCTTTAGCAGCTA
CAAATAATTTTAACATCAACGAAAATTACTAATTTATATATATCTTTGACCTACAGATCAGGCTAT
TCAATGCGCGGGAGGAAGTCCAAACTAAATGTTAAACAAGAACACTGAAAATAACAAGATAGA
CGAGTCGGAGTACACGACACAACCCACAAAGCCCATTTATGACAAGTACTTAAGAACTGGCAAT
CCTTTTATTGATGTTTTAATACGGTATTTTCAATTTTTGGCTTTTCAGGTGTTGCCCTCAGCTGCCAT
TAGCTATTTTGTTTACGAGTTCATGAAGATAGTTCTCAAAGTAGAATCAACATAGATGATTATATA
TATTTGATCTTTGAAGATGGGAGTTTCTCCTCATTTCCTTAATCATGGTCACCTTTGTTTTTTTGTGT
GTTGGAATGTTATTATATGCGGGGGAATCGAGGGTGTCTCACACAAACAATTTAATGGTATGTTT
CTGTTGTATTTTACTGATTTTATGTTTACTTAATAATAATCCGAAGAAAGGAAAAGGAATGGGGCT
CAAAAAAATTTGATGCTTGGCTAATTAGACTTGAATTTTCCTTCATGAAGCAAATCCTAAAAACC
ATTATAATAGTTTTTTTTTATCTTTCTCTTTCCGACATAATATTGTGTCAAATCTTGTACGTAATTGG
ATATAGAACCTATTATTTTATAACAAACTTTTAAAATTAAAAAAAAATTGACAAATTGATTTTTTA
AGTTATGCAAATAAAGTATATATATTATCATGTGTGTTTGAAGAAATATTGATAACGGGTGTGTTT
GCTTTGGCAGTTTGAATTGTTTACCCTTTAATTTTCACGCCGACGGAAAAAAAATGACACAGAAG
CTATAATATGTGTGGATCATTGGCTTCAAACGTATCTAAGAAAATAATTTTGAATTTTAATTTTTAT
TAAAATTGATTTTAAAGTGATATAATTTATGTTTTAATATTAAAAAAATATGATTAATATTTAAAT
ATTTTTATTATAAAAACAAGTTAATAGTAATACTTAGTATATAATCTTTTAAGTGACACAAAAATT
ATTTCAATTAAAAATTAATTATTTTCATAAAACTATTCCCTAACAAGTGTAAAAGTTTACTTAAAA
TAACAATAACTAGTATATTAACGTGATTTTAGAAAATCCAGCATGAATAAAAAACATTAAATTTA
GAATATTCATCTCTAAACAATTTCTTTTTTGGTACATATGTTCACCTCTAAACAATTGACCCTAAAT
TTATTTTCTTAGCACATATCAAAACGCCGTCATATAATCCCCGAGTATAGGCCAAACAAAAATCT
GAGAAGCTGCGACGAAAAAGGAAGGATAAGCCAAATATCAGTCAAACTGGAATCACACAATGC
TATTTGACTTGGACAACCCTTTCAATGTCTTTTTCTTCTCTTTGATAGTACAACACAACACAACAC
AACAATGCTATCACAGACAAGTTCCCGCGTTGAAAACTTTTCTTTTTTCGTTCATTGGTATTTGGTA
CATCTATCTCCCTCTTAACACCCATTACACTCTCTCTCCGCGTTAAAAAAACTATCTGAACTTTCCT
AGTTATTACAATTTATTACACAACCCATCTCTCTTTTTTGTTTCGCTTAGATTTAGAAACACAGACT
TTTCCAAAGTGCTCGTTTACCTCAAACCTCAGAAAAATAAACATCGAGTTATCACTTGTTTTCTTT
TGTCGGGAGACAAAAACATTGATCAGTTCAGCTTCTCACTTCC

>Glyma.08G135200

TGAGATAAAGCGCTGCAGGTGAATGTTAAAATTACTCATGATTTCATTTTAAATTTTTTTTATACTT
TGAAGGTCGTAAGTAATTTTATTTTTTTCGGTTTTACAACTTTGAAGTTGTAAAACCTTTTTTCTAA
ACAAAAAATTGTTTAAGTTTTTTTTTTTTTGAAAATATAAAAGTTTATACTTTTTTATGGTTTATTTT

CTTCTAATTTTTTAAAATTATAAAAAAATTATTTAAATGTCAAATAAATAATTTTAATTTTACTTAT
TAATAGTTTTTTATTTAATATGGTGTATATTTTTAATGGTTATATTTTATATATTATTAATTTATTTTA
ATGGTTTATTATTTAAAATATATCATATATTTTTAATATTATTTTATTTAAATATTTTTATTTATTTA
AAATTTAGATAATCTATTAATAAAAATAGATAATGAAATTAAAAACAACATACTACATTAATAA
TGCACAATATTAAAGAAAAACATAAAAATATGCAACTAGCCGGATTTGTTTTTGAACATTTTTAC
TGTAAAAATAACCTAATAATAAAACTTAATATACAACTAGTCGGATTACATTTATATATATATAT
ATATATATATATATATATATATATATATATATATATATATATATATATATATATATATATATATATATT
TGATATGTAGATTATATTATAAGTTGTTAAATATCATATGTTACCGTCTAACAAATATAAATGCAA
ATATAATTAATTTTATGCATATGAACGTTATTGTAATTTTATAACCACGTCAAAACAACTCATTTT
GCTGAAATTCAAATAGTCACAATATATTACTTAAAATAATAAAGATAACAGTTATTTAAGAATAA
TCAATTTATTCAGTAAAAAAAAAAAAGAATAATCATTTGCACGATTAGTAGAAATGTTTTACGTTA
CTTTGAAGAGAAGTGGGGGTATGAGTAACTTAAATTAAGTGAATATTTGGGTGAGATTGCATGCA
AAAGAAACAAAAAAAAAAAATCTTTGCCGAACTTCTTCACTCTATTTTATCTAACACAAAAATTAT
TTAAAATTATTTCAATTCAAATTCAAATTAATTATGAACATAAAATTAATCCCTAACAGTGTAAA
AGTTTACATAAAATCACAATAATTTTTATATTAACGTGATTTAAAAAAAAATCGCATGAATCAAA
CACCATTAACTTTTAGAATATTCATCTCTAAACACTTTCTTTTTTTCCTCTAAACAATTGACACTAT
GTTTAAAGGAGTGATAATATATTTTCACAATATAAAATTTTAAATATTTAATCAATAATTTTATTTT
TTTATGTCACTTTCTTTTTTGCCTCTAAACAATTGACACTATGGTTAGTCTTGACATTCAAGACAAA
TATTAATTTAGATAACAAACTGAAAAAATGCATAAGAAAAAAGTATATTAGACATTTTTTATTCC
AAAAAAATGATATAAACATCTATGAGAGATGCATCTTGAAGAATATTAGAGAGAGAATAAAAT
ATATGTGATGAGAAAAAAAAAGTTTAGTAGTCAATAGAAAAAAAGTTTTAACAAAATCTCAAAGA
TTTAGGTGAGACGGTTTGATATATGTTTTATACTAAAAAAATATGATAAAATTCATCAAAAACCT
TTTTAAAAAATAATTCATCAAATTTTTTTTTTATAAGTTGTAAAAAAAGTTTTAATTAAATATCAG
CATATTTTATTATTTTATTCAAAAATCTTAAAAATCTTACTTAAATATCACAATACTTATTTAATCA
TTTAAAAAATTTGATTAAATAGTAAGTGTCTTTTAAAATCTTTTCAATTCTAATCTAATATATTCCT
TAATTTATTTGGACATATCAAAATGACGTCATACAGTCCCCGAGTTTAGGAAAAAAAACTGAGA
AGCTGCCACGAAAAAGGAAGGATAAGCCAAATATCAGTCAACTTGGAATCACAAGAATGCTCT
GACTTAGACAGCCATTTCTATAGTTTTTTTCTTCTCTTTGATACAACAACACAACAATGCTTTCACA
CACAAGTTCCCGCGTTGAAAACTTTTCTTTTTTCGTTCATTGGTATTGGTATTTGGTATATTCATCTC
CCTCTTAGCACCCCTTATTAGTATTATTACACTCTCTCTCCGCGTTAACAAAACTATCTCTGAACTT
TCCTAGTTATTACAATTTATTACACAACCCATCTCTCTTTCTGGTTTCGCTTAGATTTAGAAACACA
GACTTTTCCAAAGTGCTCGTTCGTTTACCTCAAACCTCAAAGAAAAAAAAAAAACATTGAGTTATC
ACTTGTTTTCTTTTTTGGGAAGACAAAAACATTGAGTTCAGCTTCTCACTTCC

>Glyma.15G053600

TATTTAATTATAAATTATTATTATTATGATTTTTAAAGTAATTATCCTAAAAATGGGTTGTGATCGA
ATAAAAATGAAAAATTATTTTATAATATAATTGTCTAACTTAATTTTTTTTAGATGAAATAGTTTTA
TATAAAAGATAATACTATGATTTAAAAAACGAAGGTCTACTGTTACTCTTCAAAATATCTTAAGC
TATATTTAGACAAAATAACTTAAAATCTTCCAAAAAAAATTTAAAAAATAATATAAATTTGTTGAT
GCTAAATTATTTAGGTCCTTTAGATCTTGGGCTAAATAATTATTAGGGTTAGAGTATTTTGAACCT
GGACATATTTTTAGATCTTGTGTTAGTTTTATGGGTCTTTAGGGTGATTGTGGGTCAAATTCAAAC
AATGACCCGATAAATACAATCCAAAAAAAAAGTTGGATTGGTTTGTATCATTCGTTTAATTCGAAT
CTTAAAACTATGAATTTGATAACTTTAATTTGAATATCAGATTTTAACGTGTAAAATTCAAACTAA
TCCAAACTTATACTATTTTAGAAGATTTTAAATTTCTTATGTTGCGTAATTTCATAGTAATGTAATT

TTTATGTTATATAATTATATTATTCTTACTTTTAATATTATTTATTACCTTTATTTTATTATGATTTTTT
TATAGTTTCTCTATTATTTTTCCATTTTGTTTTATTTATAATATTTTTTTATTAGGTGATCTGATGACC
CACTCAAAACAACTTACATGTGACTAGTTTGATTCAGTTTGGGTTATATTTTTTTATATAAAATAA
AAACAATCCAAACCAATTTGTTTGGTTTAAAATATCTCCTAAATCCAAATGAATCCAAACCATGA
TCATTCATAACGAATCGATCATTGGGATAAAAATGGTACACTCCACACCACACTACATTAATGAG
AGGAAGCTACTAATCAAATGATTATAGACCTAATAAATAATTTGTCTGTAAACCATATCAAACCA
TTCATTATTATTATTATTATTATTATTATTATTATTATTATTATTATTATTATTATCTCTATATTTAAT
TGTCTAAATAGTTTAATTATTTCTTTCAATCTACTTTTTATCTTTAAATCAAATTTCAATAACTTTTT
TAATAAAAATATCAATAATTAAAAATAACTTTATATCATAATTAAAATTATTTATTATAAATTTAA
AATATAATTTAGAGAGCTTAGTTGACTTAAAAATTTAAAATATTACGTAAAAAAAATCTAAAATC
TAAAATATATATTAAATATTTAATATTATTTATTTACTAAAAATTTCAATTATATAAAAAAATAGT
TATCTTAAATAAAATAAAATATTATTTAATAATATTTCACTTTAAAGACCTAACATTTATTCATGA
GTAATTAGTTCCTATTGAGAAAAATAAAGATAAAAACGTTAAAATAATCTGATCTAATTATTCAC
TCCTCTAATCACTTAATCGAATTAGATTCTCTTTATATCTTTTTCTTTTAATTTTTTTAATTATTGAAT
TTGAGTATATAAATGTTATTCCTCATAAATTTTGTATCATCCCCATTTAATTTTCTTTTCCAAAAGA
TTATCAAAATGAAATAAATATAGGAAATAAGGAAAAAGGAAATGAAAAGGATCCACTTCCCAA
AAGTCAAAGTCAAAATACATCTAACAATTTTTTAATTTTTTTTTCTTTTTTTTTTTACCTTTCTTCGC
ATAATTTATTTTACTAAACACTCTTTTTCTCCTTACTTTTTATTCTCTCCGTTAATAATTGTAAAACA
TTTATGCAGAAGTATTTTTACTCTTTTGCAGATAACAAAACGTTCATTCTTTTCAGAATATATATAA
GGAATATGTTCTCTATATTCGTTATCCTTTCAATATCTTTCGTCTTCTTATTATCTCAAATGGATTAA
AACATATAAATCATAAATAAAATCCGTTAAATAAGAATTGAGACAGCGTTGTATACTTTTCAGTA
ATTTTTAATTGATAATACTAGTAGTAAAAGTTACTCTGCTGTATAGTATAAATACTAAATATACAG
TATCAGTTTTTTCACTTTATCGCGCGTCAGTTGAGAAAAGGAAAAGAAAGGATAAAAAGAAAAG
AAAAAAAGTCTCTGTCGTCTCTCTCGTCCTTCGTCGTTCGCGTTGATCTCAGATTCAGTCTTCGCC

>Glyma.08G072100

AAATCAATTTAAAAAAATAATGATTAAAAATTATTACAAATAGTAAAATCATCAACATTAAACG
AAGAAGGATTAAGAGTAACTTTTAAAAAAAATTAAGACTAATAAAGACAAATTATTTTTTTAAA
GACTAGAAAATAATTATCTTTAAAGGATTAAAAATATTTAATTTTTTTATAATTTTATAATAAGAA
ACTAAAAAACTCTGCAGACTGCGGAATCAGTCCGGTATGTGGCGGCATAAATCGGGCACTTCTT
AATTATTTTTACTTTTCTTAAGAGAGAGAAAAAAAAAGGCCAAGAGATGCATGTGCATTCTTTGAAA
GTTGAAAACGGACGGTTCCGGGTATCCACCTACCCGATCGCCACGCGTCCAAGAATGATGGGCT
TCAAAAACATTTTGGGAATAAATAATTCAATTTTTTTTTCGAGACACAATAATAAATTAAAATTCT
CAACTTAGGAAACGATTTTTTTCATTATTTTCACCTTTCCTATTAGATACTCCCATTTCAACATTGT
TCCTTTTATTTATCTTCAAGATACATTTTATACATTGCTCTTACAATTAATTAGAAATTTTTTATTAA
ACATTTTAAATAAAACCCAATTTTACTCATAAACAAATAAAAACTGATTTTATCTTTATTTATATA
CTTCATTTTGTTAATTATATATATTTATTTTAATGTAAACTCTTTTTTTTATTTCAAATTAAGTTCTAA
TAGTTTTCTTAAAAAAGTATTAATTATTAAAAGTAATCTTACCATAATTTAAATTATTTATTATGA
ATTCAAAATATTATGAATTATAATTATATAAAAATATTTATTCTGTATAATACGCATGTTAAAATA
ATACTCCAAGTATTGATGAATGGTATTGTATCATTATTCTGCACCAATCAAAAGTCATTTTCTACG
GGTTTTATAGAAATTAAATTTATCTTATATAATGCCTTGTGTAGGCTAAATACTGCTTTAAAGTAT
CGATTTTTTTATTTATAAATATTAATTATTAATTTATTAATTTTTGTTAATAGTTAAGAAGACTTCA
AACCCACTTATTTCTTTCTTCTTCTTTCTCTTATCATCAAATCAACTCTATTAGAGGGTGGGTGGTG
CTGCTTTGTTTGTGCCTTTTTGCATGTATGTTCTTTTTTGTCTTTCTCCTTTTTATAAGGAGAGAAAG

ATGAGAGTGAGGTGATATGTGAGTTTAATTTAATCATACAAAATCGGCTTGTAAAGATTATTCTTT
ATTTATATAGTTTATCTTGACACTATTTTTATTTATCATGAGACTTGAACATGTCCCATCACCACAT
CTCTCCTCACGCTTATCAAGGCTACCTTCCTAGAGCGCGATAATCATAAGAATCTTTTCACAAAA
CTAACTTATCAATATTGCCGGTTAAGATAAAGATAAATTCATTCTAACCTATTTTATATATTGTAG
TACAAGATTGTTGGATAAAAATACAAATAATCTTATTAGTATTGTGTGATAAAGATAATTTTGTTT
TAAATTATTTTGTATATTATATTACAAGATTTTTTGTTAGATAAAAACACAATAATCTTATTAATAT
TACATGTAAGATAAAAATAGATCTATTATAACCTCTCATATATAATATTTAAATGAGTTAACTTG
AACTCATATTATATTCAATTTAATCAATTTTGAATTTACATAAATAATCTTATCAACATTTCATATT
AGACTATGATTCAAATAAATAATTTTTTAAGAATTTTATGGAGGATAAGAATACAAATAATCTTA
TCAATATTGCATGTGAAATAAAATCAAAGATAAATTCGTTCAAACATGTTATATGTGTTATACTA
CAAAATTTTATACCAGATAAGAATACAAGTAATCTTGTCAATAGTGCGTCATAACCTCTTATGAG
CCAGATAGAGACCAAGCTAAATTCTTTATAACCTCTTATATATATATAAAATAAAATATGATAAA
AAACGGAAGAAGCAAAAATATAGGTGGTAGCTGTCCCGCCTGTCCGGGTTATTCACTTCGTCAC
GTCACTGTGTGAAAAGAAGCTGACTGGAAAGAGCGAAAGAAAGTAAGAACGAACGAGCGAAA
GCAAAAAAGTGTGTGTTGATTGAGATACTGCTAACTCCTTCAACGTTTACGCGTGGATCTCTCTCT
CTCTCTCTCTGATTCTTCGGTCTTCGCC

>Glyma.05G117000

CGGGAAATGGCAATTGAGGGAAATAAAAAACAAAAAACAAATATGAAAGCGAGTTGTTTATTT
CAAAGTATAGATTGGATCCAACCAAACTGCACTTTTTAGTTATAACTAATAATTTTAAAAGTGCC
ACAAATTTAAAATTAAAATAAGAAATAAGAAAACATACCCAAAAATATCATATTGACATTTGAT
CAAACACATAACCTGCAGCCAATAAATGTCCTAATGAACATGATCAGAAAATGCATAACTGTGA
GCACTAAAAATACCACTGAGCCACATACACCATGATCCAAATAAAAATTGCAAAAATAAAAAC
CGCAAAAAGATACTGTGAGGTGTAAAGCTTGCATGCAAATCCAAATAATTAAGCACTGAGGAAA
ATACATAAAAATTATAGCTTACCTCGTAGATTATCCCGTAAAACTCTGAACAATATCAGAAATAT
GAGTTTAAGGAAAAAATAGCAACGAAGCATCCAATAAAACCACAAAAAATCATACAAAAAAA
ACATAAACACCGTTTTAATAAAATCAAAATAAAAGAACCTCTATCTTCCTCTTAATTTCTTCGACA
ACTTCTTTGTCAAAGCTGCTGTTGTTACTGTAGCCTGTTCCAAAAATAAAACCTGTTAATTTTTTGC
TTTTTCTTTTTCTGGGGTGCATTTTCTAAGCAAAACGTGAAGAAGTGAAAAAAACACAAAAGGAA
TAGAAAGAAGAGTGAAAGGAAACAACACGAGTTGGTGAGAATGTTCCAAGTGGTTCTGTGAGAT
CCTCGTGCAGAGACCAAAGCGAGCAGCAAATGAAGAAGAAGAAGAACAAAGAATGCATTGCG
AAAAGCGGGTGGCTTCAGCATTTCGGGGAAAGTTCAGTTTCCGAACAAAAAAGAGAAAGAGAT
AAACAGAGAAAAGAGAAAGATTTAAGATTTGAGGAATAGAGAGTAACGTGGAGAAGATTGAGG
AAGAGAAAAGGAAGATTGAGGAAGGGAGAGTAATGTGGAGAAGATTCAGGAAGAGAGAGGAA
AGTTTCGATTATTAAGTGAAATTAAATGGAGAGTTGAAATCCGCCAATCAGGGAGTGACATATGT
TCGAAACTGCACATGCAACACGACACGTGGCAGGAACAATAGAAAAAAAAGTGCAAAAAAAT
AAAGGGGCAAAATGACAAAAAAATTAAGGAAATGGATAGGTGTCACAATCTTAGGAGGGACAC
AATAGGCTTTTCGAGGGACTTCTCTTTTATAGATAGTATATAGATATTACAAGATTTAGGGTTGAA
AAGAACATAAATAACCTTGTCAATATTAGGTGTTAAATAAAAATAAATTTATTCTTACTTATTTTA
TATATTATAAGATTTTGTGTTGGATAAGAACACAACTAAATAAAGATAGTTCCATTATAATATGTT
ATGTATAGTATTTAAACCGAGTTAATTTGAACTTGTATTATATTGAATTGAATTAATTTTAAATTCA
CATAAATAATCTTATCAATATTTCATATTAGTTTTGGATTCAAATAAATAAACTTTTGAGGATTTT
GTATCATAAAAGAATATAAATAATCTTATCAATCCTGTGTGCATGCCAAAAGTAAATTCGTTCTA
ACATGTTATATTATAAGATTGTCAAATATAAATTTATTCTAACATGTTATATATGATATATTACGA

GATTTTGTATTTGTTAAGAATACAAATAACATTGTCAATATTACGTGTCAGTTTGAGACAAAATA
AATTTATTTTAACCTATTAACATGTATGTAGTAAATTATATTACAAAATTTGGTGTTAGATAAAAA
CATAAATAATATTGTCAATATTGCGTGTTGGACAGGGATAAAGATAAATCCGTTATAATCCTTAT
ATATATATATATATATATATATATATATATATATATATATATATATATATATATATATATATATATA
TATATATATATATTATTTGATGAATTTTGATAAAAAAAAACGGAAATATAGGTGGTGCTGTCCCG
CCTATTCGGGTTATTCCCTTGGTCACGTCACTGTGTGAAAAGAAGCTGACTGGAAAGAGCGAAAA
GCGAAGAAAGTAAGAACGAACGAAAGCAAAAAATTGTGTGCTGATTGAGATACTGATAACTCCT
TCAACGTTTCCGCGTGGATCTCTCTCTCTGATTCTTCAGTCTTCGCC

>Glyma.08G178900

TTTAATTTCTTTTGAGTGTATTTAGTCATTATATACTTACAAAAAGTATAAGTAAAAACAAATTAT
TATTAAAAATTTAATTTTTACAAAATGTAAAATAAATTTTAAATATTTAAATTTAATTAATGCAAC
TCAAGTCAATCTAACTCATAAACATCCCATATATATATATATATATATATATATATATATATATAA
TTTTTTTTTTAATCTCATAATTCTTTAGATTGTGTAATTTTATAATTCTTACTTTTAATATTATTTATT
AGTTTTCTTTTATTTTATTATGATTTTTACCAAAAGATTTAACTTAATTGATAAATGTTTTTTTATTG
CCACATATAAAAATAATATTGTGATTTTCTTTAATTTATATATATTATTTTTAATGTTTCTGTCCAA
ACCAACCCGAATATAATCAAGCTGGTTGGGTTCGAGTTATCGTTTTTTTTTTTTTGTTGATAAAAAC
AAATCAATCCCATCCAATTAATATTAATTATTAATTAGTTTAGATTAAAATGTCTTCCAAGCCCTG
AATTAACCTAACCCGTGATCATCCCAATGGATCCAATCCTGGAATGAAAATCGTGCACTCCACAC
CAGACTACGTTAATGAGAGGAAGCAACTAATAAAATGAATATAGACCTATTAAATAATTTATCA
CTCAATGTCAAACCATTTTTTTATTATCTATATATTTAATCGTCTAAAAAGTTTCATTATATTTTTTC
AATATGCTTTTTTTATTTTTCTTTAAATTAAATTTCAATAACTTTTTATAATATATATATATATATAT
ATATATATATATATATATATATATATATATTATTTAGACGTTCATTCTTATAAATTTTCCAGAATAT
AAAGTTATTGAAATAAAGTGAGGAGAAACAACGTTTTTTTAAAAATGGTGAACGCTTTGTTATTT
TCCAAAGAGTAAAAATACTTCTCCATAAATATTTTACAATTATTAACGGAGAGATATAAAAAGC
GAGCAGAAAAAGACTGTTTAGTAAAATAAATTATGCGAAGAAAAAGTAAAAGAAGAAAAAAA
ATATTAAAAAGATTGTTAGATGTAGTTTGACTTTGACTTTTGAGACCAATAGTTGAATGGAAAAT
GAACCTCTTCATTTTCTTTTTCTTTCATTTCCTCTGTTTCTTTCGTTTTATAAACTTTTTGGAATATAA
ATTAATTGAAGATGACACACATTTTATTTTATGAAGAGTAACATTTATTTTGTCAAAAGTTTAGTA
ATGAAAAAATGCAAGGAAAAAAATAGAGAGAATTTAAATCCGGTTAAGTGACGTTTTTTAATAG
ACAAATAAATAATCGGATCGGATTTTCTGATGACCTTGATCTGAAAATCCATCTGTTATTTTTGTT
ATTTGGGGTTACTTATTCATGAATAAATTTCATAGGTTTAAAGTGGAATATTAACTAATATATATT
TATTCAATAGAAATATTTATTTTTTATAATTAAATTTATAATAAAGAAATAATTTTAAACATAATT
AAATTATAATAAATGTTATTATAATATTGAATATATAATTTATATTTTAGTTTTTTACATATTATAA
ATATCTTTAAATAAATTTTCTCTATGACACAGTTGTCTCATTAACACCCAAAAAAGTATCTTAAAA
AAAAGTAAATTTACACGAGTTAGGAAAAAAAAAATATAAATGATAAAAAATTTATGAATATTTGAT
GCAATTAGATAGTTAAGATTAAATTTAAGATAAAAAAAATTTAACTTAATTAGTTGAACATCATAT
CATACATAAATTATTATAAATTTAATATTATTTTTTTATTTTTACAGGTAAAAAAATATATCTAAAA
TCATTAATTAAGTTAAAATAATCTTTAAACTTTTTTTTATATGTTGGATTAAGCTAGTTAAGCTCTC
TAATTATATTTTAAATTTATAATAAATAATTTAAATAATGATATAAAGTTATAATAACAAAAGCTT
CAACATTTAAACAAAAAGAAAAAAGTTCATTCTATCAGTTTTTCACTTTATCGCGTCAGTTGAGA
CTTGAGAAACAGTCCAGGAGAGAAAAAAAAAGGATAAAAAGAAAAGTCTCTGTCGTCCTTCGTC
GTTCGCGTTGATCTCAGATTCAGTCTTCGCC

>Glyma.17G038800

ACTATTAAGATTTGGTTGGATTATTTAAATAAAGTGAATTCAATATAACTTAATTAAGTATGATTA
AACTGGATTGAAAGTCAAACTAAATTCAACCTAACATGTCTCTCACTTACTTTCAAATGGACTTC
CAAATTGTACTGATATTGGAAAAATAACAATAAGGCAATTGCTTCGATCCCCCTTTTTTTGCTTCC
TGTACCAACAAAAAGACTCAAACGGACAACAATGCCCCTTCATCCAAACACTCAACACCACCGT
GCCTCAGCTCCATGTAACCACCCAACACGACCCCTTCTAACAAACACCACCACCCAACACCGTT
GTCAACCACTATGATATAGGGGAAGAAGACACACACAACATCAACACACAAGAAGTGTAAAAT
GCCATAATTATGGAAACTCCTTTTTGAAATAGTTTAAAAAAATTGAACATGCCAAAAAGGATTTT
CCAAAATGTTGTCACGTTTTAAACTTCCTTGACTCCATGCATGGTTCGTGGGTGGCGACATGATGT
TGGATGCAAGGGGTGGTTGGGTGGTGGATCTGGTAATGTTTTGGTTAGGTGGTGGTGGCGGCTAT
GGAAGATGTTGGCTGAGAGGAGGAAAGGACATATTTGTCCATTCATGTTTTCTATGAGGTGCAGG
AAGAAATTGTCTAACAATAACACTAGATTATAGTAGCTGAAAGACAATTGCTTTAGATACCCAAT
ATTTTTGTTGGGATACCCATCAACACAATGAAAGGACTAAAATGTCCTTCCCCTTTTTACGGATCG
GCAATCCGTATGACTCATACAGATTGACGATCCATATGACTCATAAGGATTGACGATCCGTATGA
GTTTTTTCTTTTTTACGGATTGTCAATCCGTAGGTCCACACGGATTGTCAATCTATATTGGGTCTAC
GGATTGTCAATCCGTACTTGGTCCATAGGATTTTTTAATTTTTTTTTAAATATAATTTATGATTTAAT
TTAAAATTAATGGTCCAAAGAGGAATCAAATTTATGTCTCTCACATTATTAATAAAAACACTCAA
ACCAACTGAACTAATAGACACATTATGTTATAAACAAATAGTGTCGCTATATATAACACTAAAAT
TTCTAATTTATATTTAACGTATATGTAAATTTACATAATAAATTTTGAAATGATTATTTTTTATCTA
ATAATTAATTTGTTTATATATAAAAATTATAAATAAAAATCATAGGTTTAATAAATATTTACATAT
GTAAAAAAATATCAAATTTATTTAATTATCAATTGTTATAAAAAACATATAAATACATCATTCAA
TTAAATATCATATTTATTTAATTTTTAATCATTATAATAAACACCTAAATACATCATTCAGTTAAAT
ATCAAATTTATTTAATTATCAAGTTTTGTAAATAAATTAAATGAATAAAACAAAAAAAATTTTAA
AAAAATAAAATTTACTTTTTTAATCATTATAGTTATTCGGATCAACAATATCTACGTGTTTTAAAA
AAAATTCATACGAATTGTTATCCGTATGAGACGCTTTCAACCAAATGGGCTTGCAAATTGTCCTTT
TGGGCTTGGTAGCTGGTGATTTAGTAGATGAATGATGGAGATTAAAATATGATTAGTGGCCATAA
GACTAAAATTAAAAACGTCATACATTTTTCTAATCTGTTTTCGTTGATAACTTTTATATGCCATCTT
CGGTTGTTTCATGATTAAAAATTCATATTTCTTTGTGAATTTTTCTATGCAAATTGAATACCATTTG
CCAATTTGGACCTGGATCGATATATCTTTTTTTTTATGAAACTAACATTTTATTATAACTTTTTTTTT
TGTTAGCGTATGCCTAAAAATGGGTATTTGGGTCGTAATTCACGGACCAATTCATATTAAGTTATA
TGAGTTTACGTTAAACATTGAGGAATCTAGTTAAAAAGGTTGCGTCAGTGTGTGATAGATACTAC
CTAGGACTAGGAGGCCACAACACAACGAAGAACCCGCGTACTATCGTGGACATGCTTTGGCTTA
CATCGTAAGAAACAAGTGCCACGTCGGACATGTGTGAACGCTACGTGTTGGGCTTTGACTTCAAC
TCTCTCTTTTCTTTAAGTACCTTTCACGCGCCACCGTTGCTCTCCGTCCCTGTCTTGTCCCTCTCTTT
TCTTTTCATTATTATTCCTTCATTTCTATTTCCTTCAACACTCTTTCATTTCCCTCTTACTCTGTTTTG
GCCTTTTCCTTCATCGCTTTCATCCAATTCAATTCAATTCTCTGCACTGGCTTTTTGAAATTTCCATG
TGTGAGCGAGAGATTATTGCTATGTTGTAATC

>Glyma.15G143400

GAATATATAAAATATAAAAAATAAAATAAAGAAAATAAATTGATATGAAATTTATTATTCTATC
CTTTTACTTTTTAGGTTTAATTATTATTTTTGTTATTTAATTTGTTTTTTAGTTCAATTTAATTATTTAA
TTTATTTATTTGATTCAATTTGGTTCACGACCTTAAGTAATACTAATAAATTATGTTTTTGTCTCGAT
AAATTAATAAATTTTGTTTTTATTTTTAATAAAATTATTTTTGCGTTGAGTTTCAAATAAAAAATAT

ATATGTTATTTTGTTCAATTTTTTCATTTTTTCTCTATTGTATATAGATCATTTGTTATTATTTCTTATT
AAATATTTTTCAAAAGAACTAATATAAAATAAACAAAATTTTATAAAAAAATTAAAATAAAAAA
ATAGATATATATTAGAAAGACTAAAATAAAATAAACAAAATTAAAAATAAAAATTTAATTTTAT
TAGAAATTTAATAAAAAAAAAATTATGAAAGAATCTAAATAAGATTTGTTAATTTATTAGAGATTA
AAAATATATTTAAACTTTTTAAATTAAATTAAAATTATTAATTGTCCCCACTGATTAAAAACTTCT
ACATCCGTCGGTGCATTTAAAGCTAGTTAAGAACTTATGAGTATATTAATATTGAAACTCGTGCTT
AAAAATTAAAATACTATTAATAAATTCAAATGTGAGTGATAGAAGTTCCTATATAGTATTAGTTC
TGAATGCCGAGTTTGGATTCTTTGGAATGAAGGAAGGAAAGCAAATAAGAGGACGATGGAGGG
ACCGAAAAAAGTACTGATAATTTTCCTTCAATTATTGAACGACAGCTTTATGGGAAAGGATAAGA
AAAAAATGTAGAATCAGAATTTTTTGTAATCTGTCCACTTTTAAACGGAGTGGGAAAATACCTTA
ATTTTAGCATTTTTTTTACTAAAGATATTTATAATATATATTTTGTATTATTTTTTATAAAAGACAAT
AAAAATAAAATAAAAAGTGATTAATAATTTATTTACAAAATTAAATATTAAAGTAATTATTTATT
TTAAAGTGCATCAAGAATATCTCTTATCAGTAATCAGAGTTTAATTCTTTCCAAAGCGTAAAAGTT
ATCAACCCATGACAAATATTTAAAGAACTCGGTTATTAACCAATAGGAATGGAATTAATACATAT
TCTATCATAAATTATAGAATATGGTATTTAGACCACAATCTGTCAAAAAAAAAAAGAAAAAAGA
GACAAAAACCAAAATACTTGCGCGTGTGCTTGTTATGCAATTTCCCAACAAATTTGGGGAAATAG
ATTTGTTTGTTGAATACTACATAGTTTACATACAAATTGGATTGGAGTATATATATCAAAAAATGC
ATTTGCATGCGACATAATGATTTGATGACGATAACATGGCACCGTTTTTCTTGGCTTTGCCGATTT
GTACTGTAATAATCTTCGACCCCTCATTGAATATTTAGTAGTCATTTGTTATTTTCTAGAATATTTT
ACATTAATTTCAATTTAAAAATAAAAATATTAAAAAAGATAAATTAAAATAAAGGAAAAGTGAG
GAGTTGTATTAAAAAAACAACTGTCATTATCGTATGAATATAAAATTTTCATTCTTTTCAAGGAAA
TAAAAAATGTGTAATATAAATTCTTAAAATGAAATAATACTTAAGAAATCTGTCAAAGAATTTAA
CTTAATTAGTTAAATAAATACATAAGTTATTATAAATTTAATATTATTTTGTATTCCAATGAATAA
GAAAAATGAGATAATAGGAGTGCTACGGTTAAGTAACACTGTAATTTGATGATGTTCCCTCTAAA
ATTGGATGGAATAATGGATTATTTATATTTATATTTTTTTTCTTTCAACTTTTTCCGCCCATAAAGA
AAAAAACCCTTTTTCTCTTTTACTTTTTTTTTCTTTCTAAAATCAATCATATATGAGAGAAGCCAAT
GAAAGAAATTTAGAAGGCGAGAATATTTTTATTAAAAAAAAGAAAATAATTCGAAAATAGTAA
GTGAAGAAAGAAAAAGCAACAGAGGAGACCTGAGTGACATTATTCAGTTCCGACTTACCGTTTC
TCTTTCTTCCAATCTCCGGAATCAGGTAAAATTTCTACCTTTTTCTCTCTTCGCATTTGACTTTTGAC
TTGTTTTTTTTCTTTTTTTGTTCTCTCTTCAGTTCACAACTAGTTATCTGATTCTGTATATTATCTGAC
TTCTTGCCAAGGTTGTCGGTGCTGCAATTGTTAGTTTTGTGCTAAGAGAAGAAGCTGTTGGGAAA
GTTTCTTTTGTTGTG

>Glyma.09G038300

AAGTGACCTACAACGGTTTGATGAATAACTAAGCACTCCTTTATTTGTTAAAATTTAAAATCATAT
CCATATCTAAGATGTACTTAATGTTAATTTCAGGATGTGTCAAATACCATGTACTCATTTATGAAA
AGACGGACTCCTCCATCTAAATTTGGAAGGGATTATAGAATACAAGATTATTTATAATCATTTGA
GAAAGTCTACAAAAATTGACTATGGATAGAGGACTTTTGCACAATCACAAAATTTGAAAGTTGG
AACTCAAACAAATGCAACATTTAACTTTATTTTATTTTGGACTTGTTTGCAATTTAAGTACATTATA
CAATACTAATATCTGTAAATTTTTGTTTATAACTCTTGGTAATATATTTTGTGTAAAATGATGCTTG
GAACATCATGAAAACATTTGTTGAGAATTTTTATTTATAACTCTTGGTGGTATATTTTGTGTAATTT
TTTGTTTATAACTCTTGGTGTTGGTAGTATATTTTGTTTATAACTTTGGTTTATTTTTAGAGTTTAAA
AAAAGTTCAAAAACCAACTTCAATTTTTAGAAAAATCGTGATTTAAAAAAAATCAATGTTGTTTT
ATCGATTTTTTTAAAAATCAATATTGTTGTTTACTCACCATATCAATTTTTAAAAACCGATGTTTGT

GAGATGAAAACAACATCAACTTTTGTAAAAATTGATATTATTGTTTGGTAAAACAATATTGATTTT
TTAAAAAATGGATGTTAACATTGATACTTTTAATGTTAATGTTAGTAATTTCAATATTAATTAATA
ATCGATATTAAAAGTTCTTAATAGCCAATATTAAAAATCCATTTTCAAATATGAAATAACAATCT
CATAATTACTATGTGTGTAAAGTATAAAGAGACTAATATTCCATCGAGCAAATTGAAGGGATAA
AATAATAAATTATAATAATATGGTATTTAGATCACAATATGTAAACAAAAAAAAAACACAAAAT
TTGCGCGTGTGCTTGTTATGCAATTTCCCGACAAATTGGGGTAAATAGATTTGTTTGACTTTGTTGT
ATACGAGTACATACTTACATACAAATTGGAATTTATATATATAAAAAAAAAAAGCATTTTGCATG
GACATAATGATTCGATGACGATAACATGGCAGCGTTTCTCTTGGCTTTGCCGATTTGTACTGTAAC
AATGTTCAACCCTCATTCGTCCCCCGTGCAAAGTGCAAACCTTTTTCCTACACAGTGAAGAAAAC
TTTGGGTCCGTTATTCGGAGTTAAAAAAGAGTGGAAAAAAATTTAAAATGAACAGTAAAATTAA
ATTAAATTTTAATTTAGAATTTTATTTTAGTGCACTCTTATTTTATTTCATTCCACTTTATTGAACAA
ATTCTTTGGAAGTGTTTATTTTCGTGAGCTCACTCCTTAAATATTAGGTGAATTTGTTCAATCTTTA
AAAAAATTAAATAACTGTTTTTTTAAAAATAAATATGATTTATTTCTTAAAATAAATAAAAAAATG
ACTTATTTTAAATAAAAACAGTTTATCAAACACATAAAAAAATATAAAACTGTTTATTTTACTAAG
AAAAATACTTTTTAAAAAATAAGTTTAAATAAACTCATCCAATTTATTTTCATCAAAAGATATTTA
GTAGTCATTTGTTATTTTCTAAAATATTTTACATGAATTTCAATCTAAAAGTAAAACTATTAAAAA
AGATAAAATAAAATAAAAAAAGTAAGGAGATGCATTAAAAAATAATTCTCATTATCATATGAAT
ATAAAAATTTCACCCTTTTATAAGGAAATACTAAAGGAGAAAGAAATACGTGTAATATAAATTC
TTAAAATTAAATAACAACTTAGGAGACCAATGGAAGATGTGAGATGTTAGTAATGTTACAGTTA
AGTCTCACTGTAACTTGATGCTCCCTCTAAAATGGATGGATTATTTTATATTTACTTTTCAACTTTT
TTCTCTTTACTTTCCTTTTTTCTTTCTTTCTAAAATCCATCATATCTGAGAGAAGCCAATGGCAGAA
CTTTAGAAACCGGGAATATTTTTCATTAAAAAAAAGAAAATAATTAGAAAATAGAAAGTGAAGA
AAGAAAAAGGAACAGAGTAGCAGAGTGTCATTTTGCCGTTTCTCTTTCTTCCGATTCCAACAGAC
CAATCAGGTAAAATTCCTACCTTTTTTTCTCTCCGCGTATTTGATTGTTGACTTTTTTTTTTTTTTTG
TTCTCTCTTCACTTCACAACTTGTTTTCTGATTCTATATATTATGCTCTTCACTTATTGTTCTCTTTTG
TTTGATCATGTCTTTGCTGACTTCTTGTTAAGGTTGTCGGTGATGCAATTGTTGTTGGTTTTGTGCTA
AGAGAAGAAGCTGTTGGGAAGGTTTCTTTCGTCTTTTG

>Glyma.05G148300

ACATAAAATTAATTTAGTTAGTTATTAGAAGTTATTTAAATAAGTTAGTTGATTACTCAAGTTATA
GTTACTAAAAATTATTTTAAGTAAGTTAATGTTGATTACTCAAATTAATTATTTTCTATCTTTGTAT
AAATACATCAATTTTGTAATAATTTAATTGAATGAATGAATCCTAAAACTACTTTTCATCTATCTT
GTATTTCTTTCATTCCTAATATATATATATATATGTTAGAAGGTAAAATTATAGTTAAAATTAATTT
AAGTAAACATTTTAATTACACTTAACGGTTATAAATAAATTTATTTAAATTTAAAACAATCAGGT
CTTAATCAGATATAATACTCAGGACTCAAGTTGTTAAATGATAGTTAAATACTATCTTCATAATG
AATATTTATTTATTTCTATCAAATTTATAATAGCAAATAAAATACTACCATGCTCTGAAAAATAAA
ATCCCATATGTGCATATGGAAAACGTTTGGAATAATTTATTCCCACGATGGAAAGAATTTAAAAC
AATGGGCAGTATGTATGACTCTATCTTCCCCAAAGATTATCCAGTTTTCTTATCCAAGTTCAGCAA
TTAACATGGCTTTGAATCAAACACGTGTACTCCATACACACTTTCTCTCTCGTTCGTGTCATCATG
GACCTGAAATTCACCTCTTGTTCCCCAAGTACTTCGTGAGGAAATGACATTGATTAAATGCCACA
TGCTTCTTGAACCATACCCTCTCAAATTAAACCTCAAATTAATTTAATTCACACACTTGTGTTGCA
ATTTGTTTCAGTTTTTCAAAATAACTGAAAAGAATTTAAAATATGATTAAAATGAGAAGTTATTTT
AAATGTTTTCTCATAAAATCTGCTAGCATTTCTCAAACACATGTACTATATATAACAAATAAAAT
GAGATTAATGAATAACATACTTTGAAATTGTTTGTTTTGAAATGTGGATGAAGTCAATGTCTTTTA

TTATTATTATTATTATTGAAAGTCAATATATCAACTCAAACGCCTATTACTACTAATAAGTTTTCA
CCCACCTGAAATTAGTCCTGTACTTCAACCCTCGTTCACACAGTACAAACTTGTCTTTTGTGTTTA
ATAATGATGATCAGATCTTCCTTTATGATGATATCATTGCTAATCAGGTTGCCCAAGAGATATTCA
ACCCACTCACCTCATTCACAATTTCACATTTTGTTTTTTTATTAATAATTAATTTGACCTAAATACA
TGTAGCGGCATTTTCTAAATTTATAATGAAAGTTTAATTTCAATATATTATGCACGGTAAATATTTT
TATATTAATTAACTTTTTAATTATGGCATGCTATATTATTAAAATATGCATGATGAAACTTTTGATT
TATGGATCATCCACTCAATAATGAATGTGTTCGCCTTGCATATCTCCCTTTTTTGATGAAGTCCTTG
CATGTCGCTTAAAAACACCTTCGCTCATTTATACATTAAATATTTTATTAAGATAAAAAATGAAA
ATATTTAAAATAAACACAATTATAATCGAAGAGTAGAACTATTTATAATATTTTTTTAATCTCTAT
AGTCTAGTCAGACTTTTAATCGCGTACGTCTTTTGATATTTCTTTAAATATTTAAGAGGGTGGTTAG
CAAAGTTAACAAAAAAGTAAATTTTGTGTATAATACTACTAAATGTTTATTTAATTATAAAAGGA
CGACTACCTGATTGTGATTACCGCTTAGAAAAATCACACTTGGTCCAACTTGGAAAGTCTTTTTTT
AATCGCTGTATTGTAAAGTCTTTCTCATATAGATCTTGAATATTCTAAAATTAAATTAAAGAGGTG
AGAAAAGCAAGACATAGTAGTAGCAAATAGTAACATCTCTTGGTCTTGGTCGGGGTCTCCAACTT
GTTTAATAAAATTGTATTGTATCCAAAAAAAAAAAAAAAAGTTGTTTAATAAAAGCACGTTTCGC
TAACCTTAACCTTTATCACAACCTCAAATCAACTCTAAGCTAAGCTTCAACTAACGCCAACTGCG
TCTGTTTCAGCTTTCTGCTTTTGCCACTTCCTTTCTCAGATCCATTATTCTTCGGAACCAACTCGCA
CCGCAGCGAATCTAGCAGCTTCTGCGGTTCCAACAATGCTCTGTGTCCGGTTCTACTACTTGTAGC
TTTTTATTCCTCGGATTCAACACC

>Glyma.18G005100
TATTAGTGAGATCAATTTTAGTGCCACGTGGACTATCCAACGTGACACTAAAGGATGATGTGGCA
TGGCACGTGGATGTGCCTCTTAAAAGATGTCACGTCATTTGATGATAACAAAACGAGTAAATAA
ATAATTTAGTCTCTGACTTTGTATCCTTGTTGCATATTAGTCCCTAACTTAATGAAAAATTCAAAAT
AGCCCTTATCTTTGCATAAGTGTTGCAAAATAGTCTTTCTGTTAAATTTTAAAGTAACGTTGTTAGT
GAGGTCAATTTTAGTGTCACGTCATTTGATGATGATAGAATAAGTGGCTTCTTCAAATCTGATGGT
TCTGAACCAATTGAGGCATATATAAGAAAAAGAACCCACATACACTTACACAAATAAAAAGTAT
CAAAAATCCACAACAATAACCTTATCACTGCAGCCGTCAACACCAATGGACGAGGTCTGCATAA
CCATTTTCTTTTCTTTTTTTTTCTTCAATTACCATCAATGTATCATTCCAGGTTCTGATTTTTTTTTGT
GTTCTGAATAGGAAGAGAAAAAATCAGAGGAAAACAAAGTGGAGGAGAAAAAAGCAGAGGAA
AAAGAAAAGAAAGAAGAAGAGAAAAAATCAGAGGAATAAAAAGATGACAAGGAATCCAAAA
AGGAATCTGCGTCGCTAGAAATTGTGCAAGGCATAACCTCCGCAATGCATGAACATTTTAAGCAT
GATTTATGACATCTTTTAAATTAGGGACCATTTTACAATACTTATGTAAAAATAGAGATTATTTTG
AATTTTTCATTAAGTTAGAGACTAATATGCAATATGTGTACAAAGTTAGAGACTAAATTGCATAT
TTACTTGTTTTGTTATCATCAAATGCGACATCTTTTAAAAGACACGTCCACGTGTCATGTCACGTC
ATCCTTTAGTGTCACGTTGGATAGTCCACATGGCACTAAAATTGACCTCACAAATAACGTTAAAA
TTTAACAGAAGAACTATCGTGCAACATTTATGCAAAGATATGAACTATTTTGAATTTAACATTAA
GTTAGAGACTAATATACAAAATTGCTACAATCTCTCAAACTCAATTGTCTATTCATTCATTTTATTT
TGATTGAAAGTCGTGTACTGTATGTTGACATGTTGACATGTTGACACAGACAGGGAAATCAAAGG
CAAGTGGAGTTCAAAACGAATGATTAAACGTCATCCACTAACTATGTCTATAGGGAAGAAATTC
AAAAAGCAAAGAATGGTACATGTTAACTAATTGTCATATTTTCACACGATGAGGGTGGAGCCTTG
GATTTTGACTACTACGGTGGAAGATGAAGTCAAGGTAAGCTCAAGAAGCAAAGTGTGACACTTA
CGACTTATTCCGAGCGAGGTCATTCTCAACGAGCTCATGCTATTATTCTTATTACTGATTCACGTT
ACTCACGGGTTCTCTTCATATGTGGTATGAATCTTCATTTGTTCACACTGCAAGTGAAGTGCTACA
CAACAACACTTAACTCTCCGTCCTGGTGAACTCGAACGAAATTTCCTCCGACAGTTTCAAAATTC

AAAACATTCATACTTAAGTTAAAAATGTTTTTAACAAATATTCATAAAATTTTCATAAATAATAA
CTAGAATACATTGACATTATAAATATTCATTATACTACCATCTAATCCGCCTCGAGCAAATAAGA
CTACTAGAAAGTAGAAACAATTAGACTCTTTCTGGTTATTTAAGGTAATTACAAACTATTACTTTT
ATTTTTCAAATTTTTAGTATAATTTTGAGAGTCAGAATTAATTTATCCAAACATATTATAAAACAT
TCGATGTAAATGTAAGGTAGTGATTGATGAAAGAATTTTTCTTTTTTATTTTTAATCCTTTGTTTCAT
CATGAAAAAATGATCTAATTCATTTGAAGTTCGATTGCACAATAAGGAAAGTCTAGACTAAAAC
TATTTCCGTCAAAAGATCATGTTTGAAAATTCAATCTTTAACTTTAACGAATTAAAATCACTTGGT
TACTAAGAAAAAGAATTAGTAATGTGTGTTTAGAATTAGACAAAATAAAAGTGTAGGTAAGACA
GAAGGAAAAAAGTAAACAAGAAACATTCTGGTTCTCTTAGGTGATTGCAAATGCTCAGAATTAA
TTGGGGGTATAAAATAATGACACCAAACCTTTTTCAACCAAGTTATCTCTGTCTGTCAGTGGGCC
AGTTCAAGTCTGAGAAAAGGCCGGTGCTTGTGTTGTGTGTTGTGGCTTGTTTAACTCTACAATAAT
ATATCATCATCAACTTTTTCATTCCTTAGGTTTGTGTTTGTGGAGAAGCTAGTGCTATTTCTCCAAT
TTTAGTTCCCCTATCCTCTCCGCGATTAATTACT

>Glyma.17G031900

TATTAAAAAAAAGAAGCCTACTTGATTAGATAGATAAGACATATCATATTCATATTTCAAAAGA
GGCCATGATATCAATTTGATATTTTCATATCCCGTTATTTTTATTAAATCAACTCATAAAACAAAT
ATGTGCAGAAATCATGTTATGCTTTACAGGAGTGACAAAATCGGATCACTTCATTGGGCCAATCT
TTTTACTTTTGTTTTTTTTTTTTTAGTTTTGAGTTCTTTAATTTATCGACCCATTTCTCCTCATCCCACT
TAATTCGCTAAAAAATAATTGAGAACGACATAAGGTCGGTTAATTTGTCAACTATTTTTTTCAGA
AAATATTTAGTATGTGGAAATTTTTTAAGTATTTCTCTCAAATTTTATTTATTATAACTTATAAATTT
ATATATGAAGTTCTTTAATTTTGATATATAACTATTATCCATCAATTGGAAATTATTTTAAGTTCCT
TTTTTAAGTTTCTTTTATTAAAAAAATTGAGTATTTATTTTAATAATATATATATATATATATATGT
ATATATAATTTACAAATCATCACTAGAATAATTTAATATTAAAATATTTTTTGAGGTTGGTAAGAA
AAATAGAAATTCTTGTTTGAGAATAAACCTAGCACCTTATTTTTATATAACCAACAAAAAATTTA
CAACTAAAAAAAGATTGTGACTTTGAATACTTTCACTAACAAAAACTAAAAAATTAACACTAAT
CTTAAAAAAAAAAAACTAAAAAAAAGTCAACTTATCAAATAAGATAAAAAAATAATATAAATT
TAAGATAAAAGAAAATTTATTATTCTTCACTTCACTACGTATGAAAAATAAAATTCATGATTATTT
CCTCATTACCATATATGTTACCGTAAAATGTGAGTTAAAAGCATAAATATAAGATGCTATTACAA
TATTTTTTTAGATATTTTTATTAAATTTCTTAGTTTCTAAGAAGAGAGAAATATAATAAAAATATA
ATTATTATTATTTAACATAATCCAATAAGAAACTAAATAATTTTTTTAGAATTTCTCAAATAGCAA
AATAATAAAATAAAATTGGAAAATGTGTAACTAACTTAAGTTGATTTTTTTACTTGTTTATTTTTTT
GAGTAAAGCTTATAAGCTTTGTTTAACATGACATTTCAGTGAAAAGAAAATATTTGTTTTTCTTTTT
CTAGCTTGGTATTAACATAAAAATGAGAAGGACATATTAAAGATATCATTTCATTACTTAAATGG
TTTAACAAAAAATAATATATATATATATATATATATATATATATATATATATATATATATATATTA
TGATTCTATAATTATTAATAAAAAATATTTCAAAACTAAAAATTAGTCTTATACTTTTTCTAATATT
TTTAATTTGTTAATGTGTTAAATGTTTTTTTTATCTTTTTTGTTTTTTGTTTTTTTTTATTTTTTCCTGAT
AATATGAATTTTAATTTTCTTAATTATATTAACTAATAACTAGTATGATAAAATATTTTTATTTAAT
TAAAATTAATTTATTAGAAAAGTTGAAATATATTTGAAATTTGATTAGAATTTAATTCTTATATTA
TAATAATTTTATATTATCATTTAATTATAAAATATTATTTATATGACTTTTAAAATAATTATTATAA
AAGTTAATTAATTTATTTATTTTTATTAAAACCCACTAATACCAACCTAATGGTAAAGTTGGCATA
ATGCGCGTGGTTGTATCACTTGTATGTTTATTTTCCGTAACAGCCACGGTAAAATAAAAAAGCCA
CTGTAAAATAAACTATTTTTCCTTGGATAAGTATTTTTAGTCTTGAAAACATTTTGGTTAACTCTTG
TCTTCTTTTTTCGTCTTGAAAACAACCTTTTGTCTTCATCTATTTATTTATTTTATTCGAATTAGTATT

TTAATCGTACTTAGCTTCTTCTTTTTTTACACAAAAGCTCTTATTTTATTTATTTTATAACTTATACT
GTAACTAGTAATAATTTAACGCATAAGAGTCAGACTGTAGAATAGAAAACAGAAAGGAACAGG
AAAGAGTGTCATTCAGCTCCAACGAGGCGATCATATTTCTCTGCAATCCGAACAGACCCACCCTT
CTCTGCAATTAGGTAAATTTACCACCTTTTCTATTTTTTCTCACTACTCTTCTAATTTTTCACCCTGT
TTTTTATTTTATTTTTAGTTGAGAACGATGTTTTTTTCTTTTATCGAGTCACCACTTTGCTCTTTTTTT
CATTTTTCATGTAATATGCAGTTGCTGAGTTTTAGGTGAGAGAAAAGTGTTCGGAATTTCCCAAGG
TTTCTGTGAAT

>Glyma.07G242000

GAAAAGAGATTAAAAATATTTTTTTATAAAATACAATAACTGAATTGGATGGTATTAAATTTGGA
GTACTAAAATTAAGATTTTATGGAAATATAAGAACTGAAAACATATTTTTTTCCTTCTTTTAATCA
TCACTCGCTTCCTTAAAATCATTTTTATTTAAGTACTCTTTTGAGTTAATTTTTATCCTCCAAAGCTC
AGGCACATATCCTTACCTATAATGTCAAAGTGTTTTGCAAGAAACAACAATAGATATATACTTAG
ATAGTACATGTACACATTTTCTTTCGGTGGCCACGAGAAGCCGCCAAATGAATTTAGACTAGTGA
GCACTACAAATTTGACCCAAGTGTTTTTTTTTATCCTTAAAAAACATTATAATAATTAAACTAGAT
ATTATTTATAATTTTCATTTCTTTTAATTTTAAAAATATTTTAGAGAAGATAAAAAAATATGGAATA
AGGACAGATTGAAGAAACTTGGACTGGAAAGAAAGAAGAAAGTAAGACAAAAAGAGAAAGGA
TAAAGAAGAAGAAAAGACACCCATAGTATCATTGATTCATCAAAATAAGGTATTTAAGTTTATTT
TTAACCTTTTTTTACTAGTTAAAAGATTTGATAAATAATTTTTTTAATAATTTCTAATATTTTTTGGA
GTATTGTTTTTAAAAACATTTGCTTTTATCTTTTAATTTTTAATATTTTATTTTATTTTTATCTTCAATT
TATTTATTTTATTTTCGTATTATTTTTTAAAAATAAATTATAATTTTATTATTTTTTTTTGTCATTTTAT
ACTTTTGAATTATTTCAACGATTAATTTTATCAAACACTTATAATTTAATAAATTAATTTTTTAGTT
TTAAAGATTAGTCAATCATAGCATAAGCAAAAAAGGATAAAAAGAAGAAAACAAAATAATAAA
CATGAGATATTTTTTTAAGTTATTTGGTAAGAAAAATAAAAATTATTGTTTGAGCATAAACCTAAC
ACCTTATTTTTACATATACTCAACGAAAATTTTACAACTGAAAAAAATACAACTTATCAATTAAG
CTAAAAATAAAAGTAACCTAAAATTTAAGATAAAAAAGAAATTTACTATTCTTAACTTCATCTAC
GTATGAAAAACAAAATTCACGATTATTTCACTCATCTATGATTTATGTTACTGTAAAATATGAGTT
GAACATAAATATGATGCTATTAATATATTTTCTTAGATATGTTTATTAAATTTCTTAGATTTTGAAA
ATAGAGAAATATAATCAAAATATCATTATTCTTATTTAACATAATCTCATAAGATACTAAATTAA
AATATTAAAATTTTGTAGTACTTTCAAACAGAAAAATAATAAAATAAAATTGGAAAATATGTAAT
TAACTTTAATTGATTTTTTTACTTATTTTTTTAATAAAGTTTATAAGCTTTGTTTAACATGACATTTT
AGTGAAAATATTTTTTTCTTTCTTTTTTCTAGCTTGATATTAATATGAAAATGAAAAGGAGATATTG
AAGATATCATTTCATTACTTTTAATTTTTTAAAATAAAATATAATATATATTATGATTTTATAAATA
TTAATATAAATAAAAATAAAAAATATTTCAAAACTAAAAATTAATCTTATACATTTTAAAATATT
TTTATTTATGACTGGATGGGCGTGGTAAAGTTAATCTTATATATGGATTAAATAACATTTAATGTA
TTTTGTTAAATATTTTGAGGATTTTATCATAATGAATATTTTTTGTATTAAAACTCACTAATATCAA
CCTAGTGGTAATGTTGGGATAAATGCGCGTGGTTGTATGGTTATTTTTCATTACAGCCATTGTAAA
AGAAAATAATTTTCCTTGGATAAGTAGTTTTACTCTCGTAAAACAACCTTTTGTCTTCATCTATTAA
TTTATTTTATTCGTAGTATTTAATCGTACTTAGTTCTTATTTTATTTATTTATAACTTATAATTTTATA
ATACTATAATAGTGTAAGAGTCAGACTGTAGAATAGAAAACAGAAAGGAACAAGAAAGAGTGT
CATTCAGCTCCAACGAGGCGATCATATTTCTATCGGTTCCAATACGAACAGACCCACCCTTCTCT
GCAATTAGGTAAACTTACCACCTTTTTCTATTTTTTCTCACTACCCTTTTAGTTTCTCATCCTGTTTC
TGATTTCTTTTAGTTGAGAACGATGATTGTTTTCTTTTATTGAGTCGTCACTTTGCTCTTGTTTCATTT

TTCATGTAATACGCACTTGCTGAGTTTTAGGTGGGAGAAAAGTTTTCGGAATTTCCGAAGGTTTCT
GTGAAT

>Glyma.11G251900

CCAAACTCAAGGCACCTTGAGCGTCCAGGTACTTGAAAAAGCAAGCCAGGCATTAACAACACG
AATAGCACCACAGCCACAACCACAGGCCCACAGTCCAACATTATTAATCTATTCTTCCAATTTAT
TAATGTGTATTGATAAGTAAGAAAGAACACACAGAGAGTCCTTGTTGTGCTATTCAGAGAAAAT
ATGTACTGCTATATATATACTATGTATTAGGAAGGTGTCGTGCATGGGTGGAGGCCTCGGTGATT
AAGCTACGTTTGGATTCACCGCCACTGTGCACGTGGTGCTTGTGGTTCAGATTTGGATTAAGGATA
ACTTGCTTCACATCATAATTCTAACTATTATCCATGTGCTTCACCTTTCACCGTTTATTTCATGCTC
AAGCAAGGAATTATATTAGTTATAGAGCCAGACAGACACTACTCATGTTTAGAAATTCGGAACTT
AATTAATAATTGGCTTGGTTTCATGTTCCATACTTCATAAATCTTCTTAAAGGAACTCCACTCATC
AGCTATATAGCAAGGATGACAACTTCACAATCATTCACTTGTATTCATAAAAAAAATATCTACAA
TGACTAGAATGATTTTTTTAGGGAAACAAATGCTGTACTATTTTTTTTATATAAAAAATGTCAGG
GAATTTCTTGCAAATCGATTTGATACAAAAATATTTATATTTTTTAAAGTAAAAAATATTTTCAAA
CTGTAAAAATATACTCCTATATTTAAAGAATAAAATACACAATTTAAAAGATTGAGAATTTCACT
CTCCTAAGTTTCATAATTAAAACATTTTTTATATTCTTGATCTCTTTCATATCTCTTTTGAATGTCCA
ATTCACGCATTATCTCTCCAATCACCAAAAAAAAAATTGTATAATATTACATCACTTAAATAATTG
AAACTTGTGCAAAACTAGCATTGAAAGTTTAGTGAAGTTTTCTTTTTGCTGAATGTAAAGTTTAGT
CAAGTTGATCACTAAGGTGGCAGCGAACCAGGAACATAGTAGACTCGTTCGAGTTTGGGCCTTCC
AAGAAAAAAATGTTGTGATGAATATTGGGCTGGAAAAAAAAGAATTTAAATCCACTTCAGTAACT
TAACACAGCCTTCGAAGAAAAAAAAGTTGTAATGAATCTAATTGTTTTTTAAAATAAAACTTATTA
TTTATTTTAAATTTAATTATATATAAATAATTTGTTACATATGATCCCTACTTAAAAGTTGCTTGAT
TATGGTTGTAATTCCCTAATCCCTAAACTGTTACCATTACATTATTACATAGTTCAGCCATATGAA
GTTATGAACAGAAATTGTAACTATTCAAGAAGACTTCATCAATAGACTTCTGAATACACGATCTC
TAACTTCATTCTTTTGAGCGTTTAATTTCTCTGTATTTTGTCGGTATCAAAAGAGTATAGTAGCTAT
AGGGGAGAAATTCAAAAACCAAAGAAAATGATGCATGTTAGGTCTACTCAAGTACCAAGAGAG
TGACATTTGCGACTTATTCCCAGTCAGGTCATGCTTTTCTTACTGATTCACGTTACTCACGAGTTCT
CTTCAGGTCACCACCACCAATATTCATTTGTTCACACTTTCAAGTCTTTACTCACTGCAACTGAAT
TGCTACACAATAATTAAGGTTGTGTTTGATTTAGGATGAGAGAAAAAAAAGAACTTTATACTTTT
TATATGGAATTCATACTTTTATTACTTTTAAGTTAAAAAGAAATCTTCTATTTCTATTCTCTTTTTAT
CTTCTAAATCAAACATATCCTTAACTTTTCTTCAAAGGGAGTCGAACGAAATTTCCTCCGCGAGA
CATATCTTATCCATAATTCTCTAACTTTGGTACAATTTATCCAAGAAATTCGTCGTTAGATGTCTCC
GACACTTTCAATTTACATTACATATAACCACCACTGTTTACTTATTGTTACATACTTAGATGTCAA
AAATATTTTTAAAATTAAGAAATATAACAAAAGCTTATCCATAAAATTTTCAAAACAATAATTAC
AATACATTGGCATTATAAATATTCATTATACTACCATCGATCTAATCCGCCTCGAGCAAATAAGT
TCATGGACACAATTGTTGTCCTTCCTACCCACATGGAGGAATAATTGATTGGATTTCTATAGTTTC
AGATATAGAAAAGACGATTATTATTAAGTTCTTACTTCTTAGTGTGTGTGTGGGCACCATTAAGTT
CTGCATGATTCCAAGACTGACGTGAGCAAGAAACAATAAGATGAGTTCTGAATTCTTGGGCTAAT
TTGACATTGTC

>Glyma.08G105200

GAGTTTGTTATAGTTCTTTGGCTTGTTATAGAAAATTTATTATTGTTGTTCATGAAGGTAGTTATAT
TATGTTATATTTTCTTTGTTGTTAATAATATCGAATGTCTTGTGTTTTTAATAATGTCTTCATATAAG

ATTTTGTAAGTAATGTGATATTACAATGAAAATAATATTTTTAAATATTTTAATTTAAAATTATTAT
ATTTTTTTTTATAAATTGGTTCTAGTTGATTTATTCTAAAAACAAATAAAATATTATTTTTTTGTTGC
AAGAAAACAACAACGATTGATCTATAATTGATGTAGAAAGTATTTTTATACATTTTTTACATTAAT
TTTTTCAAAAAGCAATATTGTATAAGACTTTTAAAATTGATTTTTTCAAAACTGATATAGAAAGTA
CTTCATAATACATGATTCAACATTGATTTTTTCAAAAATCAATATTGTACAAAAAAACAATATCA
ATTTTGTTTGAAATTGATGTTGAATTTTTGCATATTTTACATTTTTTTTCTTTTTTCTTATAATACAAT
ATTAGTTTTCTTAAAAATCGATATTATAAAGTGTATTTTAGCATCGATTTTCATAATTGATGTTAAC
ATTGATACTTTTAATGTTCATAATTTCAACATCGATTTAAAACTGATGAATGTATAAAATAATTAA
TGTTAAAAACATTTTTTTCAATAATAGTGACTTTGAAATTGTTTGTTTTGAAATGCGGATGAAGTC
AATGCCATATTTATATATTGAAAGTCAATATCACTATATCAACTCAAACGCCTACTACTTCTAATA
AATCTTCATCAACCTAGAATTAATCCTCAACTTCAAGTCTCGTTCACACCGAGCAAGCTTGTCTTT
TTGTGTTTAATAATGACCAGGTCTTCCTTTATGATGATATCATTGCTATGCAGATCAGCGAGATAT
TCAGCCCAGTAACCTCATTCACAGTTTTACTCTACATTTTATATTTGACATTCTGTTTTTATTAATAT
TATAATTAAGTTGACCTAAATGTACATGTATATGTATATATATAATAAGAAAAATGTGAATTTCT
AGAGGGAAGTATTTTCCTTTTGCTCACATTTGAACCACAATCGTAGTGGTAAACAACTGTGTTTGG
GAATTAGATTTTCACTACCAATAAAAACAAAACATTTCAGTCGGTTTTACACTTCTGTAACAAAA
AAAAAGTCCGTTTTACAATTTAATCATTAGTTTGTGGCGACATTTCCGTGCGCAATAATTATATGA
GCGGCATTTTCTAAATTTGTAATAAAAGTTTACTTTTCATTTTGTATCCACGTAAAATATTTTTATA
TTGATTAAACTTTTGATTTATTGATCATCCACTCAATAATGAATGTGTTCGCCTTGCATATCGCTTA
AAAACACCTTGGCTCATTTATACATTAAATATTTTACTAAGATAAAAAATGAAAATATTTAAAAT
AAATACAAATATAATCGAAGAGTAGAATTATTTATAATATTCTTTTTTATCTTTATTCGCGCAATC
GAATATTTATTTAGTCTTTTGATATTTCTTTAATTATTTAAGAGGGTAGTTAGCAAAGTTAACAAA
AAGTAAATTTTGTGTATAATACTGCTACATATTTATTAAAGTTAACAAAAAGTTAAAAGGACTAC
CTGATTATGATTACCGCTTAGAAAAAATCAAACTTGCTCCAACTTGCAAAGTCTTTTTTTTAATCA
GTGTAAAGTCTTTCCCACATAGATACTGAATATTCTAAAAATAAATTAAAGAGGTGAGAAAAGC
AAGACATATAGTAGTAGCAATCTAATAGTAAATAGTAATATCTCTTGGTCTTGGTCTTGGTCTTGG
TCTTGGTCATGGTCTCCATATAGTATTGTATATATATAAAAAAAATTTGTTTAATAAAAGCACGTT
TCGCAAACCTCAACCTTAATCACAACCTCAAATCAACTCTGTAATAAGCTTCAACTAACGCTAAC
TGTGTCTGTTTCAGCTTTCTGCTTTTGCAACTTCCTTTCTCCGGTCCATTGTTTGCAGCGAATCTAGC
AGCCTCTGCGGTTCCAAAAATGCTCTGTGTCCGGTTCTACTACCTGTACTTGTAGCTTTTTATTCCT
CGGATTCAACACC

Alignment of Arabidopsis and Soybean CAMTA family protein sequences

```
             ....|....| ....|....| ....|....| ....|....| ....|....| ....|....| ....|....|
                     10         20         30         40         50         60         70
AtCAMTA1     MARKKKSVSF SHLDIARNEG NKIISFSVHA YSDRLGFVDS LFDYESLRSL LVDFWVYPSM VDRRSFGSIT
AtCAMTA2     ---------- ---------- ---------- ---------- ---------- ---------M ADRGSFG-FA
AtCAMTA3     ---------- ---------- ---------- ---------- ---------- ---------M AEARRFSPVH
AtCAMTA4     ---------- ---------- ---------- ---------M SSVAEDNSFT CDIATIFVAI CRNPPANPSD
AtCAMTA5     ---------- ---------- ---------- ---------- ---------- -MAGVDSGK- LIGSEIHGFH
AtCAMTA6     ---------- ---------- ---------- ---------- ---------- -MDGDGLGR- LIGSEIHGFH
GmCAMTA1     ---------- ---------- ---------- ---------- ---------- ---------M AEARHYVPPS
GmCAMTA2     ---------- ---------- ---------- ---------- ---------- ---------M AEARLYAPPS
GmCAMTA3     ---------- ---------- ---------- ---------- ---------- ---------M AEGASYG-LR
GmCAMTA4     ---------- ---------- ---------- ---------- ---------- ---------M SERSSFG-LG
GmCAMTA5     ---------- ---------- ---------- ---------- ---------- ---------M AERSCFG-LG
GmCAMTA6     ---------- ---------- ---------- ---------- ---------- ---------M AEGASYG-LR
GmCAMTA7     ---------- ---------- ---------- ---------- ---------- ---------M AETTKYIPNS
GmCAMTA8     ---------- ---------- ---------- ---------- ---------- MMSHNLTGQ- LVSAEIHGFH
GmCAMTA9     ---------- ---------- ---------- ---------- ---------M MMSHNLTGQ- LVGAEIHGFH
GmCAMTA10    ---------- ---------- ---------- ---------- ---------- ---------- --------MT
GmCAMTA11    ---------- ---------- ---------- ---------- ---------- ---------- ---------M
GmCAMTA12    ---------- ---------- ---------- ---------- ---------- -MANNLAAQQ LVGSEIHGFH
GmCAMTA13    ---------- ---------- ---------- ---------- ---------- -MANNLAVQ- LVGSEMHGFH
GmCAMTA14    ---------- ---------- ---------- ---------- ---------- ---------- ---------M
GmCAMTA15    ---------- ---------- ---------- ---------- ---------- ---------- --------MT
Clustal Co

             ....|....| ....|....| ....|....| ....|....| ....|....| ....|....| ....|....|
                     80         90        100        110        120        130        140
AtCAMTA1     PPLQLDMEQL LSEAQHRWLR PTEICEILQN YHKFHIASES PTRPASGSLF LFDRKVLRYF RKDGHNWRKK
AtCAMTA2     P--RLDIKQL LSEAQHRWLR PAEICEILRN HQKFHIASEP PNRPPSGSLF LFDRKVLRYF RKDGHNWRKK
AtCAMTA3     E---LDVGQI LSEARHRWLR PPEICEILQN YQRFQISTEP PTTPSSGSVF MFDRKVLRYF RKDGHNWRKK
AtCAMTA4     SLFQYEISTL YQEAHSRWLK PPEVLFILQN HESLTLTNTA PQRPTSGSLL LFNKRVLKFF RKDGHQWRRK
AtCAMTA5     TLQDLDIQTM LDEAYSRWLR PNEIHALLCN HKFFTINVKP VNLPKSGTIV LFDRKMLRNF RKDGHNWKKK
AtCAMTA6     TLQDLDVQTM LEEAKSRWLR PNEIHAILYN PKYFTINVKP VNLPNSGRII LFDRKMLRNF RKDGHNWKKK
GmCAMTA1     Q---LDIKQI ILEAQHRWLR PAEICAILSN HKKFLIASEP AHMPPSGSLF LFDRKVLRYF RKDGHNWRKK
GmCAMTA2     Q---LDIKQI ILEAQHRWLR PAEICAILGN YKKFRIAPEP AHMPPSGSLF LFDRKVLRHF RKDGHNWRKK
GmCAMTA3     R--PLDIQQL QFEAQHRWLR PAEICEILRN YRMFHITSEP HNRPPSGSLF LFDRKVLRYF RKDGHNWRKK
GmCAMTA4     P--RLDLQQL QLEAQHRWLR PAEICEILRN YRMFQITSEP PNRPPSGSLF LFDRKVLRYF RKDGHNWRKK
GmCAMTA5     P--RLDLQQL QLEAQHRWLR PAEICEILRN YQMFQITSEP PNGPPSGSLF LFDRKVLRYF RKDGHNWRKK
GmCAMTA6     R--SLDIQQL QFEAQHRWLR PAEICEILRN YRMFHITSEP HNRPPSGSLF LFDRKVLRYF RKDGHNWRKK
GmCAMTA7     Q---LELEEI LNEAEHRWLR PAEICEILRN HKKFKLTPDP PVMPPAGSLF LFDRKALRYF RKDGHRWRKK
GmCAMTA8     TLEDLDVSNT MEEAKSRWLR PNEIHAILCN HKYFKINVKP VNLPKSGTIV LFDRKMLRNF RKDGHNWKKK
GmCAMTA9     TLDDLDVSNT MEEAKSRWLR PNEIHAILCN HKYFKINAKP VNLPKSGTIV LFDRKMLRNF RKDGHNWKKK
GmCAMTA10    PGYEYDINDL HQEAQARWLK PAEVMYILQN HEKFQFTQEP PQQPTSGSLF LFNKRVLRFF RKDGHNWRKK
GmCAMTA11    AGLEYSIDDL FQEAKRRWLK PVEALYILRN HDQCKFTHQP PHQPAGGSLF LFNRRIMRSF RKDGHNWRKK
GmCAMTA12    TLQDLDVGSI MEEARTRWLR PNEIHAMLCN YKYFTINVKP VNLPKSGTIV LFDRKMLRNF RKDGHNWKKK
GmCAMTA13    TLQDLDVGSI MEEARTRWLR PNEIHAMLCN YKYFTINVKP VNLPKSGTIV LFDRKMLRNF RKDGHNWKKK
GmCAMTA14    AGLEYSIDDL FQEAKRRWLK PVEALYILRN HDQCKFTHQP PHQPAGGSLF LFNRRIMRSF RKDGHNWRKK
GmCAMTA15    PGYEYDINDL HQEAQARWLK PAEVMYILQN HEKFQFTQEV PQQPTSGSLF LFNKRILRYF RRDGHNWHKK
Clustal Co        .:      **  ***: * *   :* *      :        * .*:.  :*::: :: * *:***.*::*

             ....|....| ....|....| ....|....| ....|....| ....|....| ....|....| ....|....|
                    150        160        170        180        190        200        210
AtCAMTA1     KDGKTIREAH EKLKVGSIDV LHCYYAHGEA NENFQRRCYW MLEQYYYRKA SSHWVLVATL SLFSFGYLRP
AtCAMTA2     KDGKTVKEAH EKLKVGSIDV LHCYYAHGED NENFQRRCYW MLEQ------ ---------- ----------
AtCAMTA3     KDGKTVKEAH ERLKAGSVDV LHCYYAHGQD NENFQRRSYW LLQE------ ---------- ----------
AtCAMTA4     RDGRAIAEAH ERLKVGNAEA LNCYYAHGEQ DPTFRRRIYW MLDP------ ---------- ----------
AtCAMTA5     KDGKTIKEAH EHLKVGNEER IHVYYAHGED TPTFVRRCYW LLDK------ ---------- ----------
AtCAMTA6     KDGRTVKEAH EHLKVGNEER IHVYYAHGED NTTFVRRCYW LLDK------ ---------- ----------
GmCAMTA1     KDGKTVREAH ERLKAGSVDV LHCYYAHGEE NENFRRRTYW LLEE------ ---------- ----------
GmCAMTA2     KDGKTVREAH ERLKAGSVDV LHCYYAHGEE NENFQRRTYW LLEE------ ---------- ----------
GmCAMTA3     KDGKTVKEAH EKLKVGSVDV LHCYYAHGEE NENFQRRSYW MLEP------ ---------- ----------
GmCAMTA4     KDGKTVKEAH EKLKVGSVDV LHCYYAHGEE NENFQRRSYW MLEL------ ---------- ----------
GmCAMTA5     KDGKTVKEAH EKLKVGSVDV LHCYYAHGEE NENFQRRSYW MLEP------ ---------- ----------
GmCAMTA6     KDGKTVKEAH EKLKIGSVDV LHCYYAHGEE NENFQRRSYW MLEP------ ---------- ----------
GmCAMTA7     KDGKTVREAH EKLKAGSVDV LHCYYAHGED NEYFQRRSYW MLDE------ ---------- ----------
```

```
GmCAMTA8     TDGKTVKEAH EHLKVGNEER IHVYYAHGQD NPTFVRRCYW LLDK------ ---------- ----------
GmCAMTA9     TDGKTVKEAH EHLKVGNEER IHVYYAHGQD KPTFVRRCYW LLDK------ ---------- ----------
GmCAMTA10    RDGRTVGEAH ERLKVGNVEA LNCYYAHGEQ NPTFQRRSYW MLDP------ ---------- ----------
GmCAMTA11    KDGKTVGEAH ERLKVGNVEI LNCYYAHGEE NRTFQRRSYW MLEP------ ---------- ----------
GmCAMTA12    KDGKTVKEAH EHLKVGNEER IHVYYAHGQD NPNFVRRCYW LLDK------ ---------- ----------
GmCAMTA13    KDGKTVKEAH EHLKVGNEER IHVYYAHGQD NPNFVRRCYW LLDK------ ---------- ----------
GmCAMTA14    KDGKTVGEAH ERLKVGNVEI LNCYYAHGEE NRTFQRRSYW MLEP------ ---------- ----------
GmCAMTA15    SGGRTVGEAH ERLKVLNVEA LNCYYARGEQ NPAFQRRSYW MLDP------ ---------- ----------
Clustal Co    .*::: *** *:**  . :  :: ***:*:     * ** ** :*:


             ....|....| ....|....| ....|....| ....|....| ....|....| ....|....| ....|....|
                    220        230        240        250        260        270        280
AtCAMTA1     SWVRHLMHIV FVHYLEVKGN RTS------- -----IG-MK ENNSNSVNGT ASVNIDS--- ----------
AtCAMTA2     ----DLMHIV FVHYLEVKGN RMS------- -----TSGTK ENHSNSLSGT GSVNVDS--- ----------
AtCAMTA3     ----ELSHIV FVHYLEVKGS RVSTSFNRMQ RTEDAARSPQ ETGDALTS-E HDGYASCSFN QNDHSNHSQT
AtCAMTA4     ----EYEHIV LVHYRDVSER EEGQ------ -----QTGG- -------QVY QFAPILSTQN VSYNQYIGDS
AtCAMTA5     ----SQEHIV LVHYRETHEV H--------- ---------- ---------- ---------- ----------
AtCAMTA6     ----ARENIV LVHYRDTQEA A--------- ---------- ---------- ---------- ----------
GmCAMTA1     ----ELSHIV LVHYRHVKGT KAN--FTCAK ENEETLPYAQ QTDKIMPKTE METSLSSTLH PHSYQVPSQT
GmCAMTA2     ----ELSHIV LVHYRQVKGT KAN--FTSAK ENEESLPYAQ QTDKIMPQTE MDTSLSSTLH PHSYQVPSKT
GmCAMTA3     ----DMMHIV FVHYLEVKGN K-N------- -----IVVNN EGDEVPTDSQ KVTSPSSSLP THHSCVSSLS
GmCAMTA4     ----DMMHIV FVHYLDVKVN KTN------- -----IGGKT YSDEVTSDSQ KSSSLSSGFP RNYGSMPSGS
GmCAMTA5     ----DMMHIV FVHYLDVKVN KTN------- -----VGGKT YSDEVTSDSQ KSSSLSSGFP RNYGSVPSGS
GmCAMTA6     ----DMMHIV FVHYLEVKGN K-N------- -----IVVNT EGDEIPSDSQ KVTSSSSSLP THHSSVPSLS
GmCAMTA7     ----QLEHIV LVHYREIKEG CKSG------ -----ISHLP VVPVTLVGSS QNTSVLS--- ----------
GmCAMTA8     ----NLEHIV LVHYRDTQEL QLQ------- ---------- ---------- ---------- ----------
GmCAMTA9     ----SLEHIV LVHYRDTQEL QLQ------- ---------- ---------- ---------- ----------
GmCAMTA10    ----AYDHIV LVHYRNTSEG KLSS------ -----GAGAQ LSPSSSSVYT QSPSPYSTQN PGSTSILGDS
GmCAMTA11    ----EYDHIV LVHYRETSEG ---------- ---------- ---------- KSNSEHVTQL P---------
GmCAMTA12    ----SMEHIV LVHYREIQEM Q--------- ---------- ---------- ---------- ----------
GmCAMTA13    ----SMEHIV LVHYRETQEM Q--------- ---------- ---------- ---------- ----------
GmCAMTA14    ----EYDHIV LVHYRETSEG ---------- ---------- ---------- KSNSEHVTQL P---------
GmCAMTA15    ----AYEHIV LVHYRNTSEG KLSS------ -----GAGAQ LSPSSS-VYT QSPSPYSTQN PGSTSILGDS
Clustal Co          :** :*** .


             ....|....| ....|....| ....|....| ....|....| ....|....| ....|....| ....|....|
                    290        300        310        320        330        340        350
AtCAMTA1     --TASPTSTL SSLCEDADT- -------VLV QG-IVNKQVP -SYDHLLNLK LEIAMVGHL- ----------
AtCAMTA2     --TATRSSIL SPLCEDADSG DSRQASSSLQ QNPEPQTVVP -QIMHHQNAS TINSYNTTS- ----------
AtCAMTA3     TDSASVNGFH SPELEDAESA YNQHGSSTAY SHQELQQPAT GGNLTGFDPY YQISLTPRD- ----------
AtCAMTA4     SDIYQQSSTS PGVAEVNSN- ---------- -----LEGSA SSSEFGQALK MLKEQLSIG- ----------
AtCAMTA5     ---------- ---------- ---------- ----AAPATP GNSYSSSITD HLSPKIVAE- ----------
AtCAMTA6     ---------- ---------- ---------- ----TTSGDS ISSPISVSEQ TFPNRVAAE- ----------
GmCAMTA1     MD-RSMNSSQ ASEYEEAESA FNNHASSEFY SFLELERPVE KITPQPADSY SPRPLTRKSV PNMNCIIESG
GmCAMTA2     VD-TSMNSAQ TSEYEEAESA FNNHASSEFY SFLELQRPVE KISPQPADFY SPRPLIN--- ----------
GmCAMTA3     TDSVSPTTSL MSLHEDADSE DIHHASSGLH PLHESQHSGN SPLTEKIGAG SNSSYLMHP- ----------
GmCAMTA4     TDSMSPTSTL TSLCEDADSE DIHQASSGLH SYRESQNLGN DRPMDKIHAR SNSSYLMHP- ----------
GmCAMTA5     TDSMSPTSTL TSLCEDADSE DIHQASSGLH SYRESQNLGN DRPMDKIDAR SNSSYPMHP- ----------
GmCAMTA6     TDSVSPTTSL MSLREDADSE DIHQASSGLR PLYESQHSGN GPLTEKIGAG SNSSYLIHP- ----------
GmCAMTA7     ---STKINSP ISLVQTSFTS SANKVYQNGR ASEHEDVNSK NGPQASSHAQ PISNYVLHS- ----------
GmCAMTA8     ---------- ---------- ---------- ----GSPATP VNSNSSSASD PAASWIPSE- ----------
GmCAMTA9     ---------- ---------- ---------- ----GSPATP VNSNSSSVSD SAASWIPSD- ----------
GmCAMTA10    YEPNQ-SFSS PGSTEVTSDM FVLNNKMGHM DGTDTESGTS PELEVTQALR RLEVQLSLN- ----------
GmCAMTA11    ---------- ---SEVT--- ---------- ---------- ---FEAQALR QLEEQLSLN- ----------
GmCAMTA12    ---------- ---------- ---------- ----GSPVTP VNSHSSSVSD PPAPWILSE- ----------
GmCAMTA13    ---------- ---------- ---------- ----GSPVTP VNSHSSSVSD PPAPWILSE- ----------
GmCAMTA14    ---------- ---SEVT--- ---------- ---------- ---FEAQALR QLEEQLSLN- ----------
GmCAMTA15    YEPNQ-SFSS PGSTKVTSEI FVLNNKMGHM DWADTESGTS SELEVTQALR RLEVQLSLN- ----------
Clustal Co


             ....|....| ....|....| ....|....| ....|....| ....|....| ....|....| ....|....|
                    360        370        380        390        400        410        420
AtCAMTA1     LLA------- -------CVM FHRFMGT--- ------ESEK ---------- ---------- ----------
AtCAMTA2     VLGNR----- ---DGWTSAH GNRVKGS--- ------NSQR SGDVPAWDAS FE-NSLARYQ NLPYNAPLTQ
AtCAMTA3     -SYQKELRTI P-VTDSSIMV D-KSKTINSP GVTNGLKNRK SIDSQTWEEI LG-NCGSG-V EALPLQPNSE
AtCAMTA4     ---------- ---------- ---------- -------DEH VNSVDPHYIQ PESLDSLQFL EYSDIDHLAQ
AtCAMTA5     ---------- ---------- ---------- ---------- ---------D TSSGVHNTCN TGFEVRSNSL
AtCAMTA6     ---------- ---------- ---------- ---------- ---------D IDTVVR---- ----------
```

```
GmCAMTA1     TDDQEKSPVI PGVNYISLTQ DNKIKDIHNF GLT--YESPK PLGFSSWEGI LKNNAGSQHV PFQPLFPGTQ
GmCAMTA2     --DQEKLPII PGVNYISLTQ DNKNKDILNA GLT--YESPK PLGFSSWEGI LENNAGSQHV HFQPLFPGTQ
GmCAMTA3     FSGDNEQSSI SGTDYVPVVH GDKFRGND-- --TAYTDGQK PHGMAPWGTV LQ-STAKLHN D-PSLASFPS
GmCAMTA4     FSDNHGQLPV SGAEYIPHVQ GNKSRASD-- --TTYIEGQR AHGIASWDNA ME-QSAGKHA D-PSLVSSTS
GmCAMTA5     FSGDHGQLPV SGAEYIPHVL GDKSRASD-- --TTYIEGQR AQGIASWDNT ME-QSAGEYA D-PSLVSSTT
GmCAMTA6     FSGDYEQSSI SGTDYIPVVH GDKFRGND-- --TAYIDGQK THDVATWSTV LQ-STAKLHN D-PSLASSPS
GmCAMTA7     ---------- ------APWL THEAAGFS-- ------ELLR NPLISSWPSS FP-----SYS PGTGLSPWTS
GmCAMTA8     ---------- ---------- ---------- ---------- ---------D LDSGVNSAYA VELN---DNL
GmCAMTA9     ---------- ---------- ---------- ---------- ---------D LDSGVNSAYA VELN---DSL
GmCAMTA10    ---------- ---------- ---------- -------EDN FEDIVSFGSK HETTHDSNPQ HDQRVISNQE
GmCAMTA11    ---------- ---------- ---------- -------DDG FN-------- -EIALDLVSG QDQRVVYKQD
GmCAMTA12    ---------- ---------- ---------- ---------- ---------E IDSGTTTAYA GDTS---ANI
GmCAMTA13    ---------- ---------- ---------- ---------- ---------E IDSGTTTAYT GDMS---NNI
GmCAMTA14    ---------- ---------- ---------- -------DDG FN-------- -EIALDLVSG QDQRVVYKQD
GmCAMTA15    ---------- ---------- ---------- -------EDN FEDIVSFGSK HETVHDSNPK HDQRVISNQE
Clustal Co


             ....|....| ....|....| ....|....| ....|....| ....|....| ....|....| ....|....|
                    430        440        450        460        470        480        490
AtCAMTA1     MQPSNTDSML VEENSEKGGR LK-----AEH IRNPLQTQFN WQ-------- ---------- ------DDTD
AtCAMTA2     TQPSTFGLIP MEGKTEKGSL LT-----SEH LRNPLQSQVN WQTPVQES-- -VPLQKWPMD SHSGMTDATD
AtCAMTA3     ---------- ---HEVLDQI LESSFTMQ-- ---------- -------DFA SLQES----- ----------
AtCAMTA4     PTTVYQRPEN NKLERCYGGN FGAQYSAKND SNKLERCYGG YVGG------ ---------- ------AEYH
AtCAMTA5     GSRNHEIRLH EINTLDWDEL LVPADISNQS HPTEEDMLYF TEQL------ ---------- ----------
AtCAMTA6     ---NHDISLH DINTLDWDEL LVPTDLNNQS APTVDNLSYF TEPL------ ---------- ----------
GmCAMTA1     PDNMGINSKF SQGHEIMVPY LTTSIAKQHE NGSLIQAEGN WQA---YDVD SLRMSSWPID SAYSGSSCDI
GmCAMTA2     PDNMGINSNF SQGEEIMVPY LTTSIAKQHE NGSIIKAEGN WQV---YDVD SLRMSSWPID SAYSGSTCEV
GmCAMTA3     ILPSSMGDVL EQEHTIFGDL LMSKSGLTEE AESSQSLQSN WQIPFEDNSG GMPMLTQTQS FGLQFRSDYG
GmCAMTA4     IPSSAMGNIL DKNHTVPGNL LGHKIALTEV ERGAQPVQSN WQIPFEDNTG ELPNWGFTQS LGLEFGSDYG
GmCAMTA5     IPSSAVGNIL EENHTVPGKL LGRKNALTEE ERGSQPVQSN WQIPFEDNTG ELPNWGFTQS LGLEFGSDYG
GmCAMTA6     IPSSSMGDVL EQEHTIFSDL LMSKSGLTEV AESSQSLQSN WQIPFEDNSG GMPMLTQTQT FGLQFRSDYG
GmCAMTA7     IQNSSRNTIN MHDGKHHVEA SEADLTVRKL SNAGLDSVHR MQDG------ ---------- ----------
GmCAMTA8     TAKSHEQRLH EINTLEWDDL VVPN-VNTST TSNGGNVPYS FQEN------ ---------- ----------
GmCAMTA9     TAKSHEQRLH EINTLEWDDL VVSN-ANTST TSNGGNVPYS FQQN------ ---------- ----------
GmCAMTA10    QSAAFSGP-- DDQGLFYDGY NGRQGDGGEF YHELIDHGYP DGNE------ ---------- ------KALW
GmCAMTA11    KSAALSGP-- NDLGQPCDGY NGRQDDSGTY YHDFLDDCPG GNEK------ ---------- ------TIYW
GmCAMTA12    NVKSHELRLH EINTLDWDDL VDANDHNTTT VPNGGTVPYF DLQD------ ---------- ----------
GmCAMTA13    NVKSHELRLH EINTLEWDDL VDTNDHNAST VPNGGTVPYF DQQD------ ---------- ----------
GmCAMTA14    KSAALSGP-- NDLGQPCDGY NGRQDDSGTY YHDFLDDCPG GNEK------ ---------- ------TIYW
GmCAMTA15    QSAAFSRP-- DDQGLFYDGC NGRQ------ -----DHGYP DANE------ ---------- ------KALW
Clustal Co


             ....|....| ....|....| ....|....| ....|....| ....|....| ....|....| ....|....|
                    500        510        520        530        540        550        560
AtCAMTA1     LALFEQSAQD N----FETFS SLLGSENLQP FGISYQAPPS NMDSEYMP-- ---------- -------VMK
AtCAMTA2     LALFGQGAHE N----FGTFS SLLGSQDQQS S--SFQAPFT NNEAAYIPKL GPEDLIY-EA SANQTLPLRK
AtCAMTA3     MVKSQNQELN SG-------- --LTSDR--T VWFQG--QDM ELNAISNLAS NEKAP----- ---YLSTMKQ
AtCAMTA4     SSNLMLVKNG SGPSGGTGGS GDQGSESWKD VLEACEASIP LNSEGSTPSS AKGLLAGLQE DSNWS--YSN
AtCAMTA5     QTAPRGSVKQ GNHLAGYNGS VDIPSFPGLE DPVYQNNNS- -CGAGEFSSQ HSHCGVDPNL QRRDFSATVT
AtCAMTA6     QNAANGTAEH GN-------- ---------- ---------- ---------- ---------- ------ATVA
GmCAMTA1     TCSNREQEVN DVDLQKSLEQ CLLHPYKQNK VFMQNDPQEK LLNEKEKIKS DLEANRILDG IEDTYFTFKR
GmCAMTA2     SCSNCEQEVN DVDFQKSLEQ CLLHSHKQNK VLMQNDLQEK LLNEKEKIKS NLEAY----G IEDTYLSFKR
GmCAMTA3     TGLLGNETRN ASSEIAPILY SFHGEPKEQP MQQNYPQELE DGQSQHALKS NSANKVPDEE TINYGLTVKS
GmCAMTA4     TSLLGDVTNN AGPEIDPELF TFNGELKEQ- --------YT HGQSQPALKS NSAYEVPGEA SINYALTMRR
GmCAMTA5     ASLLGDVTNN AGPEIVPELF TFNGELKEQS VHQNFSKLYT HGQSQPTLKS NSEYEVPGEA SINYALTMRR
GmCAMTA6     TGLLGNETRN ACSESAAILY SFNGEPKEQP MQQNYLQELE DGQSQHALKS NSANKVPDEE TINYGLTVKR
GmCAMTA7     -VIFRDRLIT DMCVQPVIDL PTVNQVKNEH GLDSFHAQVH DHNDHPVVAT TKILVEQKLQ DGGLYNDESE
GmCAMTA8     QSLLSGRFGN VSS----NPS AEIPSFGNLT QPVSGSNSAP YSFPDSAILL ---------- -KNSPISSGG
GmCAMTA9     QSLLNGSFGN VSS----DPS AEIPSFGNLT QLVSGSDSAP YSFPESADLL ---------- -KSSPLSSGG
GmCAMTA10    TEVLESCKSS S--------- --------AV KLPQKNVYMP VENLENSVSS ARRVPVSNQE NSHWLNFNSN
GmCAMTA11    TKVLESCKPL S--------- --------VT KLPDQHAYEA IGN-ENTLFS SGRGVIANLE NNQWLNSNSN
GmCAMTA12    QILLNDSFSN VAN----NLS ADIPSFGSLT QPIAGSNSVP YNFSS-VNLQ TMDDQANPHE QRNNTVSLSG
GmCAMTA13    QILLNDSFGN VAN----NLS AEIPSFGNLT QPIAGSNRVP YNFSESVTLQ TMDNQANPHE QKNNTVSLSG
GmCAMTA14    TKVLESCKPL S--------- --------VT KLPDQHAYEA IGN-ENTLFS SGRGVIANLE NNQWLNSNSN
GmCAMTA15    TEQLESHKSS S--------- --------AV KLPQKNVYMP AEN-ENSVSS ARRVPVSNQE NSHWLNFNCN
Clustal Co


             ....|....| ....|....| ....|....| ....|....| ....|....| ....|....| ....|....|
```

```
                    570        580        590        600        610        620        630
AtCAMTA1     ILRRS---ED SLKKVDSFSK WAIKELGEM- -----EDLQM Q-SSRGDIAW TTVECET--- --------AA
AtCAMTA2     ALLKK---ED SLKKVDSFSR WVSKELGEM- -----EDLQM Q-SSSGGIAW TSVECEN--- --------AA
AtCAMTA3     HLLHGALGEE GLKKMDSFNR WMSKELGDVG VIADANESFT Q-SSSR-TYW EEVESED--G SNGHNSRRDM
AtCAMTA4     QVDQSTFLLP QDLGSFQLPA SYSALVAPEN NGEYCGMMED GMKIGL---- ---------- ---------P
AtCAMTA5     DQPGDALLNN GYGSQDSFGR WVNNFISDSP GSVDDPSLEA VYTPGQ---- ---------- -----DSSTP
AtCAMTA6     DGSLDALLND GPQSRESFGR WMNSFISESN GSLEDPSFEP MVMPRQ---- ---------- -----DPLAP
GmCAMTA1     TLLDGSPAEE GLKKLDSFNQ WMSKELADV- -----EESNK P-STSG-GYW DTVESENEVG NTTIPSQGHL
GmCAMTA2     TLLDGPPAEE GLKKLDSFNQ WMSKELGDV- -----EESNK P-STSG-GYW DTVETENEVG NTTIPSQGHL
GmCAMTA3     TLLDR---DE SLKKVDSFSR WITKELGEV- -----ADLNM Q-SSPG-ISW STDECQH--- --------VI
GmCAMTA4     GLLDG---EE SLKKVDSFSR WMTKELAGV- -----DDLHM Q-SSPG-ISW STDECGD--- --------VI
GmCAMTA5     GLLDG---EE SLKKVDSFSR WMTKEFAGV- -----DDLHM Q-SSPG-ISW STDECGD--- --------VI
GmCAMTA6     TLLDK---DE SLKKVDSFSR WITKELGEV- -----ADLNM Q-SSPG-ISW STDECQH--- --------VI
GmCAMTA7     QVEYG----- EMKKLDSFGR WMDKEIGGD- ----CDNSLM A-SDSG-NYW STLDAHSEDK EVSSLRHMQL
GmCAMTA8     VDTLGTLVNE GLQSQDSFGT WMN-IISDTP CSIDESALKA SISSVH---- ---------- -----VPYSS
GmCAMTA9     VDTLGTLVNE GLQSQDSFGT WMN-IMSDTP CSIDESALEA TTSSVH---- ---------- -----VPYSS
GmCAMTA10    NSENSVFSQP QGVDEVKFPV YSSMVETQVI NSDYYETLFD QSQIGA---- ---------- ---------P
GmCAMTA11    NIEN------ --YGGVKFPP YS-LAETPGA NSDYYETFFD QFQNQG---- ---------- ---------P
GmCAMTA12    VDSLDTLVND RLQSQNSFGM WVNPIMSDSP CSVDDPALES PVSSVH---- ---------- -----EPYSS
GmCAMTA13    VDSLDTLVND RLQSQDSFGM WVNHIMSDSP CSVDDPALES PVSSIH---- ---------- -----EPYSS
GmCAMTA14    NIEN------ --YGGVKFPP YS-LAETPGA NSDYYETFFD QFQNQG---- ---------- ---------P
GmCAMTA15    NSENSVFSQP QGVDEVKFPA YSSMLETQVI NSDYYETLFD QSQIGA---- ---------- ---------P
Clustal Co                    .:

             ....|....| ....|....| ....|....| ....|....| ....|....| ....|....| ....|....|
                    640        650        660        670        680        690        700
AtCAMTA1     AGISLSPSLS EDQRFTIVDF WPKSAKTDAE VEVMVIGTFL LSPQEVTKYN WSCMFGEVEV PAEILVDGVL
AtCAMTA2     AGSSLSPSLS EDQRFTMIDF WPKWTQTDSE VEVMVIGTFL LSPQEVTSYS WSCMFGEVEV PADILVDGVL
AtCAMTA3     DGYVMSPSLS KEQLFSINDF SPSWAYVGCE VVVFVTGKFL KTREETEIGE WSCMFGQTEV PADVISNGIL
AtCAMTA4     FEQEMRVTGA HNQKFTIQDI SPDWGYANET TKVIIIGSFL CDPTES---T WSCMFGNAQV PFEIIKEGVI
AtCAMTA5     PTVFHSHSDI PEQVFNITDV SPAWAYSTEK TKILVTGFFH DSFQHLGRSN LICICGELRV PAEFLQMGVY
AtCAMTA6     QAVFHSHSNI PEQVFNITDV SPAWAYSSEK TKILVTGFLH DSYQHLERSN LYCVCGDFCV PAEYLQAGVY
GmCAMTA1     DTYVLDPSVS HDQLFSIIDY SPSWAFEGSE IKVIISGRFL RSQHEAEQGK WSCMFGEVEV PAEIIAKGVL
GmCAMTA2     DTYVLDPSVS HDQLFSIIDY SPSWAFEGSE IKVIISGEFL RSQHEAEQCK WSCMFGEVEV PAVIIAKGVL
GmCAMTA3     DDTSLSPSLS QDQLFSINDF SPKWAYAESE IEVLIIGSFL KSQPEVTTCN WSCMFGEVEV PAEVLADGIL
GmCAMTA4     DDTSLHLSLS QDQLFSINDF SPKWAYAESE IEVLIVGTFL KSQPVVAKCN WSCMFGEVEV PAEVLADGIL
GmCAMTA5     DDTSLNLSLS QDQLFSINDF SPKWAYAESE IEVLIVGTFL KSQPVVAKCN WSCMFGEVEV PAEVLADGIL
GmCAMTA6     DDTSLSPSLS QDQLFSINDF SPKWAYAESE IEVLIIGSFL KSQPEVTTCN WSCMFGEVEI PAKVLADGIL
GmCAMTA7     DVDSLGPSLS QEQLFSIHDF SPDWAYTGVR TKVLIVGTFL GSKKPSSETK WGCMFGEIEV SAEVLADNVI
GmCAMTA8     LVADNLQSSL PEQVFNLTEV SPTWASSTEK TKVLVTGYFH NNYENLAKSN LLCVCGDVSV PVEIVQVGVY
GmCAMTA9     LVADNKQSSL PEQVFNLTEV SPVWASSTEK TKVLVTGYFH NNYQHLAKSN LLCVCGDVSV PVEIVQVGVY
GmCAMTA10    PDANSSLTVA QKQKFTIKTI SPEWGYATET TKVIVVGSLL CHPSDS---A WACMFGDVEV PVEIIQDGVI
GmCAMTA11    LGVDSSLTVV QKQKFTIRAV SPEYCYSTET TKVIIIGSFL CHDSDS---T WACMFGDVEV PAEIIQDGII
GmCAMTA12    LIVDSQQSSL PGQVFTITDV SPTCVSSTEK SKVLVTGFFH KDYMHLSKSN LLCVCGDVSV PAEIVQVGVY
GmCAMTA13    LVVDSQESSL PEQVFTITDV SPTCVSSTEK SKVLVTGFFL KDYMHLSKSN LLCVCGDVSV PAEIVQVGVY
GmCAMTA14    LGVDSSLTVV QKQKFTIRAV SPEYCYSTET TKVIIIGSFL CHDSDS---T WACMFGDVEV PAEIIQDGII
GmCAMTA15    PDANSSLTVA QKQKFTIKTI SPEWGYATET TKVIVVGSFL CHPSDS---A WACMFGDVEV PIEIIQDGVI
Clustal Co          :     * *.:     *           ::: * :               *: *:  : .   :  .:

             ....|....| ....|....| ....|....| ....|....| ....|....| ....|....| ....|....|
                    710        720        730        740        750        760        770
AtCAMTA1     CCHAPPHTAG HVPFYVTCSN RFACSEVREF DFLSGS---- -TQKINATDV YGTYTNEASL QLRFEKMLAH
AtCAMTA2     CCHAPPHEVG RVPFYITCSD RFSCSEVREF DFLPGS---- -TRKLNATDI YGANTIETSL HLRFENLLAL
AtCAMTA3     QCVAPMHEAG RVPFYVTCSN RLACSEVREF EYKVAE---- -SQVFDREAD DE-STIDILE -ARFVKLLCS
AtCAMTA4     RCEAPQCGPG KVNLCITSGD GLLCSEIREF EYREKPDTCC PKCSEPQTSD MSTSPNELIL LVRFVQTLLS
AtCAMTA5     RCFLPPQSPG VVNLYLSVDG NKPISQLFSF EHRSVQ---- FIEKAIPQDD QLYKWEEFEF QVRLAHLLFT
AtCAMTA6     RCIIPPHSPG MVNLYLSADG HKPISQCFRF EHRAVP---- VLDKTVPEDN QDSKWEEFEF QVRLSHLLFT
GmCAMTA1     CCHTPPHKAG RVPFYVTCSN RLACSEVREF DFQVNY---- -TPEVNTTGE NRGSTFDTFS -IRFGELLSL
GmCAMTA2     CCHTPPHKAG RVPFYVTCSN RLACSEVREF DFQVHY---- -TPED-TTGE NRGSTFDTFS -IRFGELLSL
GmCAMTA3     CCQAPCHKVG RVPFYVTCSN RLACSEVREF DFREGF---- -ARNVDFADF Y-ISSTEMLR HLRLEDFLSL
GmCAMTA4     CCQAPPHKIG RVPFYVTCSN RFACSEVREF EYREGF---- -DRNINFPDF F-NNSSEMEL HLRLVGLLSL
GmCAMTA5     CCQAPPHKIG RVPFYVTCSN RFACSEVREF EYREGF---- -DRNIQFADC F-NNSTEMVL HLRLVGLLSL
GmCAMTA6     CCQAPRHKVG RVPFYVTCSN RLACSEVREF DFREGF---- -ARNVDFADF Y-NSS-EILL HLRLEDFLSL
GmCAMTA7     RCQTPLHSPG RVPFYITCSN RLACSEVREF EFDENP---- -TKFLGPEGI KISPEEEVRL QMRLLKLVDL
GmCAMTA8     RCCVPPHSPG LVNLYLSFDG HKPISQVVNF EYRTPI---- LHEPTASMEE K-YNWNEFRL QMRLAHLLFA
GmCAMTA9     RCWVSPHSPG LVTLYLSFDG HKPISQVVNF EYRTPI---- LHEPTALIEE K-YNWDEFRL QMRLAHLLFA
GmCAMTA10    SCEAPSHLPG KVTLCITSGN RESCSEVREF EYRDKT-NSC TQCTQSET-E ATRSPEELLL LVRLEQMLLS
GmCAMTA11    CCEAPSNHLG KVNLCITSGN RVPCSEMREF EFRNKT-TSC TRCNSLET-E GSKSPEDLLL LVRFAEMLLS
GmCAMTA12    RCWVSPHSPG FVNLYMSIDG HKPISQVVNF EYRTPA---- LHDPAVSMEE S-DNWDEFQL QMRLAYLLF-
```

```
GmCAMTA13   RCWVSPHSPG FVNLYLSIDG HKPISQVVNF EYRTPA---- LHDPAVSMEE S-DNWDEFRQ QMRLAYLLFA
GmCAMTA14   CCEAPSNHLG KVNLCITSGN RVPCSEMREF EFRNKT-TSC TRCNSLET-E GSKSPEDLLL LVRFAEMLLS
GmCAMTA15   SCEAPSHLPG KVTLCITSGN WESCSEVREF EYHDKT-NSC TRCTQSET-E ATRSPEELLL LVRLGQMLLS
Clustal Co   *  .    *  * : :: ..     *:   * :.                          :      *:   :

            ....|....| ....|....| ....|....| ....|....| ....|....| ....|....| ....|....|
                   780        790        800        810        820        830        840
AtCAMTA1    RDFVHEHHIF EDVGDKRRQI SKIMLLK--E EKEYLLPG-- TYQRDSTKQE PKGQLFRELF EEELYIWLIH
AtCAMTA2    RCSVQEHHIF ENVGEKRRKI SKIMLLK--D EKEPPLPG-- TIEKDLTELE AKERLIREEF EDKLYLWLIH
AtCAMTA3    -KSENTSPVS GNDSDLSQLS EKISLLLFEN DDQLDQMLMN ----EISQEN MKNNLLQEFL KESLHSWLLQ
AtCAMTA4    DRSSERKSNL ESGND----- -KLLTKLKAD DDQWRHVIGT IIDGSASSTS TVDWLLQELL KDKLDTWLSS
AtCAMTA5    SSNKISVLTS KISPENLLEA KKLASRTSHL LNSWAYLMKS IQANEVPFDQ ARDHLFELTL KNRLKEWLLE
AtCAMTA6    SSNKLNVLSS KISPHNLRDA KKLASKTNHL LNSWAYLVKS IQGNKVSFDQ AKDHLFELSL KNRLKEWLME
GmCAMTA1    GHAFPQNSDS ISVSEKSQLR SKINSLLREE EDDWDKLLKL TQEEDFSPEN LQEQLLQNLL KDKLHAWLLQ
GmCAMTA2    GHAFPQNSDS ISVSEKSQLR SKINSLLRED DDDWDKLLKL TQEKDFSPEN LREQLLQNLL KDKLHAWLLQ
GmCAMTA3    KPVDPSNHSF EGDMEKRNLI FKLISLR--E EEDYSIKDEV TRELDISQHM VKEHLFHRQF KEKLYSWLLH
GmCAMTA4    NSMHTLNQVF EGDMDKRNLI FKLISLK--E EEEYSSKEET TAEMDISQQK LKEHMFHKQV KEKLYSWLLH
GmCAMTA5    NSVRTSNQVF EGDMDKRSLI FKLISLK--E EEEYSSKEET TAEMDISKHK LKELMFHKQV KEKLYSWLLH
GmCAMTA6    KPVDPSNHSF EGDMEKRNLI FQLISLR--E VEEYSIKDEV TTELDISQHM VKEHLFHKQF KEKLYSWLLH
GmCAMTA7    GPDNKWLKCS VSECEKCKLK GTMYSVR--- -DDSGVFEET FQIDGIGHIN HRDILFQRLV RDKLYEWLIY
GmCAMTA8    SDTSLNIFSS KVSPNALKEA RRFSFKTSYI SKSWQYLMKS IDDNTIPFSK VKDSLFETAL KNKLKEWLLE
GmCAMTA9    SDKSLNIFSS KVSTNALKEA RRFSFKTSYI SKSWQHLMKS IDDKTIPFSQ VKDALFETSL KNKLKEWLLE
GmCAMTA10   -ASTIKNDNI ESGI------ -PLIK-QKAD DDSWSHIIEA LLVGSGTSTG TVDWLLEELL KDKLQQWLSC
GmCAMTA11   -SSTTKDDRI ESGS------ -HLSTEQKDD DDSWSHIIDT LLDSTRTPSD AVKWLLEELL KDKLQLWLSN
GmCAMTA12   KQLNLDVIST KVSPNRLKEA RQFALKTSFI SNSWQYLIKS TEDNQIPFSQ AKDALFGIAL KSRLKEWLLE
GmCAMTA13   KQLNLDVISS KVSPNRLKEA RQFALKTSFI SNSWQYLIKS TEDNQIPFSQ AKDALFGITL KNRLKEWLLE
GmCAMTA14   -SSTTKDDRI ESGS------ -HLSTEQKDD DDSWSHIIDT LLDSTRTPSD AVKWLLEELL KDKLQLWLSN
GmCAMTA15   -ASTIKNDNI ESGI------ -PLIK-PKAD DDSWSHIIDA LLVGSGTSSG TVDWLLEELL KDKFQQWLSF
Clustal Co                          :         ..                       ::   . .. :  **

            ....|....| ....|....| ....|....| ....|....| ....|....| ....|....| ....|....|
                   850        860        870        880        890        900        910
AtCAMTA1    KVTEEGKGP- NILDEDGQGI LHFVAALGYD WAIKPVLAAG VNINFRDANG WSALHWAAFS GREETVAVLV
AtCAMTA2    KVTEEGKGP- NILDEDGQGV LHLAAALGYD WAIKPILAAG VSINFRDANG WSALHWAAFS GREDTVAVLV
AtCAMTA3    KIAEGGKGP- SVLDEGGQGV LHFAASLGYN WALEPTIIAG VSVDFRDVNG WTALHWAAFF GRERIIGSLI
AtCAMTA4    RSCDEDYIT- CSLSKQEQGI IHMVAGLGFE WAFYPILAHG VNVDFRDIKG WSALHWAAQF GSEKMVAALI
AtCAMTA5    KVIENRNTK- -EYDSKGLGV IHLCAVLGYT WSILLFSWAN ISLDFRDKQG WTALHWAAYY GREKMVAALL
AtCAMTA6    KVLEGRNTL- -DYDSKGLGV IHLCASLGYT WSVQLFSLSG LSLNFRDKQG WTALHWAAYY GREKMVAALL
GmCAMTA1    KITEEGKGP- NILDEGGQGV LHFASALGYD WALEPTIVAG VNVNFRDVNG WTALHWAAFC GRERTVAFLI
GmCAMTA2    KITEEGKGP- NVLDEGGQGV LHFAAALGYD WALEPTIVAG VNVNFRDVNG WTSLHWAAFC GRERTVAFLI
GmCAMTA3    KVTENGKGP- NVLDEDGQGV LHLAAFLGYD WAINPIISAG VNINFRDVNG WTALHWAASC GRERTVAVLV
GmCAMTA4    KVTETGKGP- LVLDEEGQGV LHLIAALGYD WAINPIITAG VNINFRDVNG WTALHWAAFC GRERTVAVLV
GmCAMTA5    KVTETGKGP- LVLDEEGQGV LHLIAALGYD WAINPIITAG VNINFRDVNG WTALHWAAFC GRERTVAVLV
GmCAMTA6    KVTESGKGP- NVLDEDGQGV LHLAAFLGYD WAINPIISAG VNINFRDVNG WTALHWAASC GRERTVAVLV
GmCAMTA7    KVHEGGKGP- HVLDDEGQGV IHLAAALGYV WAMAPLVAAG ISPNFRDSRG RTGLHWASYF GREETVIVLV
GmCAMTA8    RIILGRKST- -EYDAQGQGV IHLCAMLGYS WAISLFSWSG LSLDFRDKFG WTALHWAASY GMEKMVATLL
GmCAMTA9    RIILGSKST- -EYDAQGQAA IHLCAMLGYN WAISLFTWSG LSLDFRDKFG WTALHWAAYY GIEKMVATLL
GmCAMTA10   RSQEKDEETG CSLSKKEQGI IHMVAGLGFE WALNPILTCG VNINFRDING WTALHWAARF GREKMVASLI
GmCAMTA11   R---RDEGTG CSLSKKEQGI IHMVSGLGFE WALNPILSCG VNINFRDING WTALHWAARF GREKMVASLI
GmCAMTA12   RIVLGCKTT- -EYDAHGQSV IHLCAILGYT WAVSLFSWSG LSLDFRDRSG WTALHWAAYC GREKMVATLL
GmCAMTA13   RIVLGCKTT- -EYDAHGQSV IHLCAILGYN WAVSLFSWSG LSLDFRDRFG WTALHWAAYC GREKMVATLL
GmCAMTA14   R---RDEGTG CSLSKKEQGI IHMVSGLGFE WALNPILSCG VNINFRDING WTALHWAARF GREKMVASLI
GmCAMTA15   RSREKDEETG CSLSKKEQGI IHMVAGLGFE WALNPILTCG VNINFRDING WTALHWAARF GREKMVASLI
Clustal Co  :             .    .  :*: : **:  *:.      . :. :***  *  :.****:   * *  :  *:

            ....|....| ....|....| ....|....| ....|....| ....|....| ....|....| ....|....|
                   920        930        940        950        960        970        980
AtCAMTA1    SLGADAGALT DPSPELPLGK TAADLAYANG HRGISGFLAE SSLTSYLEKL TVDSKENSPA NSCGEKAVQT
AtCAMTA2    SLGADAGALA DPSPEHPLGK TAADLAYGNG HRGISGFLAE SSLTSYLEKL TVDAKENSSA DSSGAKAVLT
AtCAMTA3    ALGAAPGTLT DPNPDFPSGS TPSDLAYANG HKGIAGYLSE YALRAHVSLL SLN------- -DKNAETVEM
AtCAMTA4    ASGASAGAVT DPSRQDPNGK TAASIAASNG HKGLAGYLSE VALTNHLSSL TLEETENS-- -KDTAQVQTE
AtCAMTA5    SAGARPNLVT DPTKEFLGGC TAADLAQQKG YDGLAAFLAE KCLVAQFKDM QTAGNISG-- -------NLE
AtCAMTA6    SAGARPNLVT DSTKDNLGGC MAADLAQQNG YDGLAAYLAE KCLVAQFRDM KIAGNITG-- -------DLE
GmCAMTA1    SLGAAPGALT DPCPEHPSGR TPADLASANG HKGIAGYLAE SSLSAHLTTL DLNRDAG--- ENSGAKVVQR
GmCAMTA2    SLGAAPGALT DPCPEHPSGR TPADLASANG HKGIAGYLAE SSLSAHLTTL DLNRDAG--- ENSGAKVVQR
GmCAMTA3    SMGADCGALT DPSPAFPSGR TAADLASSYG HKGISGFLAE SSLTHHLETL TMDDQKGGQQ EISGMKVVQT
GmCAMTA4    SMDAAAGALT DPCPEFPLGR TPADLASSKG HKGISGFLAE SLLTSHLESL TMDENKDGRK ETSGMKVVQT
GmCAMTA5    SMGAAAGAWT DPCPEFPSGR SPADLASSKG HKGISGFLAE SLLTGHLESL TMDENKDGRK ETSGTKVVQT
```

```
GmCAMTA6      SMGADCGALT DPSPSSPAGR TAADLASSYG HKGISGFLAE SSLTHHLETL TMDDQKGGRQ EISGMKAVQT
GmCAMTA7      QLGATPGAVE DPTSAFPRGQ TAADLGSSRG HKGIAGYLAE ADLTNQLSVL TVKENETG-- NIATTIAANS
GmCAMTA8      SCGARPNLVT DPTPQYPGGC TAADLAYMKG CDGLAAFLSE KSLVEQFNEM SLAGNISG-- -------SLE
GmCAMTA9      SCGARPNLVT DPTPQYPGGC TAADLAYVKG CDGLAAYLSE KSLVEQFNDM SLAGNISG-- -------SLE
GmCAMTA10     ASGASAGAVT DPNAQDPTGK TAASIAAGNG HKGLAGYLSE IAVTSHLSSL TLEESELS-- -KSSAELQAD
GmCAMTA11     ASGASAGAVT DPSSQDPTGK TAASIAASHD HKGLAGYLSE VDLTSHLSSL TLEESELS-- -RESSELEAE
GmCAMTA12     SAGAKPNLVT DPTPQNPGGC TAADLAYMRG HDGLAAYLSE KSLVQHFNDM SLAGNISG-- -------SLE
GmCAMTA13     SAGAKPNLVT DPTPQNPGGC TAADLAYMRG HDGLAAYLSE KSLVQHFNDM SLAGNISG-- -------SLE
GmCAMTA14     ASGASAGAVT DPSSQDPTGK TAASIAASHD HKGLAGYLSE VDLTSHLSSL TLEESELS-- -RESSELEAE
GmCAMTA15     ASGASAGAVT DPNAQDPTGK TAASIAASSG HKGLAGYLSE IAVTSHLSSL TLEESESS-- -KSSAYLQAD
Clustal Co      .*  .    *.      *   .:.:.   .   *::.:*:*   :   .  :

              ....|....| ....|....| ....|....| ....|....| ....|....| ....|....| ....|....|
                     990       1000      1010      1020      1030      1040      1050
AtCAMTA1      VSERTAAPMT YGDVPEKLSL KDSLTAVRNA TQAADRLHQV FRMQSFQRKQ LCDIGDDE-- ----KIDISD
AtCAMTA2      VAERTATPMS YGDVPETLSM KDSLTAVLNA TQAADRLHQV FRMQSFQRKQ LSELGGDN-- ----KFDISD
AtCAMTA3      APS-PSSS-- --------SL TDSLTAVRNA TQAAARIHQV FRAQSFQKKQ LKEFG-DK-- ----KLGMSE
AtCAMTA4      KTLNSISEQS PSGNEDQVSL KDTLAAVRNA AQAAARIQAA FRAHSFRKRK QREAALVA-- -CLQEYGMYC
AtCAMTA5      TIKAEKSSNP GNANEEEQSL KDTLAAYRTA AEAAARIQGA FREHELK-VR SSAVRFAS-- -------KEE
AtCAMTA6      ACKAEMLN-Q GTLPEDEQSL KDALAAYRTA AEAAARIQGA FREKALKAAR SSVIQFAN-- -------KEE
GmCAMTA1      VQN-IAQVND LDGLSYELSL KDSLAAVRNA THAAARIHQV FRMQSFQRKQ LKEYD-DD-- ----KLGLSD
GmCAMTA2      LQN-IAQVND LDGLSYELSL KDSLAAVCNA TQAAARIHQV FRMQSFQRKQ LKEYD-DD-- ----KLGLSD
GmCAMTA3      VSERSATPVH YCDIPDAICL KDSLTAVRNA TQAADRIHQV YRMQSFQRKQ LTQYEGDD-- ----ELGLSD
GmCAMTA4      VSERTATPVL NGDIPDDICL KDSLNAVRNA TQAADRIYQV FRMQSFQRKQ LALYE-DD-- ----EFGLSD
GmCAMTA5      ASERTATPVL YGDIPDAICL KDSLNAVRNA TQAADRIYQV FRMQSFQRKQ FAQYE-DD-- ----EFGLSD
GmCAMTA6      VSERSATPVH FGDMPD-LCL KDSLTAVRNA TQAADRIHQV YRMQSFQRKQ LTQYE-SD-- ----ELGLSD
GmCAMTA7      ALQSVEDDSS SMTMDEQHYL KESLAVFQKS AHAAASILAA FRARSFCQRQ LAQSS----- -----SDISE
GmCAMTA8      TSSTDPVN-A ENLTEDQLYV KETLAAYRIS AEAAARIQAA FREHSFK-LR YKAVEIIS-- -------PEE
GmCAMTA9      TSSTDPVN-A ANLTEDQLYL KETLEAYRTA AEAAARIQAA FREHSFK-LR YQAVEIMS-- -------PEE
GmCAMTA10     MTVNSVSKEN LTASEDQASL KDTLAAIRNV TQAAARIQSA FRSHSFRKRR AREVAAS--- -----AGGIG
GmCAMTA11     LTVSSVSEEN LVASEDQVSL KASLDAVRNA AQAAARIQAA FRAHSFRKRK ERDAAATVLD GYCIDAGSID
GmCAMTA12     TSTTDPVI-S ANLTEDQQNL KDTLAAYRTA AEAASRIHAA FREHSLK-LR TKAVASSH-- -------PEA
GmCAMTA13     TSTTDPVN-P ANLTEDQQNL KDTLTAYRTA AEAASRIHAA FREHSLK-LR TKAVASSN-- -------PEA
GmCAMTA14     LTVSSVSEEN LVASEDQVSL KASLDAVRNA AQAAARIQAA FRAHSFRKRK ERDAAATVLD GYCIDAGSID
GmCAMTA15     RTVNSVSKEN LTANEDQASL KDTLAAIRNV TQAAARIQSA FRSHSFRKRR AREATAS--- -----TGGIG
Clustal Co                        : . :* .     :.**  :  . :* : :   :

              ....|....| ....|....| ....|....| ....|....| ....|....| ....|....| ....|....|
                    1060      1070      1080      1090      1100      1110      1120
AtCAMTA1      QLAVSFAAS- -KTKNPG--Q GDVSLSCAAT HIQKKYRGWK KRKEFLLIRQ RIVKIQAHVR GHQVRKQYRT
AtCAMTA2      ELAVSFAAA- -KTKKSG--H SSGAVHAAAV QIQKKYRGWK KRKEFLLIRQ RIVKIQAHVR GHQVRKQYRA
AtCAMTA3      ERALSMLAP- -KTHKSGRAH SDDSVQAAAI RIQNKFRGYK GRKDYLITRQ RIIKIQAHVR GYQFRKNYRK
AtCAMTA4      EDIEGISAMS KLTFGKG--R NYN---SAAL SIQKNFRGYK DRKCFLELRQ KVVKIQAHVR GYQIRKNYKV
AtCAMTA5      EAKNIIAAMK IQHAFRN--F EVRRKIAAAA RIQYRFQTWK MRREFLNMRK KAIRIQAAFR GFQVRRQYQK
AtCAMTA6      EAKSIIAAMK IQNAFRK--Y DTRRKIEAAY RIQCRFQTWK IRREYLNMRR QAIRIQAAFR GLQARRQYKK
GmCAMTA1      ERALSLVKMN MKSHKSG--P RDEPVHAAAV RIQNKFRSWK GRREFLMIRQ RIVKIQAHVR GHQVRKSCGK
GmCAMTA2      ERALSLIKMN VKSHKSG--P RDEPVHAAAI RIQNKFRSWK GRREFLMIRQ RIVKIQAHVR GHQVRKSCGK
GmCAMTA3      QQALSLLAS- -RACKSG--Q GDGLANAAAV QIQKKFRGWK KRKEFLMIRQ RVVKIQAHVR GHQIRKQYKP
GmCAMTA4      QQALSLLAS- -KACRSG--Q GEGLANAAAI QIQKKFRGWT RRKEFLIIRQ RIVKIQAHVR GHQVRKQYKP
GmCAMTA5      QQALSLLAS- -KTCKSG--Q GEGLANAAAI QIQKKFRGWT KRKEFLIIRQ RIVKIQAHVR GHQVRKQYKP
GmCAMTA6      QQALSLLAS- -RACKSG--Q GDGLANAAAV QIQKKFRGWK KRQEFLMIRQ RV----AHVR GHQVRKQYKP
GmCAMTA7      VLDVVADSLS -KVQNKG--H FEDYLHFAAL KIQKRYRGWK GRKDFLKIRD RIVKIQAHIR GHQVRKQYKK
GmCAMTA8      EARQIVAAMR IQHAFRN--Y ESKKKMTAAA RIQHRFRTWK YRREFLNMRH QAIKIQAAFR GFQARKQYRK
GmCAMTA9      EARQIVAAMR IQHAFRN--Y ESKKKMAAAA RIQLRFRTWK YRREFLNMRR QAIKIQAAFR GFQARKQYRK
GmCAMTA10     -TISEISAMS KLAFRNS--R EYNSAASAAL SIQKKYRGWK GRKDFLALRK KVVKIQAHVR GYQVRKHYK-
GmCAMTA11     NNISVLSAMS KLSSQSW--R DYK----AAL SIQKKYRNWK GRIEFLALRQ KIVKIQACVR GYQVRKQYKL
GmCAMTA12     QARKIVAAMK IQHAFRN--H KTKKVMAAAA RIQCTYRTWK IRKEFLNMRC QAVKIQAAFR CFQVRKHYCK
GmCAMTA13     QARKIVAAMK IQHAFRN--H ETKKMMAAAA RIQCTYRTWK IRKEFLNMRR QAVKIQAAFR CFQVRKHYRK
GmCAMTA14     NNISVLSAMS KLSSQSW--R DYK----AAL SIQKKYRNWK GRIEFLALRQ KIVKIQACVR GYQVRKQYKL
GmCAMTA15     -TISEISAMS KLAFRNS--H EYN---SAAL SIQKKYRGWK GRRDFLALRQ KVVKIQAHVR GYQVRKHYK-
Clustal Co                                 **   **  :: :.  *  :*  *  :     * .*   * *:

              ....|....| ....|....| ....|....| ....|....| ....|....| ....|....| ....|....|
                    1130      1140      1150      1160      1170      1180      1190
AtCAMTA1      VIWSVGLLEK IILRWRRKGN GLRGFKRNAV AKTVEPEPPV SAICPRIPQE DEYDYLKEGR KQ-TEERLQK
AtCAMTA2      IIWSVGLLEK IILRWRRKGS GLRGFKRDTI SKPTEP---- --VCP-APQE DDYDFLKEGR KQ-TEERLQK
AtCAMTA3      IIWSVGVLEK VILRWRRKGA GLRGFKSEAL VEK--MQDGT -------EKE EDDDFFKQGR KQ-TEDRLQK
AtCAMTA4      ICWAVRILDK VVLRWRRKGV GLRGFRQDVE STED-S---- ---------- EDEDILKVFR KQKVDVAVNE
```

```
AtCAMTA5     ITWSVGVLEK AILRWRLKRK GFRGLQVS-- ---QPDEKEG --------SE AVEDFYKTSQ KQ-AEERLER
AtCAMTA6     ILWSVGVLEK AVLRWRQKRK GFRGLQVA-- ---AEEDSPG ---------E AQEDFYKTSQ RQ-AEERLER
GmCAMTA1     IIWSVGILEK VILRWRRKGS GLRGFKPEAN SEGTMIQDVS -------STD DDYDVLKEGR KQ-TEQRLQK
GmCAMTA2     IIWSVGILEK VILRWRRKGS GLRGFKPEAN SEGTMIQDVS -------STD DDYDVLKEGR KQ-TEQRLQK
GmCAMTA3     IIWSVGILEK VILRWRRKGS GLRGFRPNAI NKVPNQQ--- ----NDSLKE DDYDYLKEGR KQ-KEEKIQK
GmCAMTA4     IIWSVGILEK VILRWRRKGS GLRGFRPASQ NKVPEQP--- ----SESPKE DDYDYLKEGR KQ-SEVKFKK
GmCAMTA5     IIWSVGILEK VILRWRRKGS GLRGFRPAAL NKVPEQP--- ----SESPKE DDYDYLKEGR KQ-SEVKFKK
GmCAMTA6     IIWSVGILEK IILRWRRKGS GLRGFRPNVI NEVPDQQ--- ----NNSLKE DDYDYLKEGR KQ-KEEKIQK
GmCAMTA7     VVWSVSIVEK AILRWRRKGA GLRGFRVGQP VGVVVKD--- ------AEKS DEYEFLSIGR RQ-KSDDVKK
GmCAMTA8     IIWSVGVLEK VILRWRLKRK GFRGLQVNPA REETQESD-- --------SI AEEDFFRTGR KQ-AEERIER
GmCAMTA9     IVWSVGVLEK VILRWLLKRK GFRGLQVNPA EEETQESD-- --------TI AEEDFFRTSR KQ-AEERVER
GmCAMTA10    VIWAVGILDK VVLRWRRKGA GLRGFRQEMD INENEN---- ---------- EDEDILKVFR KQKVDVEIEE
GmCAMTA11    ILWAVGILDK VVLRWRRKRI GIQSVRQEME SNEEES---- ---------- DDADFLNVFR KEKVNAAIEK
GmCAMTA12    ILWSVGVVEK AVLRWRLKRR GFRGLQVKTV EAGTGDQDQQ --------SD VEEEFFRAGR KQ-AEERVER
GmCAMTA13    ILWSVGVVEK AVLRWRLKRR GFRGLQVKTV DAGTGDQDQQ --------SD VEEEFFRTGR KQ-AEERVER
GmCAMTA14    ILWAVGILDK VVLRWRRKRI GIQSVRQEME SNEEES---- ---------- DDADFLNVFR KEKVNAAIEK
GmCAMTA15    VIWAVGILDK VVLRWRRKGA GLRGFRQEMD INEN------ ---------- EDEDILKVFR KQKLDVEIEE
Clustal Co   : *:* :::*  :***  *   *::..:                          :     : ::  .  .:.

             ....|....| ....|....| ....|....| ....|....| ....|....| ....|....| ....|....|
                     1200       1210       1220       1230       1240       1250       1260
AtCAMTA1     ALTRVKSMVQ YPEARDQYRR LLTVVEGFRE NEASSSASIN NKEEEAVN-C EEDDFIDIES LLNDD--TLM
AtCAMTA2     ALTRVKSMAQ YPEARAQYRR LLTVVEGFRE NEASSSSALK NNTEEAANYN EEDDLIDIDS LLDDD--TFM
AtCAMTA3     ALARVKSMVQ YPEARDQYRR LLNVVNDIQE SKVEKALENS E--ATCFD-- DDDDLIDIEA LLEDDDTLML
AtCAMTA4     AFSRVLSMSN SPEARQQYHR VLKRYCQTKA ELGKTETLVG E---DDD--- ---GLFDIAD MEYDTLFSLP
AtCAMTA5     SVVKVQAMFR SKKAQQDYRR MKLAHEEAQL EYDGMQELDQ MATEES---- ---------- ----------
AtCAMTA6     SVVRVQAMFR SKKAQQDYRR MKLTHEEAQL EYGCLEDI-- ---------- ---------- ----------
GmCAMTA1     ALARVKSMVQ YPEARDQYHR LLNVVTEIQE NQVKHESSYN N--SEEPR-- EFGDLNDLEA LLDED--IFM
GmCAMTA2     ALARVKSMVQ YPEARDQYHR LLNVVTEIQE NQVKHESSSN N--SEEPR-- EFGDLNDLEA LLDED--IFM
GmCAMTA3     ALSRVKSMVQ YPEARAQYRR LLNVVEDFRQ TKASNKGLIN S--EETVD-- GVEDLIDIDM LLDDD--NFI
GmCAMTA4     ALSRVKSMVQ YPEARAQYRR VLNVVEDFRQ TKGGNLNLIN S--EETVD-- GVEDLIDIDM LLDDE--NFL
GmCAMTA5     ALSRVKSMVQ YPEARAQYRR VLNVVEDFRQ TKGGNLNLIN S--EETVD-- GVEDLIDIDM LLDDE--NFL
GmCAMTA6     ALSRVKSMAQ YPEARAQYRR LLNVVEDFRQ TKASNEGLIN S--EETVD-- GMEDLIDIDM LLDDD--NFI
GmCAMTA7     ALDRVKSMVR NPEARDQYMR LIMKYEKFKI DDGGSSQSQH VG-------- ---------- ----------
GmCAMTA8     SVIRVQAMFR SKKAQEEYRR MKLTHNQAKL ELELEEFLDS EVDMLPKT-- ---------- ----------
GmCAMTA9     SVIRVQAMFR SKKAQEEYRR MKLTHNQAML D-ELEEFLNS EDDMLPKT-- ---------- ----------
GmCAMTA10    AVSRVLSMVD SPDAREQYHR MLEKYRQAKA ELAGTSDEAS L---STS--- ---VGDD--- LFIDDFYPFP
GmCAMTA11    ALKRVLSMVH STGARQQYRR LLSLYRQAKI EHGSTSDEAP L---STSEEN ASNMEDDDLC QFLDTFFWPS
GmCAMTA12    SVVRVQAMFR SKKAQEEYRR MKLALDQAKL EREFERLLST EVDMQLKT-- ---------- ----------
GmCAMTA13    SVVRVQAMFR SKKAQEEYRR MKLALNQAKL EREYEQLLST EVDMQL---- ---------- ----------
GmCAMTA14    ALKRVLSMVH STGARQQYRR LLSLYRQAKI EHGSTSDEAP L---STSEEN ASNMEDDDLC QFLDTFFWPS
GmCAMTA15    AVSRVLSMVD SPDAREQYHR MLEKYRQAKA ELAGTSDEAS L---STS--- ---VGDD--- LFMDDFYPFP
Clustal Co   :. :* :*      *: :* * :

             ....|....

AtCAMTA1     MSISP----
AtCAMTA2     SLAFE----
AtCAMTA3     PMSSSLWTS
AtCAMTA4     ---------
AtCAMTA5     ---------
AtCAMTA6     ---------
GmCAMTA1     PTAT-----
GmCAMTA2     PTAT-----
GmCAMTA3     PIAFD----
GmCAMTA4     PIAFD----
GmCAMTA5     PIAFD----
GmCAMTA6     PIAFD----
GmCAMTA7     ---------
GmCAMTA8     ---------
GmCAMTA9     ---------
GmCAMTA10    ---------
GmCAMTA11    ---------
GmCAMTA12    ---------
GmCAMTA13    ---------
GmCAMTA14    ---------
GmCAMTA15    ---------
Clustal Co
```

Regulatory Network of ATCAMTA5 (GmCAMTA12 orthologue in Arabidopsis) predicted with String database.

(The *CGCG/CGTG* motif in promoter region is shaded in green)

1. **AT3G62140** (NIP-30)

Promoter (2 kb upstream)
ATATAATATTAGAAAACTTGTGTACAGGCATAATATTATGTGTACCCCGGCCCTTTTCGAATGTAT
ATCTCTTGTGAACCAGCTCCATTGGCTTTAACTGCGTGATATTTTATTGTGCTATATTTATGATGTG
TTTGTTTTTAAACTATCATGTATTTGTTTCTAACTCGCATGAGAATTGCCAATCTATTGGACGAACC
TAGAGACCATTTTTCGACTCAAATTTATATATTATTTTATCTGTGTGCAGATAGAGATTTGTAAGG
ATCATTGTATTTTAAAACGATTGATGGGAGTTGTAATTATGAACGTGGGTATAAGTGTTCATCATG
AAGTCGACCGAATATTAGTTCACATATATCTTAGTAATTTCTTATGTTATCCCCATAGATATATTTG
TATAGATATGATTCCTCTATATGGTTAATTAATATACCTTCTAGGCTAGCTTCTAGCCATTTACTTG
TTCTTCTGCGAAGTTGTTAAATATACTCTTTAAATATATCATCCCACATATTGTTGTGAATATCTTG
GGTTGGCGTATTATCATCTAATAAAGGTGTTTAGTTATATTTGACACAAAAGATAATAATATGGG
AATCCAATGAAAAAGAAAAGAATTTGCATTATATACCATATTAATTTTTTCTATATGATCAAAAT
TGAATACAGATGAATTGCACTGTTATTTTTCAATAAAACATTTTTCTTAGCATATTGACAAATATA
TTTCAACTATTCAATAAACAAAATAAAAAACAAATAGTCATAAATGGCTGATTCTGATTAGTTAA
CGAATAAACGTATTCCGAGAATTGCAGCTGATTTTTTATTAACACTAGGTAATCATTCGCGTTACG
CTGCGATATTTTCAGATTTTTTGTATTGTTTAATTTAAATATAATTTTTAATTATCTAATCATTCACT
CATGTAATAAGTAATTATTCTTAGTATTAAAATATATTTTCTAACAACATTCTCAACAACGCATTA
TACACAAAGTTCCTAAACAATGTTCGTCTTAAACCAATTAAACAGAGAGTAATGTTGGGATTCTC
GAAACCTAAAAATTTGATGATTAAACTTCTTACATACTTTTCACCCTCGGCGATTTATTATATAGA
TATATAGCAATATGCAAACTATTCAAACTATTAACTAATGCATGACTATTATACACTCATAATTA
GAGTTAAGATTACGAAAAAAAATCTTCAAGAAAACTAATAAAATAATTTTATTAAAAACATGTA
ATAACAAAATAAATTTAGTATTAATACAATTAAATATGTAAAATTAAATAATAAATATATGATTA
ATCTATAAATTAAAAATTAAAGAGCTTACATTAATTATTCAGTGGATACATGACAAATGCTAAAA
TGATGATATCATATGAAAAAAATTGGCCAACTTTCATATATATGATTGTCGTTATCATTCATCACT
TCTCTTTTCGCTACTCGTATATAATACGTAAATATGTGTGTACATATATGATGTTATATGATTCTCT
TCTCACATGAATAATCAAATTCGCTGTGGAGTGATCTTGTCATGAAACCATGTCAAGACAAGAAA
TCGTAATCTAATTAAAATGGATCAATAATGAGAGGTCGAATTCCGTTAAATAAATTAAAGATTAG
TAAAACTTGACATGGTATATATCCAAAAACAGTGTGGTGTAATGGTATTGTTCTCTTACAATAAT
ATGAATCTCGTTTATTCTTCTTGCGGGCAGAAAAAAAAAGTATTAATTTATAAAATACTTCTATTC
TAACGAATTATTTGCGCCGAGTAATCAAGAAACCAATATTATATTAGAACTTCACAAACTCAAGT
AAACTCAAAAAGATAATCTAAACTAAAAAGTACAAAGACTTGGCCATCGATTGGATTATCTATA
GGATTAATAAATAGAGTATCTCACTTACTTTAACTTATATTTCCATCCATACTTTAATATTAATATC
CGTTGATATTATACTTACTACTGAAA

CDS
ATGGTTGATGAGACAGAAAAACCTATTAGGTTACTCAATTTCGTCTCCGAGGAACAGTTAGATGA
ATCGAAGAAAGAAAGAGGAGAAAGAGTTGAAGATGGAACATTCCAGAGGGACAGGGCTCTCTA
CGAGATTTTGAAAGAGAACAAAGACAAGAAGGACGCTGAGTTTAATGAACGATTCAAGCATAG
GCCACCCAAAGCTCTGGATGAAGATGAGACCGAGTTTCTTGATAAACTGGAAATGTCAAAGAGG
GAATATGAACGTCAATTGGCAAATGAAGAAGATGAACAGCTTCGTAATTTCCAGGCAGCGGTTG

CAGCACGATCTGCAATCCTTCACGAACCAAAGGAAGCAGCACTGCCACCACCAGCGCCAGTCAC
CAAGGAACAAAAACCAATCGGGAAGAGAAATCCTGCGACTCGTCCATTTAAAGCGATCATAAA
AGTCAAGCCACAACCCAAGAAGGCTAAAGCAACAGAGAAAGAAGAGAAAGAGATCCCAGGGA
ATGGAAAACCGGCCAGCCATATCGACCAGGCTTCTTTAGATTCTGTAAAAGGCAATGTCACAGG
TAAAACTACTGAAGGTCTGCAAACTGGTTTAGCCTTAGTCTCATATAGTGATGAAAGTGAAGACG
ATGATTAA

Protein
MVDETEKPIRLLNFVSEEQLDESKKERGERVEDGTFQRDRALYEILKENKDKKDAEFNERFKHRPPKAL
DEDETEFLDKLEMSKREYERQLANEEDEQLRNFQAAVAARSAILHEPKEAALPPPAPVTKEQKPIGKRN
PATRPFKAIIKVKPQPKKAKATEKEEKEIPGNGKPASHIDQASLDSVKGNVTGKTTEGLQTGLALVSYSD
ESEDDD*

2. **AT1G30650** (WRKY14)

Promoter (2 kb upstream)
ATATAATATTAGAAAACTTGTGTACAGGCATAATATTATGTGTACCCCGGCCCTTTTCGAATGTAT
ATCTCTTGTGAACCAGCTCCATTGGCTTTAACTGCGTGATATTTTATTGTGCTATATTTATGATGTG
TTTGTTTTTAAACTATCATGTATTTGTTTCTAACTCGCATGAGAATTGCCAATCTATTGGACGAACC
TAGAGACCATTTTTCGACTCAAATTTATATATTATTTTATCTGTGTGCAGATAGAGATTTGTAAGG
ATCATTGTATTTTAAAACGATTGATGGGAGTTGTAATTATGAACGTGGGTATAAGTGTTCATCATG
AAGTCGACCGAATATTAGTTCACATATATCTTAGTAATTTCTTATGTTATCCCCATAGATATATTTG
TATAGATATGATTCCTCTATATGGTTAATTAATATACCTTCTAGGCTAGCTTCTAGCCATTTACTTG
TTCTTCTGCGAAGTTGTTAAATATACTCTTTAAATATATCATCCCACATATTGTTGTGAATATCTTG
GGTTGGCGTATTATCATCTAATAAAGGTGTTTAGTTATATTTGACACAAAAGATAATAATATGGG
AATCCAATGAAAAAGAAAAGAATTTGCATTATATACCATATTAATTTTTTCTATATGATCAAAAT
TGAATACAGATGAATTGCACTGTTATTTTTCAATAAAACATTTTTCTTAGCATATTGACAAATATA
TTTCAACTATTCAATAAACAAAATAAAAAACAAATAGTCATAAATGGCTGATTCTGATTAGTTAA
CGAATAAACGTATTCCGAGAATTGCAGCTGATTTTTTATTAACACTAGGTAATCATTCGCGTTACG
CTGCGATATTTTCAGATTTTTTGTATTGTTTAATTTAAATATAATTTTTAATTATCTAATCATTCACT
CATGTAATAAGTAATTATTCTTAGTATTAAAATATATTTCTAACAACATTCTCAACAACGCATTA
TACACAAAGTTCCTAAACAATGTTCGTCTTAAACCAATTAAACAGAGAGTAATGTTGGGATTCTC
GAAACCTAAAAATTTGATGATTAAACTTCTTACATACTTTTCACCCTCGGCGATTTATTATATAGA
TATATAGCAATATGCAAACTATTCAAACTATTAACTAATGCATGACTATTATACACTCATAATTA
GAGTTAAGATTACGAAAAAAAATCTTCAAGAAAACTAATAAAATAATTTTATTAAAAACATGTA
ATAACAAAATAAATTTAGTATTAATACAATTAAATATGTAAAATTAAATAATAAATATATGATTA
ATCTATAAATTAAAAATTAAAGAGCTTACATTAATTATTCAGTGGATACATGACAAATGCTAAAA
TGATGATATCATATGAAAAAAATTGGCCAACTTTCATATATATGATTGTCGTTATCATTCATCACT
TCTCTTTTCGCTACTCGTATATAATACGTAAATATGTGTGTACATATATGATGTTATATGATTCTCT
TCTCACATGAATAATCAAATTCGCTGTGGAGTGATCTTGTCATGAAACCATGTCAAGACAAGAAA
TCGTAATCTAATTAAAATGGATCAATAATGAGAGGTCGAATTCCGTTAAATAAATTAAAGATTAG
TAAAACTTGACATGGTATATATCCAAAAACAGTGTGGTGTAATGGTATTGTTCTCTTACAATAAT
ATGAATCTCGTTTATTCTTCTTGCGGGCAGAAAAAAAAAGTATTAATTTATAAAATACTTCTATTC
TAACGAATTATTTGCGCCGAGTAATCAAGAACCAATATTATATTAGAACTTCACAAACTCAAGT

AAACTCAAAAAGATAATCTAAACTAAAAAGTACAAAGACTTGGCCATCGATTGGATTATCTATA
GGATTAATAAATAGAGTATCTCACTTACTTTAACTTATATTTCCATCCATACTTTAATATTAATATC
CGTTGATATTATACTTACTACTGAAA

CDS
ATGTGCAGCGTCTCTGAGCTTCTTGACATGGAAAACTTCCAAGGAGACTTAACCGACGTCGTACG
AGGAATCGGAGGCCACGTGTTATCACCGGAGACTCCTCCCTCGAACATCTGGCCTCTTCCTCTGT
CACATCCAACACCATCACCGTCAGATCTTAACATAAACCCCTTCGGAGATCCCTTTGTGAGCATG
GACGATCCACTCCTCCAAGAACTAAACTCCATCACAAACTCCGGCTATTTCTCCACCGTAGGAGA
TAACAACAACAACATTCACAACAACAATGGTTTCTTGGTTCCAAAGGTATTTGAGGAGGATCATA
TAAAGAGTCAATGTAGTATCTTCCCAAGAATCCGGATCTCGCATAGTAACATCATCCACGATTCT
TCTCCGTGTAATTCTCCGGCCATGTCGGCTCACGTTGTCGCAGCCGCAGCAGCCGCCTCGCCGAG
AGGCATCATCAACGTAGACACAAACAGTCCTAGAAACTGTCTATTGGTTGATGGTACCACGTTCT
CCTCGCAGATTCAGATATCTTCCCCTCGGAATCTAGGCCTTAAAAGAAGGAAGAGTCAGGCAAA
GAAGGTGGTGTGTATTCCGGCCCCGGCTGCAATGAACAGCCGATCAAGCGGAGAAGTGGTTCCA
TCGGATCTATGGGCTTGGCGTAAATACGGTCAAAAACCTATCAAAGGCTCTCCTTTTCCAAGGGG
TTATTATAGATGCAGCAGCTCAAAAGGTTGTTCAGCAAGAAAGCAAGTCGAAAGAAGCCGAACC
GATCCAAACATGTTGGTGATTACATATACCTCCGAACATAACCATCCTTGGCCCATCCAACGCAA
CGCTCTCGCCGGCTCCACACGCTCCTCCACCTCCTCCTCATCTAACCCTAATCCTTCCAAACCCTC
AACCGCAAACGTAAACTCCTCATCCATTGGCTCCCAAAACACCATCTACTTGCCTTCCTCCACCA
CTCCTCCTCCTACCCTCTCATCCTCCGCCATCAAAGATGAACGAGGGGACGATATGGAGTTGGAA
AACGTAGATGATGATGATGATAACCAGATTGCTCCATACAGACCGGAGCTTCATGATCATCAGC
ACCAACCAGATGATTTCTTTGCAGATCTTGAAGAGCTAGAAGGAGATTCTCTAAGCATGTTGCTT
TCTCATGGCTGTGGCGGCGACGGGAAGGATAAAACGACCGCGTCCGATGGGATCAGCAATTTCT
TCGGGTGGTCGGGAGATAATAATTATAATAATTACGACGACCAAGACTCAAGGTCGTTATAG

Protein
MCSVSELLDMENFQGDLTDVVRGIGGHVLSPETPPSNIWPLPLSHPTPSPSDLNINPFGDPFVSMDDPLL
QELNSITNSGYFSTVGDNNNNIHNNNGFLVPKVFEEDHIKSQCSIFPRIRISHSNIIHDSSPCNSPAMSAH
VVAAAAAASPRGIINVDTNSPRNCLLVDGTTFSSQIQISSPRNLGLKRRKSQAKKVVCIPAPAAMNSRSS
GEVVPSDLWAWRKYGQKPIKGSPFPRGYYRCSSSKGCSARKQVERSRTDPNMLVITYTSEHNHPWPIQR
NALAGSTRSSTSSSNPNPSKPSTANVNSSSIGSQNTIYLPSSTTPPPTLSSSAIKDERGDDMELENVDDDD
DNQIAPYRPELHDHQHQPDDFFADLEELEGDSLSMLLSHGCGGDGKDKTTASDGISNFFGWSGDNNY
NNYDDQDSRSL*

3. **AT1G53720** (CYP59)

Promoter (2 kb upstream)
AAAAGGTCAACATTGAATTAGTTGTCCCCTGTTAAAATGGCTATCTTTAAAGCTAACTCAAGTTCT
GTATGATGAAATGGAAAGGTAGAAGTTTACATTACTTCTTGATCTTGAGAATGGCGCCAAAACCG
CTGTACATGAAACAGTATACATTAGACCATAAACAAAGTGTTAATCCTTTGAGTTACATACAATT
CTAGCTGACAAATCACCTGATCAATGACAGAACTACCACGATGAGGGCCACATGGGGTTTGATC
AGGCCGATATAATGGGATGTGGGTCAATAAAACTCTAGGATGTGACTGTGCGTCTAAAAAAGAT
AAGAAAATTTTATGTATAAGCTTCACAAAGATAGTATATACAGAAGAGGATGCAGCTGAATAAC
ACACACACCGGTGGAGACATTTTGTACAAACTTCCAAACTTCTGAGGCCAGATCCTTTTTAGAAT
TCCCTGCAACATATACATCACTTTTCAGTTACAGATCCTTGGTTTCTCTTTGAGGAGAAATTCTAA

GCATGTAAAACAATATCTTAATGATCAGAGCAAAGTTTGTAACAACCATCGATAGCTTGGGCATC
AATACTGATGAACTCAACATTTCCAATCATAAATCTGCGGTTTCTGACCCCAAATACTTTTTCGTA
TCGATCAATCACCTGTTGAATTGTGAAGGTATATCAATATATAGGTCGCTGACATATGTTATACAT
TTGAGAAAAAAAAAAGTGATAAATGTACTACGCCTTGCTTATGGGAAGCAACCCGAGAATAGCC
AATGTCATGATTCCCAGGGATGTAAAATGTAGGGATATCCCCAACTCTGCCTTCTGAATTCAAAC
CAAATACATGTTTCAATCGGTTTAAAGACTCCTGCCATCTGATAAAATAAGAAGTTAAGCATTTC
CACGAACCCTTTGAGCAGAAAGAAGAACAAGTGAAAGACAAGAACAAGGAGAGCTTACTCTTC
CTCGGAAAGAAATGGCCCGCCATCGAAATAATCACCTAAAAACAAAACAACATCGGGTTTGAA
AGGCAAGACAGACCGAAAGAATGATCTCCGCATGTTTATATCAGTATAAAACTGAGCAAGCTCC
AAAGCAAGTGTCTTTGACGACAAACGAAACGATGTCTTATCCATGAGCTGCATAAAAACAACAA
CAATAAACACATAATCAGATGATCAAACTCCATTAAAACAACACACAACAAAAATCGAAAATC
ATTTGAAGAGCTCACCTGTGGATCAGTAACAATGGCTACTTTAGTAAAATTTCCATCACTCTCCAC
TCCATCACTCTGCAACACAAAGAAGCTAACAGATATTTAGTAATCACAACTAAACAGTGACTAG
AATAAATGATGAATCATAGAGATTTCTTCCACATTGGCCTTCTAATAATGTTGCATCTAAGCTATT
TTTCGATTAGAGAACGATTACATTCTCAAGAATAAGAATATAAAAACCTTGGCTTTATACAATTT
ATAGCAACACAAGGATGATTACAGAGAGAAATAAGCAAATACACAATCAAGGTCGAATTAAAC
GCAATGAATCTCCAAAATTGAGCATCCGTTACTGAAAAAAATGGAAACTTTTTGGAGAGAGAGA
CCTTATGGTGAGGCCAAGAACAAGTGAAGAGAGAAGGAACCCAGAAGGCGAACATTTCTCCGT
AGAGGATCGTCGCAGCCCAAATGAGACATAACGCCACCGTGAGCTTATGGTGGTGTTTCATTTTT
TCCGAGTATCTGAATCTCCTCCCTTCTCGGCTTTGCTTCGCCGAGATATGTATCTAATGATCGTTGT
CGTTGACCAAAAGAGCGGTCAAGACGCGACAGACGCTGAGCTGCGTTTGAGTATACCGAAAAA
CGCCGAAACTTGGGAATGAATGCGCTATTCCAAAACTACTGGGCCGAGTTTATATTGGGCCTATA
CTTTTGTTGTTATTGGGCCTATGCTTATTAGAACAGTGACGAACAAAGAAATAATGAAAAAC

CDS

ATGTCAGTTCTTATTGTGACGAGCCTTGGTGATATAGTAATTGACCTTCACTCGGATAAATGCCCT
TTGACTTGCAAGAACTTCCTTAAGCTTTGCAAGATCAAGTACTACAATGGGTGTCTTTTTCACACT
GTGCAAAAGGATTTCACAGCACAGACTGGTGATCCAACTGGTACGGGAGCTGGTGGAGATTCAA
TCTACAAATTTCTGTACGGTGAGCAGGCTCGTTTTTACAAAGATGAGATTCATCTTGATTTAAAAC
ATTCAAAGACCGGCACTGTTGCAATGGCCAGTGGTGGAGAGAATCTCAATGCTTCCCAGTTCTAT
TTCACACTTCGTGATGATTTGGACTATCTTGATGGGAAACACACTGTGTTTGGACAGATTGCTGAG
GGGTTTGACACTTTGACTAGGATAAATGAAGCTTATGTTGATCCGAAGAACAGACCCTATAAGA
ACATTAGAATCAAGCACACACATATACTTGATGATCCATTTGATGATCCCCCACAGCTGGCTGAA
ATGATGCCAGATGCTTCCCCTGAAGGAAAACCGAAAGAAGAGGTCAAAGATGACGTGCGGCTTG
AAGATGATTGGGTTCCAATGGATGAAGAACTTGGTGCTCAGGAACTGGAGGAGGTTATCCGCGA
AAAGGCAGCCCATTCAAGTGCTGTTGTACTTGAAAGTATAGGAGATATCCCCGAGGCTGAGGTA
AAACCTCCTGACAATGTGCTGTTCGTCTGCAAACTGAATCCAGTGACTGAGGATGAAGACCTCCA
TACCATTTTTTCACGCTTTGGAACCGTTGTATCGGCTGATGTAATCCGGGATTTCAAGACAGGTGA
CAGTTTGTGCTATGCTTTTATAGAGTTTGAGAACAAGGAGTCATGCGAACAAGCGTACTTTAAGA
TGGACAATGCTCTGATCGATGATAGACGGATACATGTGGATTTCAGTCAGAGTGTGTCCAAACTT
TGGTCACAGTTCAGGCAAAAAGACTCCCAAAAGGGCAAAGGGAATGGGTGTTTCAAATGCGGCT
CGACGGATCATATTGCCAAGGACTGTGTGGGTGGTCCGTCTTCAAAGTTTATTGTAAAAGATCAA
AACAGACAGCACGGAGGAGGTGAAGGCTATGAGATGGTGTTTGAGGGTGATGTGCATGAAACC
CCCAAGCATAATAGTCATGAGAGGGAGAGGAGTGAGAAGATTCAGAGGAGAAGTCCTCATGGT
AATGGGGAAGGCAAAAGACAGCACAGAGATGAACGTGATGATGGTAGAAGACAGCATGATCG

GGAAGACGCACGAGAACTGGAGAGGAAACACAGAGAGAGAAAGAAAGAGAGAGTAGGGAA
GATGAAGACAGAAGAAGAAGAAGACGCCGTGAAGAGTCTAGAGATAAAGAGAGTCGGAGAGA
GAGAGACGAAGATGATCATCGGAGTCATAGAGATTATAAAGAGAGGAGAAGAGAGAGAGACG
ATAGACATGGAAGAGAAGCTAGACATGAGAGAAGGGATAGATGA

Protein
MSVLIVTSLGDIVIDLHSDKCPLTCKNFLKLCKIKYYNGCLFHTVQKDFTAQTGDPTGTGAGGDSIYKFL
YGEQARFYKDEIHLDLKHSKTGTVAMASGGENLNASQFYFTLRDDLDYLDGKHTVFGQIAEGFDTLTR
INEAYVDPKNRPYKNIRIKHTHILDDPFDDPPQLAEMMPDASPEGKPKEEVKDDVRLEDDWVPMDEEL
GAQELEEVIREKAAHSSAVVLESIGDIPEAEVKPPDNVLFVCKLNPVTEDEDLHTIFSRFGTVVSADVIRD
FKTGDSLCYAFIEFENKESCEQAYFKMDNALIDDRRIHVDFSQSVSKLWSQFRQKDSQKGKGNGCFKC
GSTDHIAKDCVGGPSSKFIVKDQNRQHGGGEGYEMVFEGDVHETPKHNSHERERSEKIQRRSPHGNGE
GKRQHRDERDDGRRQHDREDARELERKHRERKERESREDEDRRRRRRREESRDKESRRERDEDDHRSH
RDYKERRRERDDRHGREARHERRDR*

4. **AT1G68090** (ANN5)

Promoter (2 kb upstream)
CGCCAGAGACTTTCTCTGATGAATTTACAATTGCATCAGAAGAAGATTGTTGGTCCAAGTTGTTAT
GATCGCCTTCATCCTTCAGTTTCTTACTGCCTGCATGGTGGTGGTGGTGATGATGACCCCAAGTAT
TGGACCCAGATGAGTTTTCTTCGACATACCGCACCAACTTCTCCACAAGAAGGAAGACCACAAT
CCCAGCTGGATAATTAAGAATGATGATTGAAAGTGTCAATTCTGTCAAAATATTTTGTTCAATAA
ACACAAGAGGAAGTGTTATGATACTTACCAAGAACAGACAATCCAACAGACAAATCTTGTATAG
AATGTGAGTGTGAAGGCGAATCCGAATGAGAATGATCATGATGGTCATGGTTCTCATGGTGATCA
TTAGAGTGAGAGTGGCCACCACCTGCCAAACCATAAATATATTTTGCACTTCAGGAAGATATAA
AAGCAAACAAAATGAGAAGGCATAAAAGCCAAATTATGAAACAATGCAGATACTAAAAAAGA
GATCAGAAACTTGTGTCCATTACTGTACCAAAAGCATGGGGCAGTTGGTGAAGAAAAGCATCTC
CCAACATACTGCCTGGTAAACAAATTAACTGAATCAACAGATCATATATTATTAACATTTCCAAC
TCATCCAAGTGATAAAGTTGCGACGAAACACAGTGTATAGATATTGCTAAAATATTTTTCTTGCA
TTCAGAACCACCACCGCCAAAGAAAAAGCTACTGTAGTCAGTCTAATGACTTACCCCAAAGAGA
GCCAAAGAATCAACGAACCATTTTGATGGCTTCCCTTGAACTGTTAGTTGCAACATAAGACTTGA
AACAGTTACTCTCATGAACACATTATAGCACATGAATAAACGAAAGAGAGCATTTTCTCCAGGA
CAATTAAAAGCAATGCTTTTTGGTTTTCATAGGTATACAAAAAATTGCTTACCAAACATAATGGG
AAGCAAAACAAGGCAGATGAGTGAGGCCAAGCTAACTAAAAGAGAGCATCCCAATGCATTAAG
CCACAGAGCTACAAAAACCAACAAGAAGAAGAGATTCTAATCATCAAGTAATGATTTGTAAAA
CCCAATGAAGAATGTAGCTAAAGGAACCAAATTTACCAAACCCCGTAAGAGTGGAACTTGATTC
GTGATCGTGATCATGAAGACAAGGCCCAAACCCACATAATCTCATATCCTCCTCTTCAGCAAGCT
CCTCCGGCAACTTCATCTCCACCTTCGCCGTCGTCTTCTTCACATGATGATCATGATCGTGGTCGT
GGTCGTGGTCATGAGAATGACTACACCCACCACCGTGATGATGCACGTGATCGTCACGCGCCGG
CGTTGACTGAGAGAATCCGGATTCAACGCACAGATCCAGAAACAGTACCAAAACCAGAATCGG
AACCAGTAGCTTTCTCAGCGAGAACGACATCTCTGAGTGAGCTAAGAAGAGCGACGGTTGAAGC
GAGTGCCTTCAATAGTGATGAAGCAGACGATGCAGAAGAAGAGAATGATAATTCCGATTATGAT
TCCGAAAGTGACAACATCCATTTGGGGAAAGTAATAAACCTTAGGAGATCTTTTTATAATTTGTA
GTTTTGGCCCATTGTTTCGAAATTAAGTCGAAAACCCCCTTTTACTAGGCTACAGACTCTACACAA
CTCTTATAAAAGTCTTTATGTTGATGATAATCATGAAACATAGAGATATCTATCGGATCCTAAAA
AATGTTATCCGTTAAATTTTATGCATCAAAGCATCCATCGTTACACGAATGAAACAGAGGATTCA

ACTCCTAAAATCAAGGTCTAATGTCCATTTCCTTTGCATATTTCAACCCAAAACCCAATCGCCAC
AAATCACACCACACCAACAAAACAAAAGAAAAAACAAAATCAAGAGAATACAACAAAAAAAA
GAAAGAAGAAAGGTATAGAGTTTTACAATATCTTCTTCGGAATCTTTAGAGGAAGAAAGAAAAA

CDS
ATGGCAACAATGAAAATACCAATGACGGTACCTTCTCCTCGAGTCGATGCTGACCAACTCTTTAA
GGCCTTCAAAGGAAGAGGCTGCGATACTTCGGTGATCATCAACATCTTAGCTCATCGCAATGCAA
CACAACGAGCTCTCATCGAACAAGAATACGAAACCAAATTCTCGGATGACCTCCGAAAACGTCT
CCACTCTGAGCTTCATGGTCATCTCAAGAAAGCCGTTCTTTTGTGGATGCCTGAAGCAGTGGAGC
GAGACGCTTCAATACTGAAACGCTCCTTAAGAGGAGCCGTGACTGATCATAAAGCGATTGCTGA
GATTATATGCACACGATCTGGCTCTCAGCTTCGTCAGATCAAACAGGTCTACTCAAACACTTTCG
GTGTGAAACTTGAAGAGGACATCGAATCCGAAGCTTCTGGCAATCACAAAAGAGTTTTGCTCGC
GTATTTGAACACTACGCGATATGAAGGACCAGAGATCGATAATGCGAGTGTAGAGAACGATGCT
AGGACTCTCAAGAGCGCGGTTGCAAGGAAGCATAAATCTGATGACCAGACGTTGATTCAGATAT
TCACTGACCGAAGCAGGACTCATTTGGTCGCTGTAAGATCTACTTACCGTTCCATGTACGGCAAA
GAACTTGGAAAGGCCATAAGAGATGAGACTCGCGGGAACTTCGAGCATGTCCTTCTAACAATTTT
ACAATGTGCTGAAAACTCTTGTTTCTATTTCGCAAAGGCATTGAGGAAATCAATGAAAGGATTAG
GAACAGATGACACGGCGTTGATAAGAATCGTGGTGACGAGAGCAGAGGTGGATATGCAGTTCAT
CATCACAGAATACCGTAAGAGATACAAGAAGACTTTGTACAATGCTGTTCATTCTGATACAACTA
GTCATTACAGGACTTTTCTCCTCTCTCTTTTAGGCCCCAACGTTTGA

Protein
MATMKIPMTVPSPRVDADQLFKAFKGRGCDTSVIINILAHRNATQRALIEQEYETKFSDDLRKRLHSEL
HGHLKKAVLLWMPEAVERDASILKRSLRGAVTDHKAIAEIICTRSGSQLRQIKQVYSNTFGVKLEEDIES
EASGNHKRVLLAYLNTTRYEGPEIDNASVENDARTLKSAVARKHKSDDQTLIQIFTDRSRTHLVAVRST
YRSMYGKELGKAIRDETRGNFEHVLLTILQCAENSCFYFAKALRKSMKGLGTDDTALIRIVVTRAEVDM
QFIITEYRKRYKKTLYNAVHSDTTSHYRTFLLSLLGPNV*

5. **AT4G11640** (SR)

Promoter (2 kb upstream)
TTGTCGATTCCTCTTGCGGAGAGATGCAATCGATTCGTGGCAGTACACGGTTGCTTCAGAGGCAT
CGTTTCTCCATCTCCAACACTCTTCGTTTCACTTCTTCTTCTTCTTGTGGAAGCTGGTCTGAAAATG
AATCCAGAAAACTAGGGTTTGATTCTTGCCCTTCTAGCAGAGCTGCGACATATCTCATTAGCTCGT
TGAAAAATGCAGGCTTTTCCTCTTCAGCTATACCTGTAAGCCACTTTCGTTCTCTATCTTGGTTTAA
CATTGTCGAAATGTAGAAGAGGTTTGGTTGGTAATGGAGATCTTATATCAAATGGGTTTGAAAAT
TGCAATTAGTGTAGAAAAACGCAAACTTTGCTCTTGTGTGGTAGTAAGAGTTATGTAGATATAGA
AGAACCAGCTTTGACACAAGTTTTATGATTTCACAAGTTGTTCTACTTTATATGTTTGGGTCACAG
TGATATCTATCTAGTATGTTAATGTACATTAGGTTTCTACGTGTTTACGATGTTCTTGGAAATATTT
TGGCAGAAACAAAACTTTACGTATGCGTCGAGGTGTTTTTCCACCACTGGAGATTCTTCTGTTCAA
GGATCTCCTGAGTCAGATTGTGTTGTGCCTCAGAGAATCAAGTTTAAGCGGCTTGATAAAACCGC
TAAGCATATAATGCAGGTACTACAACTCAATTACTAGACAACTGTCATGGATAAATCATGTCATA
TGACACAATCTTCTGCAGATTGTAGACAAGGAAGCGGTTGAGGAAGTGAGATCTCTTAGAGAGA
TTCCTGAGATAAAGCCCGGTTACATTGTGCAGCTAAAAGTGGTAATTTCTTTTTTCTTGGTGAATG
GGATTGAACTATCTTTGTCTTTAAAGTGAAACCTTTTCATAGGCTTGCCATTTACTTTTGTTTTTAA
CAGGAAGTGCCTGAGAACAAGAGACGTGTATCAATCGTGAAAGGCGTCGTCATAGCAAGGCGT

AATGCTGGTCTGAACTCAACATTTAGAATTAGAAGGCTTGTTGCTGGAGTGGGCGTTGAATCCAT
GTTCCCCTTGTAAGTAACTTGAAGCTCAAACTGCAACTACTTATTCTTTTTTTCTTTCTCTCAGAAT
TTGTATATGATATGATAACTCAAGCCACCATGTTTTGTGTGCCTTAAATTGATGTGAGTGAGGGTG
TTTGGTGACAGGTATTCCCCAAACCTGAGGGAGATTAAGGTGGTGGACAAGAAGAAAGTAAGAA
GAGCCAAGCTATATTACCTCAGGGACAAGGTGAATGCTCTTAAGAAGCATTAACAACCTCAGTA
AAAAAAACAATCTACTCGTGTCTTAAACAATTTTCCAATGTTTTAGTTGCACTCTCTCAAAAGGA
GACGATTTGCCTCAAATTTCACGTATTGAATTTGTGCAATGGTGAATCTTCTTCAATTCTCTTCTCT
CCCTTGTTGGAAAAAGCACATGGGCTAAAGACATGTTATAGTTTTATATATGGAGACAAGTAATC
CACGACGTTTCTTGTATAATAAAACAGCGTGACGTTCTTTTTTTTAGCTCCATAACACAACGCTGT
CTTTGAGTAAATGTATCTGTCCTCTCGAATTCTGCAATTTCCTGAAACTGGGCAAATTGAAATTCC
CAATCCTAAACCCTAAGCTCAAATTTGAGAAATTAGGTTTGTTTTCATCCTTTCTTTGTCTCAGTCA
CTTAGTTTTTGGGTTTTAGTAGACTCCTTTGTATTTTTTAACCCAAAAATTGCAATGTCAAAAGTTT
CAGTCTTTTGCAACTTCTGCTGTGTTCTTTTGTGATTTGAGACAGAAGAGAAACTTAATTTGTCTTT
AAAGCTAACATTTTTAGGTAGCAAAAATTAGTATATTGAGCATAAAGTTAACAAATTTGAAGTTA
TTTAAGATGATTGAGGATATGGTTTTCTATTCTGATAAGTAGGAAGTTGATGAAGTGATAATGAA
GCTTCTTTGAAATGTTTTTACTTTTTAGAA

CDS
ATGGAAGCAAATAGAGAGAAGTATGCTGCTGATATATTGTCCATAAAAGAAGCTCATGACCGTA
TTAAGCCGTATATACATAGAACTCCGGTGTTAACATCTGAATCGTTGAATTCAATCTCCGGAAGA
AGCTTGTTCTTTAAGTGTGAGTGCCTTCAGAAAGGTGGAGCTTTCAAATTCCGTGGAGCTTGCAAT
GCTGTTTTGTCTCTTGATGCTGAACAAGCAGCCAAAGGAGTTGTAACACACAGCAGTGGAAACC
ATGCTGCTGCATTGTCTTTGGCTGCAAAGATTCAGGGAATACCGGCATATATTGTTGTACCAAAA
GGTGCTCCAAAGTGCAAAGTTGATAATGTGATCAGGTATGGTGGTAAGGTTATATGGAGTGAAG
CAACAATGTCTTCTAGAGAGGAAATAGCTTCAAAAGTTTTGCAGGAAACAGGTTCAGTTCTGATC
CATCCATATAATGATGGACGCATTATAAGTGGTCAGGGTACAATTGCATTGGAACTGCTGGAGCA
AATCCAAGAGATTGATGCTATAGTTGTGCCGATAAGCGGGGGTGGTTTGATATCTGGTGTGGCGC
TTGCTGCCAAATCTATTAAGCCTAGTATCCGGATTATAGCCGCTGAACCAAAAGGAGCTGATGAT
GCGGCTCAGTCTAAGGTCGCTGGTAAAATCATCACTTTACCTGTGACTAATACTATAGCTGATGG
CCTTCGAGCTTCTCTTGGAGATTTAACCTGGCCGGTGGTGAGGGACTTGGTAGATGATGTTGTCAC
CTTGGAAGAGTGTGAAATAATCGAAGCCATGAAAATGTGCTATGAGATACTGAAGGTCTCTGTG
GAACCAAGTGGAGCTATTGGCCTTGCAGCAGTTTTATCAAACAGTTTCCGCAACAATCCTTCTTGT
CGTGATTGCAAAAACATAGGAATTGTACTCTCAGGAGGTAATGTTGATTTGGGCTCTCTATGGGA
TTCATTTAAGAGTTCAAAATAA

Protein
MEANREKYAADILSIKEAHDRIKPYIHRTPVLTSESLNSISGRSLFFKCECLQKGGAFKFRGACNAVLSLD
AEQAAKGVVTHSSGNHAAALSLAAKIQGIPAYIVVPKGAPKCKVDNVIRYGGKVIWSEATMSSREEIAS
KVLQETGSVLIHPYNDGRIISGQGTIALELLEQIQEIDAIVVPISGGGLISGVALAAKSIKPSIRIIAAEPKGA
DDAAQSKVAGKIITLPVTNTIADGLRASLGDLTWPVVRDLVDDVVTLEECEIIEAMKMCYEILKVSVEPS
GAIGLAAVLSNSFRNNPSCRDCKNIGIVLSGGNVDLGSLWDSFKSSK*

6. **AT5G50320** (ELO3)

Promoter (2 kb upstream)

GTCCTTTTTTCTTTTGTTGTTGTTGTTGTTATATTGGCTTGCATTATGAAATATACTGACACCACACT
GAATCTATATCTCCTCTTTCTTCAGCAATATACTTAACAGTTTATCCCGTGCGTATGGCCAAATGA
TACTGAAAAGTGGTTTCTTTCATGCGGATCCACATCCTGGAAATATCTTGATTTGCAAAGGTCAA
GAGGCAAGTCATATTTTGCTTGTAGTCATTATTTGGTAGTTTTTTAATTCGCTTAGTGTTACCTTCTG
GCAGAACTTGCCCGTCTATATAGTCTGAGTTGTAATCTTGTTTCCCGTCATTAGGTTGCATTGCTGG
ACTATGGTCAAGTGAAGGAACTACCCAACAAGTTGAGACTTGGTTATGCAAATTTGGTCATTGCC
ATGGCTGATAACAATGCTTCACGAGTATCACAGAGCTTTTGGTAACTCAAAACTCATTAAATCTC
CATGCACTACTGAAGTAACTTTGTGATCTAAATTGTTCGTTTTTGTTCATATCATCTGTTTTGTGGC
TCAAAAGCATTTTTGTCATTAGCAAATCTTTTTCTTTTGAGAAAGATATTCCAATAAGAACCAGTC
CATAAAAGGTGAAACCATTTGTTACCACAAAAGTTATTTGTGAGCGAGGATTTCATTTTGTTTAG
GCTATGAAGATTAAGTACTTGATCATTATGTTCAAAAAACGGCTTTTCCACTTTTAAATTGAATAG
TTTAGTATGTGAGAACTGTTTTGAGGCAAGTATGTGGTGGATTATTATGATTGAAAGTGATAAGA
AAACGTTTTTTTTTGTCCAATATTGGAATATTCACTGTCAACAGTTTGGATTACTGCAGCACTAAA
TTCCTTAAGTTATTGTGGCTGTGAATGTTCAGGGAAATGGGTTTACATACAGTGGCCAAGTGTGA
GAATGAGCAGCAAGAACTTCTGCGGTTAGCACAAACACTTTTTGATACAAAGATGCCTACTGGTC
AGACAGTTTTACAACCTTTCTCAGATGACTCTTCGATTAAAAAGATAGCCGTTGAGGTAAGTAAA
GCTCCATTGATGGTCACAAAGTAAAAAAAAGTTGTGTGTTCTACAGGTAATAATATATACCAAAC
TAACTTGAATGTGTTCTTTGTCGGTAGACATTTCCAGAGGAACTGTTCTCTGTACTTCGCACTGTG
GTTCTGTTAAGAGGACTCAGTGTTGGGATGGGAGTTAATTATTCGTGTGCAGAACAATGGAGAGC
AATGGCCGAAGAGGCTTTACTTGCATCTGGAAGAGTGACAAGAGGTAAGTGGTTATATCGTATA
GTACTCACTACACTTTTTGCATAAAAAACAAGTGCTGATGGAGTTGGTAACATAACACCTGAGTT
TTACAGATGGCAAAGAAAGATCTCGTAGACGGGCTTCTCTACGAAGGCTACGCACAGGAAGTTA
GGAAAAAGATAGGAGTATTTCTCATCACTTAGACTCTCCCATGAGCAGGATCTGAGCTTTTTTCAT
TTATTGTTCTTTCTTTTGTGAGAATTTGAATGATAGGAGAAGTTGGATAACACTTGAATGTAATTG
GCTGATATAAAATTATGATGGAAGATTTGTAAATCAAGATTTAATTCCCAAAAGATAGGATTATA
TGTTTTGGTACTCCTTCTCCACAATAGTTGGAGAGGACTATTATCTCCAACAGCTATAAGCCATCT
TGGCTATTACGTCGGCACATATATATGCCATTACGATTCATGGAGTCATTCTCAAAGATGACTGC
CTCAAAACCCAAGTTATCACTGCACATCATTGTCTAACACAAAAGGCTGGTGCTTCAGCTTCTTA
AGGAGACTGAATCTTCATATAGATTTTGGTTCCACACCATTTTATGTCTTTTCTGTTGCCTCAAAAA
AAAAAATTGAAGCGTTGACAAAAAAAAAAAGTTAAACAGATTAAAACGATGTCGTTTAAAGGAT
TTCATTAACACAAAATCTGCGTTCAACGAGTTC

CDS

ATGGCGACGGCGGTAGTGATGAACGGCGAGCTGAAAAAGCAACCTCGGCCAGGTAAAGGCGGC
TATCAGGGCCGTGGATTAACTGAAGAAGAAGCTCGAGTTCGCGCCATATCGGAGATTGTTAGCA
CCATGATTGAGCGTTCACACCGCAACGAGAATGTTGACCTAAACGCAATTAAAACCGCCGCTTG
CCGGAAATACGGCCTAGCACGTGCGCCTAAGCTCGTGGAGATGATTGCTGCGCTTCCTGATTCAG
AGAGAGAGACTCTTCTCCCGAAGCTCCGTGCCAAACCGGTTCGAACAGCTTCAGGGATCGCCGTT
GTGGCGGTTATGTCGAAGCCTCATAGGTGCCCGCATATAGCTACGACGGGGAATATATGCGTTTA
TTGTCCCGGTGGACCTGACTCTGACTTTGAGTATAGTACTCAGTCTTACACTGGATATGAGCCTAC
CAGCATGCGAGCTATTCGAGCCAGGTACAATCCATATGTTCAGGCAAGAAGCAGGATAGATCAG
CTGAAGCGGTTGGGTCACAGTGTAGATAAGGTTGAGTTCATTTTGATGGGAGGTACTTTCATGTC
ACTGCCTGCTGAGTATCGGGATTTCTTCATACGGAATCTTCATGATGCTTTATCAGGACACACTTC
TGCCAACGTTGAAGAGGCAGTTGCTTACTCTGAACATAGTGCAACTAAATGCATTGGGATGACA
ATTGAAACGAGGCCAGATTACTGCCTTGGACCTCATTTACGACAAATGCTGATTTACGGTTGCAC

CCGGCTAGAGATAGGTGTCCAGAGCACATATGAAGATGTTGCCCGTGACACAAATAGAGGTCAT
ACTGTTGCTGCTGTAGCTGACTGCTTCTGCTTGGCTAAAGATGCTGGTTTCAAGGTGGTTGCACAT
ATGATGCCTGATCTTCCTAATGTTGGGGTTGAGAGAGACATGGAAAGTTTCAAGGAGTTTTTCGA
GAGCCCATCTTTTAGAGCAGATGGGTTAAAAATATATCCCACCCTTGTGATCCGTGGAACTGGAC
TTTATGAACTATGGAAAACTGGGAGGTACCGAAATTATCCACCTGAGCAGCTTGTGGATATAGTT
GCAAGGATTCTCTCCATGGTACCTCCATGGACACGTGTATATAGAGTTCAGCGTGATATTCCTATG
CCTCTGGTTACGTCAGGGGTAGAAAAAGGAAATCTTCGTGAACTGGCTCTAGCCAGAATGGATG
ACTTGGGCCTTAAATGCCGTGATGTCCGTACTCGTGAAGCTGGAATTCAGGACATTCATCATAAA
ATTAAGCCAGAACAAGTAGAGCTTGTGCGTCGTGATTACACTGCCAATGAAGGTTGGGAGACGT
TCCTTTCTTATGAAGATACACGCCAGGACATTCTTGTTGGATTGCTACGTTTGCGAAAATGCGGGA
AGAATGTAACGTGTCCAGAACTCATGGGAAAGTGTTCTGTTGTCCGTGAGCTTCATGTATACGGA
ACAGCTGTACCAGTTCATGGTCGAGATGCTGATAAGTTGCAACATCAGGGCTATGGTACACTTCT
GATGGAAGAAGCAGAGAGGATTGCTAGAAGAGAACATCGATCTAACAAAATCGGTGTGATTTCT
GGTGTAGGAACCAGACATTACTACAGAAAGTTGGGTTATGAATTGGAAGGTCCTTACATGGTGA
AGCATCTTCTTTGA

Protein
MATAVVMNGELKKQPRPGKGGYQGRGLTEEEARVRAISEIVSTMIERSHRNENVDLNAIKTAACRKYG
LARAPKLVEMIAALPDSERETLLPKLRAKPVRTASGIAVVAVMSKPHRCPHIATTGNICVYCPGGPDSD
FEYSTQSYTGYEPTSMRAIRARYNPYVQARSRIDQLKRLGHSVDKVEFILMGGTFMSLPAEYRDFFIRNL
HDALSGHTSANVEEAVAYSEHSATKCIGMTIETRPDYCLGPHLRQMLIYGCTRLEIGVQSTYEDVARDT
NRGHTVAAVADCFCLAKDAGFKVVAHMMPDLPNVGVERDMESFKEFFESPSFRADGLKIYPTLVIRGT
GLYELWKTGRYRNYPPEQLVDIVARILSMVPPWTRVYRVQRDIPMPLVTSGVEKGNLRELALARMDDL
GLKCRDVRTREAGIQDIHHKIKPEQVELVRRDYTANEGWETFLSYEDTRQDILVGLLRLRKCGKNVTCP
ELMGKCSVVRELHVYGTAVPVHGRDADKLQHQGYGTLLMEEAERIARREHRSNKIGVISGVGTRHYY
RKLGYELEGPYMVKHLL*

7. **AT3G49050**

Promoter (2 kb upstream)
CAAAACTTCACTTTTAGGTACTTTGTTGATCTAATATTACATAAATTGCACTGTTCTCAGCTAAAT
ATGTCTTCTACCAAAGGAAATCAGATAGGCCAAAAATGGATCCAGCCTAAATGTGTACCGGAAT
GTTTGTTATTCCATCTTGAGACATTCGTGTGGACAGGATACGAATGGCAACGAAGAGATGAGAA
AGAAGTGGCGAGATACATCCTAAGAAACACAACAAGTTTGAAGAAAGCAACTTTCTCCACAAA
ACCCATCGAACCGGTAAAGCTCAAAGTTTTTAAAAAGAGACGTGAGATCCTCAACGAGTTGGCT
AGTTTGGGCAGGGCTTCAAATCCAAGTGATTTTGTGTTTGAATCTATTTAATCTCTCGAGCATATA
TATATTATATATGGTTTGTTGACCATTGGGAAATGTAAAACTCTCAACTTTATCAATCGATAGGAT
GCCAAATCTTGGTTACATTTTGAACTTATACTATTGTAGATATATATTTTTCTAACAAAAGCTGAA
CTTAGGTTTTGAATAGTATGGACTTTCTAGAAGAAATGATATGAATTGATTGCACATAGGCTTTTT
CTAACAAAAGCTGAATTGGCAATTTGCTACTTAAATGAATTTGATTGCATAATTTATTGTAGTGCT
TGAAAATTCTGAACTTGCAAGTTCTGTAATAGGAAACACATCACAAAATAATTGCAATTGGTCCC
AAAGGCGTATAAACTTGTTTTCCTTTCCATGACTCTTTTGACCTCTAGGTAATAGTAAAGCCAGTA
GATGGAATTAGCAGCTACCAACCTAACTTAAATGAATTTACAGTTGCTATATATGATTTTCTTTTT
AATATATAATACTAAATTAGAACCAGCCAAAGAAAATTAATTGAGAATTATTCAAAAGCCATTG
TGATTTTCTGTTTGTCAAGAAAAAAACTACTTCCTTAATTTTTTTCCTACATAAAAATTTAGATTG

AAATCTACATTAATGTTCAGAATTTACAGTTACAAACAGTTACAGTAGAAACTCTATAAATTAAT
ATTCGGTAAATTAATAAACACGATAAATTAATAAATTATGTCGATTCCAAGTCGGGTCAATGTAA
AAATTAACCAAAATCGATAAGATAATAAGATAATAATTTTTTTTGAAATCTCTTTGTCAAAATAT
GGTCTCAATAATATCATAAATCAATAATTACTAATCTTAAACATACTTTTAAAGGTAATCTAGTAC
AATATGACTTTAGTTATATTTGTTTTTTCTTAAAGTTTGAATCTTTCTGAAGTTCATCTCTAATTTTC
TTTATTGCATCAAAAACTCTTGGTGTTGTCTTTTTGATATGCATCCAAAAGTTGTGAAGAGTTTTAG
ACGCGATAATTGCTTCCTTACGTGTCAATATTTAAGAAAGTTTCACATTATCGTCTTCAAGTTCTTC
ACCAACATTATAAACAGAAATCACAATTTCTTCGAAAAACTCTGAATTTTTGAGTATGCTTCATTT
GCACCTAGATATTCGAGTAGGCTATTGACATTTATTCTATTCCGAAAGAAGATAGTGTTATATTTT
GCTTAAATATTTCTATAAATTAATAAATATTAATTTATCCTGTAAAATAATATTTATTAATTTATAG
AATAAATTAATTTCACTATAAATTAAGAAAATTTCATGGTCCCAACATTATTAATTTATAGAATTT
CTACTGTATATGGAATCTATTCAATAAAATAATATGACTAATGAAAATCAACCACTATTTATTTTG
ACTAAACAAGTTACACAACTACTACAACCACCAAATTCTCATTTTTAATTGTAATTGTGTAATGTT
GATGAACTTTTTCAGTCTAAATCTAGAAACTTGGGACAAATTAAACAAAAATTAACACTAATTAT
CTCGAAATTTAACTCTTAAATTAATAACCAAAATTTTGTAAGAAAATAGTATTATTTATATTTTAT
CTTCTTCACACAACACAACACAGCAAGCTC

CDS

ATGTCGATTCTATGTGGTTGTTGCCCTCTTTTAGAATGTGTTTACTGTCTTGGTTGTGCACGTTGGG
GTTATAAACGTTGTCTTTATACTGCTGGTCACGACAGTGAAGATTGGGGACTTGCAACAACTGAT
GAATTCGAACCGGTTCCTCGGTTTTGTCGTTATATTTTAGCTGTTTACGAGGATGATATTCGAAAT
CCTTTGTGGGAGCCTCCAGAAGGATATGGGATTAACCCTGATTGGTTGCTTCTAAAGAAGACTTA
TGAAGACACTCAGGGTCGTGCTCCAGCTTATATATTGTATCTTGATCATGTTCATCAAGATATAGT
TGTTGCAATCCGGGGGTTGAATTTGGCTAAGGAGAGTGATTATGCAATGCTTTTGGATAATAAGC
TTGGTGAAAGGAAGTTTGATGGTGGTTATGTGCACAACGGGCTTGTGAAGTCTGCTGGTTATGTGT
TAGATGAGGAATGTAAAGTTTTGAAAGAGTTGGTGAAAAAGTATCCGAGTTATACTCTGACTTTT
GCTGGACATTCACTTGGGTCTGGTGTAGCTACAATGTTGGCTTTGTTAGTGGTTCGACATCCGGAA
AGATTGGGGAATATTGATAGAAAGAGAGTTAGGTGTTTCGCTATTGCGCCTGCGAGGTGTATGTC
GTTGAATTTGGCTGTTAGATATGCAGATGTGATTAACTCTGTCATACTTCAGGATGATTTCTTGCCT
AGGACAGCTACACCTCTAGAAGACATATTCAAGTCTGTATTCTGTTTGCCATGTTTGCTATGCATA
AGGTGCATGAAGGATACATGTGTTCCAGAGCAAAAGATGCTCAAAGATCCTAGACGGCTTTATG
CTCCTGGTCGCATGTATCACATCGTCGAAAGAAAGCCTTGCAGATTAGGAAGATATCCTCCGGTT
GTGAAGACAGCAGTGCCGGTAGATGGAAGATTCGAACACATTGTTCTTTCTTGTAACGCAACTTC
AGACCACGCCATCATCTGGATCGAACGAGAAGCTCAGAGAGCTCTCAACCTAATGATGGAGAAT
GAGAAGAAAATGGAGATCCCGGAGAAGCAAAGGATGGAGAGGCAAGAATCATTAGCCAGAGA
GCACAACTTGGAGTACAGAGCAGCGTTAAGACGAGCAGTAACGTTAGATGTACCTCACGCAGAG
TCAATGGCCTATGAGTACGGAACATTCGACAAAACTCAAGAAGACGAAACCGAAGAAGAAGAA
GTAGAAACAGAGGAAGAAGAAGAAGACACAGACTCGATTGCACCAATGGTGGGTGAATCATCA
TCATCATCTTCTGTTAAACCAACTTACAGGATAAGACGAAACCGTAGAGTTAGTTGGGACGAACT
AATCGAACATCTCTTTGAACGAGACGAGTCTGGTAACTTGACATTCGAGAAATCAGATTTACCTC
AATGA

Protein

MSILCGCCPLLECVYCLGCARWGYKRCLYTAGHDSEDWGLATTDEFEPVPRFCRYILAVYEDDIRNPL
WEPPEGYGINPDWLLLKKTYEDTQGRAPAYILYLDHVHQDIVVAIRGLNLAKESDYAMLLDNKLGER

KFDGGYVHNGLVKSAGYVLDEECKVLKELVKKYPSYTLTFAGHSLGSGVATMLALLVVRHPERLGNID
RKRVRCFAIAPARCMSLNLAVRYADVINSVILQDDFLPRTATPLEDIFKSVFCLPCLLCIRCMKDTCVPE
QKMLKDPRRLYAPGRMYHIVERKPCRLGRYPPVVKTAVPVDGRFEHIVLSCNATSDHAIIWIEREAQRA
LNLMMENEKKMEIPEKQRMERQESLAREHNLEYRAALRRAVTLDVPHAESMAYEYGTFDKTQEDETE
EEEVETEEEEEDTDSIAPMVGESSSSSSVKPTYRIRRNRRVSWDELIEHLFERDESGNLTFEKSDLPQ*

8. **AT4G21390** (B120)

Promoter (2 kb upstream)
GAGGTTTGAGTTTGATTCATCATGTTAACTGAATTTGTAATTCTGTTTGTTTCTCAGGTGTGGAGAT
GTTGGAGAGAAGACATTATACTAAGCGTTATTGATCCGAGTCTAACCACGGGATCAAGAAGCGA
GATCTTGAGATGCATACACATTGGTCTTTTATGTGTTCAAGAAAGTCCAGCGAGTAGACCAACTA
TGGATTCGGTTGCTCTAATGCTCAATAGCTATTCCTATACTCTCCCAACGCCTTCAAGGCCCGCCT
TTGCGTTAGAGAGTGTCATGCCTTCGATGAATGTTTCTTCTTCCACAGAACCGTTATTAATGTCCTT
GAATGATGTCACTGTTTCTGAGTTATCTCCTCGTTAGGAAGCTTAATCTGCATATCTTTTTAGTTTC
TTCAATGATGGATTTTGTTTGTGTGATTTTTTAGACAGCTTGGCTTCATCTAGCTTTTGAAACGAGT
AGAAGTTATAGCTTTTTCTTTTAGTTCTACACATTCGTCGTTAATGGTAGATTTGTTTTGCAAATTC
GATTTTACTGTGTTTTAAAACATATAACATGAAATTTAACTTAAGAAAATGAAAATAGTATTAGA
CTTTTACTAAGAAAAGCTTTCTACTTTATCCTACACATGAAATGCATACACATTGGTCTTTTCAAA
AGCAACAAATCAATGTTAAATACAGTTTTCTTTATCTTAACTCCTAATAATGTAGCTATAGAATTC
ATGACTAAGCATTGTGGACCCTTGGAGCATTTTTAGATGGAGATGGGTGTTTGTTTCTTTATTAGG
TTTTGTTGCCTAGTAGCCACATTCTCTCAGTTAAACTAATACATTTGTAAATTTTCACTTGTAATCA
ACTAATCCACCAAATGAATCAATAATGTAAACTCCAGAATCGATCTGGCATTGTATGAGTATCAA
TATATGCTTTAGTGCCACTTGCTAGTGCAATCCACTTGAGGTTTGGCTGCATCGATATTAGATATA
AATACTTAAGCGTCATGCAAAAAAAAGATTACTAAATAATTGTAGGGTTATTATGGGATTTTAGG
CGAGCAAACAACATTTAAACTTTGAAGGAATACAATTTGAAATATCAAGATACGTAAAATATGT
CACGTGCAATAAAATAAAGAGAAGACCCATCCAAATCAAGTGTGCGATACTTTGACCATATGAG
TCAATGTGGCGGCCAAAGGCAACAAGCATATCTAATCTAAGTATTATATAACTAAGCAAATATTC
CACTAAACTAGTATACAAAGCCAACTGCTCCACTAATTGCCTGACATACTTATTTATTTCAAATT
TGGTAACCACACCTATAGCTTATACATTTTCTTCGATGGCCTTATACAATAGGAATATACTATTAC
CTTCTCATTGTTCTTATTATCATCAACCATTTGATAAATCCTCATCACTCTTAAACATTGACTATGA
ACTAAGGACGTCAGAAGTAGTCCATACAAGATATGAATGGTTGGATCGTCCTAAGTCATTGTATT
AATATACGTTTCTAATCAATGGAAACTATATAATTGTAATATAATTTTTACTAAATCATGTAACTT
GAAAACCTAACTTTCTTTCTTAATAAAAATTGAACCGCTAAAGTATCTAATCCATCATGTGTCAA
CTGACACCGTCCAAAATCCTCTTAGAGATGTAAGAAAAAGTTTCAAAACAATTAGGTCAGCCAC
CAATCACATATTTCTATGCAGGTTGTATAATCTTGAAAAAGAACAAAAAAAGTAGATGACAAAA
AAGAATTAAAAGACAATAATAATAATACCTTATAAAGATGATAATAATTCAAACAGTTTGACCT
TTTTATTTCAATTCTCTGGTCCAACTTTCCAACCTGACGAGACAAAAATATAGAAACTTCTCAACA
GCAATAAAACAGCAACCTTTCCTTTTTGTATCCCTTCTCCTTTGTCACCTCTCCTCTTACTTTTTTAT
CAATAGGAAGTTTCCGCCATTGTGACAGAC

CDS
ATGAGATTCTTCCGCAAAACCTCACTTTATCTTTCTCTCTTCTTATACTTCTTTCTTTATGAATCTTC
CATGGCTGCAAATACAATCAGAAGAGGCGAATCTTTAAGAGACGGAATCAATCACAAACCTTTA
GTCTCTCCACAAAAGACATTCGAGCTAGGTTTCTTCAGTCCTGGAAGCTCTACGCATCGTTTCCTA
GGGATTTGGTATGGGAACATCGAGGATAAAGCTGTGGTATGGGTAGCAAACAGAGCAACTCCAA

TTTCAGACCAATCTGGAGTTCTCATGATAAGCAACGATGGTAATCTCGTGTTATTAGACGGGAAG
AACATCACTGTCTGGTCTTCAAACATCGAGTCAAGCACTACCAACAACAACAACAGAGTCGTTT
CTATACATGACACTGGGAACTTTGTTTTGTCTGAAACCGACACAGATAGACCTATTTGGGAGAGT
TTCAATCACCCAACAGATACCTTCTTACCGCAGATGAGAGTTCGTGTGAATCCACAAACCGGAG
ACAATCACGCTTTTGTCTCATGGAGATCTGAGACTGATCCTTCCCCTGGTAATTACTCATTAGGGG
TTGATCCCTCTGGAGCACCAGAGATTGTGCTTTGGGAAGGGAACAAGACGAGGAAATGGAGAAG
CGGGCAATGGAACTCTGCGATTTTTACCGGGATTCCGAATATGTCTCTGTTGACGAATTATCTTTA
CGGATTCAAGCTTTCATCTCCTCCAGATGAAACAGGTAGCGTGTACTTCACGTATGTTCCTTCAGA
TCCATCAGTGTTACTTAGGTTTAAGGTTCTTTATAATGGAACCGAAGAGGAATTGAGATGGAATG
AGACTTTGAAGAAGTGGACTAAGTTTCAGTCTGAGCCAGATAGTGAGTGTGATCAGTATAATCGT
TGTGGCAAGTTTGGTATTTGTGATATGAAGGGCTCTAATGGAATCTGTAGCTGTATTCATGGTTAT
GAGCAAGTTTCTGTTGGGAATTGGAGTAGAGGTTGTAGAAGAAGAACACCGTTGAAGTGTGAAA
GGAACATTAGTGTTGGAGAGGATGAGTTCTTGACTTTGAAATCTGTGAAGTTGCCTGATTTTGAA
ATTCCTGAACATAATCTTGTTGATCCTGAGGATTGCAGAGAGAGGTGTCTGAGGAATTGTTCTTGC
AATGCTTATTCTCTTGTTGGTGGTATTGGATGCATGATTTGGAACCAAGATTTAGTGGATTTACAA
CAGTTTGAAGCTGGTGGTTCTTCGCTTCATATCCGTCTTGCTGATTCCGAAGTAGGGGAGAACAG
GAAAACAAAGATTGCAGTGATTGTCGCGGTTCTTGTTGGTGTGATCTTGATAGGAATCTTTGCTCT
GCTTCTATGGAGATTCAAGAGAAAGAAAGATGTTTCAGGAGCATATTGTGGTAAGAACACCGAT
ACATCGGTTGTTGTTGCTGATCTGACCAAGAGCAAAGAAACTACTTCAGCGTTTTCAGGATCAGT
AGACATAATGATTGAAGGGAAAGCTGTTAACACATCCGAGTTACCTGTCTTTTCTCTGAATGCTA
TAGCCATAGCTACTAATGATTTCTGCAAAGAAAATGAGCTTGGAAGAGGTGGATTCGGACCAGT
CTACAAGGGTGTTCTCGAAGATGGACGTGAGATAGCTGTGAAGAGATTGTCTGGTAAATCAGGA
CAAGGAGTTGATGAATTCAAGAATGAGATTATTCTCATAGCGAAGCTTCAACATCGGAATCTTGT
GAGATTACTTGGATGTTGCTTCGAGGGAGAAGAGAAAATGCTTGTTTATGAGTATATGCCTAACA
AGAGCTTGGATTTCTTCCTCTTTGATGAAACGAAACAAGCGTTGATAGACTGGAAACTGCGGTTT
TCGATAATTGAAGGGATTGCGAGAGGATTGCTTTATCTTCATAGAGATTCAAGATTGAGAATCAT
TCATAGAGACTTGAAGGTTAGCAATGTGTTGTTAGATGCAGAGATGAATCCAAAGATCTCAGATT
TTGGTATGGCAAGGATCTTTGGTGGTAACCAAAATGAAGCTAACACGGTTCGCGTTGTTGGAACT
TACGGATACATGTCTCCTGAATACGCGATGGAAGGACTTTTCTCGGTGAAATCCGATGTTTACAG
CTTCGGTGTCTTGTTGCTAGAGATTGTAAGTGGGAAGAGGAACACAAGTCTTCGATCTTCTGAAC
ATGGAAGTCTCATTGGTTATGCTTGGTATTTGTATACTCATGGGAGATCAGAGGAGCTTGTGGATC
CTAAGATCAGGGTCACATGCAGTAAGCGAGAGGCCTTGAGATGCATACACGTAGCGATGCTATG
TGTTCAAGATTCGGCTGCGGAGAGACCCAACATGGCTTCAGTTTTGCTTATGTTAGAGAGTGACA
CAGCGACGCTCGCTGCGCCTAGACAACCAACGTTTACTTCCACCAGAAGAAACTCCATTGATGTC
AACTTTGCTCTTGATTCGAGCCAACAATACATCGTTTCTTCTAATGAGATCACTTCAACGGTTGTT
CTTGGACGATAA

Protein
MRFFRKTSLYLSLFLYFFLYESSMAANTIRRGESLRDGINHKPLVSPQKTFELGFFSPGSSTHRFLGIWYGN
IEDKAVVWVANRATPISDQSGVLMISNDGNLVLLDGKNITVWSSNIESSTTNNNNRVVSIHDTGNFVLS
ETDTDRPIWESFNHPTDTFLPQMRVRVNPQTGDNHAFVSWRSETDPSPGNYSLGVDPSGAPEIVLWEG
NKTRKWRSGQWNSAIFTGIPNMSLLTNYLYGFKLSSPPDETGSVYFTYVPSDPSVLLRFKVLYNGTEEEL
RWNETLKKWTKFQSEPDSECDQYNRCGKFGICDMKGSNGICSCIHGYEQVSVGNWSRGCRRRTPLKC
ERNISVGEDEFLTLKSVKLPDFEIPEHNLVDPEDCRERCLRNCSCNAYSLVGGIGCMIWNQDLVDLQQF

EAGGSSLHIRLADSEVGENRKTKIAVIVAVLVGVILIGIFALLLWRFKRKKDVSGAYCGKNTDTSVVVAD
LTKSKETTSAFSGSVDIMIEGKAVNTSELPVFSLNAIAIATNDFCKENELGRGGFGPVYKGVLEDGREIAV
KRLSGKSGQGVDEFKNEIILIAKLQHRNLVRLLGCCFEGEEKMLVYEYMPNKSLDFFLFDETKQALIDW
KLRFSIIEGIARGLLYLHRDSRLRIIHRDLKVSNVLLDAEMNPKISDFGMARIFGGNQNEANTVRVVGTY
GYMSPEYAMEGLFSVKSDVYSFGVLLLEIVSGKRNTSLRSSEHGSLIGYAWYLYTHGRSEELVDPKIRVTC
SKREALRCIHVAMLCVQDSAAERPNMASVLLMLESDTATLAAPRQPTFTSTRRNSIDVNFALDSSQQYI
VSSNEITSTVVLGR*

9. **AT2G43110**

Promoter (2 kb upstream)

GATCTTTTCCATGCTCATATCCATTAAGACTTGAGTGTCCGATAGTAGAACAATACTTCACTCTTA
TTTTGTCTCTAAGACTCCTTTTTTTTTTCCTTTTTTTGTTCTATTTGTTTTGCAGGTGTCAATGGAAG
TGCATGAGAGTCACTTCAAGGATCTTGATCCAATAATAAGAATATGTAATGTTTCCAAAAGCTTA
TGAACCTAATCTATGTTTTATGAATATATATGACTATCGGCTTTAGTCTTCAGTATCAGTTTCTTGT
TTACATTTGGATGTATCAGCTGCTAAAAAAATGCATAGTTTCCCATCCCATTTGAAACGACGTTTT
CTCTCATGTCTTCCACTAATATAATCTTCTGGTCATGAGAACAGAACAGGAGCTCTGTTTTTCTCA
CACAATGATCGAACACACTTTTGTCATATAGTATAGAGAAATATTAACACTTCTTTTAGGCATCC
GTATCTAGAGTCTTTGTTTCACTGTGACCTACCTAAGTATATCAAAACGCACGTTTGGAATTCTGA
ATGGCACTTTCGTAAATAGGAGGGTACAAACATTTCTCCTTTTAAATTGTTTTCATCGTGAACTCT
CTCACTCACTCAACAAAGTAAAGAAACAATGGCGTATTCTCTTCCTACATTTCCCCAAGCCTTAC
CTTGCTCGTCAACCAAAACTTCTTCCTCCTTGGCTACCTTCCGATCTCCTTTCTTAAGATTCAATGG
TTCCACTTCCTTAATCCCCTCCTCTATCTCCATCACTTCACGTGGCACATCCTCCCCGACCATCATC
CCACGTGCTGCCGCCTCAGAATCCGACTCTAACGAAGCCCTAGCCAACACAACCTTCCACGGCC
TCTGCTATGTCTTGAAAGACAACATAGACACCGACCAGATCATCCCAGCAGGAGCCGCTTGCAC
CTTCCCATCGAACCAGCAAGAGCGTGATGAGATCGCCGCTCACGCTCTCTCTGGTCTACCAGACT
TCCACAAAACACGGTTCATTGAGCCAGGAGAGAACAGATCAAAGTACTCAATCATAATCGGCGG
CGAAAACTTTGGTTGCGGATCGTCACGTGAACATGCTCCGGTCTGTCTTGGAGCAGCTGGAGCTA
AAGCCATAGTTGCTGAGTCTTACGCAAGAATCTTTTTCCGTAACTCGGTTGCTACAGGAGAGGTG
TTTCCGCTCGAGTCAGAGGTTAGAGTCTGTGAGGAGTGTAAGACAGGAGATACGGTGACGATCG
AGCTGAGTGATAGTGGTGGTTTATTGACTAATCACACGACCGGTAAAAACTATAAGCTGAAGTC
GATCGGTGATGCTGGACCGGTTATTGATGCTGGTGGTATTTTTGCTTATGCGAGGATGATGGGAAT
GATTCCATCATTAGCTTAATCCTCTTCCATAACCATGATGGTGTTCTTGTATGCATAACTATTTGAT
GAATATTTGTGTTTTAGAGTTTTCTATTTTCTTAACTTTTTTATGTTTGTTTGATTTGGTATTGTTGTT
GTTACCTAAAGAATAAGAAATCAAAAGCAAAATAGACCGACATGTTCCAAGATATGTGAATATG
GTGAGTGTTTACTAAATTATGCGAATATTGTTGTTGCAACCTTATTTTCTTCCAAGTTGGGCATCAA
TTTCCCTTTTTGGGCTTAGTCTTCTTTTGTTTGTAAATTTAGTTTGTTTTAGTTTATTGTTGGCCCATT
TCTGTAGGGTTTGGCCCGTTAAGTAAGTACCATGTAAACCCAGAAAATTTACAACGAAAACCCTT
TAGCAAAAAAGTAATAATTCCGTTGGTAACTTTTCTCATTGTAGTACATGGCATATTTTACTTACT
CTACTAACCAATTTGGTTTGGTTATTGAAAATTTGGCGGTTTAAACAAACCGATATAAAATAACC
GTTAAGCGGTAAAAGAAAGGACATCGCTAAACCTAAATTTGCGACTCCTACTTTATAACGCCGCT
CTTCTTCTCCATTGTTTGTATACTCT

CDS

ATGGCAACTACAGAGCTAAAACCCTCCGCCGTTAAAAACCCTAAAAGAATCGAAGACCATCA
CATGGTCCGAAGAAAGATTTGAAGAAGAAGACTAAGATTACTAAGAAGACTAAGAAGTCG

AAAGCTCCCACCTTTGATAAGACCATTGAGAAGAGTAGAAGTAACGACCAGAAAACCGACAAC
GACGAAGACGAGCAACTGTATTCGGAACCGGTATCGGCCTCTGAGCAGCTCAACTACTTTTTGAA
TCATCTTGACTCCGCCATTGGTATCAAGGTTTCTTCTTTAGAGCTTGAACCCATTAAAGATACGTG
TATAGTTGAGTTATCTCAAGGGTTGGATCAAGATGTAAGCAACTTGGGAGAACATATAAAGCTTT
CTTGTGGGTCTTCATGGAGAGAGACTCTTTGTGAAGGAGAGTCACTTGAAAGGAAAGTTGAACC
GGGAAACCCATCTGTTCTTGTTATTAGTTCCTCTGCTCTGAGATCTTTGGAACTTCTAAGGGGTTTG
CATTCATTGACTAAGCAATGTCCAGCAGTGAAGTTGTTCTCAAAGCATTTGAAGGTTGAGGAACA
GGTATCTCTGTTGAAGAAACGGGTTAATATTGGGAGTGGCACACCAAACAGGATCAAAAAGCTA
GTCGACATAGAAGCTTTAGGGCTTTCGCGTTTAGATATGATTGTGATCGACATGCACCCAGATGT
CAAGGGATTTTCCTTATTCACATTGCCTCAAGTCAGAGATGAGTTTTGGGATTTGTATAAAAACTG
CTTCCACCAGAGAGTCCTAGAAGGAAGACTTCGTATCTGCATGTATGGTCCAAAGCCTTCTCCAA
ACCTAAAGAAGAAGAACAAGAAATAA

Protein
MATTELKPSAVKNPKRNRRPSHGPKKDLKKKKTKITKKTKKSKAPTFDKTIEKSRSNDQKTDNDEDEQ
LYSEPVSASEQLNYFLNHLDSAIGIKVSSLELEPIKDTCIVELSQGLDQDVSNLGEHIKLSCGSSWRETLCE
GESLERKVEPGNPSVLVISSSALRSLELLRGLHSLTKQCPAVKLFSKHLKVEEQVSLLKKRVNIGSGTPNRI
KKLVDIEALGLSRLDMIVIDMHPDVKGFSLFTLPQVRDEFWDLYKNCFHQRVLEGRLRICMYGPKPSPN
LKKKNKK*

10. **AT3G19850**

Promoter (2 kb upstream)
ACATCAAATTTTAATGTTCGTACCGCGTTTCGAATGATTTGCCGCTCTGTTGTAATATTTGAATTAT
AATCAGTTCGTTGATGAAGCAAATAAATCATGGTTCTTGAACAGTGTTAGCCGACTAGTTTTTATG
TCTTAACCATTTTTTTCTTGCTCTCTAGAGCACTGCTTGAACATTATGATATTGTATAATTTTTGGC
ATAGAAATGTAAACTTGACAAAATTTTTGATGATTACTTAAGTTTTGGTCTCGAGATTCTAAAGAT
TTGCACAAAAAAACAAAGGATTGTTCTTCTAAAAGAAAAAGTTAAATCGAAAAGGACACAAAA
TTTATGTAATTTTATTGTATCCTCTTTTTGGGCTAGCTTTAGTCGATGAGGCCAAATGGGCCTGTTA
ATTATTTATTTTTACTTTAGTATTTTTTAAGTGGGCTTCTTCCTTTGGGGCTTTACATTTTTGTTGGTC
CCTGTAAAATCCTGTTAAAGGGCTTAAATACTAGAGCACAAAAATTTTAAAACAATAAAACCTA
AATCCTCTCGTCGCCTTCGTTTCTTCGTCCCACTCCCATCCCATTGATGAACCCTCAGAAGACTTTG
CCCTAAACCTCACATACTATTCATAATTTGTATGGATATTAAAATGATATTTTATTTGGAAACTTC
ATTTTTAATAATAAATAAAAACTATTATCGGAAGTTAGTCATATTTTTTTATTTTGTACTCAAAGA
GTCGTAATGTATATCGACATTGGTGTATCTTTTTTTTATAACATTTTTGTTACATATATCGTTTTCAA
TTACATATATTTTACATGGCTGGATTTGGATAATGATGAAAAGCAAGTAGTCGACTTTATTCACA
AACGTTAGAGTCATAAAACCCTACCCGGCCCATTCACATGCAATGAAAGTGAAACTCCCAACTT
TACAAGCAACTGATATATAAATATTCTACAAATAAAAAGGGAAGAAAACTCTTGAAATTACATG
GAAGTGGAATAATAGTATTACGAATTTATAATGTAATGCCAAGAAAGGGAGAATATTTATAATTC
ACATAAAAACAAAGGGGGCACATTCATATATACGTTACGTGTGTATAAAACAGCATATAAATAC
AGAATATATATATTACAACTTGAGTAGCATAGTCCTTGAGCTATTGACAAGTCATAAGTGAGAGT
TCATGAAGCATCATTTTCTGTGCTACTGGAAATATATATTTGATATTAGTCGTGACCTATAGTTCA
CTGTCTAAAATGTAAATCTTATTTGATTTGATGCAATACATTTTTACTTTACCCCAAATAACCTGA
ATAATGTTGGTCATAATTATGAATTATTCTCAACAAATTGTAAGCTATTTCCCTGCTGTTTGTTTAC
CCACCTACTGTATTTTTTTTTTCTTCCTCTAACACCAATTTTTATTTTATTTTTTGGCCATCCTGATCA
ATGAAGCATTAAGGAATCAATCTTTGCACCTGGTTATTTTTAGTAACAACTAACAACGCCTCGTC

ATATTGATTTGTGCTCTGCACACAAGATTATATGGATTTCAAAATAAACATGCAGTCAATTAATG
CAGTTTATATAACTTTTCTTAAGTTCGATGTTAGAAAAAAGGTTTTCTAATGTAAAGAAGTAAACT
ATGCATGTCAAGCTAATTGCTCCAAAATTAATATCATATAGTACATTTCGATTAAGCAATGAATC
TTATTATCTAATACCAAAAAAACTAAACAATAACGAACAATAATTTTTTCATTACAAACGGCAAC
AAATTATTACATTTTCACTATTTTAGATGGTCTACACACTAGGGTTACAAGTTGGTCCATGCGACC
AGTTTATACAATTTTTCGATGACGTTTGTATATAAGTTCCTTTTGCATAAATTTATATGAGTTATAA
AGGATGATAAAAGAAAGTGTACATTAGATAATTGAAATAAAGACGCCGAAAAAGAGATATATA
AATAGCCGTTTCCATGTGATAACTATT

CDS

ATGTCTCTTTGCTGTGATCTTCAAATCAATCTCAATAACCAATTTACTTTCTTCGTCAATCAGGATT
TGATCTCAGAGTACTCAGGCTTCTTGAGGAAGATGATAAAACAGAGCAATAAGAAGAAAAAGA
ATCACAAGAACAGTAGAATCATCATCGAGGTTGAAGATTTTCCAGGTGGGTCAGATGGGTTCGA
CTTGGTTTTAAGATTCTGTTATGGTGGAGGAATCTCGATAGATGTCTCAAATGTGTCCATTTTGCA
TTGCTCTTCTGTCTTCCTTGAGATGACAGAGAAACTCTGTTCCTCGAATCTCTTGCTTCGAACAGA
GAAGTTTCTAGAAGGAATGTTCTACTGGTCCTGGAACGACATCGTATTGTGTCTCAAGAGCTGCG
AGCAAGTGTTCTTACACGCTGATTCTTACGGTCTTGTTGATAAGCTTGTTTTCGGGGTTTTAGCCAA
AATCCCTCAGAATTCAGACGTGAGTCATGTCTTTTCATCATCCTCTCCGTCTTCCTCTGCCTCTGCC
TCAGCCTCCTCTCAGTCGCCGGAGACGGCAATGATTAGGTCGTATTCAGACAAAAGGTCTACTTC
GAGGTCTTTTTCTTGCAGGACAAGTAACGAGTGGTGGTTCGACGATATGTCAATTCTCGGGCCAA
AAATCATTGAAAAGCTGATAAATACACTTGGTGCGCATGATAAGAACAATGACAGCTTGGTCCT
CACAAAATTTCTTCTCCATTACCTCAAGACAAAGGTCCCAAACAAGTCAACCAACAAGCTCGAG
TATTCAGGTTTAGCTGATACAGCGGTTCAAGGAGTGGTTTTCGCAGCGAAAACCGCGTTTTCATG
CAGAAAAATGTTCTGGGTTCTGCGAGTTTTATCGGGATTTAGCATAAGTAAAGAATCAAGAATTG
GTTTAGAGAGGGTTATAGGAGAAATGCTGGATCAAGCAACACTTGATGATCTTCTGATACCAGCT
GGAGGAAAAGGAGAAAAAGGGGTTTACGATGTGGATTTGGTGATAAGATTACTCAAAGTGTTTG
TAAGAATTGGAAACACAGAAGAAGGAGATCAGAATTTGAGAATGAGAAGAATTGGGAAGTTGA
TTGATAAGTATCTCAGAGAGATATCTCCAGACCAGAATCTTAAAGTGTCAAAGTTTCTTGAAGTT
GCAGAGAGTTTGCCAGATTCAGCTAGAGATTGGTTTGATGGATTATACAGAGCCATTAACATCTA
TCTTGAGTCTCATCCGAAACTATCATCCGAGGATAGAACAAAACTATGTCGATGTCTAAACTACA
AGAAATTGACATTGGACACATGCAAACAACTTGCAAAAAATCCCAAGATCCCTCCAAATATTGC
AGTTCAAGCACTCAAGTCACAACAATTATCAAACGAGACTCGACCACACTCAAGAGAGGACAA
GAACAAAGTAAACAAGATCTGGAATTCACGTAAGTACTTAGAAGAGAAACCAATACTGGTGTGT
TTGAAAGGTTTTGATATGTCGGAGAAGTTTGAAGATGATCTAATGATGAATTTGGAGAGGAAGCA
ATGGAATAATTCTGAAAAAGTTAGTAAGGAGAAGAAGAGTGAAGTAATGTCAAGATCTGTGAG
ACATGGACATACACATTCAAGTTCTAGTTTTCCAAGGCTTTGTTAA

Protein

MSLCCDLQINLNNQFTFFVNQDLISEYSGFLRKMIKQSNKKKKNHKNSRIIIEVEDFPGGSDGFDLVLRF
CYGGGISIDVSNVSILHCSSVFLEMTEKLCSSNLLLRTEKFLEGMFYWSWNDIVLCLKSCEQVFLHADSY
GLVDKLVFGVLAKIPQNSDVSHVFSSSSPSSSASASASSQSPETAMIRSYSDKRSTSRSFSCRTSNEWWFDD
MSILGPKIIEKLINTLGAHDKNNDSLVLTKFLLHYLKTKVPNKSTNKLEYSGLADTAVQGVVFAAKTAF
SCRKMFWVLRVLSGFSISKESRIGLERVIGEMLDQATLDDLLIPAGGKGEKGVYDVDLVIRLLKVFVRIG
NTEEGDQNLRMRRIGKLIDKYLREISPDQNLKVSKFLEVAESLPDSARDWFDGLYRAINIYLESHPKLSSE

DRTKLCRCLNYKKLTLDTCKQLAKNPKIPPNIAVQALKSQQLSNETRPHSREDKNKVNKIWNSRKYLE
EKPILVCLKGFDMSEKFEDDLMMNLERKQWNNSEKVSKEKKSEVMSRSVRHGHTHSSSSFPRLC*

GmCAMTA12 regulatory Network

1. GLYMA19G40910.1: (GmNIP 30)

Promoter (2 kb upstream)
TTAGAGTCTGAAGGATTAATCAAAGCGCAATAATAATTTGTGCTTAAGTTTTGCTCTTTGACGACC
TCTCTTCTAGTTTTAGTCTGACTGACATCAACATCAGGGGAGTATCGAGTAAAATAAGGAGTTTA
TAACATTAGTTAGTGGTTTAGTAATTCATGTTTATGTGTCATTCCCTTGTAACAATCTATCAATAGT
AGCATCGCCTAAGATTAAAGGGTGGGGAACTTTGTACTAAAAACTCATTCATAAAGCCTACACA
TACGTCCTCTTAGATAAACAAGCATCTAGAGGAGAAAACATATAAAGTTCCAACAAGAAAGAA
AAGTTTGTTGATTTTTCTTTCTTTCGTTGTTTTTATTACTTTTATTTGTTTTGCTTCTCATGTTTCTAGT
GAATTATTATATTTGTTGTCCATAGCCCTTCTTCATTCTATCATCTAACAATTTGCTGAGTTTTCTTG
ACATTATTGAATCAATGCAAGGCTATGAAAAATCATCGTCAACACAAGCCTCTAAACCTTTCACT
GAAAAAAAAATTATAATGTTTCACATTAGGAGATTGAAATAACACATTTCTTCTTACAACATGAT
TTTTTTTTTACATTTCTTGCAATTTGATTAAAGGTATTAATGAAAATTGCCAAATTTAGCATTAAGT
AGATAGAAAAATAAACATGATTACAACAACAACAAACTCATACTTTTGGTTCACAATTTTTAGGA
TAAATGCATCCTAATGACATATGCTTTTGGCTATGCCCCATAGGATGTCAAATTTTCATTTTTAGA
GAAAAGATATTGTTAATCCTAAGCCCTAGTTCCTTAGCTAAGGCTCAACAATGAGATTGGTTTTTT

TTTTTGCCCTACTTGAGAAATAACTGACTCATCTTTTTTAAAAGTCTCATCAGGTGAAAAAACTCA
TTGTGAAGTGTAATCTAGATCATACCAATAATCATAGAAAATCTCATATGTGACACCTTCTACTCC
TCACATATATATATTAATAATAAAAGGAATAAAAAATTAGAATTAATTTAAAAAATTTAAAACA
CATTTAAATACAAGCTTTTCGAAAGGATAAAAAATATAATTTATATGTTAAAAAATTAGTTATAA
TATAATGTATTTATTTAAAATATTTATTATAAACCTAGTTATATGAAGTCAACATTTAAGATATTA
GTTAGTTTGAAATCTTCAATTCACGTATTATTTATCCTTTTCCTTATGACCATAGATTTTTTTATATC
AAAACACTAAAATATTTGTAATATAAATAGTGATCCAAGTGTTTTAAGTGTTAGAAGTCCCATAT
CTATTTTTTCCAAACATTTTTACCCCCTTCAAATATAAATCTCTCTCTGCAGGTCTCGTATTTGTTTC
ACTTGTCATCTCATGAGCTTACACTTCCCCTAAAATGGACAATTGGCTAAATTATATCTCATATGT
GAGATTTTTTTCTCCATCCACAACTCCTTGTCATCAAGACTTTTCAAAGATTTATCATATGTTGTGT
GATTTTCTTCATCATTAACATCCAAATTGAGTTCTCTATAACGATTCTCGAATTTAATGACTATATC
ATTTGGGTTCTTAATCCATTAAAAGAATCTTGAGGTTCCTCACGAGGCAATGTTGGATCCTCTTCC
ATTGGTGTAATTATTTTATCTCGAGAATACCTTTGAGATGTATTTTGTGATGAAATATTGACATCCT
TTAAGTGCGAGGAAATGAAATGAAATCCAATTCAACAATATAATAAACATAAATGAGATGTAAC
TTTTGTATGTGTGTTCAATAATATAATCATCTTAAATAAAATGGTAAACATTTATTATTGAAGAAT
TTATGCCAATACAAGTTGACGAGACATTTATGATGAAGGTAATGTCGTTTTTTTAAAACATACTA
AACCCAATAGGACAATAAAAACATGATGTTAGTATTTATTTATTATTTGTAGTTGGATTATTGTTT
TTGTAACAATTTATAGCTGGA

CDS

ATGGATGAAGATTCGGATCGTCCTATCAGGATTATGAACTTCGTCTCCGAAGACCAATTGGTAGA
AGCTAAAAAAACAAGAGGTGAACGAGTTGAAGACGGCACTGCCCAGAGGGATAGACCTCTCTA
CGAGATTCTAAAGGAGAATAAGGATAAAAAAGATGCGGAATTTAATGAAAGGTTCAAGCACAG
ACCTCCTAAAGCTTTAGACGAAGATGAGACTGAGTTTCTTGATACTTATGAAACAACAAGAAGG
GAATATGAACGACAAGTGGCAGATGAGGAAGCCCAACAAATCCGAAGCTTTCAAGCAGCGGTG
GAAGCACAGTCCAACATCGTGCATGAAGTAAAGGAAAAAACTCCATTACCTGTCGTCCAGGAAC
AGAAGTCTGCTGGAAAGAAGAATCCAGCCTCTGGCCCATTAGGCATGATTATAAAAGTCAAGCC
TCAAGCTAAAAAAGCCAAGGTGGATCAAGGAAATGCTGAGGAAATTTCAAAGGCAGGAAACAC
TCCTGTGAATGATAAGAGTAAGTCGTTGGAAACAGTACAGCCATTGAATGGTGAAGCTGATAAG
TCCCAAGTTGTTGCCCTAACTGGCCTTGTTTCGTACAGTGACGAAAGTGACGAGGACTTGTAATA
ATCACCTCGGAA

Protein

MDEDSDRPIRIMNFVSEDQLVEAKKTRGERVEDGTAQRDRPLYEILKENKDKKDAEFNERFKHRPPKA
LDEDETEFLDTYETTRREYERQVADEEAQQIRSFQAAVEAQSNIVHEVKEKTPLPVVQEQKSAGKKNPA
SGPLGMIIKVKPQAKKAKVDQGNAEEISKAGNTPVNDKSKSLETVQPLNGEADKSQVVALTGLVSYSD
ESDEDL)

2. GLYMA12G15430.2: (GmPLA 1-IId)

Promoter (2 kb upstream)

TGGCTTTTCTTAAAACAATTGTGCTCACCCTAGGTCAAGGCCCAAAATGACTCTTCTTGAAACTAG
AGGTTTTTTTCGCAAAACACTTATTAGAACCCATTTTTCTTTCTTACAAGGCCCAAAAGCATATTTT
CTCTTATGTCTATATTTTAAAAAGGCTAAAATAGAATATTTATTATTAAATAAGGTATATGCATAA
ATAATAATAAAAAATAGAATTTGTAACGGTATAATGTGCATATAGTTAATATCTTGTTGTTATTCT
ATATATGAGTAGTGGTACATGAACAACTCATAATTTTATACATTTTATCAATACCTTTTATTTTTCT
TTATCTCTCTTTTTATCACATCATAAACCTTATCATACAATATGTGAATAGTTAATATCTAGATGTT
GGATAGCATATTAATGTCTATCAAATATTTTTCTATTATTTGAGACAAGTAGGTGTGTGGGAGTCA
AATTATCCTTCCAAATTAATCACCCTACAATAAATGCTAATCACTAATCAATTAATATAGAATATT
GAGTAGATATTAACTATCCACACCCTGATCAAGGATTGAAACGGCATAAGCAAAAATTAGTGTA
AAATTATGTTTTTAGTAATATACAAAACCTTATTATTATCATCTCTGCCCTCACCATAAGCTTTCTC
ATCTTTTTAGAGTAAAAGTCCTTTTTTTAAAATAAAAAATTATGGCCCACAAATTAAAATTCTATC
CACTAGTGTCTATTGATAAGGAATTCATTTTGTCTTGACTAGGGTAAAAGCAAAGTAGCTATGTCT
TAATCTCGATATAGAAAAAATCAGATAGGCTTTCGCCTTCCTTTTTTCCGTCCAACGAGGTAGTCT
TTGGAGAATTATTCCTAATAATATATATATATATATATATATAAAAATTAACTAATTAAGAATATA
AAATAATTTACTACGAAACCTTTTTAACAAATTGAGTAATGAATAGGTTTAATTGTTTTTTTATAT
GTAAGTTAGTATTTTTTATTTTCGGTCACCGTAAGTTTATTATAAATTTATGTTTTTTTAATTTTGAT
TTTTGTAAGATATTTTTATTTATTTTTAATTCTGTAAGATATTTTTATTTATTTTTAATTCTGTAAGTT
TACATTTTTTAATTTTAGTTTCATTTTAACTTTTCCATTTATACTCTTCAAAATTTATATTTATAGGA
ATCAAAATTAAAAAAACACAAATTTATCACACCAAAATTAGAAAAAAAATAACTTACACGTATC
AAAACTGAAAAATATACTAACTTACCGGGGGAAAAAAGATATTTAAGGCTGATGAATATAATCT
TGTTAACGCTAATAATGAGAAAAAAACACACACAGTGATTGACTAAGAAAGAAAGCCGAATAA
TGTTTTAACACGTTAAAGCATTTCCTTTATTTTAGATAACATTTTCTAATTATTTGTTAACTTTTATT
TTGTAAGGCATTGTTTTATAGGAACGTTCCTATAAATAAATCTTCTTAGTATTTGATAAAAAAAAA
TATCGAATGCCTTGAACCATAATAAGCATATTCAAAACTTATCTAGCATAACTGCATAAGAGAAT
TAATAGAAGGATGCAGTAAACTGTAAAAGGGATTTAAAATTATTATTAATGAATTTAATTTTCAC
ATATTATTAATATATATTTTTAAAATCATCTTAGTTAAATATAATTTAAAAATAATTATTATAAAA
AAATTCAATAAACTTACATAAGTGATATATATCATCATTTTTTTTATAAGTACAATGATATCATCA
TTTGTAACTAAATAATAGTAAAAAAATACCTTTTTTAAGAGGTCAGATATATTTTTCACTTGAGAT
ACTTCAACTAGAGTCATGGTGATTATTATGTCAACAATGTTCCTCTGTCAGTATTAAGAATTTTGA
CCTCAAACTACTAATTAAACCAAAATAATCTAAACTTTATTCATCGAGTAATATCTATTAAATAA
CCGATGGTAAAATCATTCC

CDS

ATGTCGGGTTGGCTCACAATCTACACGTCTGACAACCCAAAATCCCCCTTTACCAAATCCAGCGC
AAGAACGCAGCTTCAAGCCCACGTCAAATCCCTCTTACAACATTACAGCTCTGAGAACCCCAGC
TTGGTCATCGTGGGGCACAGCCTCGGCGCAACCCTATCCATCGTGAGCGCCTTCGACCTGGTCGA
AAACGGGGTAACGGAGGTCCCGGTCACCGCCATCGTGTTCGGGTCCCCCCAGGTCGGAAACAAG
GCCTTCAACGAGAGGTTCAACATGTTTCCGAACTTGAAAGTTTTGCACGTGAAGAACGTGATCGA
TTTGATCCCACACTACCCGGGGAAGTTGTTAGGGTATGAGTACATGGGCACGGAGCTGGTGATAG
ACACGAGGAAGTCGCCGAGCTTGAAGGACTCGAGGAACCCGGGTGATTGGCATAACTTGCAAGC
GATGTTGCATGTGGTGGCGGGGTGGAATGGGAAGAAGGAGGAGTTTGAGATGAGGGTGAAGAG

GAGTGTGGCGTTGGTGAATAAGTCGTGTGAGTTTCTGAAGGAGGAATATGGCGTGCCAGGGTCGT
GGTGGGTGGAGAAGAATAAGGGGATGGTGAAGAGGGAGGATGGGGAGTGGGTGTTGGATGCGC
CAGATGAGGAGGATGTGCCTGTGCTCGAAGAGATTTGA

Protein

MSGWLTIYTSDNPKSPFTKSSARTQLQAHVKSLLQHYSSENPSLVIVGHSLGATLSIVSAFDLVENGVTE
VPVTAIVFGSPQVGNKAFNERFNMFPNLKVLHVKNVIDLIPHYPGKLLGYEYMGTELVIDTRKSPSLKD
SRNPGDWHNLQAMLHVVAGWNGKKEEFEMRVKRSVALVNKSCEFLKEEYGVPGSWWVEKNKGMV
KREDGEWVLDAPDEEDVPVLEEI

3. GLYMA18G40360.1 (GmNAB)

Promoter (2 kb upstream)
ACAATGTAAAATAGGATCAAAGTATCATAAACGAAAACGAAAGTCGTAGAGGAAAGAAACATG
CAAGAAAATAGAGGGAGTGAGAGATGAAACAGGAGAATAAAACATGAGATAAACAACTAACT
TTATTGGAGAAAGGATAAAAATAGATTTTCAAATCAATATGTAAATAAAATAATAATAATAATA
AACATATAAGAAAGGTGAGAAAAAGAAATAATGGATTCAACACATAAAAAAATAAGAGGTATA
AATAGTAAAATAAAGGGGTGCATTTATTTATCTTCAAATGAAAATAAGTGAAGCAGGGGTGCAA
ACATATGTAATTAAGGATGCAAAATATAAAATTTTGACACAAATATAAGTGACTTTATTAAAATG
AGGAAGTGTGTTTGCCCACCCTCAAATATATATGGGTCTGCTGCCCTGGGTCTAGGGTTCAGATA
GGAGTGTCCTAAGGTTTAGGTTGTCATGCATAGTCACATAATGAGTTTGTTGCTCTATAACTCATT
GACTTAGTGAAAAATCTCAAGTGCTACTTGACGACAACTAGACATAAGTTAAGTGACCTGAATT
AAGATAAAATATTAGTTCAATCTTTTTATCCCTTAAACTTTTTAATTACTTGTTTTTATTATTTGTGC
ATAAATTATGTTAGAGTTTTGGGGATTTAACTCAATTCTCTTTATTATTAGAATTATTAAGTTATTG
AAGTCACTTGATTAACACAAGTTGGTCTCGATTAGCCGGACTAGACAAATCATGAACATCGCTAC
TTGTTACTTATAGTATTTTTTTCCTATCCTTGCATATTAACGCAGACTTATGTGTCAGCTAAGTTGTT
GTTATTAAATGTCAGCCTCTATTAATCGACACAAACAATTTAGTGAGACATGATCGTTGCTTAACT
AACGGTAAATTCAATTTAAGCAACACCTTTGCATGAGTAACGACTTTTAGCAGTCGTCACTAGCT
AATTGCGAATTTCTTGTAGTGTCTTGTTACTGTAGTTTGACCTGTATATTTTTTGAAAGACAAAATC
TGACACTACATTTAACTACTTATATGGTTGGTTGAAAGTTAAATTCAATATGTCTAAAATTTCATA
ACCAATGGTGCGGTTGGTCAAGTGGTTTGTAAGTTTGTCTTGATAATGAGCACAGGAATACTCTC
AAGTGAGTGGTCCTTGATTCGACTCCTGTGGAAAATAACAATTCTTGAAAAGGAGGAGTTGGTAG
AAATTGATGCAGAACTATCTCTAAAAGGAGGAAACCCATAAAATAAGTATATAAAAAGTATTTC
TAAAATTCCATAGCTATCATCATATCATGCTATAGATTTTACATAAAACAATGATAGATTCATTTG
TAGCAAGTTGCCATTTTAGTAACAAGAAAAATGGAAGATAAGAAGATCAGATAATAAATTAAAC
TATCTGATTAAAGAGACTCTAACACCATACGAACAAACCATGTAATTTAAATACTTAAACCTCTA
TATAATAAATAAGATGGAATGATCCTATCCAGCTAAAACAAGATTATCTCTGATAAAAGATATTT
TTGTCTATAAAGAAAATGGATAAATTTCATCAAAGCACTGGAAAAATAATAAACAAAATACTAA
TTCTATTTCAGAACTTATGATAGAAATATATCCCCTTATTGCTAATGAAGAGTTTAGTACCACCAC
AAGTGGACCAAAATGTTTATTTAATTTTTCACTTTTCAAATATATCGTTACATTACCAAAAAAATA
TCACAGATGACAGCTGCTACTGTGATAAACGGTTAGCTAATTCTTTGATCGAACACATAATTATT
GTATTTGCGTACCTAACACAAGCTAATACAATGAGCATATACCTGAATTAACTTTTACCCTCAGG

GAAAACATTTTTTTAGCTAGCTTTTCACCGAGACTAAAAGAGCACAACTATAAAATCATATAAAC
TCTTATTGATCCTCTCAAGGCAGTGTAAAGCAAATGTCTAGTAGAAT

CDS

ATGTCTTATTGCAATTTCCCCTTCGCTTTGCAAACAAAGATGGAGTTCAATTCCCAAAACCCTAAA
AAATCTGAGATAAGATGGAGCCCAGATAATCAAGGTGGTCCTGGACAAGGGAAGTCTTATTCTT
GTTACTTTTGCAAGAGAGGGTTCTCAAATGCACAGGCCCTGGGAGGCCACATGAATATCCATAG
AAAAGATAGGGCGGCAAAGCTCAAGCAATCTTCTGAGGAAAACTTGCTTTCATTGGATATCTCA
ATCAAGGCCACAAGTGATCATCCTAATGACCCTTCTGATTTCGAAGAGAAAATTTTGTTTCGGTTG
GGTGCTGGTGAAGAGAAGCATCCTAGAAACCATAAATATCCATTCAATTTTTTCCTCGCAAAGA
TGATCATCATGATCATGCACCACAAATTCCCCATCTTCCTTCTTTTGTTTTGGGGCAGATGGTTGA
GGAGAAGAAAGCTGAATTAGACCTTGAGCTTAGGTTAGGGCTTCATCCTCGGGAGTCAGCAACA
TTGAACACTAGATCGTTCTTTTGA

Protein

MSYCNFPFALQTKMEFNSQNPKKSEIRWSPDNQGGPGQGKSYSCYFCKRGFSNAQALGGHMNIHRKD
RAAKLKQSSEENLLSLDISIKATSDHPNDPSDFEEKILFRLGAGEEKHPRNHKYPFNFFPRKDDHHDHA
PQIPHLPSFVLGQMVEEKKAELDLELRLGLHPRESATLNTRSFF

4. GLYMA06G18150.1 (GmELO)

Promoter (2 kb upstream)
GCTATATGAGCCAAGTCAAAAAAGCTTATTTTATTTGAGTTATCTTTTTTGAAACCCAACCTAAT
CCTAATTGCGAGTTGGACCATCACCCTAGATTCATTTTGCAAGCTCTAGTTATGGGTATTGCATAG
GTGTGTGCATGAAAAATATGCTCCAACTTCAAAAATATTTTTCATTTGCACACTACACATAAGTTA
ATTAGGTAATGCTAAAAAAACTTGAAAACATTAAATGCATTACAACATCCAACTAGAAACAATT
TTGATAAAATGTTCACAATTATTATTTAAAATGTCGTCATTTCAATTAACATAACCATCATTCCAA
TTAGTGTTAATTAGCACATCATACTTTTTCCTTAACGTAATCAATGTTTCAGTAAAAAAATGAACA
AAATAATTTTTTTGAAAAACATAAAGAACCAAAGTTAGACCTTTAAAACTTAATAGACCAAAAT
AGAAAAACATAAAAATATAAAGGAGTGAAAGTGACATTTGACCAATTGTAAAATATAAATCTA
AATAAAAAATACATCAAAATGTATTTTAGTGTTTTATGTTTACAATTATATTCAAATTAGTTTGTTG
TTTTTTAAAAGTTTAATTTAGACCTTTTAAAGTGAGTTAAAATGATATTTTTAAAATAATAATAATT
TTTTATTTTTGGAACATCATTTTGAACCTTTTGTTGATTGATTGAGACATGAAAATTGTCTCATAGA
TTCACGTAGATGACAATTATAAAATATTAAAAAAAATCATAAATCAATAAAAAAGGGTTCATGT
TCCAAAATAAAAAATTAATTATTATTTTAGAAGGATGATTTTGATTCACTCAAAAAATGATTAAA
TTGAAGTTTTAAAAAATGATAGATGAATCGTTTCTAACGAGGACTCACTTAAAAATATAAATGAC
TAAATTTAATTGTTTAAACATTGGTTTGGTTGCACATTAAGATAAGGAAGCACGGGTGGAAGAAC
ACATAATTGAATGGATATTGGGATATGTGTGTGGTTTGGTAGGACAGAAGAGGGAAGACGTGTG
GAGAAAAAAATGCTACTGTTCATTTCTTCACTAAACATAGTGAATAAGCGCAAAATTGGGGTGC
ATATTGCAGACCCTTACTAGAGACTAGAGAGCCCGTAAAGGCACAGGTTTTGTGCCTTCCTTTTTT
CAATGTTTTCTACTTTTTTTTACAATATAATAATATATTTTGATTCATTTTTTTTACTTTTAAACTAA
TTTTAATTTAAATATTTATCTATTATTTTTTATTGATCTTTAACCAAATAACCTCACTTTCACTTTAT

TTCTTACCTACTTTTTTTCTATTATTTTTTTTATTCTCAATCAAACACACTTTAAAAGATAAAAAATC
AAATTAAATATAATAAAAAAAATAAAGACTAAATATCATTTTAGCCAAAGTTAATAAATTTGTTTT
CTTATTTAAGTCATAAACTTAAATATATTTTTGTTTTTTATATTTAAATTAATGTTACACAACATTT
ATAAATTTATGAATTAAGTTGATTATTTAAAAATGTGAAGATTTGTTTACAAATACATAATTCAAC
CACTAAATATTTTATATACCGGTATGATTGACAATTTATAAGATAAGATGACAAGAATATTTTTA
AGTATTAAAATTTAATTTAACAACATTAAGGCCCGTTCATTTGCATAGAAAAATGATAGAAAATA
AAAGAAAGTAACTCCATATGAGACTCATATTTTAAATCAAAACTTTATCATATTTCACTTTTATTT
TTTTATTTTCACTTTACTGGACCAACCTAAATGAGATGAAATACTAATAAAAAACCTAATTATTAT
TTTTGGAAAAAAATATTAGAAACACTGGGGCACACTTTCATTGAAGACAATTATTAAATTAACGT
ATAAAATAAGATAATTATTAAATTTACAAGAGAAAATCTGAAGAAAATAAATTACGATAAATTA
ACATTTTATTGAGAGTAGTAACACAAGTCAC

CDS

ATGGCGGCGGTAGCGGTGGCAGAGGTTGGGAAGGCCCCACGGCCGGGCCAGGGCGGTTACGAA
GCGCACGGCCTGAGCGAGGAGGAGGCTCGTGTACGCGCCATCGCGGAGATCGTGAGTTCCATGG
TGGACCTCTCCCACAAAGGCCAGAACGTGGACCTGAACGCGCTCAAGTCCGCGGCGTGCCGCAA
GTACCGGCTCTCACGCGCGCCGAAGCTCGTGGAGATGATCGCGGCGCTCCCCGACGCCGAGCGC
GAGACGCTCCTCCCCAAGCTCCGCGCCAAGCCCGTCCGCACCGCCTCCGGAATCGCCGTCGTCG
CCGTCATGTCGAAGCCGCACCGGTGCCCACACATCGCCACCACCGGGAACATCTGCGTCTACTG
CCCCGGGGGACCTGACTCCGACTTCGAGTACAGTACCCAGTCTTACACCGGTTATGAACCAACCA
GCATGCGTGCAGTTCGTGCGAGGTATAATCCATATGTCCAAGCGAGGTGCAGGATAGATCAGCTT
AAGCGTTTGGGTCATAGTGTAGACAAGGTTGAATTTATCTTAATGGGCGGTACCTTCATGTCACTT
CCGGCTGATTACCGTGATTACTTTATAAGAAATCTTCATGATGCTTTGTCTGGACACACATCTGCC
AATGTGGAAGAGGCAGTGGCTTACTCTGAGCATGGGGCAACCAAGTGTATTGGCATGACAATTG
AAACGAGGCCAGATTATTGTCTTGGGCCTCACTTGCGCCAAATGCTTTCTTATGGTTGTACACGAT
TGGAGATTGGAGTCCAAAGCACCTATGAAGATGTTGCTCGAGACACAAATAGAGGACACACTGT
TGCTGCTGTAGCTGATTGTTTTTGCCTGGCTAAAGATGCTGGTTTCAAGGTTGTTGCTCATATGATG
CCTGATCTTCCAAATGTTGGTGTTGAAAGGGACATGGAAAGTTTCCGGGAGTTTTTTGAGAGTCC
CTTGTTTAGAGCAGATGGGCTTAAAATATATCCTACACTTGTAATTCGTGGAACTGGGCTTTATGA
GCTCTGGAAAACTGGCAGGTATAGAAACTATCCACCAGAGCAACTTGTGGACATTATAGCAAGG
ATCCTTGCAATGGTACCCCCATGGACACGTGTTTATAGAGTTCAGCGGGATATTCCCATGCCTCTG
GTTACCTCTGGGGTTGAGAAAGGAAATCTGCGGGAGCTGGCATTAGCTCGAATGGAAGACCTGG
GATTGAAATGTCGTGATGTTCGGACCAGAGAAGCTGGAATCCAGGATATTCACCACCAAATTAA
GCCAGAAGAGGTGGAGCTTGTTCGGCGTGATTATATGGCAAATCAGGGTTGGGAAACATTTCTAT
CATATGAAGATACACGGCAGGATATCCTTGTTGGTTTGTTGCGGCTGCGAAAATGTGGCCGCAAC
ACAACTTGTCCAGAGCTTATGGGGAAGTGTTCTATTGTTCGTGAACTCCATGTTTATGGAACTGCT
GTACCAGTTCATGGACGGGATGCTGACAAGCTACAACACCAGGGTTATGGCACACTTTTAATGG
AGGAGGCAGAGCGCATTGCTTGCAGAGAACACAGATCAACAAAAATAGCTGTAATTTCAGGGGT
TGGCACACGCCATTATTACAGGAAACTGGGATATGAGCTTGAAGGACCTTACATGGTGAAATAT
CTAGTGAAATAA

Protein

MAAVAVAEVGKAPRPGQGGYEAHGLSEEEARVRAIAEIVSSMVDLSHKGQNVDLNALKSAACRKYR
LSRAPKLVEMIAALPDAERETLLPKLRAKPVRTASGIAVVAVMSKPHRCPHIATTGNICVYCPGGPDSD
FEYSTQSYTGYEPTSMRAVRARYNPYVQARCRIDQLKRLGHSVDKVEFILMGGTFMSLPADYRDYFIRN
LHDALSGHTSANVEEAVAYSEHGATKCIGMTIETRPDYCLGPHLRQMLSYGCTRLEIGVQSTYEDVAR
DTNRGHTVAAVADCFCLAKDAGFKVVAHMMPDLPNVGVERDMESFREFFESPLFRADGLKIYPTLVIR
GTGLYELWKTGRYRNYPPEQLVDIIARILAMVPPWTRVYRVQRDIPMPLVTSGVEKGNLRELALARME
DLGLKCRDVRTREAGIQDIHHQIKPEEVELVRRDYMANQGWETFLSYEDTRQDILVGLLRLRKCGRNT
TCPELMGKCSIVRELHVYGTAVPVHGRDADKLQHQGYGTLLMEEAERIACREHRSTKIAVISGVGTRH
YYRKLGYELEGPYMVKYLVK

5. GLYMA05G37930.4 (GmSRL)

Promoter (2 kb upstream)
CCCAGTCACCATGATGCTCCTAATTTTCATAGACTTGAGAATGGAAATGACTTCTTGTGCAGCTGG
TTTCAATGGATCAGATACTACTAAAACCCCAACTACTTCCCTATTTATAGACACTATAATTCCAGT
TTGAGCCATTCCTTCCGCTGCTGCAAGTATCTCTTCAGAATCAACAGGAAGTGCAACATTGTGGT
CCGCCATCAAGGTCTTGTTAACCATAAGTCAATTGTAAGTTTTGTGTTTATCTCTATATTAATTTTT
GAGTTTGCATATTTGGTTCTTCTAATTTTTTAAGTAAATCAAGTCTATTTATTTTAAGATTAAACCA
ATTTAATTTTTCTGTCAATTGTATTTTATTGAAAACTTTTATATAGTTTATTTGGTAAGGAGAGAAA
TAAAATAATATAAAAAGAGAATAGAAAATAGGATCATTTTTAAGTTGTTCAATAAAAAAGAAAA
TGAAAGAAAAAAAGTTAAACTTTTTCTTTCATTTTACTTAACCGTGAAAAAGTTAAATAATATAT
ATATATATATATATATATATATATATATATGTATATATATATATATCCATCTGTATAAAAATAAGAAT
AATTTAATTACACAAAAGCAATTTTCTCTCACTTTCTATCCAGAATGGGATGAAATAAAACAACA
ATGAGCTCATAAATATTTCCAATAAAAAATTATAAAAATAAGAGTTATTTAATAATTAATTACCC
AAAACTCCAAAACTCCAAAACAACCTTTTTCTGCTTCTGCCCAGAATGAAAAAAAATAAATGAA
AAAAATAGCGAGTCTCAATTACTTTTTTATGGTTCCTTTCTTCCTTCTGAACCATTATCATGTAGTG
AAAAGATTTTTCTTTCTTTTTCCTTTTCATTTCATAGGCTGATTTCGGTCTTCTCCCTTTCATTGTTCC
AAACATTATGTAGAATTCTTGGTTTCTTTCCAAATCTTTACTTCCTCAGCTCCATAATAATTTTCGT
ATAATTTTTTAAAATAATTATTACTTTAACTTTTGAAAGTAACATTAAACATTTTTATTAATATTTT
TTTTATAATATTAATGATCAACAATAAAAATTAATATTAATTGATAGGATAAAATTAAATTTTTTT
ATACATGATAAGATTAATTTTGTATAATTATCATTTTTAATCTAAGATAATATTTATAATGAGATA
GGAAGAGTTTATTTTATGATAGTTTAAAACGTAAAATAATTTTAAAAATTTAGAAGAGACTTCCT
GTAGATTATAACAAATTTTATAAAGAGAAAATTTGTTATGTACTGCGAACGTAACTCACTTAATC
ATAATTTACAATATATGAAGTTGTTGACTTTTAACTTTTTTTTAAATAATTTAAATAATGATTTAT
GATTGAATAATAATAAAAAATTATATAGTTATTAAACTTTTTATAAGATATATAGTTGTGTTCGGT
AAAATTAATTTGAAACTAAAAGGTCAACTAGTAACTGGAAAACTAATTTATTAAATTACAAGAA
TTTAGTAAAATTAAATTTTGAAGTAACTAAAAAATATAAATGACATAAATAATAAAATTATGATT
TGTTTAAAAAAGGATAATCAAGAAATTAAATAAATATATTGAAAATAATAAAAAAATATAAAA
AATTAGAAATTAATGTTAAAAACACACTAATTCAAGTAATTTTTTAAAATACACTATAAATTATTT
AAAAATTATTATTTATTAATCAGTCAAATAAATTTTTCAATTAATAAAAAAATTAAAAACTAATTT
AAATATCTTGTAAAAAATAGCCATAAGATTATATTCAAAAAGAGAAAACATTTAAGATTCTATGT
AGTAAAATTTAATGTATATTTTTAGTGAGAGAAATATTTAAAACAATTTCAATGAATCAATTTTAA

AAACAATGTTTAAAAAATAAAGAGCTATAATATAAGATTTTAAATTGAAGAGAAAACGAAATTT
CCTAACAAGATTTAATTATTAATTAACTA

CDS

ATGGAAGAAGAGAGCCAAATAACAAAGGGAAAATATGCTGCTGATATTTCTTCCATAAAAGAA
GCACATGCCAGAATAAAATCATTGGTTCATAAAACTCCGCTTCTGTCCTCCAGCTCTCTGAATGCT
ATGTCAGGAAGGAAGCTCTACTTTAAATGTGAATGTTTACAAAAGGGTGGAGCTTTTAAATTTAG
GGGAGCCTGCAATGCTGTGTTTTCTCTCAATGATGAAGATGCTTCTAAAGGGGTTGTAACACACA
GCAGTGGAAACCATGCCGCAGCATTGGCTTTGGCTGCAAAACTACGGGGGATCCCTTCGTATATT
GTTATTCCAAAAAATGCCCCAACTTGCAAAATTGAAAATGTAAAGCGGTATGGTGGTCAGGTTGT
CTGGTCTGAAGCCTCTGTTCAATCAAGGGAAGAAATTGCCAACAAAGTGTGGCAAGAAAGTGGT
GCTATCTTTATACATCCATATAATGATGGACGCATATTAAGTGGCCAGGGTACCATATCCTTGGA
GATTCTGGAACAAGCACCACAAATAGATACGCTAGTAGTACCCATAAGTGGTGGTGGTTTGATAT
CAGGAATAGCATTGGCTGCCAAGTCCATTAACCCAGCTATTCGTATTTTTGCTGCTGAACCTAAA
GGTGCTGATGATGCAGCACAATCTAAAGCAGCTGGGAGGATAATACGATTGCCTGAGACCAACA
CTATAGCTGACGGGCTTCGAGCATTTCTTGGAGATTTCACATGGCCTGTTGTACGAGATCTTGTTG
AGGAAATTATAACTGTTGAAGACAGTGAAATAATAAAAGCCATGAAACTGTGTTTTGAAATTCTT
AAGGTTGTTGTAGAACCAAGTGGAGCAATTGGCCTTGCTGCTGTTCTATCCGATACTTTCCAGAA
AAATCCTGCATGGAAGGATTGCAACCATATAGGAATAGTAGTTTCAGGAGGCAATGTTGATCTG
GCCATGCTTTGGGATTCTTTGAACAAAGGAAAGTGA

Protein

MEEESQITKGKYAADISSIKEAHARIKSLVHKTPLLSSSSLNAMSGRKLYFKCECLQKGGAFKFRGACNA
VFSLNDEDASKGVVTHSSGNHAAALALAAKLRGIPSYIVIPKNAPTCKIENVKRYGGQVVWSEASVQSR
EEIANKVWQESGAIFIHPYNDGRILSGQGTISLEILEQAPQIDTLVVPISGGGLISGIALAAKSINPAIRIFAA
EPKGADDAAQSKAAGRIIRLPETNTIADGLRAFLGDFTWPVVRDLVEEIITVEDSEIIKAMKLCFEILKVV
VEPSGAIGLAAVLSDTFQKNPAWKDCNHIGIVVSGGNVDLAMLWDSLNKGK

6. GLYMA08G01670.2 (GmSR2)

Promoter (2 kb upstream)
AAAACAATGAGCACATAAATATTTATAATAAAAAAAGATAAGTAAGAGTTATTTAATAATTAAT
TACACAAAACTCAAAAGCAACATTTTCTCTGTTTCGTCCAGAATGGACAAAATAGTAAAATTTAA
TGTATTTTTTAATGAATTTTAAGAGGAGAAATAATTAAAATAATTTAATTGATTCAATTTGCAAAA
AATTTATTTTAAAAATAAAGAGCTATAAAATTTTAAATAGAAAAAAAAAATTACTAACAATATTTA
ATTAATTAAAGGATATATTTGAGTAAGTAATCATTTTTGTCTTTAAAAATATATAATATTAACAGA
TTTATTCAAAAAAGATGAAAAAATAAAATTTAATCATGAAAGGATAAAAAATGAGACAAATAT
GTCTCACCTTAATGTTAGAAAACTTGCCTATATGATAAGTAAGATAAAAATGTCATGAATTTAAT
GCTCATATGAACATAAGAATTAACTAAAGTCACTTAGAGATGCATTTCTAATTTAACAACTTATT
AACATTTTAGTACTTCAATTATACAATTTACTCACAAATATATCTATGAAATATATAAATCTAACT
AGCTAGCATCATTAACTATGATACATGTATCAATAACACATTATATTTAGAAGTTTATGATTTTGT
TATTACAAATATTTATTACAATTATAGTTATACTTATAATGTGAACTTATCATAAATAAATCAAGA

CTCAAGAGGAGAAGGTAAGATAAGAGTAAATTTTTAAATAAAAAATTAGTTTATAAAATCTTAA
GAGTGCGTTTATAAAATTTGAATTTATTATAATTTAGAATTCATTGTTTGAATACTTTTTATGAAGA
ATTTAAAATTTTGAAATTTTAAAACAAAATTTTAAATAACTAAAAATCTGGAATTTCAATTTTCTT
TTAGAAGGTGAGAAATTTGAAATTTTCAAGAGGAGTATAACTAGCACACCAATCATATATTTTCT
TTTTTTCATCCTCATAATTTTAATTTTTTTTATCCAAACACAAAAATTTGAAAATAAAAGAATTTCA
TTTAAAGTATTTGAAATTCTTAATTTAAAATTTTTCAAAATTTTAAATTTTTGTAAATGAATTGTGT
AAGTATAAAAGATGTATAGAAACAGAACTAAAATATATTTATAGCTTTCCGGTAATTACCACCTA
TATTATAATAGAAATTGACAAATTATACTTTCAAATCAATCTAATATTTTCATCTTTGATGAATTTG
TTAGCGCACAAGTTTATAGGGAGAAAAATGACTGTTTTTACCTTGGATTAAAGTTGAAGAACTGG
GGTGAGGGTTCATTTCCCCGACCAGAACTGCTTCAGGTTGCCAGCCTGCATTTTCAATTCAACTAG
TAAGTTTCGGATTCTCTTCAAAATCAATACCACATATATAACACTTGTATTTGACTTCGGATTCTCT
TTTCGTTTCACCTGCGGACAACCTTCTGTCAGTTTTTTTTTTTTTATCAGCAAATGTTAGATATTAGA
TTTTTGTTAACCGATGATTGGAACCCTCGACCTCACTCCCTTCGCCACCAAGTCAACCTTATAAGA
CTTTACTAACCGTGGAATACTCCCCTTGTCAAACATTCAACGTTGTACCAATGTTTTTCATTCATGTT
TCTAGGAATTTACCCCACACTTTATTTACCCCCCACACTCTTTTATGCAATTTTAAA
TTTGAGATCATAAGAGAATTAATTGGTACTGTGGGTAGGTAATCTCTATTTTATTTTCGTACAAAT
TCAGATTATGGAACTAACTTACCTTTATGCCTTTGGCTTTGTTGTTGTTGTATATTGCTTGGTAATC
ATGGGACACAATACTAAAATTGTTTGATAGCGTGTTTTTTGTTTAATAGTTAGCTCAATTTCATAG
TGAAAGGAGAAAATTGGTGGATTGGCATTAAACATTGTTTCTGTTGGAAAGACAAATACGCACT
GATCTGTCTTCATTCACTTCTGAGACCAATTACTTAG

CDS

ATGGAAGAAGAGAGCCAAATAACAAAAGGAAAATATGCTGCTGATATTTCTTCCATAAAAGAA
GCACATGCCAGAATCAAATCATTGCTCTCTGACTGCTGTCAGGAAGGCAGCTCTACTTCAAATGT
GAATGTTTACAAAAGGGAAGAAAAGATGATGACACACTTCCTTGTGCATCCTATGGCCATCAGT
GGAGCTTTTAAATTTAGGGGAGCCTGCAATGCTGTGTTCTCTCTCAATGATGAAGATGCTTCTAAA
GGGGTTGTAACACACAGCAGTGGAAACCATGCTGCAGCATTGGCTTTGGCTGCAAAACTACAGG
GGATCCCTTCGTATATTGTTATCCCAAAAAATGTGCCAACTTGCAAAATTGAAAATGTAAAGTGG
TATGGTGGTCAGTTTGTCTGGTCTGAAGCCTCTGTTCAATCAAGGGAAGAAGTTGCCAACAAAGT
GTGGCCAGAAACCGGTGCTATCTTTATACATCCATATAATGATGGACGCAAATTAAGTGGCCAGG
GTACCATATCCTTAGAGATTCTGGAACAAGCACCACAAATAGATATGCTAGTGGTACTCATAAGT
GGTGGTGGTTTGATATCAGGAATAGCATTGGCTGCCAAGTCCATTAACCCAGCTATTCGTATTTTT
GCCGCTGAACCTAAAGGTGCTGATGATGCAGCACAATCTAAAGCAGCTGGGAGGATAATACGAT
TGCCTGAGACCAATACTATAGCTGATGGGCTTCGAGTATTTCTTGGAGATTTGACATGGCCTGTTG
TACAAGATCTTGTTGAGGACATTATAACTGTTGAAGACGGTGAAATAATAAAAGCCATGAAACT
GTTTTTTGAAATTCTTAAGGTTGTTGTAGAACCAAGTGGAGCAATTGGCCTTGCTGCTTTTCTATCC
GATACTTTCCAGAAAAATCCTGCTTGGAAGGATTGTAACCATATAGGAATTGTAATTTCAGGAGG
CAATGTTGATCTGGCCTTGCTTTGGGATTCTTTGAACAAAGGAAAGTGA

Protein

MEEESQITKGKYAADISSIKEAHARIKSLLSDCCQEGSSTSNVNVYKREEKMMTHFLVHPMAISGAFKFR
GACNAVFSLNDEDASKGVVTHSSGNHAAALALAAKLQGIPSYIVIPKNVPTCKIENVKWYGGQFVWS

EASVQSREEVANKVWPETGAIFIHPYNDGRKLSGQGTISLEILEQAPQIDMLVVLISGGGLISGIALAAKSI
NPAIRIFAAEPKGADDAAQSKAAGRIIRLPETNTIADGLRVFLGDLTWPVVQDLVEDIITVEDGEIIKAM
KLFFEILKVVVEPSGAIGLAAFLSDTFQKNPAWKDCNHIGIVISGGNVDLALLWDSLNKGK

7. GLYMA20G32540.2 (GmUC1)

Promoter (2 kb upstream)
TTATTTATGTTTTGTCTAAAAATTTATAACAAATAATGTATTCATTGCAATTAGTAATTAGTGAAG
AAAGCAGCAAGGTTATATTGGTGTGTGTAATGTGTTTAATAGTATCGCTTCACTTGTTATGTTGCG
GATAGATAGCCAAAGTTTCTTGCTCAGATGTTCCAATACAATAATAAAAAAAATTTAAATAGTAG
AAAAATATTATAGAAAATAAATATAACAAAAAACACACATATAATAATAATAATTTATTATTAT
ATAAATAATAATTTCATATATATATATATATATATATATATATATATATATATTAAAATAATACAAAA
ATAAGATATATTTTTATTCACAAATAGCAAGTCTTGTTTAAATTAATTTCAATCATAAATTTATATT
AAAATGTTTATTTTTACCTCAAACATACCTCATTCCATCTCAAAATTTATGACATAAATGTGAGAG
GTATTGCTCTAAAACAGTTGTCATAGAAACAGTTTAGGAGGCAATGAATTTCTTAAACTTACACT
ACTCGCAAGAAGTGATTATTTCTGTGATTTTTTAAAAAGGTTTATTATAATTATTTTATTTTATACT
ATAAATCTAAGATGTTATTTAAAATGTAATGTTTTTTTTTTTACTTTTTTCTAATGAGTTATATATTT
ATTTATCATTTTTAAAAAATTTAAGCTATCAACCAGGTTCAATTTAAAAAATTGAACCTTTTCAAT
TAATTATTTATAAATTTCATATTAATTTTTTCTCTAATTATTTAACCTTTAAATGATTTTTAAAGTTG
TATAATCTTTTTTCTCTTTAAATTTAAACCCTTTATGTTTCTTACTTGCTTGTCTGAATTAATTCTGA
ATATTTATCTTTTAGTTTTAAATATTTTTATAAATTAAATTAAATTAAATCACATGTATCATCCACA
AAATACTTATTAATTTTTATATTATTATTTAGTCGTTATTTTCTTTTGGATAAATTTTTTAACACGAG
TCTTTCACTCACACTAGTTGTGACTTGGGTCTAATTATTAGATTTTTGTTGTGGTTAAGAAATATGT
AAATTTCTCAATAAATTCATATTAAGATTGGTGGTCTTTCTTAGTCTTACAAAGTAATTTTTCTTTT
CCTAATCTAAACTTTTTTAAGTGAAAAAAAAAGGTGGTGTCAAATCATATGATTGGGCGATTATTC
AATTTTTAACCTAAATTTATGCAAATATTTAGATTTAATGACACTTTTAGTCTCTTTATTATCATCA
AAATTTGAATTTTATATTTTCTTTTTGCAAATTTGATTTTCATTATTTGTATAATTATGCTTTTATCTA
TTAAGACTTAAATAATTTTTTAGTCTCTATAAAGTATGATTTTTTTAATTAAAGACCTTTAAAGATT
TTTTTCTTTAGTTCTTTAAAGATTTAAAGTTTTTTATTTTAATTCTTGTTGTAAAGTAATACCGTTAA
TTAATAATGTAATAATGATGTTGATAATTCATTAATTTTTCATATTATCAATGAGTTTGATAGGAT
AACTATAAGTAATTTTTTTGAAAATCTATTTTTAAATTTTCCATTAAGTAGTTTAACCTCATTAATT
TTTTAAAATAAACTAAACCCGAAACTCGTACATTGTACGTAAAAAAGATAAGTAAAAAAGAATT
AATATTTTAAAATGCAAACAAAAAAAAGTGTAAATGCAACAGAGTATGACGCATGGAATTTTGA
TTTGGTGGCTGAACTGATTTTTTATATTTATTGGTAGAGTTTAAAAACGTAATTTATTTAAAAAAA
ATATTATCTCTTATTCCCATGATATATTTTATGCAAATATAAAACTATGTTGTAATTGCATGACCAT
AATTTGTTTTCTTAAGTCTTAATCAAACTCCTTCATTCTTGAGTCCATTTTGATTTCCTCTGCATCTA
AACTCTCTTAATTGCACACACAAGACTCAAGTTACTCCGCACGTGGCACCATTCCACAACCGATG
CTCTA
CDS
ATGTCTGACCCTTCTACCCTCTTACCCTCCTCCCACCACCACCACAACCACCATGTGCCACTCATC
CAAGGCGGCGCCACCGCACCCTCCTCCTCCTCCACCACCCTAGCCCGAGAATACCGCAAGGGCA
ACTGGACCATCCAAGAGACCCTCATTCTCATCACTGCAAAAAAGCTCGACGACGAGCGCAGGCT
CAAAACCCCTGCTGCATGTAGCACCTCCACCACCACCACCAGAACCAGCGGCGAGCTCCGGTGG
AAATGGGTGGAGAACTATTGCTGGAGCCATGGCTGCTTGCGAAGCCAGAACCAATGCAACGACA
AATGGGACAACCTCCTCCGCGATTACAAAAAGGTTCGCGACTACGAGTCCAAATCAAACGACAA
CGACAACAACAACAACAAACACTTCCCTTCTTATTGGACCCTCAACAAACAACAACGTAAGGAA
CAGAACCTCCCCTCCAACATGGTCTTCGAAGTCTACCAAACCATCGCCGATGTCCTCCAACGAAA
ACAAACACAATCCCAAAGACAACATCAACAACCTCTGGCTATTCCATTAGTTACTTCCTCACCCT

CACCACTTCAAACACTTCCACCACCTCCTCTACCACCACCGCCGCCTCCGCCACCTCCTCCTCCGC
CAGTTAGCTCCACCACTCCGGTAGGCTCAGAGAGATCGGAATCATCAGGAACTGAGCACAGTGA
GGATGATGATGATGGGTCGGAATCAAAACGTAGGAAAGTTAAGAACCTTGGTTCAAGAATAATG
CAAAGTGCATCGGTGTTGGCACGAGCTCTTAGGAGTTGTGAGGAGAAGAAGGAGAAACGGCAC
CGTGAAATGATTGAGCTGGAGCAAAGGAGGATTCAGATGGAGGAAGCTCGGAATGAGGTTCAC
CGGCAAGGCATCGCGACCCTTGTCGCCGCCGTCACCAACCTTTCCGGTGCCATTGAGTCACTCAT
TAATAATTCTGAACGCCATGGCCAAAGATAA
Protein

MSDPSTLLPSSHHHHNHHVPLIQGGATAPSSSSTTLAREYRKGNWTIQETLILITAKKLDDERRLKTPAA
CSTSTTTTRTSGELRWKWVENYCWSHGCLRSQNQCNDKWDNLLRDYKKVRDYESKSNDNDNNNNK
HFPSYWTLNKQQRKEQNLPSNMVFEVYQTIADVLQRKQTQSQRQHQQPLAIPLVTSSPSPLQTLPPPPL
PPPPPPPPPPPPVSSTTPVGSERSESSGTEHSEDDDDGSESKRRKVKNLGSRIMQSASVLARALRSCEEKKE
KRHREMIELEQRRIQMEEARNEVHRQGIATLVAAVTNLSGAIESLINNSERHGQR

8. GLYMA17G07200.2 (GmUC2)

Promoter (2 kb upstream)
AACAATCTTATAAATTTTTTGTTAGACACTTTTAATTATTCAAGCCTATTATTTCTTTATATATAATT
TTTTTTTTTATGTTAATGGGTCTAATTCTGAACGAATGCGTTACTGTAAATCATCTAACACTTTTTTT
AATTCACACGGATTAAAAAAATACATTTTTTTCTATCTTATTTCATCGTATCATACACTTTCCTCTT
TGTTATACAAATTTTTGTATAAAGAAATTATACAAACATAATGTCTCATTTTATAATTCCATCTCC
CATGTGCTTGGTCCAGTCAACAATTTGTAAAATCACTGAACCAGGTTGGGAGGGAAGGAGAGAG
AAAGAAAGATAGAATAGAAGAGTAAGTTTGGTCCTAATTAATAATGGCATCGTGCCAAAGACAG
AGTACAGAGCTAGATAGAACTGCTTGTTTCTCCATTCTGGCACTGACTATAGTTGATGAAATTGA
AGGCAAAGTCATCAATACAGATTCAATAGAGTCAGGTCTGTAACTGTTAGTAGTAGTAGTTGTTC
TGTAACTTAACATCACAGACGATTAGAGATATGGATTCAAAGGGACCAGTACGAAATTCACTAC
ATATCTACATTTAGGAATTATGTACAAGCTACCCTGCCGTTTTATATTTATTTGATTCTTTTCAAAG
CGATCGAGGAAGTACGGTAAAAGGTAAATCCGAAAACAAAAAAATTATTAAAGTTAAAGGTTG
TTAAAAGAATTTTATTTTTCTTTCGTTTTTTAAATTTCATTAATTTTTTAGTAAGAGCATTTGAATTT
TAAATATCACATCAATACTTTTTTTTTTTTTTATCTTTTTCTTCTATCACATCACATTATTTATCATAT
ATGTTTTTTTTTCTCTTATTAGGAGTAGAATAATATTTAAGGTGCCTAATTATATTTATTCTTAATTT
TTAAGAGTAGTAATATTTGAATACCTGATAATTTTAAACATCTCACCAATTTTGCTTCTTTTTTTAT
TTTTTCTTTTCCTATTTGTATATTATCTATCACATTTATATTTTTCGTTTTTTTTTTTCTTTCTCATACA
TATACTAAGTGCTGAATAGCATTGTGGGTGTTGAATAACATGTGGATGTCTATAGTCTATCTATAT
TTTCCATTTTTAATCAGCAAGTATAAATACGTACTTCACAGGATAAAAAATAAATAAATACGTAC
TTCATCCTTTAAAAATACACCTGACACACCTTAAAAGATTATTTTTCGGTGGAGGGGTAGATTTGC
TAATTGTCAAGTAATTGCGTGCTTTGTATTGGGTTCGAATCGTAACAATGCACGTTATGTAAATTC
AATTAATTTCAAGGGAAAATATGAATTATTGGTAGGTCTGCTATATATTATTGACTGAATACACC
CAAATTGAGTAGCCTTTGTTCTTATCATATGTACATACATGGGCAGACAGATATACATTGCTTCTA
TATAACTTGATTATTCTACATATAGACTTCGTATCGTAATTCATACGAGGTGTTCATGAGCAGAAT
GCTTCAATATTAGTTAATTAATCTAAGCCGCCGATTTCGTTAAAACGGGTTCATTGTCCAATCCTC
TATGGACAGATAGTGCCAGAGTGAAACATAGGTATAGTACGTCCTTGATTTAAGACAAAAATGT
AAAAATAGTACTCCAATCATGTTCATTGTTTAGTCGAGTCAAGGTGGTTTATCTTGCCCATTTTGT

AATTCCAAAATTGGAACATTGATGGAACACTTCATGATTCGAGTTTAAACTCGTTGTTGACAAGG
GGTCACAACAATGCTAGTTAAATTTTTGTGATTATATGCTTCTTAAATTGAACGTCCACTCATTTTC
AAGACAGAAAACAAAGTTAAAATACAAATAAACCTGATTAATCTTCTTGGTGGGTTTCATACGG
GGTACCCCATAACATTATTTTTTGGCCATCGACCGACAAAGGATCCAAACTCTGCCATTAATGAA
GGCGACAACCTCTTTGATTC

CDS

ATGAATTTCCATAACCCCAATACTTCTCTTCACCTAAGCCAACACGATGATGAGAACCATCTCAA
CCTAGATCTTCTTCTTGAGCCATCTTCTTCTTCTTCATCTTCACCTTCACCTATTTGTTCCATGGAGC
CACGGATATTCTCATGCAACTATTGCCAGAGAAAGTTCTATAGCTCTCAAGCACTAGGGGGTCAC
CAAAATGCCCACAAGCTTGAAAGGACACTGGCCAAGAAGAGCAGGGAACTGAGTTTGAACATG
CAATCTTATGGAGCAGCGGAGCAAAGATCAAACTTTGGTGCTTCACACCATCTTGGTCATCGTGC
TCTTGGGGTTGTGGATAACCAAGGACAAAGTTATGTGAGGCTTGGTGGAAGGAAAGAGTTCAGT
TATGGTTCCAAGGAAGGGGTAGGGGTGGCTTCCTGGTCAAGGGCTTCTGAACATGTTCAGGAGG
ATATGGGCCAACTTGACTTGTCGTTAAGGCTTTGA

Protein

MNFHNPNTSLHLSQHDDENHLNLDLLLEPSSSSSSSPSPICSMEPRIFSCNYCQRKFYSSQALGGHQNA
HKLERTLAKKSRELSLNMQSYGAAEQRSNFGASHHLGHRALGVVDNQGQSYVRLGGRKEFSYGSKEG
VGVASWSRASEHVQEDMGQLDLSLRL

Table S1. Physico-chemical properties of all *GmCAMTA* proteins. (aa- amino acids, MW- molecular weight, pI- isoelectric point, Asp- Aspartate, Glu- Glutamate, Arg- Arginine, Lys- Lysine, II- instability index, AI- aliphatic index, GRAVY- grand average of hydropathy).

Proteins	No. of aa	MW (kDa)	pI	Asp+ Glu	Arg +Lys	No. of atoms	II	AI	GRAVY
Glyma.05g178200 (GmCAMTA1)	1122	126989.43	5.79	159	130	17668	47.68	75.97	-0.625
Glyma.08g135200 (GmCAMTA2)	1102	124429.56	5.66	156	124	17322	43.50	77.79	-0.596
Glyma.15g053600 (GmCAMTA3)	1088	122478.36	5.69	151	118	16999	49.97	76.17	-0.552
Glyma.08g072100 (GmCAMTA4)	1079	121261.43	5.72	148	122	16874	45.99	77.27	-0.522
Glyma.05g117000 (GmCAMTA5)	1088	121941.02	5.58	150	123	16966	45.00	75.90	-0.514
Glyma.08g178900 (GmCAMTA6)	1081	121609.22	5.58	150	117	16890	50.60	77.12	-0.541
Glyma.17g038800 (GmCAMTA7)	999	112283.90	6.81	131	126	15695	40.79	80.39	-0.490
Glyma.15g143400 (GmCAMTA8)	911	103084.74	7.64	105	106	14419	42.07	80.72	-0.428
Glyma.09g038300 (GmCAMTA9)	911	102969.37	6.55	109	103	14385	43.48	80.42	-0.420
Glyma.05g148300 (GmCAMTA10)	983	109314.7	5.43	132	103	15159	49.09	73.92	-0.547
Glyma.18g005100 (GmCAMTA11)	962	108036.77	5.39	137	109	14981	46.47	76.13	-0.520
Glyma.17g031900 (GmCAMTA12)	922	103869.70	7.39	104	104	14503	41.29	81.3	-0.394
Glyma.07g242000 (GmCAMTA13)	921	104293.99	7.03	107	105	14545	41.60	79.60	-0.455
Glyma.11g251900 (GmCAMTA14)	910	102394.56	5.54	130	106	14222	45.31	78.58	-0.531
Glyma.08g105200 (GmCAMTA15)	965	107767.11	5.81	124	104	14945	50.69	73.30	-0.552

Table S2. miRNA potential targets in *GmCAMTA* transcripts. The potential miRNA target sites predicted with the online psRNATarget tool. Keeping a Max. Expectation value = 5, Max UPE= 25. (EV – Expectation Value, M – Multiplicity).

miRNA vs Transcript	Alignment	EV	UPE Target Access	Inhibition	M
gma-miR5780c<br>vs<br>GMCAMTA1	22 AGGUUCAGGGACUAUAUAAUUU 1<br>: : : : : : : : : : : : : : : : . :<br>2541 GGAAAGGCCCUAAUAUAUUAGA 2562	3.0	21.668	Translation	1
gma-miR5780c<br>vs<br>GMCAMTA2	22 AGGUUCAGGGACUAUAUAAUUU 1<br>: : : : : : : : : . : : : : : . :<br>2496 GGAAAGGCCCUAAUAUAUUAGA 2517	3.5	21.158	Translation	1
gma-miR9726<br>vs<br>GMCAMTA3	22 CUUCUUUUUUAUUACGGAUAU 1<br>. : : . : : : . : : : : : : . : : : :<br>965 AGAGGAAAUGAUACUGCUUAUA 986	4.0	18.124	Cleavage	1
gma-miR343b<br>vs<br>GMCAMTA4	24 AGGCUUAGUUGAACUAGACAUUCU 1<br>: : : : : : . : : : . : : : : : . . : :<br>3038 AACCAAUCA--UUUGGUCUGUGGGA 3060	4.0	19.255	Cleavage	1
gma-miR1533<br>vs<br>GMCAMTA5	19 AGUAAUAAUAAAAAUAAUA 1<br>: : : : : : : : : : : : : : :<br>3731 ACAUGAUUAUUUUUAUUUA 3749	4.5	12.197	Cleavage	1
gma-miR5369<br>vs<br>GMCAMTA6	19 ACUGUAGGAGGAAAAGAGU 1<br>: : : : : . . : . . : : : : : : . .<br>3896 UCACAUUUUUUUUUUCUUG 1474	4.0	11.815	Cleavage	1
gma-miR5041-5p<br>vs<br>GMCAMTA7	21 AACUCGUUCAACUUCUACUUU 1<br>: : : : : : : : : : : : : . : .<br>1454 CAGAACAAGUUGAAUAUGGAG 1474	3.5	13.098	Cleavage	1

gma-miR6299	22	ACUGUUUAGUUAUUAAAAUUUA 1				
vs		: ::: . : . . ::: :: :: ::. :	4.0	15.803	Cleavage	1
GMCAMTA8	1507	AGCCAAGUUGUUAAUUUUGAGU 1528				
gma-miR6299	22	ACUGUUUAGUUAUUAAAAUUUA 1				
vs		: : :: . : . . : ::: :: :. : . :	4.0	18.409	Cleavage	1
GMCAMTA9	1562	AGCCAAGUUGUUAAUUUUGAGU 1583				
gma-miR5030	22	GGCCAUUUUGUGUUUAACAAGA 1				
vs		: : : : : : :. : . . : : :: : : :	4.5	18.387	Cleavage	1
GMCAMTA10	3309	UUUGUAAAAUAUGAAAUGGUCU 3330				
gma-miR2111b	21	AUUUGGAGUCCUACGUCUAAU 1				
vs		.: :: :: : :: . :: .: : : : : :	3.5	13.344	Cleavage	1
GMCAMTA11	1488	AGAACCUCUGGGUGUAGAUUC 1508				
gma-miR6299	22	ACUGUUUAGUUAUUAAAAUUUA 1				
vs		: :: : . : . : : : : : : :: . : ::	3.5	14.396	Cleavage	1
GMCAMTA12	1529	AGCCAAGUUGUUAAUUUUGAAU 1550				
gma-miR6299	22	ACUGUUUAGUUAUUAAAAUUUA 1				
vs		: : :: : . . : :: : ::: . : ::	3.5	14.287	Cleavage	1
GMCAMTA13	1507	AGCCAAGUUGUUAAUUUUGAAU 1528				
gma-miR2111b	21	AUUUGGAGUCCUACGUCUAAU 1				
vs		. . :: :: : :: . :: . ::: :::	4.0	15.576	Cleavage	1
GMCAMTA14	1266	AGGACCUCUGGGUGUAGAUUC 1286				
gma-miR1522	20	UAAAGUAAAAUUCGUUAUUU 1				
vs		:: : .:: : .: :: :: :::	5.0	14.966	Cleavage	1
GMCAMTA15	700	UAUUCGUUCUGAACAAUAAA 719				

Table S3. Nuclear localization signal in *GmCAMTA* family. The position, sequence (NLS) and cut off score are shown. *GmCAMTA10* has the highest score of 9.5 while *GmCAMTA3* has the lowest value of 5. Predicted with the online cNLS mapper tool at http://nls-mapper.iab.keio.ac.jp/cgi-bin/NLS_Mapper_form.cgi

Proteins	Pos.	NLS	Score
GmCAMTA1	110	ENFRRRTYWLLEEELSHIVLVHYRHVKGTKA	6.4
GmCAMTA2	38	NYKKFRIAPEPAHMPPSGSLFLFDRKVLRH	6.3
GmCAMTA3	924	RGWKKRKEF	5
GmCAMTA4	110	ENFQRRSYWMLELDMMHIVFVHYLDVKVNKT	5.9
GmCAMTA5	110	ENFQRRSYWMLEPDMMHIVFVHYLDVKVNKT	5.9
GmCAMTA6	920	FRGWKKRQEFLMIRQRVAHVRGHQVRKQYKPI	6.1
GMCAMTA7	35	ILRNHKKFKLT	7.5
GmCAMTA8	794	RGFQARKQYRKIIWSVGVLEKVILRWRLKRKGF	5.2
GmCAMTA9	795	RGFQARKQYRKIVWSVGVLEKVILRWLLKRKGF	5.1
GmCAMTA10	786	SFRKRRAREV	9.5
GmCAMTA11	828	RGYQVRKQYKLILWAVGILDKVVLRWRRKRIGI	5.1
GmCAMTA12	803	RCFQVRKHYCKILWSVGVVEKAVLRWRLKRRGF	6.1
GmCAMTA13	804	RCFQVRKHYRKILWSVGVVEKAVLRWRLKRRGF	6
GmCAMTA14	777	RGYQVRKQYKLILWAVGILDKVVLRWRRKRIGI	5.1
GmCAMTA15	773	SFRKRRARE	7

Table S4. GmCAMTA Protein Interaction Network predicted with STRING database.

Protein	Description
GLYMA14G39640.1	GIBBERELLIN-REGULATED PROTEIN
GLYMA17G07370.1	CBL-INTERACTING SERINE/THREONINE-PROTEIN KINAS
GLYMA19G40910.1	---
GLYMA06G15080.1	Uncharacterized
EDS1	Ehanced disease susceptibility 1
GLYMA03G38310.1	NEFA-INTERACTING NUCLEAR PROTEIN NIP30
GLYMA02G41280	GIBBERELLIN-REGULATED PROTEIN 12-RELATED
GLYMA04G10750.1	SERINE--TRNA LIGASE, MITOCHONDRIAL
GLYMA01G40090.1	BTB AND MATH DOMAIN-CONTAINING PROTEIN 42
GLYMA06G10621.1	Serine--tRNA ligase / Seryl-tRNA synthetase
GLYMA04G38630.1	alpha 1,3-glucosidase (GANAB)
GLYMA19G41740.1	Calmodulin binding protein-like
GLYMA19G41730.1	Calmodulin binding protein-like
GLYMA06G16371.1	Glucan 1,3-alpha-glucosidase / Exo-1,3-alpha-glucanase
ICE2	
GLYMA09G34300.1	EXTRA-LARGE GUANINE NUCLEOTIDE-BINDING PROTEIN 3
GLYMA01G01470.1	EXTRA-LARGE GUANINE NUCLEOTIDE-BINDING PROTEIN 3
GLYMA18G23030.1	SERINE/THREONINE-PROTEIN KINASE RIO1
GLYMA09G30230.2	EXTRA-LARGE GUANINE NUCLEOTIDE-BINDING PROTEIN 3
GLYMA12G15430.2	PHOSPHOLIPASE A1-IIDELTA
GLYMA18G40360.1	Uncharacterized
GLYMA17G07200.2	Uncharacterized
GLYMA20G32540.2	Myb/SANT-like DNA-binding domain (Myb_DNA-bind_4)
GLYMA04G36790.1	elongator complex protein 3 [EC:2.3.1.48] (ELP3, KAT9)
GLYMA08G01670.2	L-serine ammonia-lyase / Serine deaminase
GLYMA05G37930.4	serine racemase (SRR)
GLYMA06G18150.1	elongator complex protein 3 [EC:2.3.1.48] (ELP3, KAT9)
GLYMA06G01080.2	2-OXOGLUTARATE (2OG) AND FE(II)-DEPENDENT OXYGENASE-LIKE PROTEIN-RELATED
GLYMA16G31930.2	RING-H2 FINGER PROTEIN ATL60
GLYMA16G14080.2	G-TYPE LECTIN S-RECEPTOR-LIKE SERINE/THREONINE-PROTEIN KINASE SD1-13
GLYMA20G39240.2	U3-containing 90S pre-ribosomal complex subunit
GLYMA03G04960.1	Protease inhibitor/seed storage/LTP family (Tryp_alpha_amyl)
GLYMA11G11100.1	NPH3 family (NPH3)
GLYMA15G41680.1	CELL CYCLE CONTROL PROTEIN 50 // SUBFAMILY NOT NAMED
GLYMA03G28660.1	TAG lipase / steryl ester hydrolase / phospholipase A2 / LPA acyltransferase (TGL4)
GLYMA12G35680.3	PHD FINGER TRANSCRIPTION FACTOR
GLYMA20G28680.1	POLYPHOSPHATE 5'-PHOSPHATASE-RELATED
GLYMA07G01300.2	CALCINEURIN B-LIKE PROTEIN 10
GLYMA06G42750.1	Fruit bromelain
GLYMA08G19410.2	Premnaspirodiene oxygenase / Hyoscymus muticus premnaspirodiene oxygenase
GLYMA04G42640.1	F-box protein 9 (FBXO9)
GLYMA12G20180.1	PHD FINGER TRANSCRIPTION FACTOR
GLYMA16G30140.1	CGI-141-RELATED/LIPASE CONTAINING PROTEIN // SUBFAMILY NOT NAMED
GLYMA16G04700.1	THIOREDOXIN-LIKE 1-1, CHLOROPLASTIC-RELATED
GLYMA13G34740.1	PHD FINGER TRANSCRIPTION FACTOR
GLYMA06G01180.1	FINGER AND BAH MOTIF CONTAINING PUTATIVE TRANSCRIPTION FACTOR-RELATED
GLYMA06G34850.3	PHD FINGER TRANSCRIPTION FACTOR
GLYMA04G01140.1	FINGER AND BAH MOTIF CONTAINING PUTATIVE TRANSCRIPTION FACTOR-RELATED

Table S5. Primers for qPCR of GmCAMTA family plus internal references.

#	Gene	Primer	Primer Sequence (5` - 3`)	bp	Product size (bp)
1`	*GmCAMTA1*	Forward	GCTACGTCCGGCTGAAATC	19	228
		Reverse	TAGCAATGCAACACATCCACG	21	
2	*GmCAMTA2*	Forward	CTCGGTAATTATAAGAAGTTTCG	23	169
		Reverse	TTAGTCTCTCATGAGCTTCTCTC	23	
3	*GmCAMTA3*	Forward	TTCTTACGGATTGCGTCGTC	20	197
		Reverse	CCTTTCTAAAGTATCTCAAAACC	23	
4	*GmCAMTA4*	Forward	CAGAGATGGATATATCACAACAG	23	179
		Reverse	AGCCCAATCATAACCAAGAG	20	
5	*GmCAMTA5*	Forward	GAAATTATGGTAGTGTGCCTTCT	23	226
		Reverse	TGCCCATGATCACCTGAAAAC	21	
6	*GmCAMTA6*	Forward	TCAATTAAGGATGAAGTGACTAC	23	141
		Reverse	TACATTTGGCCCTTTACCAC	20	
7	*GmCAMTA7*	Forward	CTGAAATATGTGAGATACTTCGC	23	216
		Reverse	AACAATGCAAGACATCCACACTA	23	
8	*GmCAMTA8*	Forward	ATGACCTAGTGGTTCCAAATGT	22	202
		Reverse	CTATCTGGGAAACTGTAAGGG	21	
9	*GmCAMTA9*	Forward	TCACTGAGGTGTCCCCTGT	19	220
		Reverse	CCATCAAAGCTCAAATAGAGAG	22	
10	*GmCAMTA10*	Forward	GAAAGGCTGAAGGTTGGA	18	204
		Reverse	TACACAGAAGAGGAACTTGG	20	
11	*GmCAMTA11*	Forward	TGGTTTATAAGCAAGATAACTCTG	24	180
		Reverse	GCTTACACGATTCCAGCA	18	
12	*GmCAMTA12*	Forward	ATGCAAATGATCATAACACAA	21	160
		Reverse	GTCTCACTCAGCCTATTGCT	20	
13	*GmCAMTA13*	Forward	ATGAACCATATTCGTCTCTGG	21	266
		Reverse	ATTCACAAATCCTGGAGAGTG	21	
14	*GmCAMTA14*	Forward	TGAGTTTGAATGATGATGGC	20	247
		Reverse	TTACACGATTCCAGCACTTTC	21	
15	*GmCAMTA15*	Forward	CTAATCAGAGTTTCTCCAGTCCT	23	305
		Reverse	CCCTTGATCATCGGGTCT	18	
16	*GmActin11*	Forward	ACCGGAATGGTTAAGGCTGG	20	139
		Reverse	ACTGAGCCTCATCACCAACG	20	
17	*GmEF1*	Forward	TGCCGCCAAGAAGAAGTGAT	20	160
		Reverse	ACATGAAATCGGCGGACACT	20	

Table S6. Primers for the qPCR of the GmCAMTA12 orthologue's regulatory network and internal reference in Arabidopsis.

#	Gene/Protein	Primer	Primer Sequence (5` - 3`)	bp	Product size (bp)
1	At3G62140 (NIP30)	Forward	CGAACCAAAGGAAGCAGCAC	20	131
		Reverse	TAGCCTTCTTGGGTTGTGGC	20	
2	At1G30650 (WRKY14	Forward	CGTCGTACGAGGAATCGGAG	20	185
		Reverse	AGCCGGAGTTTGTGATGGAG	20	
3	At1G53720 (CYP59)	Forward	CAAAAGGGCAAAGGGAATGGG	21	132
		Reverse	ATAGCCTTCACCTCCTCCGT	20	
4	At1G68090 (ANN5)	Forward	TTCACTGACCGAAGCAGGAC	20	180
		Reverse	TTTCCTCAATGCCTTTGCGA	20	
5	At4G11640 (SR)	Forward	AGATTTAACCTGGCCGGTGG	20	134
		Reverse	GGCCAATAGCTCCACTTGGT	20	
6	At5G50320 (ELO3)	Forward	GGTTCGAACAGCTTCAGGGA	20	168
		Reverse	TGCCTAGGACAGCTACACCT	20	
7	At3G49050 (CaMHSP)	Forward	TGCCTAGGACAGCTACACCT	20	153
		Reverse	GATACATGCGACCAGGAGCA	20	
8	At4G21390 (B120)	Forward	CAGCTTCGGTGTCTTGTTGC	20	101
		Reverse	ACAAATACCAAGCATAACCAATGA	24	
9	At2G43110 (DEAD BOX Containing Helicase)	Forward	ACCGGGAAACCCATCTGTTC	20	146
		Reverse	ACAGAGATACCTGTTCCTCAACC	20	
10	At3G19850	Forward	CAGGTGGGTCAGATGGGTTC	20	198
		Reverse	CGATGTCGTTCCAGGACCAG	20	
11	At3G12110 (AtActin1)	Forward	ACCGGAATGGTTAAGGCTGG	20	139
		Reverse	ACTGAGCCTCATCACCAACG	20	

Table S7. Primers for the qPCR of GmCAMTA12 regulatory network.

#	Gene/Protein	Primer	Primer Sequence (5′–3′)	bp	Product size (bp)
1	GLYMA19G40910 (NIP30)	Forward	AGATGCGGAATTTAATGAAAGGTTC	25	116
		Reverse	CCACTTGTCGTTCATATTCCCT	22	
2	GLYMA12G15430.2: (GmPLA 1-IId)	Forward	GTCGAAAACGGGGTAACG	18	139
		Reverse	TCAAATCGATCACGTTCTTCACG	23	
3	GLYMA18G40360.1 (GmTAC1)	Forward	TATCCATAGAAAAGATAGGGCGGCA	25	148
		Reverse	ACCAGCACCCAACCGAAACA	20	
4	GLYMA06G18150 (GmECP3)	Forward	CTTGTTGGTTTGTTGCGGCT	20	198
		Reverse	TTCTCTGCAAGCAATGCGCT	20	
5	GLYMA05G37930 (GmSRL)	Forward	TGTTTACAAAAGGGTGGAGCTT	22	100
		Reverse	TGCTGTGTGTTACAACCCCT	20	
6	GLYMA08G01670 (GmSDA)	Forward	GCTGATGATGCAGCACAATCTAAAGC	26	112
		Reverse	CAACAGGCCATGTCAAATCTCCA	20	
7	GLYMA20G32540 (GmASR3)	Forward	GGTGGAAATGGGTGGAGAACTATTG	25	107
		Reverse	GCGAACCTTTTTGTAATCGCG	21	
8	GLYMA17G07200 (GmZFP)	Forward	TAGGGGGTCACCAAAATGCC	20	149
		Reverse	AACCCCAAGAGCACGATGAC	20	
